# Stereospecific Construction of Quaternary Carbon Stereocenters from Quaternary Carbon Stereocenters

**DOI:** 10.1021/jacs.2c01695

**Published:** 2022-04-12

**Authors:** Kaushalendra Patel, Veeranjaneyulu Lanke, Ilan Marek

**Affiliations:** The Mallat Family Laboratory of Organic Chemistry, Schulich Faculty of Chemistry, Technion-Israel Institute of Technology, Haifa 32000, Israel

## Abstract

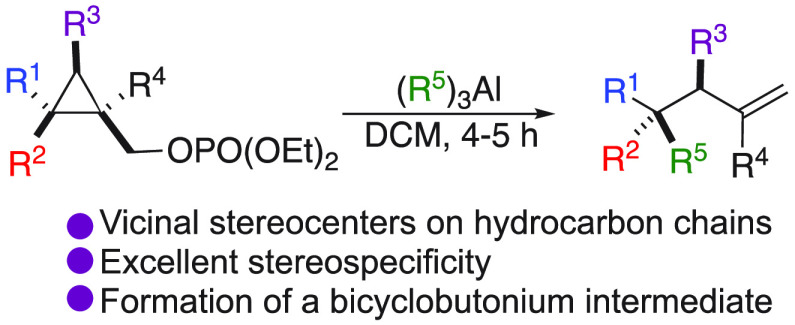

Organoaluminum species promote a smooth nucleophilic substitution at the quaternary carbon stereocenter of stereodefined polysubstituted cyclopropyl methyl phosphate with a complete inversion of configuration, even when more reactive functional groups are present. The regio- and diastereoselectivity of the substitution is attributed to the existence of a bicyclobutonium intermediate.

The stereospecific synthesis of quaternary carbon stereocenter **A**, a molecular fragment found in many natural products,^1^ has constantly aroused the curiosity of organic chemists, leading in the past decade to numerous efficient strategies.^2^ However, if one needs to add an additional vicinal stereocenter next to the quaternary, the proximity of these bulky alkyl chains induces distorted geometries and the diastereocontrol of the overall spatial arrangement of these two stereocenters in acyclic systems becomes more difficult.^3^ Even more challenging is the preparation of vicinal stereocenter **B** that is only constituted of carbons and hydrogen atoms (hydrocarbon chains) and therefore devoid of polar functions (Scheme 1),^4^ which are usually required as a chemical handle to perform selective transformations, eliminating most of the synthetically powerful [3,3]-sigmatropic rearrangements^5^ and enolate approaches.^6^ While the nucleophilic substitution reaction at primary, secondary,^7^ and even at tertiary stereocenters^8^ are well explored with carbon nucleophiles, nucleophilic substitution at quaternary carbon stereocenter with carbon nucleophile has never been considered since the amount of energy required to break a C–C single bond is too high (bond energy 348 kJ/mol). Is, nevertheless, the nucleophilic substitution at a quaternary carbon center a conceivable process? Could we promote a carbon–carbon bond cleavage at a quaternary carbon center with pure inversion of configuration? Could it be used for the preparation of regio-, diastereo-, and even enantiomerically enriched products possessing vicinal stereocenters including a quaternary carbon stereocenter? If the C–C bond is the least reactive functional group, how could a C–C bond cleavage be performed under mild conditions that would allow the presence of more sensitive functional groups? Thrilled by these fundamental questions, we have recently reported a contra-intuitive approach, where nucleophilic substitution at the quaternary carbon stereocenter was indeed a viable concept to create tertiary alcohol and halides with a complete inversion of configuration (Scheme 1).^9^ Actually, cyclopropyl carbinol derivatives could undergo a regio- and stereoselective nucleophilic substitution at the quaternary carbon center C_1_, with pure inversion of configuration, to provide the acyclic products as a single diastereoisomer. The selectivity of the substitution was attributed to the existence of a bicyclobutonium species,^10^ reacting at the most substituted carbon center. Spurred by this remarkable transformation, we implemented this discovery in a newly devised strategy to address the highly challenging and selective preparation of three-dimensional hydrocarbon chains possessing vicinal and congested sp^3^ centers. In this communication, we wish to report the results of this overall synthetic approach, ultimately representing a modular route to efficiently prepare acyclic hydrocarbon chains featuring vicinal congested quaternary and tertiary carbon stereocenters.

**Scheme 1:**
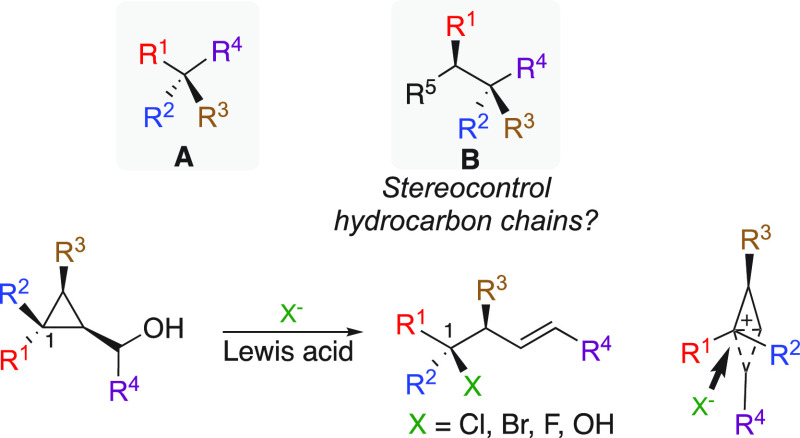
Vicinal Stereocenters and Nucleophilic Substitution at Quaternary Carbon Stereocenter

Based on the selective metal-catalyzed ring opening of cyclopropylcarbinol with heteroatoms,^9^ we recognized the unique potential of these three-membered rings to serve as a central platform to promote highly selective nucleophilic substitution with carbon nucleophiles. Accordingly, a modular and efficient preparation of these starting polysubstituted cyclopropyl carbinols was easily achieved by a diastereoselective copper-catalyzed carbomagnesiation reaction of achiral cyclopropenes (Scheme 2a).^11^ After reaction with CO_2_ and subsequent reduction, the corresponding polysubstituted cyclopropyl carbinols **2a**–**k** were obtained in excellent diastereomeric ratios even when spiro-cyclic products were formed (Scheme 2a).^11^ Among all tested leaving groups, phosphates **3** displayed the right compromise between stability and reactivity.^10^

**Scheme 2:**
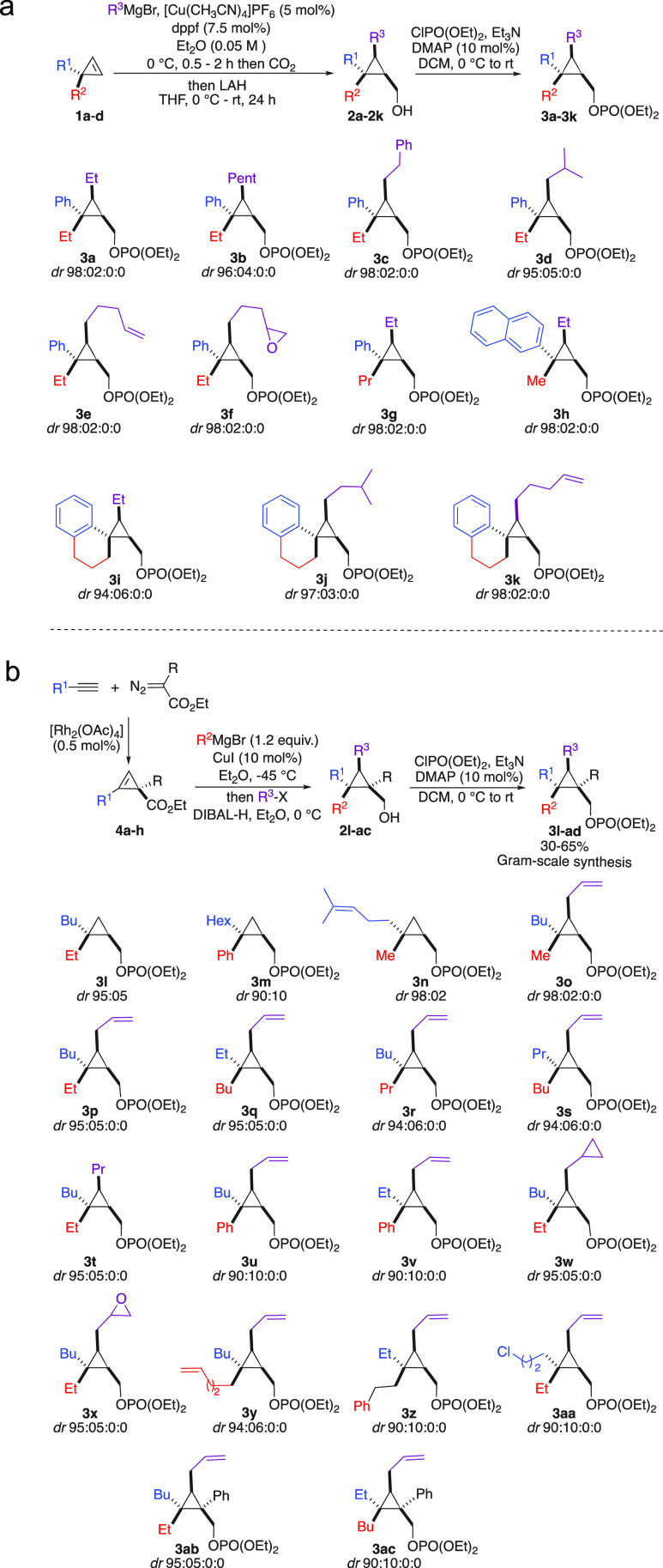
Diastereoselective Preparation of Polysubstituted Cyclopropyl Methyl Phosphates

To further extend the nature of potential groups present on the three-membered rings, we also developed an alternative approach to cyclopropyl methyl phosphates through the copper-catalyzed carbomagnesiation reaction of cyclopropenyl esters **4a**–**4h**,^12^ easily prepared by a [2 + 1]-cycloaddition of Rh-carbene with alkynes,^13^ followed by reaction with various electrophiles (**2l**–**ac**, Scheme 2b).^14^ By a simple reduction of the ester and protection of the resulting alcohol, the substrates bearing the phosphate leaving group (**3l**–**3ac**, Scheme 2b) were easily obtained with excellent diastereomeric ratios in overall yields ranging from 40% to 60%. In this case, the nature of the R^3^ groups originates from the electrophiles and not from the nucleophilic addition as previously discussed in Scheme 2a. It should be noted that, for **3f**, **3t**, **3w**, and **3x**, an additional chemical step was used to transform the alkenyl moieties into their respective functionalized products whereas the Simmons–Smith cyclopropenation reaction^15^ of geraniol was used to prepare **3n** (see Supporting Information). The advantage of the latter strategy (Scheme 2b) is that the electrophile (i.e., allyl bromide) could easily be transformed into valuable functionalized groups; see **3w** and **3x**, Scheme 2b. As a result of these two well-coordinated strategies, a variety of bench-stable diversely polysubstituted cyclopropyl methyl phosphates **3a**–**ac** were quickly assembled, in high stereoisomeric purities. Keys to the success of our strategies were that enantioselective catalytic copper-carbomagnesiation of achiral cyclopropenes (Scheme 2a)^16^ and the enantioselective catalytic [2 + 1] cycloaddition reaction leading to enantiomerically enriched cyclopropenyl ester (Scheme 2b)^13^ have been successfully reported. With these straightforward and general methods for the preparation of **3** in hand, we focused on probing the feasibility of nucleophilic substitution at the quaternary carbon stereocenter with carbon nucleophiles. Following a thorough optimization (see the Supporting Information for all details), it became rapidly clear that a Lewis acid was necessary to promote the reaction while a nucleophile was needed to perform the substitution at the quaternary carbon stereocenter of the cyclopropyl ring. Organoaluminum compound R_3_Al possessing not only a polar C–Al bond but also a high Lewis acidity property was the perfect candidate to perform this transformation (Scheme 3).^17^ Under the optimal conditions, when **3a** was treated with Me_3_Al at low temperature, **5a** was obtained in an excellent yield (90%) and diastereomeric ratio (*dr* 94:06, specificity *sp* 96:04). It is important to note that the nucleophilic substitution occurs exclusively at the quaternary carbon stereocenter, as no trace of substitution was detected at either the primary C_4_ [CH_2_OPO(OEt)_2_] or tertiary C2[C–CHR^3^–C] carbon centers. The relative configuration was determined by X-ray analysis of derivatized **5u**,^18^ and all other configurations were assigned by analogy. Considering the configurations of the starting materials,^11−14^ the nucleophilic substitution at the quaternary carbon center proceeds with a complete inversion of configuration. The nature of the R^3^ group on C_2_ could be varied as longer or branched alkyl chains, and terminus phenyl or alkenyl groups are tolerated (**5b**–**5e**, Scheme 3). More sophisticated molecular backbones in the starting materials possessing spiro moieties such as **3i**–**k** are compatible and proceed selectively at the rather bulky quaternary carbon stereocenter to provide **5k**–**m** in excellent yields and selectivities (Scheme 3). The two diastereomers at the quaternary carbon C_1_ can easily be accessed by simply permuting the nature of the two substituents (**5n** and **5o**, Scheme 3). Interestingly, when **3f** or **3x**, possessing a terminal reactive epoxide functionality, is treated with Me_3_Al, the nucleophilic substitution still proceeds at the quaternary carbon stereocenter without any traces of ring opening to the epoxide (**5f** and **5u**, Scheme 3). This chemoselective transformation (selective carbon–carbon bond cleavage in the presence of an epoxide) illustrates the power of the exclusive formation of the bicyclobutonium species as a reactive intermediate. In this context, **3w** possessing a second cyclopropyl ring on the same molecular backbone but unable to provide the bicyclobutonium remains untouched under this reaction condition (formation of **5v**, Scheme 3). The nature of the two substituents at C_1_ could be successfully varied, and in all cases, hydrocarbon chains possessing the desired quaternary and tertiary stereocenters are obtained with very high stereospecificity (Scheme 3), even when functional groups are present (**5w**, Scheme 3).

**Scheme 3:**
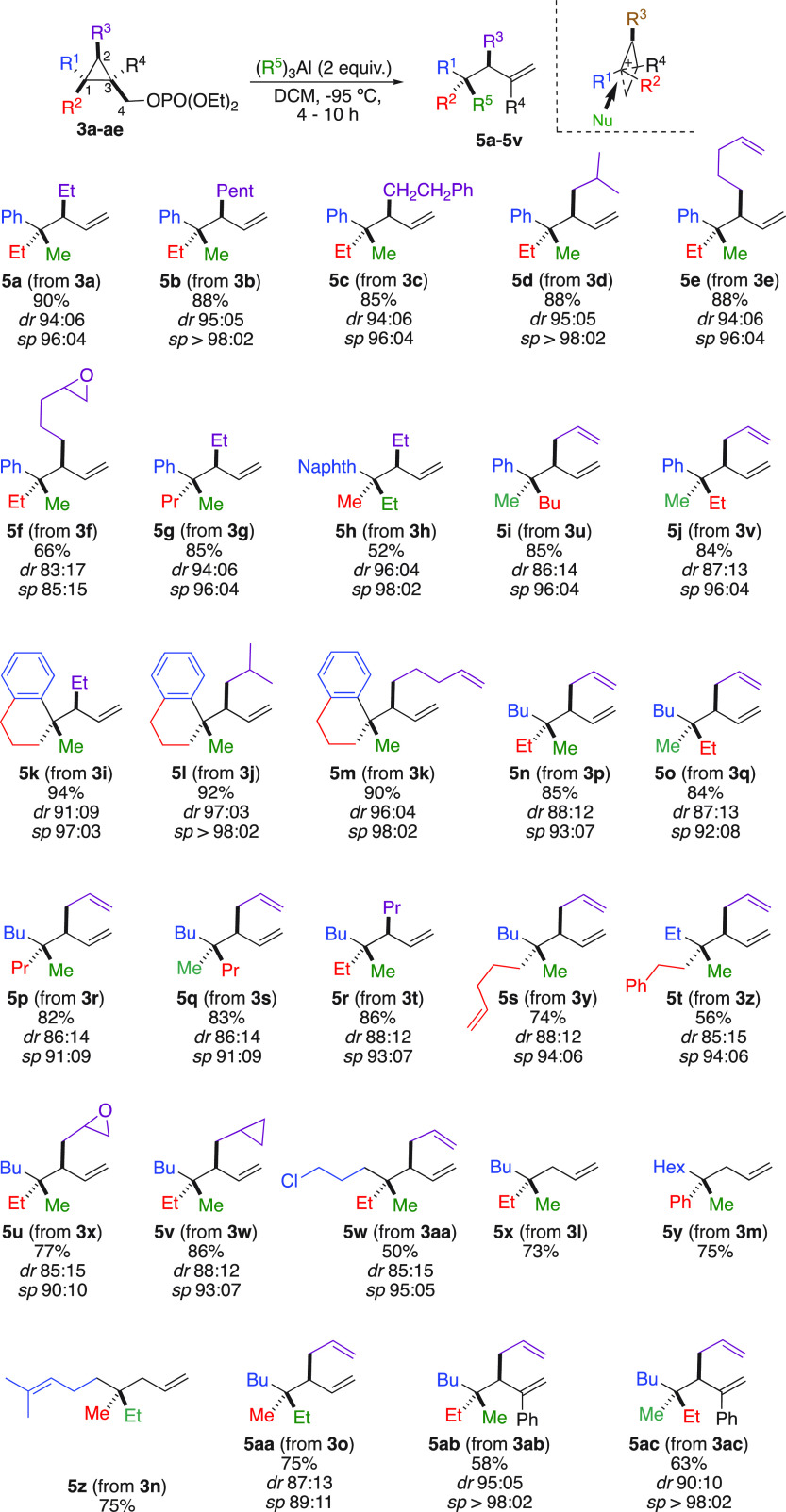
Nucleophilic Substitution at Quaternary Carbon Stereocenters

Remarkably, even when the nucleophilic substitution reaction might have two competitive sites of reaction such as an unsubstituted cyclopropyl carbon center (C_2_) and a fully substituted carbon center (C_1_), the transformation exclusively proceeds on C_1_ to give the products **5x**–**z** in excellent yields.

The reaction is not limited to the addition of Me_3_Al, and the higher Et_3_Al homologue could also be used (**5h**, **5z,** and **5aa**, Scheme 3). Finally, the presence of an additional substituent on C_3_ (R^4^ = Ph) does not impede the reaction to proceed, as the skipped dienes **5ab** and **5ac** were obtained with excellent stereospecificities (Scheme 3). When the copper-catalyzed carbomagnesiation of achiral cyclopropenes **1a** (R^1^ = Ph, R^2^ = Et) and **1d** (R^1^, R^2^ = −C_6_H_4_–(CH_2_)_3_−) were performed with H_2_C=CH–(CH_2_)_3_MgBr in the presence of Cu(CH_3_CN)_4_PF_6_ (5 mol %) and (*R,S*)-Josiphos (7.5 mol %) as a chiral ligand,^16e^ the corresponding cyclopropanes **3e** and **3k** were obtained with moderate enantiomeric ratios of 80:20 and 90:10, respectively.^13^ However, the subsequent addition of Me_3_Al promotes the nucleophilic substitution at the quaternary carbon stereocenter in excellent yield and stereospecificities to give the corresponding two products **5e** and **5m** with the same enantiomeric ratios. It should be noted that the two enantiomers could not be separated by classical HPLC due to their nonpolar nature and lack of functional groups. Hence, the enantiomeric ratios of **5e** and **5m** were determined after subsequent transformation of the less sterically hindered terminal alkene into alcohols using a hydroboration–oxidation sequence (See the Supporting Information).^19^ Based on the observations above, the reaction is both highly enantiospecific (>99%) and diastereoselective, implying that the reaction proceeds without racemization.

To have additional insight on the reaction mechanism, two secondary cyclopropyl phosphates **3ad** and **3ae** were prepared as a mixture of two diastereomers at the secondary protected alcohol moiety C_4_ (Scheme 4).

**Scheme 4:**
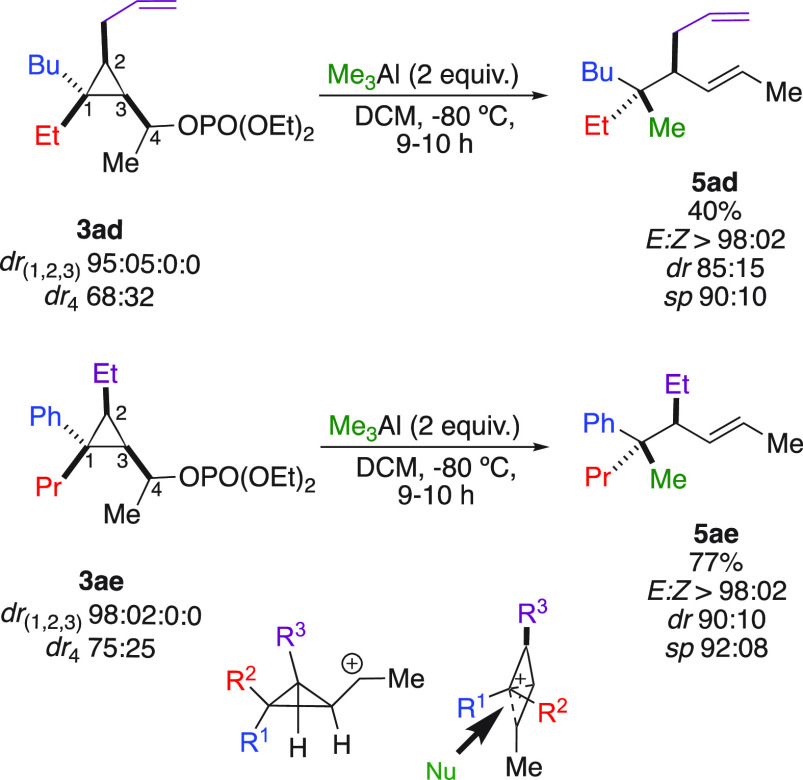
Nucleophilic Substitution of Secondary Cyclopropyl Phosphate

Upon addition of Me_3_Al, two pure *E*-stereodefined hydrocarbon derivatives **5ad** and **5ae** were obtained with excellent stereospecificities. In both cases, direct nucleophilic addition on C_4_ was also observed in 40% and 10% yields respectively. As expected, the nucleophilic substitution proceeding at the most substituted carbon center is independent of the stereochemistry at the starting carbinol center C_4_ suggesting that the reaction proceeds through the formation of an initial cyclopropyl carbocation, best represented as the hybrid bicyclobutonium form (Scheme 4). The addition occurs at the carbon center possessing the highest positive charge and therefore proceeds selectively at the initial quaternary carbon center with a complete inversion of configuration. The unique feature of this bicyclobutonium species is that one face of the transient carbocation is shielded by the molecular backbone, and although it relates to the substitution at the level of intimate ion-pairs, the selectivity of the substitution is therefore easier to control.

In conclusion, organoaluminum species promote smooth nucleophilic substitution at quaternary carbon stereocenters with a complete inversion of configuration, even when more reactive functional groups are present such as epoxides or alkyl halides. The regio- and diastereoselectivity of the substitution is attributed to the existence of a bicyclobutonium intermediate. As a result, otherwise difficult to access, acyclic hydrocarbons are easily prepared from readily available starting materials.

## Supporting Information Available

Experimental procedures, characterization data for all new compounds, along with copies of spectra (PDF)

## Accession Codes

CCDC 2129117 contains the supplementary crystallographic data for this paper. These data can be obtained free of charge via www.ccdc.cam.ac.uk/data_request/cif, or by emailing data_request@ccdc.cam.ac.uk, or by contacting The Cambridge Crystallographic Data Centre, 12 Union Road, Cambridge CB2 1EZ, UK; fax: +44 1223 336033.
