# Supplementary material for: Stereospecific Construction of Quaternary Carbon Stereocenters from Quaternary Carbon Stereocenters
Source: J Am Chem Soc. 2022 Apr 12;144(16):7066–71. doi: 10.1021/jacs.2c01695 (PMC9052742; doi:10.1021/jacs.2c01695)
Supplement: Supplementary file 1 — ja2c01695_si_001.pdf [file ja2c01695_si_001.pdf]

Stereospecific Construction of Quaternary Carbon Stereocenters from Quaternary Carbon Stereocenters

Kaushalendra Patel, Veeranjaneyulu Lanke, and Ilan Marek*

Schulich Faculty of Chemistry. Technion – Israel Institute of Technology, Technion City 3200009, Haifa, Israel.

Table of contents

1	General experimental details	p. 3
2	General Procedure A for the preparation of Polysubstituted Cyclopropyl Methyl Phosphates 3a-3k	p. 4
3	General Procedure B for the preparation of Polysubstituted Cyclopropyl Methyl Phosphates 3l-3ac	p. 4
4	General Procedure C for the preparation of phosphates 3f, 3x	p. 5
5	General Procedure D for the Simmons-Smith cyclopropanation	p. 6
6	General Procedure E for the preparation of secondary Cyclopropyl phosphates 3ad and 3ae	p. 6
7	General Procedure F for Nucleophilic Substitution at the Quaternary Carbon Stereocenters	p. 7
8	Optimization Table 1	p. 7
9	Optimization Table 1	p. 8
10	Characterization data for 2a to 2k	p. 9
11	Characterization data for 3a to 3k	p. 11
12	Characterization data for 2l to 2ae	p. 14
13	Characterization data for 3l to 3ae	p. 17
14	Characterization data for 5a to 5ae	p. 22
15	Determination of the relative configuration	p. 30
16	Determination of the enantiospecificity	p. 33
17	Spectral Data	p. 37
18	References	p. 140

General experimental details

Unless stated otherwise, reactions were conducted in flame-dried glassware under a positive pressure of argon. Ether and THF were dried from Pure-Solv® Purification System (Innovative Technology©). All other commercially obtained reagents were used as received. Dichloromethane was distilled from CaH_2. Copper iodide, rhodium acetate dimer, $AlMe_3$ (2 M in hexane), $AlEt_3$ (1M in hexane) were purchased from Aldrich. $[Cu(MeCN)_4]PF_6$, (R)-(S)-JOSIPHOS and dppf were purchased from Aldrich, all alkyl Grignard reagents were prepared from the corresponding alkyl bromides. Thin-layer chromatography (TLC) was conducted with Merck silica gel 60 F254 pre-coated plates (0.25 mm) and visualized by exposure to UV light (254 nm) or stained with anisaldehyde, phosphomolybdic acid, or potassium permanganate. Column chromatography was performed using Fluka silica gel 60Å (40-63mm, 230-400 mesh). ^{1}H-NMR and ^{13}C-NMR spectra were recorded on a Bruker© spectrometers AVIII400, using $CDCl_3$ (unless otherwise specified) as solvent. Chemical shifts are reported in parts per million (ppm) with respect to the residual solvent signal $CDCl_3$ (^{1}H NMR: δ = 7.26 ppm; ^{13}C NMR: δ = 77.00 ppm). Peak multiplicities are reported as follows: s = singlet, bs = broad singlet, d = doublet, t = triplet, dd = doublet of doublets, td = triplet of doublets, m = multiplet. The GC chromatograms were recorded using Varian© 3800 apparatus with Varian© CP-Sil 8CB® column. High-resolution mass spectra (HRMS) were obtained by the mass spectrometry facility at the Technion-Israel Institute of Technology. Reactions were monitored by gas chromatography spectrometry (GC) using an Agilent Technologies 7820A GC with an Agilent Technologies 19091J-413 (30 m × 0.3 mm) column or (GC-MS) Thermo Scientific TM Ion Trap GC/MS: ITQTM 900 with a Varian Factor Four Capillary column (VF-5 ms, 30m × 0.25mm). Crystal XRD data were collected on a diffractometer Nonius Kappa CCD at Schulich Faculty of Chemistry at Technion-Israel Institute of Technology. Enantiomeric excesses were determined by chiral-HPLC using Agilent© 1100 Series line with CHIRALCEL® OD (0.46 cm Ø×25 cm).

General Procedure A for the preparation of polysubstituted cyclopropyl methyl phosphates 3a-3k

All the starting materials (**3a** to **3k**) were prepared according to the previously developed protocols in our group with slight modifications.[1, 2]

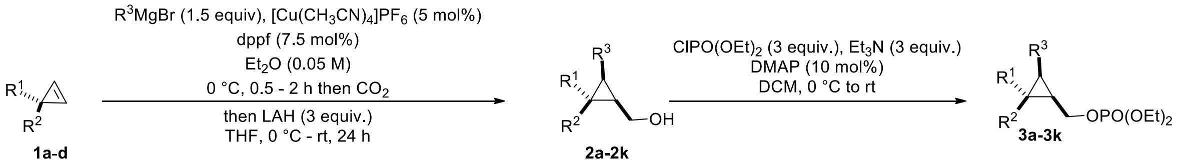

Unless noted otherwise, all reactions were performed on a 2 mmol scale of cyclopropene (**1a-1d**). To a flame dried 10 ml vial fitted with a Teflon stirring bar, $[Cu(MeCN)_4]PF_6$ (5 mol%) and dppf (1,1'-bis(diphenylphosphino)ferrocene) ligand (7.5 mol%) were added, dissolved in Et_2O (10 ml) at room temperature under inert conditions, and stirred for 1 h. After that, to a flame dried and under inert conditions, a three-necked round bottom flask fitted Teflon stirring bar, in Et_2O (10 ml), alkyl magnesium bromide (1.5 equiv.) was added. Then, the yellow copper complex was transferred to the Grignard reagent and allowed to mix for an additional 1 h at room temperature. The reaction mixture was cooled to -5 ºC and the cyclopropene (solution in 2 mL Et_2O) was added dropwise over two minutes. The reaction was followed by TLC analysis of hydrolyzed aliquots (2-3 h). Then the reaction was transferred at -78 ºC and 2 mL of THF was added. The atmosphere of reaction was changed from argon to carbon dioxide (CO_2) and stirred overnight. The reaction was then quenched with an aqueous 1 M solution of HCl. The reaction mixture was extracted twice with Et_2O and the combined organic phases were washed with brine, dried over Na_2SO_4, filtered, and concentrated under reduced pressure. Crude mixtures were then passed through a short pad of silica using petroleum ether/Et_2O as eluent.

After concentration under reduced pressure, the reaction mixture was subjected to a slow addition of LAH (3 equiv.) in THF (0.1 M) at 0 ºC, slowly warmed to room temperature, and stirred for 24 h. After completion of the reaction, it was slowly hydrolyzed with a saturated aqueous solution of sodium sulphate (Na_2SO_4). The organic layer was separated and the white slurry was washed three times with Et_2O. The reaction mixture was extracted twice with Et_2O, dried over Na_2SO_4, filtered, and concentrated under vacuum to give the crude alcohol product which was further purified by column chromatography on silica gel using 20-30% diethyl ether in petroleum ether as eluent (to deliver **2a**-**2k)**.

Preparation of phosphates 3a-3k

To a stirred solution of alcohols **2a**-**2k** (1.0 equiv.) in distilled DCM (0.1M) at 0 °C under argon atmosphere was added Et_3N (3.0 equiv.), DMAP (10 mol%). Then, to this solution was slowly added diethyl chlorophosphate (3.0 equiv.) at 0 °C. The resulting mixture was then stirred at 0 °C until complete consumption of the starting materials (typically 3-4 h), monitored by TLC (stained with PMA). Then, an aqueous solution of NH_4Cl was added and the aqueous layer was extracted twice with DCM. Combined organic layers were washed with brine, dried on Na_2SO_4, filtered and, concentrated under vacuum to give the crude phosphates, which were purified by column chromatography on silica gel using 60-70% diethyl ether in petroleum ether as eluent (to deliver **3a**-**3k)**.

General procedure B for the preparation of polysubstituted cyclopropyl methyl phosphates 3l-3ac

All the starting materials (**3l** to **3ac**) were prepared according to the previously developed protocols in our group with slight modifications.[3, 4]

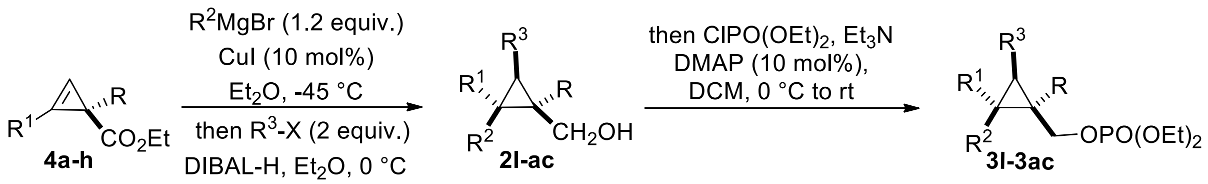

To a stirred solution of cyclopropene **4** (1.0 equiv.) and CuI (0.10 equiv.) in dry Et_2O (0.1M) at -45 °C under argon atmosphere was added the Grignard reagent (1.2 equiv.). The reaction mixture was stirred up to -35 °C for 1.0-1.5 h (TLC monitoring). Then at -35 °C, electrophile (2 equiv. dissolved in Et_2O) was added dropwise. This suspension was stirred for almost 1 h (TLC monitoring) up to -20 °C. Then, a saturated solution of NH_4Cl was added and the aqueous layer was extracted twice with Et_2O. The combined organic layers were washed with brine, dried on Na_2SO_4 and filtered. The volatiles were removed under reduced pressure to give the crude cyclopropyl esters which were further proceeded without purification.

To a stirred solution of crude cyclopropyl esters (1.0 equiv.) in dry Et_2O (0.1 M) at 0 °C under argon atmosphere was added DIBAL-H (2.1 equiv., 1 M in hexane). The reaction mixture was stirred at 0 °C until completion of the starting material (typically 1 h). Then, a saturated solution of potassium sodium tartrate was added and the mixture was stirred for 1 h at rt. Then the aqueous layer was extracted twice with Et_2O. The combined organic layers were washed with brine, dried on Na_2SO_4 and, filtered. The volatiles were removed under reduced pressure to give crude alcohols which were purified by column chromatography on silica gel using 20-30% diethyl ether in petroleum ether as eluent (to deliver **3l-3ad**).

Preparation of phosphates 3l-3ac

As reported for **3a-3k**.

General Procedure C for the preparation of phosphates 3f, 3x

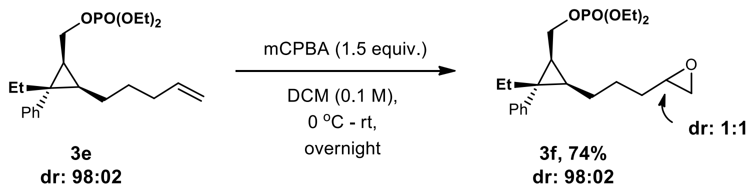

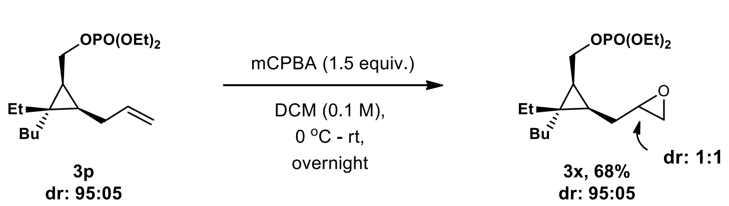

To a stirred solution of phosphates (**3e** or **3p**, 1.0 equiv.) in DCM (0.1 M) at 0 °C, was added *m*CPBA (meta-c hloroperoxybenzoic acid) (1.5 equiv.) in one portion. The reaction was stirred at 0 ºC followed by warming up to room temperature. The reaction mixture was quenched with a saturated solution of $NaHCO_3$. The combined aqueous layer was then extracted twice with DCM. The combined organic layer was dried with Na_2SO_4, filtered, and the volatiles were removed under reduced pressure. The crude product was purified by column chromatography on silica gel using petroleum ether and Et_2O as eluents.

General Procedure D for the Simmons-Smith cyclopropanation[5a]

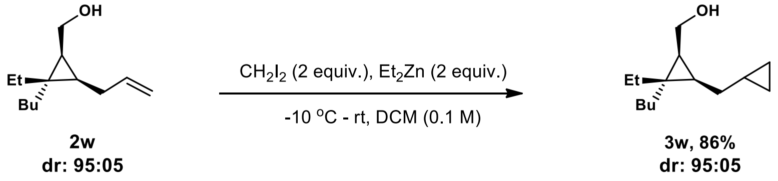

To a stirred solution of alcohol (**2w**, 1.0 equiv.) in DCM (0.1 M) at -10 °C, was added dropwise diethylzinc (2 equiv.) followed by diiodomethane (2 equiv.). The reaction was allowed to warm to room temperature and stirred overnight. The reaction mixture was then quenched with 1 M HCl solution. The combined aqueous layers were then extracted twice with DCM. The layers were separated and the organic layer was then successively washed with a saturated solution of Na_2SO_3 (2 ml), a saturated solution of $NaHCO_3$ (2 ml). The organic layer was dried with Na_2SO_4, filtered, and the volatiles were removed under reduced pressure. The crude product was purified by column chromatography on silica gel using petroleum ether and Et_2O as eluents.

The compound **2n** was prepared from geraniol following literature procedure.[5b]

General Procedure E for the preparation of secondary cyclopropyl phosphates 3ad and 3ae

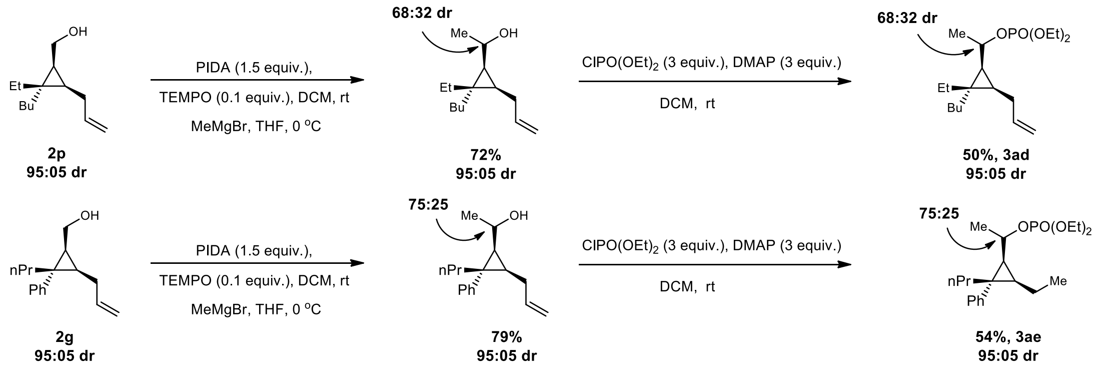

In a round-bottom flask, a solution of cyclopropyl alcohols (**2p** or **2g**, 1 equiv.) and phenyliodine(III) diacetate (PIDA) (1.5 equiv.) in DCM (0.1 M), was added (2,2,6,6-tetramethyl-1-piperidin-1-yl)oxy (TEMPO) (10 mol%) at room temperature. The resulting reaction mixture was stirred for 2 – 4 h (monitored by TLC analysis). The reaction mixture was quenched with a saturated aqueous solution of $Na_2S_2O_3$. The aqueous layer was extracted twice with DCM and the combined organic layers were dried over Na_2SO_4, filtered, and evaporated under vacuum to give the crude aldehyde product which was further purified by column chromatography on silica gel using 5-10% Et_2O/ petroleum ether as eluents. Then, cyclopropyl aldehyde (1 equiv.) was taken in an oven-dried round-bottom flask containing a magnetic stirring bar under argon atmosphere. Anhydrous THF (0.1 M) was added at 0 °C. At the same temperature, methylmagnesium bromide (1.2 equiv.) was added dropwise and the reaction mixture was stirred for an additional 1 h. Upon complete consumption of the starting compound (monitored by TLC), the reaction mixture was then quenched with a saturated aqueous solution of NH_4Cl and extracted with ethyl acetate. The organic layer was dried over Na_2SO_4, filtered, and the volatiles were removed under reduced pressure. The crude product was purified by column chromatography on silica gel using Et_2O/ petroleum ether as eluents.

The phosphorylation of these secondary alcohols was performed according to the general procedure **A** using DMAP (3 equiv.) as a base.

General Procedure F for nucleophilic substitution at the quaternary carbon stereocenters

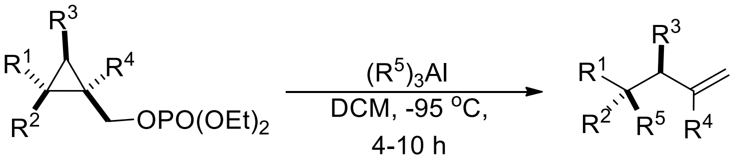

To a stirred solution of phosphates **3a-3ac** (1.0 equiv., 0.3 mmol-0.5 mmol) in dry DCM (0.1 M) at -95 °C under argon was added dropwise (over 2-3 min) a solution of trialkylaluminum (2 equiv.). The resulting mixture was then stirred at -95 °C until the complete consumption of the starting material, the reaction being monitored by TLC (stained with PMA) (typically 8 to 10 h for starting materials **3a-3k, 3ab-3ae** and 4 to 5 h for starting materials **3l-3aa**). Then, a saturated aqueous solution of potassium sodium tartrate was added dropwise and the mixture was stirred 1 h at rt. Then, the aqueous layer was extracted twice with DCM. The combined organic layers were washed with brine, dried on Na_2SO_4, and filtered. The volatiles were removed under reduced pressure to give crudes **5a-5ae** which were purified by flash column chromatography on silica gel (pure pentane as eluent or Et_2O/pentane as eluent).

Note: For the phosphates **3f** and **3x**, 3 equiv. of Me_3Al was used. For phosphates **3ab, 3ac, 3ad,** and **3ae** reaction temperature was maintained at -80 °C.

Optimization Table 1

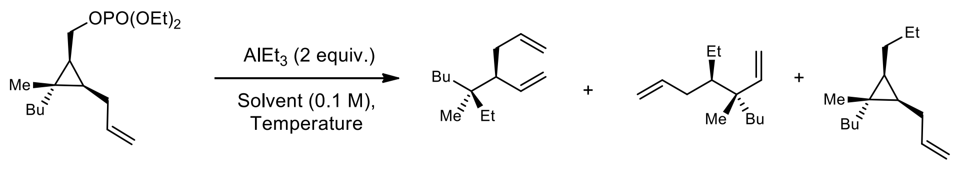

3o
dr 98:02
5aa **B** **C**

Entry[a]	Solvent	T (°C)	Time (h)	Yield (%)[b]	d.r. [c]	**5aa/B/C**
1	DCM	-50 to -30	2.5	85	77:23	85/0/15
2	DCM	-70 to -50	2.5	87	81:19	85/0/15
3	Pentane	-70 to -50	2.5	90	68:32	60/0/40

4	DCM	20	0.5	88	60:40	74/0/26
5	DCM:DCE (1:1)	-50	2.5	88	75:25	87/0/13
6	DCM:Heptane (1:1)	-78	2.5	86	80:20	87/0/13

[a] Reactions were performed on 0.3 mmol scale. [b] crude yields after workup [c] d.r. were determined by ^{13}C NMR

Optimization Table 2

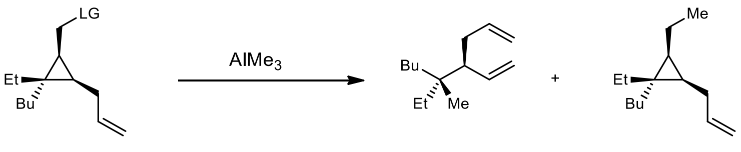

dr: 95:05 5n D

Entry[a]	Solvent	Me$_3$Al (equiv.)	T °C	Time (h)	LG	Yield (%)[b]	d.r. [c]	5n/D
1	DCM	2	-78	1.5	OPO(OEt)$_2$	84	85:15	92/8
2	DCM	2	-90	2.5	OPO(OEt)$_2$	86	86:14	94/6
3	DCM	2	-78	1.5	OPO(OMe)$_2$	84	85:15	92/8
4	DCM	2	-78	1.5	OPO(O^{i}Pr)$_2$	83	85:15	92/8
**5**	**DCM**	**2**	**-95**	**4**	**OPO(OEt)$_2$**	**90**	**88:12**	**96/4**
6	THF	2	-78	2	OPO(OEt)$_2$	0	-	-
7	DCM	1.2	-78	2.5	OPO(OEt)$_2$	80	75:25	90/10
8	DCM	5	-78	1.5	OPO(OEt)$_2$	85	80:20	90/10
9	DCM:Toluene (1:1)	2	-78	1.5	OPO(OEt)$_2$	84	80:20	89/11
10	DCM:Hexane (1:1)	2	-78	1.5	OPO(OEt)$_2$	84	78:22	88/12
11	DCM	2	-78	1.5	OCOCH$_3$	0	-	-
12	DCM	2	-50	1.5	OCOCH$_3$	0	-	-
13	DCM	2	20	0.5	OPO(OEt)$_2$	86	58:42	75/25

[a] Reactions were performed on 0.3 mmol scale. [b] crude yields after workup [c] d.r. were determined by ^{13}C NMR

Determination of diastereomeric ratio

For products **5a**-**5m**, **5ae** the diastereomeric ratios were determined by analysis of the ^{1}H NMR of crude product (also confirmed by analysis of GC-analysis of crude product). For products **5n**-**5w**, **5aa**-**5ac**, and **5ad** the diastereomeric ratios were determined by ^{13}C NMR spectrum of crude product. For the pairs (**5n**, **5o**) and (**5p**, **5q**) both the diastereomers were prepared to confirm the diastereomeric ratio using ^{13}C NMR.[6]

Characterization data for 2a to 2k

((1R*,2R*,3S*)-2,3-diethyl-2-phenylcyclopropyl)methanol (2a)

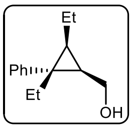

The title compound was prepared according to the general procedure **A**. The product was obtained as a colourless liquid (52%, *dr* 98:02). ^{1}H NMR (400 MHz, Chloroform-*d*) δ 7.20 (d, *J* = 4.3 Hz, 4H), 7.10 (dt, *J* = 8.7, 4.2 Hz, 1H), 3.90 – 3.61 (m, 2H), 1.63 (qd, *J* = 7.3, 5.4 Hz, 2H), 1.49 (ddd, *J* = 14.2, 10.0, 7.2 Hz, 2H), 1.33 (dt, *J* = 9.2, 7.6 Hz, 2H), 1.11 – 1.05 (m, 1H), 1.02 (d, *J* = 7.4 Hz, 3H), 0.71 (t, *J* = 7.4 Hz, 3H); ^{13}C NMR (101 MHz, Chloroform-*d*) δ 147.35, 129.46, 127.99, 125.80, 59.78, 34.12, 29.04, 28.16, 22.72, 17.38, 14.82, 11.57; **HRMS** (APCI) [M-H]$^{+}$, calculated for $C_{14}H_{19}O$; 203.1436; found 203.1462.

((1R*,2R*,3S*)-2-ethyl-3-pentyl-2-phenylcyclopropyl)methanol (2b)

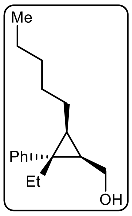

The title compound was prepared according to the general procedure **A**. The product was obtained as a colourless liquid (57%, *dr* 96:04). ^{1}H NMR (400 MHz, Chloroform-*d*) δ 7.19 (d, *J* = 4.4 Hz, 4H), 7.08 (dt, *J* = 8.7, 4.3 Hz, 1H), 3.92 – 3.59 (m, 2H), 1.61 (qd, *J* = 7.2, 5.2 Hz, 2H), 1.49 (dd, *J* = 9.2, 3.0 Hz, 1H), 1.42 (dt, *J* = 11.5, 4.3 Hz, 3H), 1.31 – 1.23 (m, 4H), 1.08 (dt, *J* = 9.1, 6.5 Hz, 1H), 0.84 (td, *J* = 7.2, 2.7 Hz, 3H), 0.70 (t, *J* = 7.3 Hz, 3H); ^{13}C NMR (101 MHz, Chloroform-*d*) δ 147.38, 129.40, 127.95, 125.75, 59.73, 33.94, 31.96, 30.17, 28.04, 27.22, 24.06, 22.75, 22.60, 14.05, 11.51. **HRMS** (APCI) [M-H]$^{+}$, calculated for $C_{17}H_{25}O$; 245.1900; found 245.1900.

((1R*,2R*,3S*)-2-ethyl-3-phenethyl-2-phenylcyclopropyl)methanol (2c)

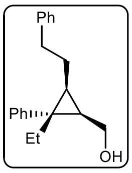

The title compound was prepared according to the general procedure **A**. The product was obtained as a colourless liquid (51%, *dr* 98:02). ^{1}H NMR (400 MHz, Chloroform-*d*) δ 7.46 – 7.18 (m, 10H), 3.94 (dd, *J* = 11.4, 7.5 Hz, 1H), 3.79 (dd, *J* = 11.5, 7.7 Hz, 1H), 2.95 – 2.75 (m, 2H), 2.02 (ddq, *J* = 11.8, 9.0, 6.0, 5.4 Hz, 1H), 1.94 – 1.85 (m, 1H), 1.79 (qd, *J* = 7.3, 2.6 Hz, 2H), 1.53 – 1.47 (m, 1H), 1.35 – 1.29 (m, 1H), 1.01 – 0.92 (m, 1H), 0.87 (t, *J* = 7.3 Hz, 3H); ^{13}C NMR (101 MHz, Chloroform-*d*) δ 147.08, 142.17, 129.36, 128.41, 128.38, 127.99, 125.91, 125.85, 59.46, 36.74, 33.80, 28.12, 26.86, 26.44, 22.79. **HRMS** (APCI) [M-H]$^{+}$, calculated for $C_{20}H_{23}O$; 279.1743; found 279.1734.

((1R*,2R*,3S*)-2-ethyl-3-isobutyl-2-phenylcyclopropyl)methanol (2d)

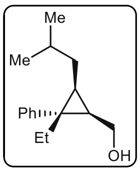

The title compound was prepared according to the general procedure **A**. The product was obtained as a colourless liquid (50%, *dr* 95:05). ^{1}H NMR (400 MHz, Chloroform-*d*) δ 7.23 – 7.15 (m, 4H), 7.11 – 7.05 (m, 1H), 3.88 – 3.63 (m, 2H), 1.65 – 1.56 (m, 3H), 1.47 – 1.32 (m, 2H), 1.23 (dd, *J* = 14.0, 7.1 Hz, 1H), 1.12 (dt, *J* = 9.4, 7.0 Hz, 1H), 0.90 (dd, *J* = 6.6, 1.7 Hz, 6H), 0.85 – 0.74 (m, 1H), 0.69 (t, *J* = 7.3 Hz, 3H); ^{13}C NMR (101 MHz, Chloroform-*d*) δ 147.39, 129.28, 127.96, 125.75, 59.73, 33.68, 32.96, 29.06, 27.98, 25.48, 22.96, 22.83, 22.57, 11.48. **HRMS** (APCI) [M-OH]$^+$, calculated for $C_{16}H_{23}$; 215.1794; found 215.1788.

((1R*,2R*,3S*)-2-ethyl-3-(pent-4-en-1-yl)-2-phenylcyclopropyl)methanol (2e)

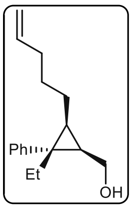

The title compound was prepared according to the general procedure **A**. The product was obtained as a colourless liquid (54%, *dr* 98:02). ^{1}H NMR (400 MHz, Chloroform-*d*) δ 7.22 – 7.16 (m, 4H), 7.12 – 7.06 (m, 1H), 5.77 (ddt, *J* = 16.9, 10.2, 6.7 Hz, 1H), 4.97 (dt, *J* = 17.1, 1.8 Hz, 1H), 4.90 (ddt, *J* = 10.1, 2.2, 1.2 Hz, 1H), 3.85 – 3.69 (m, 2H), 2.13 – 2.02 (m, 2H), 1.62 (p, *J* = 7.1 Hz, 2H), 1.50 (dddd, *J* = 10.9, 8.1, 4.7, 3.2 Hz, 4H), 1.41 – 1.38 (m, 1H), 1.36 – 1.30 (m, 1H), 1.13 – 1.05 (m, 1H), 0.70 (t, *J* = 7.4 Hz, 3H); ^{13}C NMR (101 MHz, Chloroform-*d*) δ 147.27, 138.68, 129.39, 127.99, 125.81, 114.60, 59.72, 33.98, 33.81, 29.71, 28.03, 27.07, 23.60, 22.78, 11.53. **HRMS** (APCI) [M-H]$^+$, calculated for $C_{17}H_{23}O$; 243.1743; found 243.1713.

((1R*,2R*,3S*)-3-ethyl-2-phenyl-2-propylcyclopropyl)methanol (2g)

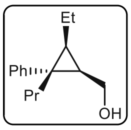

The title compound was prepared according to the general procedure **A**. The product was obtained as a colourless liquid (55%, *dr* 98:02). ^{1}H NMR (400 MHz, Chloroform-*d*) δ 7.22 – 7.09 (m, 4H), 7.06 (td, *J* = 6.2, 3.1 Hz, 1H), 3.98 – 3.53 (m, 2H), 1.97 (s, 1H), 1.59 – 1.39 (m, 4H), 1.35 – 1.23 (m, 1H), 1.11 – 1.05 (m, 2H), 1.01 (td, *J* = 7.4, 1.2 Hz, 4H), 0.70 (td, *J* = 7.3, 1.1 Hz, 3H); ^{13}C NMR (101 MHz, Chloroform-*d*) δ 147.78, 129.23, 127.90, 125.65, 59.55, 33.15, 32.24, 28.88, 28.04, 20.42, 17.36, 14.69, 14.44. **HRMS** (APCI) [M-H]$^+$, calculated for $C_{15}H_{21}O$; 217.1587; found 217.1603.

((1R*,2R*,3S*)-3-ethyl-2-methyl-2-(naphthalen-2-yl)cyclopropyl)methanol (2h)

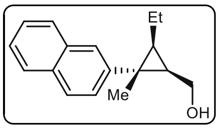

The title compound was prepared according to the general procedure **A**. The product was obtained as a colourless liquid (52%, *dr* 98:02). ^{1}H NMR (400 MHz, Chloroform-*d*) δ 7.71 – 7.62 (m, 3H), 7.59 (d, *J* = 1.8 Hz, 1H), 7.36 – 7.27 (m, 3H), 3.85 – 3.65 (m, 2H), 1.50 – 1.37 (m, 3H), 1.30 (d, *J* = 0.8 Hz, 3H), 1.17 – 1.11 (m, 1H), 1.01 (t, *J* = 7.3 Hz, 3H), 0.84 – 0.70 (m, 1H); ^{13}C NMR (101 MHz, Chloroform-*d*) δ 146.83, 133.43, 131.80, 127.90, 127.48, 127.44, 126.55, 125.86, 125.49, 125.19, 59.75, 29.41, 28.73, 27.59, 17.58, 15.35, 14.59. **HRMS** (APCI) [M-H]$^+$, calculated for $C_{17}H_{19}O$; 239.1430; found 239.1448.

((1R*,2S*,3R*)-2-ethyl-3',4'-dihydro-2'H-spiro[cyclopropane-1,1'-naphthalen]-3-yl)methanol (2i)

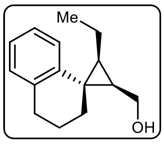

The title compound was prepared according to the general procedure **A**. The product was obtained as a colourless liquid (52%, *dr* 94:06). ^{1}H NMR (400 MHz, Chloroform-*d*) δ 7.03 – 6.98 (m, 1H), 6.97 – 6.91 (m, 2H), 6.62 (d, *J* = 7.9 Hz, 1H), 3.67 (qd, *J* = 11.5, 7.4 Hz, 2H), 2.75 (t, *J* = 6.3 Hz, 2H), 1.76 (ddd, *J* = 6.8, 5.7, 4.4 Hz, 2H), 1.67 (td, *J* = 6.5, 5.9, 2.1 Hz, 2H),

1.48 – 1.35 (m, 3H), 1.16 (td, *J* = 8.1, 7.2, 2.0 Hz, 1H), 0.93 (t, *J* = 7.4 Hz, 3H); ^{13}C NMR (101 MHz, Chloroform-*d*) δ 142.90, 136.91, 128.59, 126.13, 124.41, 121.54, 59.14, 34.27, 33.43, 30.71, 24.75, 23.52, 22.59, 17.36, 14.49. **HRMS** (APCI) [M-H]$^+$, calculated for $C_{15}H_{19}O$; 215.1430; found 215.1428.

((1R*,2S*,3R*)-2-isobutyl-3',4'-dihydro-2'H-spiro[cyclopropane-1,1'-naphthalen]-3-yl)methanol (2j)

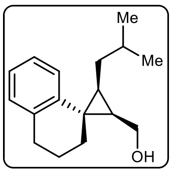

The title compound was prepared according to the general procedure **A**. The product was obtained as a colourless liquid (48%, *dr* 94:06). ^{1}H NMR (400 MHz, Chloroform-*d*) δ 7.13 (ddd, *J* = 8.2, 5.2, 3.6 Hz, 1H), 7.05 (dd, *J* = 4.0, 1.2 Hz, 2H), 6.75 (d, *J* = 7.8 Hz, 1H), 3.89 – 3.72 (m, 2H), 2.86 (t, *J* = 6.3 Hz, 2H), 1.87 (dtd, *J* = 10.9, 6.0, 3.1 Hz, 2H), 1.82 – 1.74 (m, 2H), 1.65 (dt, *J* = 13.1, 6.6 Hz, 1H), 1.56 – 1.40 (m, 3H), 1.38 – 1.25 (m, 3H), 0.95 (d, *J* = 3.3 Hz, 3H), 0.94 (d, *J* = 3.3 Hz, 3H); ^{13}C NMR (101 MHz, Chloroform-*d*) δ 142.85, 137.08, 128.68, 126.22, 124.51, 121.57, 59.58, 33.67, 32.93, 30.83, 30.74, 28.88, 24.59, 23.89, 22.84, 22.63, 22.52. **HRMS** (APCI) [M-OH]$^+$, calculated for $C_{17}H_{23}$; 227.1794; found 227.1790.

((1R*,2S*,3R*)-2-(pent-4-en-1-yl)-3',4'-dihydro-2'H-spiro[cyclopropane-1,1'-naphthalen]-3-yl)methanol (2k)

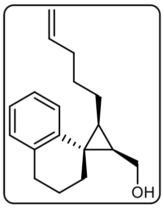

The title compound was prepared according to the general procedure **A**. The product was obtained as a colourless liquid (56%, *dr* 98:02). ^{1}H NMR (400 MHz, Chloroform-*d*) δ 7.17 – 7.10 (m, 1H), 7.07 (d, *J* = 4.4 Hz, 2H), 6.75 (d, *J* = 7.9 Hz, 1H), 5.84 (ddt, *J* = 16.8, 10.1, 6.6 Hz, 1H), 5.09 – 4.95 (m, 2H), 3.79 (qd, *J* = 11.5, 7.4 Hz, 2H), 2.88 (t, *J* = 6.3 Hz, 2H), 2.12 (td, *J* = 7.4, 7.0, 4.0 Hz, 2H), 1.95 – 1.85 (m, 2H), 1.79 (t, *J* = 5.4 Hz, 3H), 1.63 – 1.45 (m, 5H), 1.34 – 1.29 (m, 1H); ^{13}C NMR (101 MHz, Chloroform-*d*) δ 142.77, 138.58, 136.95, 128.61, 126.15, 124.45, 121.51, 114.51, 59.20, 33.64, 33.45, 32.26, 30.69, 29.23, 24.69, 23.62, 23.57, 22.55. **HRMS** (APCI) [M+H]$^+$, calculated for $C_{18}H_{25}O$; 257.1900; found 257.1901.

Characterization data for 3a to 3k

((1R*,2R*,3S*)-2,3-diethyl-2-phenylcyclopropyl)methyl diethyl phosphate (3a)

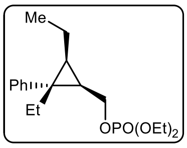

The title compound was prepared according to the general procedure **A**. The product was obtained as a colourless liquid (86%, *dr* 98:02). ^{1}H NMR (400 MHz, Chloroform-*d*) δ 7.20 (d, *J* = 4.4 Hz, 4H), 7.11 (q, *J* = 4.4 Hz, 1H), 4.22 (ddd, *J* = 8.4, 6.4, 1.8 Hz, 2H), 4.08 (p, *J* = 7.3 Hz, 4H), 1.64 (ddd, *J* = 11.4, 7.9, 4.3 Hz, 2H), 1.59 – 1.39 (m, 3H), 1.29 (tt, *J* = 7.1, 1.4 Hz, 6H), 1.13 (dd, *J* = 9.3, 7.4 Hz, 1H), 1.03 (t, *J* = 7.3 Hz, 3H), 0.70 (t, *J* = 7.3 Hz, 3H); ^{13}C NMR (101 MHz, Chloroform-*d*) δ 146.85, 129.47, 128.00, 125.95, 65.61 (d, *J* = 5.7 Hz), 63.66 (d, *J* = 5.9 Hz), 34.49, 29.27, 25.52 (d, *J* = 8.8 Hz), 22.70, 17.36, 16.15 (d, *J* = 6.8 Hz), 14.69, 11.40; ^{31}P NMR (162 MHz, Chloroform-*d*) δ -0.64. **HRMS** (APCI) [M]$^+$, calculated for $C_{18}H_{29}O_4P$; 340.1798; found 340.1788.

Diethyl (((1R*,2R*,3S*)-2-ethyl-3-(pent-4-en-1-yl)-2-phenylcyclopropyl)methyl) phosphate (3b)

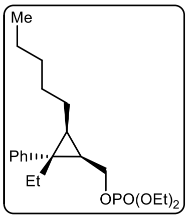

The title compound was prepared according to the general procedure **A**. The product was obtained as a colourless liquid (82%, *dr* 96:04). ^{1}H NMR (400 MHz, Chloroform-*d*) δ 7.28 (d, *J* = 4.3 Hz, 4H), 7.19 (q, *J* = 4.5 Hz, 1H), 4.29 (dt, *J* = 8.5, 6.4 Hz, 2H), 4.21 – 4.09 (m, 4H), 1.77 – 1.66 (m, 2H), 1.53 – 1.45 (m, 4H), 1.40 – 1.34 (m, 9H), 1.30 – 1.19 (m, 2H), 0.96 – 0.84 (m, 4H), 0.78 (t, *J* = 7.3 Hz, 3H); ^{13}C NMR (101 MHz, Chloroform-*d*) δ 146.86, 129.42, 127.96, 125.91, 65.65 (d, *J* = 5.7 Hz), 63.62 (d, *J* = 5.9 Hz), 34.32, 31.89, 30.05, 27.43, 25.43 (d, *J* = 8.9 Hz), 24.04, 22.66 (d, *J* = 14.7 Hz), 16.12 (d, *J* = 6.7 Hz), 14.03, 11.34; ^{31}P NMR (162 MHz, Chloroform-*d*) δ -0.64. **HRMS** (APCI) $[M-H]^+$, calculated for $C_{21}H_{35}O_4P$; 381.2189; found 381.2220.

Diethyl (((1R*,2R*,3S*)-2-ethyl-3-phenethyl-2-phenylcyclopropyl)methyl) phosphate (3c)

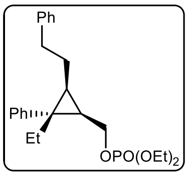

The title compound was prepared according to the general procedure **A**. The product was obtained as a colourless liquid (85%, *dr* 98:02). ^{1}H NMR (400 MHz, Chloroform-*d*) δ 7.36 – 7.14 (m, 10H), 4.35 – 4.05 (m, 6H), 2.80 (t, *J* = 7.9 Hz, 2H), 2.00 – 1.88 (m, 1H), 1.81 (ddd, *J* = 13.6, 8.2, 6.8 Hz, 1H), 1.72 (q, *J* = 7.4 Hz, 2H), 1.59 – 1.51 (m, 1H), 1.36 (tt, *J* = 7.1, 1.1 Hz, 6H), 1.32 – 1.26 (m, 1H), 0.79 (t, *J* = 7.4 Hz, 3H); ^{13}C NMR (101 MHz, Chloroform-*d*) δ 146.49, 141.89, 129.34, 128.33, 128.31, 127.96, 125.97, 125.85, 65.37 (d, *J* = 5.6 Hz), 63.61 (d, *J* = 5.9 Hz), 36.51, 34.36, 27.01, 26.36, 25.51 (d, *J* = 8.9 Hz), 22.74, 16.09 (d, *J* = 6.7 Hz), 11.36; ^{31}P NMR (162 MHz, Chloroform-*d*) δ -0.64. **HRMS** (APCI) $[M]^+$, calculated for $C_{24}H_{33}O_4P$; 416.2111; found 416.2112.

Diethyl (((1R*,2R*,3S*)-2-ethyl-3-isobutyl-2-phenylcyclopropyl)methyl) phosphate (3d)

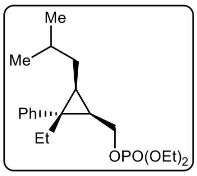

The title compound was prepared according to the general procedure **A**. The product was obtained as a colourless liquid (85%, *dr* 95:05). ^{1}H NMR (400 MHz, Chloroform-*d*) δ 7.22 – 7.16 (m, 4H), 7.09 (ddd, *J* = 5.8, 4.7, 2.7 Hz, 1H), 4.27 – 4.12 (m, 2H), 4.07 (tt, *J* = 8.2, 6.6 Hz, 4H), 1.61 (pd, *J* = 7.5, 3.2 Hz, 3H), 1.50 – 1.39 (m, 2H), 1.28 (tdd, *J* = 7.1, 2.0, 1.0 Hz, 6H), 1.24 – 1.15 (m, 2H), 0.90 (dd, *J* = 6.7, 2.5 Hz, 6H), 0.69 (t, *J* = 7.3 Hz, 3H); ^{13}C NMR (101 MHz, Chloroform-*d*) δ 146.74, 129.21, 127.89, 125.83, 65.58 (d, *J* = 5.7 Hz), 63.52 (d, *J* = 5.9 Hz), 33.96, 32.78, 28.85, 25.58, 25.32 (d, *J* = 8.9 Hz), 22.73, 22.45, 16.01 (d, *J* = 6.6 Hz), 11.20; ^{31}P NMR (162 MHz, Chloroform-*d*) δ -0.68. **HRMS** (APCI) $[M-H]^+$, calculated for $C_{20}H_{33}O_4P$; 367.2033; found 367.2073.

Diethyl (((1R*,2R*,3S*)-2-ethyl-3-pentyl-2-phenylcyclopropyl)methyl) phosphate (3e)

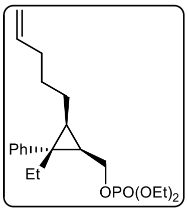

The title compound was prepared according to the general procedure **A**. The product was obtained as a colourless liquid (87%, *dr* 98:02). ^{1}H NMR (400 MHz, Chloroform-*d*) δ 7.19 (d, *J* = 4.4 Hz, 4H), 7.13 – 7.07 (m, 1H), 5.76 (ddt, *J* = 16.9, 10.2, 6.7 Hz, 1H), 5.02 – 4.83 (m, 2H), 4.20 (ddd, *J* = 8.0, 6.4, 1.1 Hz, 2H), 4.13 – 4.00 (m, 4H), 2.06 (td, *J* = 7.5, 3.7 Hz, 2H), 1.62 (qd, *J* = 7.2, 4.7 Hz, 2H), 1.57 – 1.38 (m, 5H), 1.28 (tt, *J* = 7.1, 1.4 Hz, 6H), 1.19 – 1.12 (m, 1H), 0.70 (t, *J* = 7.4 Hz, 3H); ^{13}C NMR (101 MHz, Chloroform-*d*) δ 146.67, 138.48, 129.32, 127.93, 125.90, 114.55, 65.50 (d, *J* = 5.6 Hz), 63.57 (d, *J* = 5.9 Hz), 34.29, 33.69, 29.50, 27.21, 25.35 (d, *J* = 8.9 Hz), 23.50, 22.69, 16.06 (d, *J* = 6.8 Hz), 11.29; ^{31}P NMR (162 MHz, Chloroform-*d*) δ -0.65. **HRMS** (APCI) $[M]^+$, calculated for $C_{21}H_{35}O_4P$; 380.2116; found 380.2094.

Diethyl (((1R*,2R*,3S*)-2-ethyl-3-(3-(oxiran-2-yl)propyl)-2-phenylcyclopropyl)methyl) phosphate (3f)

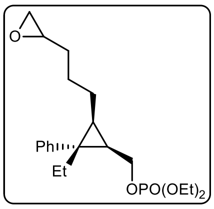

The title compound was prepared according to the general procedure **C**. The product was obtained as a colourless liquid (74%, *dr* 98:02). ^{1}H NMR (400 MHz, Chloroform-*d*) (*mixture of diastereomers*) δ 7.20 (d, *J* = 5.7 Hz, 4H), 7.11 (td, *J* = 5.9, 3.1 Hz, 1H), 4.21 (dd, *J* = 8.0, 6.5 Hz, 2H), 4.12 – 4.04 (m, 4H), 2.91 – 2.84 (m, 1H), 2.70 (dd, *J* = 5.0, 3.9 Hz, 1H), 2.43 (dd, *J* = 5.0, 2.7 Hz, 1H), 1.68 – 1.46 (m, 8H), 1.29 (tt, *J* = 7.1, 1.3 Hz, 6H), 1.17 (q, *J* = 8.7, 7.9 Hz, 2H), 0.70 (t, *J* = 7.3 Hz, 3H); ^{13}C NMR (101 MHz, Chloroform-*d*) (*mixture of diastereomers*) δ 146.55, 129.32, 127.98, 125.98, 65.43 (d, *J* = 5.6 Hz) & 65.12 (t, *J* = 3.0 Hz), 63.63 (d, *J* = 5.9 Hz), 52.15 & 52.08, 46.95 & 46.93, 34.33, 32.45 & 32.37, 30.21, 27.26 & 27.22, 26.72 (d, *J* = 8.7 Hz) & 25.35 (d, *J* = 8.9 Hz), 23.96 & 23.88, 22.72, 16.09 (d, *J* = 6.7 Hz) & 15.89 (t, *J* = 3.6 Hz), 11.32; ^{31}P NMR (162 MHz, Chloroform-*d*) (*mixture of diastereomers*) δ -0.66. **HRMS** (APCI) $[M+H]^+$, calculated for $C_{21}H_{34}O_5P$; 397.2138; found 397.2116.

Diethyl (((1R*,2R*,3S*)-3-ethyl-2-phenyl-2-propylcyclopropyl)methyl) phosphate (3g)

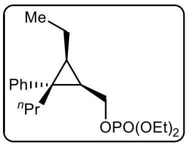

The title compound was prepared according to the general procedure **A**. The product was obtained as a colourless liquid (85%, *dr* 98:02). ^{1}H NMR (400 MHz, Chloroform-*d*) δ 7.20 (d, *J* = 4.2 Hz, 4H), 7.10 (dt, *J* = 8.8, 4.3 Hz, 1H), 4.21 (dd, *J* = 8.0, 6.4 Hz, 2H), 4.08 (tt, *J* = 7.9, 6.6 Hz, 4H), 1.57 – 1.41 (m, 5H), 1.33 – 1.26 (m, 6H), 1.10 (ddt, *J* = 10.5, 8.5, 7.2 Hz, 3H), 1.03 (t, *J* = 7.4 Hz, 3H), 0.72 (t, *J* = 7.3 Hz, 3H); ^{13}C NMR (101 MHz, Chloroform-*d*) δ 147.30, 129.34, 128.01, 125.93, 65.67 (d, *J* = 5.5 Hz), 63.67 (d, *J* = 6.0 Hz), 33.68, 32.29, 29.22, 25.52 (d, *J* = 8.8 Hz), 20.36, 17.43, 16.17 (d, *J* = 6.8 Hz), 14.66, 14.48; ^{31}P NMR (162 MHz, Chloroform-*d*) δ -0.64. **HRMS** (APCI) $[M+H]^+$, calculated for $C_{19}H_{32}O_4P$; 355.2033; found 355.2041.

Diethyl (((1R*,2R*,3S*)-3-ethyl-2-methyl-2-(naphthalen-1-yl)cyclopropyl)methyl) phosphate (3h)

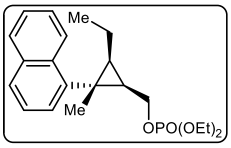

The title compound was prepared according to the general procedure **A**. The product was obtained as a colourless liquid (90%, *dr* 98:02). ^{1}H NMR (400 MHz, Chloroform-*d*) δ 6.52 (td, *J* = 8.4, 7.3, 3.8 Hz, 3H), 6.43 (d, *J* = 1.8 Hz, 1H), 6.23 – 6.11 (m, 3H), 3.17 – 3.09 (m, 1H), 3.03 – 2.95 (m, 1H), 2.90 (dq, *J* = 8.0, 7.1 Hz, 4H), 0.50 – 0.46 (m, 1H), 0.40 – 0.27 (m, 3H), 0.17 (s, 3H), 0.11 (td, *J* = 7.1, 1.0 Hz, 6H), 0.08 – 0.02 (m, 1H), -0.13 (t, *J* = 7.3 Hz, 3H); ^{13}C NMR (101 MHz, Chloroform-*d*) δ 146.39, 133.43, 131.92, 127.99, 127.53, 127.51, 126.72, 125.95, 125.75, 125.34, 65.75 (d, *J* = 5.6 Hz), 63.70 (d, *J* = 5.9 Hz), 29.48, 28.32, 26.00 (d, *J* = 8.5 Hz), 17.64, 16.17 (d, *J* = 6.7 Hz), 15.65, 14.53; ^{31}P NMR (162 MHz, Chloroform-*d*) δ -0.56. **HRMS** (APCI) $[M]^+$, calculated for $C_{19}H_{32}O_4P$; 376.1798; found 376.1807.

Diethyl (((1R*,2S*,3R*)-2-ethyl-3',4'-dihydro-2'H-spiro[cyclopropane-1,1'-naphthalen]-3-yl)methyl) phosphate (3i)

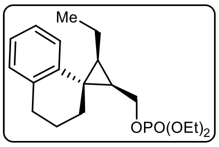

The title compound was prepared according to the general procedure **A**. The product was obtained as a colourless liquid (82%, *dr* 94:06). ^{1}H NMR (400 MHz, Chloroform-*d*) δ 7.14 – 7.06 (m, 1H), 7.03 (dd, *J* = 4.0, 0.9 Hz, 2H), 6.70 (d, *J* = 7.9 Hz, 1H), 4.34 – 4.25 (m, 1H), 4.19 (ddd, *J* = 11.1, 8.1, 7.3 Hz, 1H), 4.14 – 4.00 (m, 4H), 2.83 (t, *J* = 6.2 Hz, 2H), 1.95 – 1.76 (m, 4H), 1.65 – 1.45 (m, 3H), 1.28 (dtd, *J* = 14.0, 7.1, 1.0 Hz, 7H), 1.02 (t, *J* = 7.4 Hz, 3H); ^{13}C NMR

(101 MHz, Chloroform-*d*) δ 142.05, 136.90, 128.50, 126.02, 124.50, 121.39, 64.86 (d, *J* = 5.6 Hz), 33.99, 30.49 (t, *J* = 4.4 Hz), 25.10, 23.43, 22.26, 17.21, 16.51 – 15.80 (m), 14.24; ^{31}P NMR (162 MHz, Chloroform-*d*) δ -0.75. **HRMS** (APCI) [M-H]$^+$, calculated for $C_{19}H_{28}O_4P$; 351.1720; found 351.1767.

Diethyl (((1R*,2S*,3R*)-2-isobutyl-3',4'-dihydro-2'H-spiro[cyclopropane-1,1'-naphthalen]-3-yl)methyl) phosphate (3j)

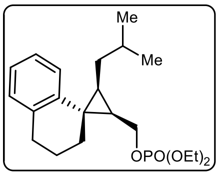

The title compound was prepared according to the general procedure **A**. The product was obtained as a colourless liquid (80%, *dr* 97:03). ^{1}H NMR (400 MHz, Chloroform-*d*) δ 7.16 – 7.07 (m, 1H), 7.07 – 7.00 (m, 2H), 6.72 (d, *J* = 7.8 Hz, 1H), 4.28 (ddd, *J* = 11.0, 7.1, 5.8 Hz, 1H), 4.18 – 4.02 (m, 5H), 2.84 (t, *J* = 6.2 Hz, 2H), 1.95 – 1.79 (m, 2H), 1.76 (td, *J* = 5.9, 5.0, 1.4 Hz, 2H), 1.67 – 1.54 (m, 2H), 1.47 – 1.40 (m, 1H), 1.37 – 1.24 (m, 8H), 0.96 – 0.89 (m, 6H); ^{13}C NMR (101 MHz, Chloroform-*d*) δ 142.23, 137.22, 128.69, 126.20, 124.67, 121.55, 65.17 (d, *J* = 5.7 Hz), 63.62 (d, *J* = 5.9 Hz), 32.82, 30.76 (d, *J* = 8.8 Hz), 30.64 (d, *J* = 2.0 Hz), 28.76, 25.00, 23.86, 22.73, 22.59, 22.25, 16.06 (t, *J* = 6.4 Hz); ^{31}P NMR (162 MHz, Chloroform-*d*) δ -0.73. **HRMS** (APCI) [M]$^+$, calculated for $C_{21}H_{33}O_4P$; 380.2116; found 380.2091.

Diethyl (((1R*,2S*,3R*)-2-(pent-4-en-1-yl)-3',4'-dihydro-2'H-spiro[cyclopropane-1,1'-naphthalen]-3-yl)methyl) phosphate (3k)

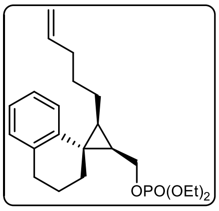

The title compound was prepared according to the general procedure **A**. The product was obtained as a colourless liquid (89%, *dr* 98:02). ^{1}H NMR (400 MHz, Chloroform-*d*) δ 7.11 (dt, *J* = 8.1, 4.4 Hz, 1H), 7.04 (d, *J* = 4.2 Hz, 2H), 6.71 (d, *J* = 7.9 Hz, 1H), 5.79 (ddt, *J* = 16.9, 10.2, 6.6 Hz, 1H), 5.07 – 4.89 (m, 2H), 4.32 – 4.23 (m, 1H), 4.18 (dt, *J* = 11.1, 7.6 Hz, 1H), 4.14 – 4.00 (m, 4H), 2.84 (t, *J* = 6.1 Hz, 2H), 2.08 (q, *J* = 7.1 Hz, 2H), 1.93 – 1.82 (m, 2H), 1.78 (dt, *J* = 6.5, 3.7 Hz, 2H), 1.63 (dd, *J* = 9.3, 7.8 Hz, 1H), 1.58 – 1.42 (m, 4H), 1.35 – 1.25 (m, 7H); ^{13}C NMR (101 MHz, Chloroform-*d*) δ 142.13, 138.50, 137.13, 128.69, 126.19, 124.70, 121.54, 114.58, 65.01 (d, *J* = 5.6 Hz), 33.65, 32.15, 30.70, 30.63, 29.15, 25.20, 23.63 (d, *J* = 9.9 Hz), 22.36, 16.04 (dd, *J* = 6.9, 5.2 Hz); ^{31}P NMR (162 MHz, Chloroform-*d*) δ -0.72. **HRMS** (TOF-MS ES$^+$) [M+Na]$^+$, calculated for $C_{22}H_{33}O_4NaP$; 415.2014; found 415.2012.

Characterization data for 2l to 2ae (for new compounds)

((1R*,2S*,3S*)-3-allyl-2-butyl-2-ethylcyclopropyl)methanol (2q)

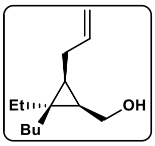

The title compound was prepared according to the general procedure **B**. The product was obtained as a colourless liquid (65%, *dr* 95:05). ^{1}H NMR (400 MHz, Chloroform-*d*) δ 5.93 (dddd, *J* = 17.1, 10.2, 6.9, 5.4 Hz, 1H), 5.11 (dq, *J* = 17.2, 1.8 Hz, 1H), 5.00 (dq, *J* = 10.2, 1.6 Hz, 1H), 3.73 (dd, *J* = 11.5, 6.7 Hz, 1H), 3.59 (dd, *J* = 11.5, 8.6 Hz, 1H), 2.25 (dddd, *J* = 13.6, 7.6, 3.7, 1.7 Hz, 1H), 2.10 – 2.01 (m, 1H), 1.36 – 1.21 (m, 9H), 0.88 (dt, *J* = 10.2, 7.0 Hz, 7H), 0.74 (td, *J* = 8.8, 6.3 Hz, 1H); ^{13}C NMR (101 MHz, Chloroform-*d*) δ 139.17, 114.41, 59.83, 31.66, 28.78, 28.33, 28.30, 27.53, 25.85, 25.10, 23.32, 14.11, 10.30; **HRMS** (APCI) [M-OH]$^+$, calculated for $C_{13}H_{23}$; 179.1800; found 179.1729.

((1R*,2S*,3S*)-3-allyl-2-butyl-2-propylcyclopropyl)methanol (2r)

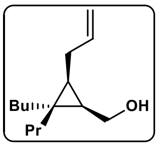

The title compound was prepared according to the general procedure **B**. The product was obtained as a colourless liquid (66%, *dr* 94:06). ^{1}H NMR (400 MHz, Chloroform-*d*) δ 5.93 (dddd, *J* = 17.2, 10.2, 6.9, 5.4 Hz, 1H), 5.12 (dq, *J* = 17.2, 1.8 Hz, 1H), 5.01 (dq, *J* = 10.2, 1.6 Hz, 1H), 3.79 – 3.68 (m, 1H), 3.59 (dd, *J* = 11.5, 8.7 Hz, 1H), 2.26 (dtt, *J* = 13.3, 5.7, 1.8 Hz, 1H), 2.04 (dddt, *J* = 15.8, 8.5, 6.8, 1.5 Hz, 1H), 1.33 – 1.19 (m, 10H), 0.88 (dtd, *J* = 10.1, 5.0, 4.2, 1.9 Hz, 8H), 0.74 (td, *J* = 8.9, 6.1 Hz, 1H); ^{13}C NMR (101 MHz, Chloroform-*d*) δ 139.21, 114.43, 59.85, 38.90, 28.43, 28.41, 28.30, 28.25, 26.46, 25.96, 22.94, 19.81, 14.72, 14.16. **HRMS** (APCI) [M-H]$^+$, calculated for $C_{14}H_{25}O$; 209.1900; found 209.1908.

((1R*,2S*,3S*)-3-allyl-2-butyl-2-propylcyclopropyl)methanol (2s)

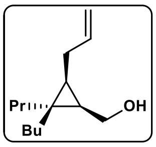

The title compound was prepared according to the general procedure **B**. The product was obtained as a colourless liquid (65%, *dr* 94:06). ^{1}H NMR (400 MHz, Chloroform-*d*) δ 5.93 (dddd, *J* = 17.2, 10.2, 6.9, 5.4 Hz, 1H), 5.12 (dq, *J* = 17.2, 1.8 Hz, 1H), 5.01 (dq, *J* = 10.2, 1.6 Hz, 1H), 3.74 (dd, *J* = 11.6, 6.6 Hz, 1H), 3.59 (dd, *J* = 11.5, 8.7 Hz, 1H), 2.32 – 2.22 (m, 1H), 2.09 – 1.99 (m, 1H), 1.36 – 1.25 (m, 8H), 1.22 – 1.16 (m, 2H), 0.93 – 0.84 (m, 8H), 0.75 (td, *J* = 8.8, 6.1 Hz, 1H); ^{13}C NMR (101 MHz, Chloroform-*d*) δ 139.22, 114.43, 41.43, 28.87, 28.35, 28.26, 26.40, 25.90, 25.61, 23.33, 19.32, 14.34, 14.12. **HRMS** (APCI) [M-H]$^+$, calculated for $C_{14}H_{25}O$; 209.1900; found 209.1908.

((1R*,2R*,3S*)-2-butyl-2-ethyl-3-propylcyclopropyl)methanol (2t)

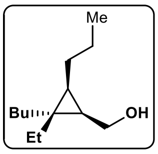

The title compound was prepared according to literature procedure[7] from cyclopropyl carbinol corresponding to **3p** using H_2, Pd/C. The product was obtained as a colourless liquid (90%, *dr* 95:05). ^{1}H NMR (400 MHz, Chloroform-*d*) δ 3.66 (td, *J* = 11.8, 11.3, 5.9 Hz, 2H), 1.39 – 1.24 (m, 11H), 1.15 – 1.05 (m, 2H), 0.90 (td, *J* = 7.1, 2.8 Hz, 9H), 0.77 (t, *J* = 8.0 Hz, 1H), 0.71 – 0.56 (m, 1H); ^{13}C NMR (101 MHz, Chloroform-*d*) δ 60.02, 38.33, 28.62, 28.42, 27.14 (d, *J* = 2.3 Hz), 26.28, 22.98, 18.30, 14.23, 14.16, 10.93; **HRMS** (APCI) [M+H]$^+$, calculated for $C_{13}H_{27}O$; 199.2062; found 199.2068.

((1R*,2S*,3S*)-3-allyl-2-ethyl-2-phenylcyclopropyl)methanol (2v)

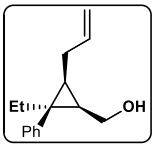

The title compound was prepared according to the general procedure **B**. The product was obtained as a colourless liquid (65%, *dr* 90:10). ^{1}H NMR (400 MHz, Chloroform-*d*) δ 7.25 – 7.18 (m, 2H), 7.18 – 7.12 (m, 1H), 7.09 (d, *J* = 7.6 Hz, 2H), 6.04 – 5.87 (m, 1H), 5.08 (dt, *J* = 17.3, 1.7 Hz, 1H), 4.99 (dt, *J* = 10.3, 1.7 Hz, 1H), 3.73 (dt, *J* = 12.7, 6.7 Hz, 1H), 3.21 (t, *J* = 10.4 Hz, 1H), 2.33 (dtd, *J* = 16.5, 5.1, 2.5 Hz, 1H), 1.70 – 1.57 (m, 1H), 1.40 (dtd, *J* = 32.8, 13.3, 12.8, 6.8 Hz, 3H), 1.23 (td, *J* = 8.9, 5.7 Hz, 1H), 1.13 (dd, *J* = 9.3, 5.2 Hz, 1H), 0.74 (t, *J* = 7.3 Hz, 3H); ^{13}C NMR (101 MHz, Chloroform-*d*) δ 139.34, 138.94, 131.08, 128.09, 126.25, 114.73, 61.22, 37.36, 35.51, 30.51, 28.63, 25.61, 10.86; **HRMS** (APCI) [M-OH]$^+$, calculated for $C_{15}H_{19}$; 199.1487; found 199.1492.

((1R*,2R*,3S*)-2-butyl-3-(cyclopropylmethyl)-2-ethylcyclopropyl)methanol (2w)

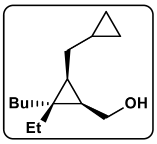

The title compound was prepared according to the general procedure **D**. The product was obtained as a colourless liquid (86%, *dr* 95:05). ^{1}H NMR (400 MHz, Chloroform-*d*) δ 3.75 – 3.61 (m, 2H), 1.30 (tdt, *J* = 20.9, 10.1, 5.3 Hz, 9H), 1.11 (t, *J* = 7.7 Hz, 2H), 0.90 (q, *J* = 7.5 Hz,

7H), 0.79 (dd, *J* = 13.8, 7.3 Hz, 2H), 0.43 (dd, *J* = 8.5, 4.5 Hz, 2H), 0.03 (t, *J* = 4.8 Hz, 2H); ^{13}C NMR (101 MHz, Chloroform-*d*) δ 60.05, 38.27, 29.13, 28.67, 28.38, 27.10, 27.03, 22.94, 18.46, 14.18, 11.40, 10.95, 4.52; **HRMS** (APCI) $[M+H]^+$, calculated for $C_{14}H_{25}O$; 209.1900; found 209.1898.

((1R*,2S*,3S*)-3-allyl-2-butyl-2-(pent-4-en-1-yl)cyclopropyl)methanol (2y)

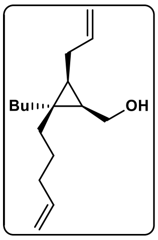

The title compound was prepared according to the general procedure **B**. The product was obtained as a colourless liquid (60%, *dr* 94:06). ^{1}H NMR (400 MHz, Chloroform-*d*) δ 5.93 (dddd, *J* = 17.1, 10.2, 6.9, 5.4 Hz, 1H), 5.84 – 5.75 (m, 1H), 5.12 (dq, *J* = 17.2, 1.8 Hz, 1H), 5.04 – 4.93 (m, 3H), 3.73 (dd, *J* = 11.6, 6.7 Hz, 1H), 3.59 (dd, *J* = 11.5, 8.6 Hz, 1H), 2.26 (dtt, *J* = 15.4, 5.7, 1.8 Hz, 1H), 2.11 – 1.97 (m, 3H), 1.42 – 1.20 (m, 10H), 0.92 – 0.83 (m, 5H), 0.75 (td, *J* = 8.8, 6.1 Hz, 1H); ^{13}C NMR (101 MHz, Chloroform-*d*) δ 139.13, 138.74, 114.60, 114.48, 59.78, 38.85, 34.29, 28.41, 28.27, 26.44, 25.96, 25.90, 25.42, 22.92, 14.16. **HRMS** (APCI) $[M-H]^+$, calculated for $C_{16}H_{27}O$; 235.2056; found 235.2042.

((1R*,2S*,3S*)-3-allyl-2-ethyl-2-phenethylcyclopropyl)methanol (2z)

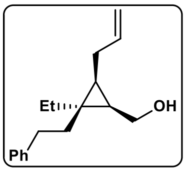

The title compound was prepared according to the general procedure **B**. The product was obtained as a colourless liquid (52%, *dr* 90:10). ^{1}H NMR (400 MHz, Chloroform-*d*) δ 7.22 (t, *J* = 7.4 Hz, 2H), 7.12 (t, *J* = 7.0 Hz, 3H), 5.86 (ddt, *J* = 16.6, 11.0, 6.1 Hz, 1H), 5.06 (dt, *J* = 17.2, 1.7 Hz, 1H), 4.95 (dt, *J* = 10.3, 1.6 Hz, 1H), 3.68 – 3.51 (m, 2H), 2.56 (ddt, *J* = 13.8, 9.7, 5.5 Hz, 2H), 2.23 – 2.15 (m, 1H), 2.02 (q, *J* = 7.9 Hz, 1H), 1.57 (td, *J* = 13.9, 12.6, 6.7 Hz, 3H), 1.30 (ddd, *J* = 24.3, 14.2, 6.9 Hz, 2H), 0.90 (t, *J* = 7.5 Hz, 3H), 0.84 – 0.71 (m, 2H); ^{13}C NMR (101 MHz, Chloroform-*d*) δ 142.80, 138.94, 128.22, 125.81, 114.62, 59.67, 33.08, 31.58, 28.38, 28.31, 28.23, 27.47, 25.83, 10.34; **HRMS** (APCI) $[M-H]^+$, calculated for $C_{17}H_{23}O$; 243.1749; found 243.1756.

((1R*,2R*,3S*)-3-allyl-2-(3-chloropropyl)-2-ethylcyclopropyl)methanol (2a)

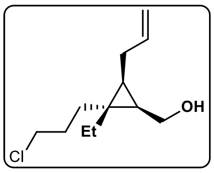

The title compound was prepared according to the general procedure **B**. The product was obtained as a colourless liquid (60%, *dr* 90:10). ^{1}H NMR (400 MHz, Chloroform-*d*) δ 5.99 – 5.86 (m, 1H), 5.11 (dt, *J* = 17.1, 1.8 Hz, 1H), 5.02 (dd, *J* = 10.2, 1.7 Hz, 1H), 3.76 – 3.70 (m, 1H), 3.62 (dd, *J* = 11.6, 8.4 Hz, 1H), 3.53 (t, *J* = 6.7 Hz, 2H), 2.29 – 2.22 (m, 1H), 2.10 – 2.01 (m, 1H), 1.86 – 1.78 (m, 2H), 1.43 – 1.36 (m, 4H), 0.96 – 0.90 (m, 4H), 0.82 (ddd, *J* = 17.4, 8.6, 2.2 Hz, 2H); ^{13}C NMR (101 MHz, Chloroform-*d*) δ 138.81, 114.69, 59.53, 45.15, 35.51, 29.42, 28.36, 28.09, 26.54, 25.95, 18.40, 10.87; **HRMS** (APCI) $[M-H]^+$, calculated for $C_{12}H_{20}ClO$; 215.1197; found 215.1175.

((1R*,2R*,3R*)-3-allyl-2-butyl-2-ethyl-1-phenylcyclopropyl)methanol (2ab)

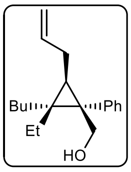

The title compound was prepared according to the general procedure **B**. The product was obtained as a colourless liquid (62%, *dr* 85:15, after column purification, *dr* = 95:05). ^{1}H NMR (400 MHz, Chloroform-*d*) δ 7.13 (d, *J* = 7.4 Hz, 2H), 7.09 – 7.04 (m, 3H), 5.86 (ddt, *J* = 16.9, 10.1, 6.7 Hz, 1H), 5.00 (dd, *J* = 17.1, 1.8 Hz, 1H), 4.89 (ddt, *J* = 10.1, 2.3, 1.3 Hz, 1H), 3.84 (dd, *J* = 11.7, 5.6 Hz, 1H), 3.62 (dd, *J* = 11.8, 7.0 Hz, 1H), 2.25 – 2.15 (m, 2H), 1.51 (dd, *J* = 14.6, 7.3 Hz, 1H), 1.31 – 1.23 (m, 2H), 1.19 – 1.08 (m, 2H), 0.94 (dt, *J* = 10.3, 2.8 Hz, 3H), 0.85 (t, *J* = 7.4 Hz, 3H), 0.72 – 0.65 (m, 1H), 0.60 (d, *J*

= 7.1 Hz, 3H), 0.06 – -0.05 (m, 1H); ^{13}C NMR (101 MHz, Chloroform-*d*) δ 142.24, 138.86, 130.42, 128.29, 126.34, 114.93, 64.58, 40.14, 34.64, 32.84, 32.27, 29.36, 28.52, 22.85, 18.76, 14.11, 11.32; **HRMS** (APCI) [M-H]$^+$, calculated for $C_{19}H_{27}O$; 271.2056; found 271.2046.

((1R*,2S*,3R*)-3-allyl-2-butyl-2-ethyl-1-phenylcyclopropyl)methanol (2ac)

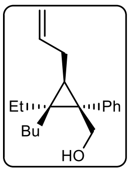

The title compound was prepared according to the general procedure **B**. The product was obtained as a colourless liquid (58%, *dr* 85:15, after column purification, *dr* = 90:10). ^{1}H NMR (400 MHz, Chloroform-*d*) δ 7.26 – 7.22 (m, 2H), 7.19 – 7.13 (m, 3H), 6.01 – 5.89 (m, 1H), 5.11 (dq, *J* = 17.1, 1.7 Hz, 1H), 4.99 (ddt, *J* = 10.1, 2.3, 1.3 Hz, 1H), 3.98 – 3.89 (m, 1H), 3.72 (d, *J* = 11.7 Hz, 1H), 2.40 – 2.23 (m, 2H), 1.63 – 1.53 (m, 1H), 1.44 – 1.25 (m, 5H), 1.20 – 1.16 (m, 1H), 0.99 (t, *J* = 7.5 Hz, 1H), 0.90 (d, *J* = 7.2 Hz, 3H), 0.81 – 0.77 (m, 1H), 0.71 (t, *J* = 7.4 Hz, 3H), 0.16 (ddd, *J* = 14.1, 7.6, 1.1 Hz, 1H); ^{13}C NMR (101 MHz, Chloroform-*d*) δ 142.36, 138.85, 130.42, 128.30, 126.37, 114.92, 64.74, 40.69, 33.03, 31.84, 29.40, 29.26, 28.31, 25.21, 23.38, 14.16, 10.52; **HRMS** (APCI) [M-H]$^+$, calculated for $C_{19}H_{27}O$; 271.2056; found 271.2046.

1-((1R*,2R*,3S*)-3-ethyl-2-phenyl-2-propylcyclopropyl)ethanol (2ae)

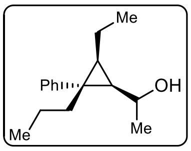

The title compound was prepared according to the general procedure **E**. The product was obtained as a colourless liquid (79%, *dr* 98:02). ^{1}H NMR (400 MHz, Chloroform-*d*) δ 7.23 – 7.12 (m, 4H), 7.11 – 7.05 (m, 1H), 3.76 (dq, *J* = 9.5, 6.1 Hz, 1H), 1.67 – 1.47 (m, 4H), 1.47 – 1.41 (m, 1H), 1.40 (d, *J* = 6.1 Hz, 3H), 1.10 (t, *J* = 7.4 Hz, 4H), 1.07 – 1.00 (m, 3H), 0.70 (t, *J* = 7.3 Hz, 3H); ^{13}C NMR (101 MHz, Chloroform-*d*) δ 147.73, 129.19, 127.98, 125.77, 65.47, 35.02, 33.30, 32.78, 28.17, 24.37, 20.43, 17.61, 14.94, 14.61; **HRMS** (APCI) [M-OH]$^+$, calculated for $C_{16}H_{23}$; 215.1794; found 215.1791.

Characterization data for 3l to 3ae

((1R*,2R*)-2-butyl-2-ethylcyclopropyl)methyl diethyl phosphate (3l)

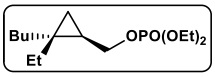

The title compound was prepared according to the general procedure **B**. The product was obtained as a colourless liquid (85%, *dr* 95:05). ^{1}H NMR (400 MHz, Chloroform-*d*) δ 4.14 – 4.05 (m, 5H), 3.96 (dt, *J* = 10.9, 8.1 Hz, 1H), 1.39 – 1.24 (m, 13H), 1.11 (dt, *J* = 14.4, 7.2 Hz, 1H), 0.96 – 0.83 (m, 7H), 0.52 (dd, *J* = 8.6, 4.7 Hz, 1H), 0.18 (t, *J* = 5.0 Hz, 1H); ^{13}C NMR (101 MHz, Chloroform-*d*) δ 68.81 (d, *J* = 5.8 Hz), 63.52 (dd, *J* = 5.9, 1.9 Hz), 29.68 (d, *J* = 2.4 Hz), 28.84, 25.83, 23.38 (d, *J* = 8.2 Hz), 23.04, 17.15, 16.10 (d, *J* = 6.7 Hz), 14.08, 10.38; ^{31}P NMR (162 MHz, Chloroform-*d*) δ -0.88; **HRMS** (APCI) [M+H]$^+$, calculated for $C_{14}H_{30}O_4P$; 293.1905; found 293.1876.

Diethyl (((1R*,2S*)-2-hexyl-2-phenylcyclopropyl)methyl) phosphate (3m)

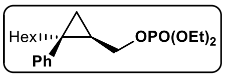

The title compound was prepared according to the general procedure **B**. The product was obtained as a colourless liquid (82%, *dr* 90:10). ^{1}H NMR (400 MHz, Chloroform-*d*) δ 7.13 (d, *J* = 4.2 Hz, 4H), 7.06 (tt, *J* = 5.9, 3.5 Hz, 1H), 4.07 – 3.80 (m, 4H), 3.66 – 3.41 (m, 2H), 1.80 – 1.68 (m, 1H), 1.28 – 1.20 (m, 1H), 1.15 (tt, *J* = 7.1, 1.0 Hz, 6H), 1.11 – 0.95 (m, 9H), 0.80 – 0.73 (m, 2H), 0.69 (t, *J* = 6.9

Hz, 3H); ^{13}C NMR (101 MHz, Chloroform-*d*) δ 140.69, 130.16, 128.12, 126.44, 69.49 (d, *J* = 5.6 Hz), 63.50 (d, *J* = 5.9 Hz), 41.68, 32.15, 31.76, 29.25, 26.69, 24.04 (d, *J* = 8.1 Hz), 22.58, 16.08 (d, *J* = 6.8 Hz), 15.53, 14.031; ^{31}P NMR (162 MHz, Chloroform-*d*) δ -1.01; **HRMS** (APCI) [M+H]$^+$, calculated for $C_{20}H_{34}O_4P$; 369.2195; found 369.2176.

Diethyl (((1R*,2R*)-2-methyl-2-(4-methylpent-3-en-1-yl)cyclopropyl)methyl) phosphate (3n)

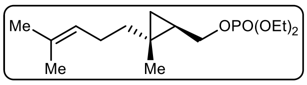

The title compound was prepared according to the general procedure **D**. The product was obtained as a colourless liquid (88%, *dr* 98:02). ^{1}H NMR (400 MHz, Chloroform-*d*) δ 5.12 – 5.02 (m, 1H), 4.18 (dd, *J* = 11.3, 6.3 Hz, 1H), 4.09 (q, *J* = 7.2 Hz, 4H), 3.88 (dt, *J* = 10.5, 8.4 Hz, 1H), 2.03 (q, *J* = 7.9 Hz, 2H), 1.66 (s, 3H), 1.58 (s, 3H), 1.32 (t, *J* = 7.1 Hz, 7H), 1.20 – 1.12 (m, 1H), 1.08 (s, 3H), 1.01 – 0.97 (m, 1H), 0.56 (dd, *J* = 8.7, 4.7 Hz, 1H), 0.19 (t, *J* = 5.0 Hz, 1H); ^{13}C NMR (101 MHz, Chloroform-*d*) δ 131.25, 124.33, 69.14 (d, *J* = 5.8 Hz), 63.53 (d, *J* = 5.8 Hz), 40.98, 25.63, 25.24, 23.28 (d, *J* = 8.2 Hz), 20.52, 18.00, 17.52, 17.12, 16.11 (d, *J* = 6.7 Hz); ^{31}P NMR (162 MHz, Chloroform-*d*) δ -0.87; **HRMS** (APCI) [M+H]$^+$, calculated for $C_{15}H_{30}O_4P$; 305.1876; found 305.1891.

((1R*,2R*,3S*)-3-allyl-2-butyl-2-methylcyclopropyl)methyl diethyl phosphate (3o)

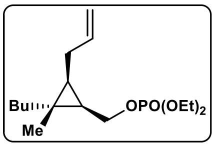

The title compound was prepared according to the general procedure **B**. The product was obtained as a colourless liquid (80%, *dr* 98:02). ^{1}H NMR (400 MHz, Chloroform-*d*) δ 5.90 – 5.76 (m, 1H), 5.04 (dt, *J* = 17.1, 1.7 Hz, 1H), 4.95 (dt, *J* = 10.2, 1.6 Hz, 1H), 4.18 – 3.99 (m, 6H), 2.15 – 1.99 (m, 2H), 1.32 (t, *J* = 7.1 Hz, 7H), 1.28 – 1.14 (m, 5H), 0.98 (d, *J* = 1.2 Hz, 3H), 0.97 – 0.91 (m, 1H), 0.90 – 0.83 (m, 3H), 0.74 (q, *J* = 8.0 Hz, 1H); ^{13}C NMR (101 MHz, Chloroform-*d*) δ 837.98, 814.49, 766.00 (d, *J* = 5.8 Hz), 763.52 (d, *J* = 5.9 Hz), 742.48, 728.59, 728.38, 725.96, 725.03 (d, *J* = 8.6 Hz), 722.73, 722.64, 716.05 (d, *J* = 6.8 Hz), 714.05, 712.00; ^{31}P NMR (162 MHz, Chloroform-*d*) δ -0.73; **HRMS** (APCI) [M+H]$^+$, calculated for $C_{16}H_{32}O_4P$; 319.2038; found 319.2027.

((1R*,2R*,3S*)-2,3-diethyl-2-phenylcyclopropyl)methyl diethyl phosphate (3p)

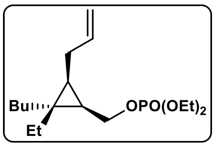

The title compound was prepared according to the general procedure **B**. The product was obtained as a colourless liquid (81%, *dr* 95:05). ^{1}H NMR (400 MHz, Chloroform-*d*) δ 5.83 (ddt, *J* = 16.4, 10.9, 5.9 Hz, 1H), 5.04 (dd, *J* = 17.0, 4.7 Hz, 1H), 4.99 – 4.90 (m, 1H), 4.09 (p, *J* = 7.2 Hz, 6H), 2.12 (q, *J* = 7.8 Hz, 2H), 1.44-1.13 (m, 14H), 0.98 – 0.84 (m, 7H), 0.77 (q, *J* = 8.0 Hz, 1H); ^{13}C NMR (101 MHz, Chloroform-*d*) δ 138.20, 114.55, 65.71 (d, *J* = 5.7 Hz), 63.56 (d, *J* = 5.9 Hz), 37.99, 28.20 (d, *J* = 7.5 Hz), 27.53, 26.50, 25.59 (d, *J* = 8.8 Hz), 22.87, 18.32, 16.11 (d, *J* = 6.7 Hz), 14.11, 10.77; ^{31}P NMR (162 MHz, Chloroform-*d*) δ -0.75; **HRMS** (TOF-MS ES$^+$) [M+Na]$^+$, calculated for $C_{17}H_{33}O_4NaP$; 355.2014; found 355.2017.

((1R*,2S*,3S*)-3-allyl-2-butyl-2-ethylcyclopropyl)methyl diethyl phosphate (3q)

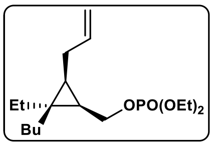

The title compound was prepared according to the general procedure **B**. The product was obtained as a colourless liquid (83%, *dr* 95:05). ^{1}H NMR (400 MHz, Chloroform-*d*) δ 5.83 (ddtd, *J* = 16.6, 10.2, 6.3, 1.6 Hz, 1H), 5.05 (dq, *J* = 17.1, 1.7 Hz, 1H), 4.95 (dt, *J* = 10.1, 1.6 Hz, 1H), 4.15 – 4.04 (m, 6H), 2.11 (dtd, *J* = 7.7, 6.3, 1.7 Hz, 2H), 1.37 – 1.23 (m, 14H),

0.95 (td, *J* = 7.9, 6.4 Hz, 1H), 0.87 (qd, *J* = 7.2, 3.5 Hz, 6H), 0.75 (td, *J* = 9.1, 8.3, 6.7 Hz, 1H); ^{13}C NMR (101 MHz, Chloroform-*d*) δ 138.18, 114.53, 65.75 (d, *J* = 5.6 Hz), 63.55 (d, *J* = 5.9 Hz), 31.48, 28.60, 28.34, 27.81, 26.39, 25.51 (d, *J* = 8.8 Hz), 24.93, 23.31, 16.11 (d, *J* = 6.7 Hz), 14.08, 10.17; ^{31}P NMR (162 MHz, Chloroform-*d*) δ -0.75; **HRMS** (TOF-MS ES$^+$) [M+Na]$^+$, calculated for $C_{17}H_{33}O_4NaP$; 355.2014; found 355.2017.

((1R*,2R*,3S*)-3-allyl-2-butyl-2-propylcyclopropyl)methyl diethyl phosphate (3r)

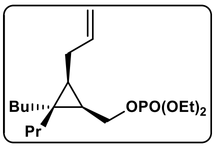

The title compound was prepared according to the general procedure **B.** The product was obtained as a colourless liquid (86%, *dr* 94:06). ^{1}H NMR (400 MHz, Chloroform-*d*) δ 5.77 (ddt, *J* = 16.6, 10.2, 6.2 Hz, 1H), 4.99 (dq, *J* = 17.1, 1.8 Hz, 1H), 4.89 (dq, *J* = 10.2, 1.6 Hz, 1H), 4.14 – 3.92 (m, 6H), 2.12 – 1.99 (m, 2H), 1.31 – 1.17 (m, 14H), 1.17 – 1.08 (m, 2H), 0.90 (q, *J* = 8.3 Hz, 1H), 0.82 (dt, *J* = 9.2, 6.9 Hz, 6H), 0.69 (dt, *J* = 9.0, 7.5 Hz, 1H); ^{13}C NMR (101 MHz, Chloroform-*d*) δ 138.20, 114.56, 65.78 (d, *J* = 5.7 Hz), 63.58 (d, *J* = 5.9 Hz), 38.70, 28.35, 28.25, 28.07, 26.77, 26.50, 25.64 (d, *J* = 8.9 Hz), 22.89, 19.66, 16.13 (d, *J* = 6.7 Hz), 14.70, 14.12; ^{31}P NMR (162 MHz, Chloroform-*d*) δ -0.75; **HRMS** (APCI) [M+H]$^+$, calculated for $C_{18}H_{36}O_4P$; 347.2346; found 347.2345.

((1R*,2S*,3S*)-3-allyl-2-butyl-2-propylcyclopropyl)methyl diethyl phosphate (3s)

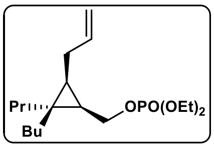

The title compound was prepared according to the general procedure **B**. The product was obtained as a colourless liquid (85%, *dr* 94:06). ^{1}H NMR (400 MHz, Chloroform-*d*) δ 5.81 (ddt, *J* = 16.7, 10.2, 6.3 Hz, 1H), 5.03 (dt, *J* = 17.2, 1.8 Hz, 1H), 4.93 (dq, *J* = 10.1, 1.5 Hz, 1H), 4.07 (p, *J* = 7.3 Hz, 6H), 2.14 – 2.02 (m, 2H), 1.34 – 1.23 (m, 14H), 1.16 (ddd, *J* = 11.6, 9.5, 6.0 Hz, 2H), 0.93 (q, *J* = 8.3 Hz, 1H), 0.90 – 0.81 (m, 6H), 0.73 (dt, *J* = 9.0, 7.5 Hz, 1H); ^{13}C NMR (101 MHz, Chloroform-*d*) δ 138.20, 114.56, 65.76 (d, *J* = 5.8 Hz), 63.57 (d, *J* = 5.9 Hz), 41.25, 28.68, 28.31, 26.70, 26.45, 25.56 (d, *J* = 8.9 Hz), 25.45, 23.34, 19.18, 16.12 (d, *J* = 6.7 Hz), 14.30, 14.09; ^{31}P NMR (162 MHz, Chloroform-*d*) δ -0.76; **HRMS** (APCI) [M+H]$^+$, calculated for $C_{18}H_{36}O_4P$; 347.2346; found 347.2330.

((1R*,2R*,3S*)-2-butyl-2-ethyl-3-propylcyclopropyl)methyl diethyl phosphate (3t)

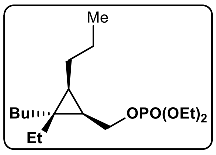

The title compound was prepared according to the general procedure **B**. The product was obtained as a colourless liquid (84%, *dr* 95:05). ^{1}H NMR (400 MHz, Chloroform-*d*) δ 4.17 – 4.01 (m, 6H), 1.39 – 1.20 (m, 17H), 1.14 – 1.05 (m, 1H), 0.93 – 0.82 (m, 10H), 0.66 (q, *J* = 7.1 Hz, 1H); ^{13}C NMR (101 MHz, Chloroform-*d*) δ 65.98 (d, *J* = 5.6 Hz), 63.50 (d, *J* = 5.9 Hz), 38.11, 28.21, 27.52, 27.46, 26.15, 25.72 (d, *J* = 8.8 Hz), 23.46, 22.88, 18.23, 16.10 (d, *J* = 6.8 Hz), 14.12 (d, *J* = 2.5 Hz), 10.77; ^{31}P NMR (162 MHz, Chloroform-*d*) δ -0.73; **HRMS** (APCI) [M+H]$^+$, calculated for $C_{17}H_{36}O_4P$; 335.2351; found 335.2378.

((1R*,2S*,3S*)-3-allyl-2-butyl-2-phenylcyclopropyl)methyl diethyl phosphate (3u)

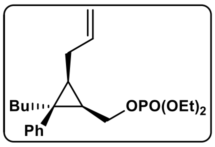

The title compound was prepared according to the general procedure **B**. The product was obtained as a colourless liquid (75%, *dr* 90:10). ^{1}H NMR (400 MHz, Chloroform-*d*) δ 7.24 – 7.19 (m, 2H), 7.15 (td, *J* = 6.3, 1.2 Hz, 3H), 5.87 (ddt, *J* = 16.6, 10.2, 6.0 Hz, 1H), 5.05 (dq, *J* = 17.2, 1.8 Hz, 1H), 4.96 (dq, *J* = 10.2, 1.6 Hz, 1H), 4.09 – 3.99 (m, 5H), 3.81 (dt, *J* = 10.9, 7.2 Hz, 1H), 2.08 (dtt, *J* = 16.3, 6.6, 1.6 Hz, 1H), 1.76 (dddt, *J* = 15.3, 7.5, 5.6, 1.8 Hz, 1H), 1.31 – 1.23

(m, 8H), 1.16 – 1.09 (m, 5H), 0.88 – 0.78 (m, 1H), 0.76 – 0.71 (m, 3H); ^{13}C NMR (101 MHz, Chloroform-*d*) δ 138.49, 138.04, 131.12, 128.24, 126.43, 114.83, 66.67 (d, *J* = 5.6 Hz), 63.63 (d, *J* = 5.8 Hz), 44.27, 34.94, 30.37, 28.79, 26.26 (d, *J* = 8.7 Hz), 26.11, 22.63, 16.13 (dd, *J* = 6.7, 2.0 Hz), 14.02; ^{31}P NMR (162 MHz, Chloroform-*d*) δ -0.84. **HRMS** (APCI) [M+H]$^+$, calculated for $C_{21}H_{34}O_4P$; 381.2189; found 381.2196.

((1R*,2S*,3S*)-3-allyl-2-ethyl-2-phenylcyclopropyl)methyl diethyl phosphate (3v)

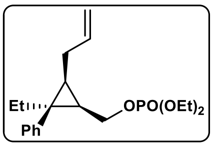

The title compound was prepared according to the general procedure **B**. The product was obtained as a colourless liquid (75%, *dr* 90:10). ^{1}H NMR (400 MHz, Chloroform-*d*) δ 7.25 – 7.19 (m, 2H), 7.15 (t, *J* = 4.4 Hz, 3H), 5.88 (ddt, *J* = 16.6, 11.0, 6.0 Hz, 1H), 5.06 (dt, *J* = 17.3, 1.8 Hz, 1H), 4.97 (dt, *J* = 10.3, 1.7 Hz, 1H), 4.04 (dp, *J* = 14.7, 7.0 Hz, 5H), 3.81 (dt, *J* = 11.1, 7.3 Hz, 1H), 2.08 (dt, *J* = 16.0, 6.6 Hz, 1H), 1.84 – 1.74 (m, 1H), 1.42 (ddt, *J* = 20.7, 13.3, 6.7 Hz, 2H), 1.27 (q, *J* = 7.3 Hz, 7H), 1.11 (q, *J* = 7.8 Hz, 1H), 0.74 (t, *J* = 7.3 Hz, 3H); ^{13}C NMR (101 MHz, Chloroform-*d*) δ 138.09, 138.06, 131.22, 128.24, 126.47, 114.82, 66.66 (d, *J* = 5.6 Hz), 63.63 (d, *J* = 5.8 Hz), 37.18, 36.05, 30.36, 26.08 (d, *J* = 8.6 Hz), 25.98, 16.14 (dd, *J* = 6.8, 2.2 Hz), 10.85; ^{31}P NMR (162 MHz, Chloroform-*d*) δ -0.81. **HRMS** (APCI) [M]$^+$, calculated for $C_{19}H_{29}NaO_4P$; 375.1701; found 375.1707.

((1R*,2R*,3S*)-2-butyl-3-(cyclopropylmethyl)-2-ethylcyclopropyl)methyl diethyl phosphate (3w)

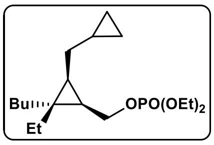

The title compound was prepared according to the general procedure **B**. The product was obtained as a colourless liquid (77%, *dr* 95:05). ^{1}H NMR (400 MHz, Chloroform-*d*) δ 4.08 (p, *J* = 7.4 Hz, 6H), 1.30 (qt, *J* = 13.7, 7.3 Hz, 15H), 1.13 – 0.99 (m, 1H), 0.86 (ddt, *J* = 24.1, 16.2, 8.9 Hz, 8H), 0.66 (ddd, *J* = 13.0, 8.1, 5.5 Hz, 1H), 0.39 (d, *J* = 7.8 Hz, 2H), 0.00 (d, *J* = 5.0 Hz, 2H); ^{13}C NMR (101 MHz, Chloroform-*d*) δ 65.95 (d, *J* = 5.7 Hz), 63.47 (d, *J* = 5.9 Hz), 38.01, 29.02, 28.13, 27.50, 27.31, 25.74 (d, *J* = 8.7 Hz), 22.80, 18.33, 16.06 (d, *J* = 6.8 Hz), 14.08, 11.18, 10.75, 4.37 (d, *J* = 10.3 Hz); ^{31}P NMR (162 MHz, Chloroform-*d*) δ -0.74; **HRMS** (APCI) [M+H]$^+$, calculated for $C_{18}H_{36}O_4P$; 347.2346; found 347.2340.

((1R*,2R*,3S*)-2-butyl-2-ethyl-3-(oxiran-2-ylmethyl)cyclopropyl)methyl diethyl phosphate (3x)

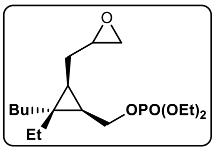

The title compound was prepared according to the general procedure **C**. The product was obtained as a colourless liquid (72%, *dr* 95:05). ^{1}H NMR (400 MHz, Chloroform-*d*) (*mixture of diastereomers*) δ 4.13 – 3.98 (m, *J* = 8.7, 7.9 Hz, 6H), 2.90 (d, *J* = 6.2 Hz, 1H), 2.70 (q, *J* = 4.2 Hz, 1H), 2.45 (q, *J* = 4.3 Hz, 1H), 1.98 (s, 1H), 1.57 (h, *J* = 8.4 Hz, 2H), 1.30 – 1.20 (m, 12H), 1.09 (t, *J* = 10.4 Hz, 1H), 0.94 (t, *J* = 8.1 Hz, 1H), 0.87 – 0.77 (m, 7H); ^{13}C NMR (101 MHz, Chloroform-*d*) (*mixture of diastereomers*) δ 65.52 (d, *J* = 5.7 Hz), 63.60 (d, *J* = 5.9 Hz), 52.15 (d, *J* = 9.5 Hz), 47.05 (d, *J* = 5.9 Hz), 37.87, 28.09, 27.50 (d, *J* = 10.9 Hz), 27.17 (d, *J* = 13.0 Hz), 25.65 (dd, *J* = 11.6, 8.7 Hz), 23.52 (d, *J* = 31.3 Hz), 22.81, 18.46, 16.09 (d, *J* = 6.8 Hz), 14.07, 10.69 (d, *J* = 6.0 Hz); ^{31}P NMR (162 MHz, Chloroform-*d*) (*mixture of diastereomers*) δ -0.76, -0.76; **HRMS** (TOF-MS ES$^+$) [M+Na]$^+$, calculated for $C_{17}H_{33}O_5NaP$; 371.1963; found 371.1963.

((1R*,2S*,3S*)-3-allyl-2-butyl-2-(pent-4-en-1-yl)cyclopropyl)methyl diethyl phosphate (3y)

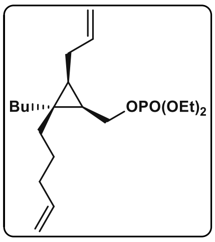

The title compound was prepared according to the general procedure **B**. The product was obtained as a colourless liquid (83%, *dr* 94:06). ^{1}H NMR (400 MHz, Chloroform-*d*) δ 5.90 – 5.71 (m, 2H), 5.08 – 4.90 (m, 4H), 4.08 (td, *J* = 8.5, 8.0, 6.3 Hz, 6H), 2.18 – 1.97 (m, 4H), 1.40 (td, *J* = 7.1, 1.1 Hz, 3H), 1.35 – 1.30 (m, 8H), 1.28 – 1.24 (m, 3H), 1.20 (dd, *J* = 8.8, 5.5 Hz, 2H), 0.96 (q, *J* = 8.2 Hz, 1H), 0.91 – 0.83 (m, 3H), 0.76 (dt, *J* = 9.0, 7.5 Hz, 1H); ^{13}C NMR (101 MHz, Chloroform-*d*) δ 138.67, 138.10, 114.60, 114.55, 65.69 (d, *J* = 5.7 Hz), 63.59 (d, *J* = 5.9 Hz), 38.64, 34.27, 28.30, 28.22, 26.72, 26.49, 25.70, 25.62 (d, *J* = 8.9 Hz), 25.25, 22.85, 16.11 (d, *J* = 6.6 Hz), 14.09; ^{31}P NMR (162 MHz, Chloroform-*d*) δ -0.79; **HRMS** (APCI) [M+H]$^+$, calculated for $C_{20}H_{38}O_4P$; 373.2508; found 373.2489.

((1R*,2S*,3S*)-3-allyl-2-ethyl-2-phenethylcyclopropyl)methyl diethyl phosphate (3z)

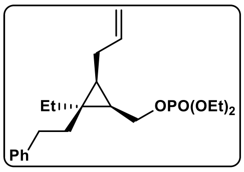

The title compound was prepared according to the general procedure **B**. The product was obtained as a colourless liquid (78%, *dr* = 90:10). ^{1}H NMR (400 MHz, Chloroform-*d*) δ 7.25 – 7.15 (m, 2H), 7.16 – 7.08 (m, 4H), 5.78 (ddt, *J* = 16.7, 10.6, 6.2 Hz, 1H), 5.00 (dt, *J* = 17.3, 1.8 Hz, 1H), 4.91 (d, *J* = 10.2 Hz, 1H), 4.04 (ddtd, *J* = 18.0, 14.2, 11.0, 7.2 Hz, 7H), 2.69 – 2.45 (m, 2H), 2.07 (t, *J* = 6.9 Hz, 2H), 1.59 (td, *J* = 7.6, 3.9 Hz, 2H), 1.34 (dt, *J* = 14.4, 7.2 Hz, 1H), 1.25 (td, *J* = 7.1, 4.6 Hz, 6H), 0.97 (q, *J* = 8.3 Hz, 1H), 0.90 (t, *J* = 7.4 Hz, 3H), 0.77 (q, *J* = 7.9 Hz, 2H); ^{13}C NMR (101 MHz, Chloroform-*d*) δ 142.70, 137.98, 128.38, 128.20, 125.77, 114.76, 65.55 (d, *J* = 5.7 Hz), 63.59 (d, *J* = 5.9 Hz), 32.85, 31.40, 28.23, 28.07, 27.74, 26.42, 25.65 (d, *J* = 8.7 Hz), 16.11 (d, *J* = 6.6 Hz), 10.23; ^{31}P NMR (162 MHz, Chloroform-*d*) δ -0.73; **HRMS** (APCI) [M+H]$^+$, calculated for $C_{21}H_{34}O_4P$; 381.2189; found 381.2178.

((1R*,2R*,3S*)-3-allyl-2-(3-chloropropyl)-2-ethylcyclopropyl)methyl diethyl phosphate (3aa)

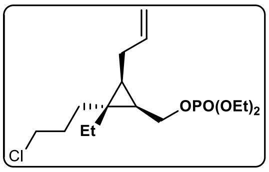

The title compound was prepared according to the general procedure **B**. The product was obtained as a colourless liquid (72%, *dr* 90:10). ^{1}H NMR (400 MHz, Chloroform-*d*) δ 5.83 (ddt, *J* = 16.6, 10.1, 6.3 Hz, 1H), 5.09 – 5.03 (m, 1H), 4.98 (dd, *J* = 10.2, 1.7 Hz, 1H), 4.17 – 4.06 (m, 6H), 3.53 (t, *J* = 6.6 Hz, 2H), 2.18 – 2.07 (m, 2H), 1.87 – 1.78 (m, 2H), 1.64 (d, *J* = 1.7 Hz, 1H), 1.43 – 1.38 (m, 3H), 1.34 (td, *J* = 7.1, 1.1 Hz, 6H), 1.07 – 1.01 (m, 1H), 0.93 (t, *J* = 7.4 Hz, 3H), 0.86 – 0.80 (m, 1H); ^{13}C NMR (101 MHz, Chloroform-*d*) δ 137.86, 114.88, 65.45 (d, *J* = 5.5 Hz), 63.66 (d, *J* = 5.9 Hz), 45.06, 35.24, 29.29, 28.15, 26.78, 26.50, 25.63 (d, *J* = 9.2 Hz), 18.18, 16.15 (d, *J* = 6.7 Hz), 10.71; ^{31}P NMR (162 MHz, Chloroform-*d*) δ -0.76; **HRMS** (APCI) [M+H]$^+$, calculated for $C_{16}H_{31}ClO_4P$; 353.1643; found 353.1639.

((1R*,2R*,3R*)-3-allyl-2-butyl-2-ethyl-1-phenylcyclopropyl)methyl diethyl phosphate (3ab)

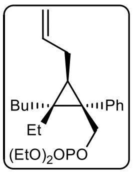

The title compound was prepared according to the general procedure B with DMAP (3 equiv.) as a base. The product was obtained as a colourless liquid (52%, *dr* 95:05). ^{1}H NMR (400 MHz, Chloroform-*d*) δ 7.21 – 7.16 (m, 4H), 7.11 (td, *J* = 6.5, 2.1 Hz, 1H), 5.95 – 5.82 (m, 1H), 5.08 (dd, *J* = 17.1, 1.7 Hz, 1H), 4.97 (dt, *J* = 10.1, 1.6 Hz, 1H), 4.38 (dd, *J* = 10.6, 4.0 Hz, 1H), 4.13 (dd, *J* = 10.6, 2.8 Hz, 1H), 3.82 (dt, *J* = 10.1, 7.3 Hz, 1H), 3.76 – 3.68 (m, 1H), 3.60 (td, *J* = 7.3, 2.3 Hz, 2H), 2.36 (dt, *J* = 15.2, 6.9 Hz, 1H), 2.28 – 2.16 (m, 1H), 1.64 (dt, *J* = 14.7, 7.3 Hz, 1H), 1.44 – 1.32 (m, 2H), 1.24 (tt, *J* = 8.0,

5.3 Hz, 1H), 1.15 – 1.09 (m, 4H), 1.03 (td, *J* = 7.0, 1.0 Hz, 6H), 0.95 (t, *J* = 7.3 Hz, 3H), 0.69 (t, *J* = 7.1 Hz, 3H), 0.12 (ddd, *J* = 14.2, 11.4, 4.3 Hz, 1H); ^{13}C NMR (101 MHz, Chloroform-*d*) δ 142.03, 138.10, 130.50, 127.89, 126.17, 115.07, 70.47 (d, *J* = 5.7 Hz), 63.29 (dd, *J* = 7.5, 5.8 Hz), 37.58 (d, *J* = 10.5 Hz), 34.46, 33.44, 32.74, 29.34, 28.45, 22.80, 18.78, 16.06 – 15.67 (m), 14.05, 11.32; ^{31}P NMR (162 MHz, Chloroform-*d*) δ -1.49; **HRMS** (APCI) [M+H]$^+$, calculated for $C_{23}H_{38}O_4P$; 409.2502; found 409.2508.

((1R*,2S*,3R*)-3-allyl-2-butyl-2-ethyl-1-phenylcyclopropyl)methyl diethyl phosphate (3ac)

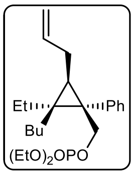

The title compound was prepared according to the general procedure B with DMAP (3 equiv.) as a base. The product was obtained as a colourless liquid (56%, *dr* 90:10). ^{1}H NMR (400 MHz, Chloroform-*d*) δ 7.21 – 7.16 (m, 4H), 7.13 – 7.08 (m, 1H), 5.88 (ddt, *J* = 16.9, 10.1, 6.7 Hz, 1H), 5.08 (dt, *J* = 17.1, 1.7 Hz, 1H), 5.02 – 4.92 (m, 1H), 4.38 (dd, *J* = 10.5, 4.1 Hz, 1H), 4.13 (dd, *J* = 10.5, 2.8 Hz, 1H), 3.82 (ddd, *J* = 10.3, 7.8, 7.0 Hz, 1H), 3.77 – 3.68 (m, 1H), 3.59 (dtt, *J* = 10.6, 7.1, 3.9 Hz, 2H), 2.45 – 2.32 (m, 1H), 2.27 – 2.14 (m, 1H), 1.68 – 1.58 (m, 2H), 1.46 – 1.40 (m, 1H), 1.33 – 1.24 (m, 2H), 1.12 (td, *J* = 7.0, 1.0 Hz, 3H), 1.07 – 1.00 (m, 4H), 0.89 (t, *J* = 7.2 Hz, 3H), 0.82 – 0.76 (m, 2H), 0.71 (t, *J* = 7.4 Hz, 3H), 0.25 – 0.13 (m, 1H); ^{13}C NMR (101 MHz, Chloroform-*d*) δ 142.18, 138.12, 130.52, 127.91, 126.22, 115.06, 70.54 (d, *J* = 5.7 Hz), 63.31 (dd, *J* = 10.2, 6.0 Hz), 38.14 (d, *J* = 10.5 Hz), 33.60, 32.32, 29.29 (d, *J* = 12.4 Hz), 28.19, 25.34, 23.37, 16.00 (d, *J* = 6.8 Hz), 15.92 (d, *J* = 7.0 Hz), 14.15, 10.50; ^{31}P NMR (162 MHz, Chloroform-*d*) δ - 1.50; **HRMS** (APCI) [M+H]$^+$, calculated for $C_{23}H_{38}O_4P$; 409.2502; found 409.2508.

1-((1R*,2R*,3S*)-3-allyl-2-butyl-2-ethylcyclopropyl)ethyl diethyl phosphate (3ad)

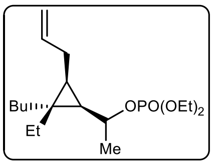

The title compound was prepared according to the general procedure **E**. The product was obtained as a colourless liquid (50%, *dr* 95:05). ^{1}H NMR (400 MHz, Chloroform-*d*) (*mixture of diastereomers*) δ 6.01 – 5.68 (m, 1H), 5.15 – 4.84 (m, 2H), 4.28 (dtd, *J* = 10.0, 6.4, 3.6 Hz, 1H), 4.04 (ddt, *J* = 9.2, 6.9, 3.2 Hz, 4H), 2.15 – 1.92 (m, 2H), 1.63 – 1.52 (m, 1H), 1.41 (t, *J* = 6.7 Hz, 3H), 1.28 (t, *J* = 7.2 Hz, 8H), 1.24 – 1.19 (m, 3H), 0.93 – 0.76 (m, 9H), 0.68 (td, *J* = 7.0, 3.2 Hz, 1H); ^{13}C NMR (101 MHz, Chloroform-*d*) (*mixture of diastereomers*) δ 138.39 & 137.78, 114.30 & 113.87, 74.74 (d, *J* = 5.8 Hz) & 74.62 (d, *J* = 5.9 Hz), 62.97 (d, *J* = 2.7 Hz) & 62.85 (d, *J* = 6.4 Hz), 37.88 & 37.64, 32.60 & 32.50, 27.96 & 27.92, 27.81 & 27.69, 26.67 & 26.46, 25.71 (d, *J* = 1.4 Hz), 22.94 & 22.83, 22.57 & 22.52, 18.20 & 18.12, 15.68 (d, *J* = 7.1 Hz), 13.71 & 13.65, 10.25 & 10.19; ^{31}P NMR (162 MHz, Chloroform-*d*) (*mixture of diastereomers*) δ -2.06, -2.18; **HRMS** (APCI) [M+H]$^+$, calculated for $C_{18}H_{36}O_4P$; 347.2346; found 347.2353.

Diethyl (1-((1R*,2R*,3S*)-3-ethyl-2-phenyl-2-propylcyclopropyl)ethyl) phosphate (3ae)

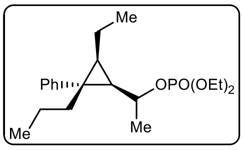

The title compound was prepared according to the general procedure **E**. The product was obtained as a colourless liquid (54%, *dr* 98:02). ^{1}H NMR (400 MHz, Chloroform-*d*) δ 7.22 – 7.13 (m, 4H), 7.12 – 7.06 (m, 1H), 4.45 (dp, *J* = 10.1, 6.2 Hz, 1H), 4.03 (pd, *J* = 7.2, 3.6 Hz, 4H), 1.69 (ddd, *J* = 14.1, 7.4, 5.6 Hz, 1H), 1.59 (d, *J* = 6.1 Hz, 3H), 1.57 – 1.48 (m, 2H), 1.47 – 1.34 (m, 2H), 1.29 – 1.24 (m, 5H), 1.19 (d, *J* = 9.8 Hz, 1H), 1.14 – 1.08 (m, 4H), 1.08 – 0.94 (m, 2H), 0.70 (t, *J* = 7.3 Hz, 3H); ^{13}C NMR (101 MHz, Chloroform-*d*) δ 147.30, 129.11, 128.01, 125.89, 74.70 (d, *J* = 5.8 Hz), 63.39 (d, *J* = 5.9 Hz), 33.45, 33.34 (d, *J* = 1.8 Hz), 32.70, 28.76, 23.39, 20.42, 17.35, 16.07 (d, *J* = 7.1 Hz), 14.85, 14.53; ^{31}P NMR (162 MHz, Chloroform-*d*) δ -1.95; **HRMS** (APCI) [M+H]$^+$, calculated for $C_{20}H_{34}O_4P$; 369.2195; found 369.2198.

Characterization data for 5a to 5ae

((3S*,4S*)-4-ethyl-3-methylhex-5-en-3-yl)benzenene (5a)

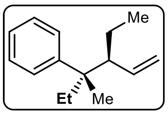

The title compound was prepared according to the general procedure **F**. The product was obtained as a colourless liquid (90%, *dr* 94:06). ^{1}H NMR (400 MHz, Chloroform-*d*) δ 7.37 – 7.28 (m, 4H), 7.22 (tt, *J* = 7.0, 1.5 Hz, 1H), 5.45 (dt, *J* = 17.1, 9.9 Hz, 1H), 5.01 (dd, *J* = 10.2, 2.3 Hz, 1H), 4.86 (dd, *J* = 17.1, 2.3 Hz, 1H), 2.09 – 1.96 (m, 2H), 1.71 – 1.56 (m, 2H), 1.33 (s, 3H), 0.97 (ddd, *J* = 13.2, 11.3, 6.9 Hz, 1H), 0.81 (t, *J* = 7.3 Hz, 3H), 0.78 – 0.72 (m, 3H); ^{13}C NMR (101 MHz, Chloroform-*d*) δ 146.17, 139.33, 127.53, 127.41, 125.17, 116.39, 58.07, 43.85, 31.09, 21.39, 21.31, 12.87, 8.71. **HRMS** (APCI) $[M-H]^+$, calculated for $C_{15}H_{21}$; 201.1638; found 201.1636.

((3S*,4S*)-3-methyl-4-vinylnonan-3-yl)benzene (5b)

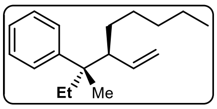

The title compound was prepared according to the general procedure **F**. The product was obtained as a colourless liquid (88%, *dr* 95:05). ^{1}H NMR (400 MHz, Chloroform-*d*) δ 7.39 – 7.28 (m, 4H), 7.26 – 7.18 (m, 1H), 5.47 (dt, *J* = 17.4, 9.8 Hz, 1H), 4.99 (dd, *J* = 10.3, 2.3 Hz, 1H), 4.84 (dd, *J* = 17.1, 2.2 Hz, 1H), 2.17 – 1.97 (m, 2H), 1.66 (dq, *J* = 14.5, 7.4 Hz, 1H), 1.56 – 1.47 (m, 1H), 1.33 (s, 3H), 1.31 – 1.25 (m, 3H), 1.22 – 0.97 (m, 3H), 0.91 (t, *J* = 6.7 Hz, 3H), 0.75 (t, *J* = 7.3 Hz, 3H); ^{13}C NMR (101 MHz, Chloroform-*d*) δ 146.18, 139.77, 127.52, 127.41, 125.16, 116.07, 55.85, 43.81, 31.85, 31.18, 28.52, 27.80, 22.63, 21.14, 14.09, 8.72. **HRMS** (APCI) $[M-H]^+$, calculated for $C_{18}H_{27}$; 243.2107; found 243.2083.

((3S*,4S*)-4-methyl-3-vinylhexane-1,4-diyl)dibenzene (5c)

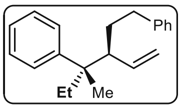

The title compound was prepared according to the general procedure **F**. The product was obtained as a colourless liquid (85%, *dr* 94:06). ^{1}H NMR (400 MHz, Chloroform-*d*) δ 7.22 – 7.11 (m, 6H), 7.07 (t, *J* = 6.9 Hz, 2H), 6.99 (d, *J* = 7.7 Hz, 2H), 5.40 (dt, *J* = 17.4, 9.8 Hz, 1H), 4.94 (dd, *J* = 10.2, 2.2 Hz, 1H), 4.77 (dd, *J* = 17.0, 2.2 Hz, 1H), 2.54 (ddd, *J* = 14.4, 10.2, 4.7 Hz, 1H), 2.23 (ddd, *J* = 13.9, 9.9, 7.0 Hz, 1H), 2.01 (ddd, *J* = 11.6, 9.5, 2.2 Hz, 1H), 1.86 (dq, *J* = 14.6, 7.4 Hz, 1H), 1.81 – 1.70 (m, 1H), 1.51 (dt, *J* = 13.9, 7.1 Hz, 1H), 1.26 – 1.17 (m, 1H), 1.17 (s, 3H), 0.59 (t, *J* = 7.3 Hz, 3H); ^{13}C NMR (101 MHz, Chloroform-*d*) δ 145.85, 142.70, 139.31, 128.41, 128.18, 127.60, 127.38, 125.52, 125.25, 116.86, 55.38, 43.78, 34.26, 31.06, 30.52, 21.19, 8.68. **HRMS** (APCI) $[M-H]^+$, calculated for $C_{21}H_{25}$; 277.1951; found 277.1949.

((3S*,4S*)-3,6-dimethyl-4-vinylheptan-3-yl)benzene (5d)

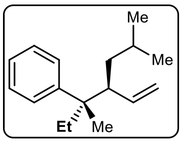

The title compound was prepared according to the general procedure **F**. The product was obtained as a colourless liquid (88%, *dr* 95:05). ^{1}H NMR (400 MHz, Chloroform-*d*) δ 7.25 – 7.13 (m, 4H), 7.11 – 7.04 (m, 1H), 5.32 (dt, *J* = 17.1, 9.9 Hz, 1H), 4.83 (dd, *J* = 10.3, 2.2 Hz, 1H), 4.70 (dd, *J* = 17.1, 2.3 Hz, 1H), 2.07 (ddd, *J* = 11.6, 9.4, 2.5 Hz, 1H), 1.87 (dt, *J* = 14.6, 7.3 Hz, 1H), 1.57 – 1.42 (m, 1H), 1.34 (dtt, *J* = 13.2, 6.6, 3.2 Hz, 1H), 1.18 (s, 3H), 1.05 (ddd, *J* = 13.1, 10.5, 2.4 Hz, 1H), 0.91 (ddd, *J* = 13.6, 11.2, 3.1 Hz, 1H), 0.74 (d, *J* = 6.7 Hz, 3H), 0.67 (d, *J* = 6.5 Hz, 3H), 0.60 (t, *J* = 7.4 Hz, 3H); ^{13}C NMR (101 MHz, Chloroform-*d*) δ 146.12, 139.84, 127.51, 127.44, 125.16, 115.88, 53.43, 43.68,

38.00, 31.20, 25.29, 24.36, 21.09, 20.85, 8.72. **HRMS** (APCI) [M-H]$^+$, calculated for $C_{18}H_{25}$; 229.1951; found 229.1935.

((3S*,4S*)-3-methyl-4-vinylnon-8-en-3-yl)benzene (5e)

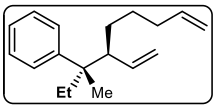

The title compound was prepared according to the general procedure **F**. The product was obtained as a colourless liquid (88%, *dr* 94:06). ^{1}H NMR (400 MHz, Chloroform-*d*) δ 7.25 – 7.14 (m, 4H), 7.08 (ddt, *J* = 8.5, 6.4, 1.6 Hz, 1H), 5.67 (ddt, *J* = 17.0, 10.2, 6.7 Hz, 1H), 5.39 – 5.27 (m, 1H), 4.91 – 4.78 (m, 3H), 4.70 (ddd, *J* = 17.1, 2.2, 0.7 Hz, 1H), 2.02 – 1.77 (m, 4H), 1.57 – 1.47 (m, 1H), 1.46 – 1.36 (m, 1H), 1.29 (ddddd, *J* = 13.1, 10.3, 8.7, 6.4, 4.3 Hz, 1H), 1.18 (s, 3H), 1.11 – 0.99 (m, 1H), 0.88 (dddd, *J* = 12.8, 11.2, 9.9, 4.3 Hz, 1H), 0.60 (t, *J* = 7.4 Hz, 3H); ^{13}C NMR (101 MHz, Chloroform-*d*) δ 146.08, 139.56, 139.05, 127.55, 127.40, 125.19, 116.26, 114.14, 55.73, 43.82, 33.68, 31.12, 28.03, 27.40, 21.14, 8.71. **HRMS** (APCI) [M+H]$^+$, calculated for $C_{18}H_{27}$; 243.2107; found 243.2101.

2-((4S*,5S*)-5-methyl-5-phenyl-4-vinylheptyl)oxirane (5f)

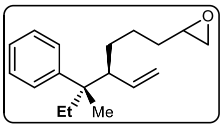

The title compound was prepared according to the general procedure **F**. The product was obtained as a colourless liquid (66%, *dr* 83:17). ^{1}H NMR (400 MHz, Chloroform-*d*) *(mixture of diastereomers)* δ 7.22 – 7.15 (m, 4H), 7.13 – 7.05 (m, 1H), 5.34 (dtd, *J* = 17.1, 10.0, 2.3 Hz, 1H), 4.87 (ddd, *J* = 10.3, 3.2, 2.2 Hz, 1H), 4.73 (ddd, *J* = 17.0, 5.7, 2.2 Hz, 1H), 2.74 (ddd, *J* = 4.9, 3.8, 2.5 Hz, 1H), 2.62 (d, *J* = 4.5 Hz, 1H), 2.32 (ddd, *J* = 7.7, 5.1, 2.8 Hz, 1H), 2.02 – 1.83 (m, 2H), 1.53 – 1.38 (m, 3H), 1.35 – 1.22 (m, 2H), 1.19 (s, 3H), 1.12 – 1.02 (m, 1H), 1.01 – 0.89 (m, 1H), 0.61 (td, *J* = 7.4, 1.7 Hz, 3H); ^{13}C NMR (101 MHz, Chloroform-*d*) *(mixture of diastereomers)* δ 145.90, 139.31 (d, *J* = 2.0 Hz), 127.58 (d, *J* = 1.3 Hz), 127.36, 125.25, 116.52 (d, *J* = 3.4 Hz), 55.93 & 55.86, 52.31 & 52.25, 47.15 & 47.00, 43.76 & 43.74, 32.36 & 32.33, 30.98 & 30.86, 28.32 & 28.29, 24.43, 21.39 &21.28, 8.68. **HRMS** (APCI) [M+H]$^+$, calculated for $C_{18}H_{27}O$; 259.2056; found 259.2058.

((3S*,4S*)-3-ethyl-4-methylhept-1-en-4-yl)benzene (5g)

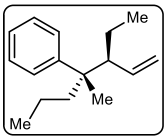

The title compound was prepared according to the general procedure **F**. The product was obtained as a colourless liquid (85%, *dr* 94:06). ^{1}H NMR (400 MHz, Chloroform-*d*) δ 7.27 – 7.14 (m, 4H), 7.08 (ddt, *J* = 7.6, 6.2, 1.8 Hz, 1H), 5.33 (dt, *J* = 17.2, 9.9 Hz, 1H), 4.88 (dd, *J* = 10.3, 2.3 Hz, 1H), 4.73 (dd, *J* = 17.1, 2.3 Hz, 1H), 1.91 – 1.73 (m, 2H), 1.53 – 1.41 (m, 2H), 1.21 (s, 3H), 1.16 – 1.07 (m, 1H), 0.96 – 0.83 (m, 2H), 0.79 (t, *J* = 7.0 Hz, 3H), 0.68 (t, *J* = 7.3 Hz, 3H); ^{13}C NMR (101 MHz, Chloroform-*d*) δ 146.73, 139.25, 127.53, 127.22, 125.15, 116.43, 58.23, 43.66, 41.25, 21.95, 21.35, 17.47, 14.93, 12.87. **HRMS** (APCI) [M-H]$^+$, calculated for $C_{16}H_{23}$; 215.1794; found 215.1782.

1-((3R*,4S*)-4-ethyl-3-methylhex-5-en-3-yl)naphthalene (5h)

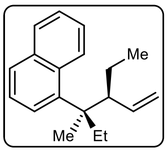

The title compound was prepared according to the general procedure **F**. The product was obtained as a colourless liquid (52%, *dr* 96:04). ^{1}H NMR (400 MHz, Chloroform-*d*) δ 8.03 – 7.71 (m, 3H), 7.68 (d, *J* = 1.9 Hz, 1H), 7.53 – 7.39 (m, 3H), 5.64 (dt, *J* = 17.0, 9.9 Hz, 1H), 5.21 – 5.07 (m, 2H), 2.19 (td, *J* = 9.5, 4.1 Hz, 1H), 1.85 (dq, *J* = 14.8, 7.5 Hz, 1H), 1.69 (dq, *J* = 14.5, 7.4 Hz, 1H), 1.34 (s, 3H), 1.11 – 0.98 (m, 2H), 0.65 (t, *J* = 7.4 Hz, 3H), 0.54 (t, *J* = 7.4 Hz, 3H); ^{13}C NMR

(101 MHz, Chloroform-*d*) δ 144.62, 139.52, 133.22, 131.59, 127.89, 127.41, 127.28, 125.95, 125.66, 125.23, 125.19, 117.13, 57.61, 44.36, 34.17, 21.96, 17.73, 12.66, 8.60. **HRMS** (APCI) $[M+H]^+$, calculated for $C_{19}H_{25}$; 253.1956; found 253.1966.

((4S*,5R*)-5-methyl-4-vinylnon-1-en-5-yl)benzene (5i)

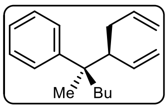

The title compound was prepared according to the general procedure **F**. The product was obtained as a colourless liquid (85%, *dr* 86:14). ^{1}H NMR (400 MHz, Chloroform-*d*) δ 7.39 – 7.28 (m, 4H), 7.26 – 7.20 (m, 1H), 5.69 (ddt, *J* = 17.8, 9.4, 6.9 Hz, 1H), 5.48 (ddd, *J* = 17.1, 10.3, 9.3 Hz, 1H), 5.02 (dd, *J* = 10.3, 2.2 Hz, 1H), 5.00 – 4.90 (m, 2H), 4.85 (ddd, *J* = 17.1, 2.2, 0.7 Hz, 1H), 2.34 (dddt, *J* = 14.0, 6.7, 2.8, 1.4 Hz, 1H), 2.22 (ddd, *J* = 11.7, 9.2, 2.7 Hz, 1H), 2.03 – 1.91 (m, 1H), 1.81 (dddt, *J* = 13.9, 11.3, 7.1, 1.3 Hz, 1H), 1.67 – 1.58 (m, 1H), 1.36 (s, 3H), 1.35 – 1.27 (m, 2H), 1.25 – 1.14 (m, 1H), 1.06 – 0.95 (m, 1H), 0.90 (t, *J* = 7.3 Hz, 3H); ^{13}C NMR (101 MHz, Chloroform-*d*) δ 146.17, 138.79, 138.38, 127.65, 127.22, 125.34, 116.60, 114.88, 55.86, 43.51, 38.52, 33.59, 26.44, 23.55, 21.85, 14.09. **HRMS** (APCI) $[M+H]^+$, calculated for $C_{18}H_{27}$; 243.2113; found 243.2127.

((3R*,4S*)-3-methyl-4-vinylhept-6-en-3-yl)benzene (5j)

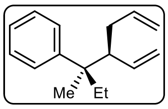

The title compound was prepared according to the general procedure **F**. The product was obtained as a colourless liquid (84%, *dr* 87:13). ^{1}H NMR (400 MHz, Chloroform-*d*) δ 7.42 – 7.29 (m, 4H), 7.27 – 7.21 (m, 1H), 5.78 – 5.64 (m, 1H), 5.55 – 5.43 (m, 1H), 5.08 – 4.91 (m, 3H), 4.86 (dd, *J* = 17.2, 2.0 Hz, 1H), 2.41 – 2.31 (m, 1H), 2.30 – 2.20 (m, 1H), 2.06 (dq, *J* = 14.6, 7.4 Hz, 1H), 1.88 – 1.75 (m, 1H), 1.68 (dq, *J* = 14.5, 7.3 Hz, 1H), 1.36 (s, 3H), 0.76 (t, *J* = 7.3 Hz, 3H); ^{13}C NMR (101 MHz, Chloroform-*d*) δ 145.64, 138.84, 138.36, 127.65, 127.36, 125.36, 116.56, 114.88, 55.67, 43.82, 33.62, 31.13, 21.15, 8.70. **HRMS** (APCI) $[M+H]^+$, calculated for $C_{16}H_{23}$; 215.1794; found 215.1824.

(S*)-1-methyl-1-((S*)-pent-1-en-3-yl)-1,2,3,4-tetrahydronaphthalene (5k)

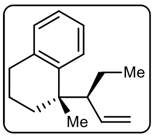

The title compound was prepared according to the general procedure **F**. The product was obtained as a colourless liquid (84%, *dr* 91:09). ^{1}H NMR (400 MHz, Chloroform-*d*) δ 7.21 – 7.14 (m, 1H), 7.06 (ddd, *J* = 8.1, 6.5, 2.1 Hz, 1H), 7.00 – 6.94 (m, 2H), 5.58 (dt, *J* = 17.1, 9.9 Hz, 1H), 5.13 – 4.95 (m, 2H), 2.66 – 2.58 (m, 2H), 2.18 (td, *J* = 10.1, 2.5 Hz, 1H), 1.78 – 1.68 (m, 2H), 1.62 (dddd, *J* = 15.2, 12.9, 6.2, 3.1 Hz, 1H), 1.51 – 1.45 (m, 1H), 1.15 (s, 3H), 1.08 – 0.86 (m, 2H), 0.62 (dd, *J* = 7.9, 6.9 Hz, 3H); ^{13}C NMR (101 MHz, Chloroform-*d*) δ 144.51, 139.33, 137.81, 128.95, 126.36, 125.90, 124.93, 117.34, 57.53, 40.29, 31.41, 30.97, 30.60, 22.29, 19.65, 12.81. **HRMS** (APCI) $[M+H]^+$, calculated for $C_{16}H_{23}$; 215.1794; found 215.1790.

(S*)-1-methyl-1-((S*)-5-methylhex-1-en-3-yl)-1,2,3,4-tetrahydronaphthalene (5l)

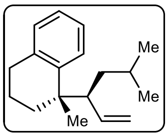

The title compound was prepared according to the general procedure **F**. The product was obtained as a colourless liquid (92%, *dr* 97:03). ^{1}H NMR (400 MHz, Chloroform-*d*) δ 7.18 – 7.14 (m, 1H), 7.05 (ddd, *J* = 8.0, 6.5, 2.2 Hz, 1H), 7.00 – 6.93 (m, 2H), 5.58 (dt, *J* = 17.0, 9.9 Hz, 1H), 5.12 – 4.88 (m, 2H), 2.62 (dd, *J* = 8.0, 4.1 Hz, 2H), 2.42 (ddd, *J* = 11.5, 9.4, 2.1 Hz, 1H), 1.73 (ddd, *J* = 11.2, 8.8, 5.7 Hz, 2H), 1.69 – 1.56 (m, 1H), 1.44 (td, *J* = 7.0, 6.3, 3.5 Hz, 1H), 1.41 – 1.30 (m,

1H), 1.15 (s, 3H), 1.09 (ddd, J = 13.4, 11.4, 3.1 Hz, 1H), 0.66 (d, J = 6.7 Hz, 3H), 0.62 (d, J = 6.5 Hz, 3H), 0.54 (ddd, J = 13.1, 10.7, 2.1 Hz, 1H); ^{13}C NMR (101 MHz, Chloroform-*d*) δ 144.29, 139.89, 137.80, 128.90, 126.75, 125.76, 124.92, 116.96, 52.57, 40.08, 38.50, 31.32, 30.95, 30.65, 25.45, 24.27, 20.82, 19.63. **HRMS** (APCI) $[M+H]^+$, calculated for $C_{18}H_{27}$; 243.2107; found 243.2126.

(S*)-1-methyl-1-((R*)-octa-1,7-dien-3-yl)-1,2,3,4-tetrahydronaphthalene (5m)

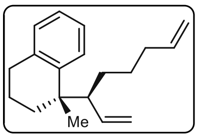

The title compound was prepared according to the general procedure **F**. The product was obtained as a colourless liquid (90%, *dr* 97:03). ^{1}H NMR (400 MHz, Chloroform-*d*) δ 7.16 (d, J = 7.9 Hz, 1H), 7.06 (td, J = 8.0, 7.2, 2.2 Hz, 1H), 7.02 – 6.92 (m, 2H), 5.68 – 5.52 (m, 2H), 5.11 – 4.95 (m, 2H), 4.90 – 4.70 (m, 2H), 2.62 (dd, J = 8.8, 4.2 Hz, 2H), 2.30 (td, J = 10.0, 2.3 Hz, 1H), 1.91 – 1.79 (m, 1H), 1.78 – 1.67 (m, 3H), 1.67 – 1.56 (m, 1H), 1.45 (q, J = 5.6 Hz, 1H), 1.29 – 1.19 (m, 1H), 1.14 (s, 3H), 1.09 – 0.99 (m, 2H), 0.92 – 0.84 (m, 1H); ^{13}C NMR (101 MHz, Chloroform-*d*) δ 144.32, 139.57, 139.07, 137.76, 128.96, 126.41, 125.90, 124.96, 117.28, 113.95, 55.04, 40.21, 33.50, 31.38, 30.92, 30.57, 28.89, 27.41, 19.62. **HRMS** (APCI) $[M+H]^+$, calculated for $C_{19}H_{27}$; 255.2107; found 255.2123.

(4S*,5R*)-5-ethyl-5-methyl-4-vinylnon-1-ene (5n)

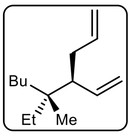

The title compound was prepared according to the general procedure **F**. The product was obtained as a colourless liquid (85%, *dr* 88:12). ^{1}H NMR (400 MHz, Chloroform-*d*) δ 5.71 (ddt, J = 16.7, 10.1, 6.6 Hz, 1H), 5.64 – 5.52 (m, 1H), 5.04 (dd, J = 10.2, 2.3 Hz, 1H), 4.98 – 4.88 (m, 3H), 2.29 – 2.20 (m, 1H), 1.97 – 1.84 (m, 2H), 1.26 (ddd, J = 14.9, 9.0, 5.1 Hz, 6H), 1.21 – 1.14 (m, 2H), 0.91 (t, J = 7.0 Hz, 3H), 0.80 – 0.73 (m, 6H); ^{13}C NMR (101 MHz, Chloroform-*d*) δ 139.32, 138.83, 116.03, 114.62, 51.30, 37.21, 35.86, 32.91, 29.50, 25.44, 23.69, 21.33, 14.20, 7.78. **HRMS** (APCI) $[M-H]^+$, calculated for $C_{14}H_{25}$; 193.1956; found 193.1957.

(4S*,5S*)-5-ethyl-5-methyl-4-vinylnon-1-ene (5o)

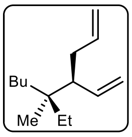

The title compound was prepared according to the general procedure **F**. The product was obtained as a colourless liquid (84%, *dr* 87:13). ^{1}H NMR (400 MHz, Chloroform-*d*) δ 5.71 (ddt, J = 16.8, 10.1, 6.8 Hz, 1H), 5.64 – 5.52 (m, 1H), 5.04 (dd, J = 10.3, 2.3 Hz, 1H), 4.98 – 4.87 (m, 3H), 2.24 (ddq, J = 13.4, 6.9, 1.7 Hz, 1H), 1.98 – 1.83 (m, 2H), 1.34 – 1.13 (m, 8H), 0.89 (t, J = 6.9 Hz, 3H), 0.82 – 0.72 (m, 6H); ^{13}C NMR (101 MHz, Chloroform-*d*) δ 139.31, 138.85, 116.02, 114.61, 51.28, 37.20, 36.52, 32.87, 28.85, 25.45, 23.66, 21.39, 14.21, 7.78. **HRMS** (APCI) $[M-H]^+$, calculated for $C_{14}H_{25}$; 193.1956; found 193.1957.

(4S*,5R*)-5-methyl-5-propyl-4-vinylnon-1-ene (5p)

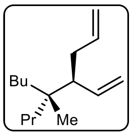

The title compound was prepared according to the general procedure **F**. The product was obtained as a colourless liquid (82%, *dr* 86:14). ^{1}H NMR (400 MHz, Chloroform-*d*) δ 5.71 (ddt, J = 16.8, 10.1, 6.7 Hz, 1H), 5.59 (ddd, J = 17.1, 10.2, 9.2 Hz, 1H), 5.04 (dd, J = 10.3, 2.3 Hz, 1H), 4.99 – 4.87 (m, 3H), 2.28 – 2.21 (m, 1H), 1.97 – 1.83 (m, 2H), 1.32 – 1.13 (m, 10H), 0.93 – 0.85 (m, 6H), 0.78 (s, 3H); ^{13}C NMR (101 MHz, Chloroform-*d*) δ 139.33, 138.83, 116.03, 114.62, 51.66, 40.00, 37.29, 36.56,

32.92, 25.51, 23.70, 21.90, 16.48, 15.03, 14.20. **HRMS** (APCI) [M-H]$^+$, calculated for $C_{15}H_{27}$; 207.2107; found 207.2099.

(4R*,5R*)-5-methyl-5-propyl-4-vinylnon-1-ene (5q)

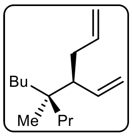

The title compound was prepared according to the general procedure **F**. The product was obtained as a colourless liquid (83%, *dr* 86:14). ^{1}H NMR (400 MHz, Chloroform-*d*) δ 5.72 (ddd, *J* = 17.0, 10.2, 6.9 Hz, 1H), 5.59 (ddd, *J* = 17.1, 10.2, 9.2 Hz, 1H), 5.04 (dd, *J* = 10.3, 2.3 Hz, 1H), 4.99 – 4.87 (m, 3H), 2.29 – 2.19 (m, 1H), 1.97 – 1.84 (m, 2H), 1.31 – 1.12 (m, 10H), 0.89 (td, *J* = 7.1, 2.8 Hz, 6H), 0.79 (s, 3H); ^{13}C NMR (101 MHz, Chloroform-*d*) δ 139.31, 138.83, 116.04, 114.61, 51.68, 39.39, 37.29, 37.20, 32.92, 25.53, 23.67, 21.92, 16.48, 15.07, 14.20; **HRMS** (APCI) [M-H]$^+$, calculated for $C_{15}H_{27}$; 207.2107; found 207.2099.

(4S*,5R*)-5-ethyl-5-methyl-4-vinylnonane (5r)

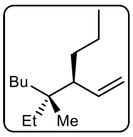

The title compound was prepared according to the general procedure **F**. The product was obtained as a colourless liquid (86%, *dr* 88:12). ^{1}H NMR (400 MHz, Chloroform-*d*) δ 5.56 (dt, *J* = 17.1, 10.0 Hz, 1H), 5.00 (dd, *J* = 10.3, 2.5 Hz, 1H), 4.90 (dd, *J* = 17.0, 2.4 Hz, 1H), 1.82 (t, *J* = 10.0 Hz, 1H), 1.36 (dd, *J* = 8.9, 4.7 Hz, 2H), 1.29 – 1.06 (m, 10H), 0.92 – 0.86 (m, 6H), 0.79 – 0.71 (m, 6H); ^{13}C NMR (101 MHz, Chloroform-*d*) δ 140.29, 115.47, 50.97, 37.03, 35.90, 30.08, 29.60, 25.48, 23.74, 21.41, 21.22, 14.22, 14.17, 7.80; **HRMS** (APCI) [M+H]$^+$, calculated for $C_{14}H_{29}$; 197.2269; found 197.2293.

(4S,*5S*)-5-butyl-5-methyl-4-vinyldeca-1,9-diene (5s)

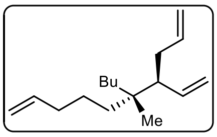

The title compound was prepared according to the general procedure **F**. The product was obtained as a colourless liquid (74%, *dr* 88:12). ^{1}H NMR (400 MHz, Chloroform-*d*) δ 5.90 – 5.78 (m, 1H), 5.76 – 5.66 (m, 1H), 5.60 (ddd, *J* = 17.1, 10.2, 9.2 Hz, 1H), 5.08 – 4.89 (m, 6H), 2.26 (ddt, *J* = 12.3, 7.0, 1.6 Hz, 1H), 2.04 – 1.86 (m, 4H), 1.37 – 1.15 (m, 10H), 0.92 (t, *J* = 7.0 Hz, 3H), 0.81 (s, 3H); ^{13}C NMR (101 MHz, Chloroform-*d*) δ 139.22, 139.18, 138.77, 116.14, 114.67, 114.22, 51.62, 37.20, 36.95, 36.51, 34.66, 32.90, 25.49, 23.66, 22.74, 21.88, 14.19. **HRMS** (APCI) [M-H]$^+$, calculated for $C_{15}H_{27}$; 233.2264; found 233.2243.

((3S*,4S*)-3-ethyl-3-methyl-4-vinylhept-6-en-1-yl)benzene (5t)

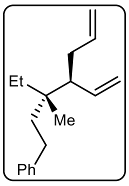

The title compound was prepared according to the general procedure **F**. The product was obtained as a colourless liquid (56%, *dr* 85:15). ^{1}H NMR (400 MHz, Chloroform-*d*) δ 7.34 – 7.30 (m, 2H), 7.22 (ddd, *J* = 7.7, 5.6, 2.0 Hz, 3H), 5.78 (ddt, *J* = 17.0, 10.1, 6.9 Hz, 1H), 5.72 – 5.62 (m, 1H), 5.13 (dd, *J* = 10.2, 2.2 Hz, 1H), 5.06 – 4.99 (m, 3H), 2.63 – 2.46 (m, 2H), 2.35 (dddd, *J* = 13.7, 5.8, 2.4, 1.3 Hz, 1H), 2.07 (ddd, *J* = 11.7, 9.6, 2.4 Hz, 1H), 2.01 – 1.93 (m, 1H), 1.60 – 1.53 (m, 2H), 1.48 (h, *J* = 6.6 Hz, 2H), 0.94 – 0.89 (m, 6H); ^{13}C NMR (101 MHz, Chloroform-*d*) δ 143.57, 138.95, 138.60, 128.30, 125.53, 116.41, 114.84, 51.30, 39.41, 37.50, 32.92, 29.87, 28.81, 21.23, 7.82. **HRMS** (APCI) [M+H]$^+$, calculated for $C_{18}H_{27}$; 243.2107; found 243.2100.

2-((2S*,3R*)-3-ethyl-3-methyl-2-vinylheptyl)oxirane (5u)

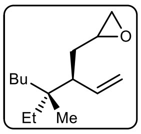

The title compound was prepared according to the general procedure **F**. The product was obtained as a colourless liquid (77%, *dr* 85:15). ^{1}H NMR (400 MHz, Chloroform-*d*) (*mixture of diastereomers*) δ 5.68 (ddt, *J* = 31.4, 16.5, 10.2 Hz, 1H), 5.12 – 4.95 (m, 2H), 2.96 – 2.83 (m, 1H), 2.73 (dt, *J* = 15.0, 4.5 Hz, 1H), 2.43 (ddd, *J* = 17.5, 5.1, 2.8 Hz, 1H), 2.25 – 2.03 (m, 1H), 1.69 – 1.52 (m, 2H), 1.45 – 1.30 (m, 2H), 1.27 – 1.23 (m, 3H), 1.17 (dd, *J* = 8.9, 5.0 Hz, 3H), 0.89 (t, *J* = 7.2 Hz, 4H), 0.75 (d, *J* = 6.4 Hz, 5H); ^{13}C NMR (101 MHz, Chloroform-*d*) (*mixture of diastereomers*) δ 139.34 & 138.99, 116.62 & 116.24, 52.18 & 51.80, 49.15 & 48.47, 46.96, 37.06 & 36.94, 35.78 & 35.73, 31.65 & 31.52, 29.34 & 29.24, 25.39 & 25.32, 23.63, 21.23, 14.16, 7.71; **HRMS** (APCI) $[M-OH]^+$, calculated for $C_{14}H_{25}$; 193.1951; found 193.1956.

((2S*,3R*)-3-ethyl-3-methyl-2-vinylheptyl)cyclopropane (5v)

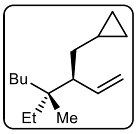

The title compound was prepared according to the general procedure **F**. The product was obtained as a colourless liquid (86%, *dr* 88:12). ^{1}H NMR (400 MHz, Chloroform-*d*) δ 5.65 (dt, *J* = 17.0, 10.0 Hz, 1H), 5.06 – 4.96 (m, 2H), 2.01 (ddd, *J* = 11.9, 9.8, 2.5 Hz, 1H), 1.45 – 1.38 (m, 1H), 1.28 – 1.22 (m, 5H), 1.16 (ddt, *J* = 4.9, 3.1, 2.0 Hz, 3H), 0.91 – 0.87 (m, 4H), 0.75 (d, *J* = 7.5 Hz, 2H), 0.72 (d, *J* = 3.1 Hz, 3H), 0.68 – 0.62 (m, 1H), 0.42 – 0.34 (m, 2H), 0.08 – 0.02 (m, 2H), -0.05 – -0.16 (m, 1H); ^{13}C NMR (101 MHz, Chloroform-*d*) δ 140.32, 115.50, 51.79, 36.83, 35.86, 33.45, 29.51, 25.42, 23.71, 21.38, 14.23, 9.86, 7.77, 5.41, 4.11. **HRMS** (APCI) $[M+H]^+$, calculated for $C_{15}H_{29}$; 209.3908; found 209.3947.

(4S*,5R*)-8-chloro-5-ethyl-5-methyl-4-vinyloct-1-ene (5w)

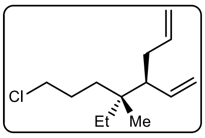

The title compound was prepared according to the general procedure **F**. The product was obtained as a colourless liquid (50%, *dr* 85:15). ^{1}H NMR (400 MHz, Chloroform-*d*) δ 5.76 – 5.64 (m, 1H), 5.57 (ddd, *J* = 17.0, 8.6, 1.2 Hz, 1H), 5.06 (dt, *J* = 10.2, 2.0 Hz, 1H), 5.00 – 4.87 (m, 3H), 3.49 (dtd, *J* = 10.6, 6.7, 1.6 Hz, 2H), 2.23 (dddt, *J* = 10.2, 4.7, 3.3, 1.6 Hz, 1H), 1.97 – 1.84 (m, 2H), 1.69 (dqt, *J* = 11.1, 4.5, 2.1 Hz, 2H), 1.41 – 1.24 (m, 4H), 0.85 – 0.73 (m, 6H); ^{13}C NMR (101 MHz, Chloroform-*d*) δ 138.78, 138.39, 116.46, 114.90, 51.26, 46.00, 37.13, 33.48, 32.86, 29.40, 26.87, 21.31, 7.71; **HRMS** (APCI) $[M+H]^+$, calculated for $C_{13}H_{24}Cl$; 215.1549; found 215.1561.

(R*)-4-ethyl-4-methyloct-1-ene (5x)

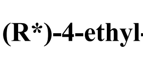

The title compound was prepared according to the general procedure **F**. The product was obtained as a colourless liquid (73%). ^{1}H NMR (400 MHz, Chloroform-*d*) δ 5.80 (ddt, *J* = 15.2, 9.5, 7.4 Hz, 1H), 5.08 – 4.95 (m, 2H), 1.96 (d, *J* = 7.4 Hz, 2H), 1.30 – 1.18 (m, 8H), 0.93 – 0.90 (m, 3H), 0.85 – 0.76 (m, 6H); ^{13}C NMR (101 MHz, Chloroform-*d*) δ 135.78, 116.36, 43.65, 38.50, 35.36, 31.43, 25.74, 24.33, 23.67, 14.19, 7.92; **HRMS** (APCI) $[M+H]^+$, calculated for $C_{11}H_{23}$; 155.1800; found 155.1813.

(R*)-4-methyl-4-phenyldec-1-en-5-one (5y)

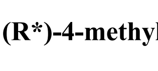

The title compound was prepared according to the general procedure **F**. The product was obtained as a colourless liquid (75%). ^{1}H NMR (400 MHz, Chloroform-*d*) δ 7.32 (d, *J* = 4.3 Hz, 4H), 7.19 (q, *J* = 4.5 Hz, 1H), 5.64 – 5.42 (m, 1H), 5.05 – 4.91 (m, 2H), 2.49 (dd, *J* = 13.8, 6.5 Hz, 1H), 2.31

(dd, J = 13.8, 8.0 Hz, 1H), 1.74 (td, J = 12.7, 4.3 Hz, 1H), 1.55 (td, J = 13.0, 12.4, 3.8 Hz, 1H), 1.29 (s, 3H), 1.27 – 1.09 (m, 7H), 0.99 (p, J = 7.2, 6.2 Hz, 1H), 0.86 (t, J = 6.7 Hz, 3H); ^{13}C NMR (101 MHz, Chloroform-*d*) δ 147.70, 135.40, 127.92, 126.35, 125.33, 116.81, 47.68, 42.66, 40.69, 31.75, 30.02, 24.11, 24.07, 22.65, 14.06; **HRMS** (APCI) $[M+H]^+$, calculated for $C_{17}H_{27}$; 231.2113; found 231.2124.

(S*)-4-ethyl-4,8-dimethylnona-1,7-diene (5z)

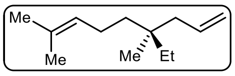

The title compound was prepared according to the general procedure **F**. The product was obtained as a colourless liquid (75%). ^{1}H NMR (400 MHz, Chloroform-*d*) δ 5.86 – 5.74 (m, 1H), 5.14 – 5.05 (m, 1H), 5.05 – 4.97 (m, 2H), 1.96 (d, J = 7.5 Hz, 2H), 1.89 (d, J = 10.0 Hz, 2H), 1.68 (s, 3H), 1.60 (s, 3H), 1.28 – 1.16 (m, 4H), 0.87 – 0.75 (m, 6H); ^{13}C NMR (101 MHz, Chloroform-*d*) δ 135.66, 130.86, 125.21, 116.50, 43.50, 38.75, 35.44, 31.33, 25.71, 24.21, 22.21, 17.54, 7.92. **HRMS** (APCI) $[M-H]^+$, calculated for $C_{13}H_{23}$; 179.1800; found 179.1783.

(4S*,5S*)-5-ethyl-5-methyl-4-vinylnon-1-ene (5aa)

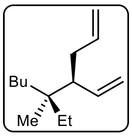

The title compound was prepared according to the general procedure **F**. The product was obtained as a colourless liquid (75%, *dr* 87:13). ^{1}H NMR (400 MHz, Chloroform-*d*) δ 5.71 (ddt, J = 16.8, 10.1, 6.8 Hz, 1H), 5.64 – 5.53 (m, 1H), 5.04 (dd, J = 10.2, 2.3 Hz, 1H), 4.99 – 4.87 (m, 3H), 2.24 (ddq, J = 13.4, 6.9, 1.7 Hz, 1H), 1.98 – 1.79 (m, 2H), 1.35 – 1.13 (m, 8H), 0.89 (t, J = 6.9 Hz, 3H), 0.84 – 0.70 (m, 6H); ^{13}C NMR (101 MHz, Chloroform-*d*) δ 139.31, 138.85, 116.02, 114.61, 51.28, 37.20, 36.52, 32.87, 28.84, 25.44, 23.66, 21.39, 14.20, 7.78; **HRMS** (APCI) $[M-H]^+$, calculated for $C_{14}H_{25}$; 193.1956; found 193.1957.

((3R*,4R*)-3-allyl-4-ethyl-4-methyloct-1-en-2-yl)benzene (5ab)

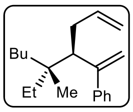

The title compound was prepared according to the general procedure **F**. The product was obtained as a colourless liquid (58%, 90% brsm, *dr* = 95:05). ^{1}H NMR (400 MHz, Chloroform-*d*) δ 7.27 – 7.19 (m, 4H), 7.17 – 7.11 (m, 1H), 5.92 – 5.78 (m, 1H), 5.33 (t, J = 1.0 Hz, 1H), 5.03 – 4.91 (m, 3H), 2.62 (dd, J = 12.1, 3.4 Hz, 1H), 2.34 – 2.19 (m, 2H), 1.23 – 1.15 (m, 4H), 1.05 (ddd, J = 11.1, 8.6, 5.4 Hz, 4H), 0.76 (t, J = 7.0 Hz, 3H), 0.68 (s, 3H), 0.51 (t, J = 7.5 Hz, 3H); ^{13}C NMR (101 MHz, Chloroform-*d*) δ 149.86, 146.74, 138.69, 128.03, 126.58, 126.42, 115.98, 115.57, 50.13, 39.40, 35.81, 34.27, 29.60, 25.48, 23.55, 21.19, 14.17, 7.89; **HRMS** (APCI) $[M-H]^+$, calculated for $C_{20}H_{29}$; 269.2264; found 269.2274.

((3R*,4S*)-3-allyl-4-ethyl-4-methyloct-1-en-2-yl)benzene (5ac)

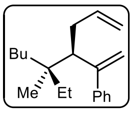

The title compound was prepared according to the general procedure **F**. The product was obtained as a colourless liquid (63%, 88% brsm, *dr* = 90:10). ^{1}H NMR (400 MHz, Chloroform-*d*) δ 7.26 – 7.18 (m, 4H), 7.15 – 7.11 (m, 1H), 5.92 – 5.78 (m, 1H), 5.33 (t, J = 1.0 Hz, 1H), 5.03 – 4.92 (m, 3H), 2.66 – 2.57 (m, 1H), 2.33 – 2.21 (m, 2H), 1.56 – 1.49 (m, 1H), 1.47 – 1.36 (m, 1H), 1.34 – 1.20 (m, 3H), 1.06 – 0.99 (m, 1H), 0.88 – 0.75 (m, 3H), 0.67 – 0.59 (m, 8H); ^{13}C NMR (101 MHz, Chloroform-*d*) δ 149.97, 146.72, 138.72, 128.00, 126.57, 126.46, 115.94, 115.55, 50.22, 39.34, 36.75, 34.18, 28.88, 25.59, 23.37, 21.14, 14.00, 7.87; **HRMS** (APCI) $[M-H]^+$, calculated for $C_{20}H_{29}$; 269.2264; found 269.2274.

(4S*,5R*)-5-ethyl-5-methyl-4-((*E*)-prop-1-en-1-yl)non-1-ene (5ad)

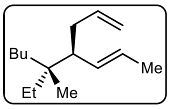

The title compound was prepared according to the general procedure **F**. The product was obtained as a colourless liquid (40%, *dr* 85:15). ^{1}H NMR (400 MHz, Chloroform-*d*) δ 5.71 (ddt, *J* = 16.9, 10.1, 6.5 Hz, 1H), 5.31 (dq, *J* = 15.2, 6.1 Hz, 1H), 5.23 – 5.13 (m, 1H), 4.98 – 4.87 (m, 2H), 2.23 – 2.15 (m, 1H), 1.87 – 1.83 (m, 1H), 1.66 (dd, *J* = 6.1, 1.4 Hz, 3H), 1.24 (ddt, *J* = 10.2, 7.2, 3.8 Hz, 5H), 1.15 (ddd, *J* = 14.8, 6.8, 4.0 Hz, 2H), 1.02 – 0.96 (m, 1H), 0.90 (t, *J* = 7.1 Hz, 5H), 0.77 – 0.71 (m, 5H); ^{13}C NMR (101 MHz, Chloroform-*d*) δ 139.27, 131.88, 126.26, 114.28, 49.89, 37.41, 35.89, 33.31, 29.49, 25.45, 23.71, 21.34, 17.98, 14.21, 7.81; **HRMS** (APCI) [M-H]$^+$, calculated for $C_{15}H_{27}$; 207.2107; found 207.2106.

((4S*,5S*,*E*)-5-ethyl-4-methyloct-6-en-4-yl)benzene (5ae)

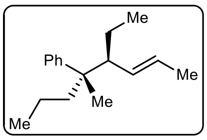

The title compound was prepared according to the general procedure **F**. The product was obtained as a colourless liquid (77%, *dr* 90:10). ^{1}H NMR (400 MHz, Chloroform-*d*) δ 7.17 (ddd, *J* = 13.3, 8.3, 6.6 Hz, 4H), 7.09 – 7.03 (m, 1H), 5.12 (dq, *J* = 15.2, 6.3 Hz, 1H), 5.00 – 4.90 (m, 1H), 1.81 – 1.72 (m, 2H), 1.52 (dd, *J* = 6.3, 1.6 Hz, 3H), 1.45 – 1.36 (m, 2H), 1.17 (s, 3H), 1.14 – 1.08 (m, 1H), 0.91 (dtd, *J* = 13.0, 6.9, 3.9 Hz, 1H), 0.78 (t, *J* = 7.2 Hz, 4H), 0.63 (t, *J* = 7.3 Hz, 3H); ^{13}C NMR (101 MHz, Chloroform-*d*) δ 147.03, 131.81, 127.42, 127.28, 126.84, 124.99, 57.03, 43.93, 40.83, 22.55, 21.81, 18.01, 17.50, 14.98, 12.98; **HRMS** (APCI) [M-H]$^+$, calculated for $C_{17}H_{25}$; 229.1951; found 229.1940.

Determination of the relative configuration

The relative configuration of compound **5u** was determined by X-Ray analysis of their hydrazine derivative **5ub**, prepared according to the following procedures.

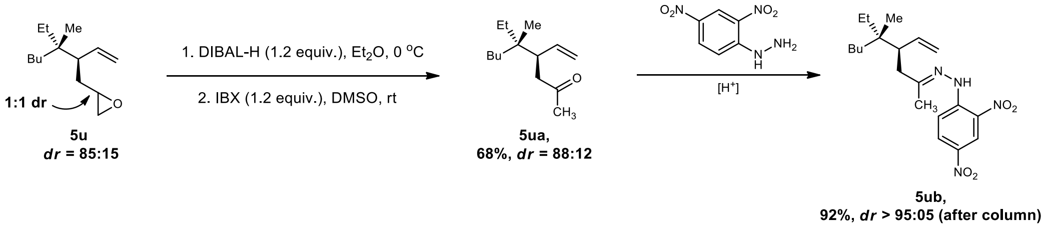

In order to determine the relative configuration of **5u**, it was reduced with DIBAL-H and oxidized with IBX (2-iodoxybenzoic acid) to give the ketone **5ua.** To a solution of ketone **5ua** (30 mg, 0.9 mmol, 1.0 equiv) and 2,4-dinitrophenylhydrazine (26 mg, 0.13, 1.1 equiv.) in EtOH (2 ml), conc. H_2SO_4 (1 drop) was added and the reaction mixture was stirred at 70 °C overnight. After completion of the reaction (as monitored by TLC), water (5 ml) was added and the reaction mixture was extracted by ethyl acetate (3 x 5 ml). The combined organic layer was dried over anhydrous sodium sulphate and evaporated to give the crude product which was further purified by column chromatography using 5-10% diethyl ether in petroleum ether as eluent. The product was obtained as a yellow-orange solid which was recrystallized using dichloromethane and hexane (1:1).

(*E*)-1-(2,4-dinitrophenyl)-2-((4R*,5S*)-5-ethyl-5-methyl-4-vinylnonan-2-ylidene)hydrazine (5ub)

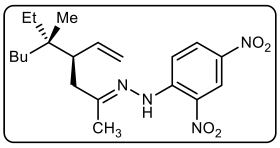

The compound **5ub** was obtained as yellow-orange solid in 92% yield. E/Z > 99:01. *dr* > 95:05. ^{1}H NMR (400 MHz, Chloroform-*d*) δ 10.98 (s, 1H), 9.12 (d, *J* = 2.6 Hz, 1H), 8.29 (dd, *J* = 9.6, 2.6 Hz, 1H), 7.94 (d, *J* = 9.7 Hz, 1H), 5.74 – 5.52 (m, 1H), 4.99 (dd, *J* = 10.2, 2.1 Hz, 1H), 4.90 (dd, *J* = 17.0, 2.1 Hz, 1H), 2.56 (d, *J* = 10.6 Hz, 1H), 2.41 – 2.30 (m, 2H), 1.99 (s, 3H), 1.35 – 1.25 (m, 8H), 0.93 (t, *J* = 7.0 Hz, 3H), 0.84 – 0.79 (m, 6H); ^{13}C NMR (101 MHz, Chloroform-*d*) δ 158.29, 145.15, 138.44, 137.53, 129.93, 128.92, 123.54, 116.64, 116.49, 48.78, 38.65, 37.41, 35.84, 29.43, 25.47, 23.63, 21.34, 16.24, 14.21, 7.82.

iCrystal structure for 5ub (CCDC number: 2129117)

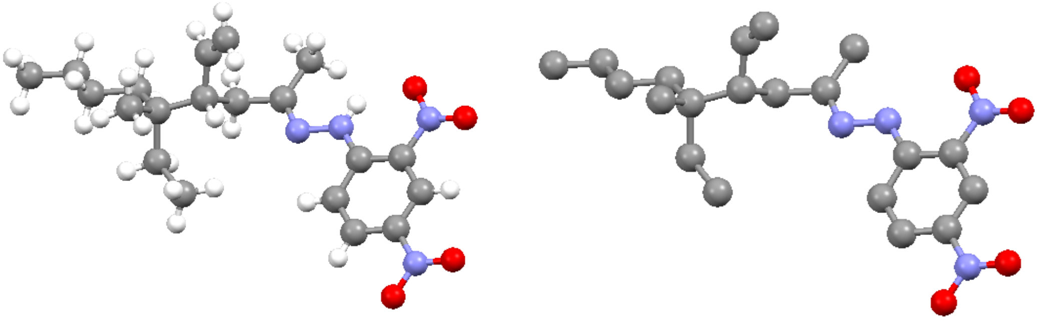

Crystal data and structure refinement of compound 5ub

Compound	5ub
CCDC number	2129117
Empirical formula	$C_{20}H_{30}N_4O_4$
Formula weight	390.48
Temperature/K	200.15
Crystal system	monoclinic
Space group	$P2_1/c$
a/Å	19.475(5)
b/Å	15.833(4)
c/Å	7.0608(18)
α/°	90
β/°	100.278(6)
γ/°	90
Volume/Å3	2142.2(9)
Z	4
ρ_{calc}g/cm3	1.211
μ/mm^{-1}	0.085
F(000)	840.0
Crystal size/mm3	0.15 × 0.12 × 0.06
Radiation	MoKα (λ = 0.71073)
2Θ range for data collection/°	3.336 to 47.198
Index ranges	-21 ≤ h ≤ 20, -17 ≤ k ≤ 17, -6 ≤ l ≤ 7
Reflections collected	8259
Independent reflections	1753 [R_{int} = 0.1164, R_{sigma} = 0.2409]
Data/restraints/parameters	1753/258/308
Goodness-of-fit on F^2	0.924
Final R indexes [I>=2σ (I)]	R_1 = 0.0831, wR_2 = 0.1483
Final R indexes [all data]	R_1 = 0.2829, wR_2 = 0.2197
Largest diff. peak/hole / e Å^{-3}	0.21/-0.23

Determination of the enantiospecificity

The enantioselective addition of a Grignard was performed based on the catalytic enantioselective copper-catalyzed carbomagnesation that our group has recently developed[1,2] on cyclopropenes **1a** and **1d** (see the general procedure A for more details)

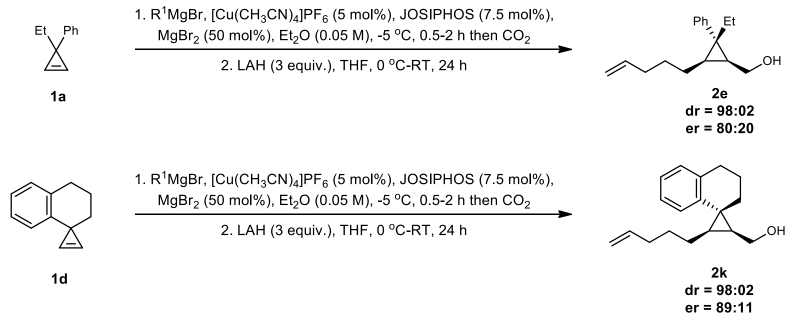

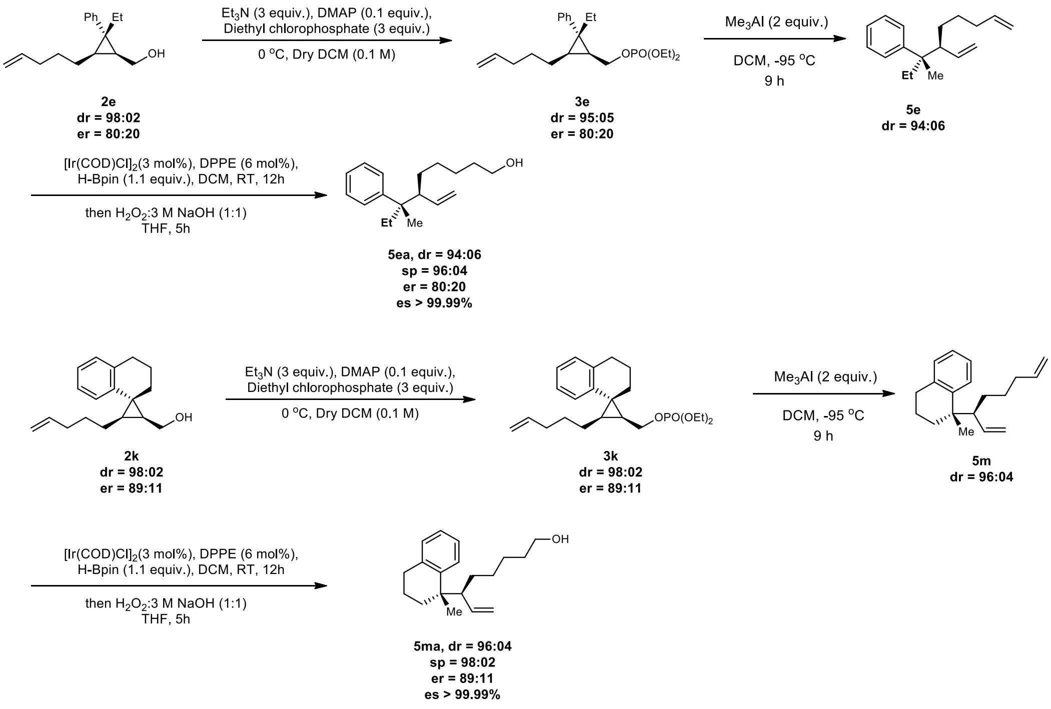

The product could not be separated by HPLC due to its non-polar nature and lack of functional groups. So, to determine the enantiomeric ratio of the reaction, the final product was further functionalized using hydroboration-oxidation sequence to transform the olefin into an alcohol[8]. It was then separated by HPLC.

(6S,7S)-7-methyl-7-phenyl-6-vinylnonan-1-ol (5ea)

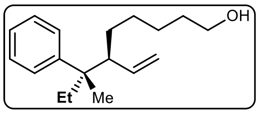

The title compound was prepared according to the literature procedure[8] from **5e**. The product was obtained as a colourless liquid (70%, *dr* 94:06). ^{1}H NMR (400 MHz, Chloroform-*d*) δ 7.22 – 7.15 (m, 4H), 7.12 – 7.04 (m, 1H), 5.33 (dt, *J* = 17.0, 9.9 Hz, 1H), 4.85 (dd, *J* = 10.3, 2.3 Hz, 1H), 4.71 (dd, *J* = 17.1, 2.3 Hz, 1H), 3.50 (t, *J* = 6.6 Hz, 2H), 2.00 – 1.81 (m, 2H), 1.51 (dd, *J* = 14.0, 7.3 Hz, 1H), 1.43 – 1.35 (m, 3H), 1.31 – 1.21 (m, 2H), 1.18 (s, 3H), 1.15 – 1.09 (m, 1H), 1.06

– 0.73 (m, 3H), 0.61 (t, J = 7.3 Hz, 3H); ^{13}C NMR (101 MHz, Chloroform-d) δ 146.07, 139.63, 127.54, 127.40, 125.18, 116.21, 62.98, 55.81, 43.78, 32.66, 30.99, 28.45, 27.80, 25.57, 21.28, 8.69. **HRMS** (APCI) [M+H]$^+$, calculated for $C_{18}H_{29}O$; 261.2213; found 261.2241.

(S)-6-((S)-1-methyl-1,2,3,4-tetrahydronaphthalen-1-yl)oct-7-en-1-ol (5ma)

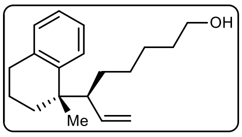

The title compound was prepared according to the literature procedure[8] from **5m**. The product was obtained as a colourless liquid (74%, *dr* 96:04). ^{1}H NMR (400 MHz, Chloroform-d) δ 7.21 – 7.14 (m, 1H), 7.06 (ddd, J = 8.0, 6.5, 2.2 Hz, 1H), 7.02 – 6.89 (m, 2H), 5.59 (dt, J = 16.9, 9.9 Hz, 1H), 5.13 – 4.93 (m, 2H), 3.46 (t, J = 6.6 Hz, 2H), 2.68 – 2.55 (m, 2H), 2.29 (td, J = 10.1, 2.1 Hz, 1H), 1.78 – 1.59 (m, 3H), 1.56 (d, J = 4.1 Hz, 1H), 1.51 – 1.42 (m, 1H), 1.39 – 1.29 (m, 2H), 1.28 – 1.16 (m, 2H), 1.15 (s, 3H), 1.06 – 0.82 (m, 4H); ^{13}C NMR (101 MHz, Chloroform-d) δ 144.37, 139.65, 137.81, 128.97, 126.39, 125.87, 124.95, 117.20, 62.96, 55.08, 40.20, 32.61, 31.38, 30.94, 30.56, 29.18, 27.75, 25.36, 19.63. **HRMS** (APCI) [M+H]$^+$, calculated for $C_{19}H_{29}O$; 273.2213; found 273.2234.

HPLC chromatogram of compound **2e**

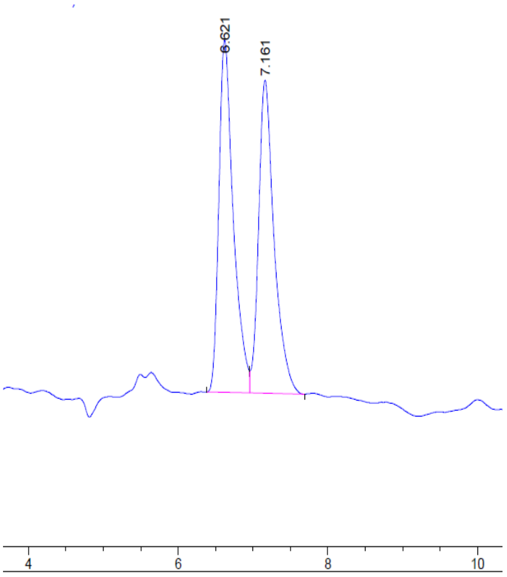

Peak #	RetTime [min]	Type	Width [min]	Area [mAU*s]	Height [mAU]	Area %
1	6.621	BV	0.1989	44.82578	3.33350	51.1757
2	7.161	VB	0.2146	42.76606	2.95940	48.8243

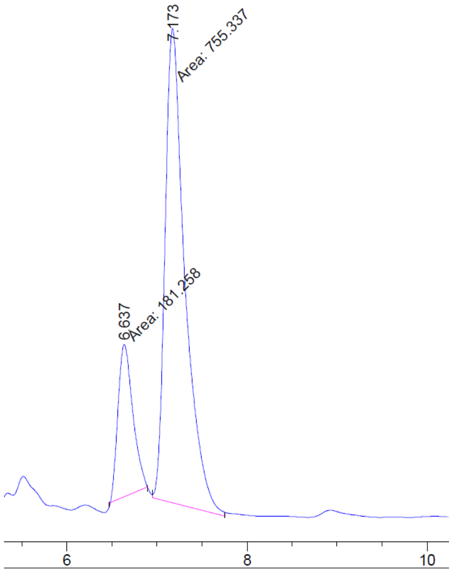

Peak #	RetTime [min]	Type	Width [min]	Area [mAU*s]	Height [mAU]	Area %
1	6.637	MM	0.1913	181.25816	15.79117	19.3529
2	7.173	MM	0.2558	755.33746	49.21220	80.6471

HPLC chromatogram of compound **5ea**

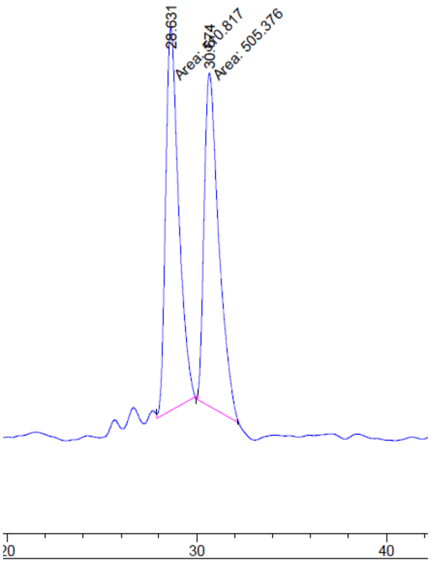

Peak #	RetTime [min]	Type	Width [min]	Area [mAU*s]	Height [mAU]	Area %
1	28.631	MM	0.7932	510.81708	10.73310	50.2677
2	30.674	MM	0.9016	505.37643	9.34212	49.7323

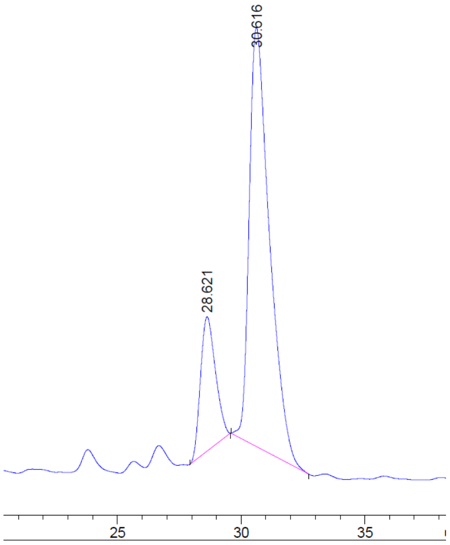

Peak #	RetTime [min]	Type	Width [min]	Area [mAU*s]	Height [mAU]	Area %
1	28.621	BB	0.6392	166.58199	3.94874	19.1819
2	30.616	BB	0.8236	701.85089	12.32002	80.8181

HPLC chromatogram of compound **2k**

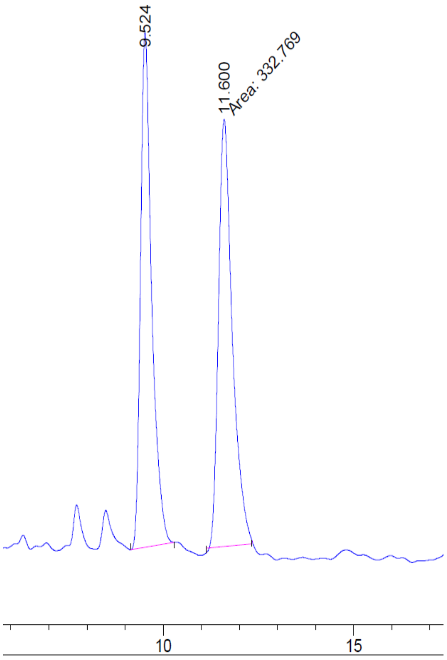

Peak #	RetTime [min]	Type	Width [min]	Area [mAU*s]	Height [mAU]	Area %
1	9.524	BB	0.3023	322.54639	15.85797	49.2200
2	11.600	MM	0.4211	332.76904	13.17162	50.7800

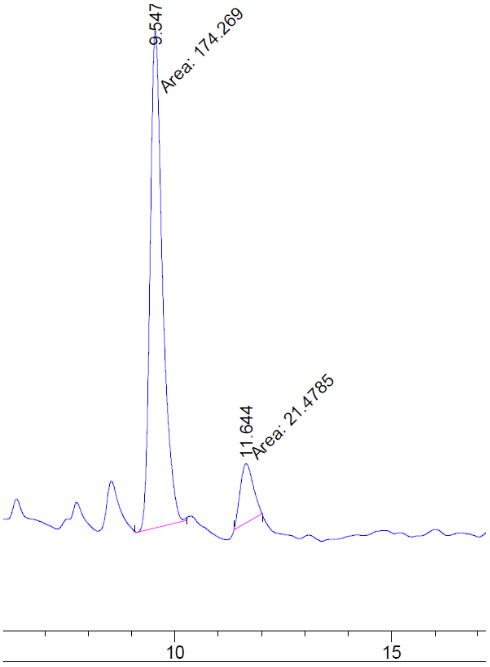

Peak #	RetTime [min]	Type	Width [min]	Area [mAU*s]	Height [mAU]	Area %
1	9.547	MM	0.3336	174.26947	8.70700	89.0275
2	11.644	MM	0.3447	21.47852	1.03857	10.9725

HPLC chromatogram of compound **5ma**

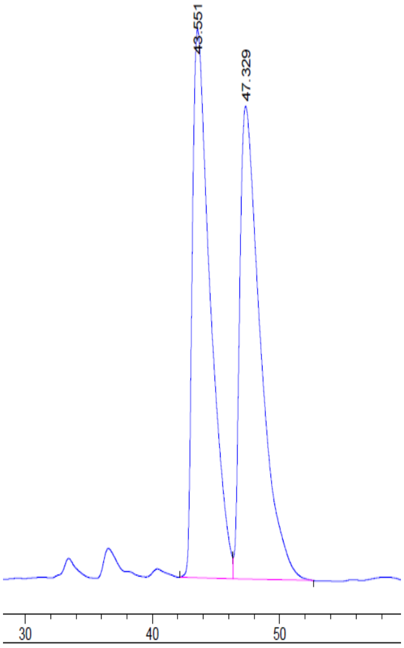

Peak #	RetTime [min]	Type	Width [min]	Area [mAU*s]	Height [mAU]	Area %
1	43.551	BV	1.4152	3894.01147	39.77504	49.8451
2	47.329	VB	1.5943	3918.21484	34.31047	50.1549

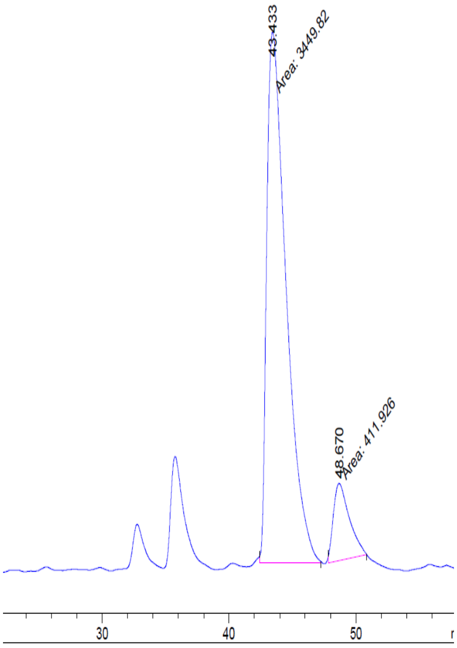

Peak #	RetTime [min]	Type	Width [min]	Area [mAU*s]	Height [mAU]	Area %
1	43.433	MM	1.7767	3449.81934	32.36232	89.3332
2	48.670	MM	1.4608	411.92645	4.69965	10.6668

Spectral Data

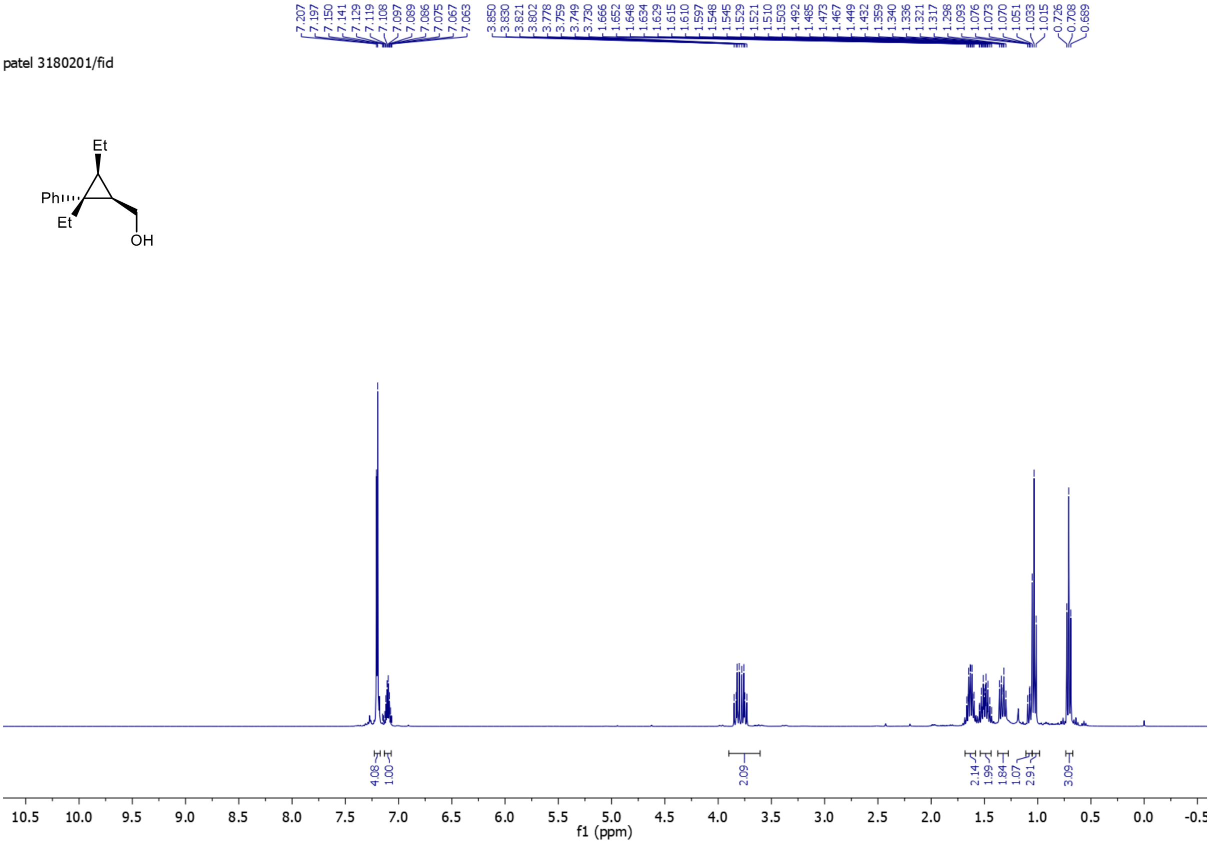

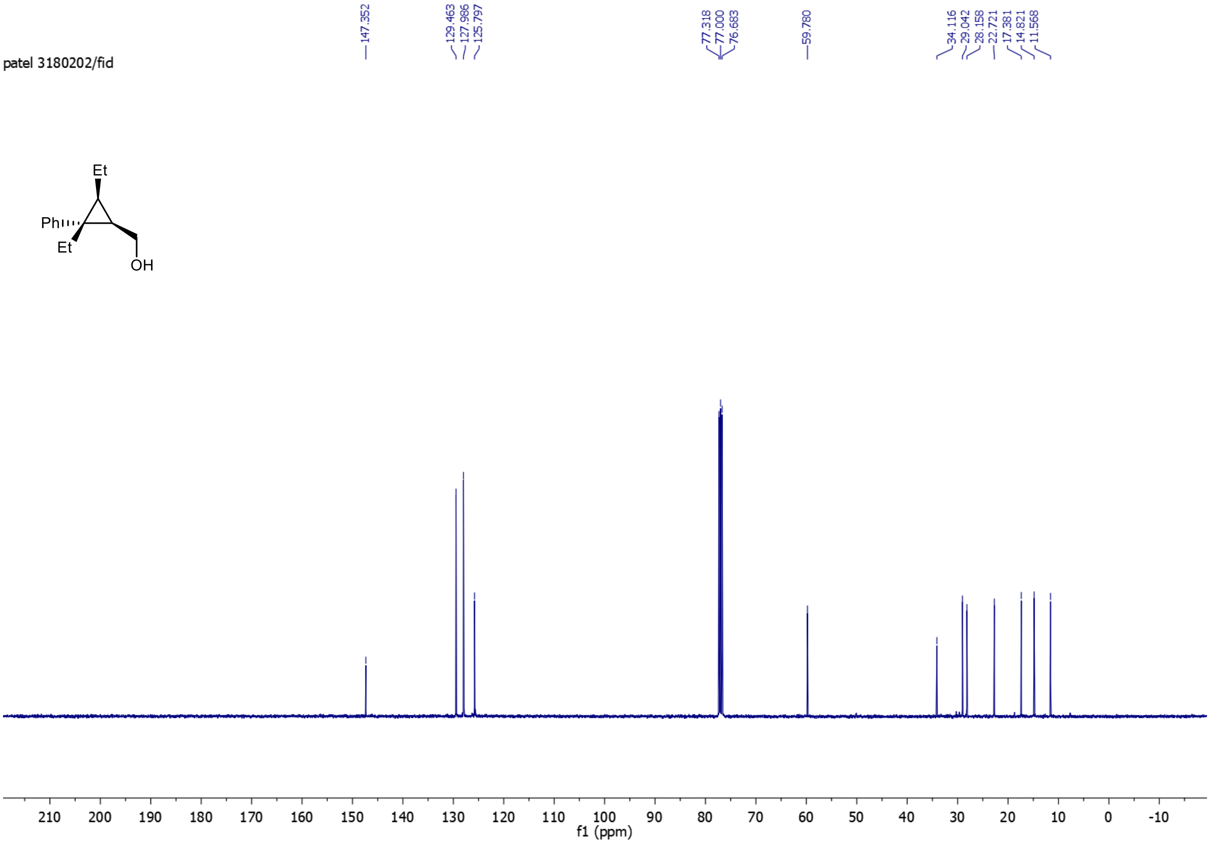

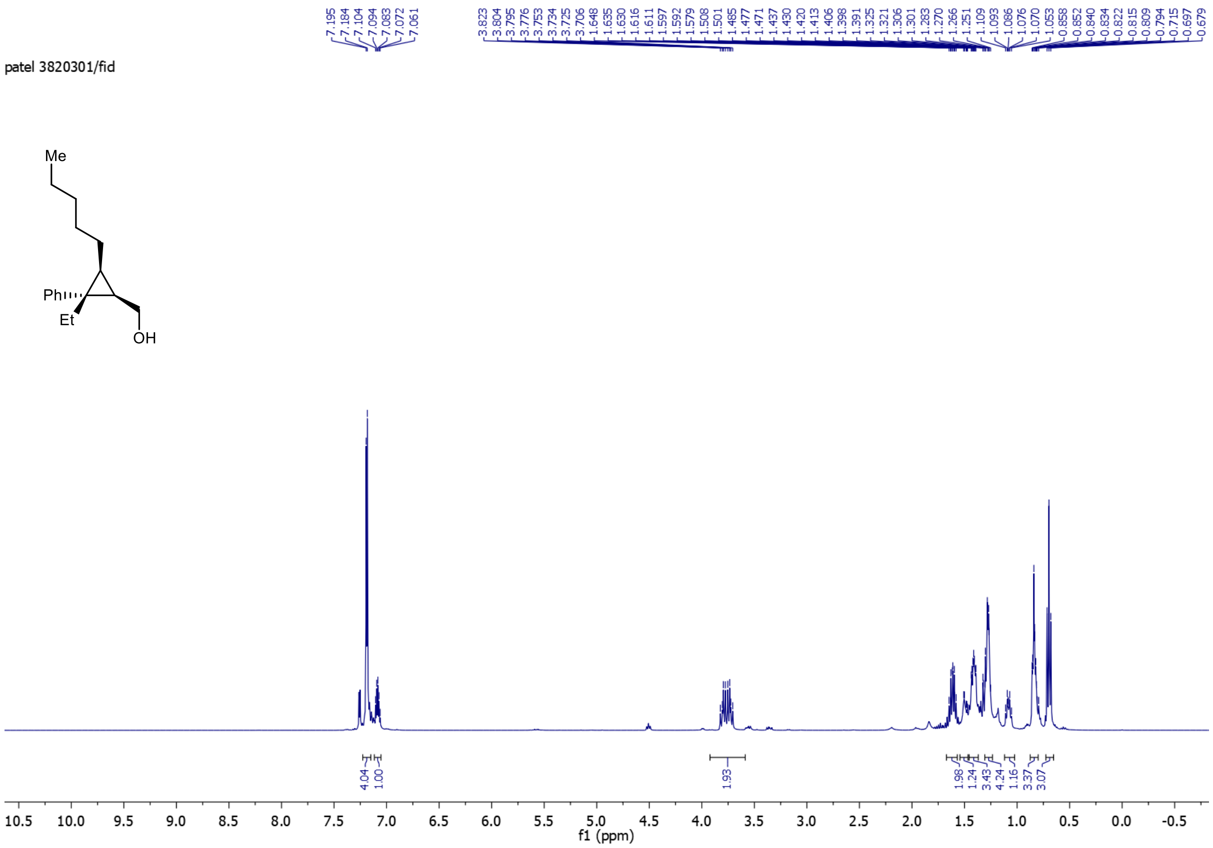
patel 3820301/fid
7.195
7.184
7.104
7.094
7.083
7.072
7.061
3.823
3.804
3.795
3.776
3.753
3.734
3.725
3.706
1.648
1.635
1.630
1.616
1.611
1.597
1.592
1.579
1.508
1.501
1.485
1.477
1.471
1.437
1.430
1.420
1.413
1.406
1.398
1.391
1.325
1.321
1.306
1.301
1.283
1.270
1.266
1.251
1.109
1.093
1.086
1.076
1.070
1.053
0.858
0.852
0.840
0.834
0.822
0.815
0.809
0.794
0.715
0.697
0.679
Me
Ph
Et
OH
4.04
1.00
1.93
1.98
1.24
3.43
4.24
1.16
3.37
3.07
10.5 10.0 9.5 9.0 8.5 8.0 7.5 7.0 6.5 6.0 5.5 5.0 4.5 4.0 3.5 3.0 2.5 2.0 1.5 1.0 0.5 0.0 -0.5
f1 (ppm)

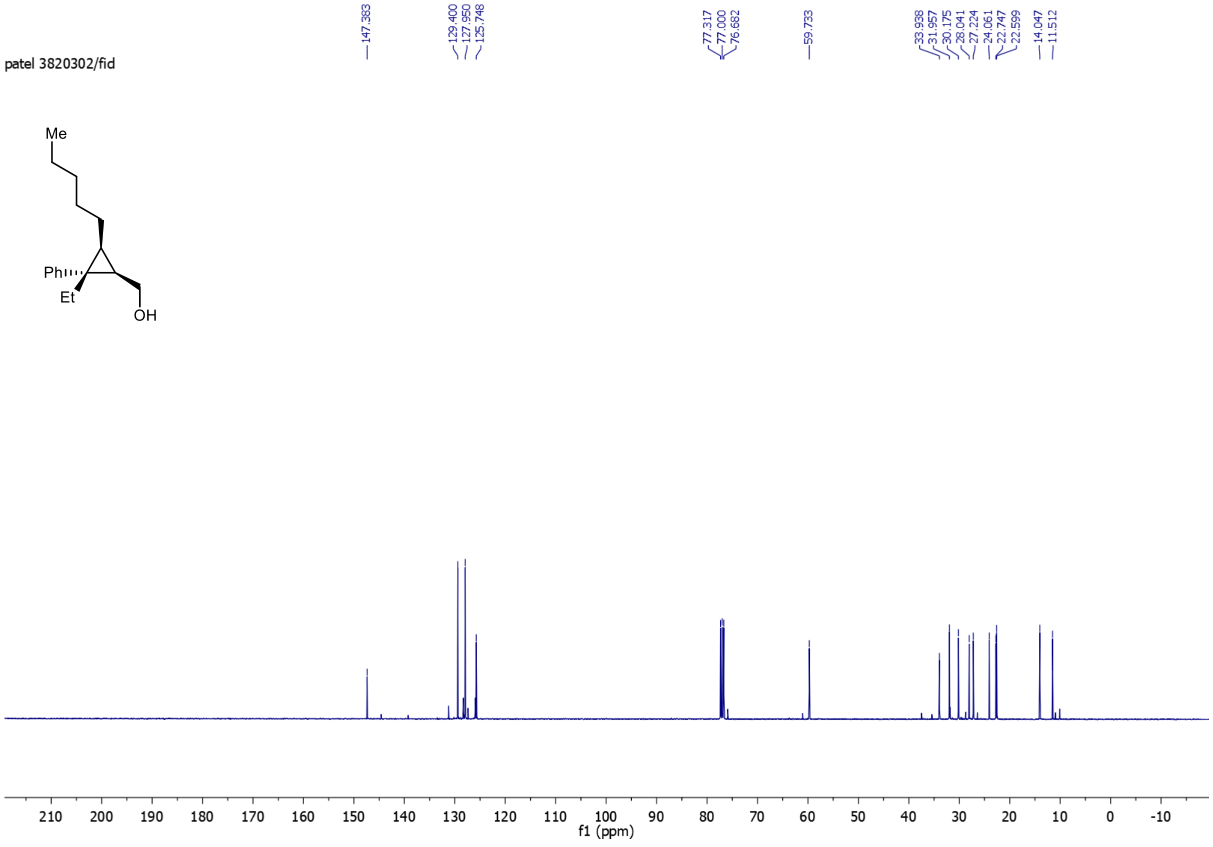
patel 3820302/fid
147.383
129.400
127.950
125.748
77.317
77.000
76.682
59.733
33.938
31.957
30.175
28.041
27.224
24.061
22.747
22.599
14.047
11.512
Me
Ph
Et
OH
210 200 190 180 170 160 150 140 130 120 110 100 90 80 70 60 50 40 30 20 10 0 -10
f1 (ppm)

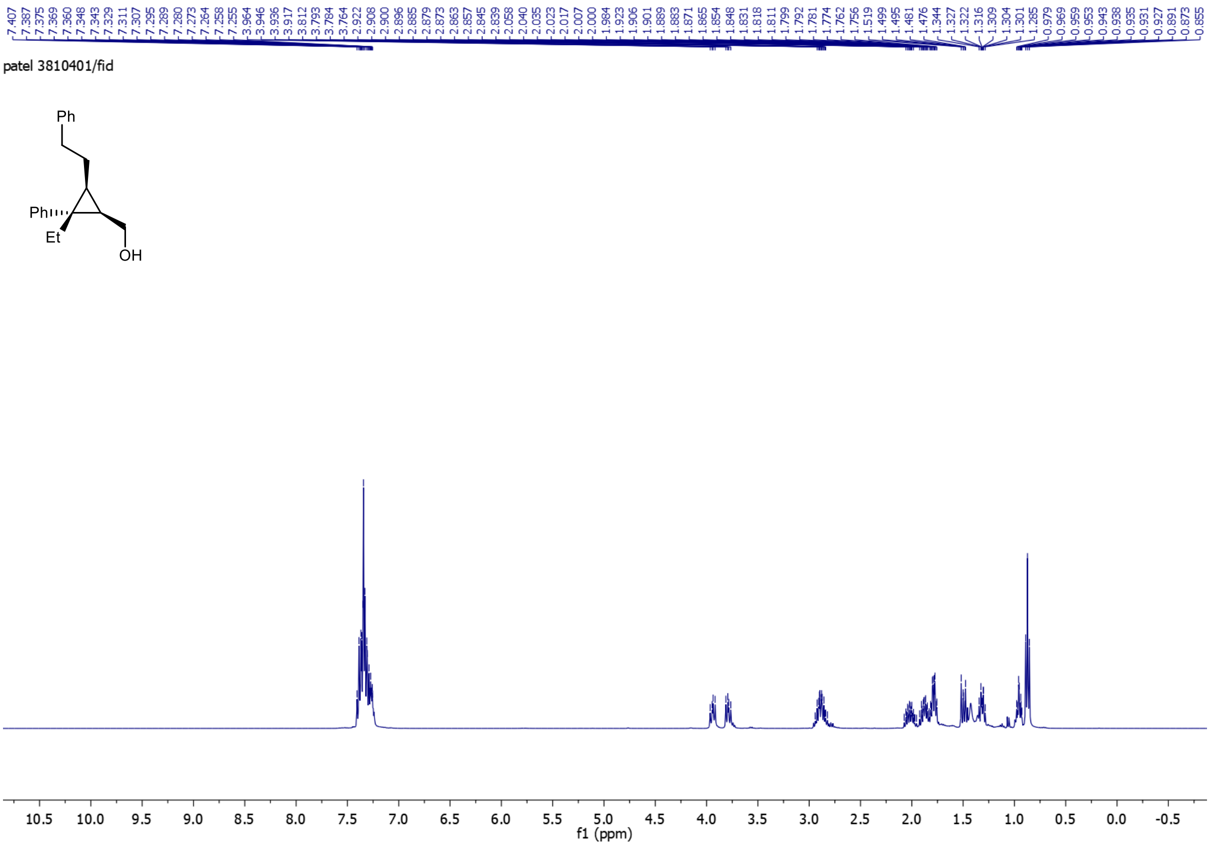
7.407
7.387
7.375
7.369
7.360
7.348
7.343
7.329
7.311
7.307
7.295
7.289
7.280
7.273
7.264
7.258
7.255
3.964
3.946
3.936
3.917
3.812
3.793
3.784
3.764
2.922
2.908
2.900
2.896
2.885
2.879
2.873
2.863
2.857
2.845
2.839
2.058
2.040
2.035
2.023
2.017
2.007
2.000
1.984
1.923
1.906
1.901
1.889
1.883
1.871
1.865
1.854
1.848
1.831
1.818
1.811
1.799
1.792
1.781
1.774
1.762
1.756
1.519
1.499
1.495
1.481
1.476
1.344
1.327
1.322
1.316
1.309
1.304
1.301
1.285
0.979
0.969
0.959
0.953
0.943
0.938
0.935
0.931
0.927
0.891
0.873
0.855
patel 3810401/fid
Ph
Ph
Et
OH
10.5 10.0 9.5 9.0 8.5 8.0 7.5 7.0 6.5 6.0 5.5 5.0 4.5 4.0 3.5 3.0 2.5 2.0 1.5 1.0 0.5 0.0 -0.5
f1 (ppm)

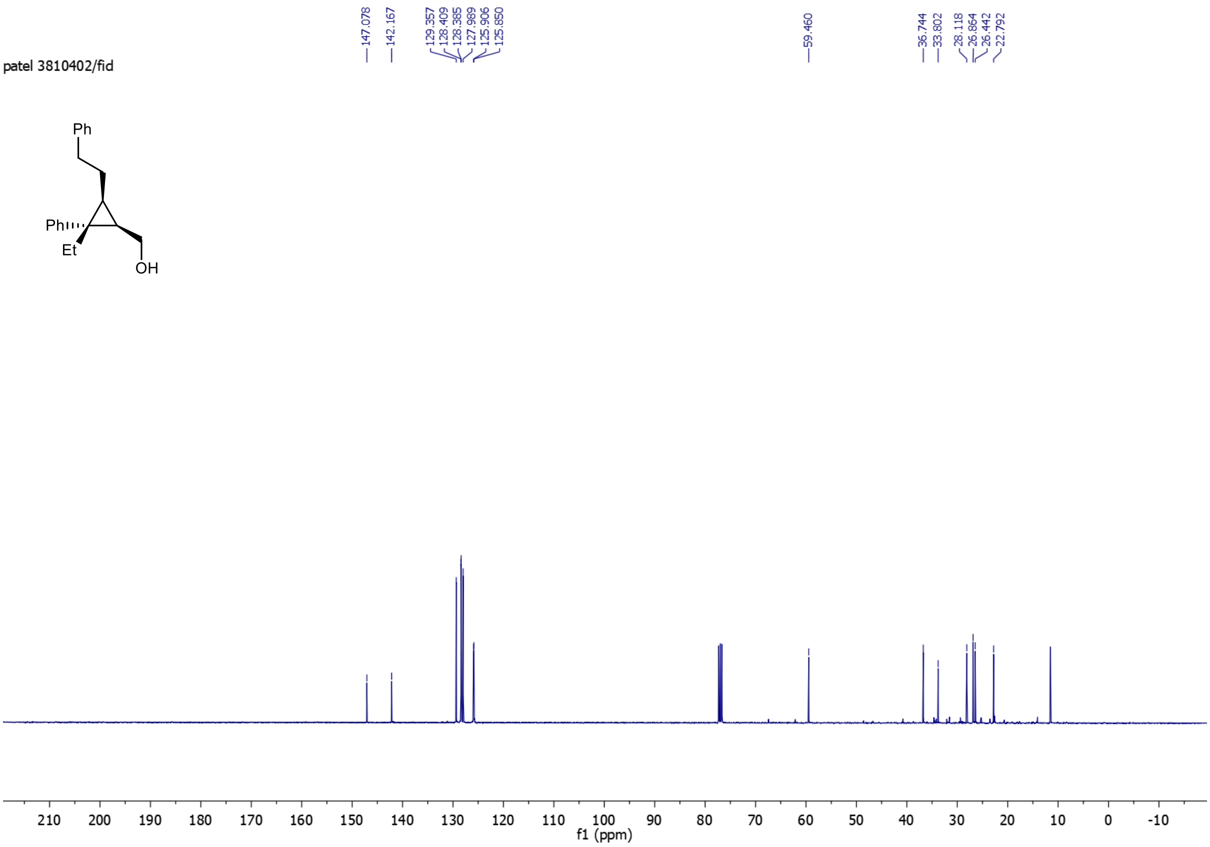
147.078
142.167
129.357
128.409
128.385
127.989
125.906
125.850
59.460
36.744
33.802
28.118
26.864
26.442
22.792
patel 3810402/fid
Ph
Ph
Et
OH
210 200 190 180 170 160 150 140 130 120 110 100 90 80 70 60 50 40 30 20 10 0 -10
f1 (ppm)

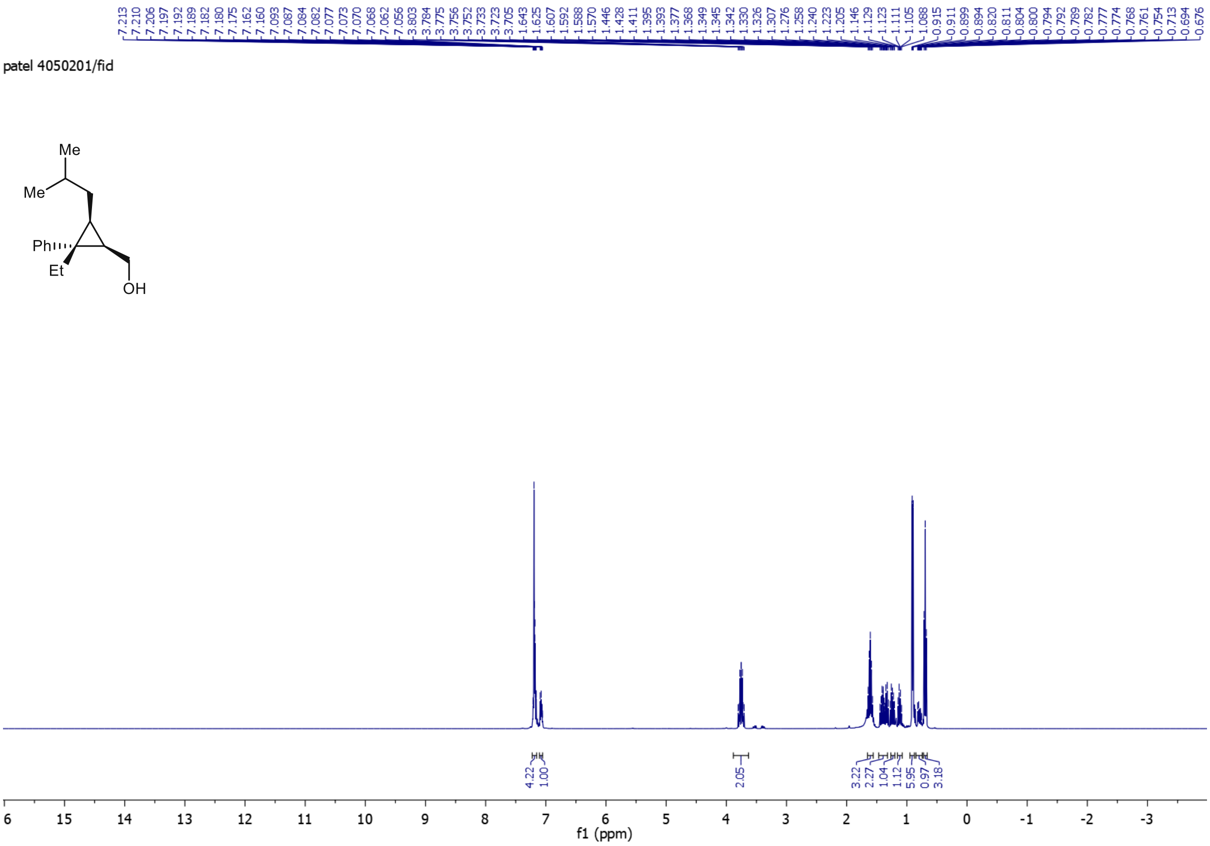
7.213
7.210
7.206
7.197
7.192
7.189
7.182
7.180
7.175
7.162
7.160
7.093
7.087
7.084
7.082
7.077
7.073
7.070
7.068
7.062
7.056
3.803
3.784
3.775
3.756
3.752
3.733
3.723
3.705
1.643
1.625
1.607
1.592
1.588
1.570
1.446
1.428
1.411
1.395
1.393
1.377
1.368
1.349
1.345
1.342
1.330
1.326
1.307
1.276
1.258
1.240
1.223
1.205
1.146
1.129
1.123
1.111
1.105
1.088
0.915
0.911
0.899
0.894
0.820
0.811
0.804
0.800
0.794
0.792
0.789
0.782
0.777
0.774
0.768
0.761
0.754
0.713
0.694
0.676
patel 4050201/fid
Me
Me
Ph
Et
OH
4.22
1.00
2.05
3.22
2.27
1.04
1.12
5.95
0.97
3.18
f1 (ppm)

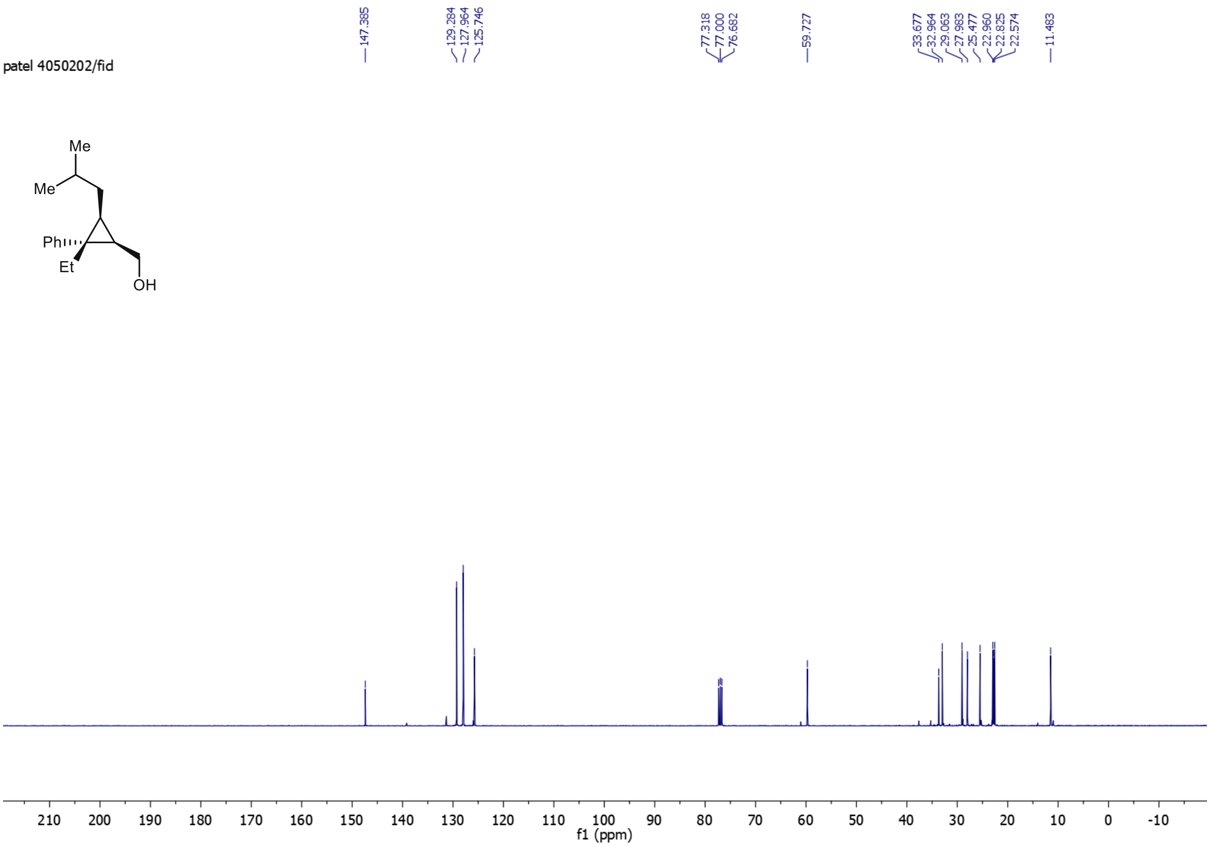
147.385
129.284
127.964
125.746
77.318
77.000
76.682
59.727
33.677
32.964
29.063
27.983
25.477
22.960
22.825
22.574
11.483
patel 4050202/fid
Me
Me
Ph
Et
OH
f1 (ppm)

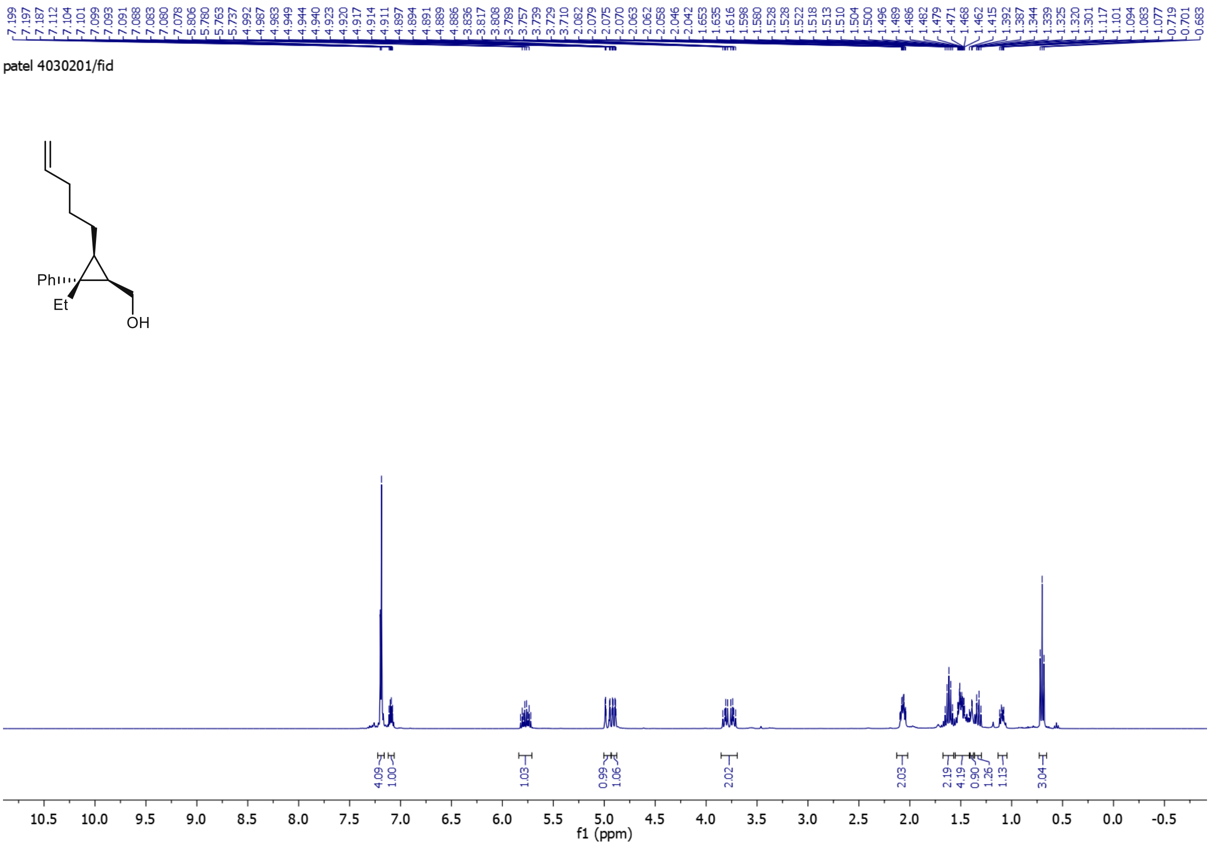
7.199
7.197
7.187
7.112
7.104
7.101
7.099
7.093
7.091
7.088
7.083
7.080
7.078
5.806
5.780
5.763
5.737
4.992
4.987
4.983
4.949
4.944
4.940
4.923
4.920
4.917
4.914
4.911
4.897
4.894
4.891
4.889
4.886
3.836
3.817
3.808
3.789
3.757
3.739
3.729
3.710
2.082
2.079
2.075
2.070
2.063
2.062
2.058
2.046
2.042
1.653
1.635
1.616
1.598
1.580
1.528
1.528
1.522
1.518
1.513
1.510
1.504
1.500
1.496
1.489
1.486
1.482
1.479
1.471
1.468
1.462
1.415
1.392
1.387
1.344
1.339
1.325
1.320
1.301
1.117
1.101
1.094
1.083
1.077
0.719
0.701
0.683
patel 4030201/fid
Ph
Et
OH
4.09
1.00
1.03
0.99
1.06
2.02
2.03
2.19
4.19
0.90
1.26
1.13
3.04
10.5
10.0
9.5
9.0
8.5
8.0
7.5
7.0
6.5
6.0
5.5
5.0
4.5
4.0
3.5
3.0
2.5
2.0
1.5
1.0
0.5
0.0
-0.5
f1 (ppm)

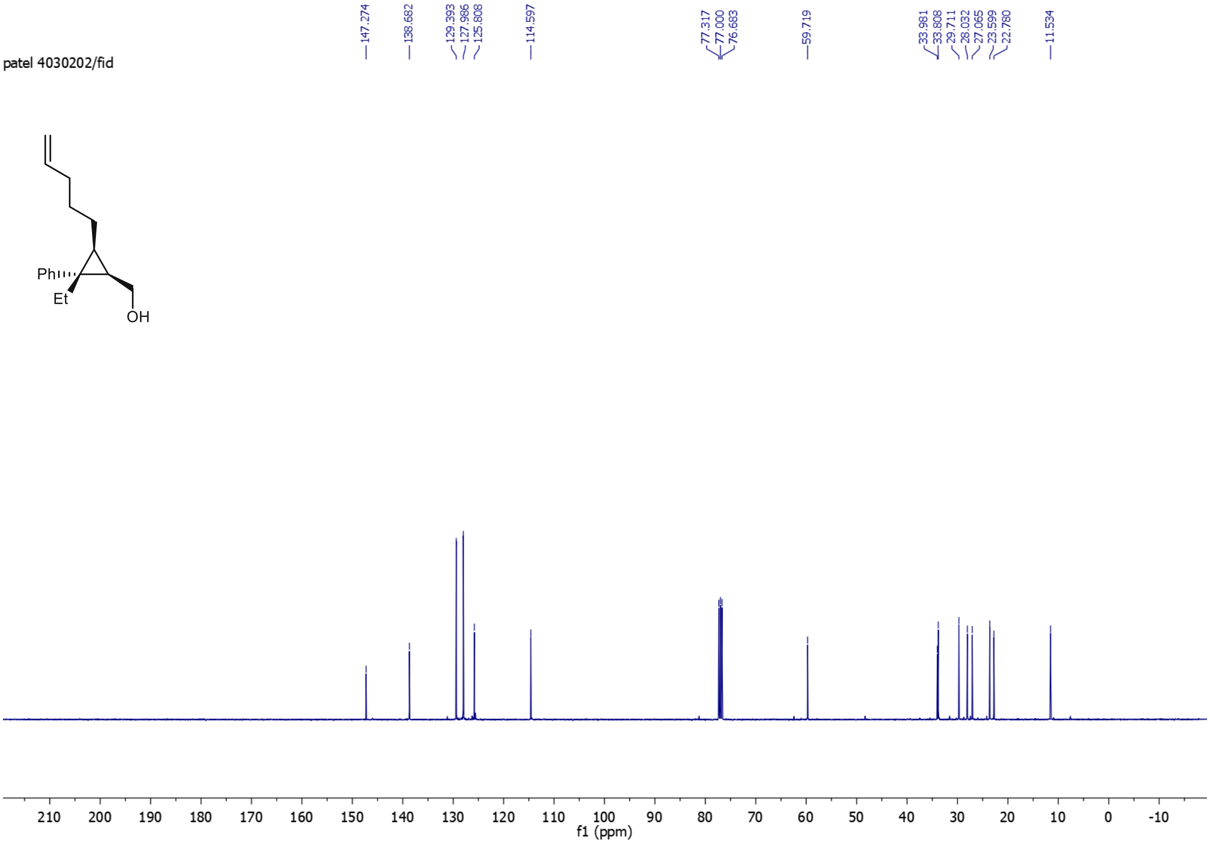
147.274
138.682
129.393
127.986
125.808
114.597
77.317
77.000
76.683
59.719
33.981
33.808
29.711
28.032
27.065
23.599
22.780
11.534
patel 4030202/fid
Ph
Et
OH
210
200
190
180
170
160
150
140
130
120
110
100
90
80
70
60
50
40
30
20
10
0
-10
f1 (ppm)

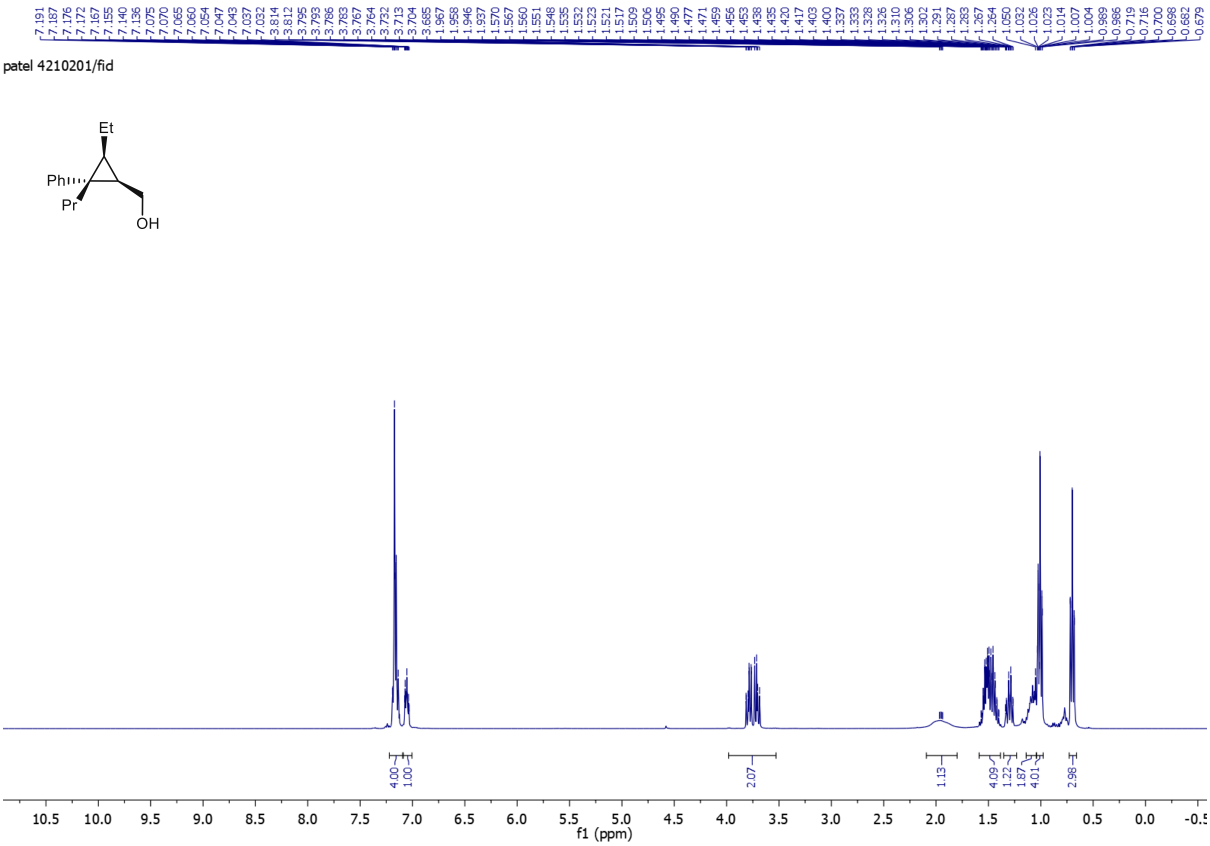
7.191 7.187 7.176 7.172 7.167 7.155 7.140 7.136 7.075 7.070 7.065 7.060 7.054 7.047 7.043 7.037 7.032 3.814 3.812 3.795 3.793 3.786 3.783 3.767 3.764 3.732 3.713 3.704 3.685 1.967 1.958 1.946 1.937 1.570 1.567 1.560 1.551 1.548 1.535 1.532 1.523 1.521 1.517 1.509 1.506 1.495 1.490 1.477 1.471 1.459 1.456 1.453 1.438 1.435 1.420 1.417 1.403 1.400 1.337 1.333 1.328 1.326 1.310 1.306 1.302 1.291 1.287 1.283 1.267 1.264 1.050 1.032 1.026 1.023 1.014 1.007 1.004 0.989 0.986 0.719 0.716 0.700 0.698 0.682 0.679
patel 4210201/fid
Et
Ph
Pr
OH
4.00 1.00 2.07 1.13 4.09 1.22 1.87 4.01 2.98
f1 (ppm)

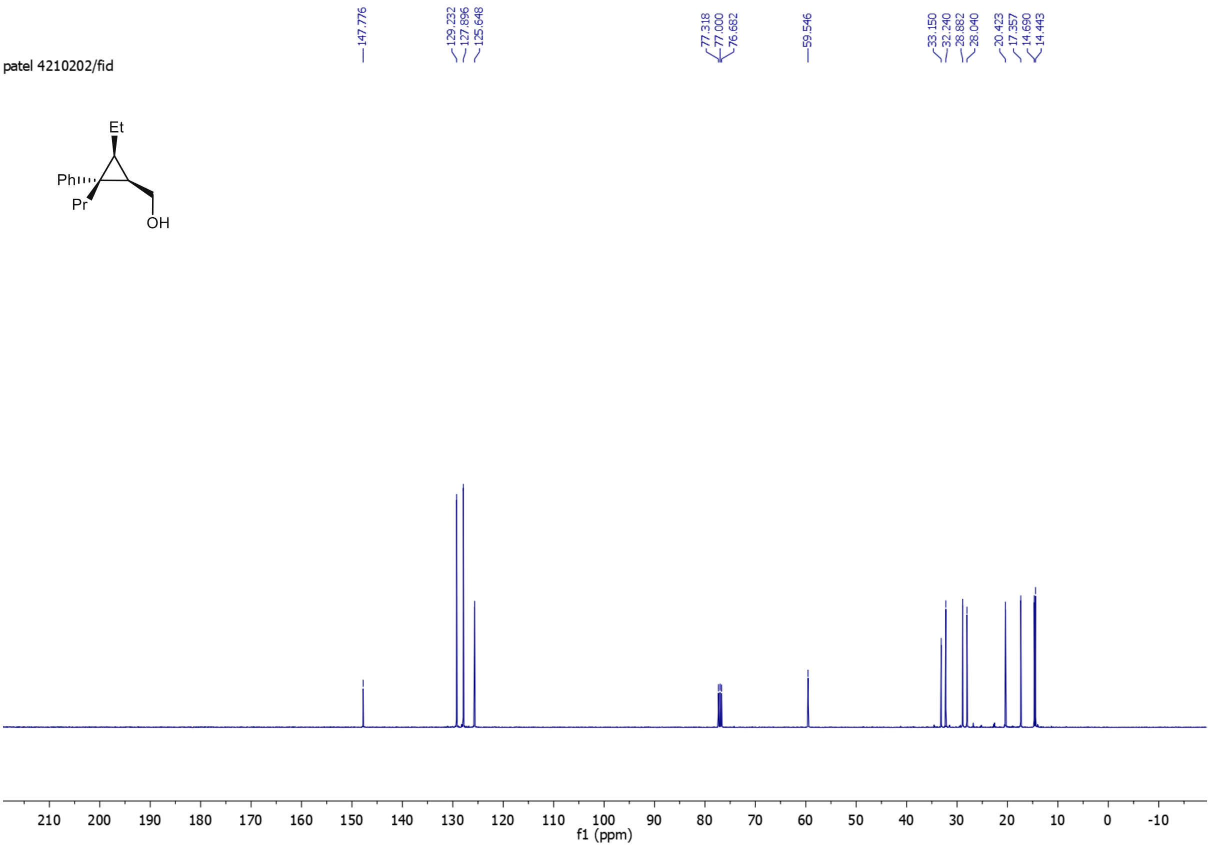
147.776
129.232 127.896 125.648
77.318 77.000 76.682
59.546
33.150 32.240 28.882 28.040 20.423 17.357 14.690 14.443
patel 4210202/fid
Et
Ph
Pr
OH
f1 (ppm)

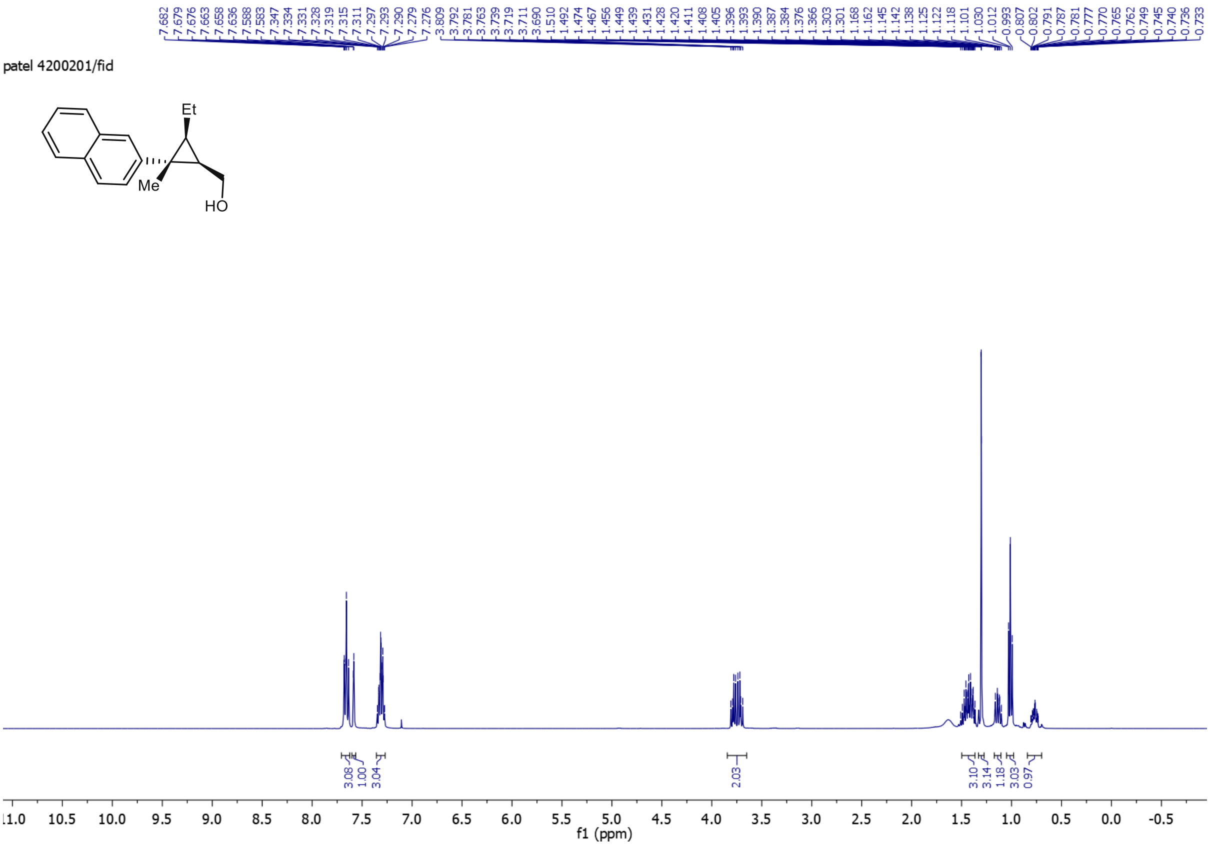
patel 4200201/fid
Et
Me
HO
7.682 7.679 7.676 7.663 7.658 7.636 7.588 7.583 7.347 7.334 7.331 7.328 7.319 7.315 7.311 7.297 7.293 7.290 7.279 7.276 3.809 3.792 3.781 3.763 3.739 3.719 3.711 3.690 1.510 1.492 1.474 1.467 1.456 1.449 1.439 1.431 1.428 1.420 1.411 1.408 1.405 1.396 1.393 1.390 1.387 1.384 1.376 1.366 1.303 1.301 1.168 1.162 1.145 1.142 1.138 1.125 1.122 1.118 1.101 1.030 1.012 0.993 0.807 0.802 0.791 0.787 0.781 0.777 0.770 0.765 0.762 0.749 0.745 0.740 0.736 0.733
3.08 1.00 3.04 2.03 3.10 3.14 1.18 3.03 0.97
f1 (ppm)

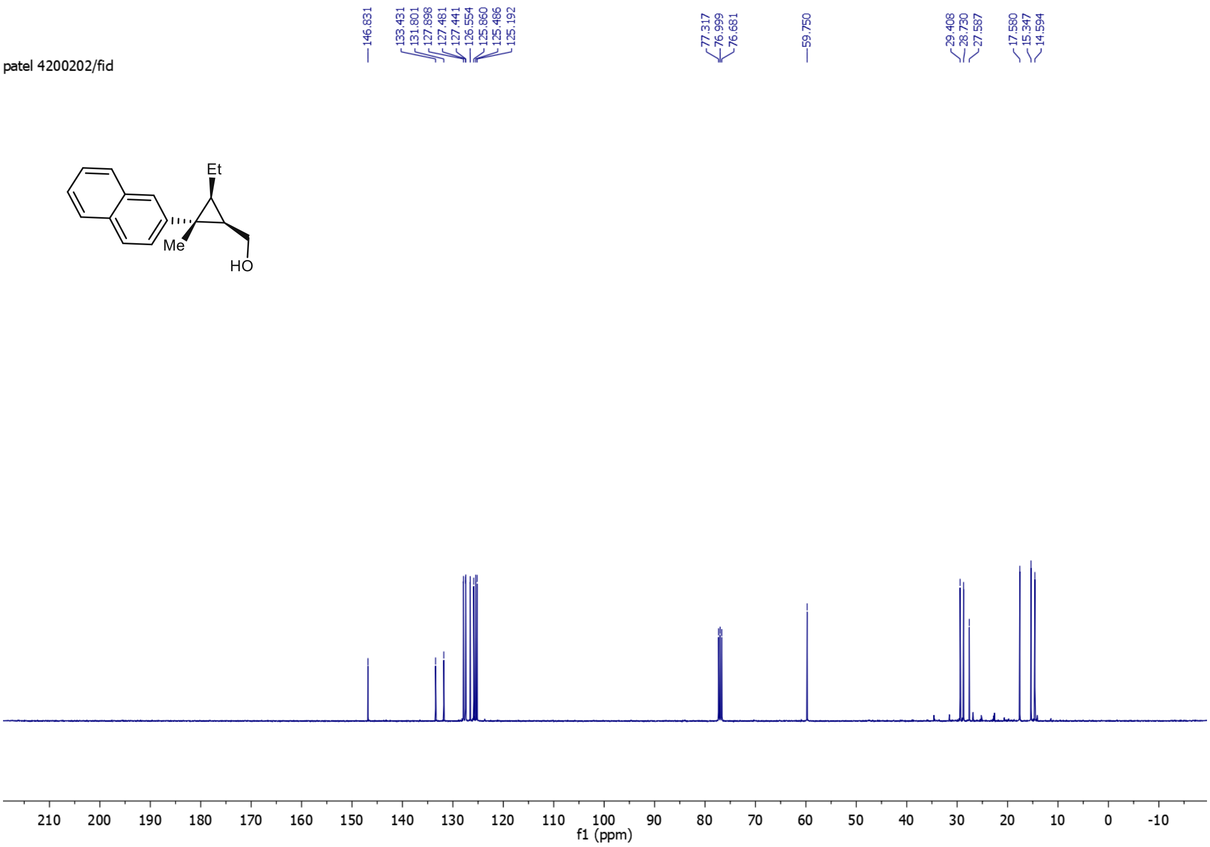
patel 4200202/fid
Et
Me
HO
146.831 133.431 131.801 127.898 127.481 127.441 126.554 125.860 125.486 125.192 77.317 76.999 76.681 59.750 29.408 28.730 27.587 17.580 15.347 14.594
f1 (ppm)

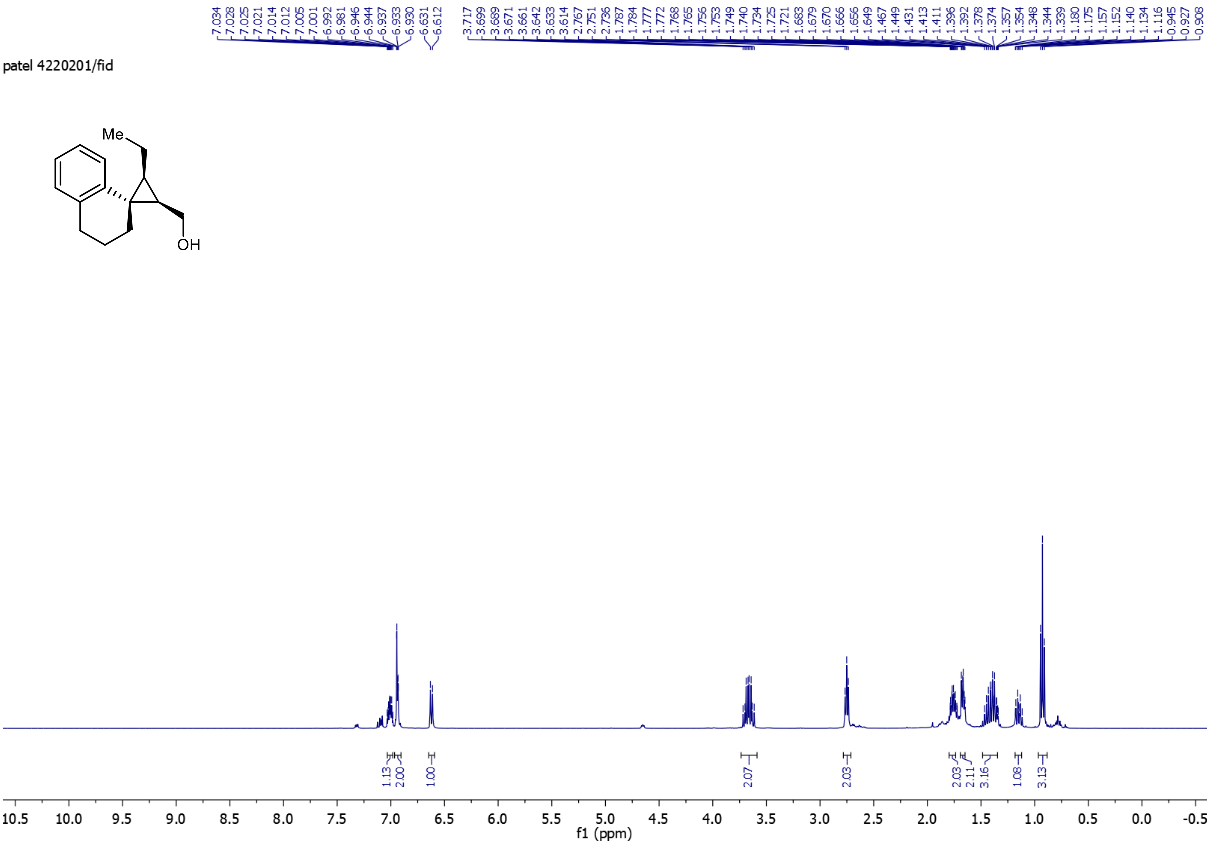
patel 4220201/fid
7.034 7.028 7.025 7.021 7.014 7.012 7.005 7.001 6.992 6.981 6.946 6.944 6.937 6.933 6.930 6.631 6.612
3.717 3.699 3.689 3.671 3.661 3.642 3.633 3.614 2.767 2.751 2.736 1.787 1.784 1.777 1.772 1.768 1.765 1.756 1.753 1.749 1.740 1.734 1.725 1.721 1.683 1.679 1.670 1.666 1.656 1.649 1.467 1.449 1.431 1.413 1.411 1.396 1.392 1.378 1.374 1.357 1.354 1.348 1.344 1.339 1.180 1.175 1.157 1.152 1.140 1.134 1.116 0.945 0.927 0.908
Me
OH
1.13 2.00 1.00 2.07 2.03 2.03 2.11 3.16 1.08 3.13
10.5 10.0 9.5 9.0 8.5 8.0 7.5 7.0 6.5 6.0 5.5 5.0 4.5 4.0 3.5 3.0 2.5 2.0 1.5 1.0 0.5 0.0 -0.5
f1 (ppm)

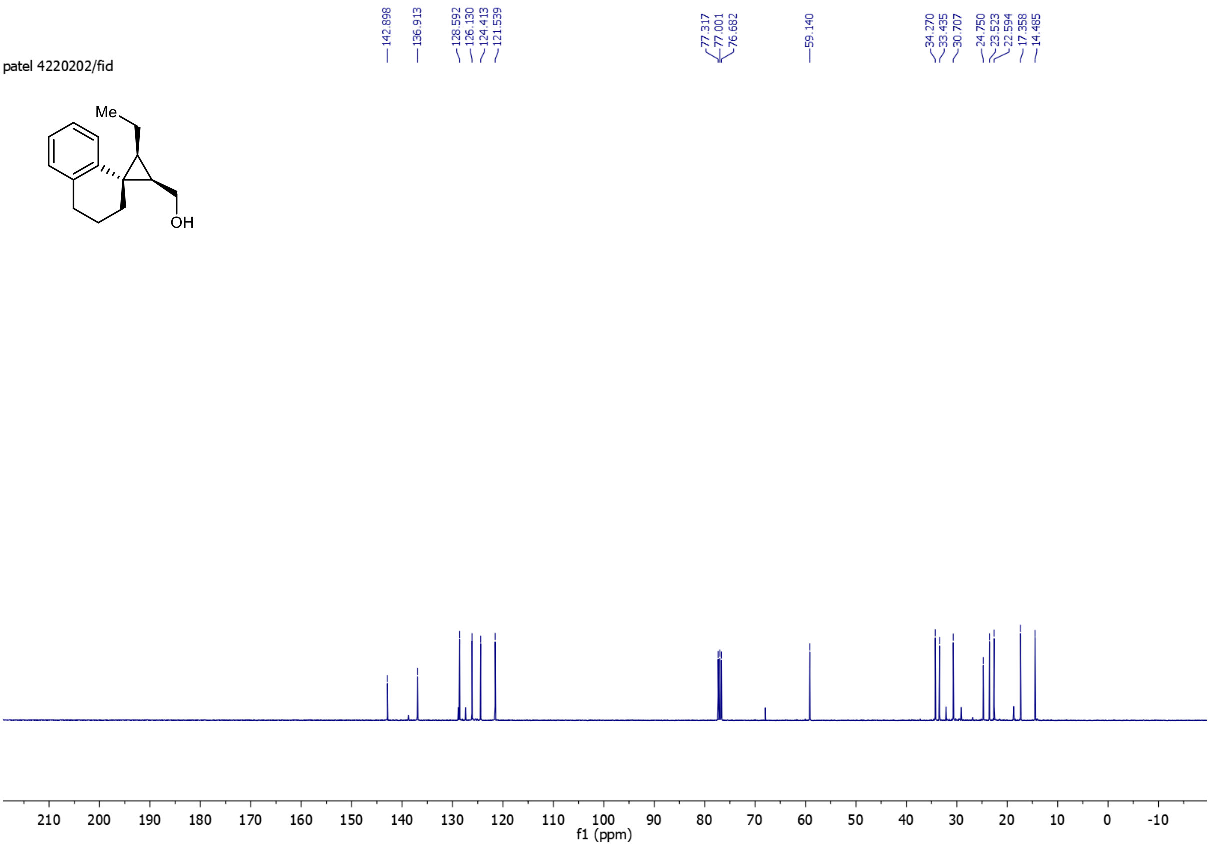
patel 4220202/fid
142.898 136.913 128.592 126.130 124.413 121.539
77.317 77.001 76.682
59.140
34.270 33.435 30.707 24.750 23.523 22.594 17.358 14.485
Me
OH
210 200 190 180 170 160 150 140 130 120 110 100 90 80 70 60 50 40 30 20 10 0 -10
f1 (ppm)

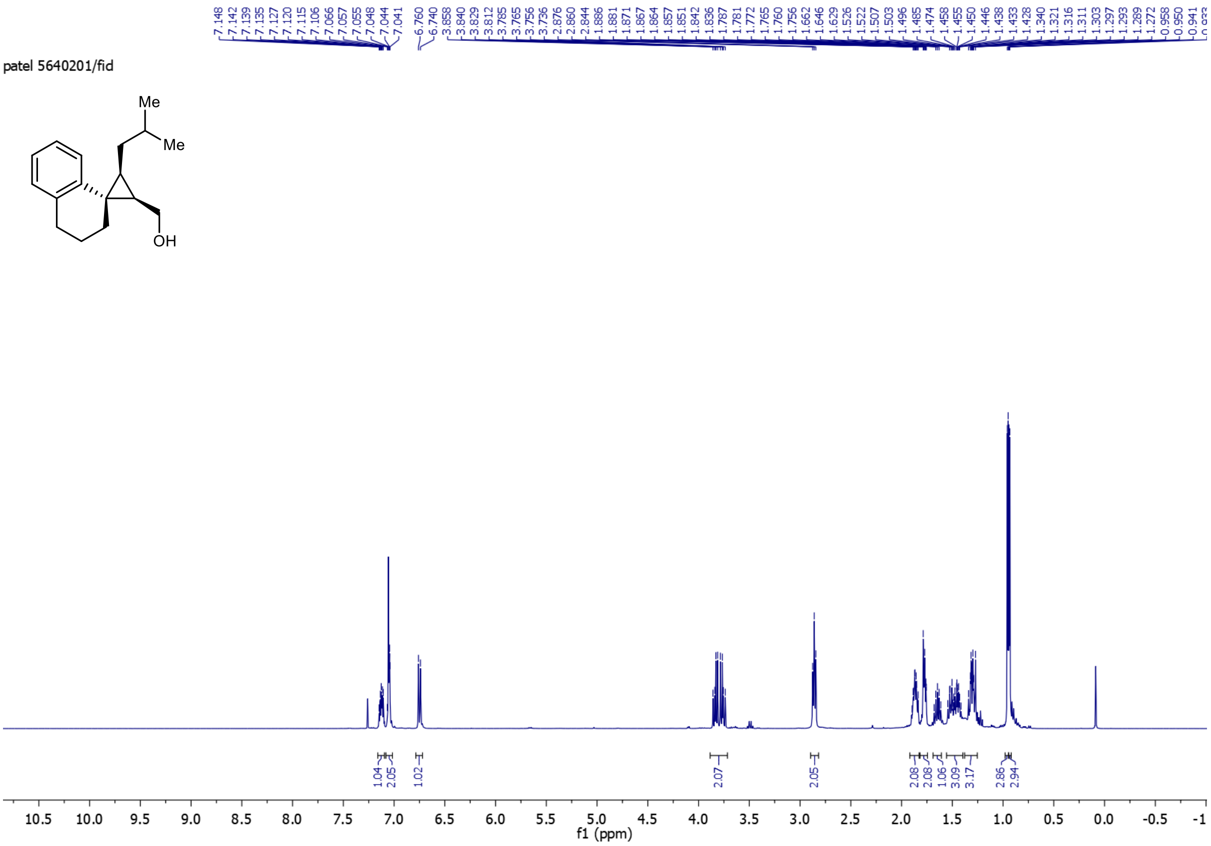
7.148
7.142
7.139
7.135
7.127
7.120
7.115
7.106
7.066
7.057
7.055
7.048
7.044
7.041
6.760
6.740
3.858
3.840
3.829
3.812
3.785
3.765
3.756
3.736
2.876
2.860
2.844
1.886
1.881
1.871
1.867
1.864
1.857
1.851
1.842
1.836
1.787
1.781
1.772
1.765
1.760
1.756
1.662
1.646
1.629
1.526
1.522
1.507
1.503
1.496
1.485
1.474
1.458
1.455
1.450
1.446
1.438
1.433
1.428
1.340
1.321
1.316
1.311
1.303
1.297
1.293
1.289
1.272
0.958
0.950
0.941
patel 5640201/fid
Me
Me
OH
1.04
2.05
1.02
2.07
2.05
2.08
2.08
1.06
3.09
3.17
2.86
2.94
10.5 10.0 9.5 9.0 8.5 8.0 7.5 7.0 6.5 6.0 5.5 5.0 4.5 4.0 3.5 3.0 2.5 2.0 1.5 1.0 0.5 0.0 -0.5 -1
f1 (ppm)

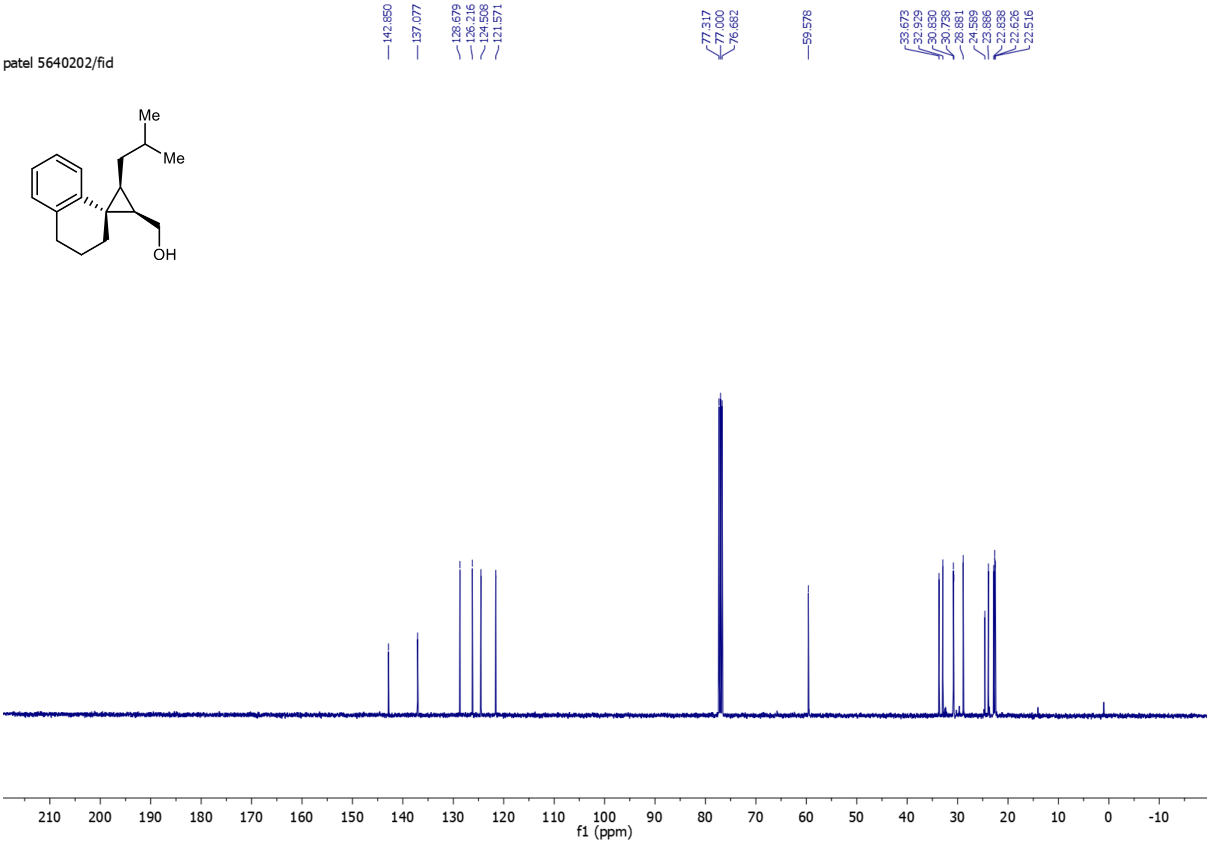
142.850
137.077
128.679
126.216
124.508
121.571
77.317
77.000
76.682
59.578
33.673
32.929
30.830
30.738
28.881
24.589
23.886
22.838
22.626
22.516
patel 5640202/fid
Me
Me
OH
210 200 190 180 170 160 150 140 130 120 110 100 90 80 70 60 50 40 30 20 10 0 -10
f1 (ppm)

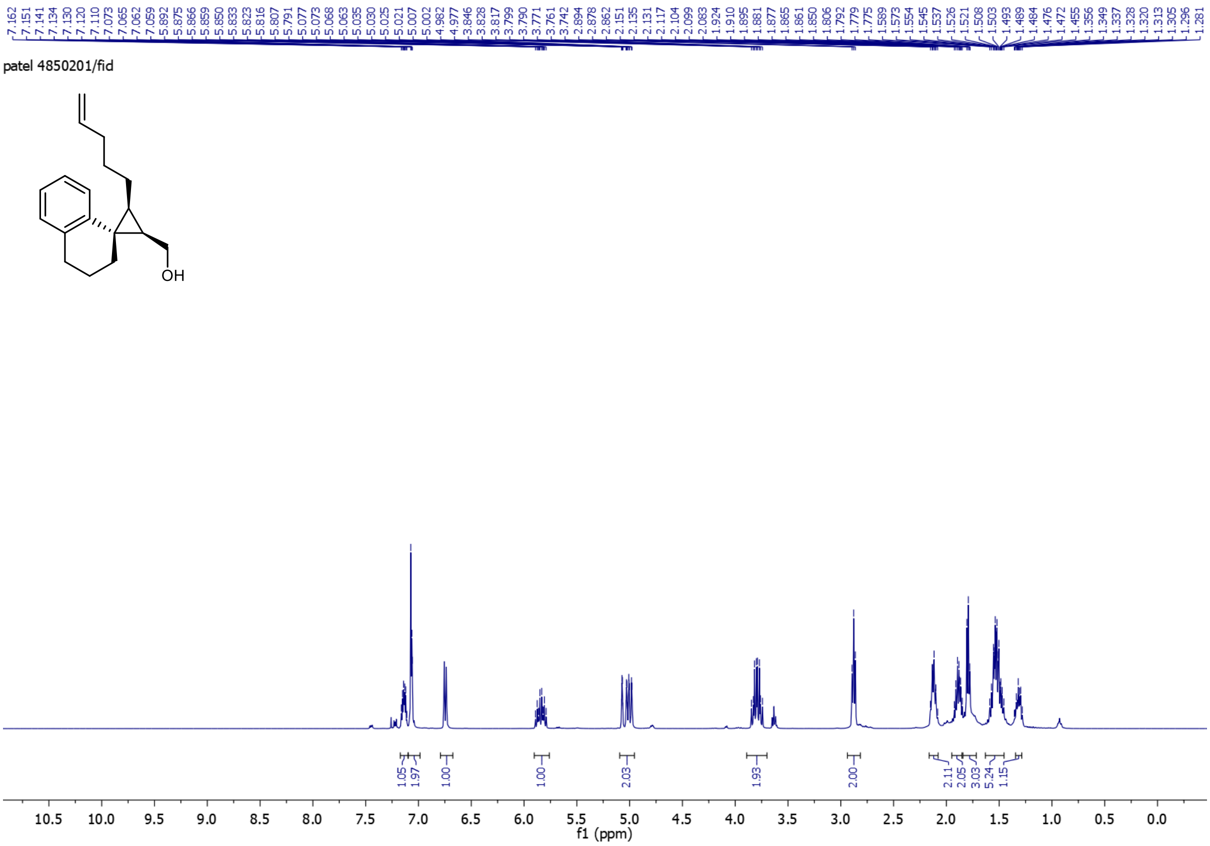

7.162 7.151 7.141 7.134 7.130 7.120 7.110 7.073 7.065 7.062 7.059 5.892 5.875 5.866 5.859 5.850 5.833 5.823 5.816 5.807 5.791 5.077 5.073 5.068 5.063 5.035 5.030 5.025 5.021 5.007 5.002 4.982 4.977 3.846 3.828 3.817 3.799 3.790 3.771 3.761 3.742 2.894 2.878 2.862 2.151 2.135 2.131 2.117 2.104 2.099 2.083 1.924 1.910 1.895 1.881 1.877 1.865 1.861 1.850 1.806 1.792 1.779 1.775 1.589 1.573 1.554 1.545 1.537 1.526 1.521 1.508 1.503 1.493 1.489 1.484 1.476 1.472 1.455 1.356 1.349 1.337 1.328 1.320 1.313 1.305 1.296 1.281
patel 4850201/fid
OH
1.05 1.97 1.00 1.00 2.03 1.93 2.00 2.11 2.05 3.03 5.24 1.15
10.5 10.0 9.5 9.0 8.5 8.0 7.5 7.0 6.5 6.0 5.5 5.0 4.5 4.0 3.5 3.0 2.5 2.0 1.5 1.0 0.5 0.0
f1 (ppm)

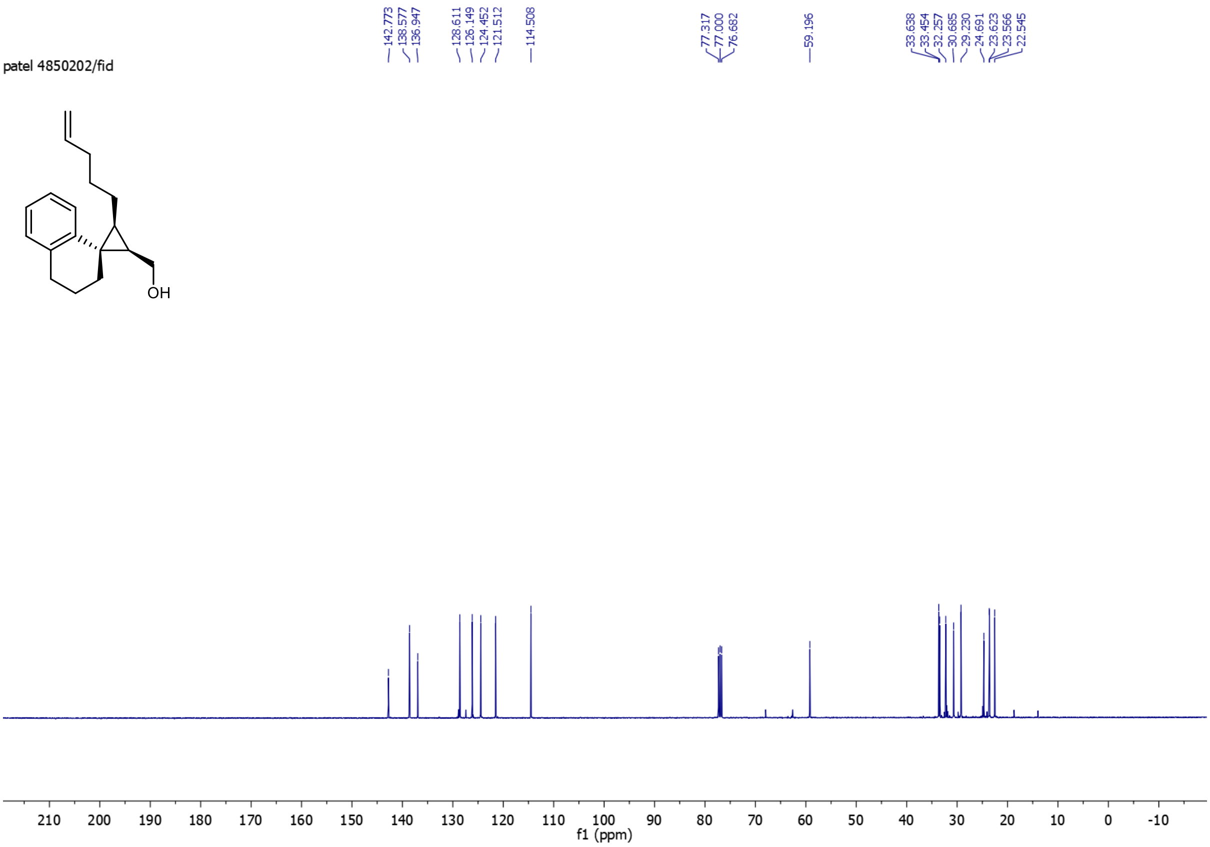

142.773 138.577 136.947 128.611 126.149 124.452 121.512 114.508 77.317 77.000 76.682 59.196 33.638 33.454 32.257 30.685 29.230 24.691 23.623 23.566 22.545
patel 4850202/fid
OH
210 200 190 180 170 160 150 140 130 120 110 100 90 80 70 60 50 40 30 20 10 0 -10
f1 (ppm)

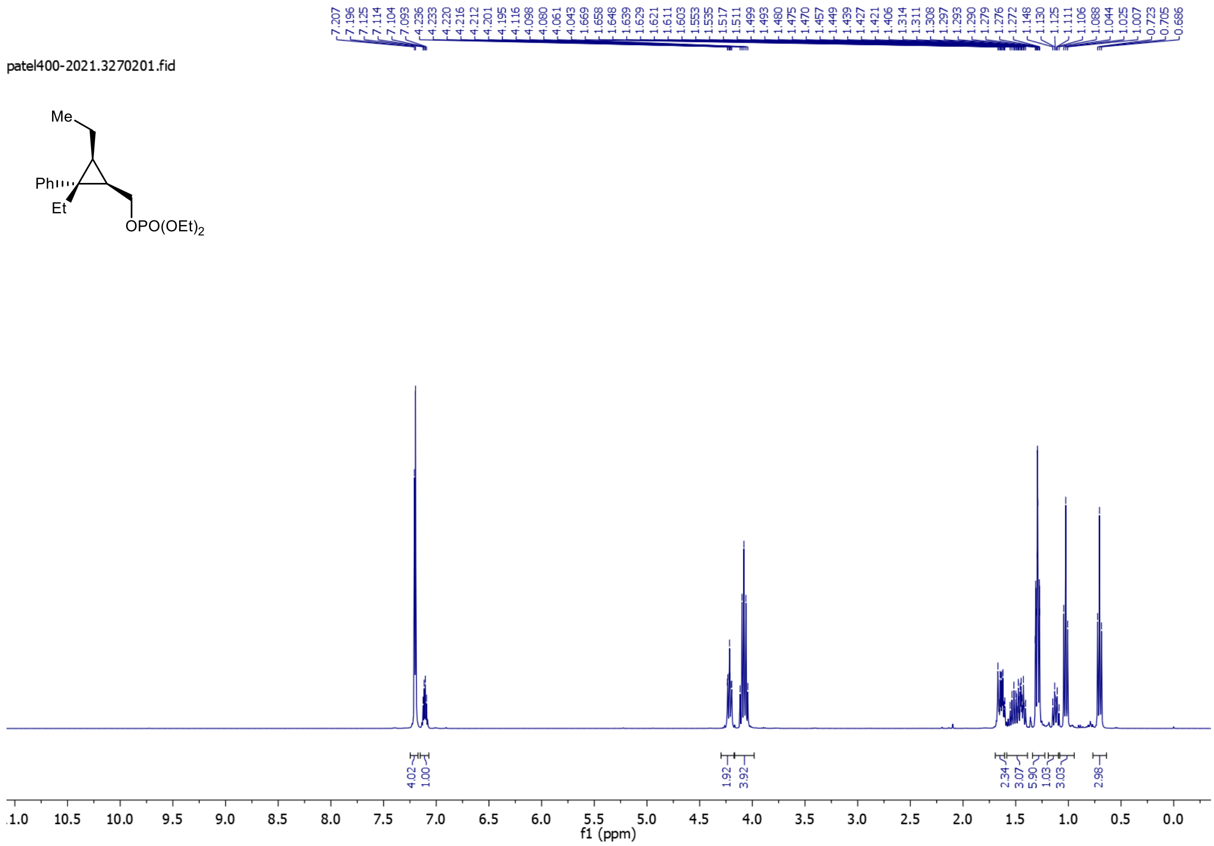
patel400-2021.3270201.fid
Me
Ph
Et
OPO(OEt)2
7.207 7.196 7.125 7.114 7.104 7.093 4.236 4.233 4.220 4.216 4.212 4.201 4.195 4.116 4.098 4.080 4.061 4.043 1.669 1.658 1.648 1.639 1.629 1.621 1.611 1.603 1.553 1.535 1.517 1.511 1.499 1.493 1.480 1.475 1.470 1.457 1.449 1.439 1.427 1.421 1.406 1.314 1.311 1.308 1.297 1.293 1.290 1.279 1.276 1.272 1.148 1.130 1.125 1.111 1.106 1.088 1.044 1.025 1.007 0.723 0.705 0.686
4.02 1.00 1.92 3.92 2.34 3.07 5.90 1.03 3.03 2.98
f1 (ppm)
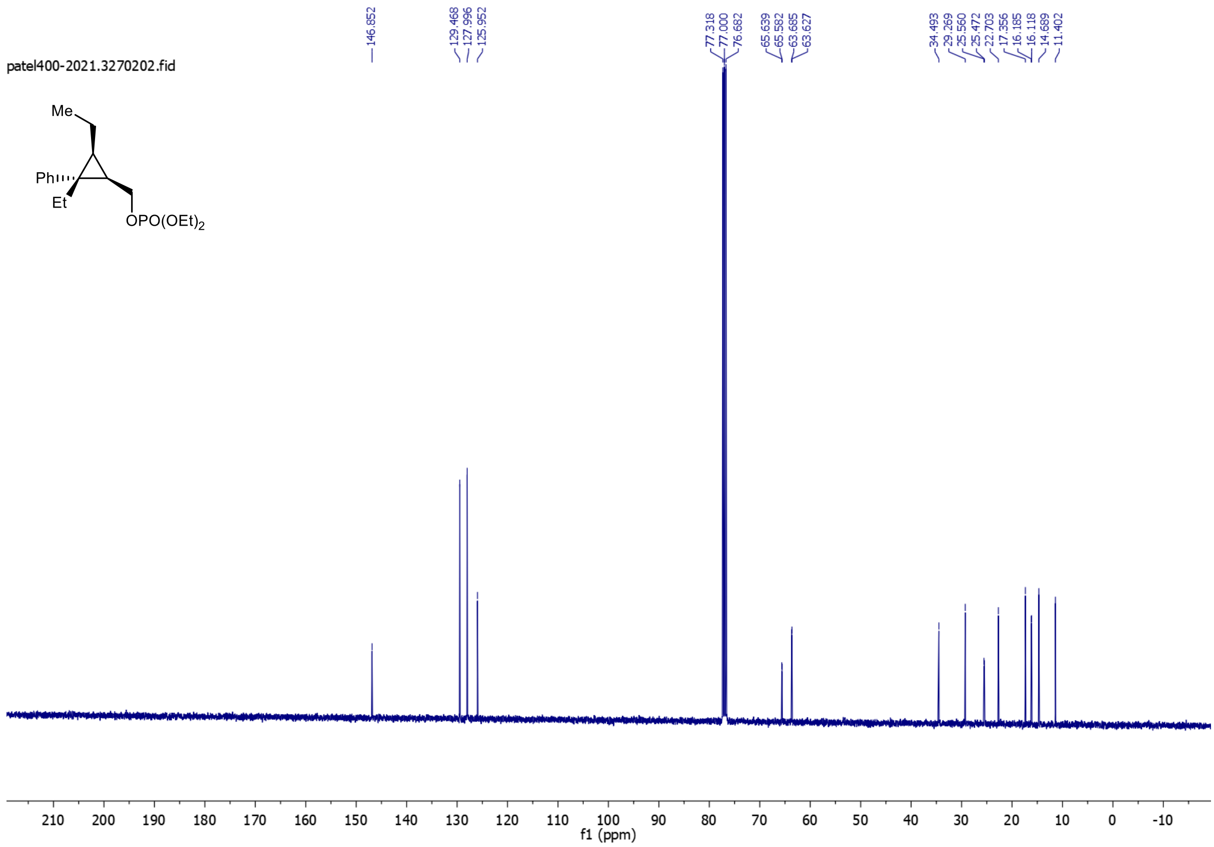
patel400-2021.3270202.fid
Me
Ph
Et
OPO(OEt)2
146.852 129.468 127.996 125.952 77.318 77.000 76.682 65.639 65.582 63.685 63.627 34.493 29.269 25.560 25.472 22.703 17.356 16.185 16.118 14.689 11.402
f1 (ppm)

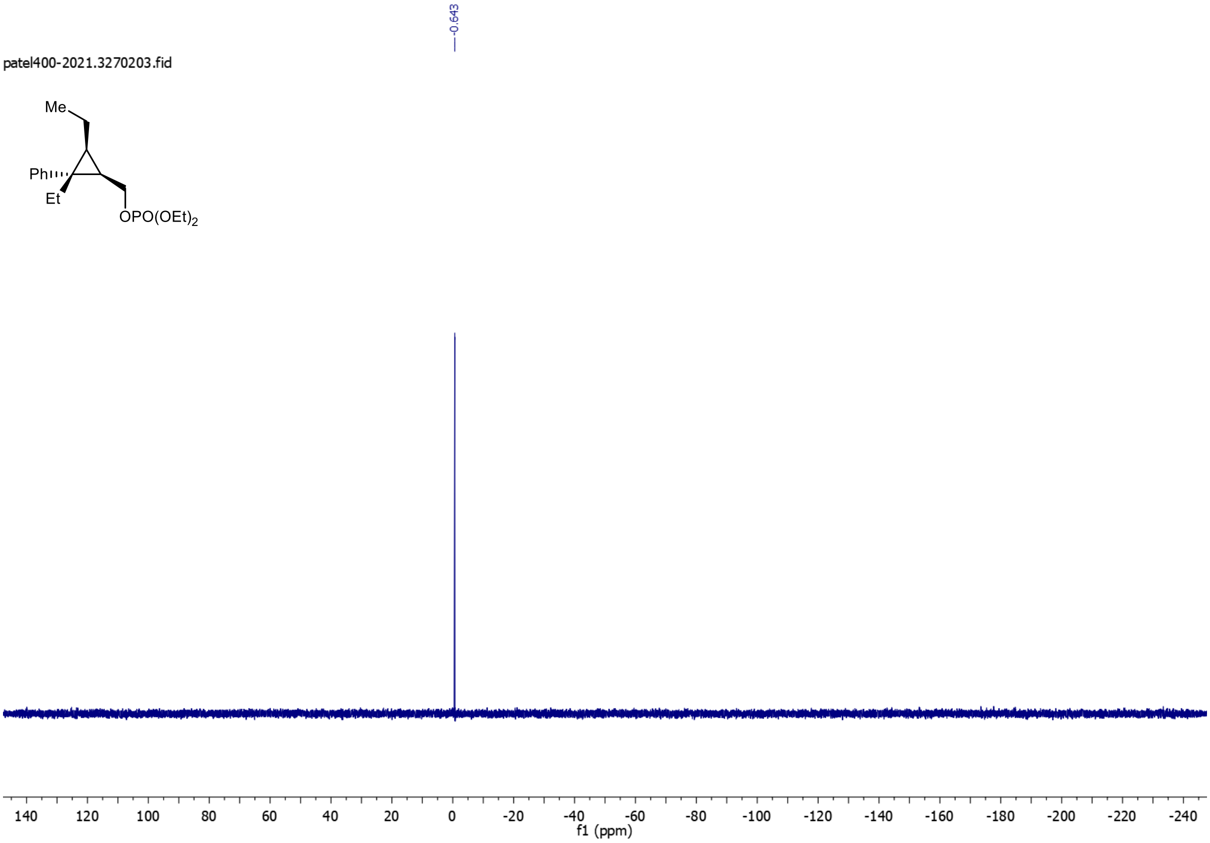

patel400-2021.3270203.fid
Me
Ph
Et
OPO(OEt)2
-0.643
f1 (ppm)

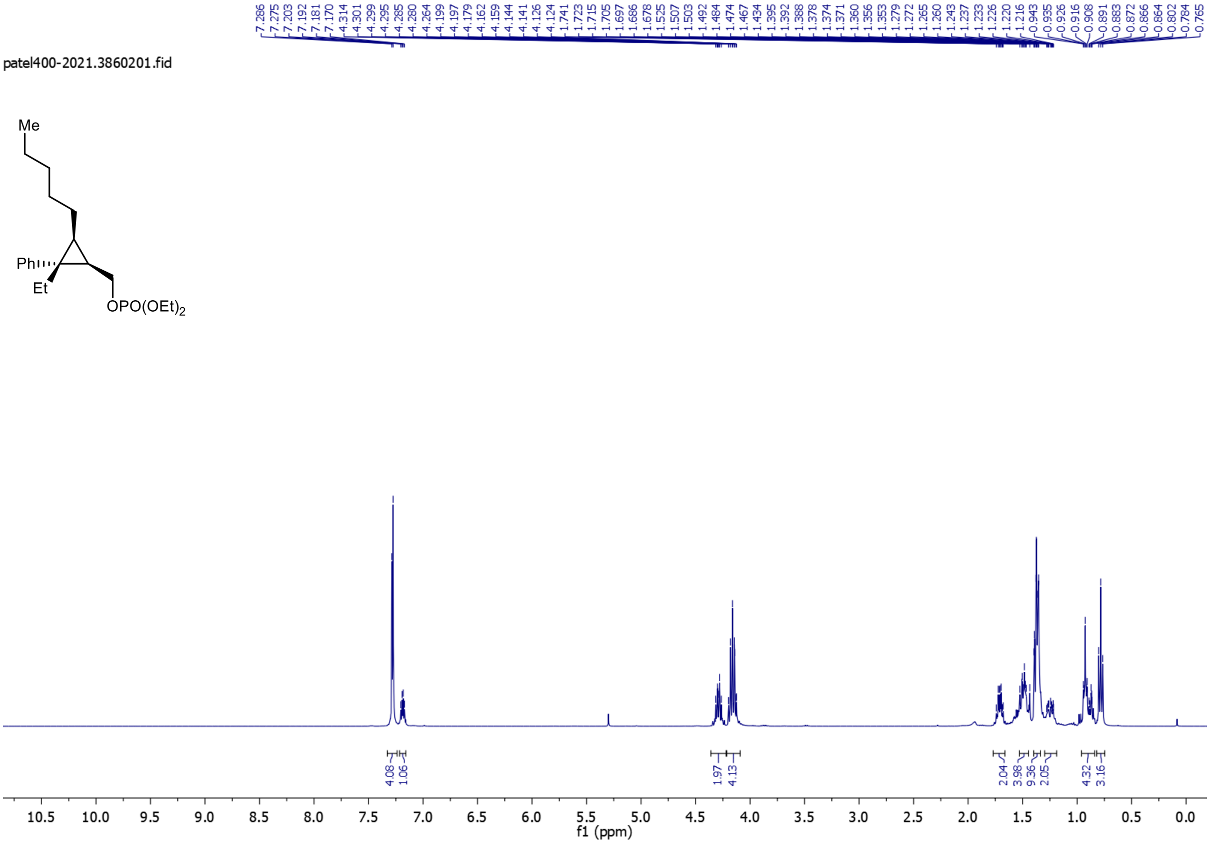

patel400-2021.3860201.fid
Me
Ph
Et
OPO(OEt)2
7.286 7.275 7.203 7.192 7.181 7.170 4.314 4.301 4.299 4.295 4.285 4.280 4.264 4.199 4.197 4.179 4.162 4.159 4.144 4.141 4.126 4.124 1.741 1.723 1.715 1.705 1.697 1.686 1.678 1.525 1.507 1.503 1.492 1.484 1.474 1.467 1.434 1.395 1.392 1.388 1.378 1.374 1.371 1.360 1.356 1.353 1.279 1.272 1.265 1.260 1.243 1.237 1.233 1.226 1.220 1.216 0.943 0.935 0.926 0.916 0.908 0.891 0.883 0.872 0.866 0.864 0.802 0.784 0.765
4.08 1.06 1.97 4.13 2.04 3.98 9.36 2.05 4.32 3.16
f1 (ppm)

patel400-2021.3860202.fid

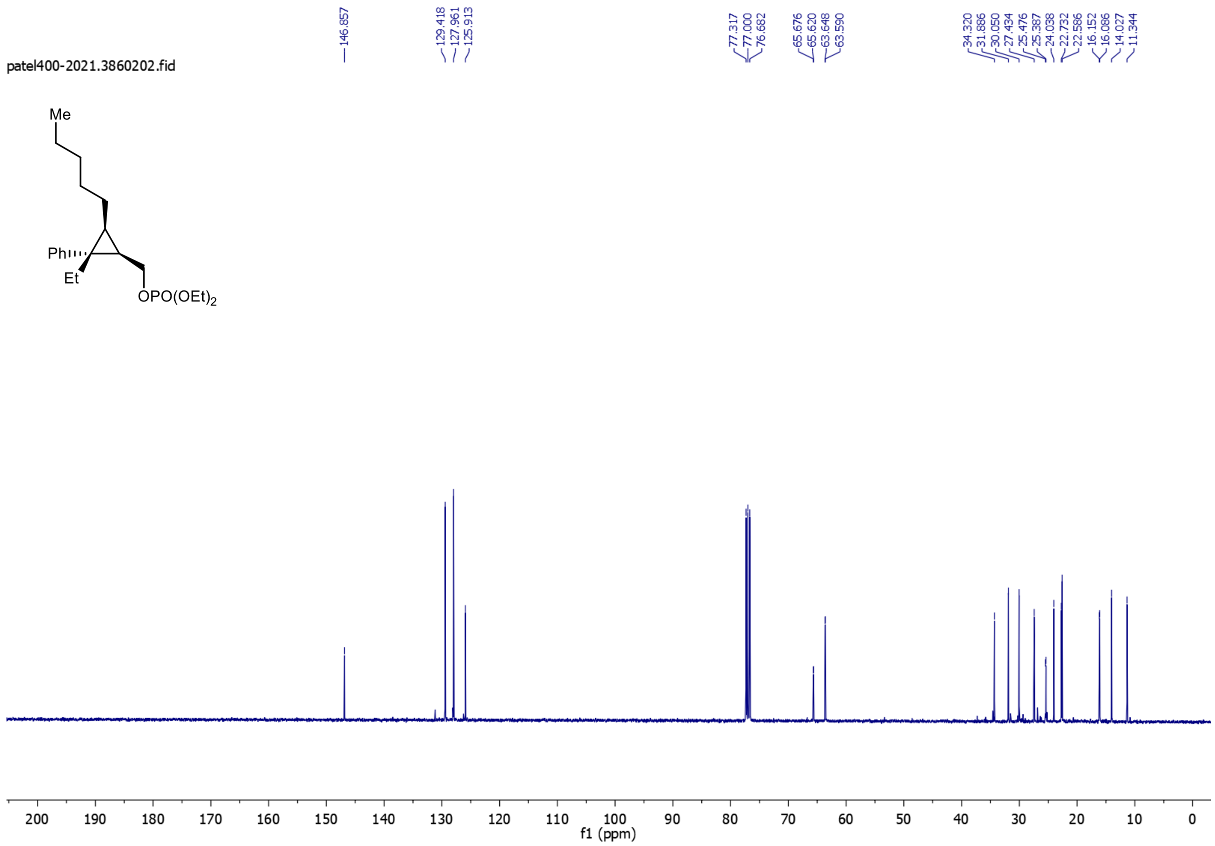

patel400-2021.3860203.fid

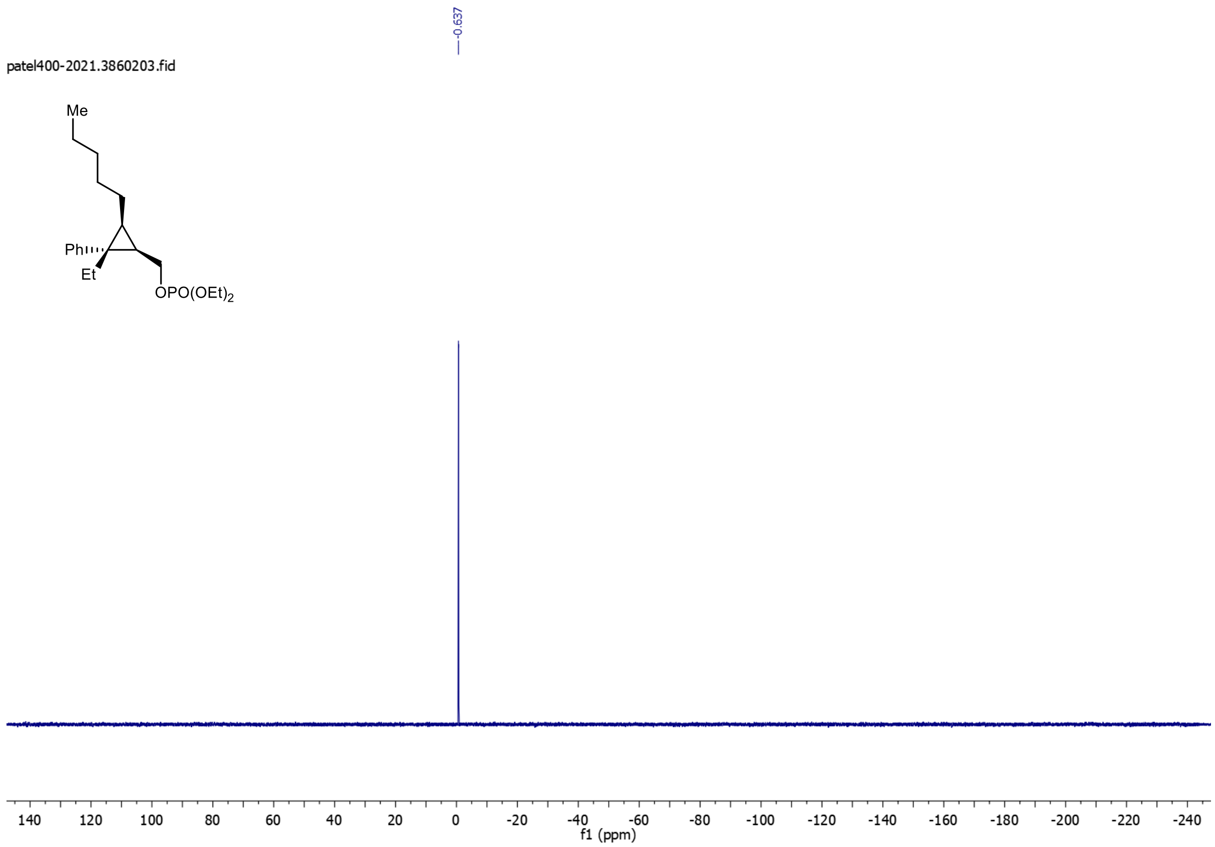

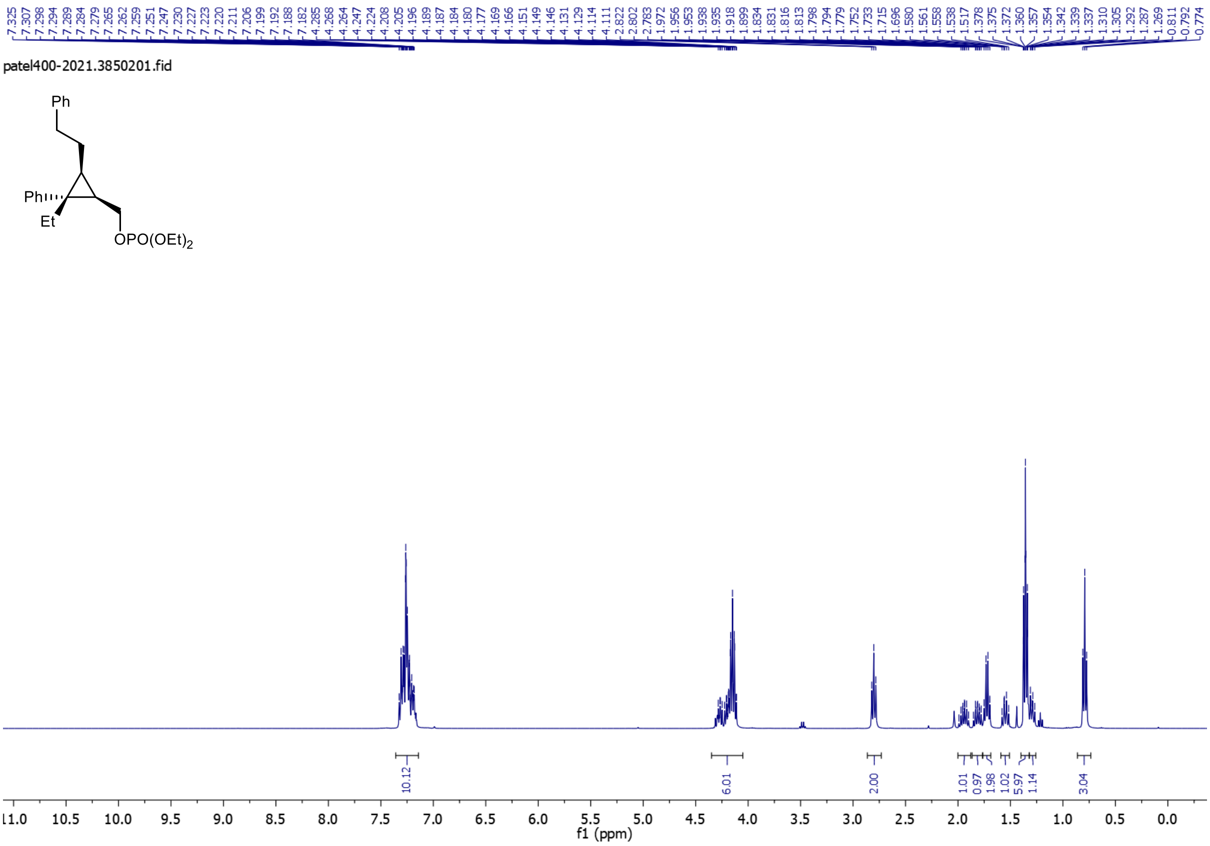
7.325 7.307 7.298 7.294 7.289 7.284 7.279 7.265 7.262 7.259 7.251 7.247 7.230 7.227 7.223 7.220 7.211 7.206 7.199 7.192 7.188 7.182 4.285 4.268 4.264 4.247 4.224 4.208 4.205 4.196 4.189 4.187 4.184 4.180 4.177 4.169 4.166 4.151 4.149 4.146 4.131 4.129 4.114 4.111 2.822 2.802 2.783 1.972 1.956 1.953 1.938 1.935 1.918 1.899 1.834 1.831 1.816 1.813 1.798 1.794 1.779 1.752 1.733 1.715 1.696 1.580 1.561 1.558 1.538 1.517 1.378 1.375 1.372 1.360 1.357 1.354 1.342 1.339 1.337 1.310 1.305 1.292 1.287 1.269 0.811 0.792 0.774
patel400-2021.3850201.fid
Ph
Ph
Et
OPO(OEt)2
10.12
6.01
2.00
1.01
0.97
1.98
1.02
5.97
1.14
3.04
f1 (ppm)

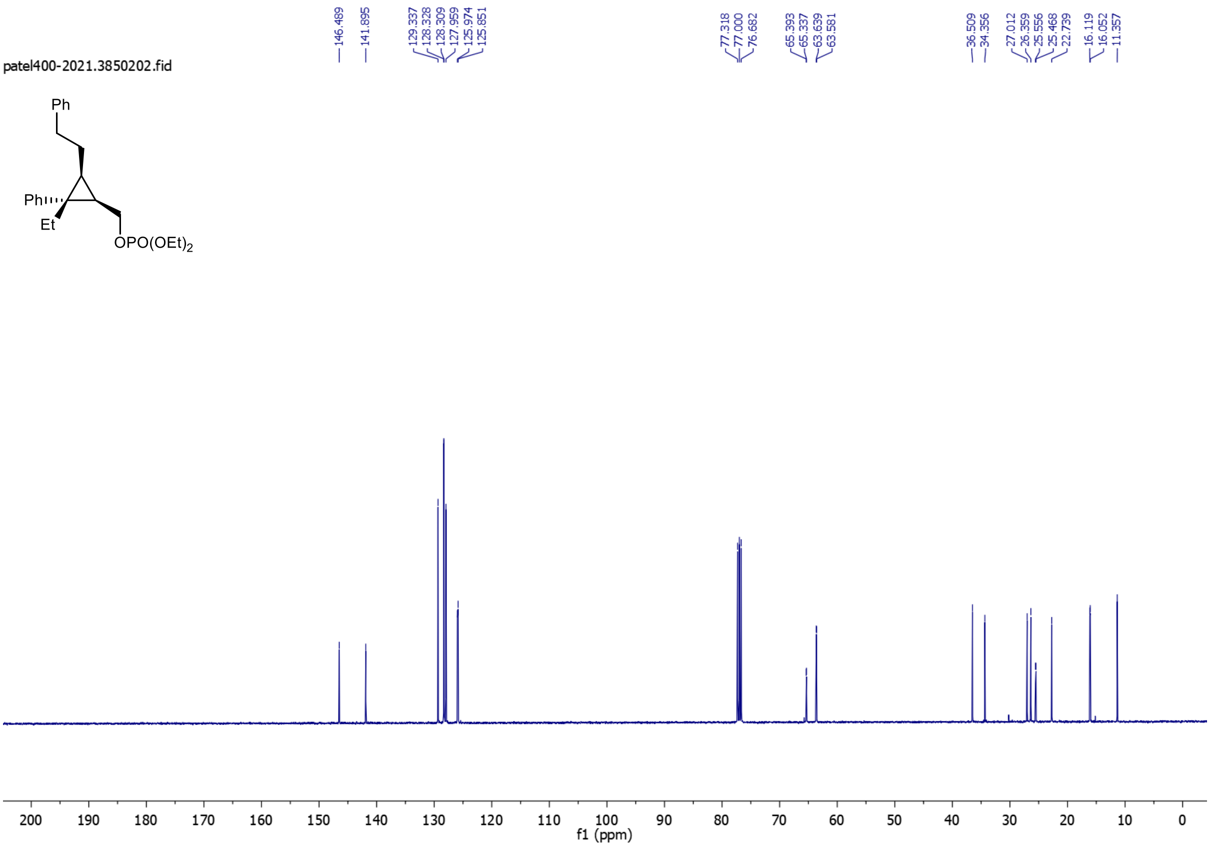
146.489
141.895
129.337
128.328
128.309
127.959
125.974
125.851
77.318
77.000
76.682
65.393
65.337
63.639
63.581
36.509
34.356
27.012
26.359
25.556
25.468
22.739
16.119
16.052
11.357
patel400-2021.3850202.fid
Ph
Ph
Et
OPO(OEt)2
f1 (ppm)

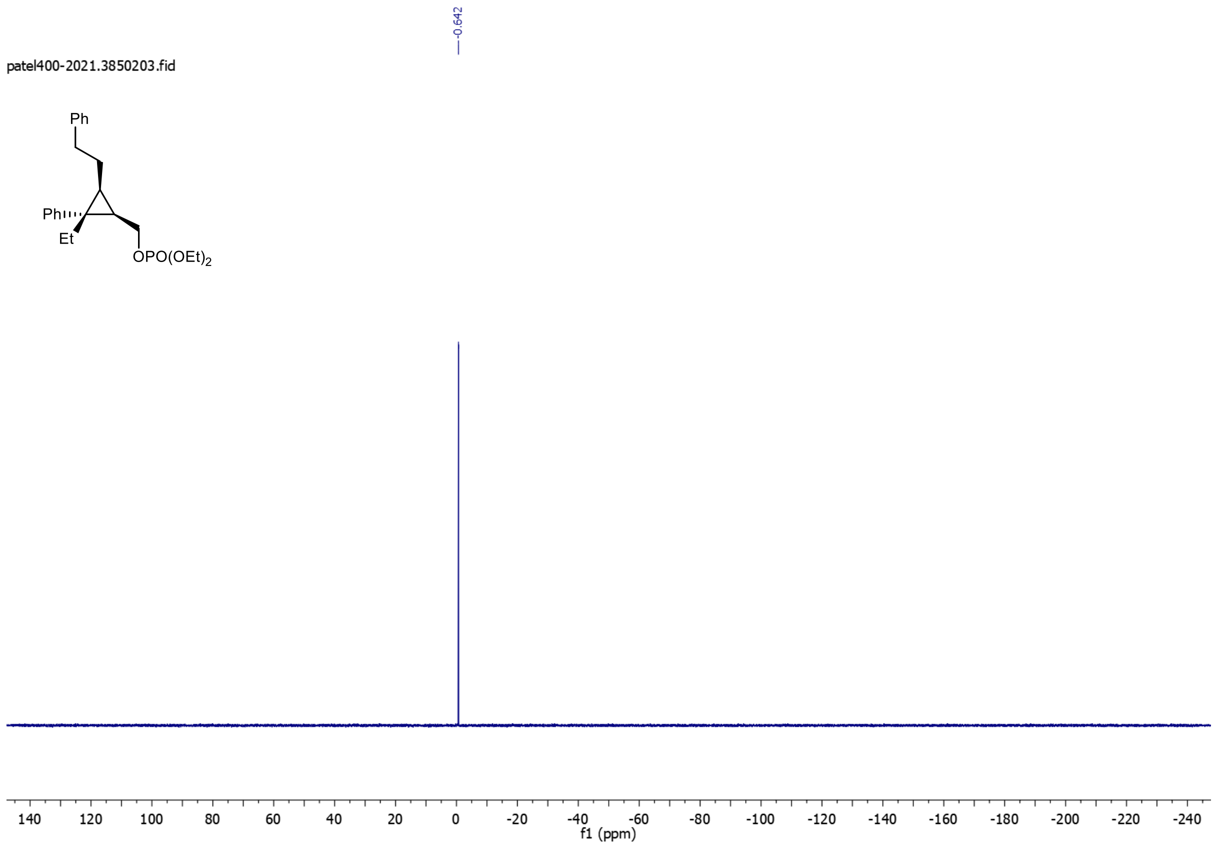
patel400-2021.3850203.fid
Ph
Ph
Et
OPO(OEt)2
-0.642
f1 (ppm)

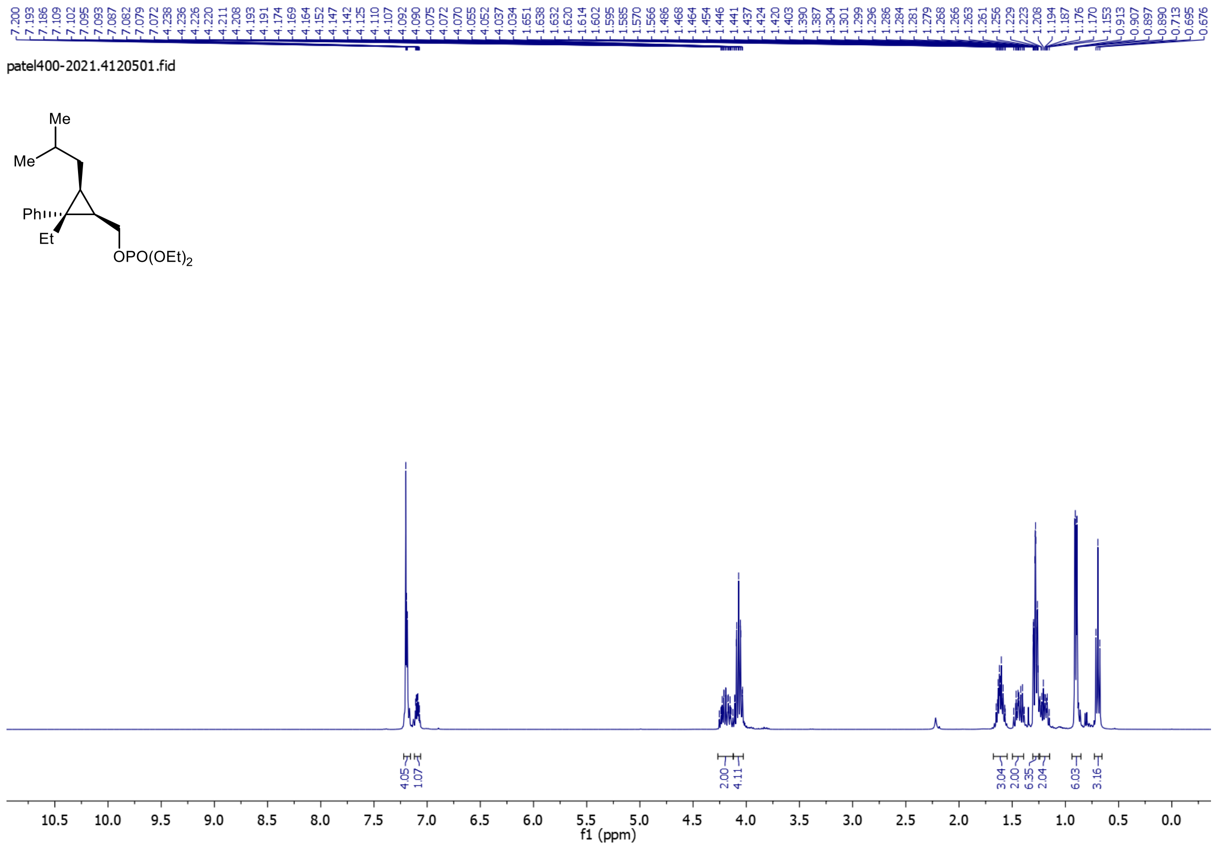
patel400-2021.4120501.fid
Me
Me
Ph
Et
OPO(OEt)2
4.05
1.07
2.00
4.11
3.04
2.00
6.35
2.04
6.03
3.16
f1 (ppm)

patel400-2021.4120502.fid

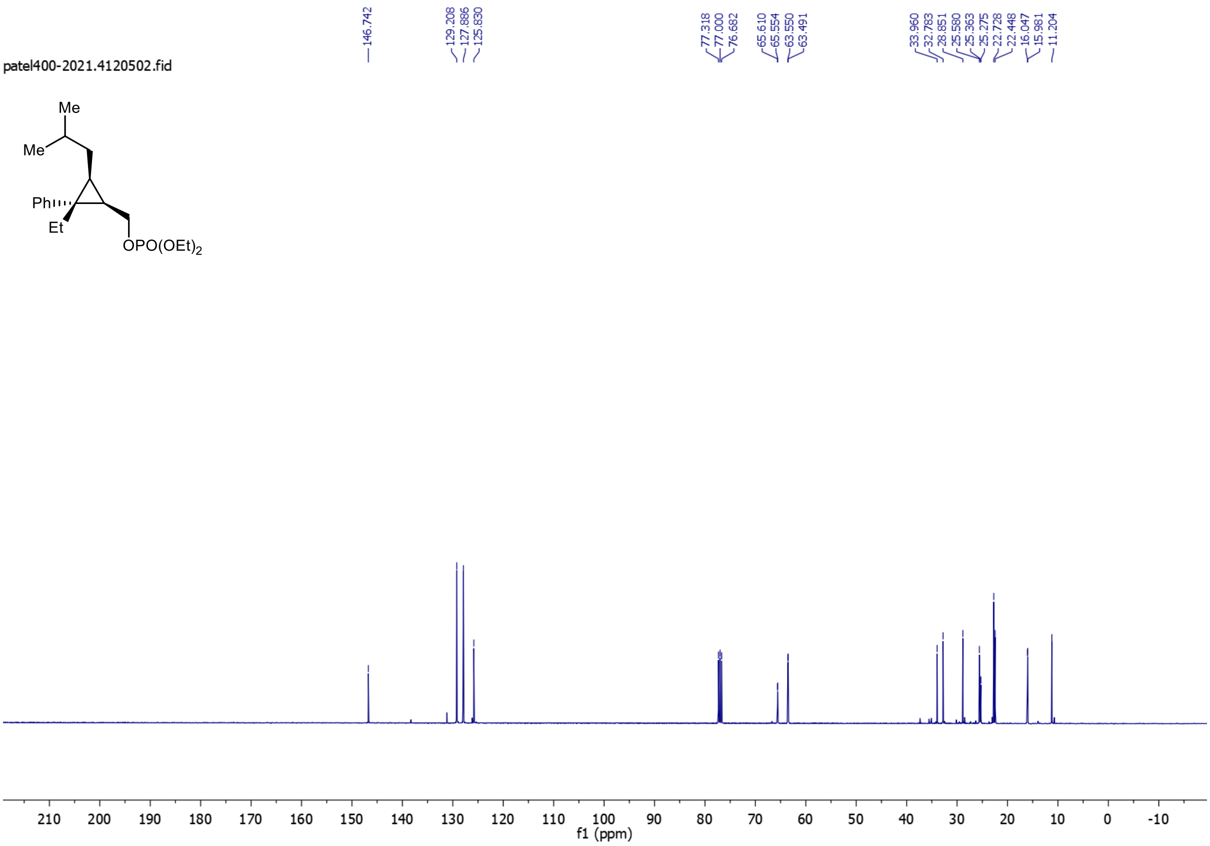

patel400-2021.4120503.fid

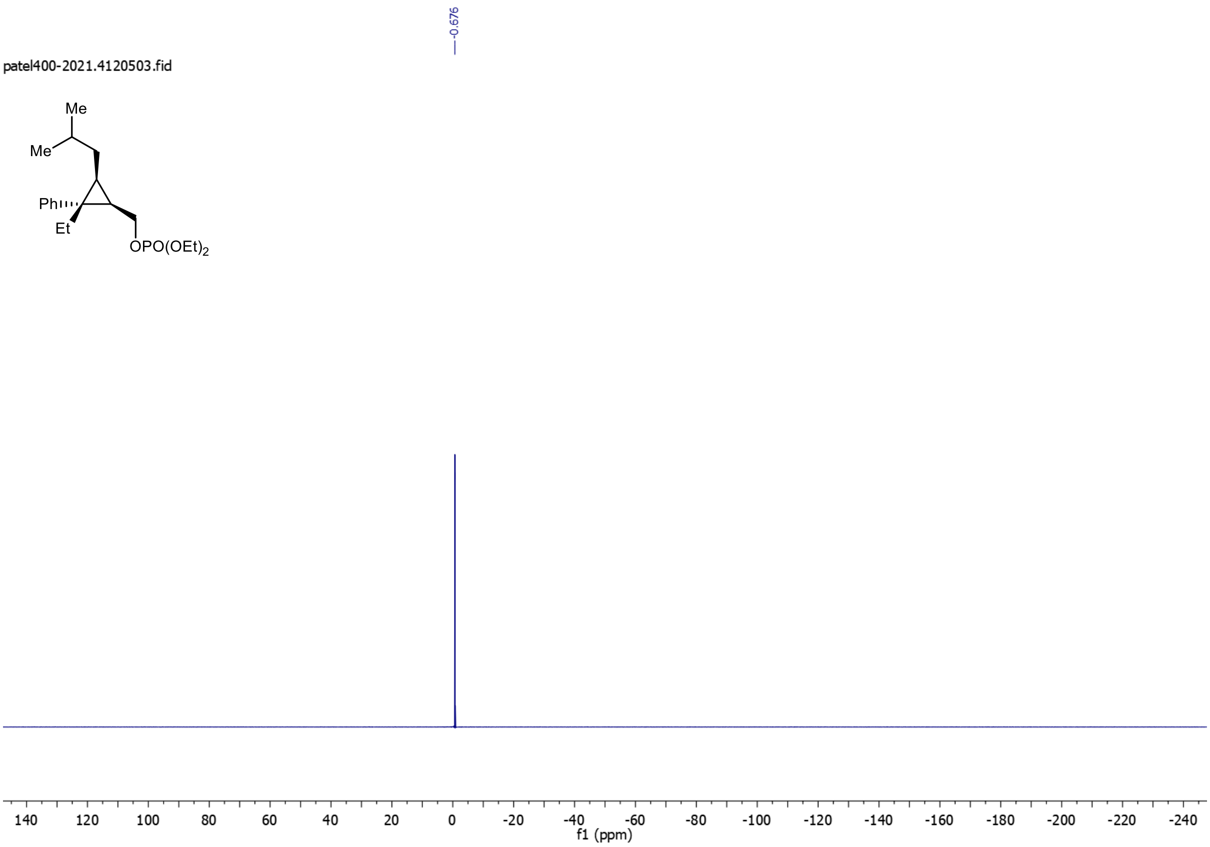

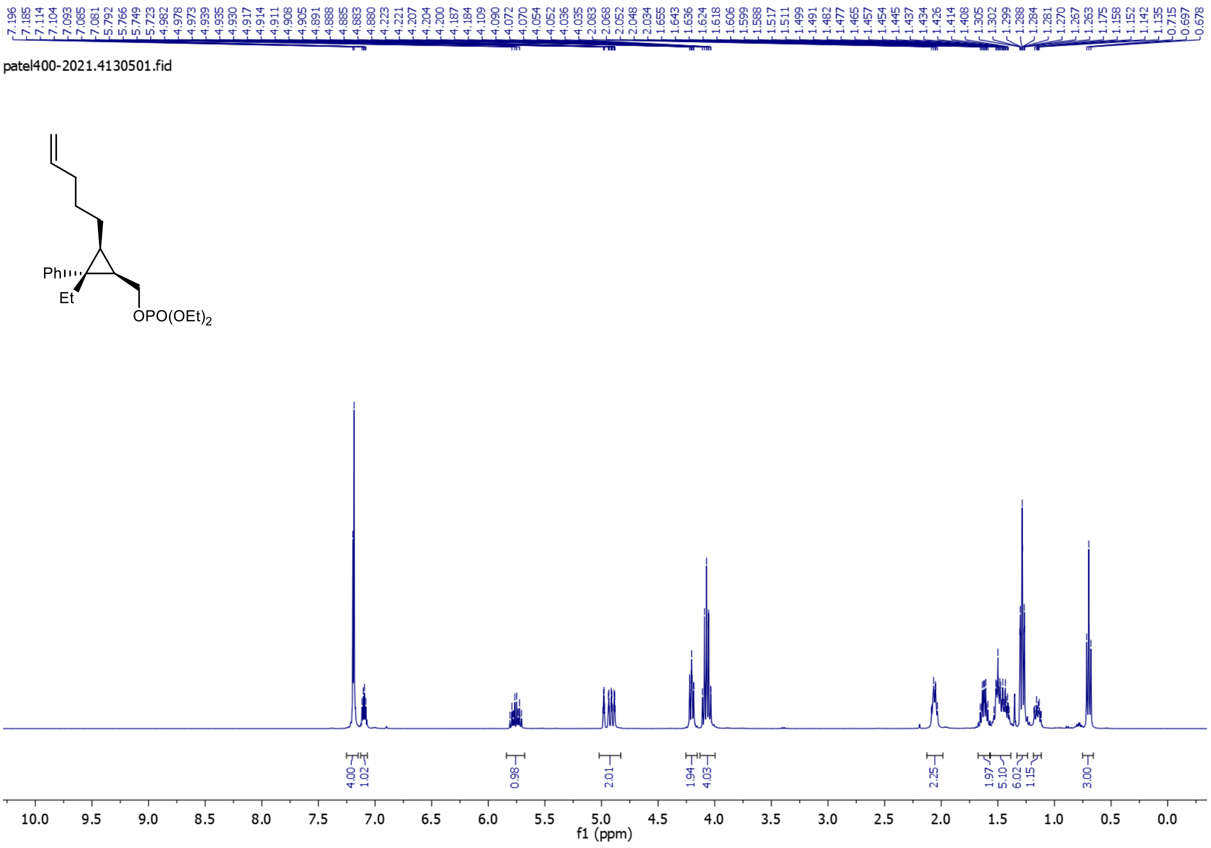
7.196 7.185 7.114 7.104 7.093 7.085 7.081 5.792 5.766 5.749 5.723 4.982 4.978 4.973 4.939 4.935 4.930 4.917 4.914 4.911 4.908 4.905 4.891 4.888 4.885 4.883 4.880 4.223 4.221 4.207 4.204 4.200 4.187 4.184 4.109 4.090 4.072 4.070 4.054 4.052 4.036 4.035 2.068 2.063 2.052 2.048 2.034 1.655 1.643 1.636 1.624 1.618 1.606 1.599 1.588 1.517 1.511 1.499 1.491 1.482 1.477 1.465 1.457 1.454 1.445 1.437 1.434 1.426 1.414 1.408 1.305 1.302 1.299 1.288 1.284 1.281 1.270 1.267 1.263 1.175 1.158 1.152 1.142 1.135 0.715 0.697 0.678
patel400-2021.4130501.fid
Ph
Et
OPO(OEt)2
4.00 1.02 0.98 2.01 1.94 4.03 2.25 1.97 5.10 6.02 1.15 3.00
10.0 9.5 9.0 8.5 8.0 7.5 7.0 6.5 6.0 5.5 5.0 4.5 4.0 3.5 3.0 2.5 2.0 1.5 1.0 0.5 0.0
f1 (ppm)

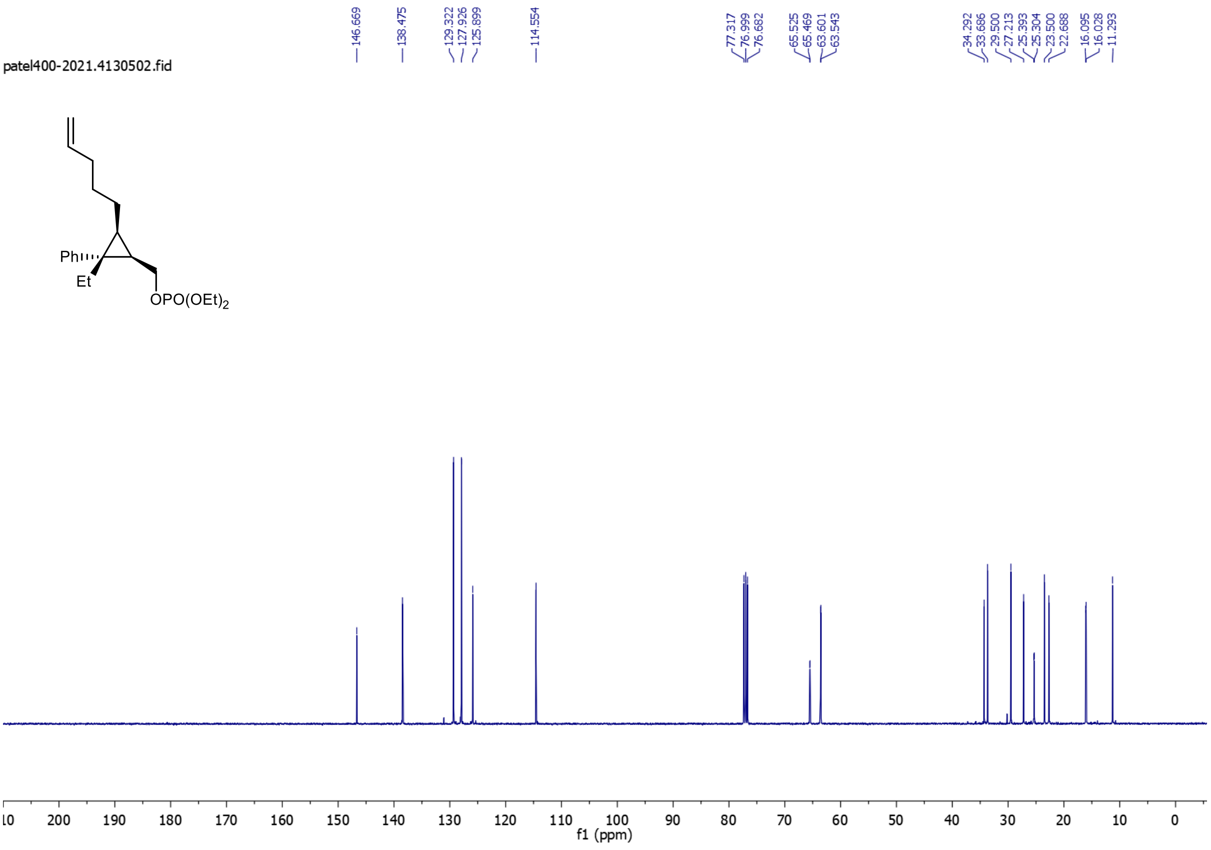
146.669 138.475 129.322 127.926 125.899 114.554 77.317 76.999 76.682 65.525 65.469 63.601 63.543 34.292 33.686 29.500 27.213 25.393 25.304 23.500 22.688 16.095 16.028 11.293
patel400-2021.4130502.fid
Ph
Et
OPO(OEt)2
200 190 180 170 160 150 140 130 120 110 100 90 80 70 60 50 40 30 20 10 0
f1 (ppm)

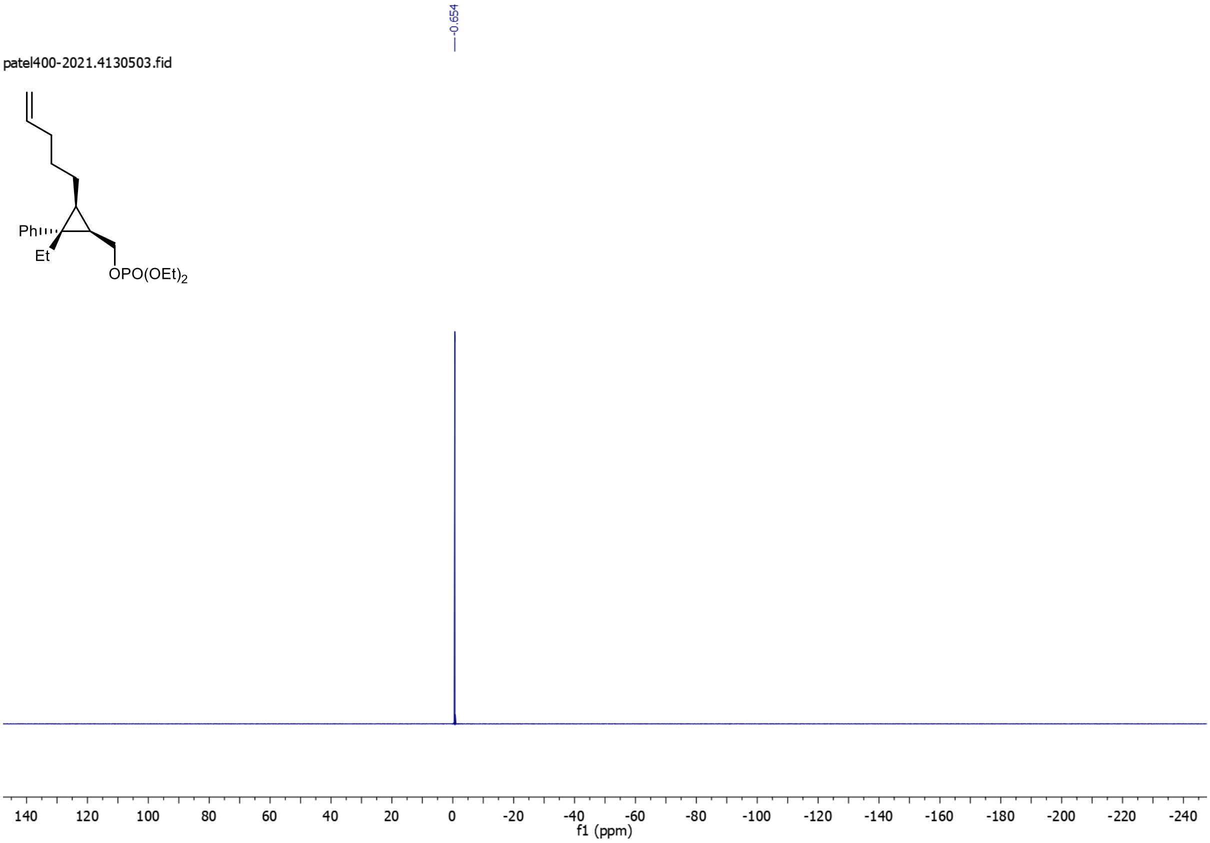

-0.654
patel400-2021.4130503.fid
Ph
Et
OPO(OEt)2
140 120 100 80 60 40 20 0 -20 -40 -60 -80 -100 -120 -140 -160 -180 -200 -220 -240
f1 (ppm)

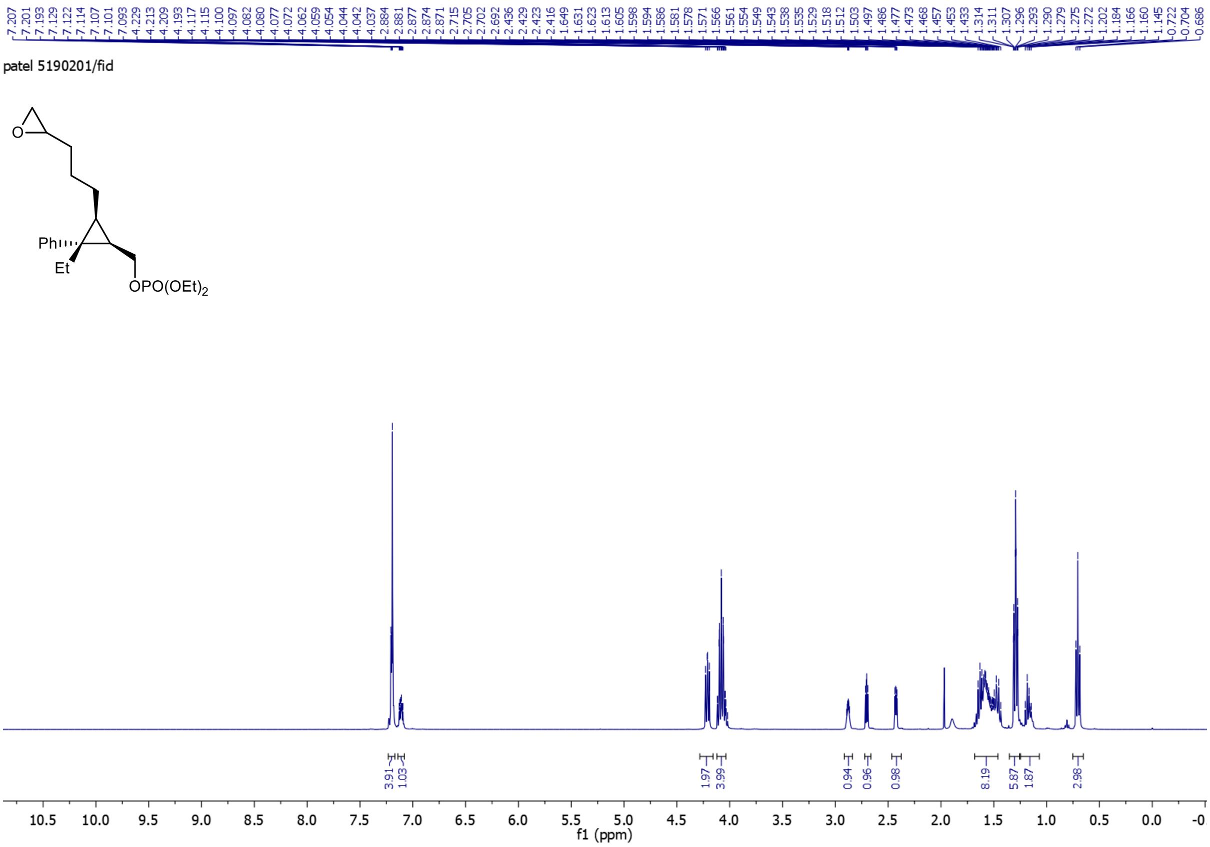

patel 5190201/fid
O
Ph
Et
OPO(OEt)2
3.91 1.03 1.97 3.99 0.94 0.96 0.98 8.19 5.87 1.87 2.98
10.5 10.0 9.5 9.0 8.5 8.0 7.5 7.0 6.5 6.0 5.5 5.0 4.5 4.0 3.5 3.0 2.5 2.0 1.5 1.0 0.5 0.0 -0
f1 (ppm)

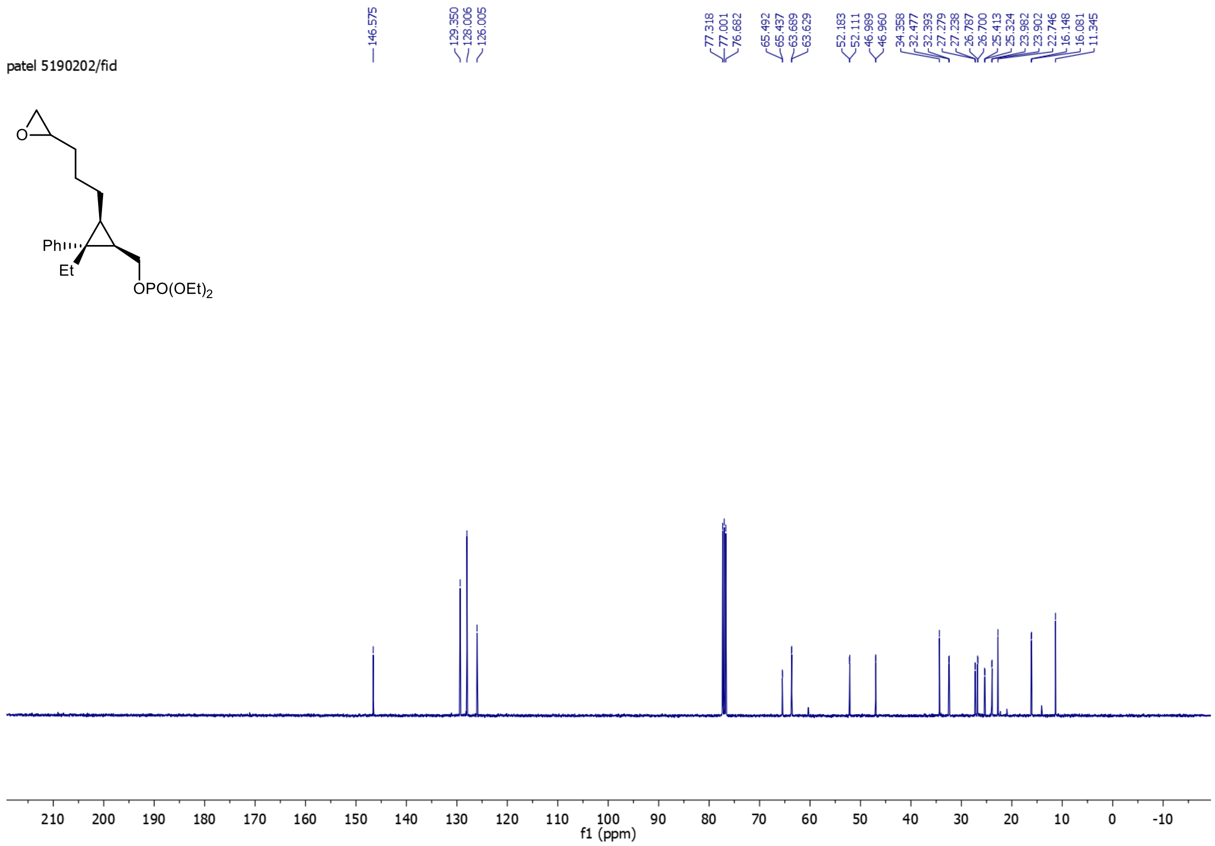
patel 5190202/fid
146.575
129.350
128.006
126.005
77.318
77.001
76.682
65.492
65.437
63.689
63.629
52.183
52.111
46.989
46.960
34.358
32.477
32.393
27.279
27.238
26.787
26.700
25.413
25.324
23.982
23.902
22.746
16.148
16.081
11.345
O
Ph
Et
OPO(OEt)2
f1 (ppm)

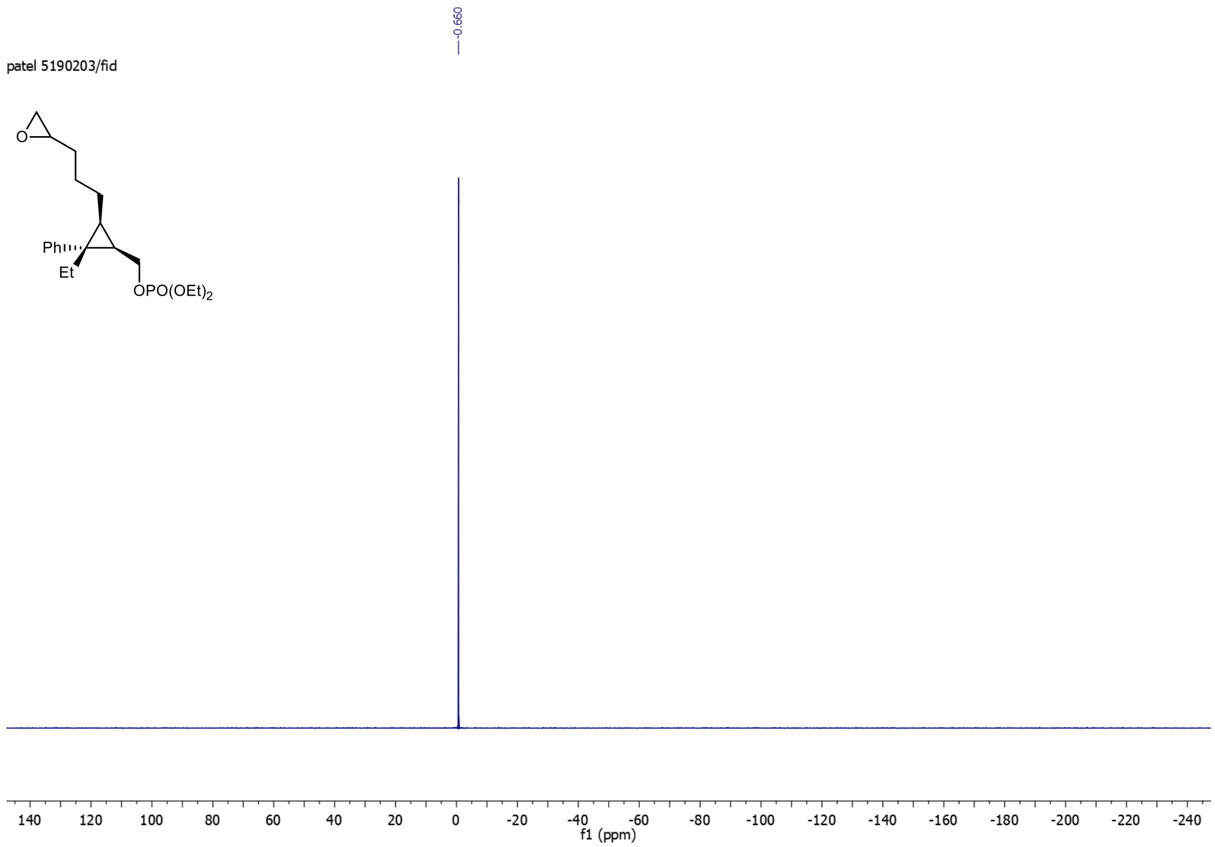
patel 5190203/fid
-0.660
O
Ph
Et
OPO(OEt)2
f1 (ppm)

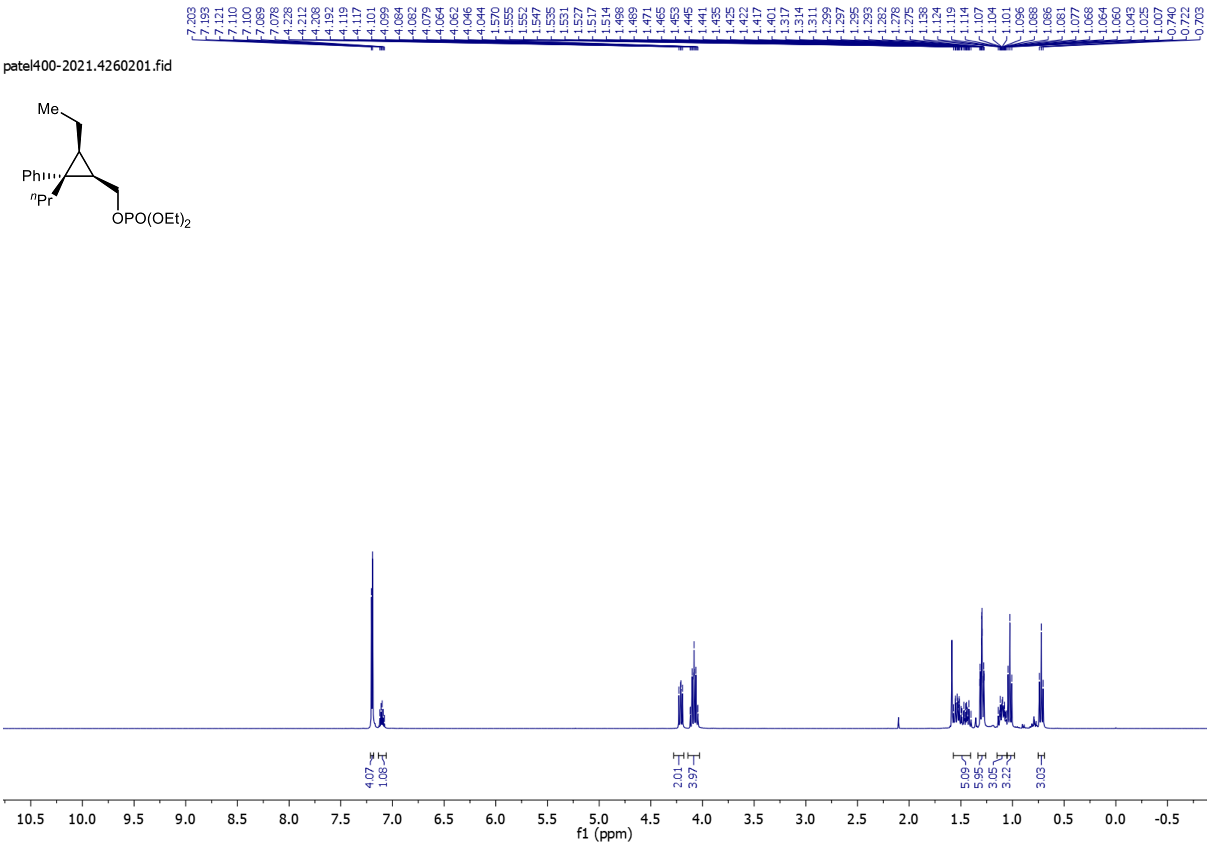
patel400-2021.4260201.fid
Me
Ph
nPr
OPO(OEt)2
f1 (ppm)

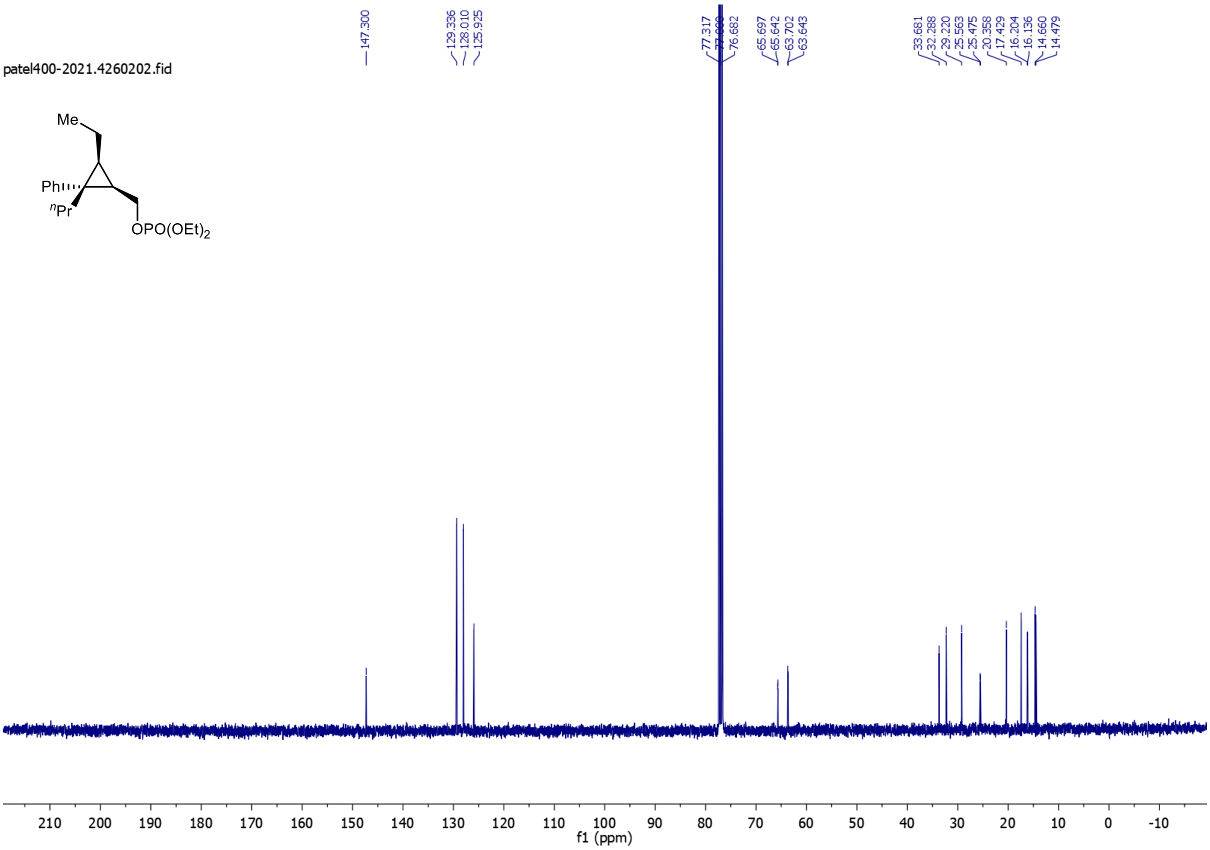
patel400-2021.4260202.fid
Me
Ph
nPr
OPO(OEt)2
147.300
129.336
128.010
125.925
77.317
76.682
65.697
65.642
63.702
63.643
33.681
32.288
29.220
25.563
25.475
20.358
17.429
16.204
16.136
14.660
14.479
f1 (ppm)

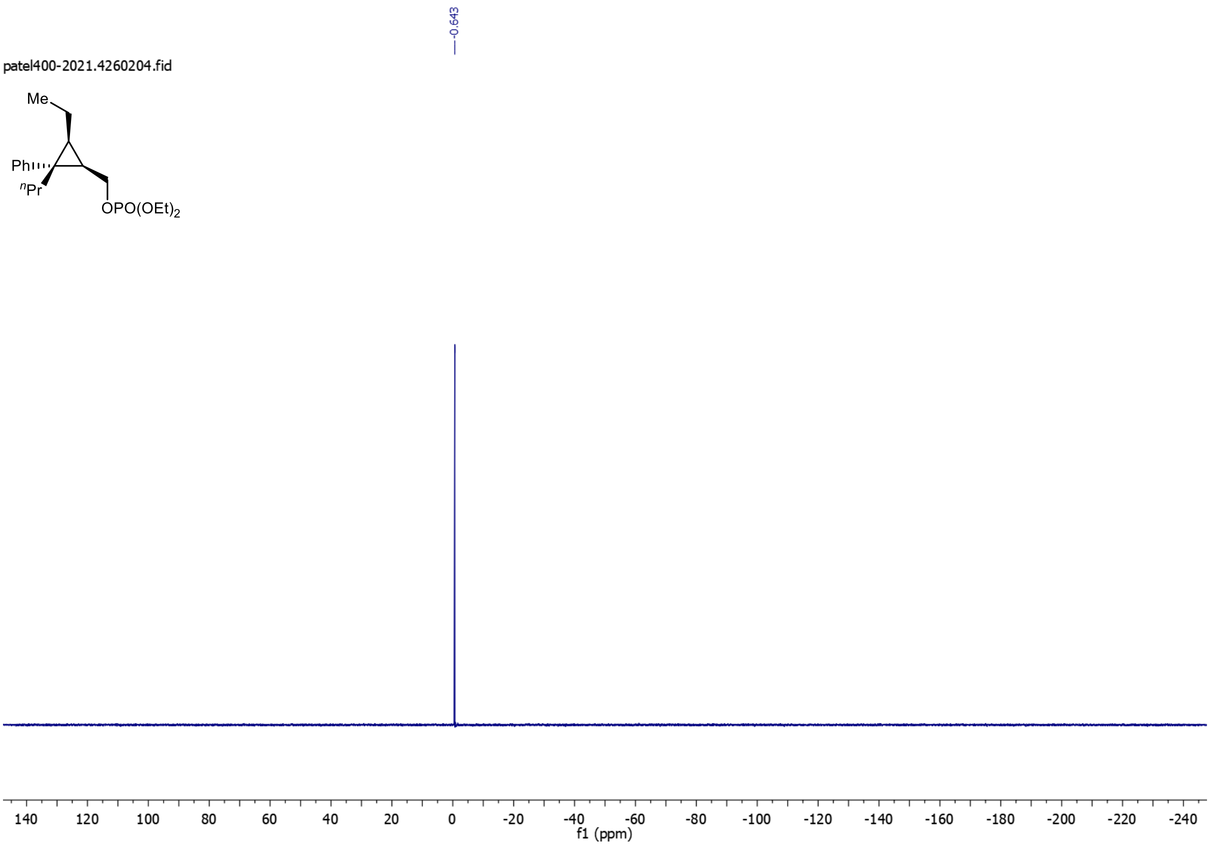
patel400-2021.4260204.fid
-0.643
Me
Ph
nPr
OPO(OEt)2
140 120 100 80 60 40 20 0 -20 -40 -60 -80 -100 -120 -140 -160 -180 -200 -220 -240
f1 (ppm)

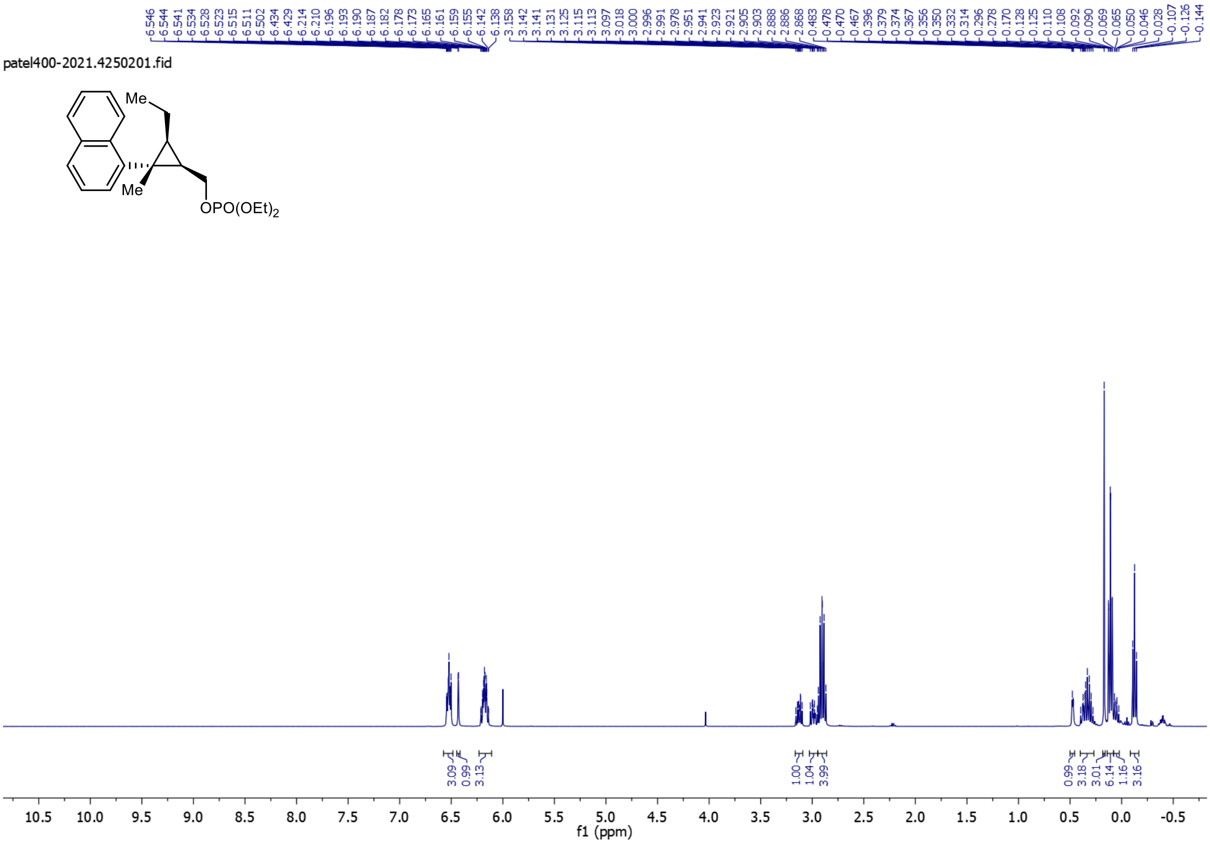
patel400-2021.4250201.fid
6.546 6.544 6.541 6.534 6.528 6.523 6.515 6.511 6.502 6.434 6.429 6.214 6.210 6.196 6.193 6.190 6.187 6.182 6.178 6.173 6.165 6.161 6.159 6.155 6.142 6.138 3.158 3.142 3.141 3.131 3.125 3.115 3.113 3.097 3.018 3.000 2.996 2.991 2.978 2.951 2.941 2.923 2.921 2.905 2.903 2.888 2.886 2.868 0.483 0.478 0.470 0.467 0.396 0.379 0.374 0.367 0.356 0.350 0.332 0.314 0.296 0.278 0.170 0.128 0.125 0.110 0.108 0.092 0.090 0.069 0.065 0.050 0.046 0.028 -0.107 -0.126 -0.144
Me
Me
OPO(OEt)2
3.09 0.99 3.13
1.00 1.04 3.99
0.99 3.18 3.01 6.14 1.16 3.16
10.5 10.0 9.5 9.0 8.5 8.0 7.5 7.0 6.5 6.0 5.5 5.0 4.5 4.0 3.5 3.0 2.5 2.0 1.5 1.0 0.5 0.0 -0.5
f1 (ppm)

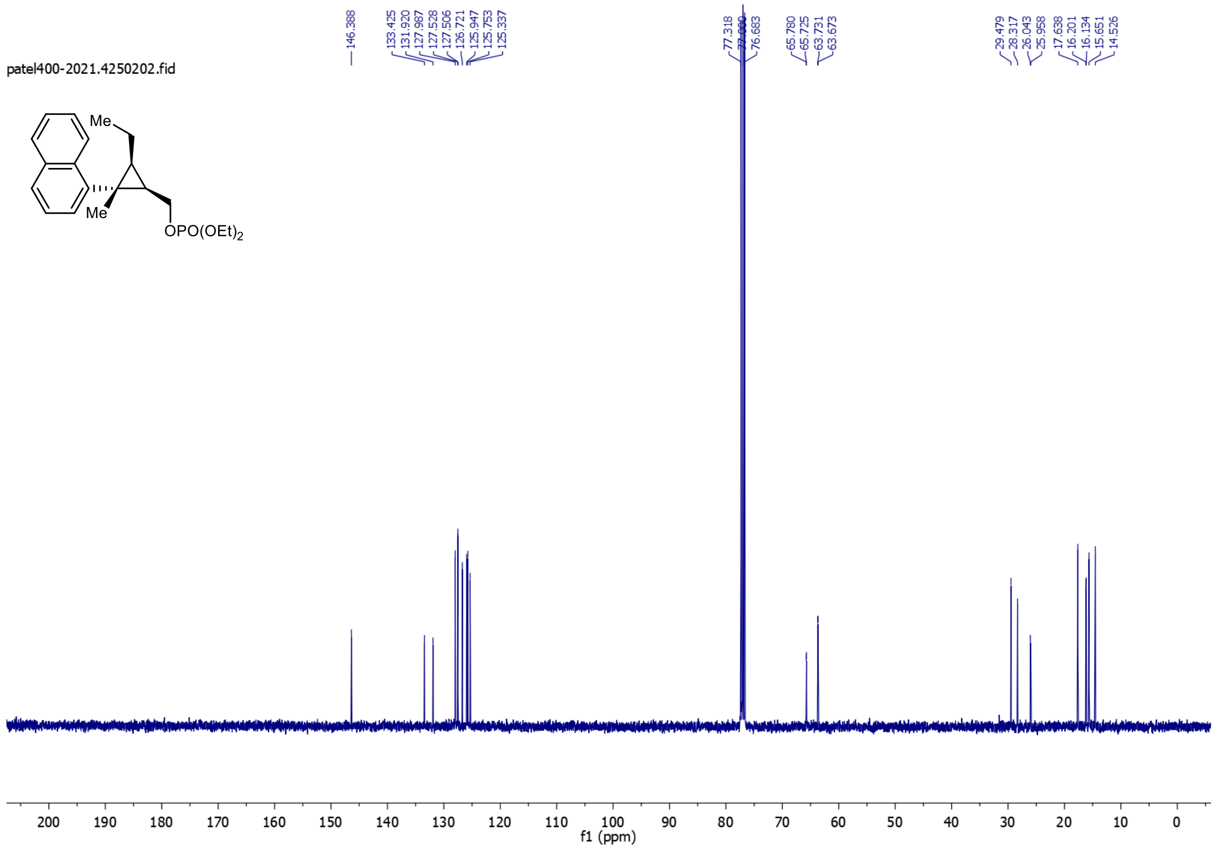
patel400-2021.4250202.fid
Me
Me
OPO(OEt)2
146.388
133.425
131.920
127.987
127.528
127.506
126.721
125.947
125.753
125.337
77.318
76.683
65.780
65.725
63.731
63.673
29.479
28.317
26.043
25.958
17.638
16.201
16.134
15.651
14.526
f1 (ppm)

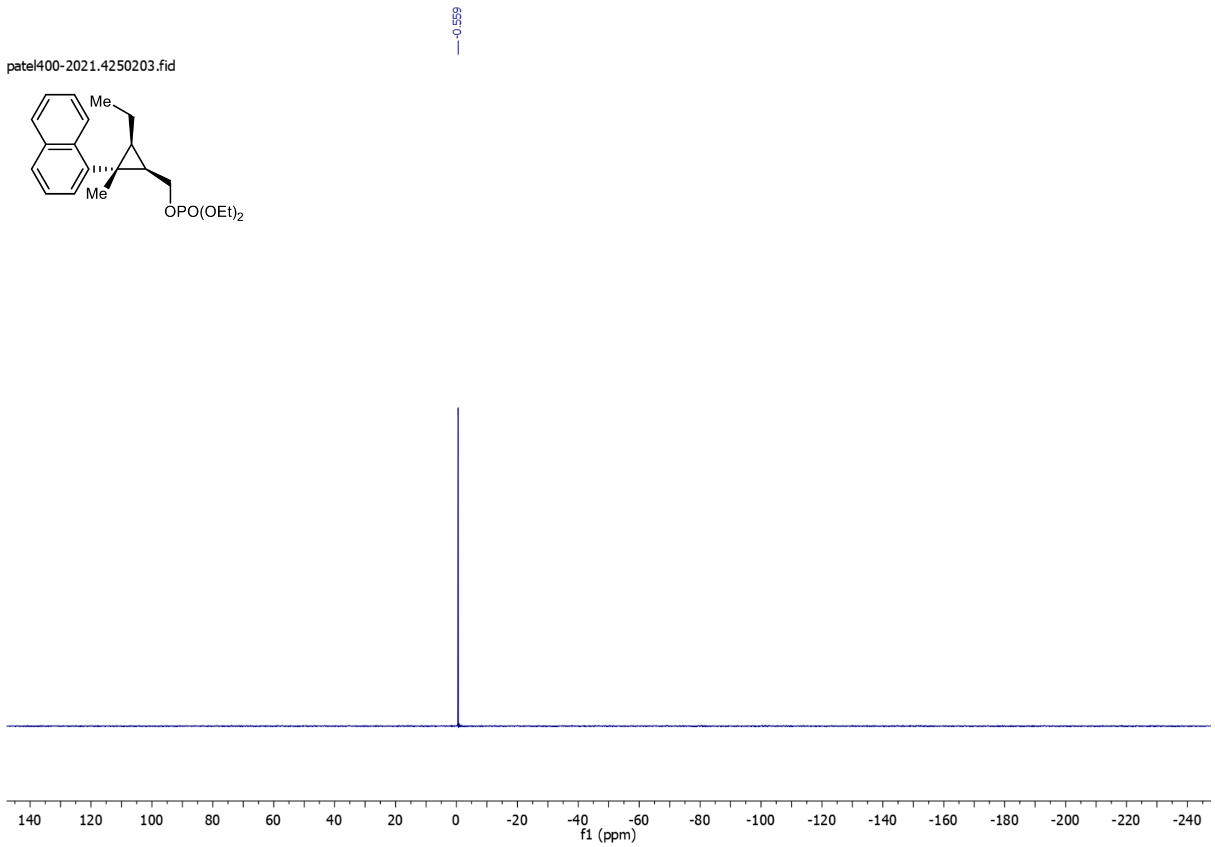
patel400-2021.4250203.fid
Me
Me
OPO(OEt)2
-0.559
f1 (ppm)

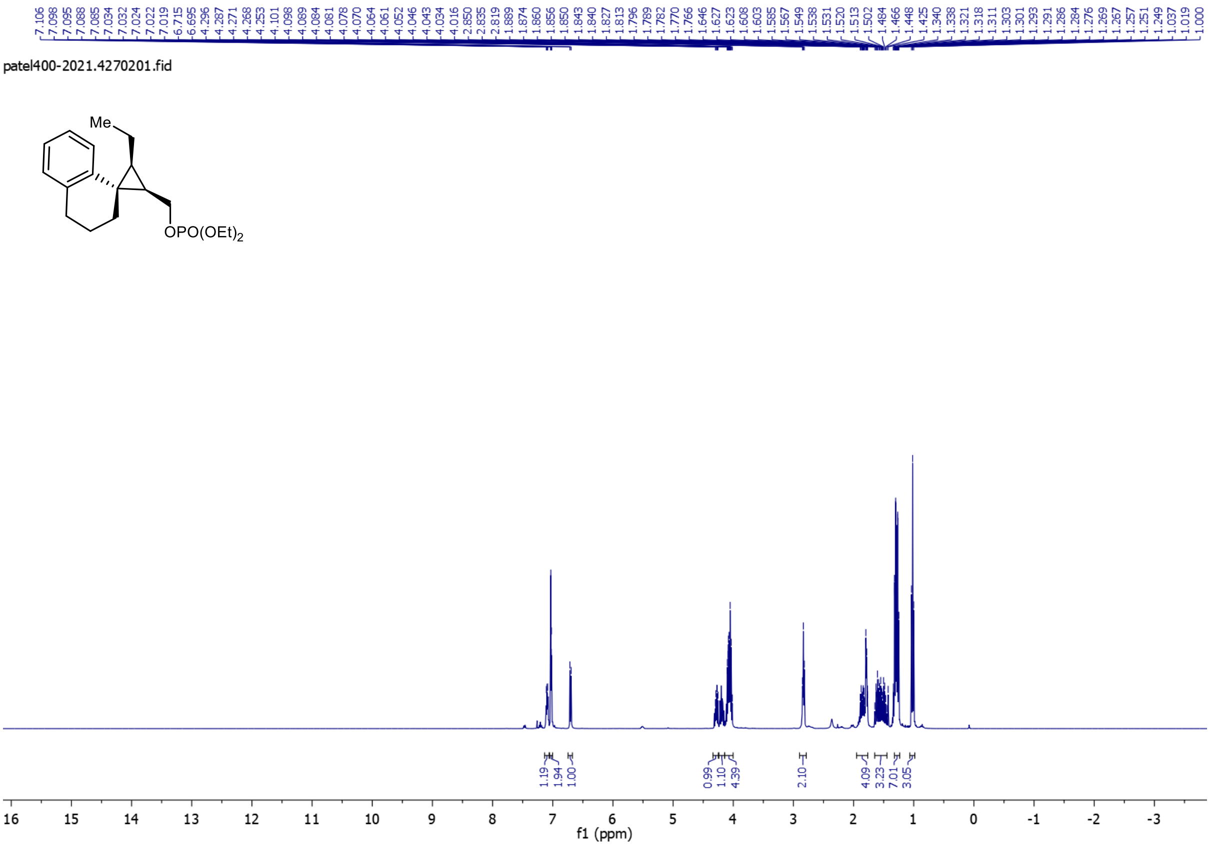
7.106 7.098 7.095 7.088 7.085 7.034 7.032 7.024 7.022 7.019 6.715 6.695 4.296 4.287 4.271 4.268 4.253 4.101 4.098 4.089 4.084 4.081 4.078 4.070 4.064 4.061 4.052 4.046 4.043 4.034 4.016 2.850 2.835 2.819 1.889 1.874 1.860 1.856 1.850 1.843 1.840 1.827 1.813 1.796 1.789 1.782 1.770 1.766 1.646 1.627 1.623 1.608 1.603 1.585 1.567 1.549 1.538 1.531 1.520 1.513 1.502 1.484 1.466 1.448 1.425 1.340 1.338 1.321 1.318 1.311 1.303 1.301 1.293 1.291 1.286 1.284 1.276 1.269 1.267 1.257 1.251 1.249 1.037 1.019 1.000
patel400-2021.4270201.fid
Me
OPO(OEt)2
1.19 1.94 1.00 0.99 1.10 4.39 2.10 4.09 3.23 7.01 3.05
16 15 14 13 12 11 10 9 8 7 6 5 4 3 2 1 0 -1 -2 -3
f1 (ppm)

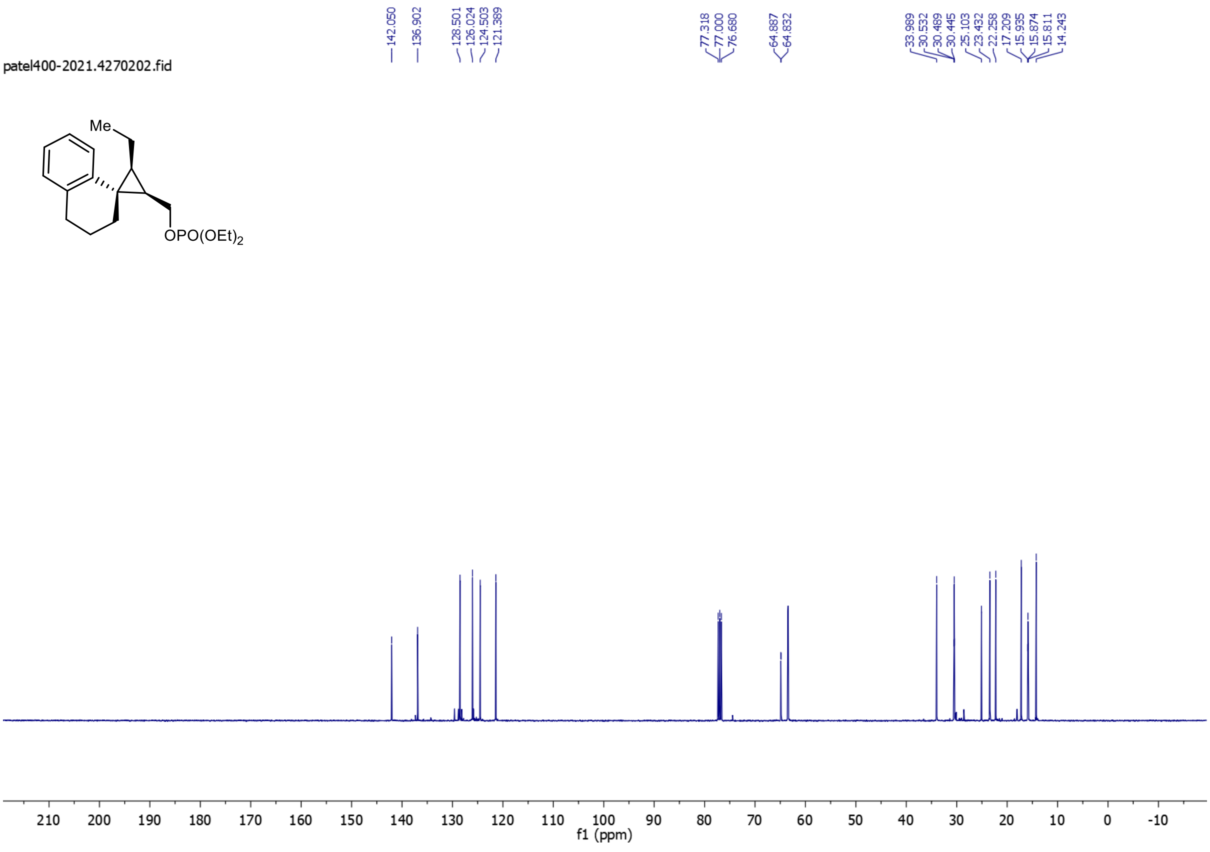
142.050 136.902 128.501 126.024 124.503 121.389 77.318 77.000 76.680 64.887 64.832 33.989 30.532 30.489 30.445 25.103 23.432 22.258 17.209 15.935 15.874 15.811 14.243
patel400-2021.4270202.fid
Me
OPO(OEt)2
210 200 190 180 170 160 150 140 130 120 110 100 90 80 70 60 50 40 30 20 10 0 -10
f1 (ppm)

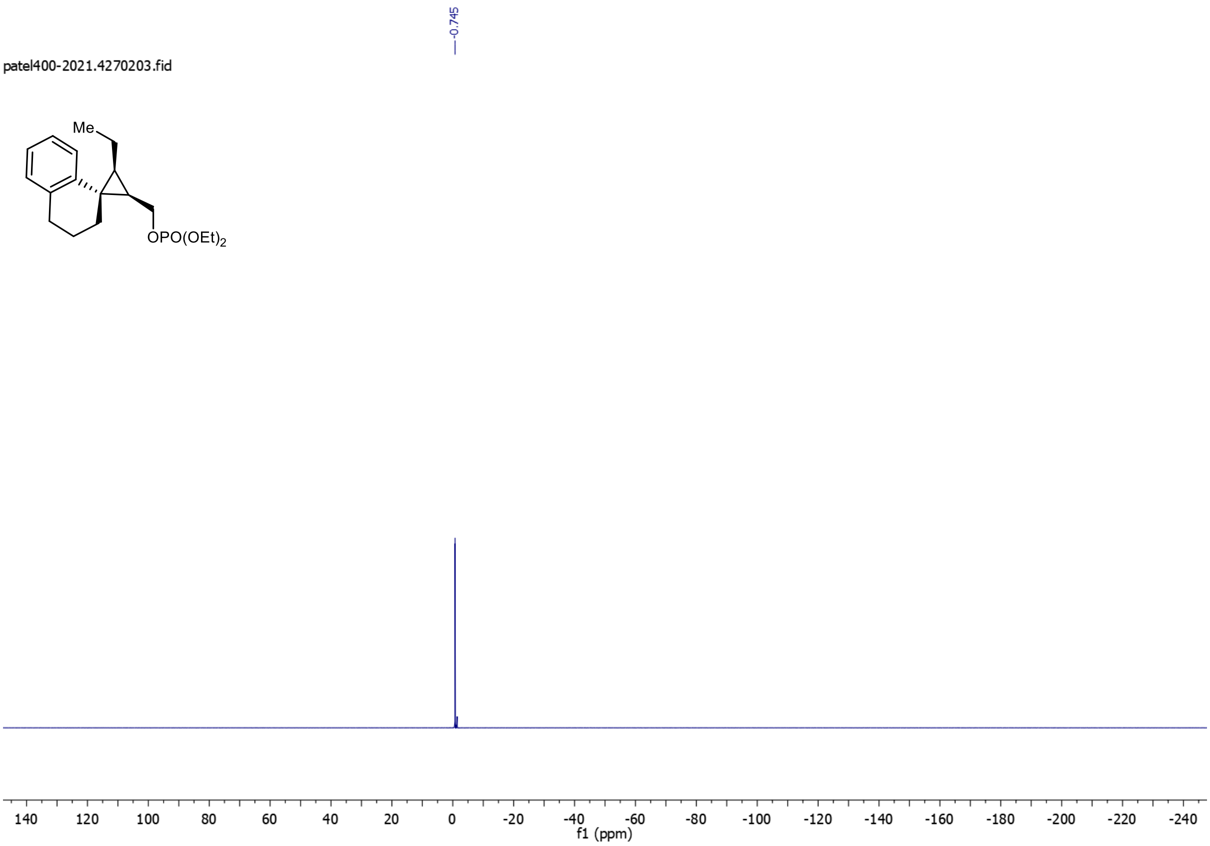
-0.745
patel400-2021.4270203.fid
Me
OPO(OEt)2
140 120 100 80 60 40 20 0 -20 -40 -60 -80 -100 -120 -140 -160 -180 -200 -220 -240
f1 (ppm)

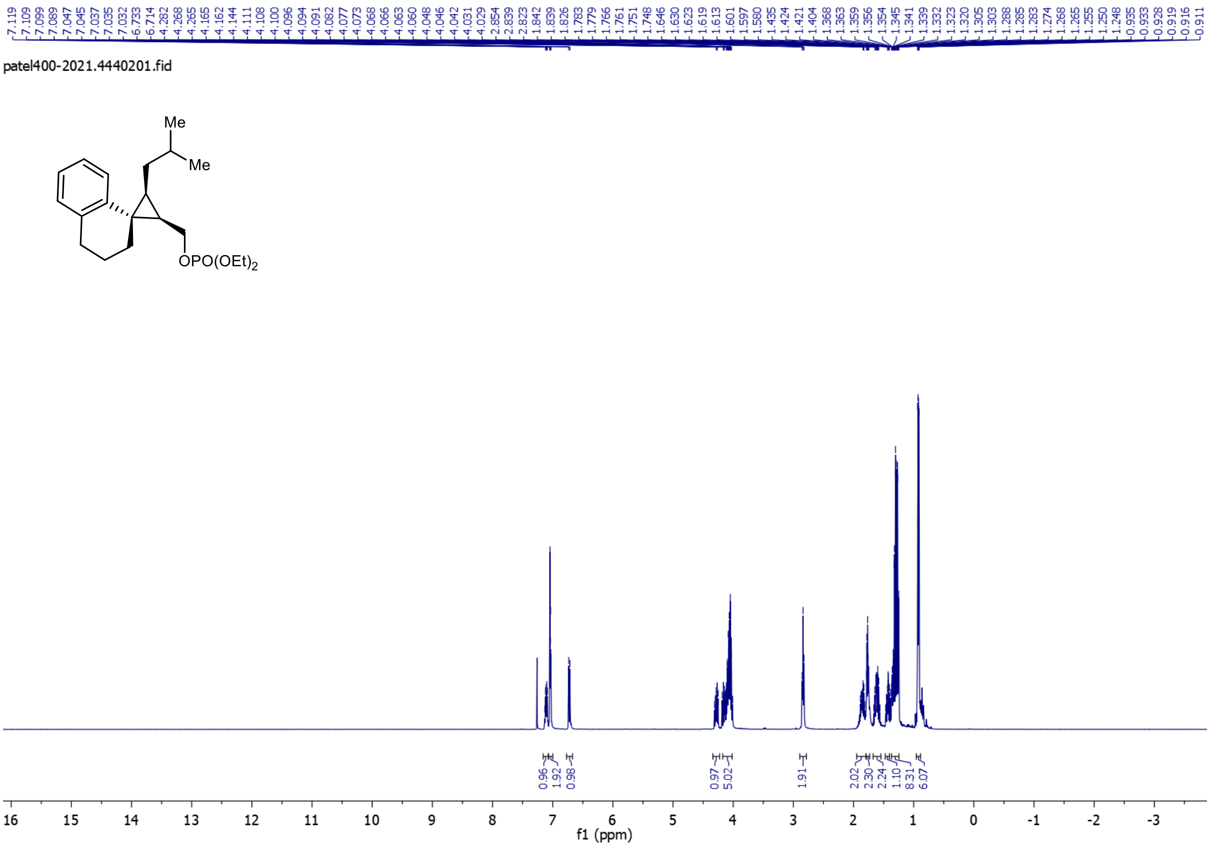
7.119 7.109 7.099 7.089 7.047 7.045 7.037 7.035 7.032 6.733 6.714 4.282 4.268 4.265 4.165 4.162 4.144 4.111 4.108 4.100 4.096 4.094 4.091 4.082 4.077 4.073 4.068 4.066 4.063 4.060 4.048 4.046 4.042 4.031 4.029 2.854 2.839 2.823 1.842 1.839 1.826 1.783 1.779 1.766 1.761 1.751 1.748 1.646 1.630 1.623 1.619 1.613 1.601 1.597 1.580 1.435 1.424 1.421 1.404 1.368 1.363 1.359 1.356 1.354 1.345 1.341 1.339 1.332 1.323 1.320 1.305 1.303 1.288 1.285 1.283 1.274 1.268 1.265 1.255 1.250 1.248 0.935 0.933 0.928 0.919 0.916 0.911
patel400-2021.4440201.fid
Me
Me
OPO(OEt)2
0.96 1.92 0.98 0.97 5.02 1.91 2.02 2.30 2.24 1.10 8.31 6.07
16 15 14 13 12 11 10 9 8 7 6 5 4 3 2 1 0 -1 -2 -3
f1 (ppm)

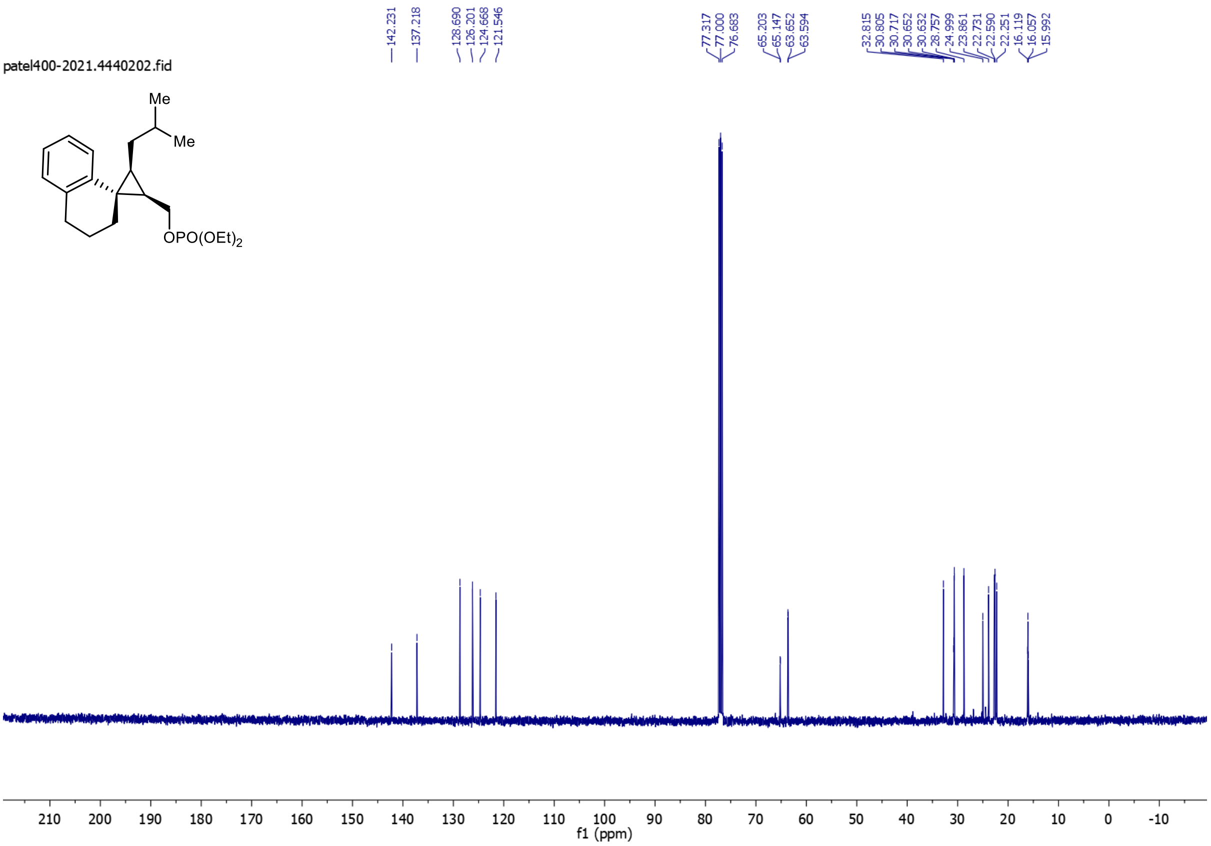
patel400-2021.4440202.fid
Me
Me
OPO(OEt)2
142.231
137.218
128.690
126.201
124.668
121.546
77.317
77.000
76.683
65.203
65.147
63.652
63.594
32.815
30.805
30.717
30.652
30.632
28.757
24.999
23.861
22.731
22.590
22.251
16.119
16.057
15.992
f1 (ppm)

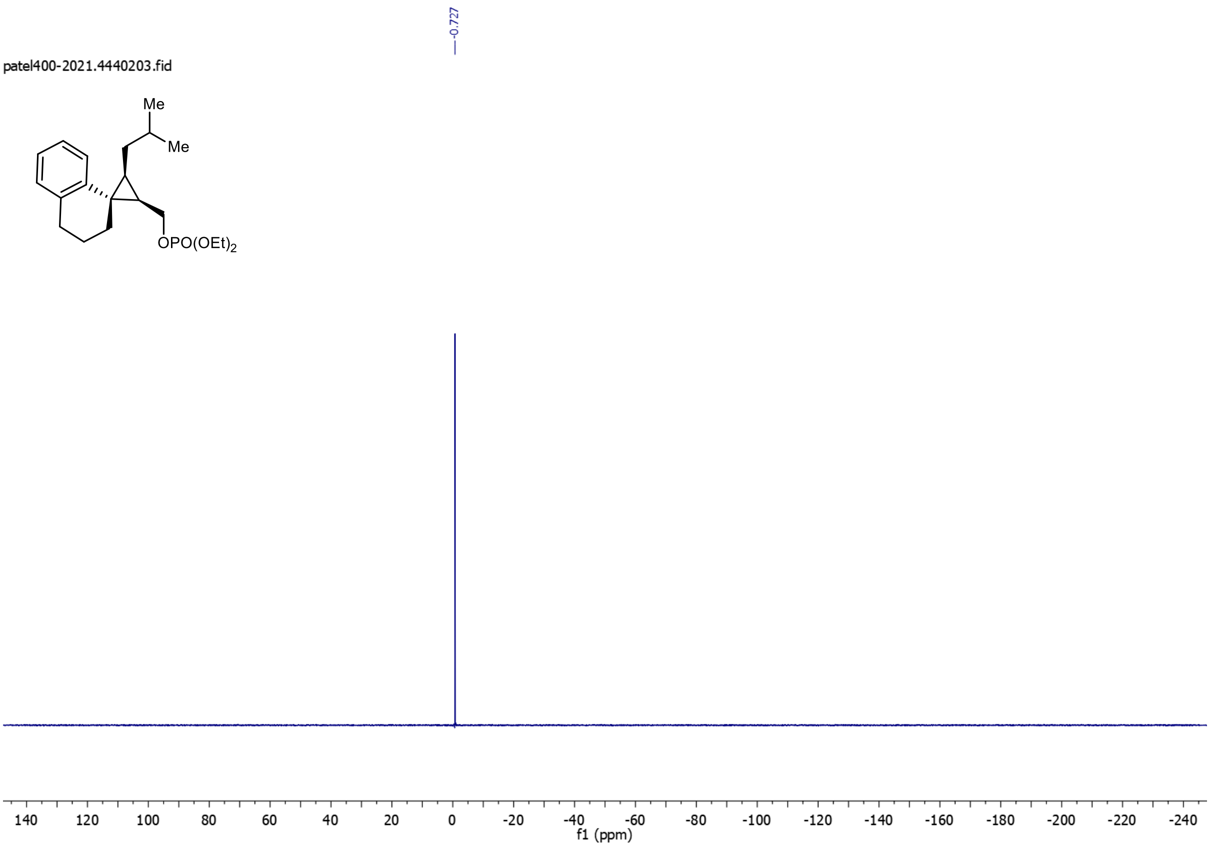
patel400-2021.4440203.fid
Me
Me
OPO(OEt)2
-0.727
f1 (ppm)

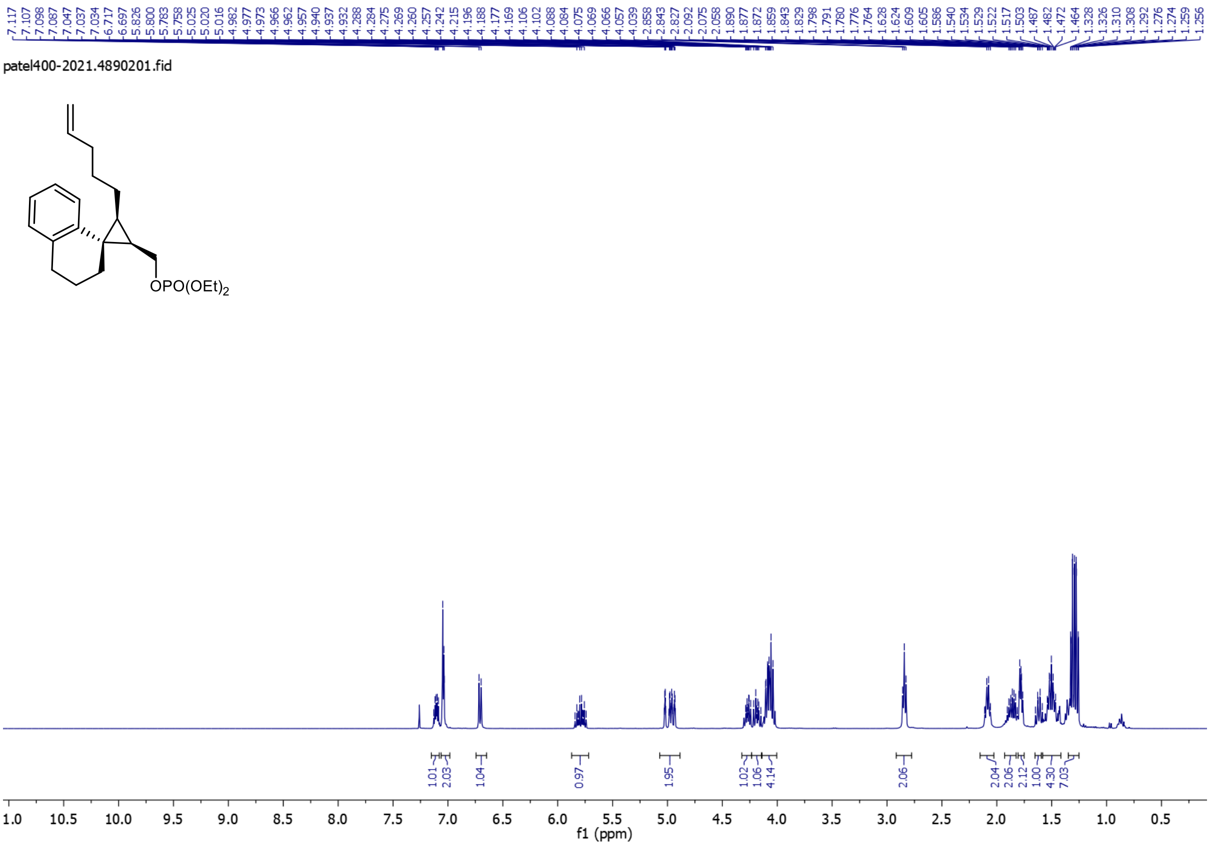
patel400-2021.4890201.fid
OPO(OEt)2
f1 (ppm)

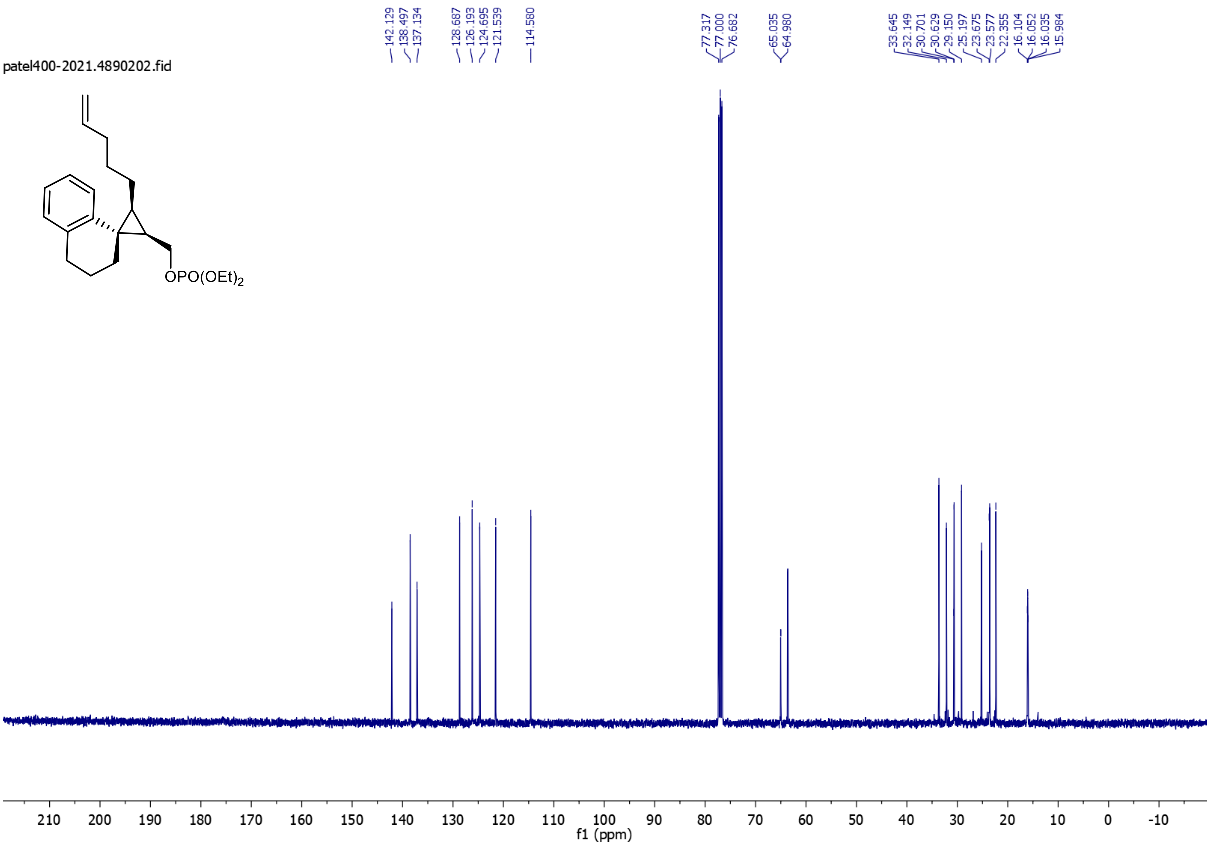
patel400-2021.4890202.fid
OPO(OEt)2
f1 (ppm)

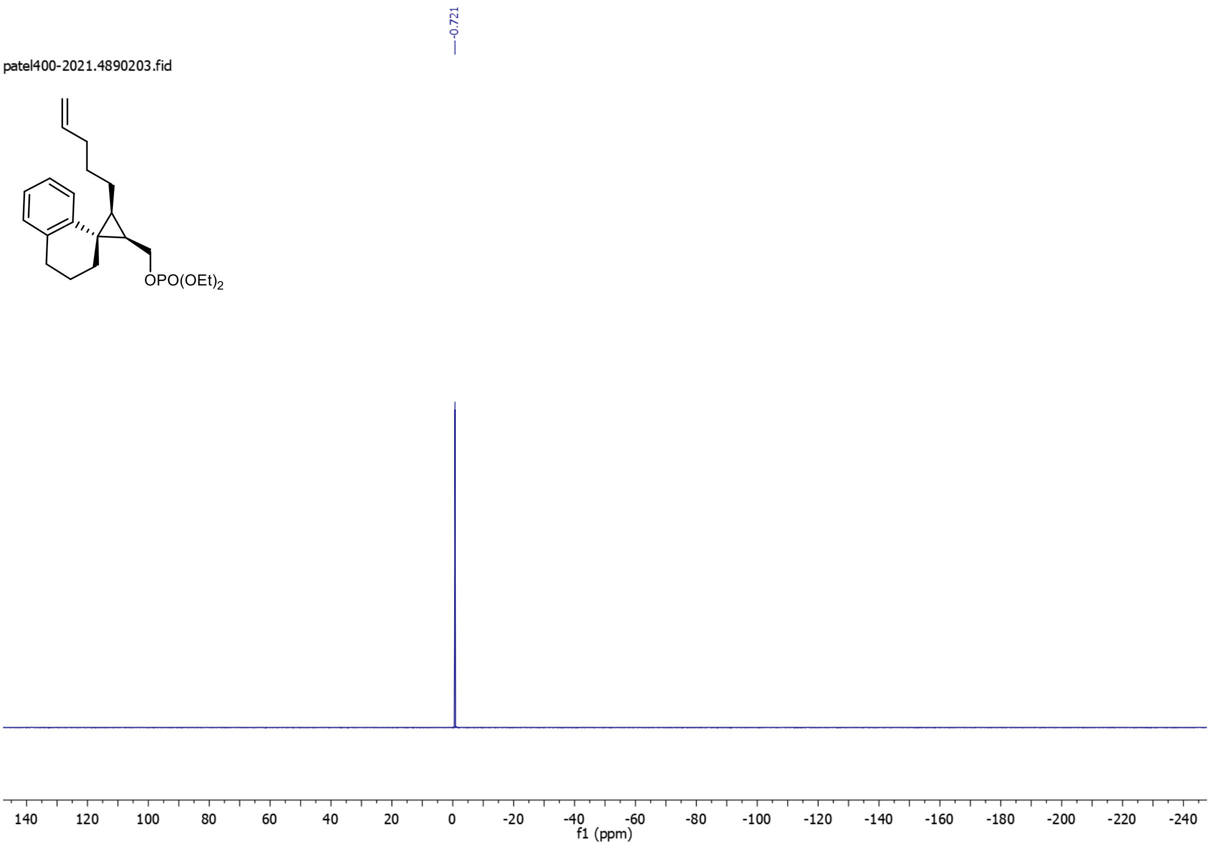
patel400-2021.4890203.fid
-0.721
OPO(OEt)2
140 120 100 80 60 40 20 0 -20 -40 -60 -80 -100 -120 -140 -160 -180 -200 -220 -240
f1 (ppm)

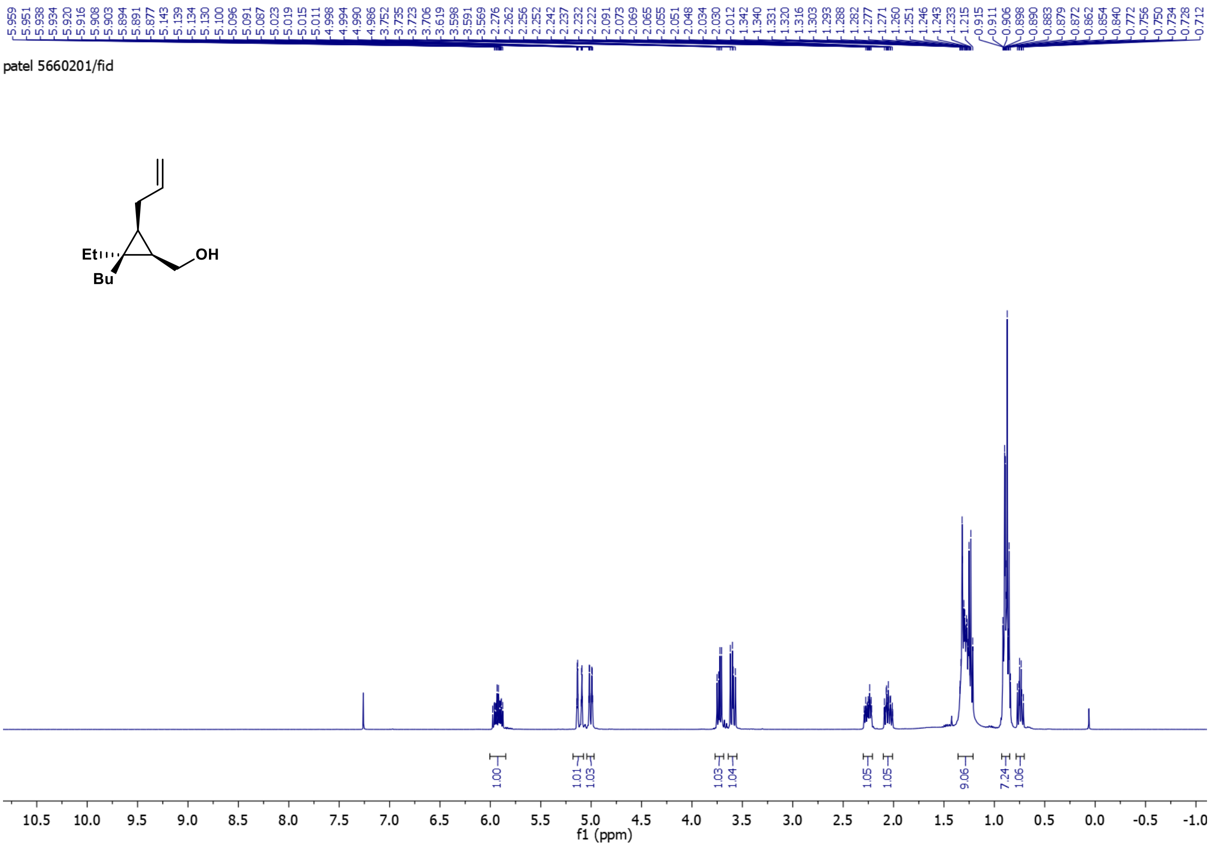
5.959 5.951 5.938 5.934 5.920 5.916 5.908 5.903 5.894 5.891 5.877 5.143 5.139 5.134 5.130 5.100 5.096 5.091 5.087 5.023 5.019 5.015 5.011 4.998 4.994 4.990 4.986 3.752 3.735 3.723 3.706 3.619 3.598 3.591 3.569 2.276 2.262 2.256 2.252 2.242 2.237 2.232 2.222 2.091 2.073 2.069 2.065 2.055 2.051 2.048 2.034 2.030 2.012 1.342 1.340 1.331 1.320 1.316 1.303 1.293 1.288 1.282 1.277 1.271 1.260 1.251 1.246 1.243 1.233 1.215 0.915 0.911 0.906 0.898 0.890 0.883 0.879 0.872 0.862 0.854 0.840 0.772 0.756 0.750 0.734 0.728 0.712
patel 5660201/fid
Et
Bu
OH
1.00
1.01
1.03
1.03
1.04
1.05
1.05
9.06
7.24
1.06
10.5 10.0 9.5 9.0 8.5 8.0 7.5 7.0 6.5 6.0 5.5 5.0 4.5 4.0 3.5 3.0 2.5 2.0 1.5 1.0 0.5 0.0 -0.5 -1.0
f1 (ppm)

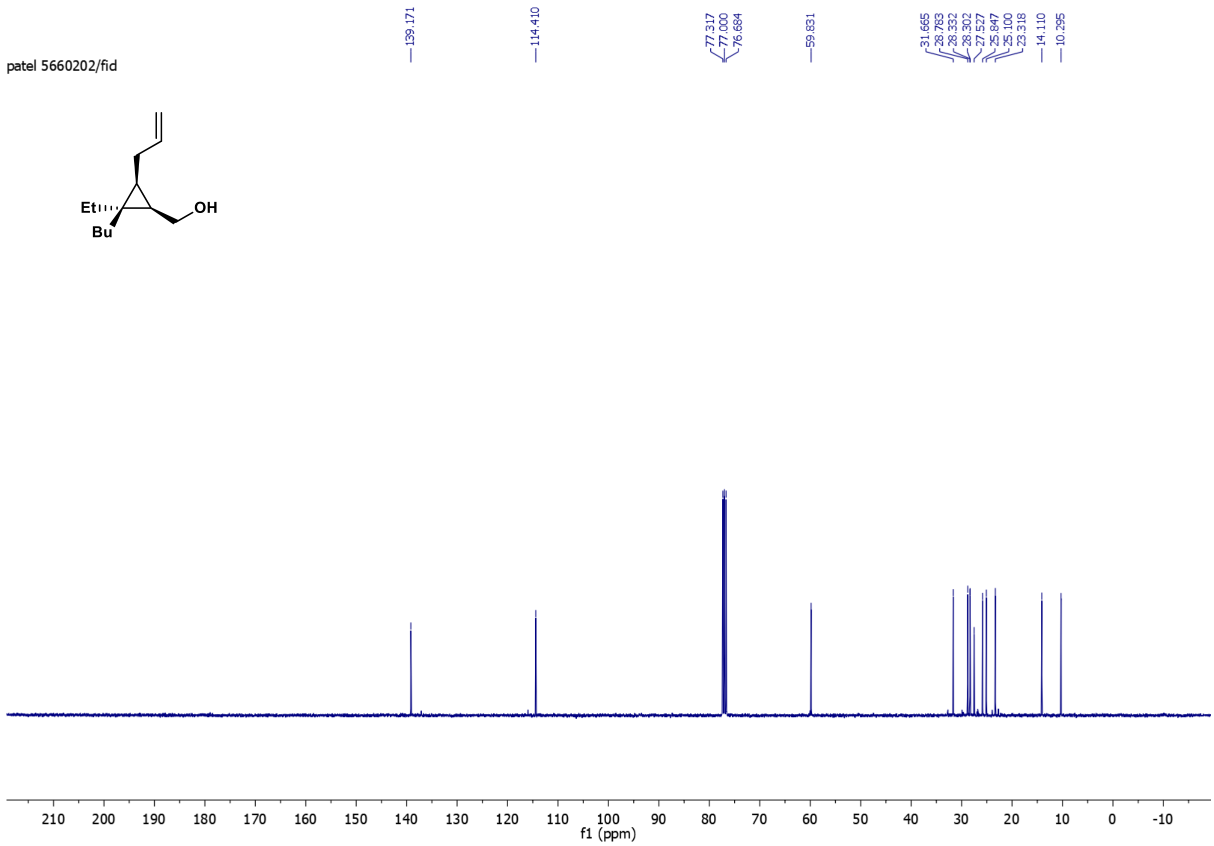

patel 5660202/fid
139.171
114.410
77.317
77.000
76.684
59.831
31.665
28.783
28.332
28.302
27.527
25.847
25.100
23.318
14.110
10.295
Et
Bu
OH
210 200 190 180 170 160 150 140 130 120 110 100 90 80 70 60 50 40 30 20 10 0 -10
f1 (ppm)

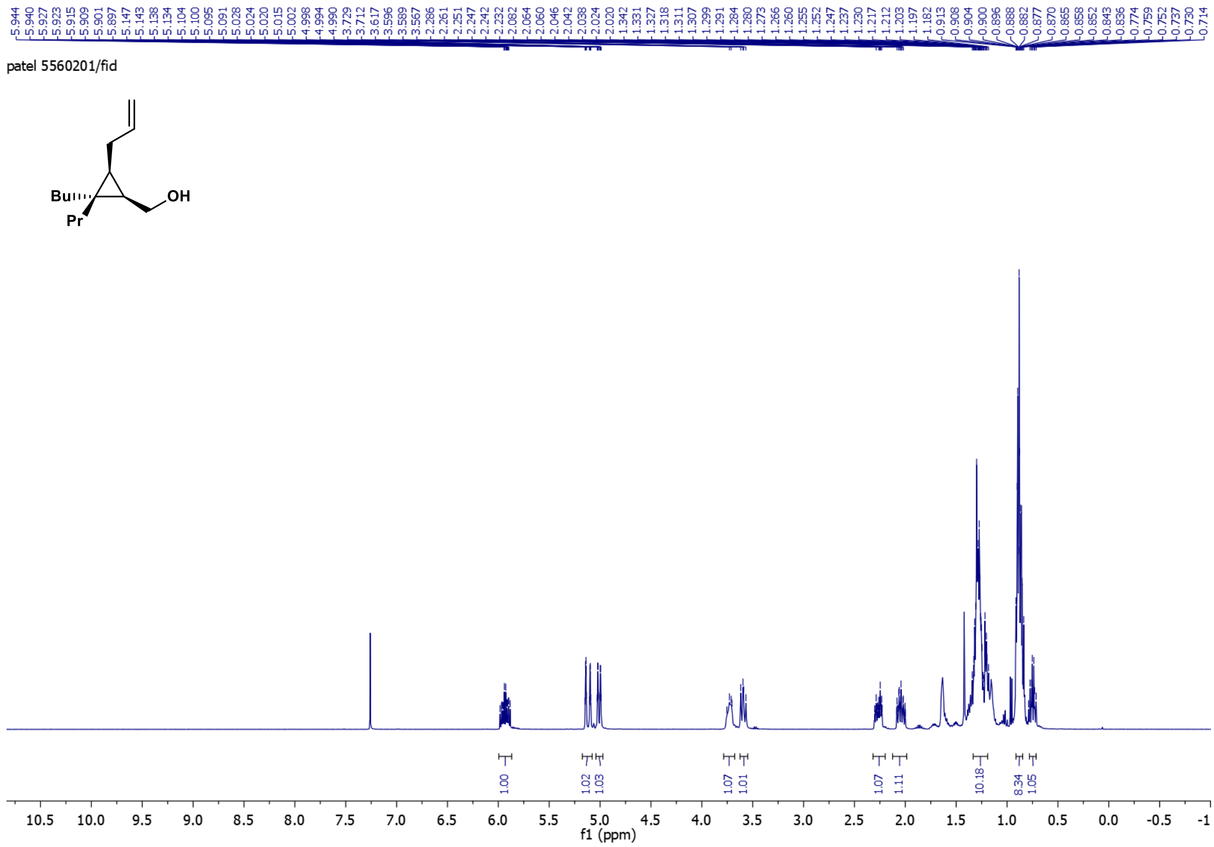

5.944 5.940 5.927 5.923 5.915 5.909 5.901 5.897 5.147 5.143 5.138 5.134 5.104 5.100 5.095 5.091 5.028 5.024 5.020 5.015 5.002 4.998 4.994 4.990 3.729 3.712 3.617 3.596 3.589 3.567 2.286 2.261 2.251 2.247 2.242 2.232 2.082 2.064 2.060 2.046 2.042 2.038 2.024 2.020 1.342 1.331 1.327 1.318 1.311 1.307 1.299 1.291 1.284 1.280 1.273 1.266 1.260 1.255 1.252 1.247 1.237 1.230 1.217 1.212 1.203 1.197 1.182 0.913 0.908 0.904 0.900 0.896 0.888 0.882 0.877 0.870 0.865 0.858 0.852 0.843 0.836 0.774 0.759 0.752 0.737 0.730 0.714
patel 5560201/fid
Bu
Pr
OH
1.00
1.02
1.03
1.07
1.01
1.07
1.11
10.18
8.34
1.05
10.5 10.0 9.5 9.0 8.5 8.0 7.5 7.0 6.5 6.0 5.5 5.0 4.5 4.0 3.5 3.0 2.5 2.0 1.5 1.0 0.5 0.0 -0.5 -1
f1 (ppm)

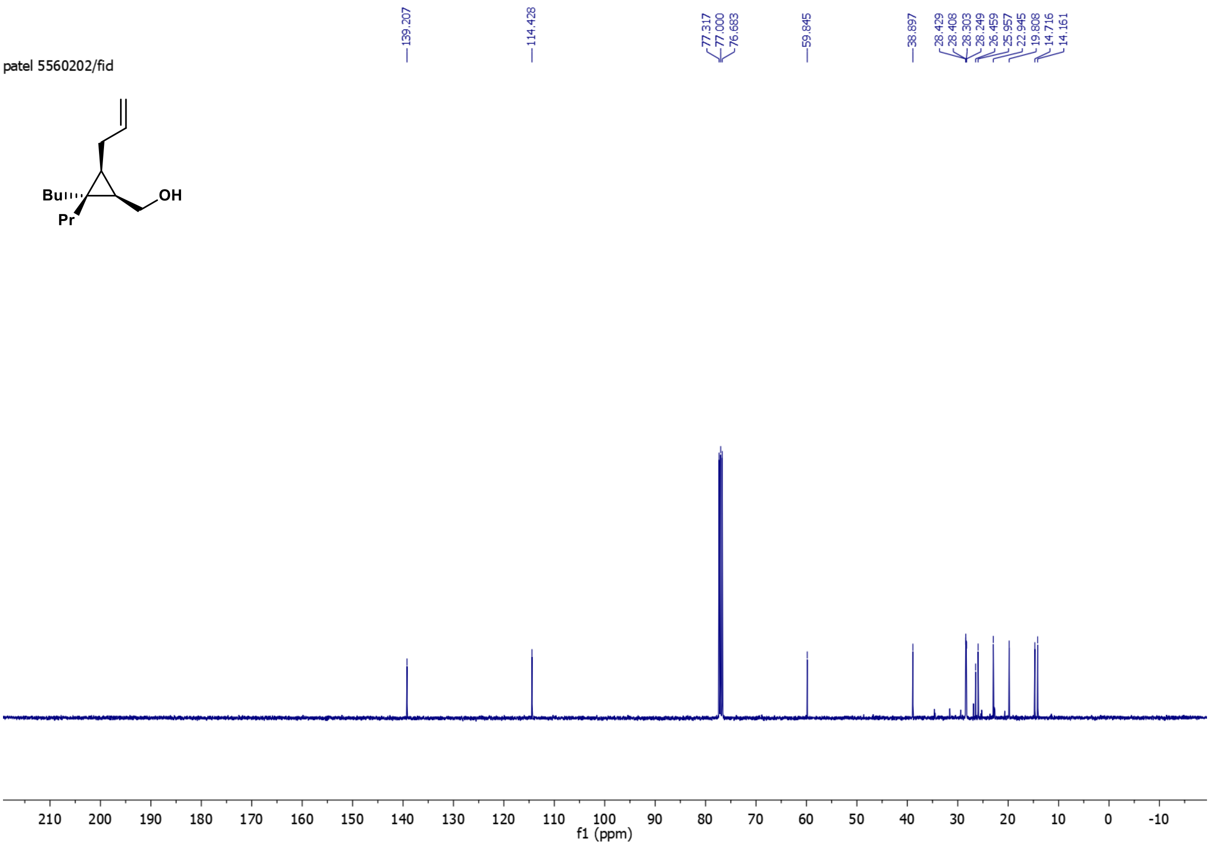

139.207
114.428
77.317
77.000
76.683
59.845
38.897
28.429
28.408
28.303
28.249
26.459
25.957
22.945
19.808
14.716
14.161
patel 5560202/fid
Bu
Pr
OH
210 200 190 180 170 160 150 140 130 120 110 100 90 80 70 60 50 40 30 20 10 0 -10
f1 (ppm)

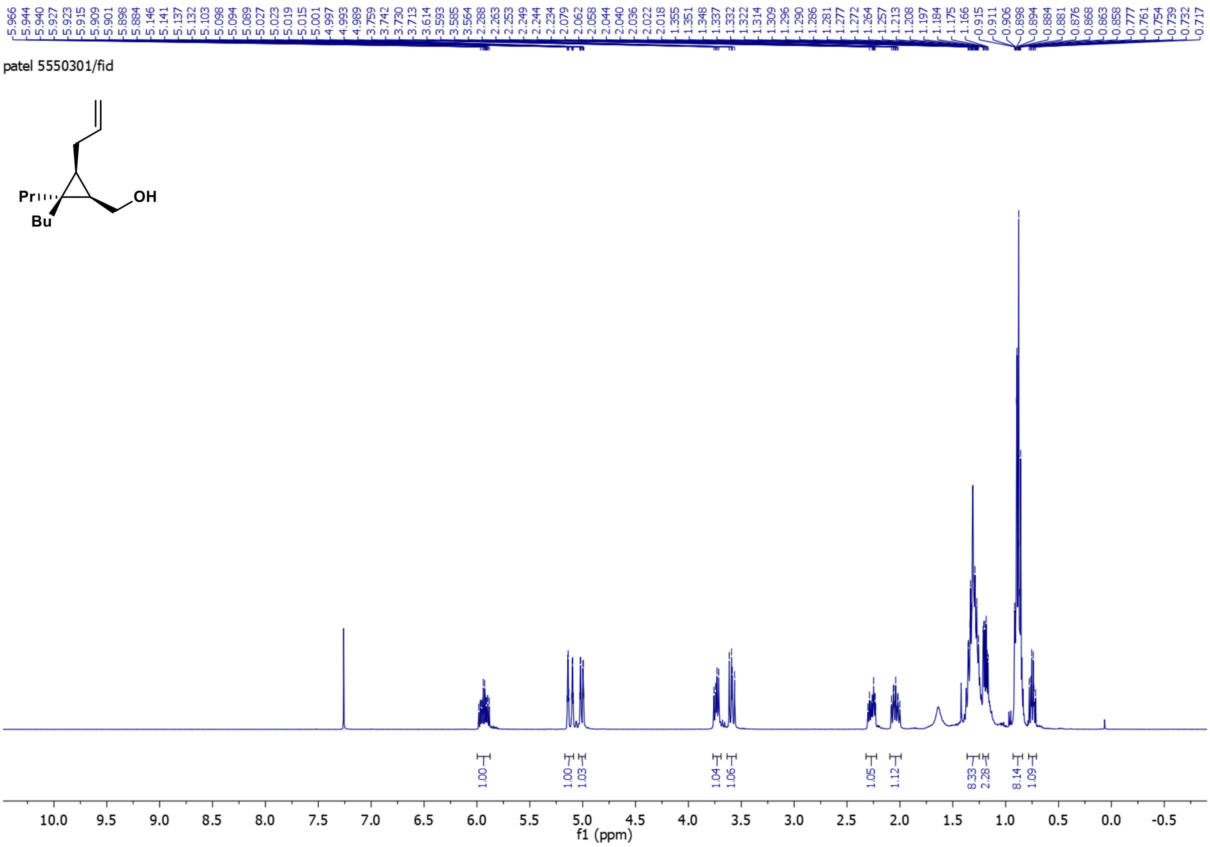

5.966 5.944 5.940 5.927 5.923 5.915 5.909 5.901 5.898 5.884 5.146 5.141 5.137 5.132 5.103 5.098 5.094 5.089 5.027 5.023 5.019 5.015 5.001 4.997 4.993 4.989 3.759 3.742 3.730 3.713 3.614 3.593 3.585 3.564 2.288 2.263 2.253 2.249 2.244 2.234 2.079 2.062 2.058 2.044 2.040 2.036 2.022 2.018 1.355 1.351 1.348 1.337 1.332 1.322 1.314 1.309 1.296 1.290 1.286 1.281 1.277 1.272 1.264 1.257 1.213 1.208 1.197 1.184 1.175 1.166 0.915 0.911 0.906 0.898 0.894 0.884 0.881 0.876 0.868 0.863 0.858 0.777 0.761 0.754 0.739 0.732 0.717
patel 5550301/fid
Pr
Bu
OH
1.00
1.00
1.03
1.04
1.06
1.05
1.12
8.33
2.28
8.14
1.09
10.0 9.5 9.0 8.5 8.0 7.5 7.0 6.5 6.0 5.5 5.0 4.5 4.0 3.5 3.0 2.5 2.0 1.5 1.0 0.5 0.0 -0.5
f1 (ppm)

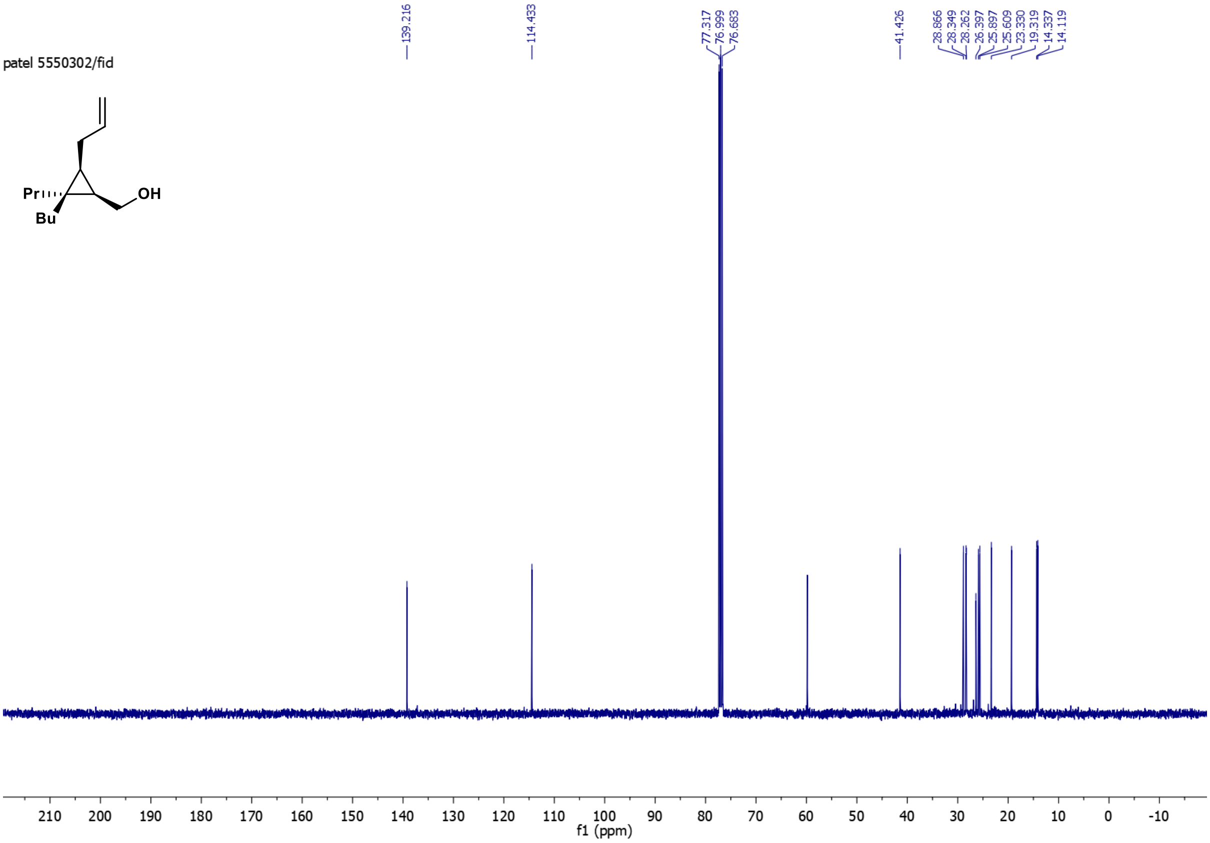
patel 5550302/fid
Pr
Bu
OH
139.216
114.433
77.317
76.999
76.683
41.426
28.866
28.349
28.262
26.397
25.897
25.609
23.330
19.319
14.337
14.119
210 200 190 180 170 160 150 140 130 120 110 100 90 80 70 60 50 40 30 20 10 0 -10
f1 (ppm)

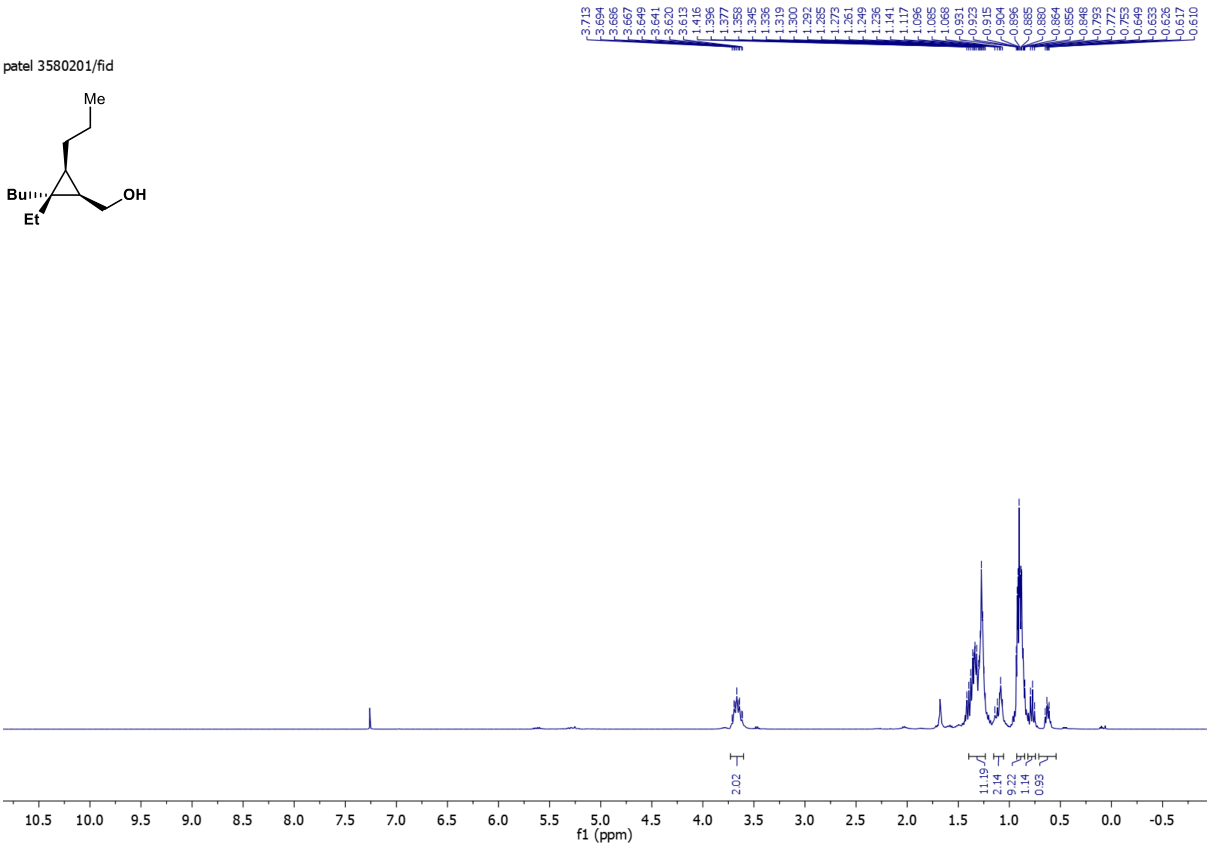
patel 3580201/fid
Me
Bu
Et
OH
3.713
3.694
3.686
3.667
3.649
3.641
3.620
3.613
1.416
1.396
1.377
1.358
1.345
1.336
1.319
1.300
1.292
1.285
1.273
1.261
1.249
1.236
1.141
1.117
1.096
1.085
1.068
0.931
0.923
0.915
0.904
0.896
0.885
0.880
0.864
0.856
0.848
0.793
0.772
0.753
0.649
0.633
0.626
0.617
0.610
2.02
11.19
2.14
9.22
1.14
0.93
10.5 10.0 9.5 9.0 8.5 8.0 7.5 7.0 6.5 6.0 5.5 5.0 4.5 4.0 3.5 3.0 2.5 2.0 1.5 1.0 0.5 0.0 -0.5
f1 (ppm)

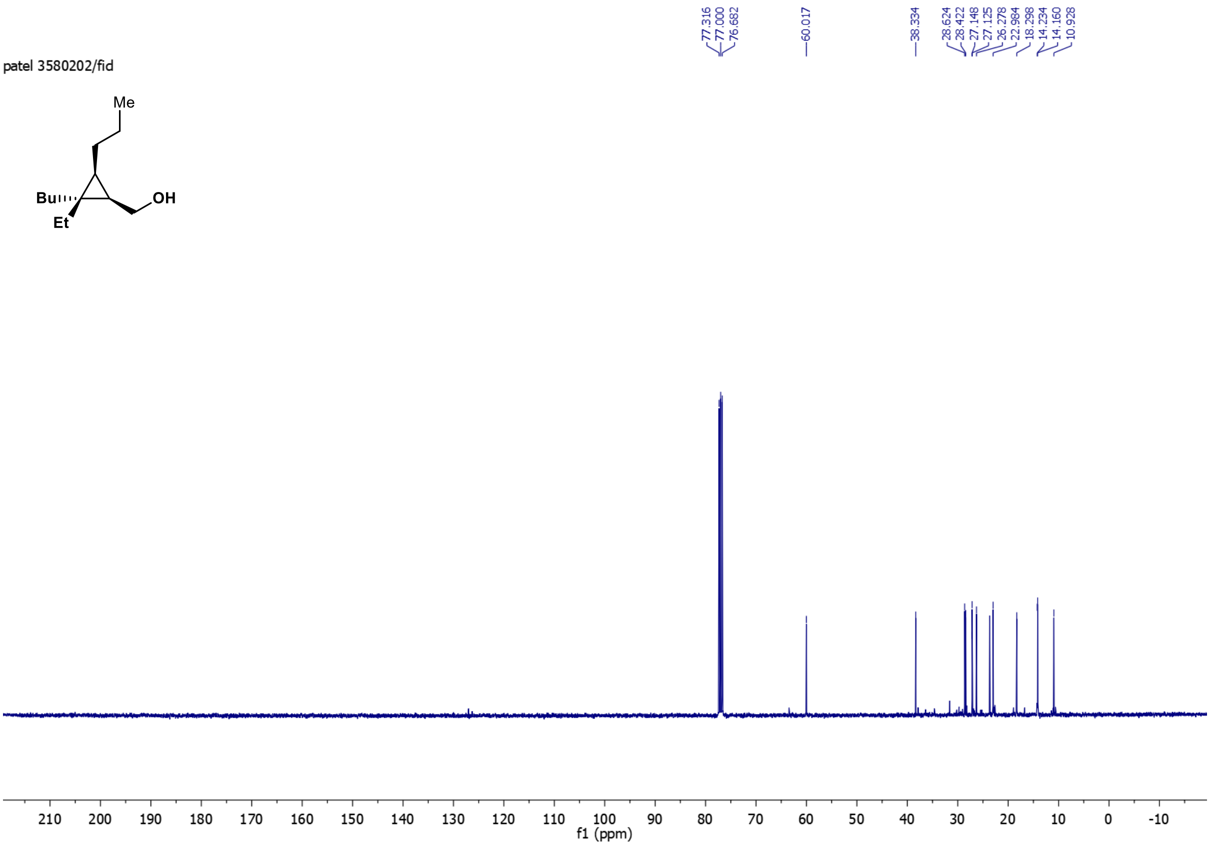
patel 3580202/fid
Me
Bu
OH
Et
77.316
77.000
76.682
60.017
38.334
28.624
28.422
27.148
27.125
26.278
22.984
18.298
14.234
14.160
10.928
210 200 190 180 170 160 150 140 130 120 110 100 90 80 70 60 50 40 30 20 10 0 -10
f1 (ppm)

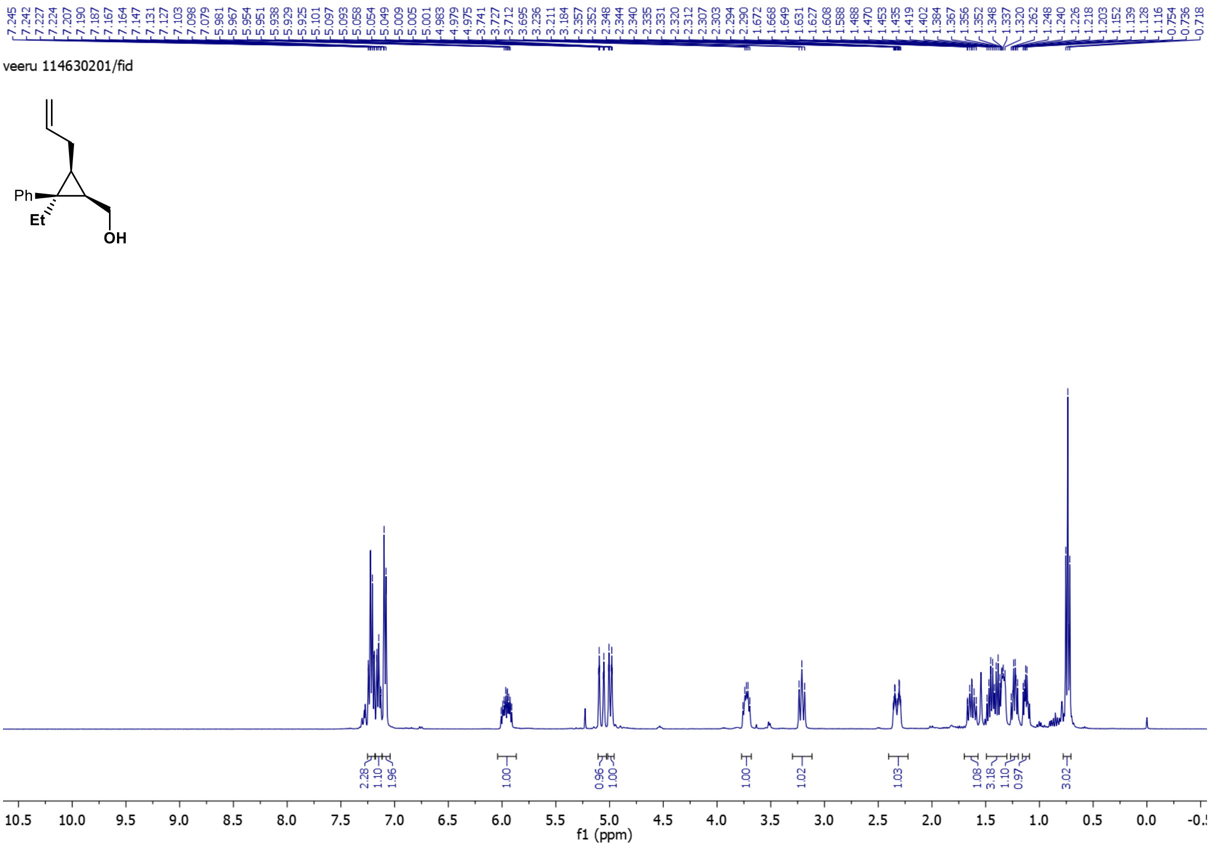
veeru 114630201/fid
Ph
Et
OH
7.245 7.242 7.227 7.224 7.207 7.190 7.187 7.167 7.164 7.147 7.131 7.127 7.103 7.098 7.079 5.981 5.967 5.954 5.951 5.938 5.929 5.925 5.101 5.097 5.093 5.058 5.054 5.049 5.009 5.005 5.001 4.983 4.979 4.975 3.741 3.727 3.712 3.695 3.226 3.211 3.184 2.357 2.352 2.348 2.344 2.340 2.335 2.331 2.320 2.312 2.307 2.303 2.294 2.290 1.672 1.668 1.649 1.631 1.627 1.608 1.588 1.488 1.470 1.453 1.435 1.419 1.402 1.384 1.367 1.356 1.352 1.348 1.337 1.320 1.262 1.248 1.240 1.226 1.218 1.203 1.152 1.139 1.128 1.116 0.754 0.736 0.718
2.28 1.10 1.96 1.00 0.96 1.00 1.00 1.02 1.03 1.08 3.18 1.10 0.97 3.02
10.5 10.0 9.5 9.0 8.5 8.0 7.5 7.0 6.5 6.0 5.5 5.0 4.5 4.0 3.5 3.0 2.5 2.0 1.5 1.0 0.5 0.0 -0.5
f1 (ppm)

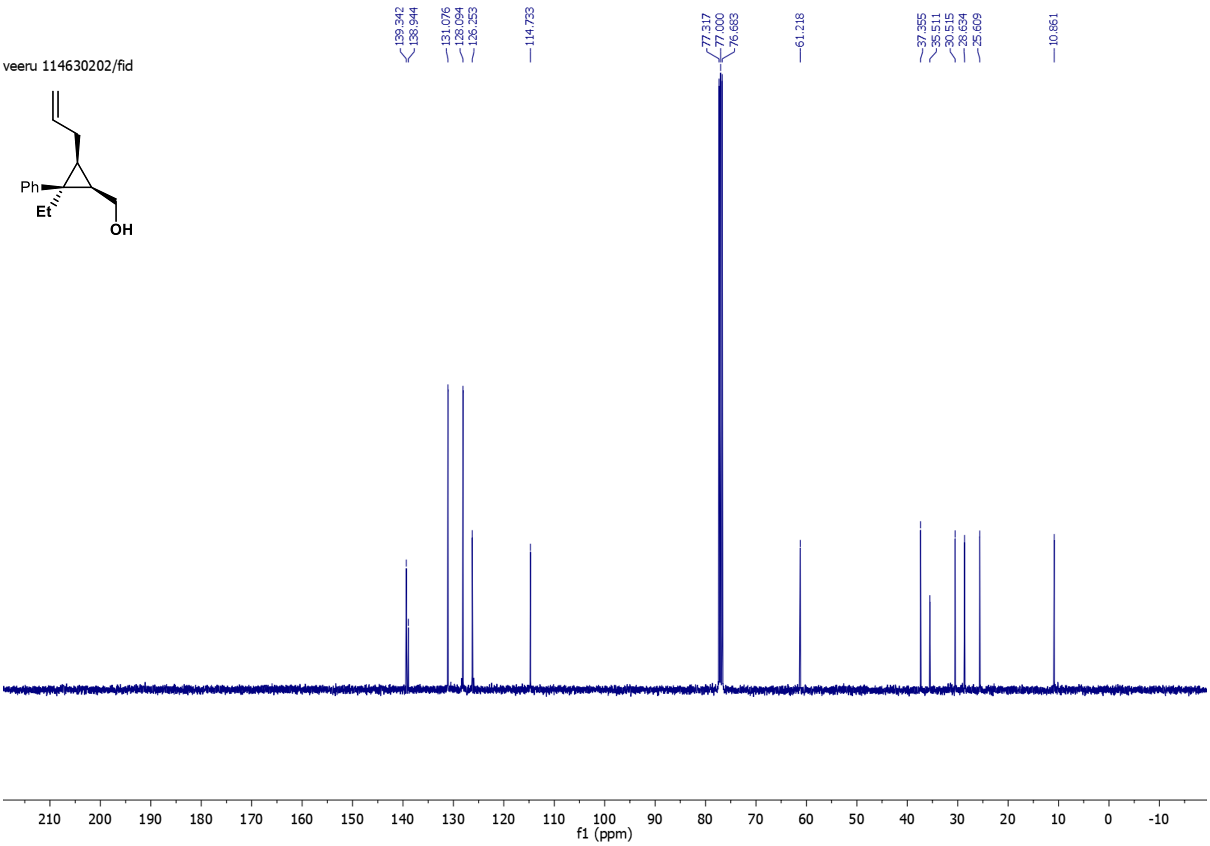
veeru 114630202/fid
Ph
Et
OH
139.342
138.944
131.076
128.094
126.253
114.733
77.317
77.000
76.683
61.218
37.355
35.511
30.515
28.634
25.609
10.861
210 200 190 180 170 160 150 140 130 120 110 100 90 80 70 60 50 40 30 20 10 0 -10
f1 (ppm)

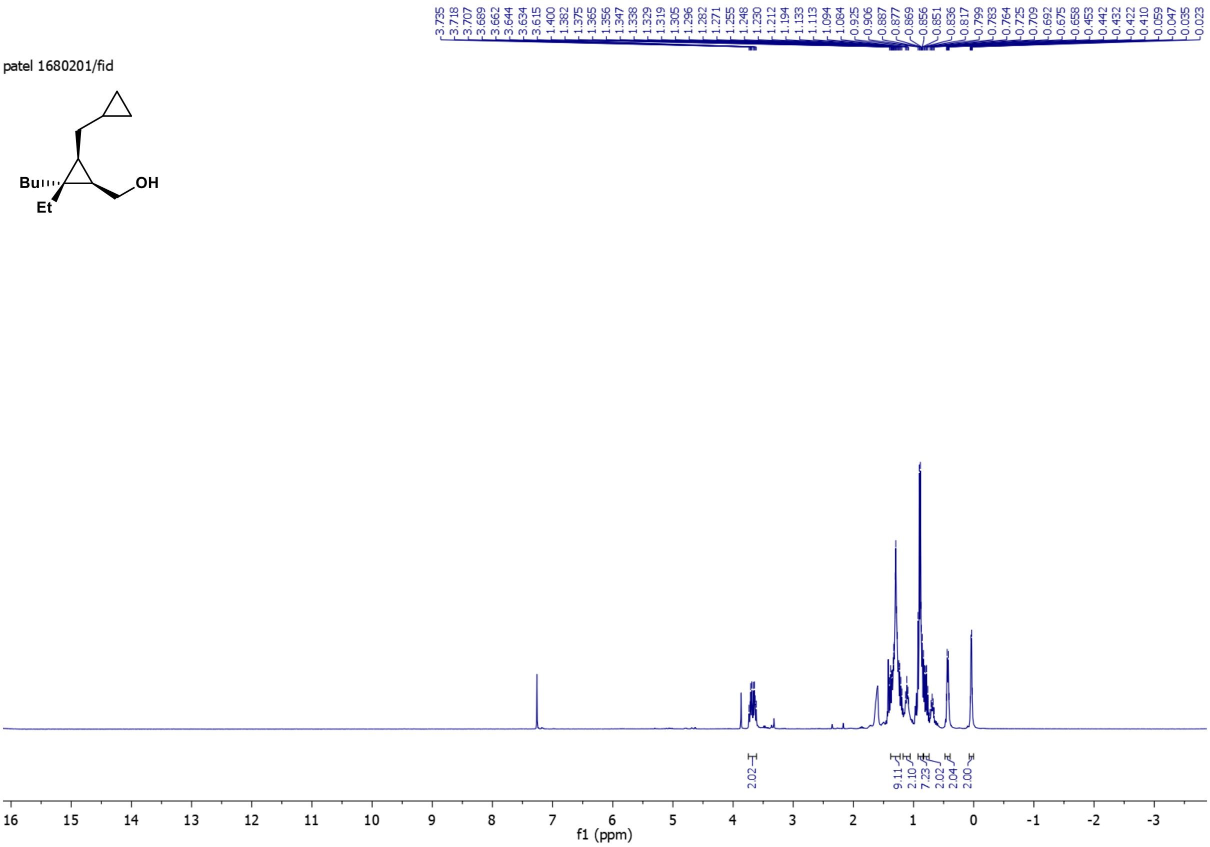
patel 1680201/fid
Bu
Et
OH
3.735
3.718
3.707
3.689
3.662
3.644
3.634
3.615
1.400
1.382
1.375
1.365
1.356
1.347
1.338
1.329
1.319
1.305
1.296
1.282
1.271
1.255
1.248
1.230
1.212
1.194
1.133
1.113
1.094
1.084
0.925
0.906
0.887
0.877
0.869
0.856
0.851
0.836
0.817
0.799
0.783
0.764
0.725
0.709
0.692
0.675
0.658
0.453
0.442
0.432
0.422
0.410
0.059
0.047
0.035
0.023
2.02
9.11
2.10
7.23
2.02
2.04
2.00
16 15 14 13 12 11 10 9 8 7 6 5 4 3 2 1 0 -1 -2 -3
f1 (ppm)

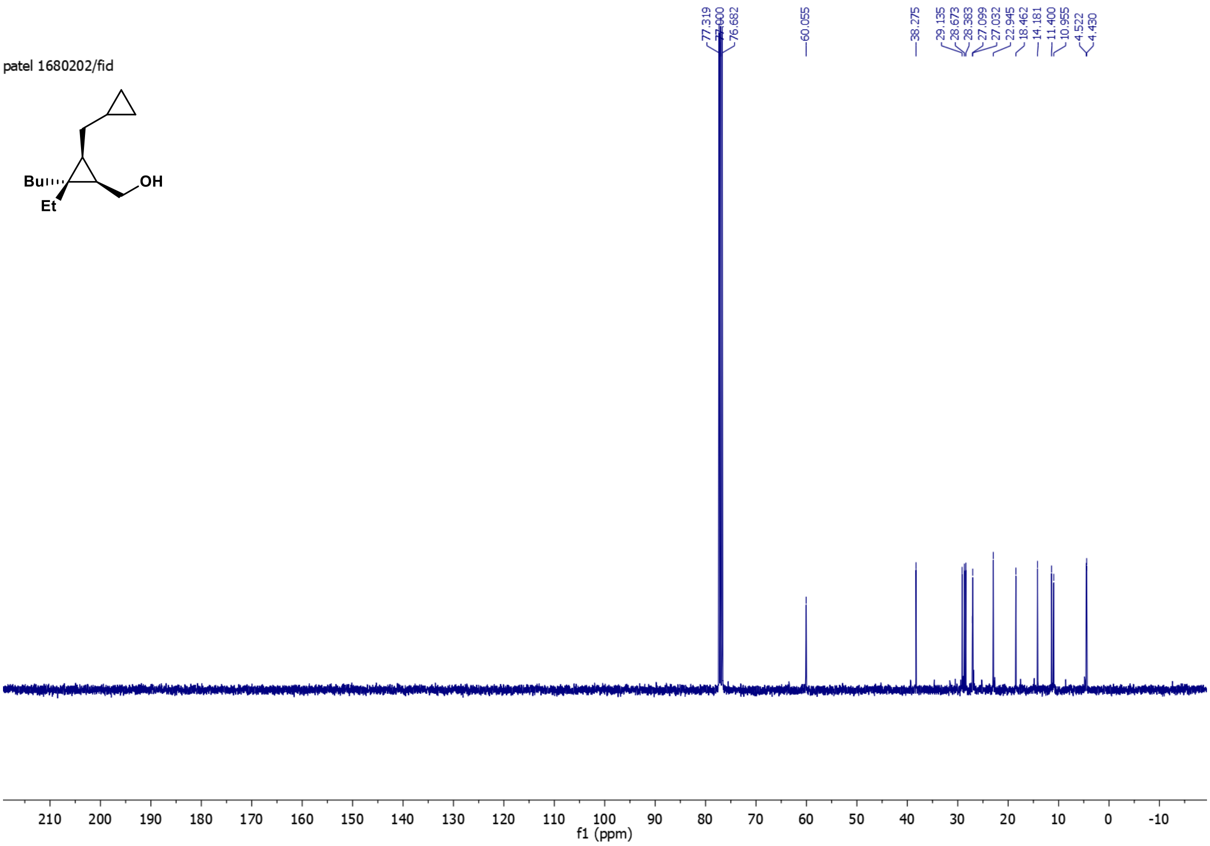
patel 1680202/fid
Bu
Et
OH
f1 (ppm)

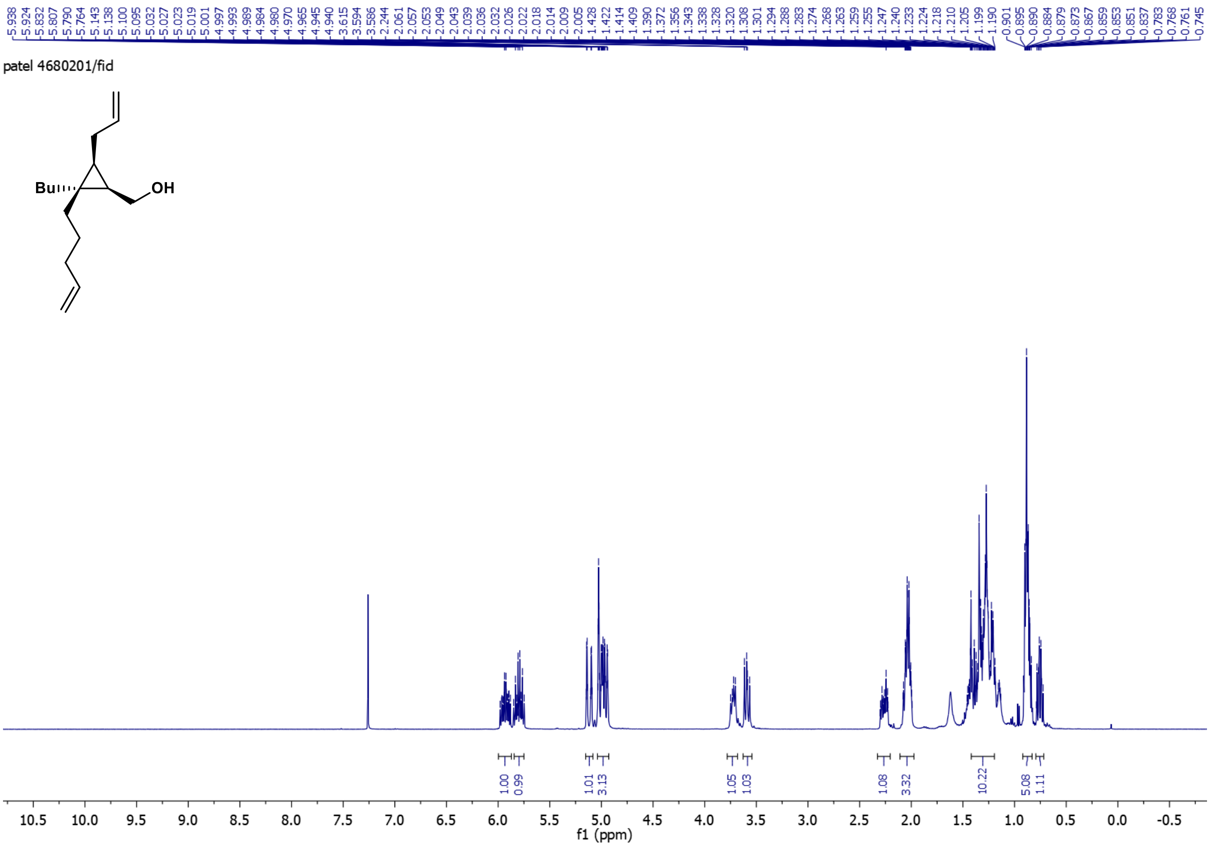
patel 4680201/fid
Bu
OH
f1 (ppm)

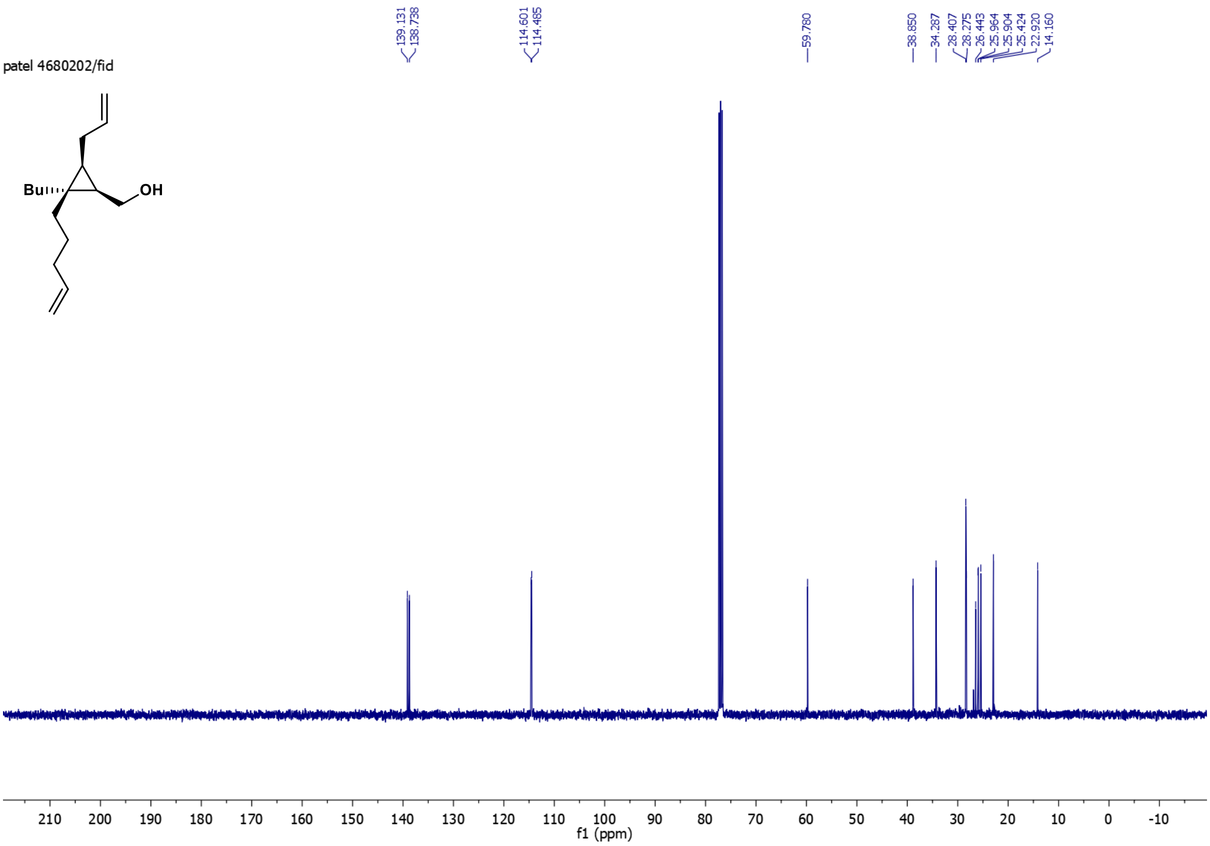
patel 4680202/fid
139.131
138.738
114.601
114.485
59.780
38.850
34.287
28.407
28.275
26.443
25.964
25.904
25.424
22.920
14.160
Bu
OH
210 200 190 180 170 160 150 140 130 120 110 100 90 80 70 60 50 40 30 20 10 0 -10
f1 (ppm)

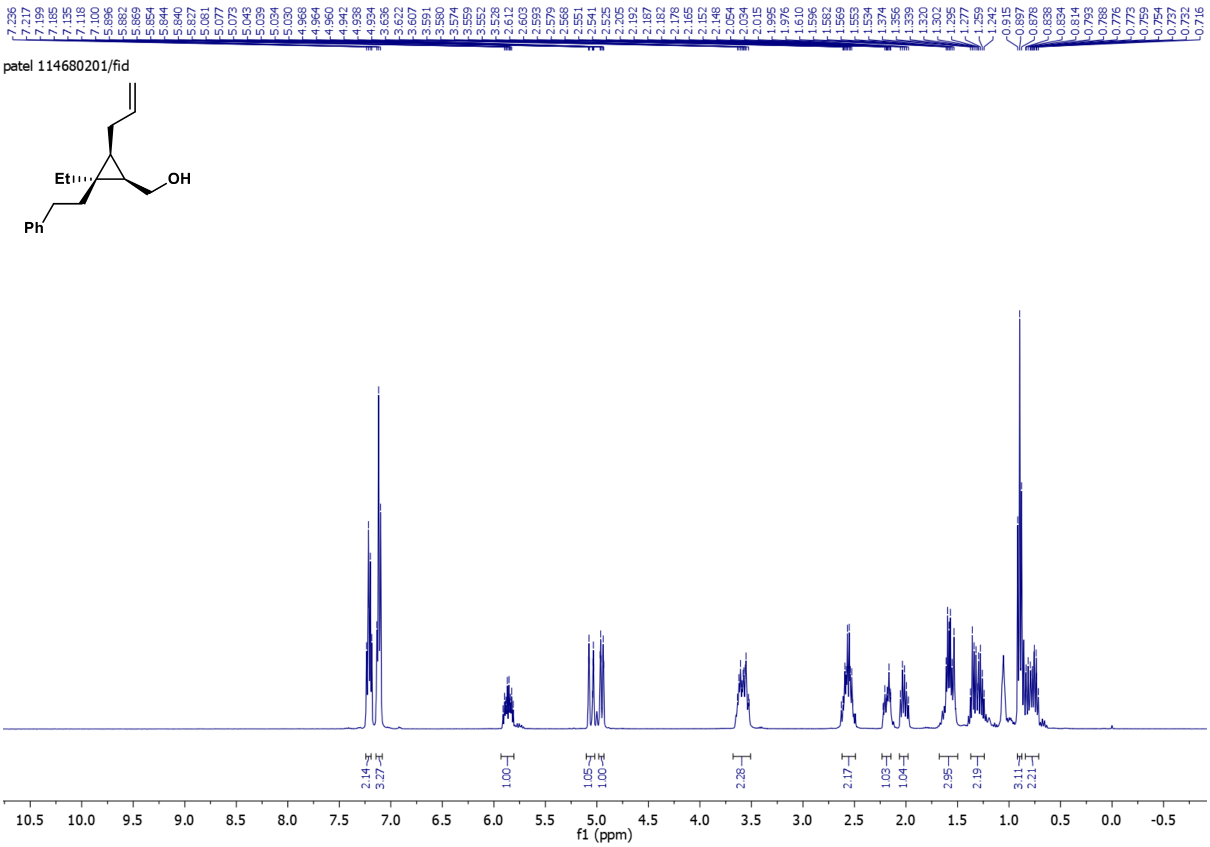
patel 114680201/fid
7.236 7.217 7.199 7.185 7.135 7.118 7.100 5.896 5.882 5.869 5.854 5.844 5.840 5.827 5.081 5.077 5.073 5.043 5.039 5.034 5.030 4.968 4.964 4.960 4.942 4.938 4.934 3.636 3.622 3.607 3.591 3.580 3.574 3.559 3.552 3.528 2.612 2.603 2.593 2.579 2.568 2.551 2.541 2.525 2.205 2.192 2.187 2.182 2.178 2.165 2.152 2.148 2.054 2.034 2.015 1.995 1.976 1.610 1.596 1.582 1.569 1.553 1.534 1.374 1.356 1.339 1.320 1.302 1.295 1.277 1.259 1.242 0.915 0.897 0.878 0.838 0.834 0.814 0.793 0.788 0.776 0.773 0.759 0.754 0.737 0.732 0.716
Et
OH
Ph
2.14 3.27 1.00 1.05 1.00 2.28 2.17 1.03 1.04 2.95 2.19 3.11 2.21
10.5 10.0 9.5 9.0 8.5 8.0 7.5 7.0 6.5 6.0 5.5 5.0 4.5 4.0 3.5 3.0 2.5 2.0 1.5 1.0 0.5 0.0 -0.5
f1 (ppm)

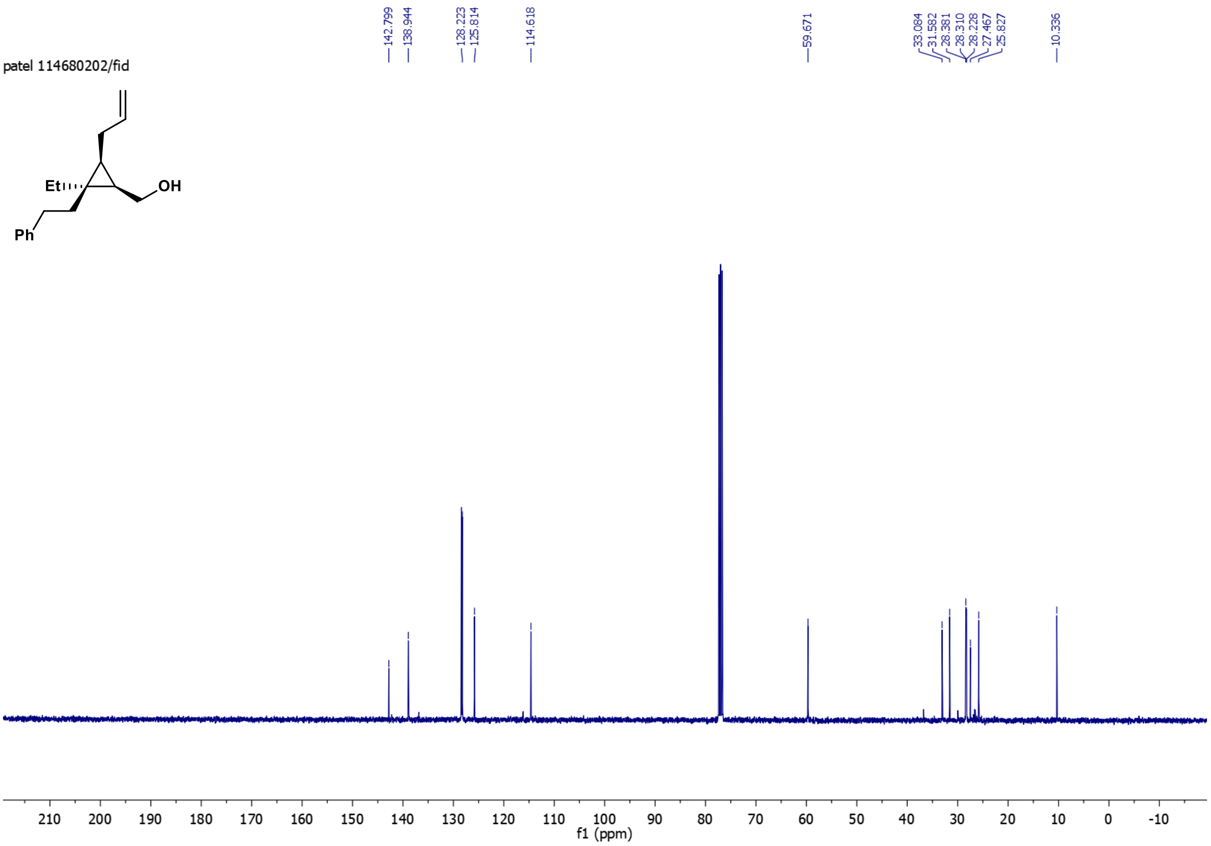
patel 114680202/fid
Et
OH
Ph
142.799
138.944
128.223
125.814
114.618
59.671
33.084
31.582
28.381
28.310
28.228
27.467
25.827
10.336
210 200 190 180 170 160 150 140 130 120 110 100 90 80 70 60 50 40 30 20 10 0 -10
f1 (ppm)

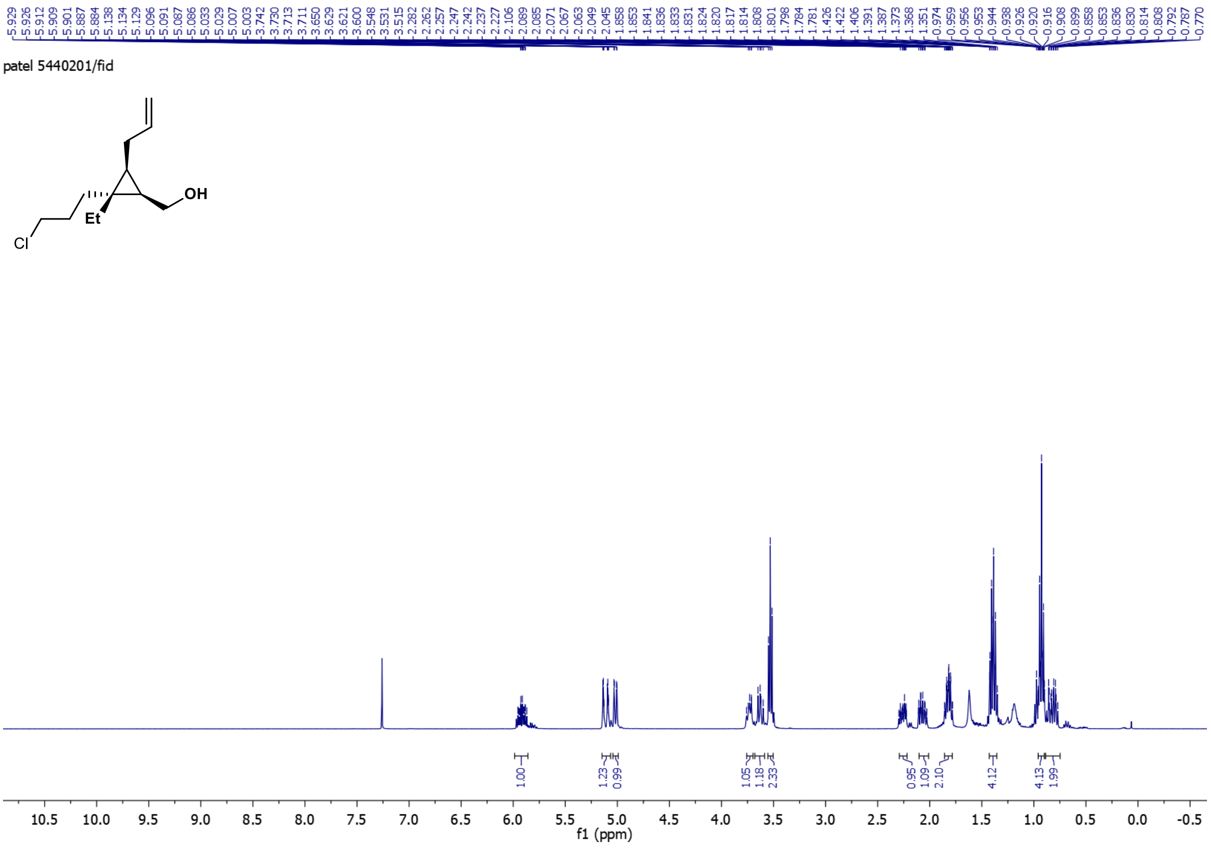
patel 5440201/fid
OH
Et
Cl
5.929 5.926 5.912 5.909 5.901 5.887 5.884 5.138 5.134 5.129 5.096 5.091 5.087 5.086 5.033 5.029 5.007 5.003 3.742 3.730 3.713 3.711 3.650 3.629 3.621 3.600 3.548 3.531 3.515 2.282 2.262 2.257 2.247 2.242 2.237 2.227 2.106 2.089 2.085 2.071 2.067 2.063 2.049 2.045 1.858 1.853 1.841 1.836 1.833 1.831 1.824 1.820 1.817 1.814 1.808 1.801 1.798 1.784 1.781 1.426 1.422 1.406 1.391 1.387 1.373 1.368 1.351 0.974 0.959 0.956 0.953 0.944 0.938 0.926 0.920 0.916 0.908 0.899 0.858 0.853 0.836 0.830 0.814 0.808 0.792 0.787 0.770
1.00
1.23
0.99
1.05
1.18
2.33
0.95
1.09
2.10
4.12
4.13
1.99
10.5 10.0 9.5 9.0 8.5 8.0 7.5 7.0 6.5 6.0 5.5 5.0 4.5 4.0 3.5 3.0 2.5 2.0 1.5 1.0 0.5 0.0 -0.5
f1 (ppm)

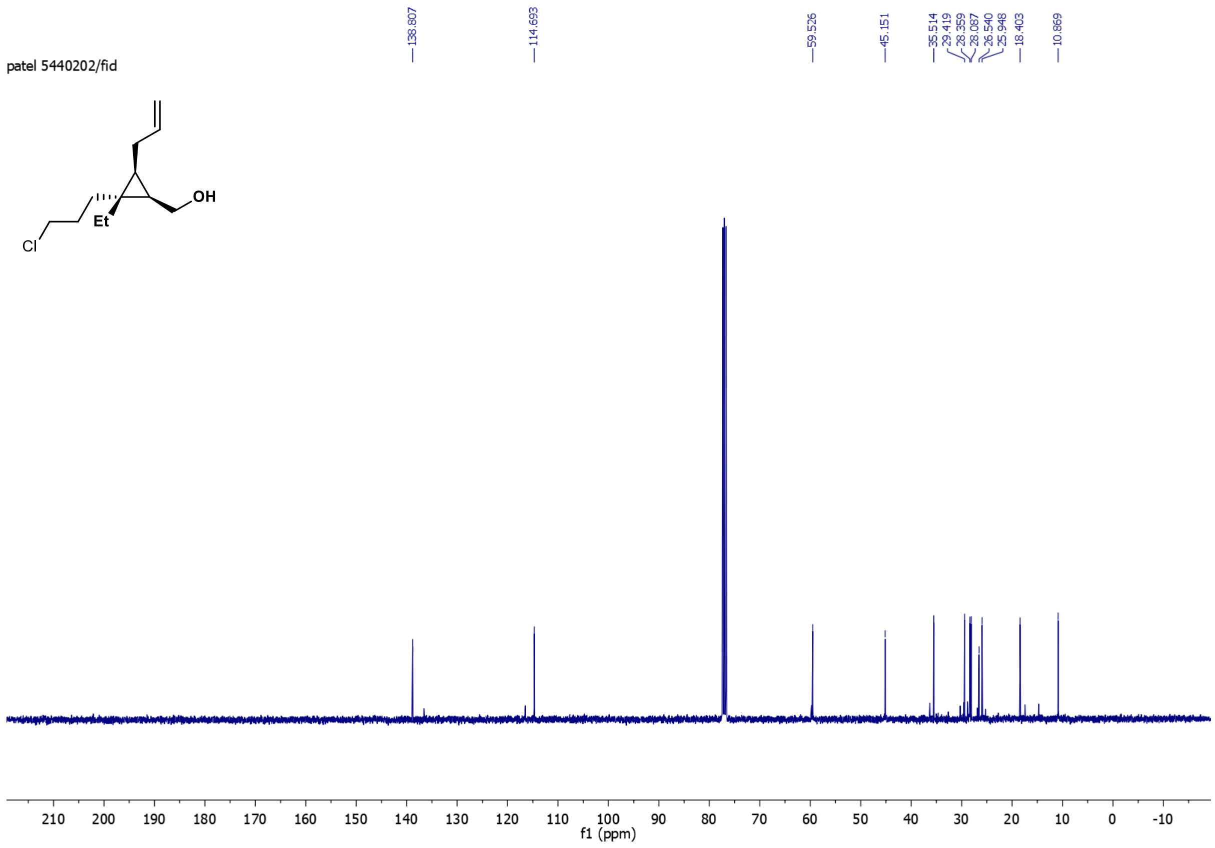
patel 5440202/fid
138.807
114.693
59.526
45.151
35.514
29.419
28.359
28.087
26.540
25.948
18.403
10.869
OH
Et
Cl
f1 (ppm)

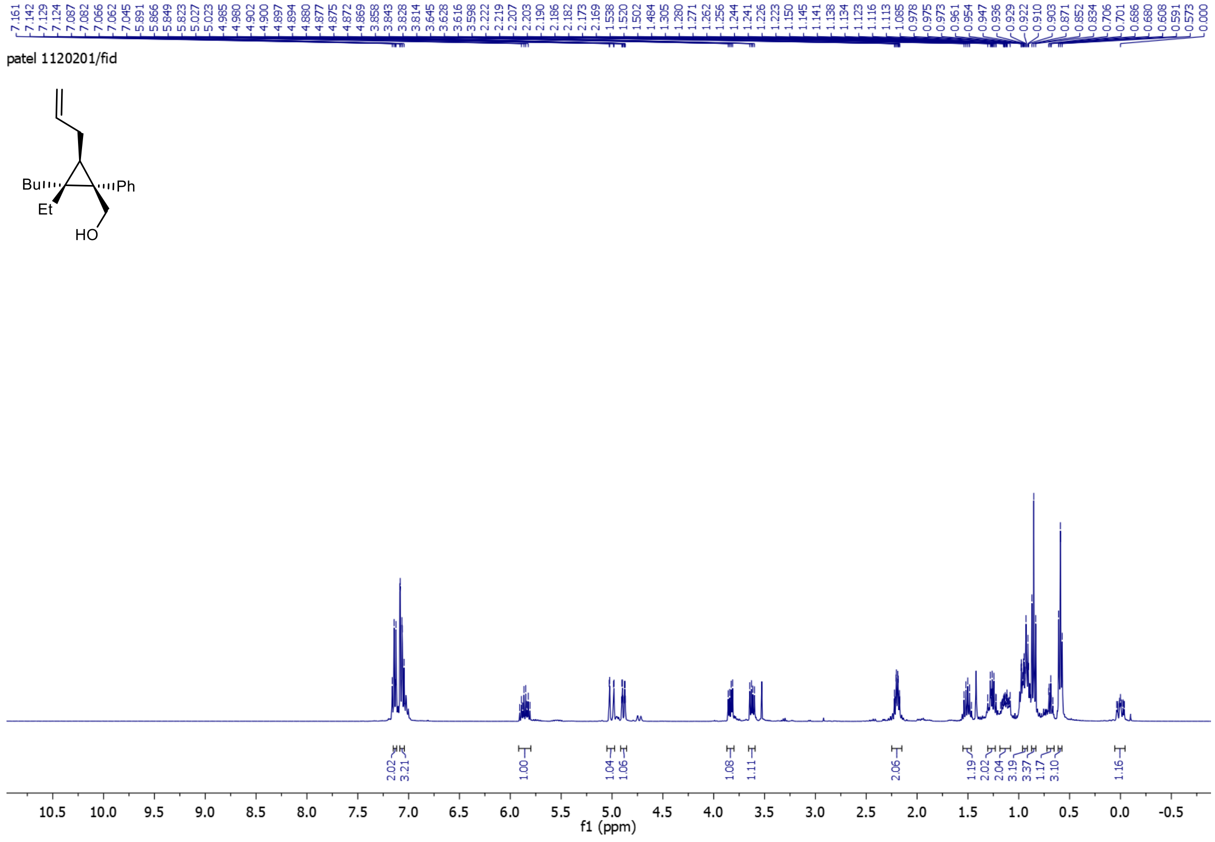
patel 1120201/fid
Bu
Ph
Et
HO
f1 (ppm)

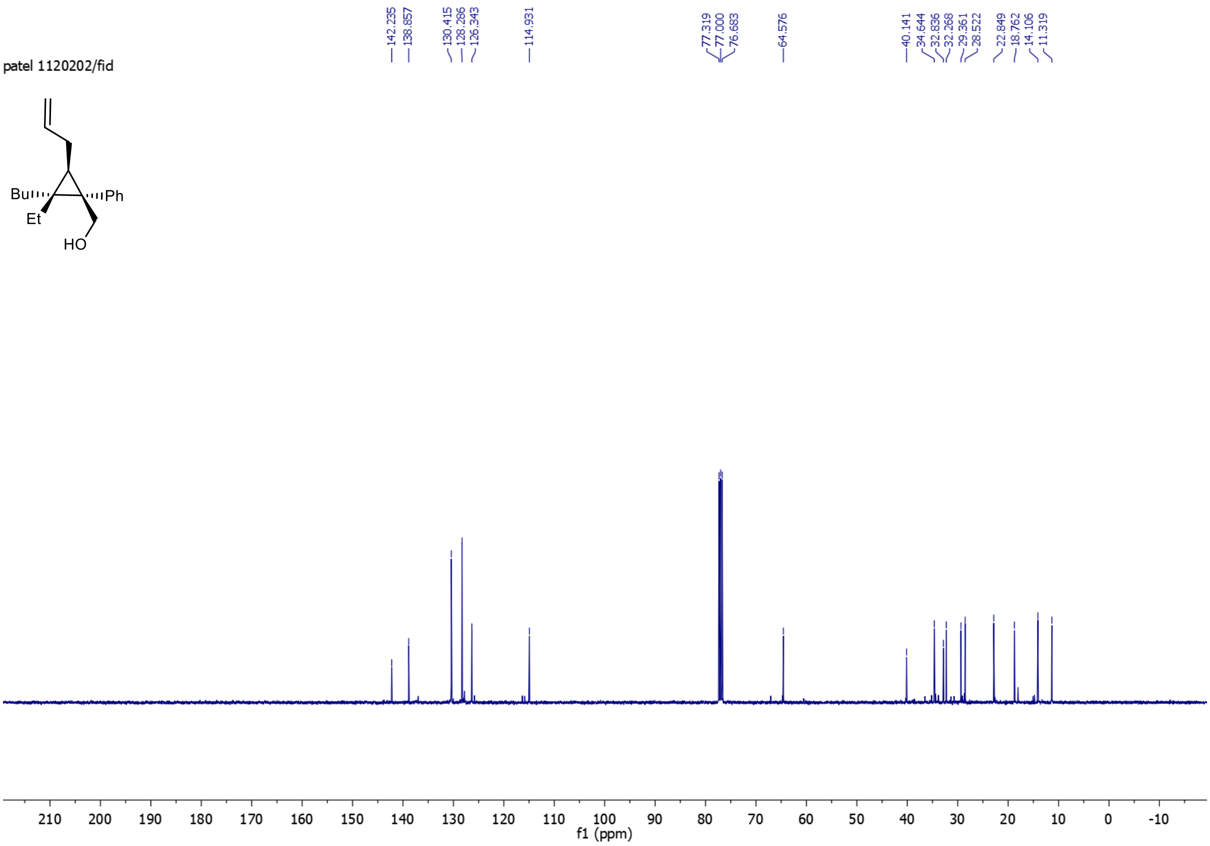
patel 1120202/fid
Bu
Et
Ph
HO
142.235
138.857
130.415
128.286
126.343
114.931
77.319
77.000
76.683
64.576
40.141
34.644
32.836
32.268
29.361
28.522
22.849
18.762
14.106
11.319
f1 (ppm)

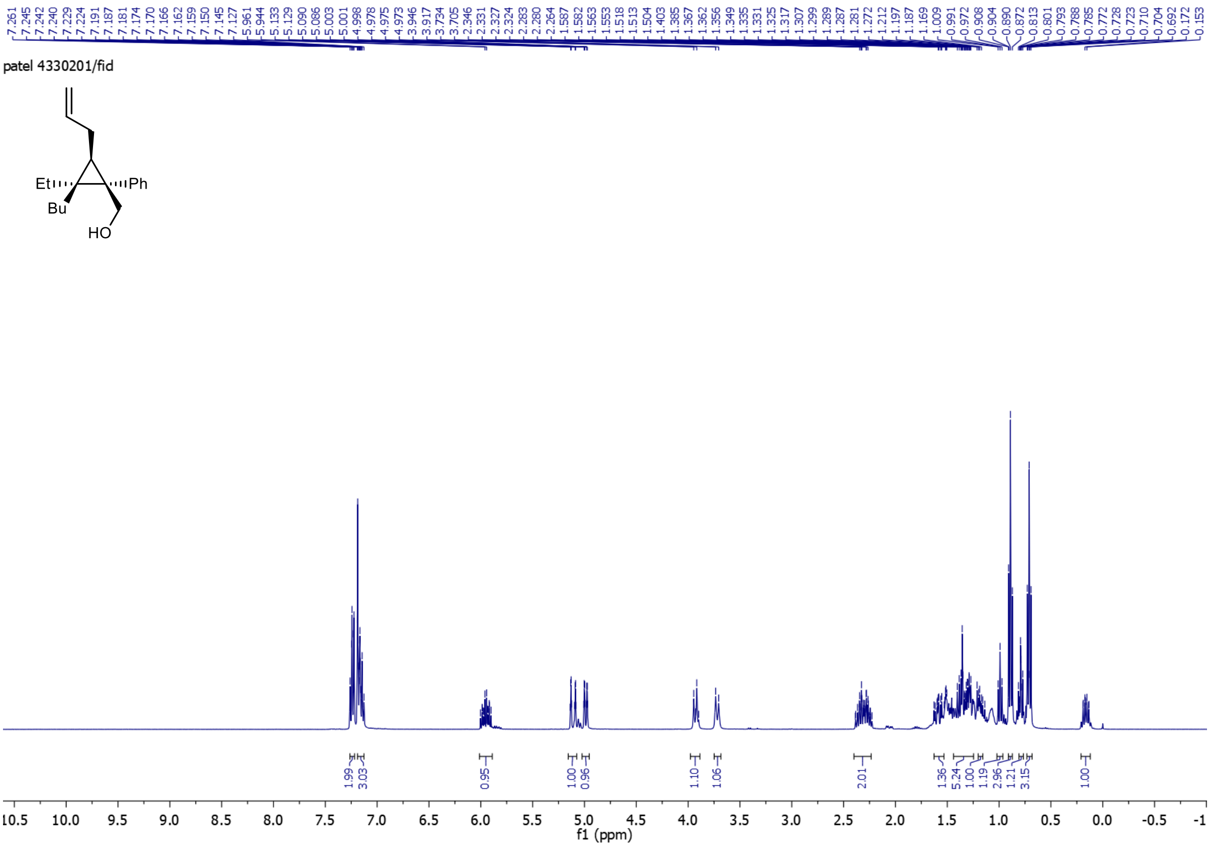
patel 4330201/fid
Et
Bu
Ph
HO
1.99
3.03
0.95
1.00
0.96
1.10
1.06
2.01
1.36
5.24
1.00
1.19
2.96
1.21
3.15
1.00
f1 (ppm)

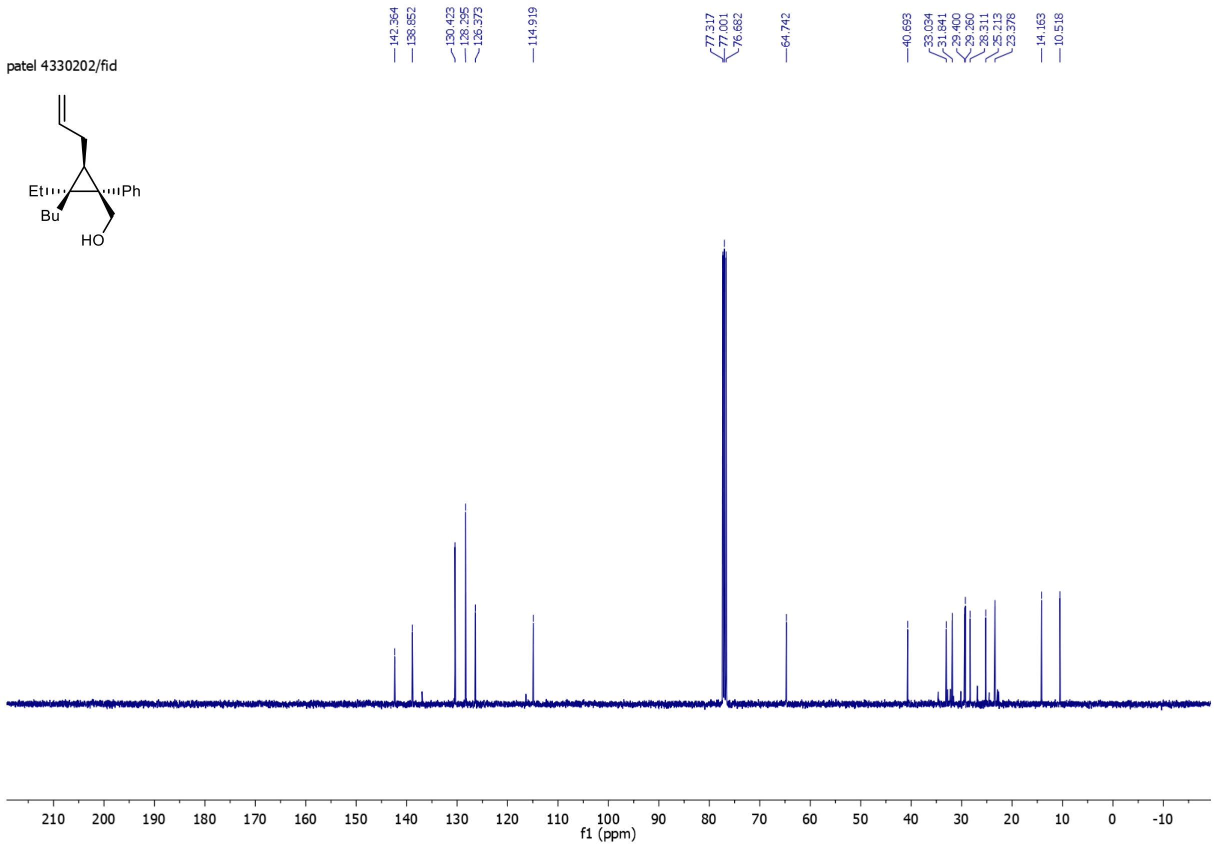
patel 4330202/fid
142.364
138.852
130.423
128.295
126.373
114.919
77.317
77.001
76.682
64.742
40.693
33.034
31.841
29.400
29.260
28.311
25.213
23.378
14.163
10.518
Et
Ph
Bu
HO
210
200
190
180
170
160
150
140
130
120
110
100
90
80
70
60
50
40
30
20
10
0
-10
f1 (ppm)

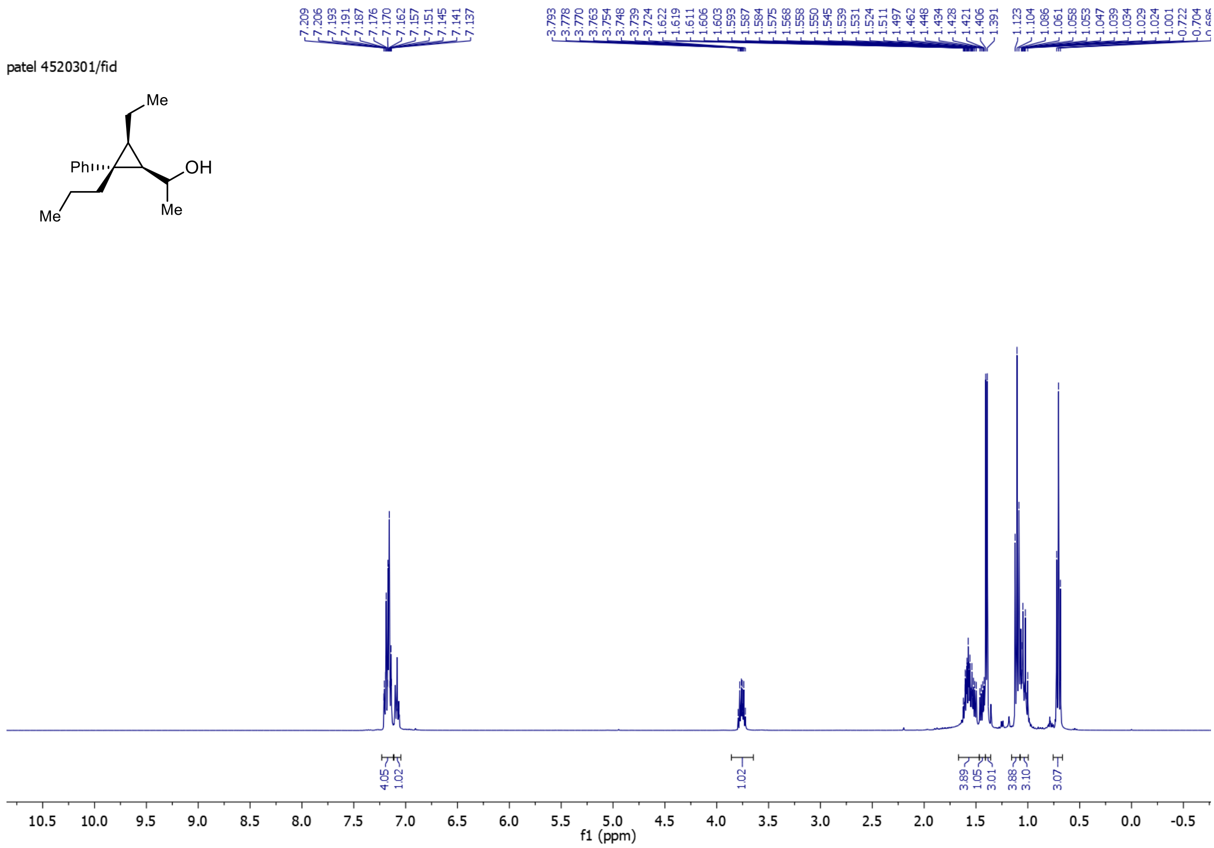
patel 4520301/fid
7.209
7.206
7.193
7.191
7.187
7.176
7.170
7.162
7.157
7.151
7.145
7.141
7.137
3.793
3.778
3.770
3.763
3.754
3.748
3.739
3.724
1.622
1.619
1.611
1.606
1.603
1.593
1.587
1.584
1.575
1.568
1.558
1.550
1.545
1.539
1.531
1.524
1.511
1.497
1.462
1.448
1.434
1.428
1.421
1.406
1.391
1.123
1.104
1.086
1.061
1.058
1.053
1.047
1.039
1.034
1.029
1.024
1.001
0.722
0.704
Me
Ph
OH
Me
Me
4.05
1.02
1.02
3.89
1.05
3.01
3.88
3.10
3.07
10.5
10.0
9.5
9.0
8.5
8.0
7.5
7.0
6.5
6.0
5.5
5.0
4.5
4.0
3.5
3.0
2.5
2.0
1.5
1.0
0.5
0.0
-0.5
f1 (ppm)

patel 4520302/fid

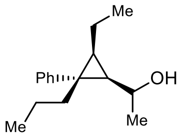

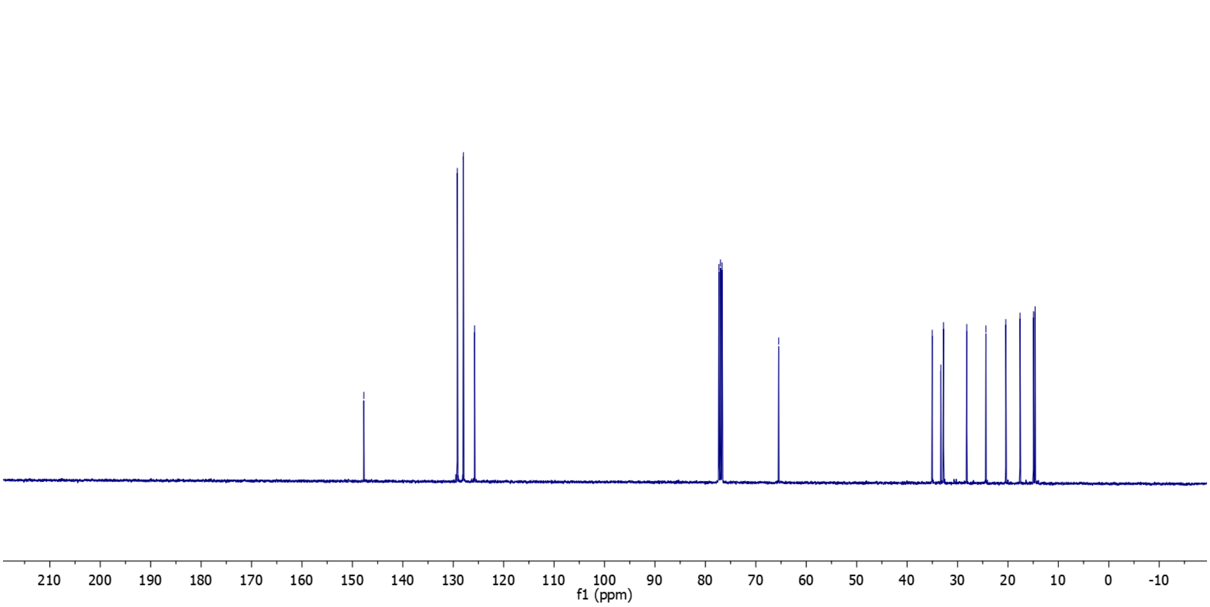

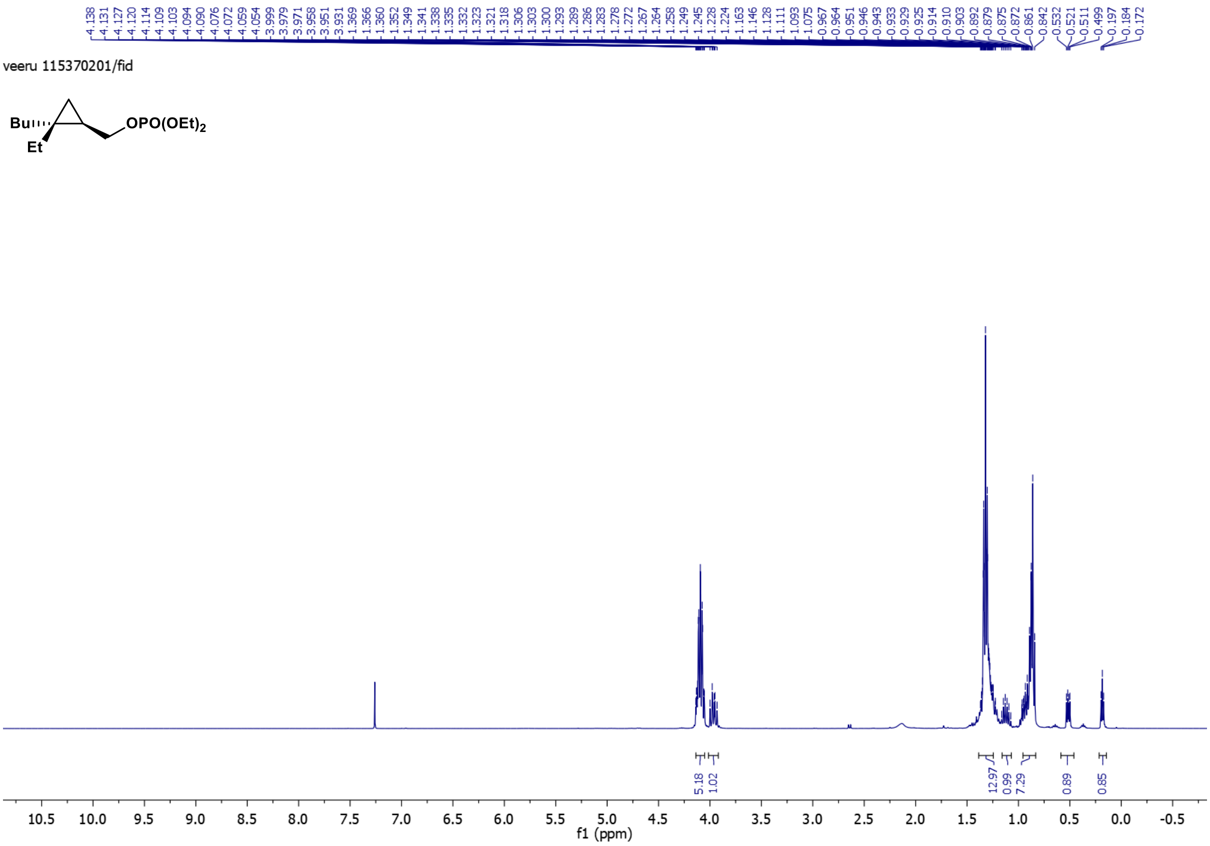
4.138 4.131 4.127 4.120 4.114 4.109 4.103 4.094 4.090 4.076 4.072 4.059 4.054 3.999 3.979 3.971 3.958 3.951 3.931 1.369 1.366 1.360 1.352 1.349 1.341 1.338 1.335 1.332 1.323 1.321 1.318 1.306 1.303 1.300 1.293 1.289 1.286 1.283 1.278 1.272 1.267 1.264 1.258 1.249 1.245 1.228 1.224 1.163 1.146 1.128 1.111 1.093 1.075 0.967 0.964 0.951 0.946 0.943 0.933 0.929 0.925 0.914 0.910 0.903 0.892 0.879 0.875 0.872 0.861 0.842 0.532 0.521 0.511 0.499 0.197 0.184 0.172
veeru 115370201/fid
Bu
Et
OPO(OEt)2
5.18
1.02
12.97
0.99
7.29
0.89
0.85
10.5 10.0 9.5 9.0 8.5 8.0 7.5 7.0 6.5 6.0 5.5 5.0 4.5 4.0 3.5 3.0 2.5 2.0 1.5 1.0 0.5 0.0 -0.5
f1 (ppm)

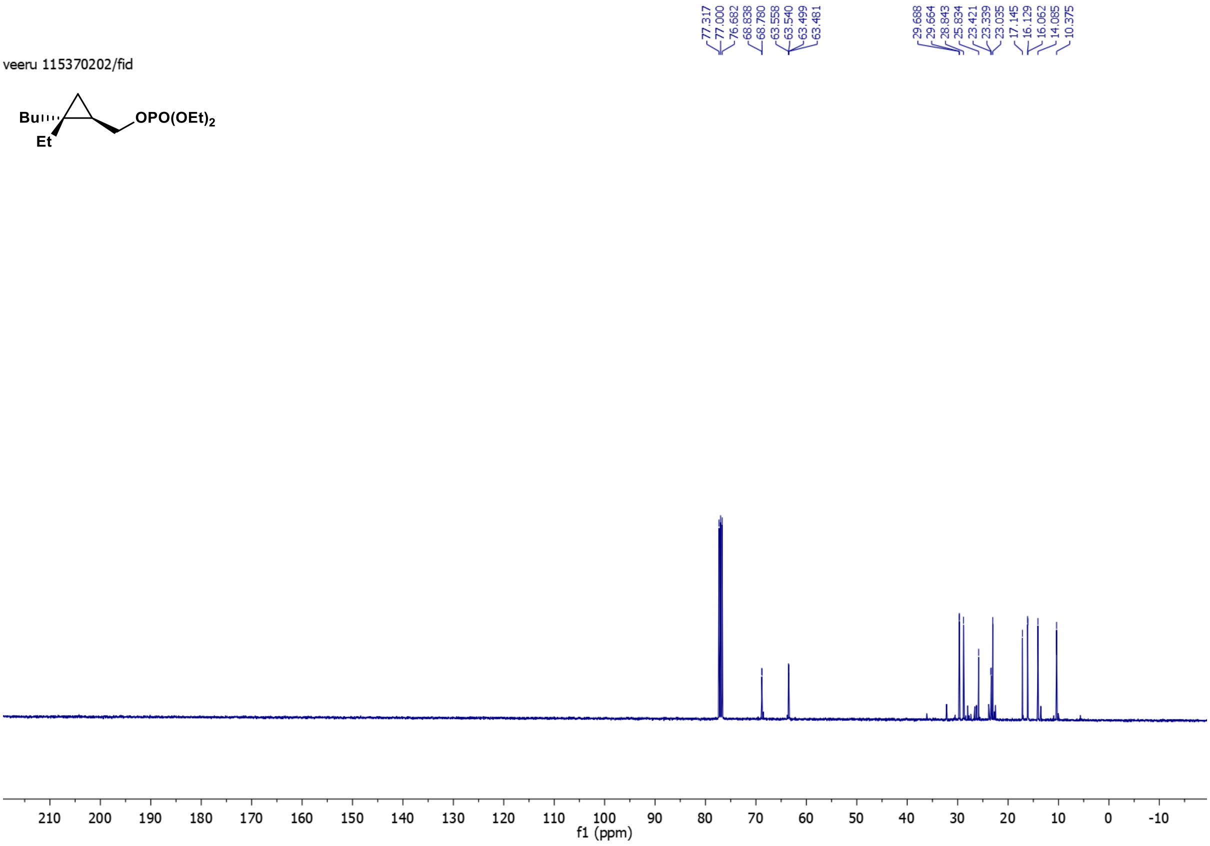
77.317 77.000 76.682 68.838 68.780 63.558 63.540 63.499 63.481
29.688 29.664 28.843 25.834 23.421 23.339 23.035 17.145 16.129 16.062 14.085 10.375
veeru 115370202/fid
Bu
Et
OPO(OEt)2
210 200 190 180 170 160 150 140 130 120 110 100 90 80 70 60 50 40 30 20 10 0 -10
f1 (ppm)

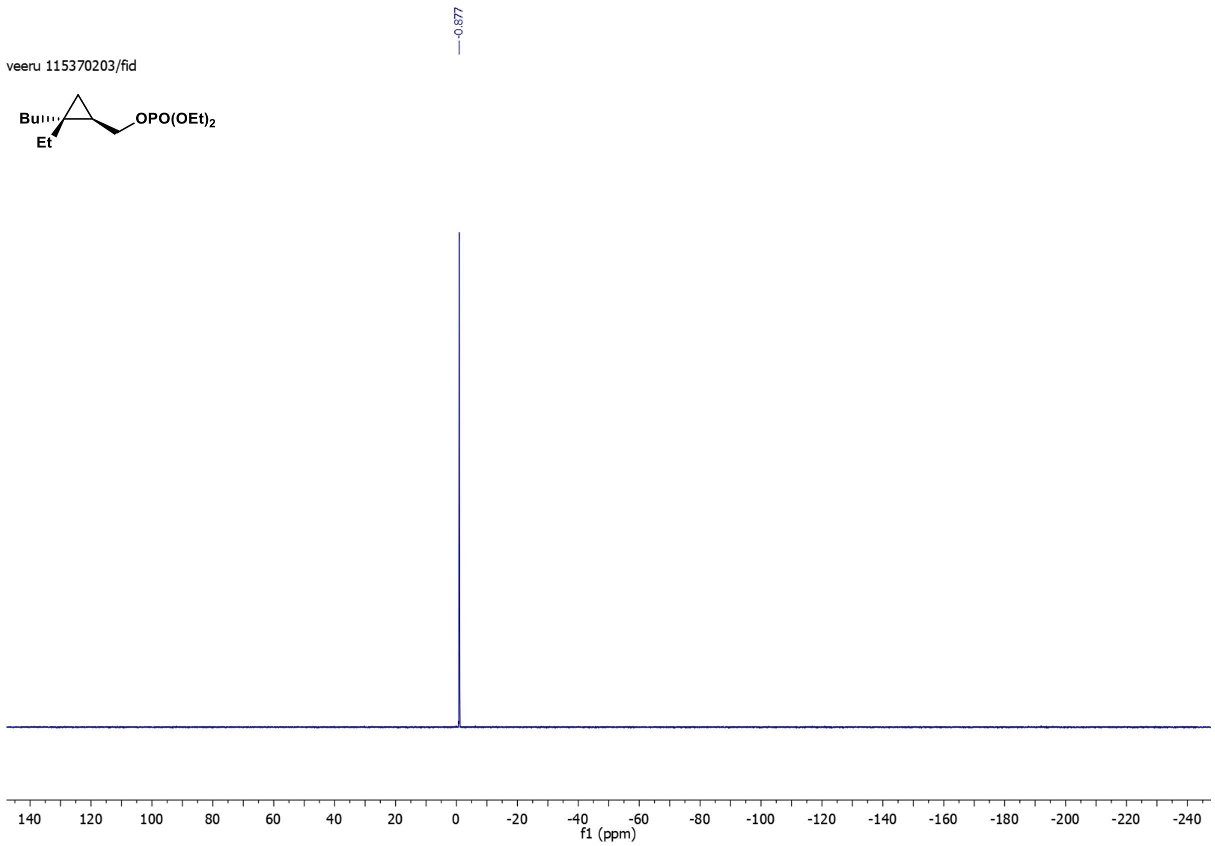
—0.877
veeru 115370203/fid
Bu
Et
OPO(OEt)2
140 120 100 80 60 40 20 0 -20 -40 -60 -80 -100 -120 -140 -160 -180 -200 -220 -240
f1 (ppm)

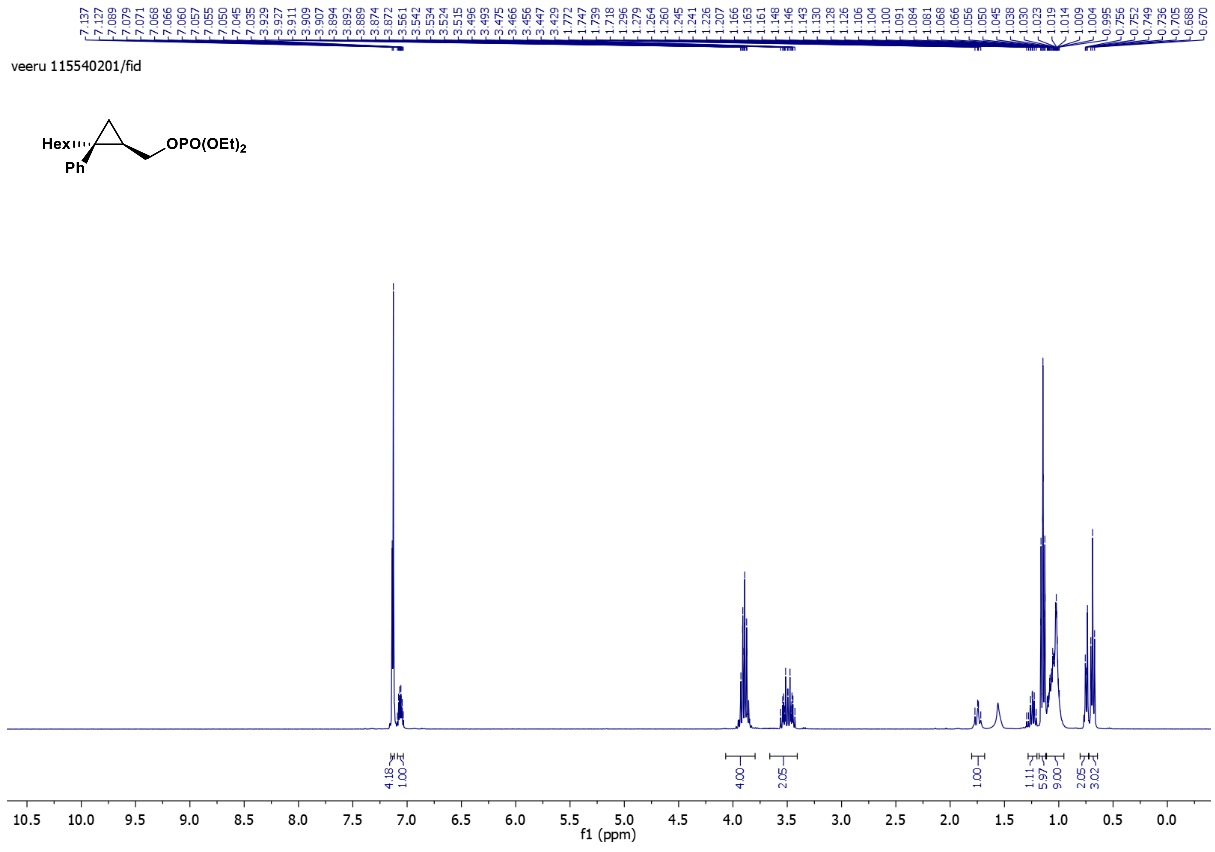
veeru 115540201/fid
Hex
Ph
OPO(OEt)2
4.18
1.00
4.00
2.05
1.00
1.11
5.97
9.00
2.05
3.02
10.5 10.0 9.5 9.0 8.5 8.0 7.5 7.0 6.5 6.0 5.5 5.0 4.5 4.0 3.5 3.0 2.5 2.0 1.5 1.0 0.5 0.0
f1 (ppm)

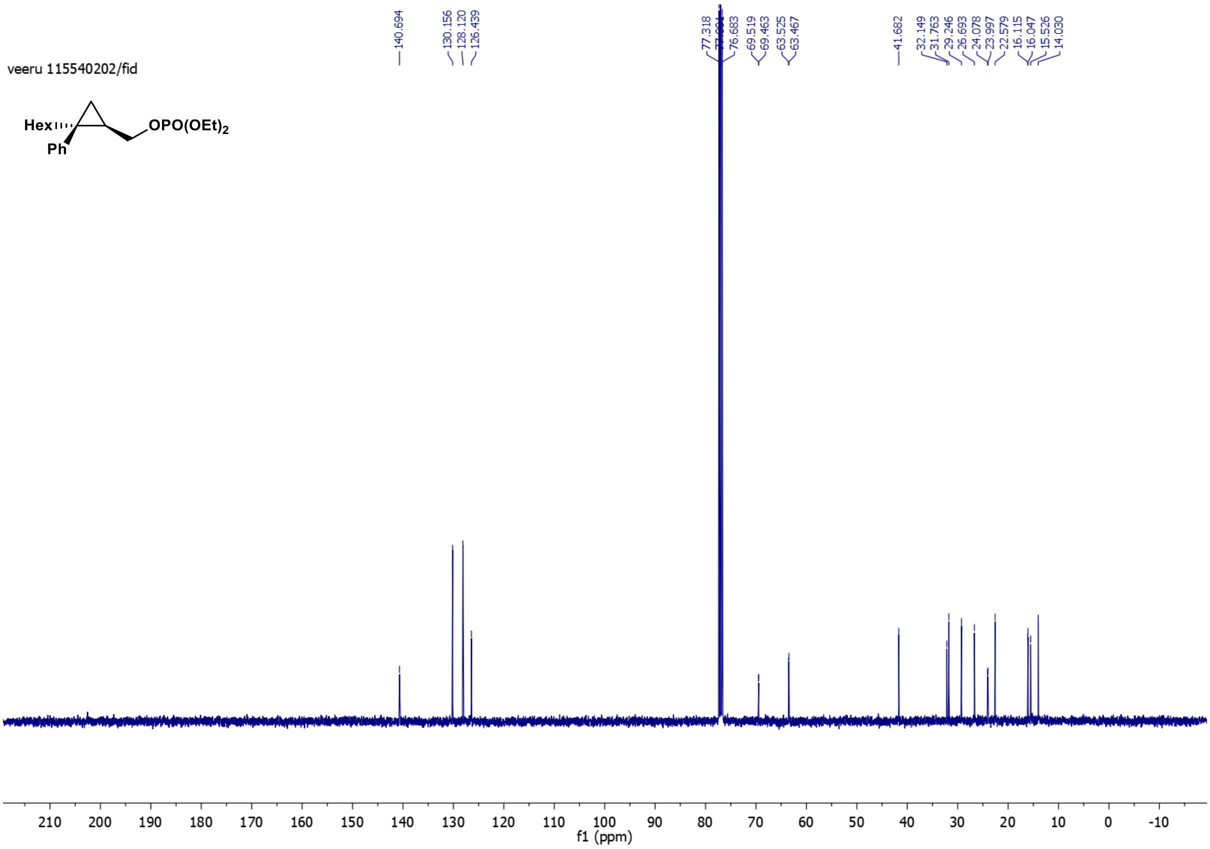
veeru 115540202/fid
Hex
OPO(OEt)2
Ph
140.694
130.156
128.120
126.439
77.318
76.683
69.519
69.463
63.525
63.467
41.682
32.149
31.763
29.246
26.693
24.078
23.997
22.579
16.115
16.047
15.526
14.030
f1 (ppm)

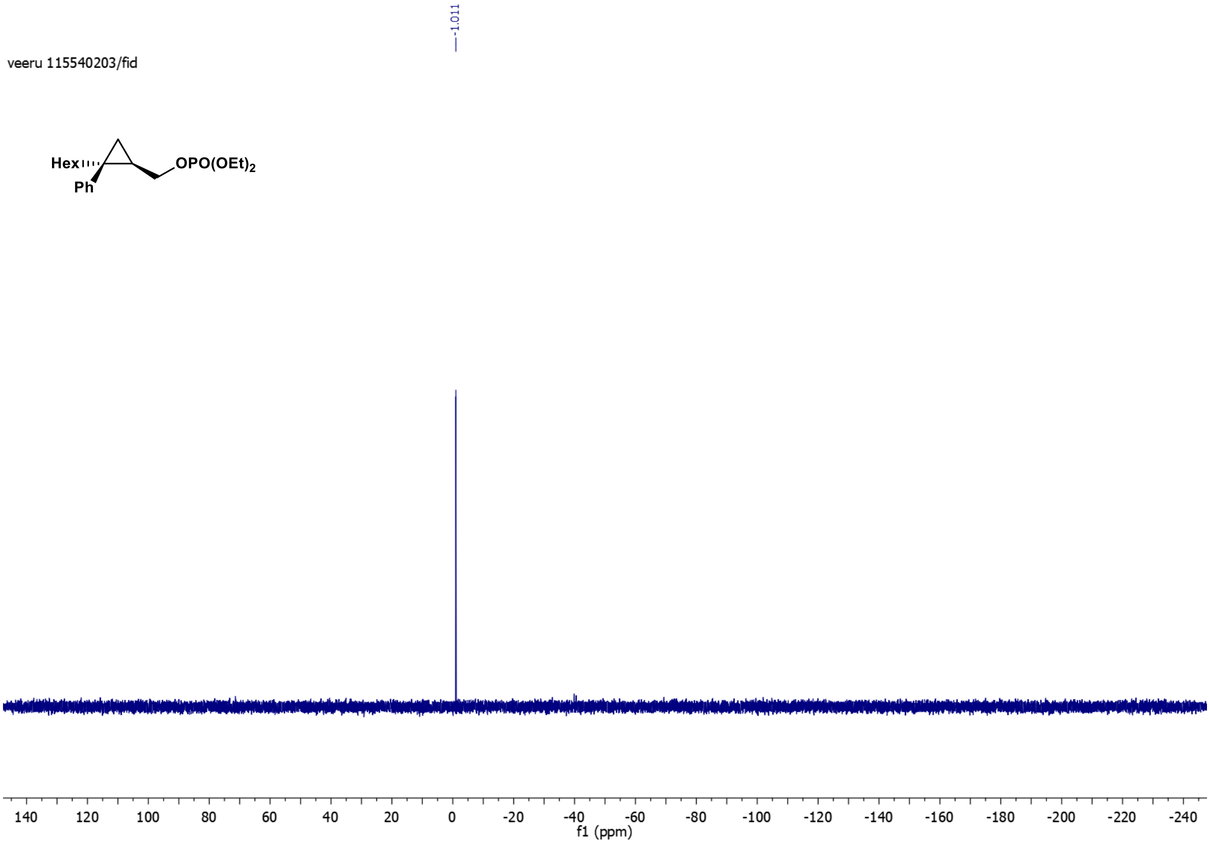
veeru 115540203/fid
Hex
OPO(OEt)2
Ph
-1.011
f1 (ppm)

patel 2080201/fid

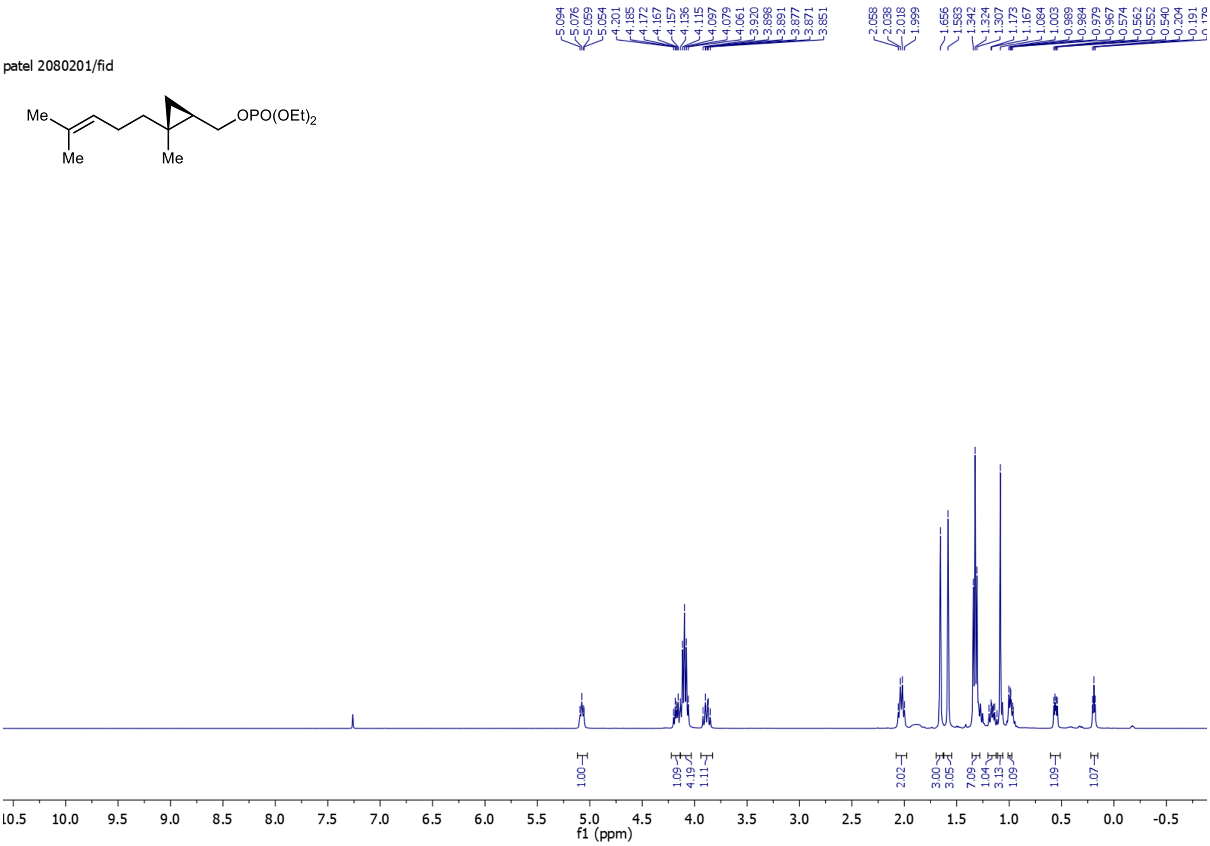

patel 2080202/fid

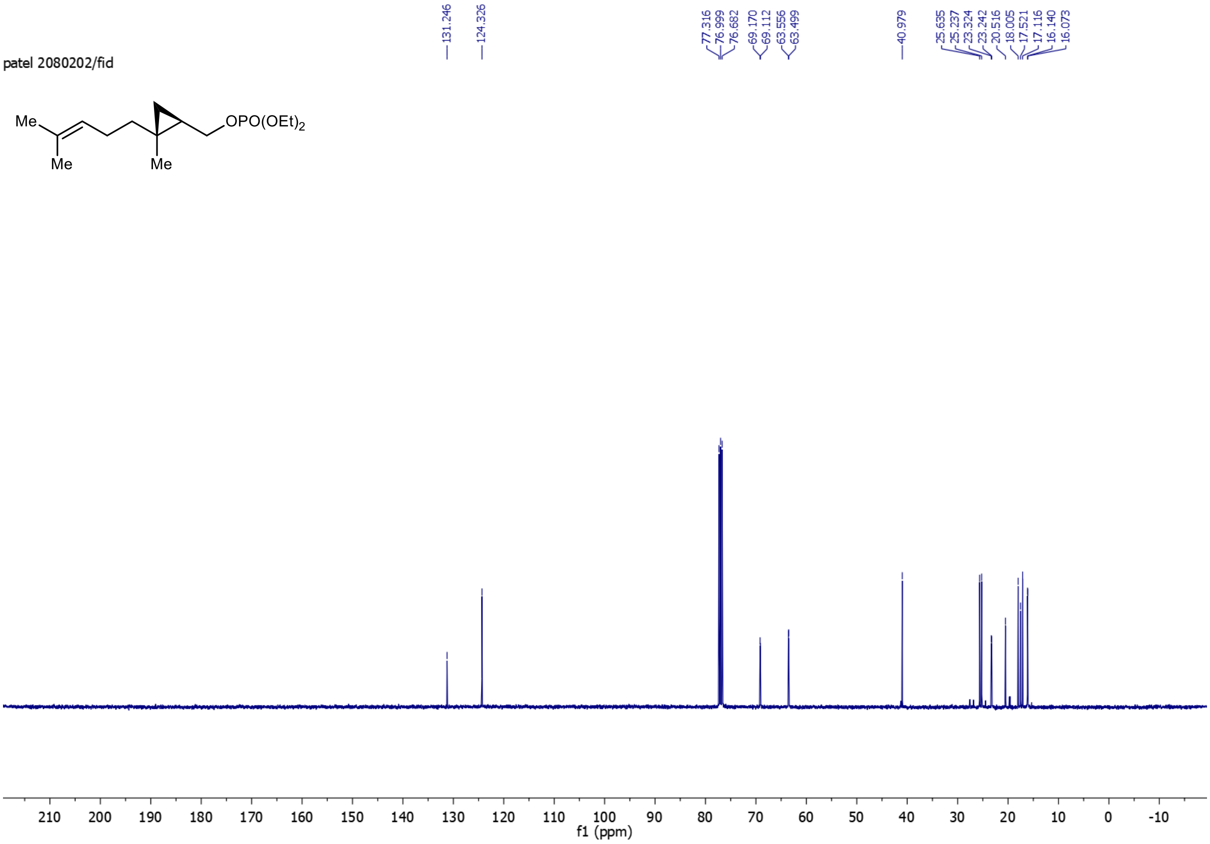

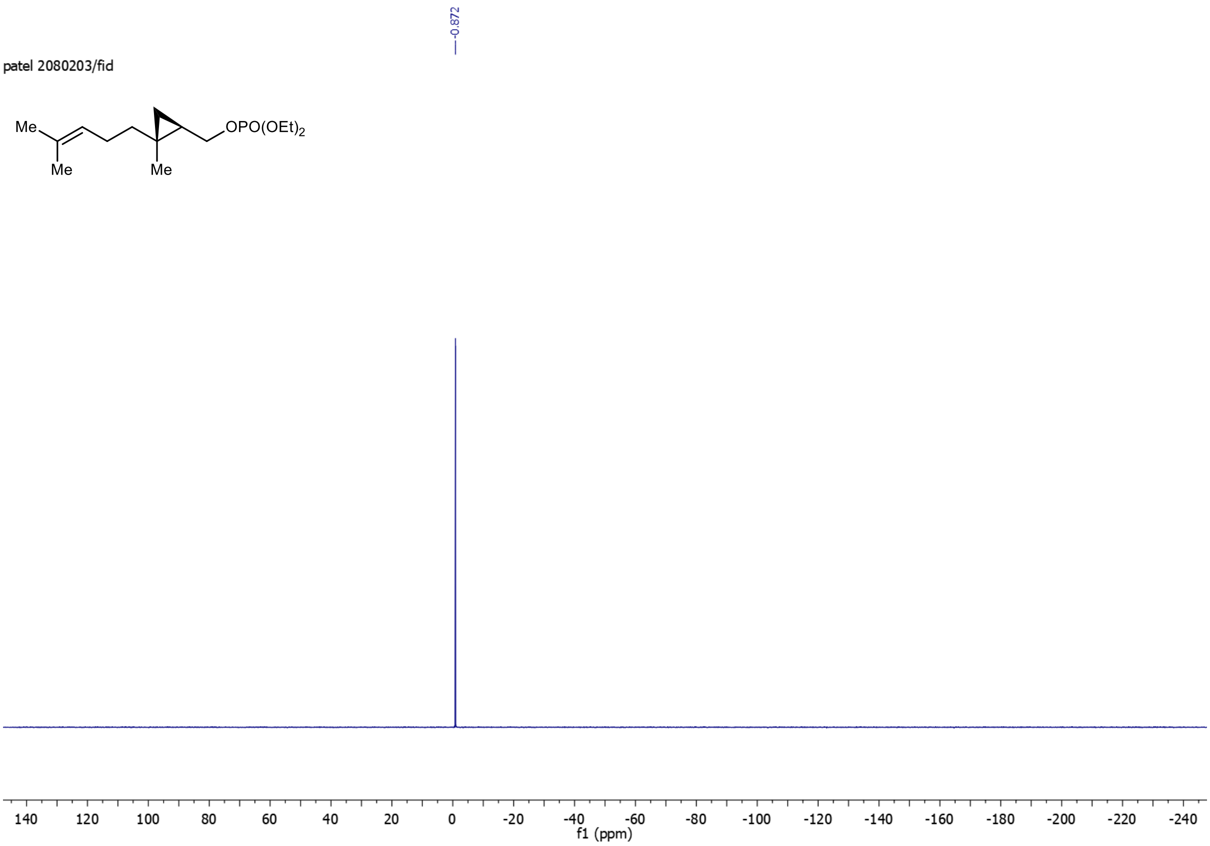
patel 2080203/fid
-0.872
Me
Me
Me
OPO(OEt)2
140 120 100 80 60 40 20 0 -20 -40 -60 -80 -100 -120 -140 -160 -180 -200 -220 -240
f1 (ppm)

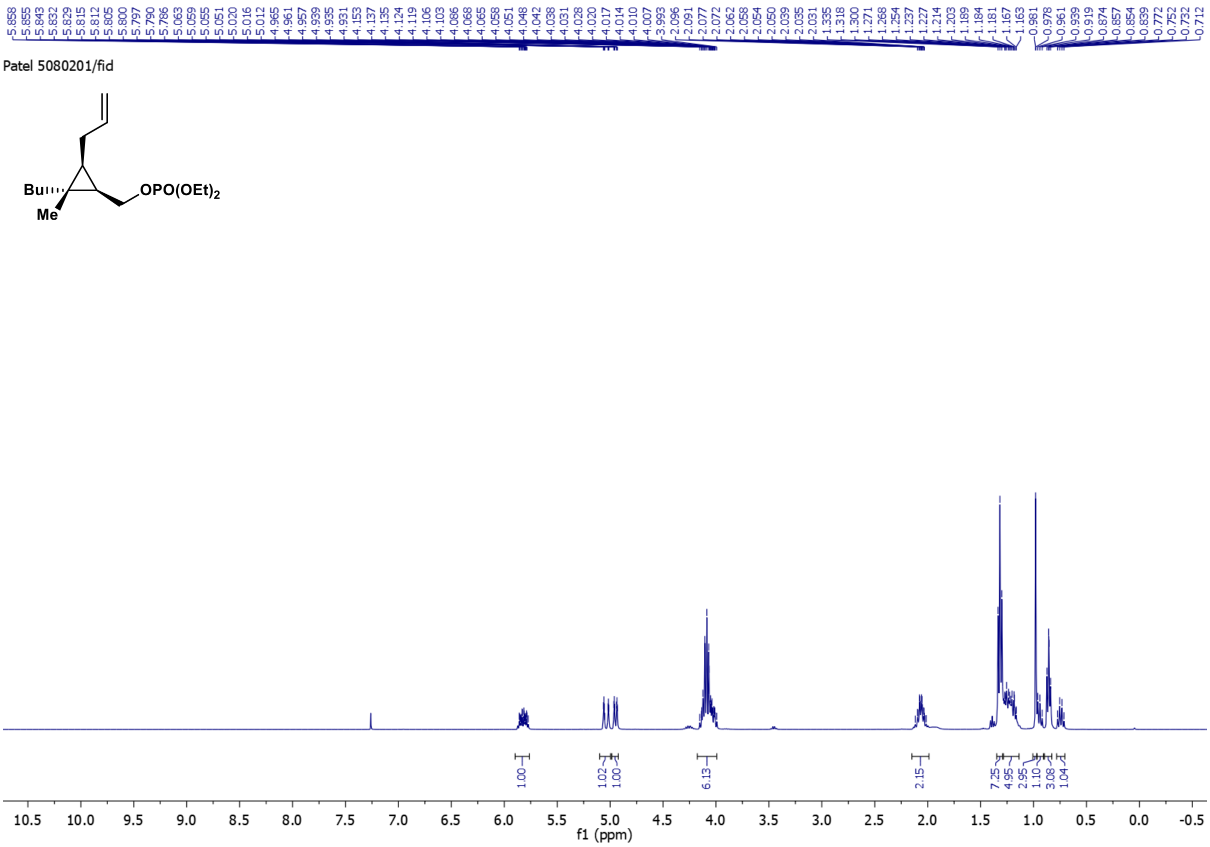
Patel 5080201/fid
5.858 5.855 5.843 5.832 5.829 5.815 5.812 5.805 5.800 5.797 5.790 5.786 5.063 5.059 5.055 5.051 5.020 5.016 5.012 4.965 4.961 4.957 4.939 4.935 4.931 4.153 4.137 4.135 4.124 4.119 4.106 4.103 4.086 4.068 4.065 4.058 4.051 4.048 4.042 4.038 4.031 4.028 4.020 4.017 4.014 4.010 4.007 3.993 2.096 2.091 2.077 2.072 2.062 2.058 2.054 2.050 2.039 2.035 2.031 1.335 1.318 1.300 1.271 1.268 1.254 1.237 1.227 1.214 1.203 1.189 1.184 1.181 1.167 1.163 0.981 0.978 0.961 0.939 0.919 0.874 0.857 0.854 0.839 0.772 0.752 0.732 0.712
Bu
Me
OPO(OEt)2
1.00
1.02
1.00
6.13
2.15
7.25
4.95
2.95
1.10
3.08
1.04
10.5 10.0 9.5 9.0 8.5 8.0 7.5 7.0 6.5 6.0 5.5 5.0 4.5 4.0 3.5 3.0 2.5 2.0 1.5 1.0 0.5 0.0 -0.5
f1 (ppm)

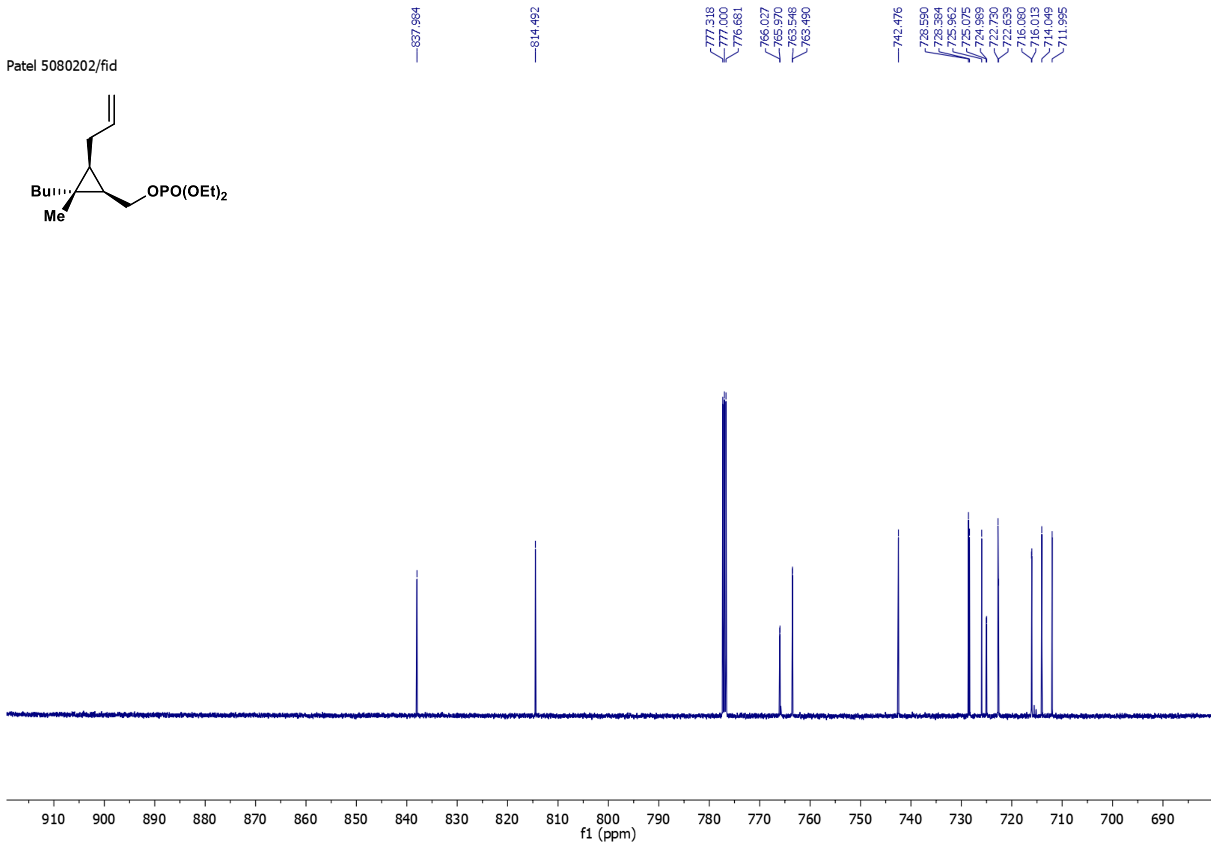
Patel 5080202/fid
Bu
Me
OPO(OEt)2
837.984
814.492
777.318
777.000
776.681
766.027
765.970
763.548
763.490
742.476
728.590
728.384
725.962
725.075
724.989
722.730
722.639
716.080
716.013
714.049
711.995
910 900 890 880 870 860 850 840 830 820 810 800 790 780 770 760 750 740 730 720 710 700 690
f1 (ppm)

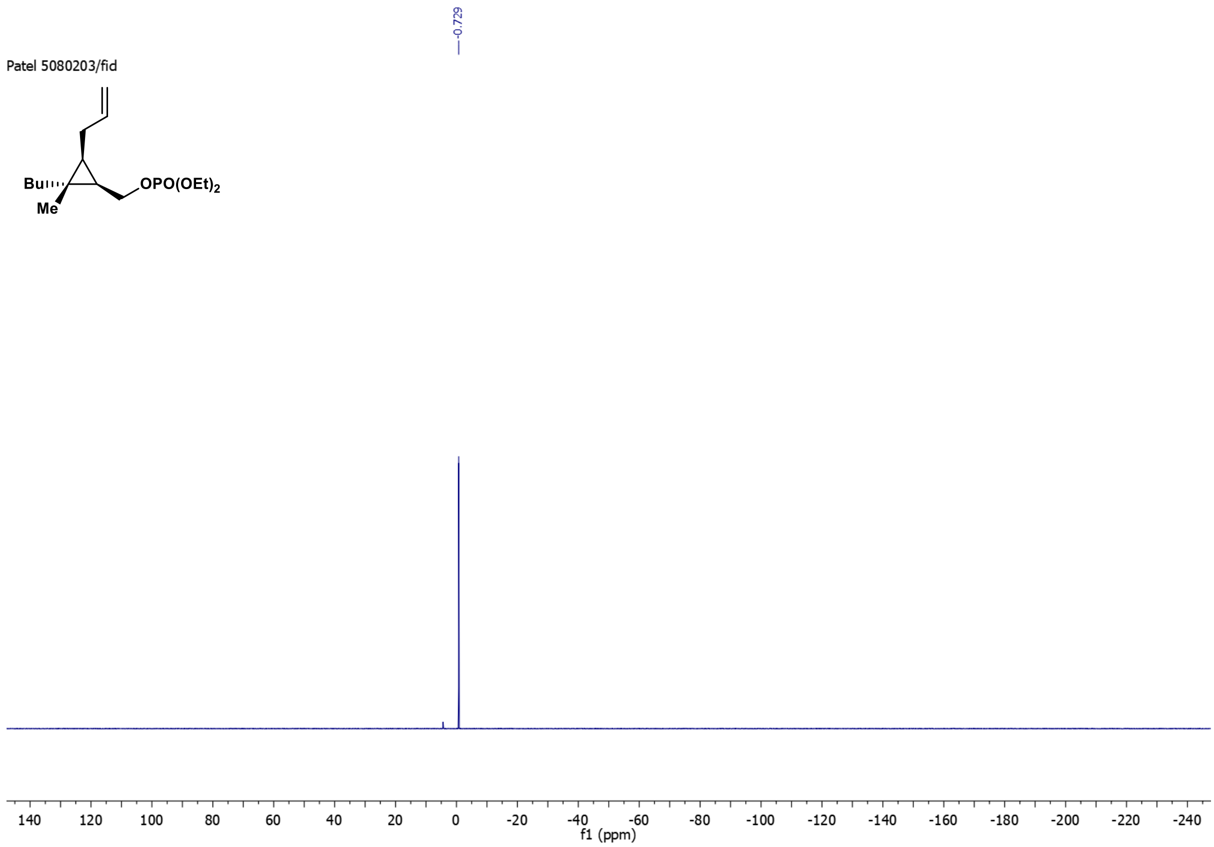
Patel 5080203/fid
Bu
Me
OPO(OEt)2
-0.729
140 120 100 80 60 40 20 0 -20 -40 -60 -80 -100 -120 -140 -160 -180 -200 -220 -240
f1 (ppm)

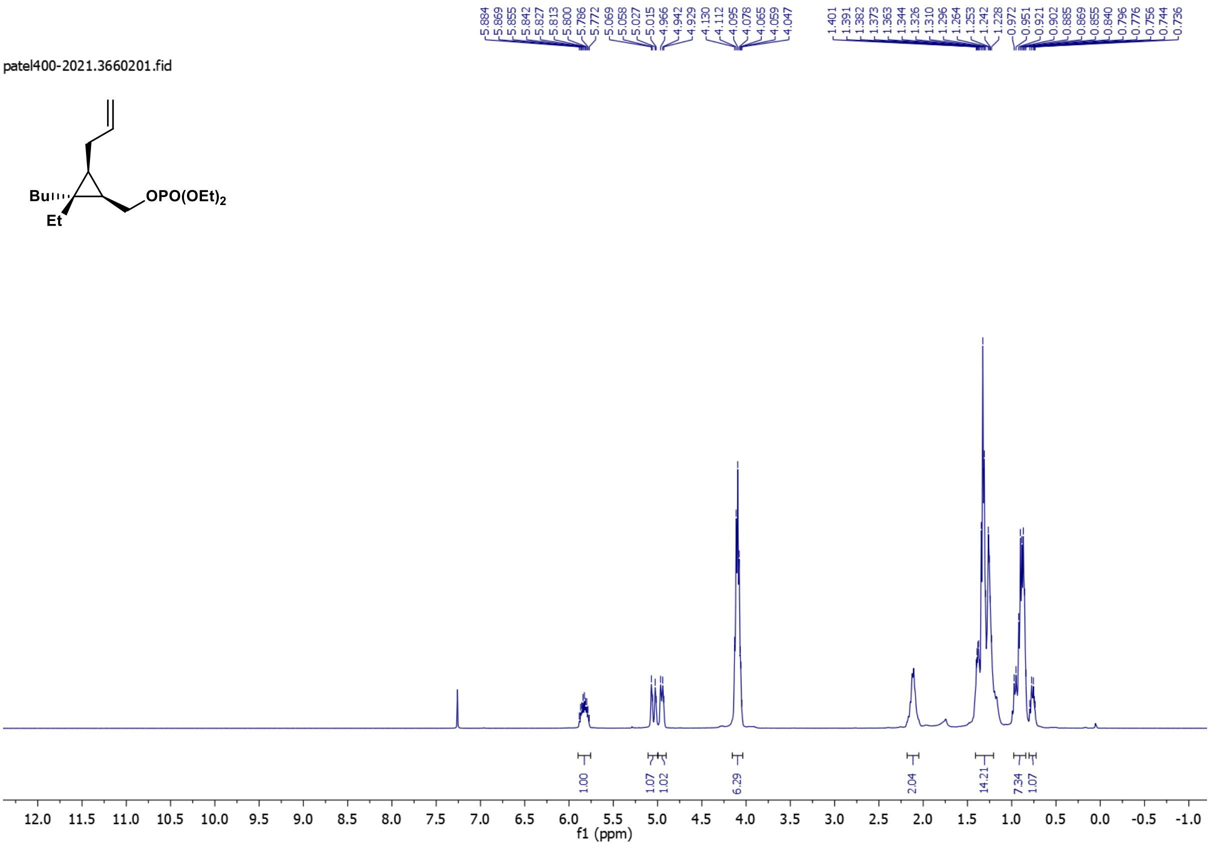
patel400-2021.3660201.fid
Bu
Et
OPO(OEt)2
f1 (ppm)

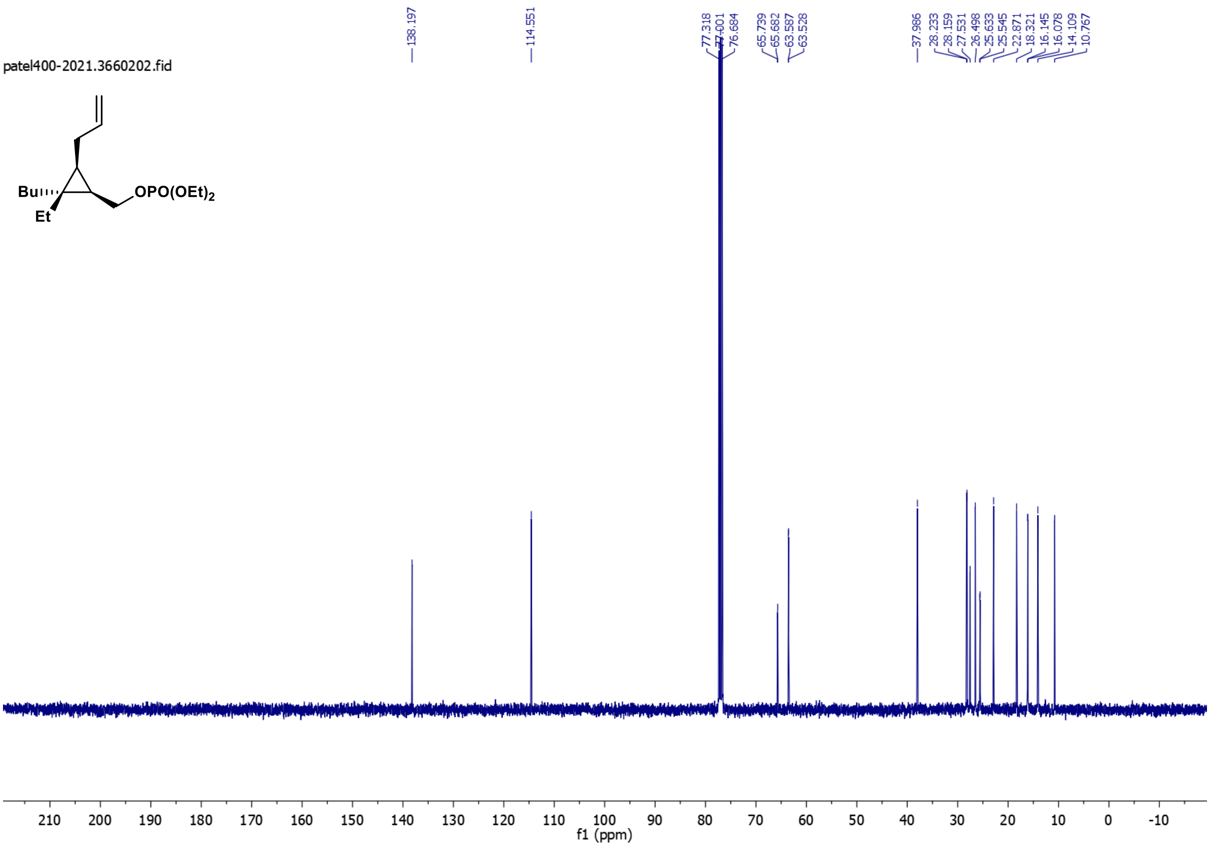
patel400-2021.3660202.fid
Bu
Et
OPO(OEt)2
f1 (ppm)

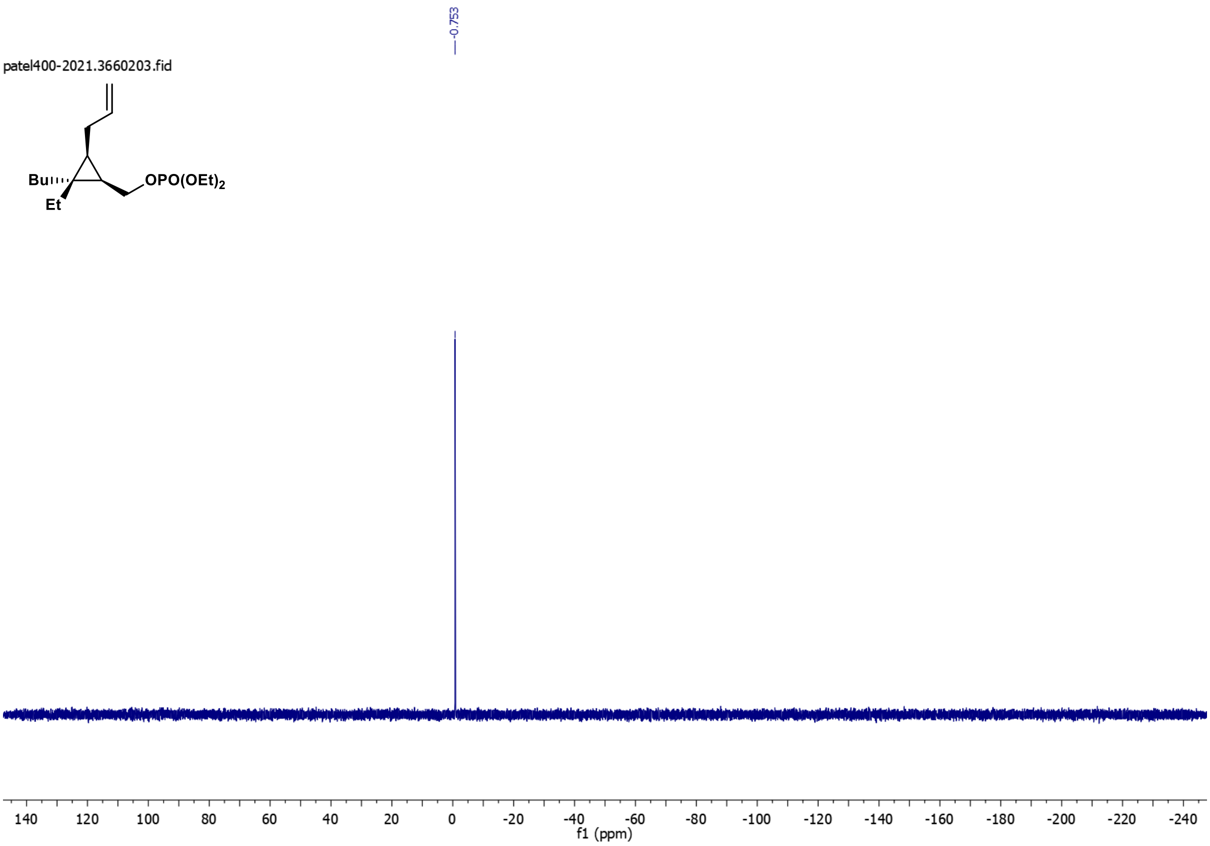
-0.753
patel400-2021.3660203.fid
Bu
Et
OPO(OEt)2
140 120 100 80 60 40 20 0 -20 -40 -60 -80 -100 -120 -140 -160 -180 -200 -220 -240
f1 (ppm)

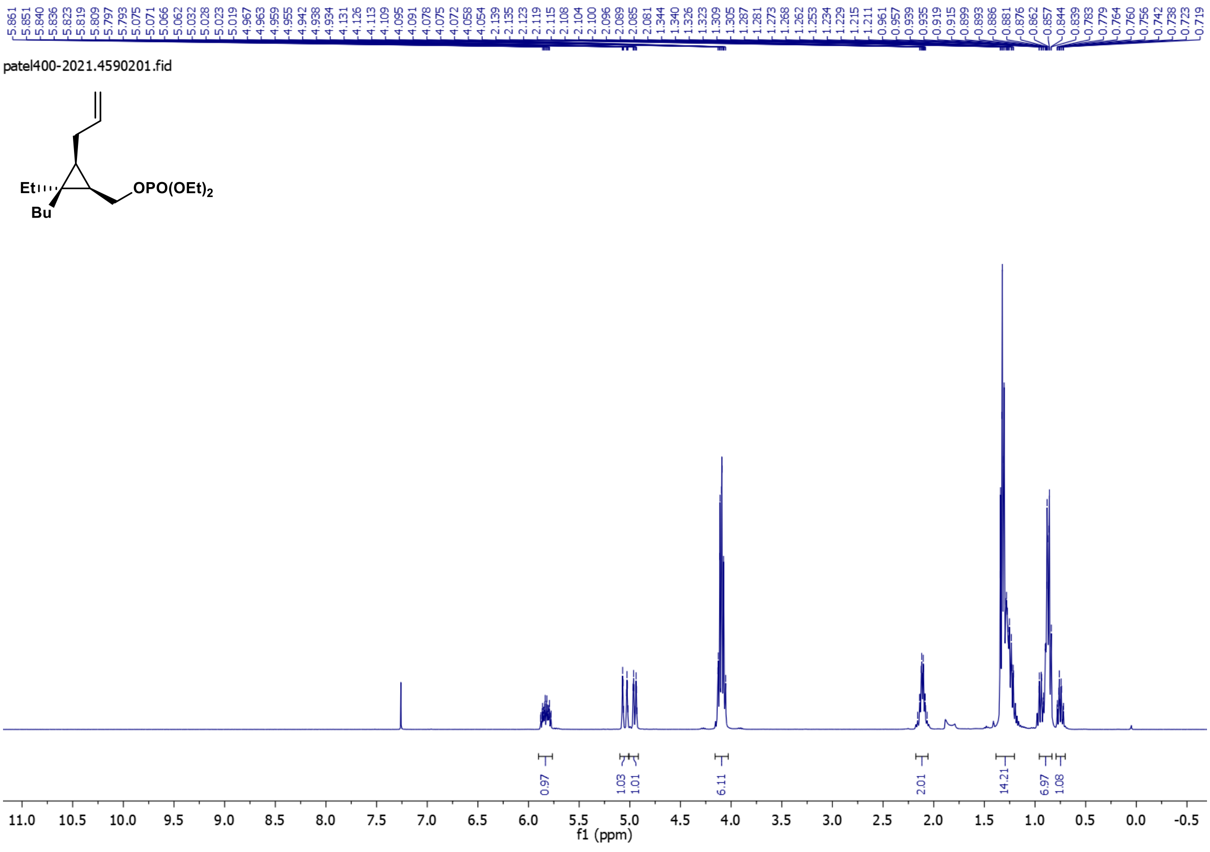
5.861 5.851 5.840 5.836 5.823 5.819 5.809 5.797 5.793 5.075 5.071 5.066 5.062 5.032 5.028 5.023 5.019 4.967 4.963 4.959 4.955 4.942 4.938 4.934 4.131 4.126 4.113 4.109 4.095 4.091 4.078 4.075 4.072 4.058 4.054 2.139 2.135 2.123 2.119 2.115 2.108 2.104 2.100 2.096 2.089 2.085 2.081 1.344 1.340 1.326 1.323 1.309 1.305 1.287 1.281 1.273 1.268 1.262 1.253 1.234 1.229 1.215 1.211 0.961 0.957 0.939 0.935 0.919 0.915 0.899 0.893 0.886 0.881 0.876 0.862 0.857 0.844 0.839 0.783 0.779 0.764 0.760 0.756 0.742 0.738 0.723 0.719
patel400-2021.4590201.fid
Et
Bu
OPO(OEt)2
0.97 1.03 1.01 6.11 2.01 14.21 6.97 1.08
11.0 10.5 10.0 9.5 9.0 8.5 8.0 7.5 7.0 6.5 6.0 5.5 5.0 4.5 4.0 3.5 3.0 2.5 2.0 1.5 1.0 0.5 0.0 -0.5
f1 (ppm)

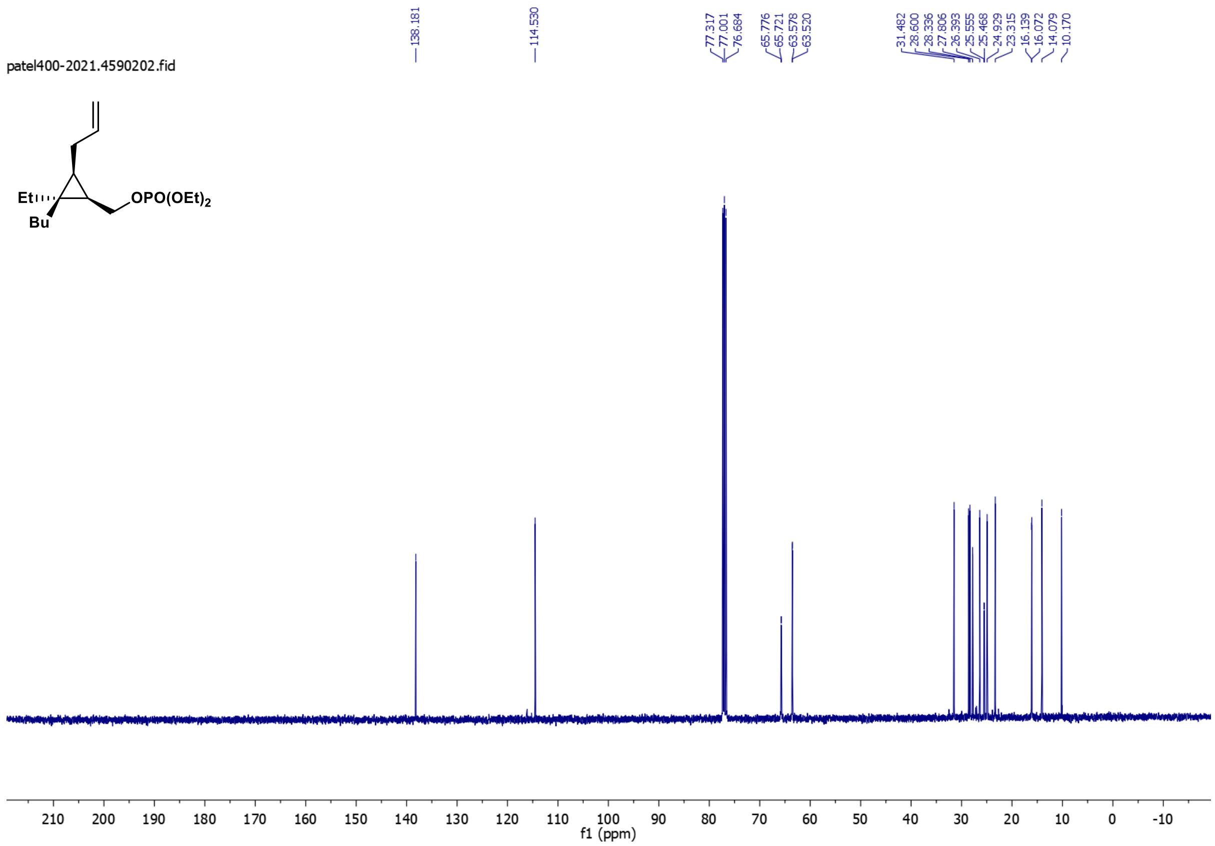

patel400-2021.4590202.fid
138.181
114.530
77.317
77.001
76.684
65.776
65.721
63.578
63.520
31.482
28.600
28.336
27.806
26.393
25.555
25.468
24.929
23.315
16.139
16.072
14.079
10.170
Et
Bu
OPO(OEt)2
210 200 190 180 170 160 150 140 130 120 110 100 90 80 70 60 50 40 30 20 10 0 -10
f1 (ppm)

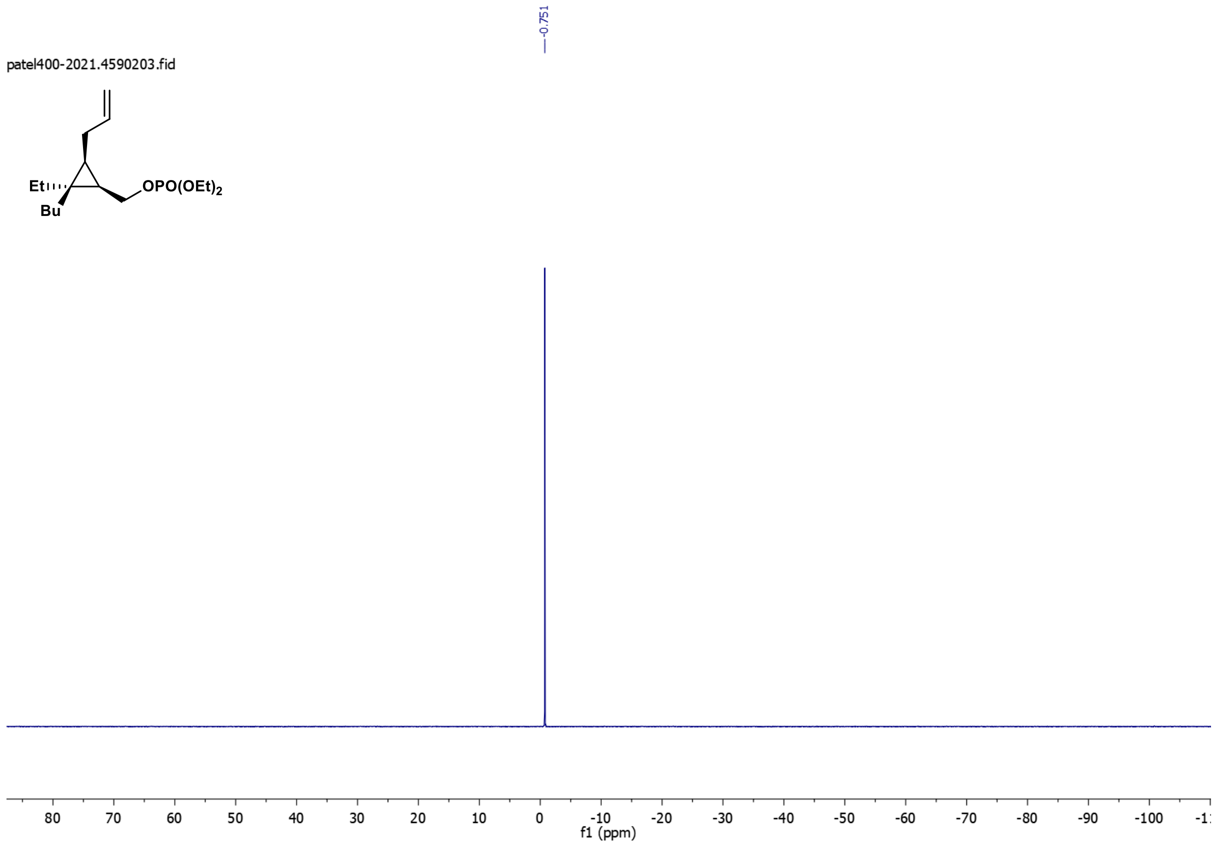

patel400-2021.4590203.fid
-0.751
Et
Bu
OPO(OEt)2
80 70 60 50 40 30 20 10 0 -10 -20 -30 -40 -50 -60 -70 -80 -90 -100
f1 (ppm)

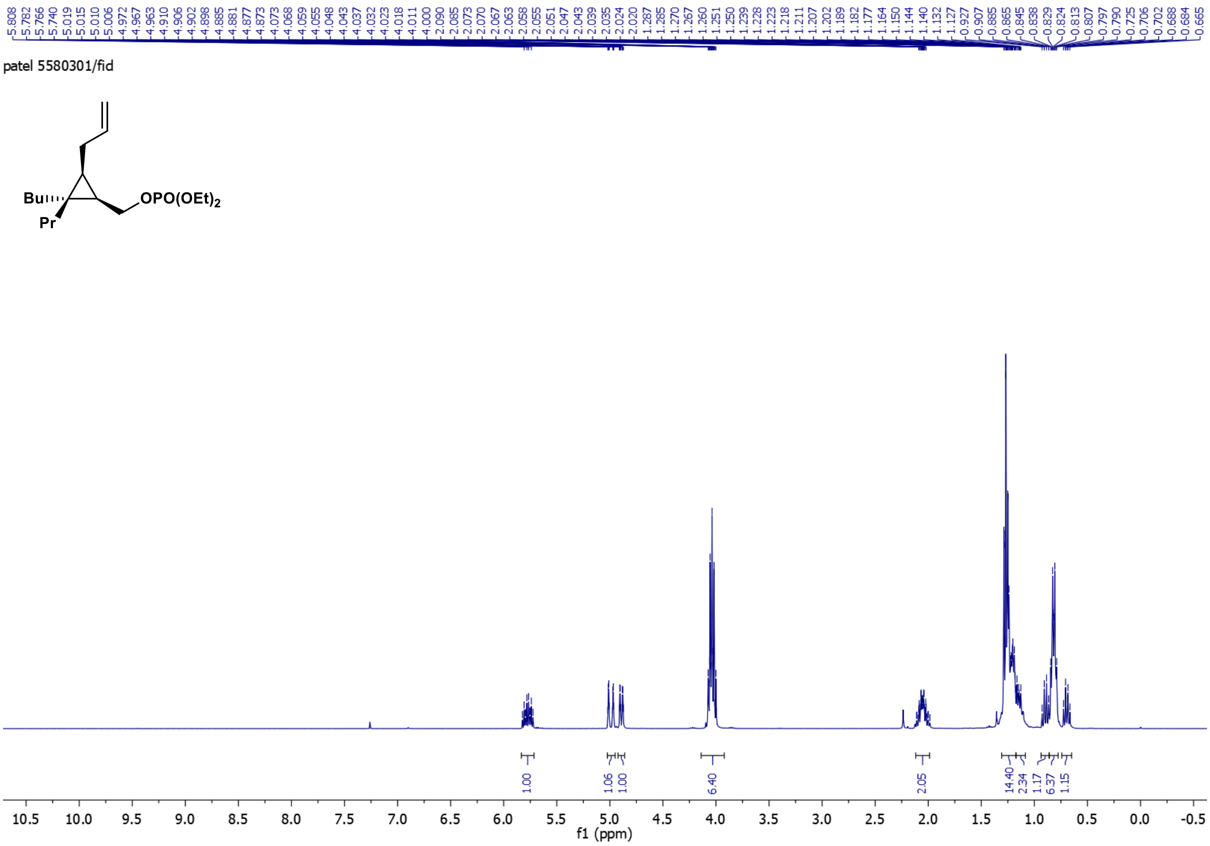
patel 5580301/fid
Bu
Pr
OPO(OEt)2
f1 (ppm)

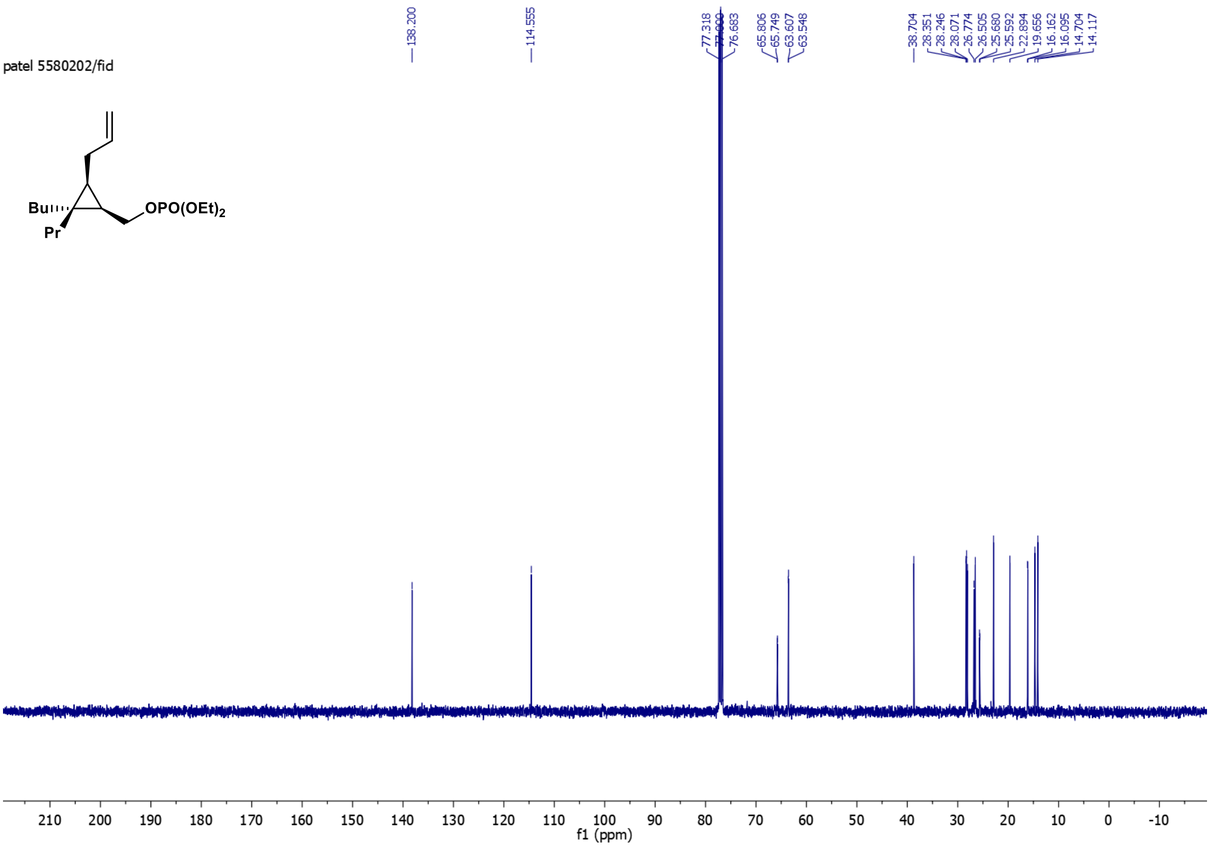
patel 5580202/fid
Bu
Pr
OPO(OEt)2
f1 (ppm)

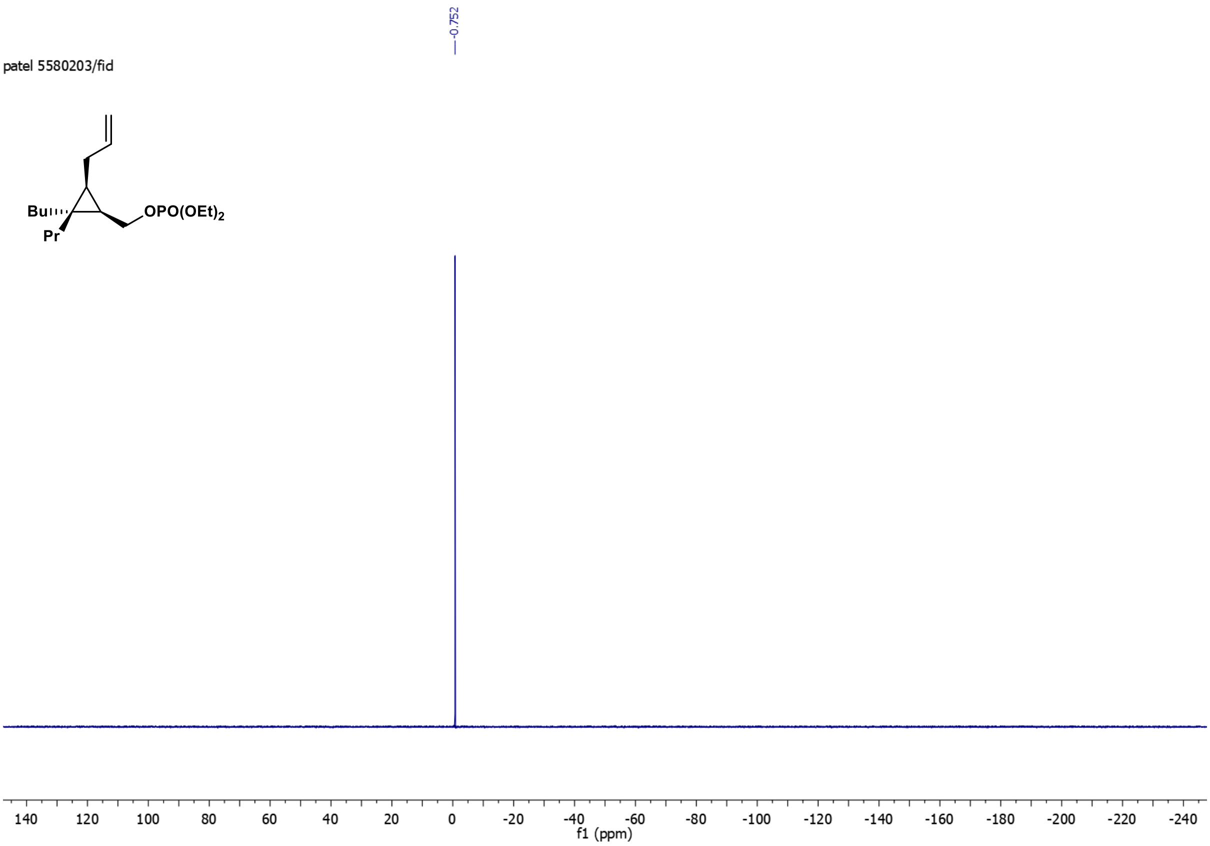
patel 5580203/fid
Bu
Pr
OPO(OEt)2
-0.752
f1 (ppm)

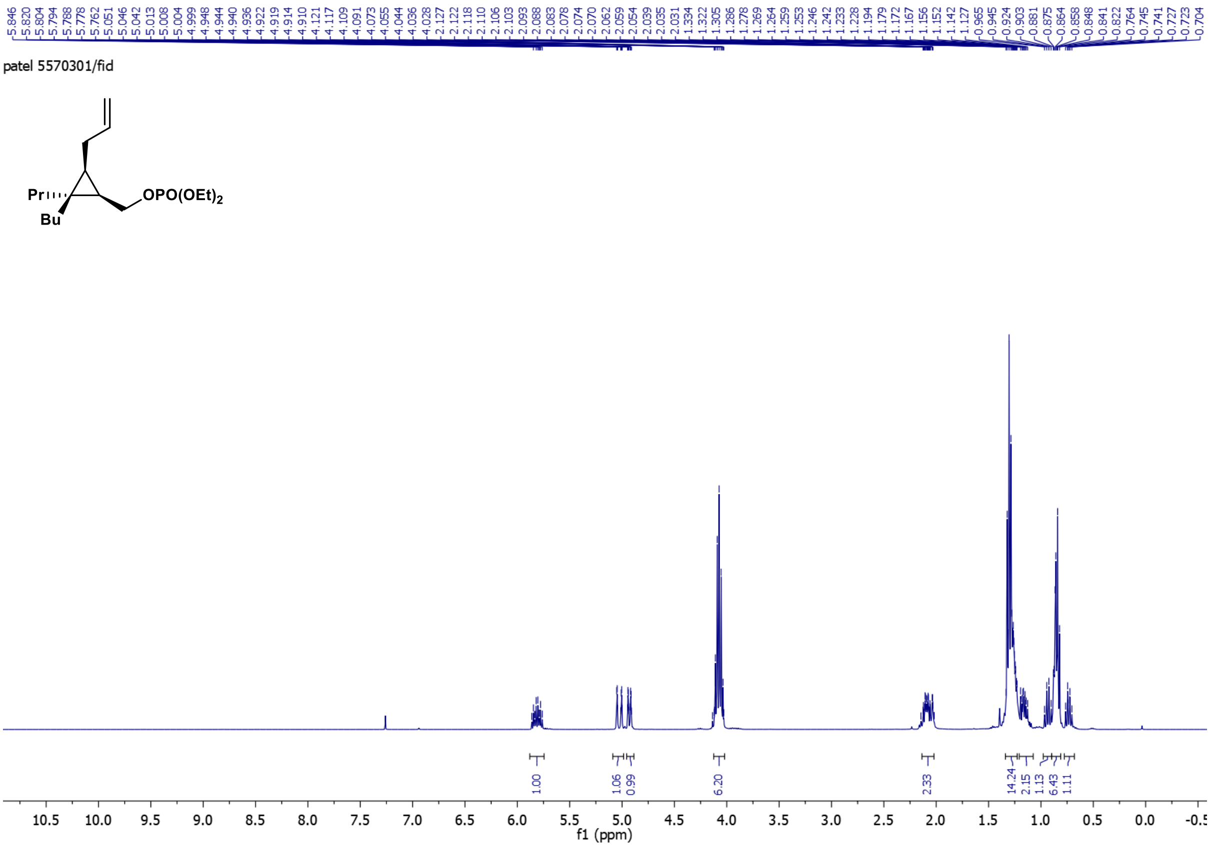
patel 5570301/fid
Pr
Bu
OPO(OEt)2
1.00
1.06
0.99
6.20
2.33
14.24
2.15
1.13
6.43
1.11
f1 (ppm)

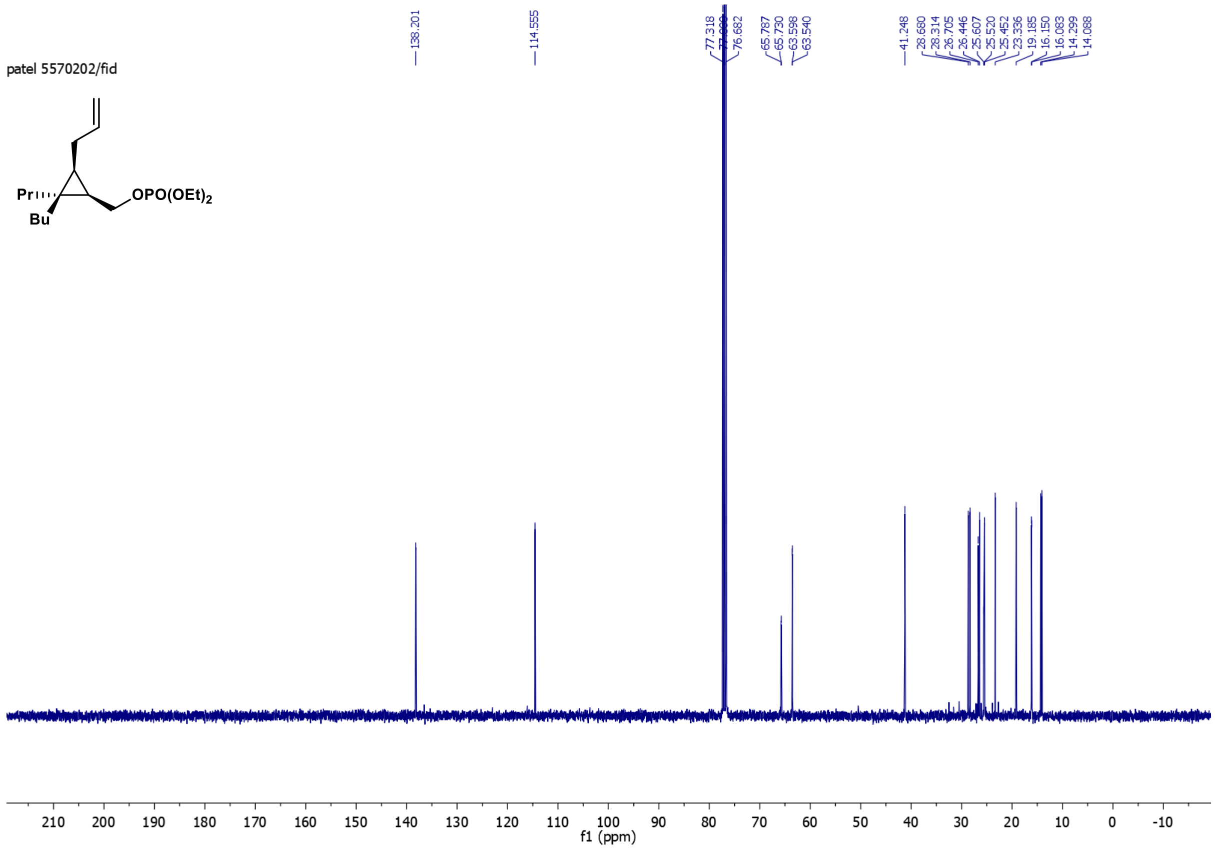
patel 5570202/fid
Pr
Bu
OPO(OEt)2
138.201
114.555
77.318
76.682
65.787
65.730
63.598
63.540
41.248
28.680
28.314
26.705
26.446
25.607
25.520
25.452
23.336
19.185
16.150
16.083
14.299
14.088
210 200 190 180 170 160 150 140 130 120 110 100 90 80 70 60 50 40 30 20 10 0 -10
f1 (ppm)

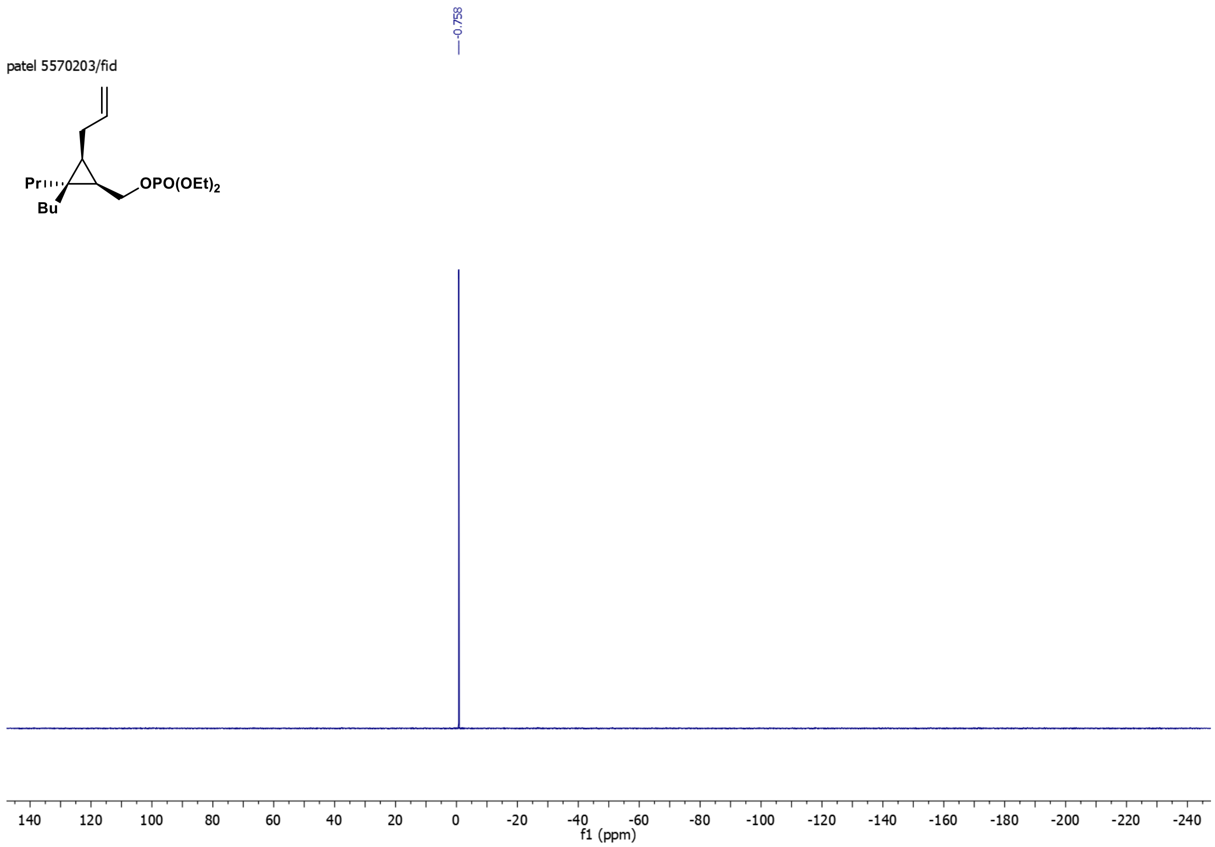
patel 5570203/fid
Pr
Bu
OPO(OEt)2
-0.758
140 120 100 80 60 40 20 0 -20 -40 -60 -80 -100 -120 -140 -160 -180 -200 -220 -240
f1 (ppm)

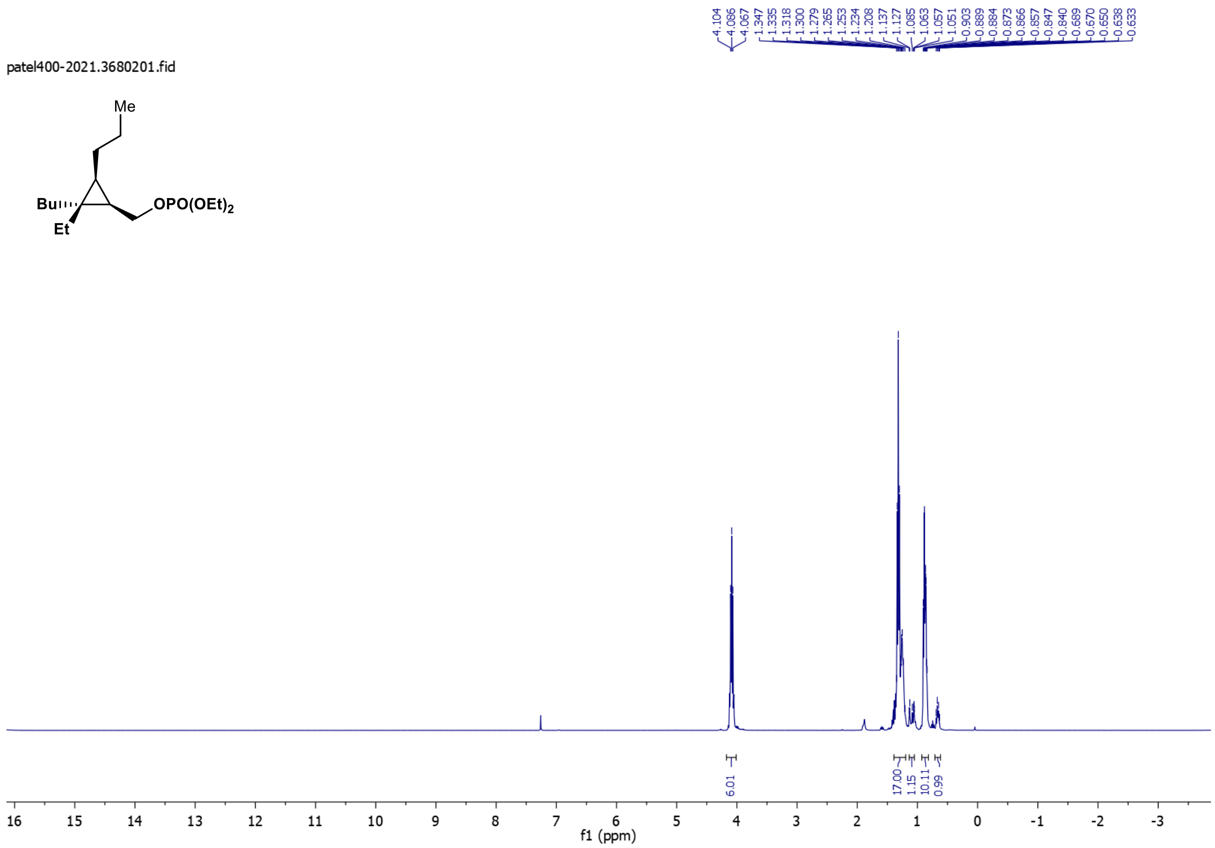
patel400-2021.3680201.fid
Me
Bu
Et
OPO(OEt)2
f1 (ppm)

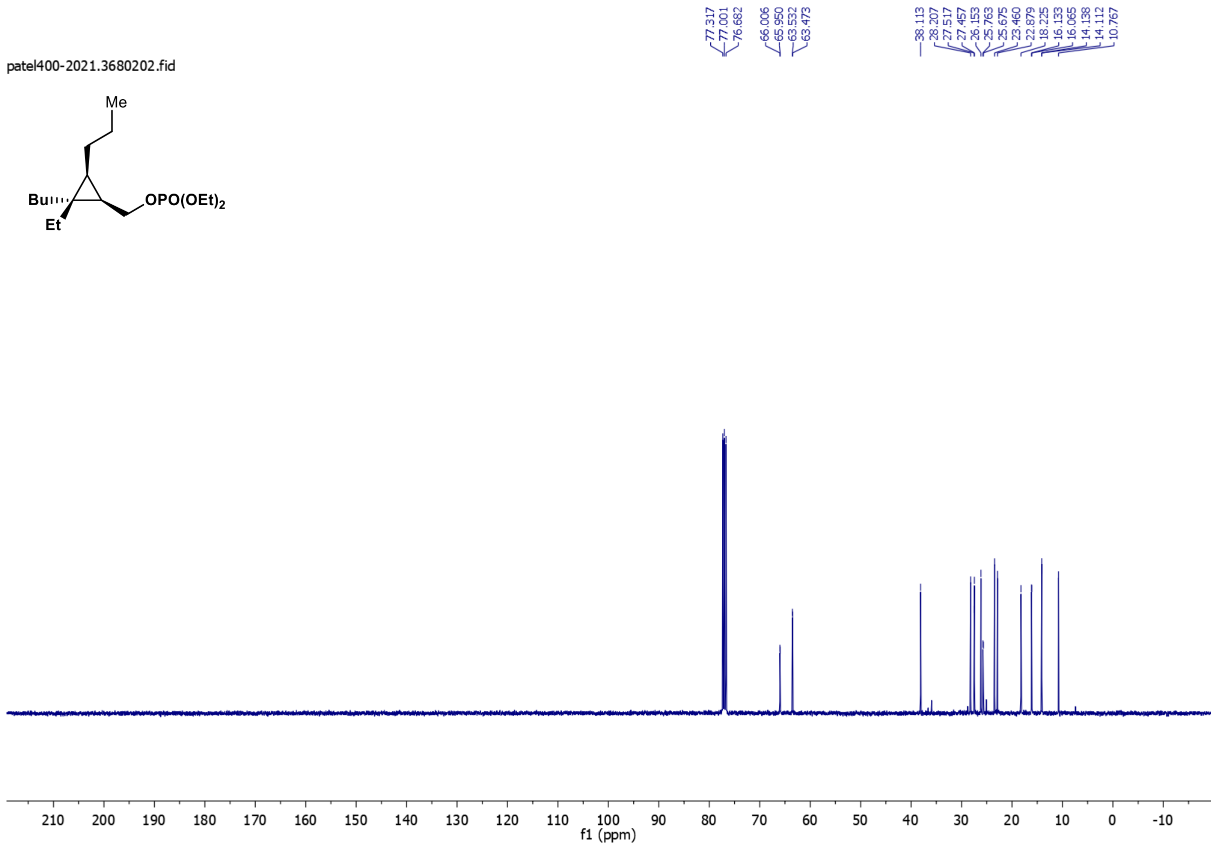
patel400-2021.3680202.fid
Me
Bu
Et
OPO(OEt)2
f1 (ppm)

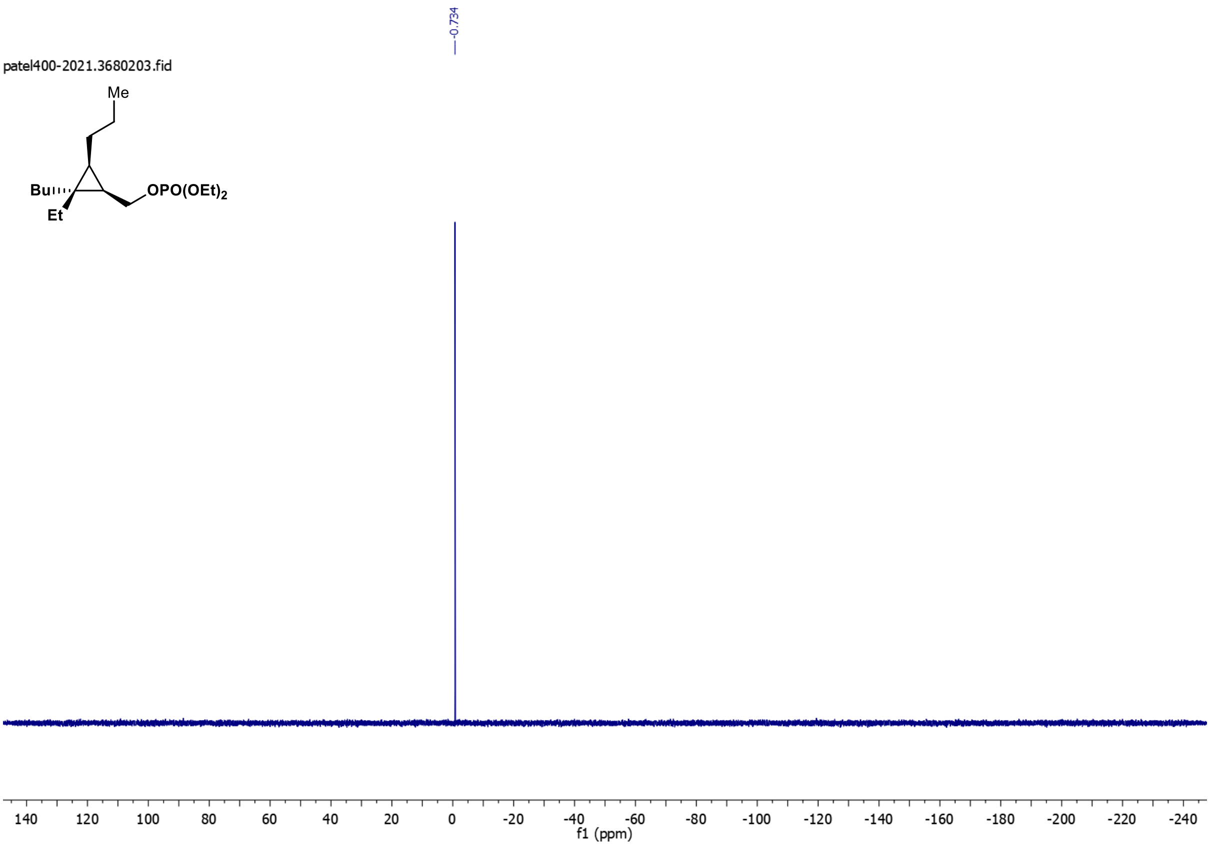

patel400-2021.3680203.fid
-0.734
Me
Bu
Et
OPO(OEt)2
f1 (ppm)

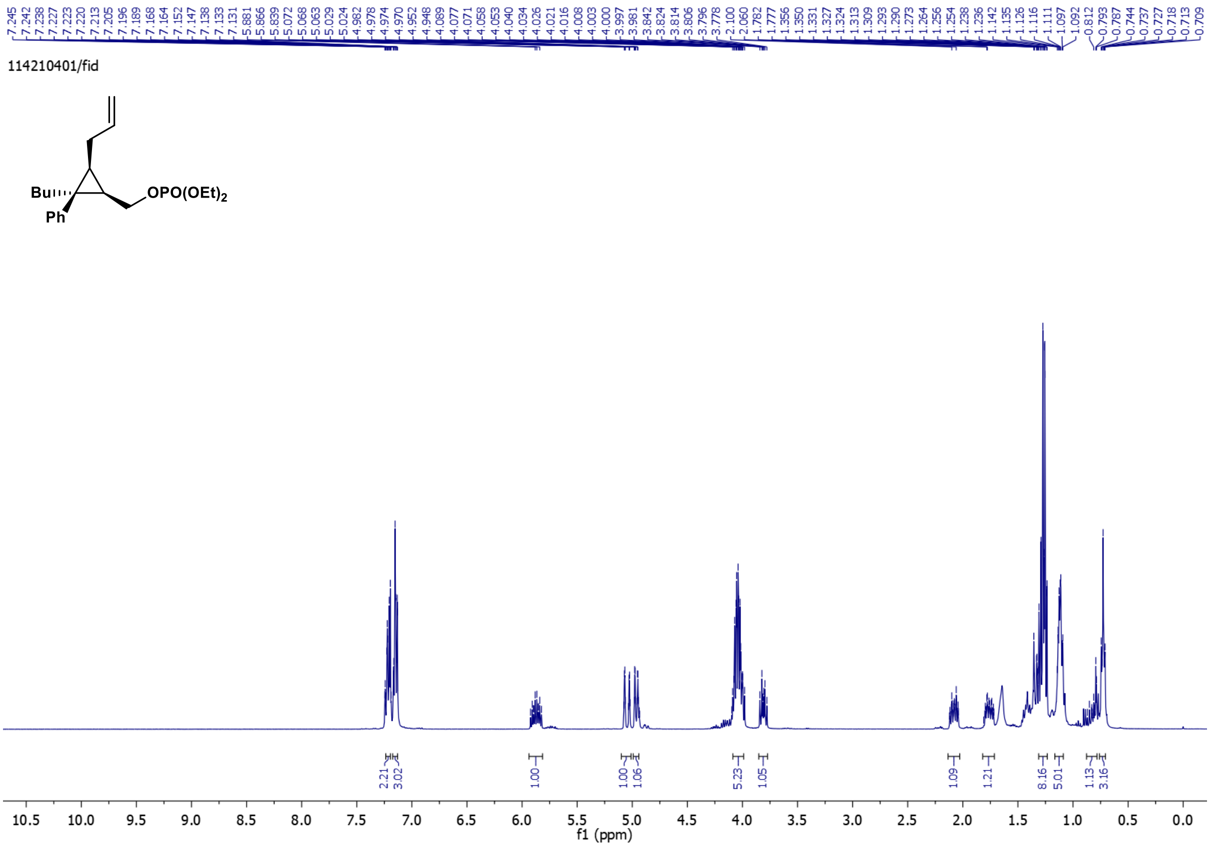

114210401/fid
Bu
Ph
OPO(OEt)2
f1 (ppm)

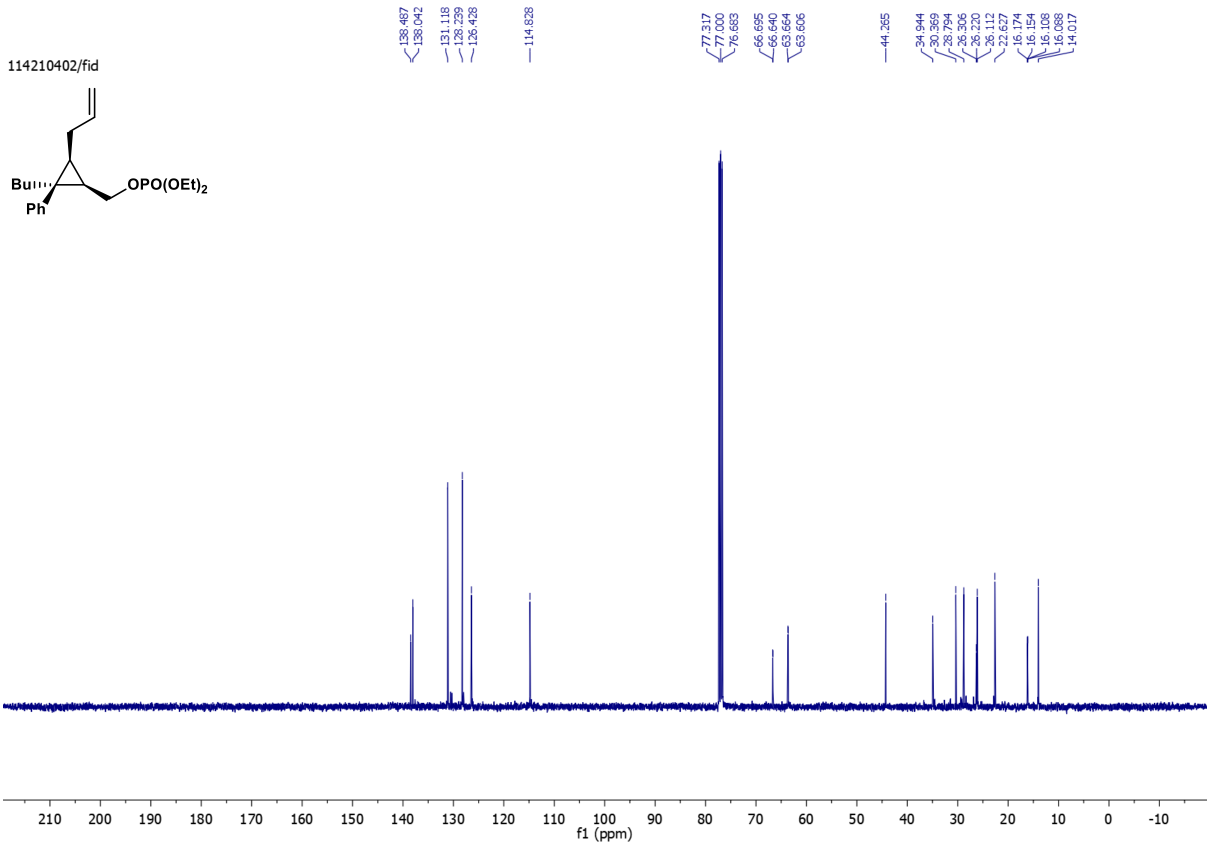
114210402/fid
Bu
Ph
OPO(OEt)2
138.487
138.042
131.118
128.239
126.428
114.828
77.317
77.000
76.683
66.695
66.640
63.664
63.606
44.265
34.944
30.369
28.794
26.306
26.220
26.112
22.627
16.174
16.154
16.108
16.088
14.017
210 200 190 180 170 160 150 140 130 120 110 100 90 80 70 60 50 40 30 20 10 0 -10
f1 (ppm)

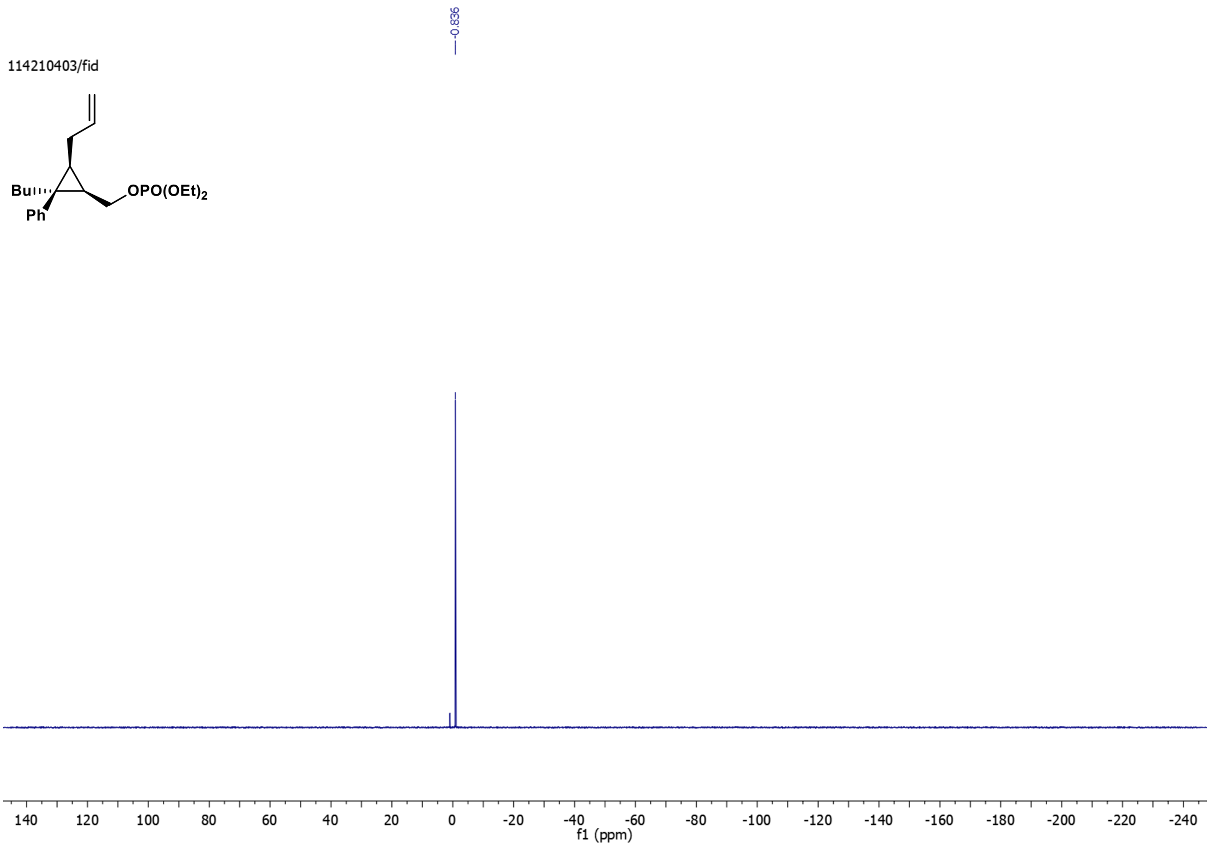
114210403/fid
Bu
Ph
OPO(OEt)2
-0.836
140 120 100 80 60 40 20 0 -20 -40 -60 -80 -100 -120 -140 -160 -180 -200 -220 -240
f1 (ppm)

114650301/fid

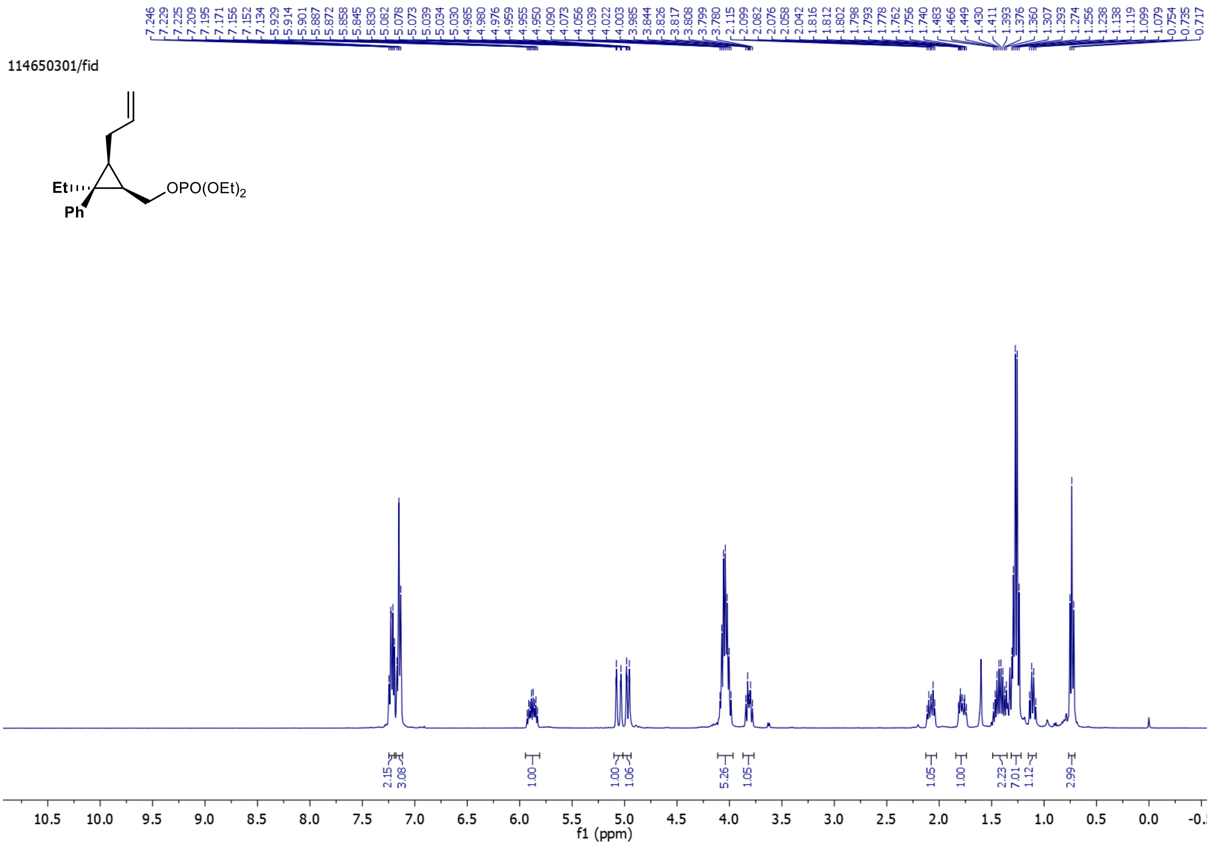

114650302/fid

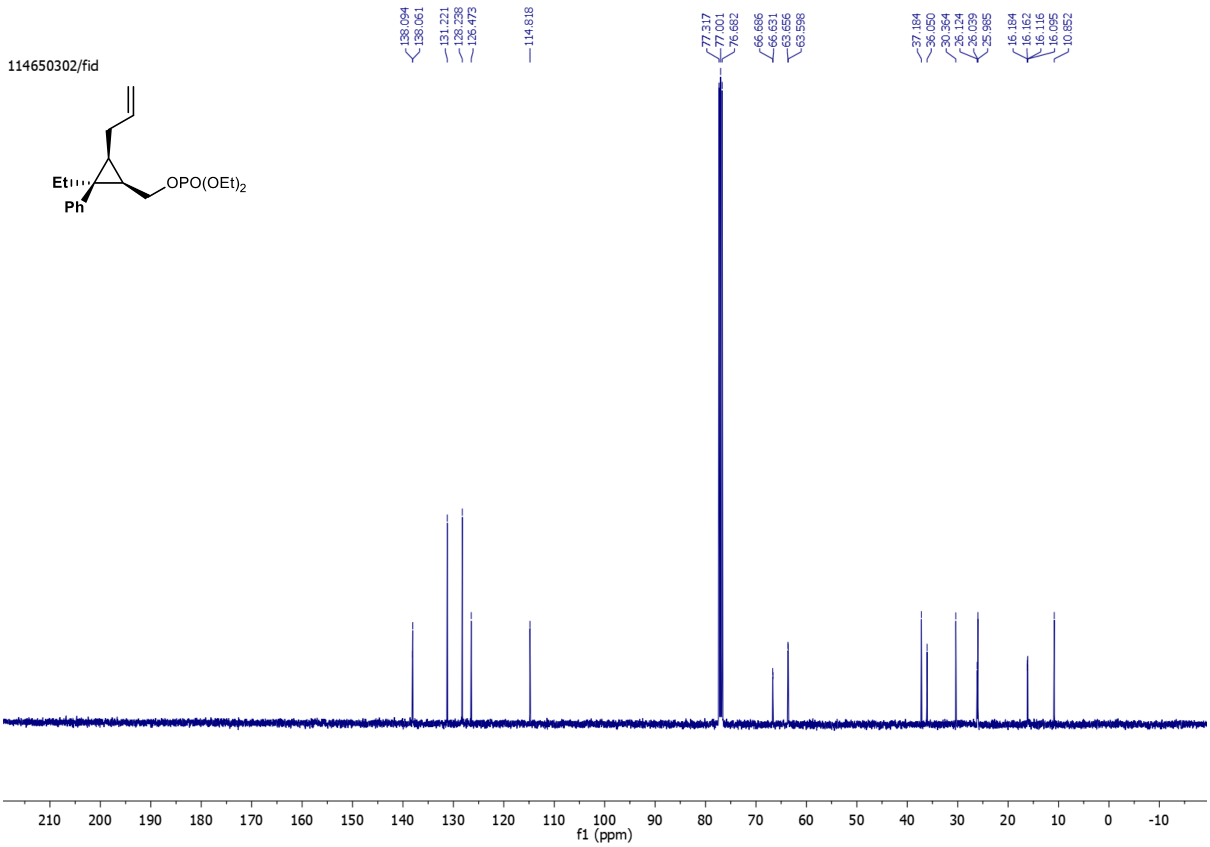

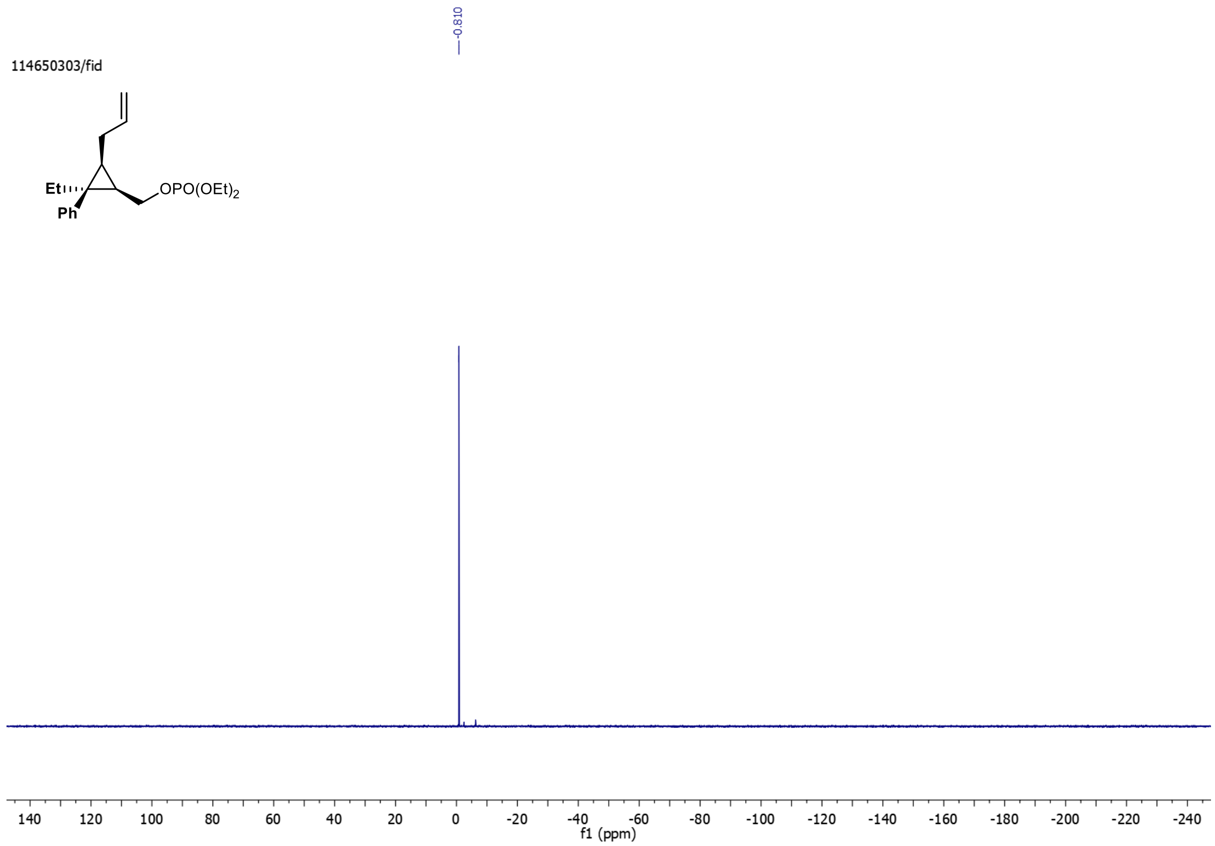
114650303/fid
0.810
Et
Ph
OPO(OEt)2
140 120 100 80 60 40 20 0 -20 -40 -60 -80 -100 -120 -140 -160 -180 -200 -220 -240
f1 (ppm)

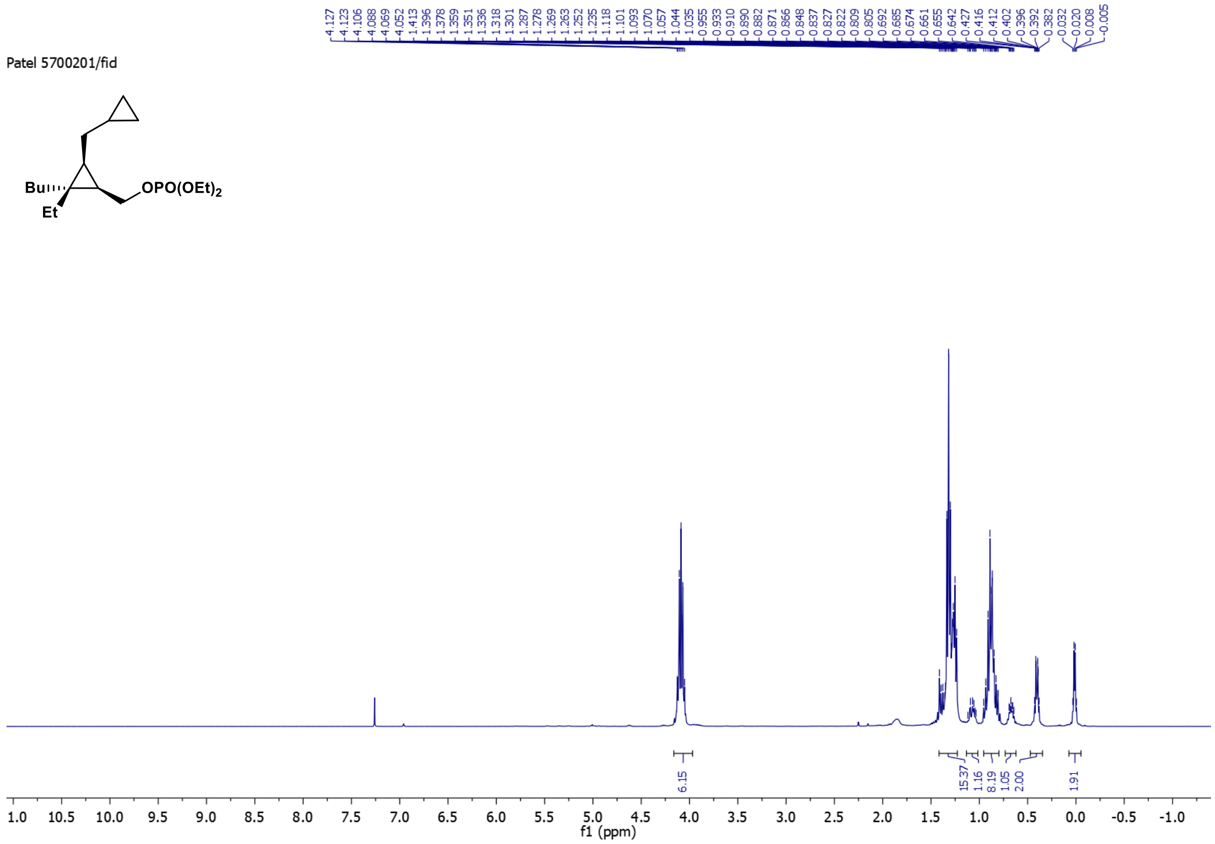
Patel 5700201/fid
4.127 4.123 4.106 4.088 4.069 4.052 1.413 1.396 1.378 1.359 1.351 1.336 1.318 1.301 1.287 1.278 1.269 1.263 1.252 1.235 1.118 1.101 1.093 1.070 1.057 1.044 1.035 0.955 0.933 0.910 0.890 0.882 0.871 0.866 0.848 0.837 0.827 0.822 0.809 0.805 0.692 0.685 0.674 0.661 0.655 0.642 0.427 0.416 0.412 0.402 0.396 0.392 0.382 0.032 0.020 0.008 -0.005
Bu
Et
OPO(OEt)2
6.15
15.37
1.16
8.19
1.05
2.00
1.91
1.0 10.5 10.0 9.5 9.0 8.5 8.0 7.5 7.0 6.5 6.0 5.5 5.0 4.5 4.0 3.5 3.0 2.5 2.0 1.5 1.0 0.5 0.0 -0.5 -1.0
f1 (ppm)

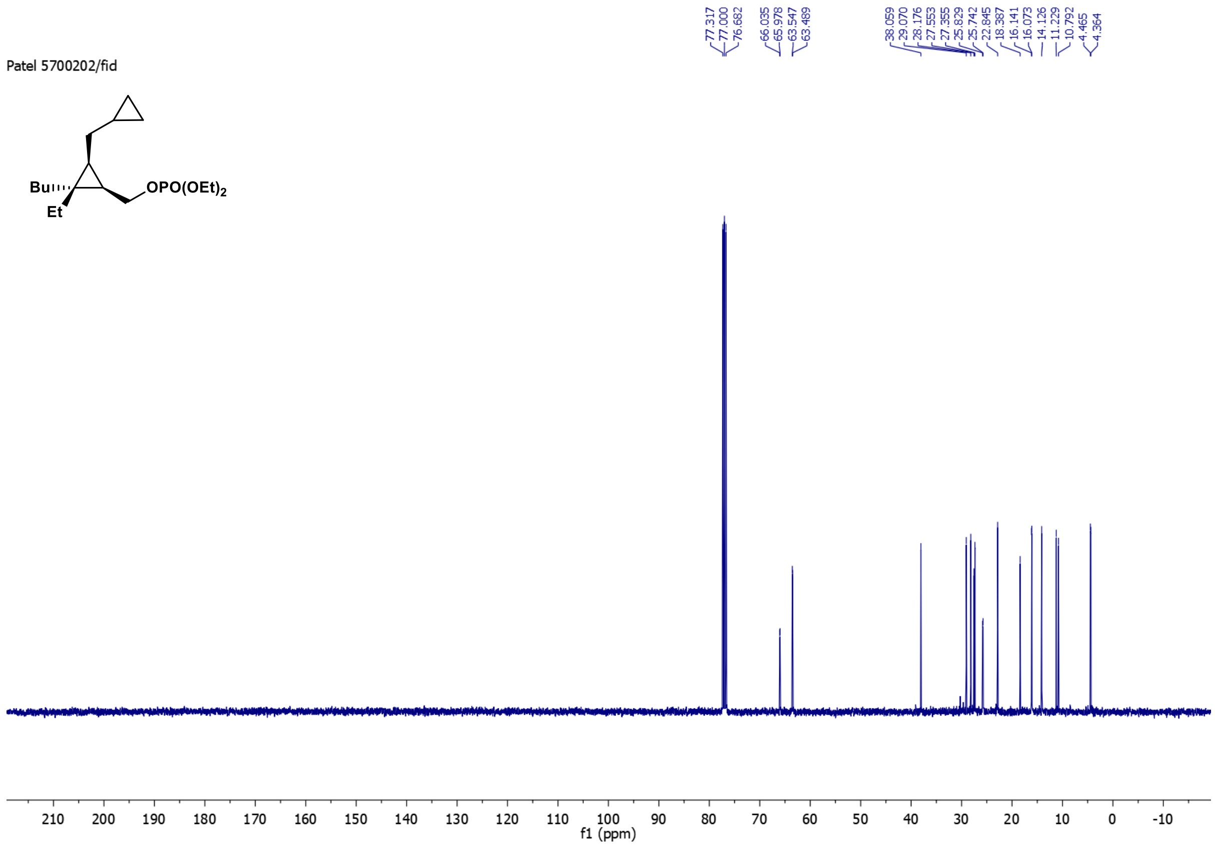
Patel 5700202/fid
77.317
77.000
76.682
66.035
65.978
63.547
63.489
38.059
29.070
28.176
27.553
27.355
25.829
25.742
22.845
18.387
16.141
16.073
14.126
11.229
10.792
4.465
4.364
Bu
Et
OPO(OEt)2
f1 (ppm)

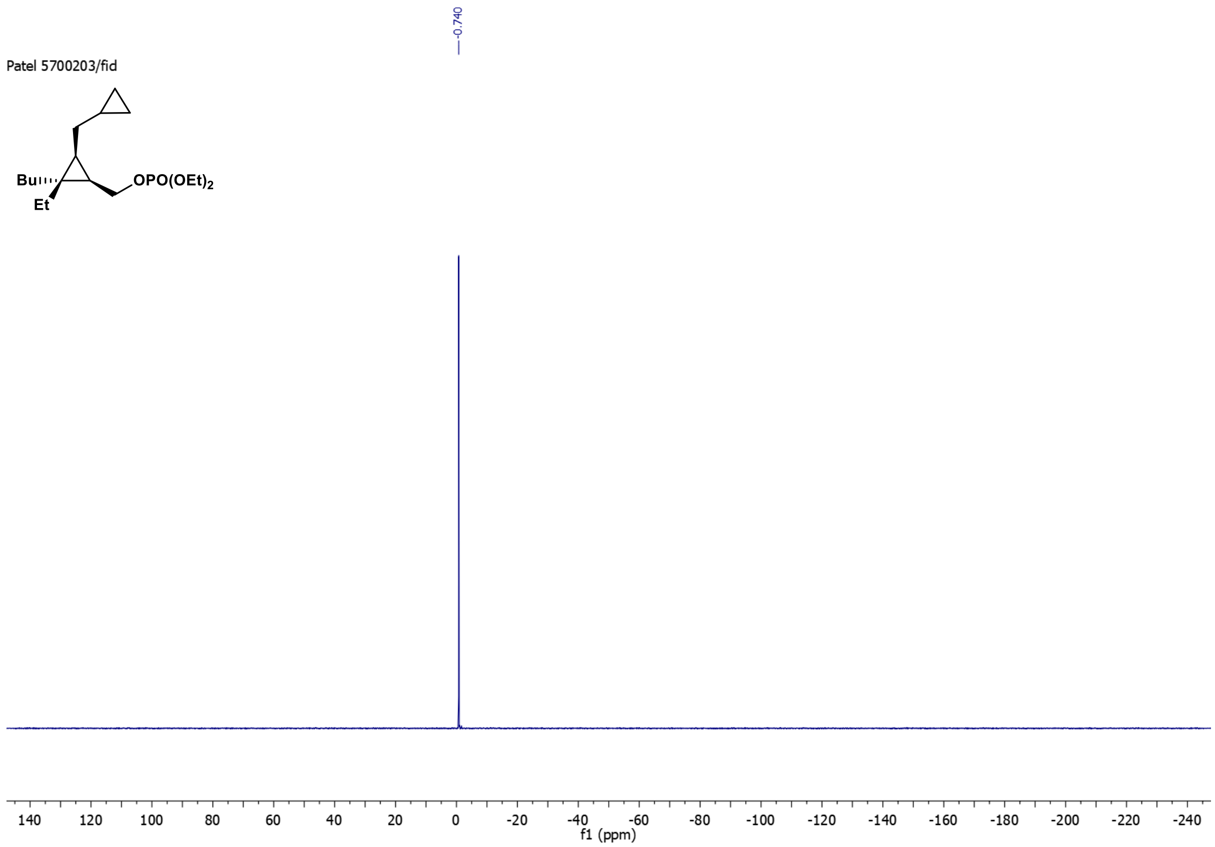
Patel 5700203/fid
-0.740
Bu
Et
OPO(OEt)2
f1 (ppm)

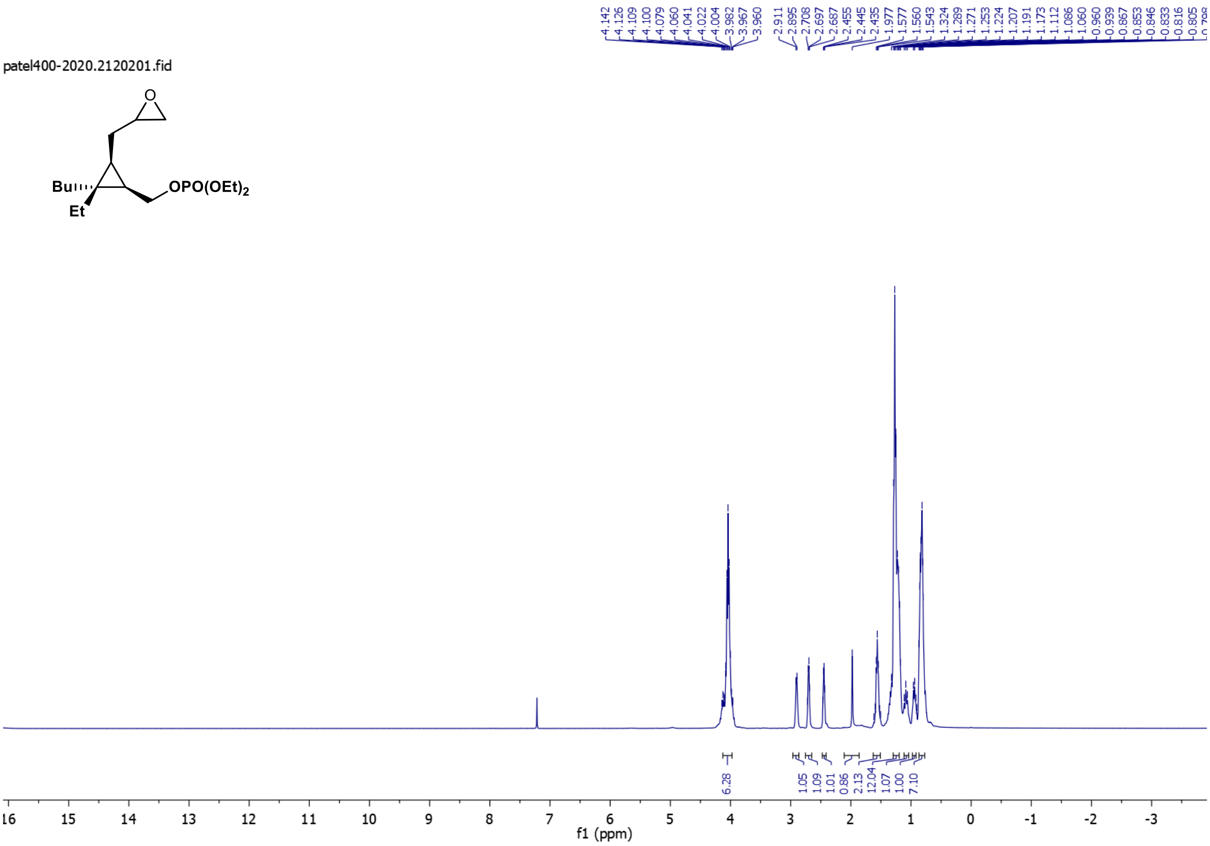
patel400-2020.2120201.fid
Bu
Et
OPO(OEt)2
O
f1 (ppm)

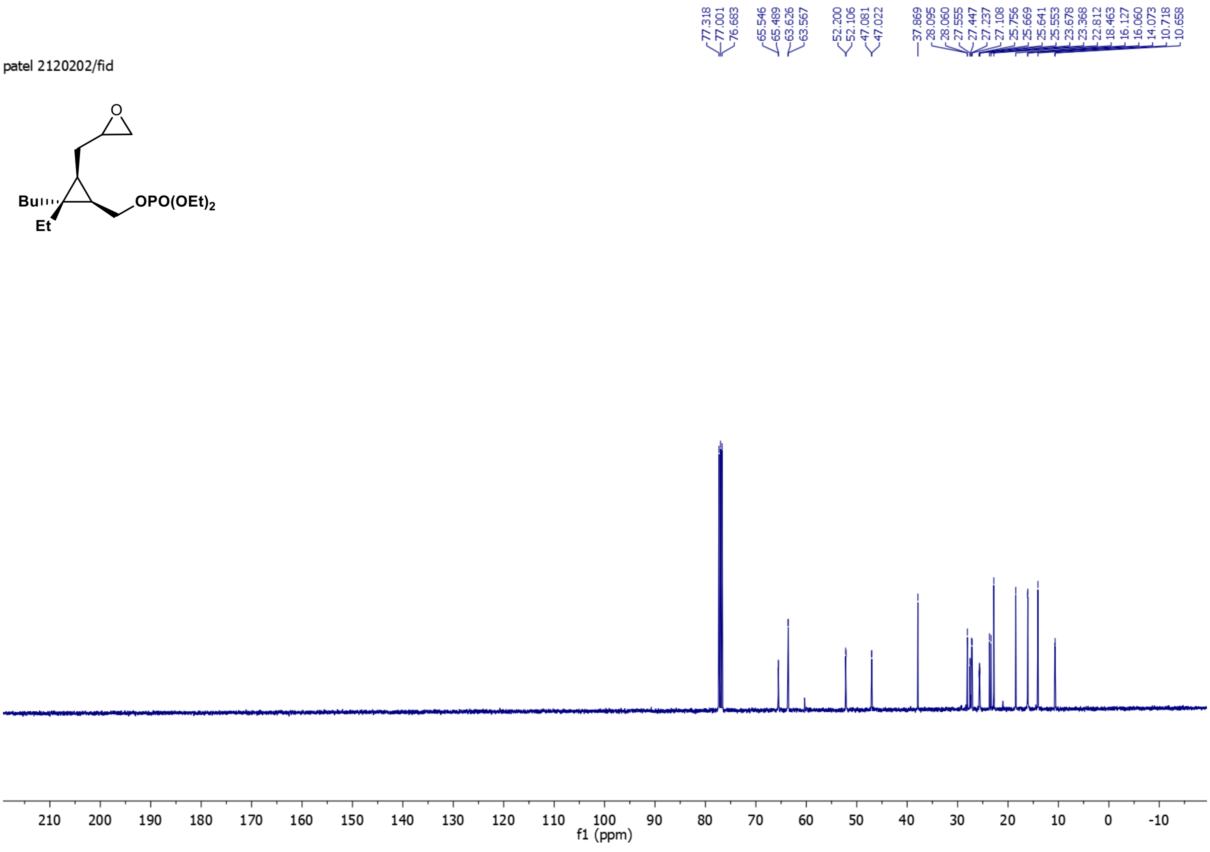
patel 2120202/fid
Bu
Et
OPO(OEt)2
O
f1 (ppm)

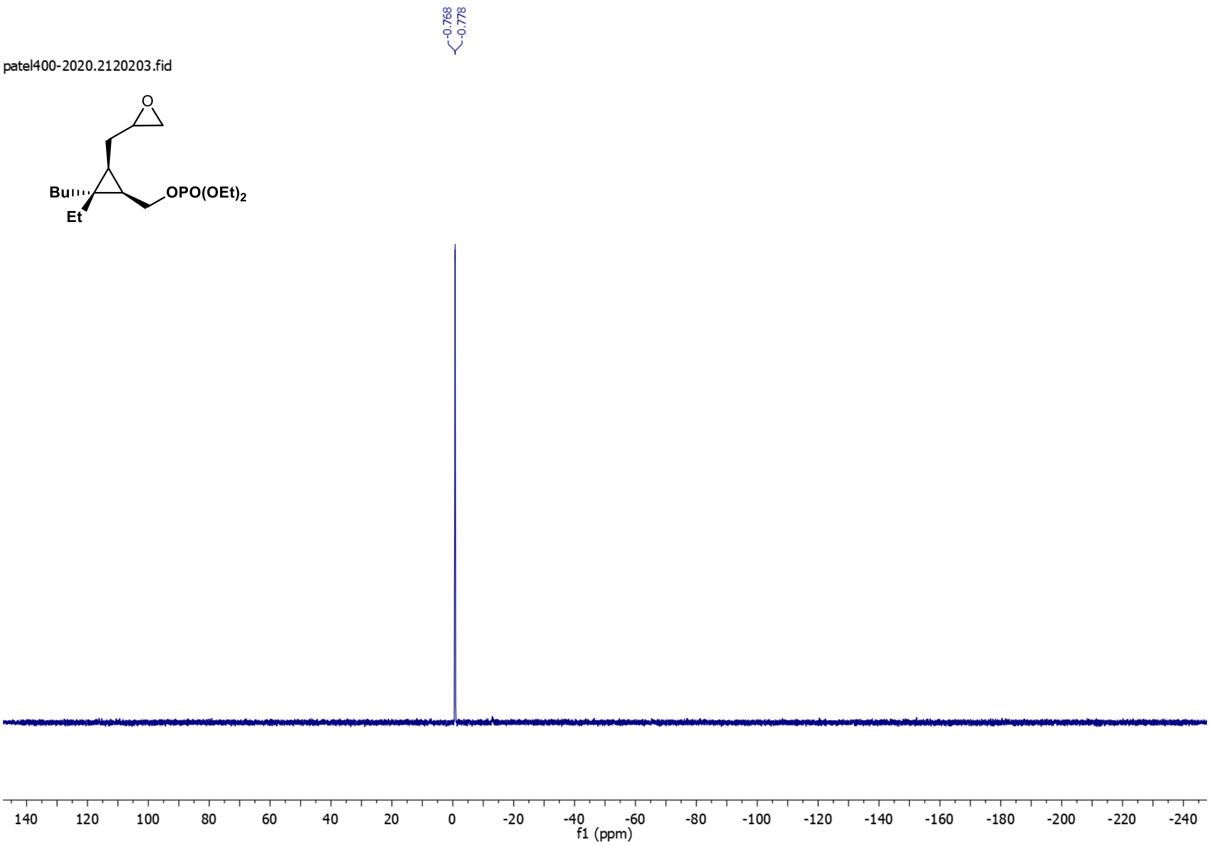
patel400-2020.2120203.fid
O
Bu
OPO(OEt)2
Et
f1 (ppm)

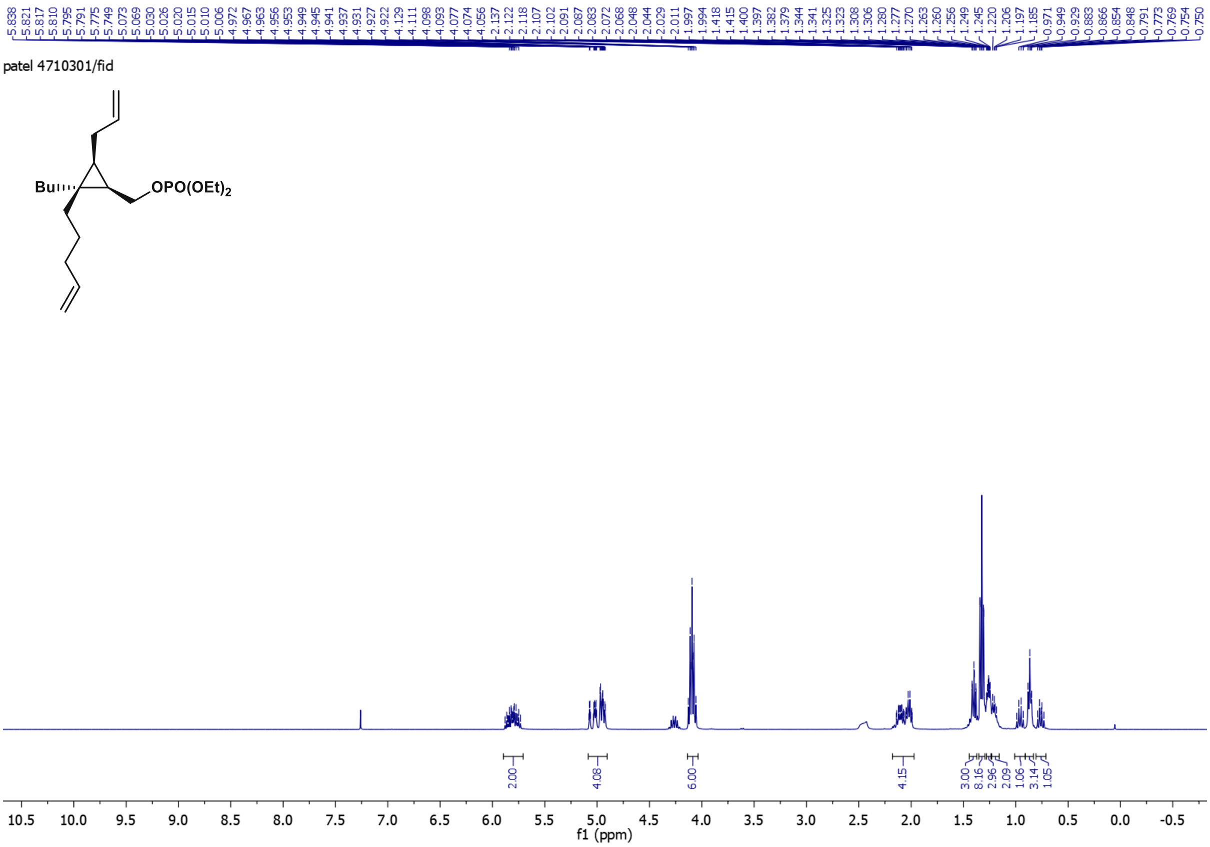
patel 4710301/fid
Bu
OPO(OEt)2
f1 (ppm)

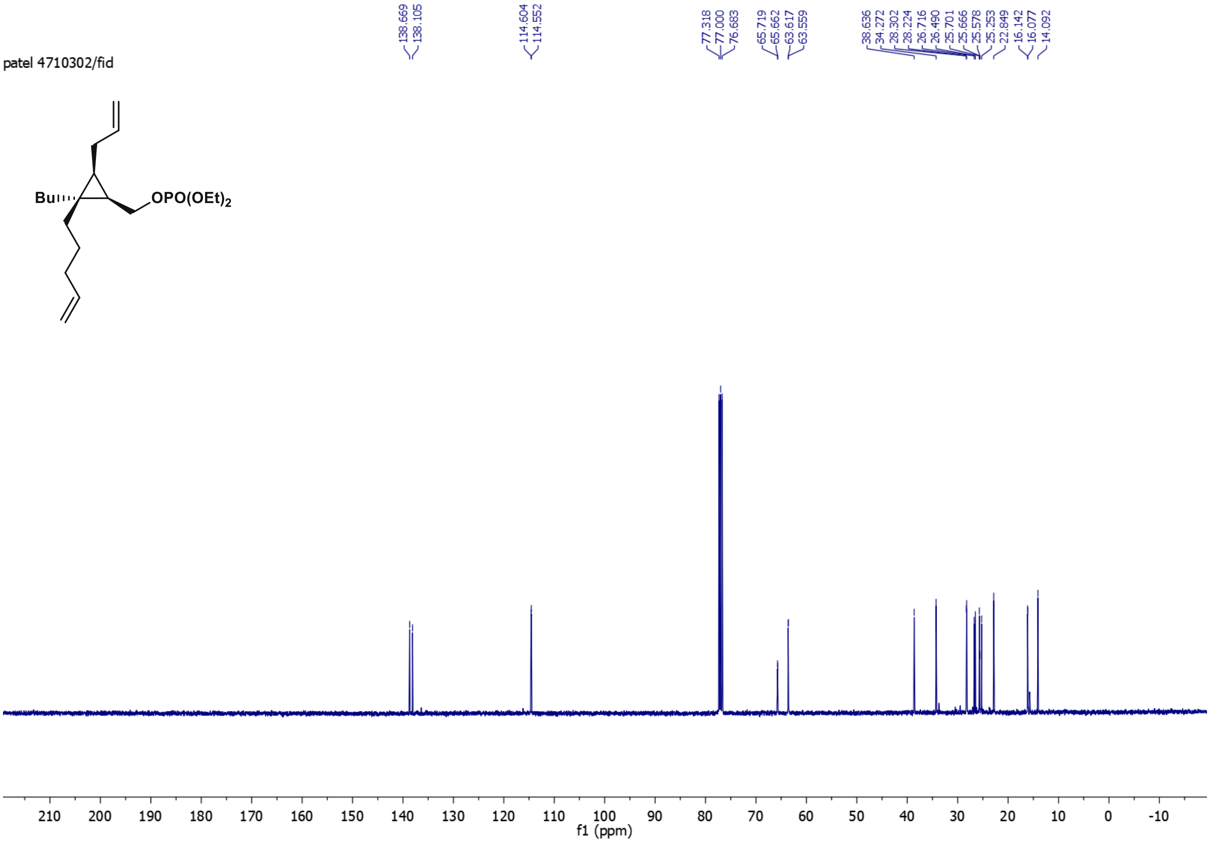
patel 4710302/fid
138.669
138.105
114.604
114.552
77.318
77.000
76.683
65.719
65.662
63.617
63.559
38.636
34.272
28.302
28.224
26.716
26.490
25.701
25.666
25.578
25.253
22.849
16.142
16.077
14.092
Bu
OPO(OEt)$_2$
f1 (ppm)

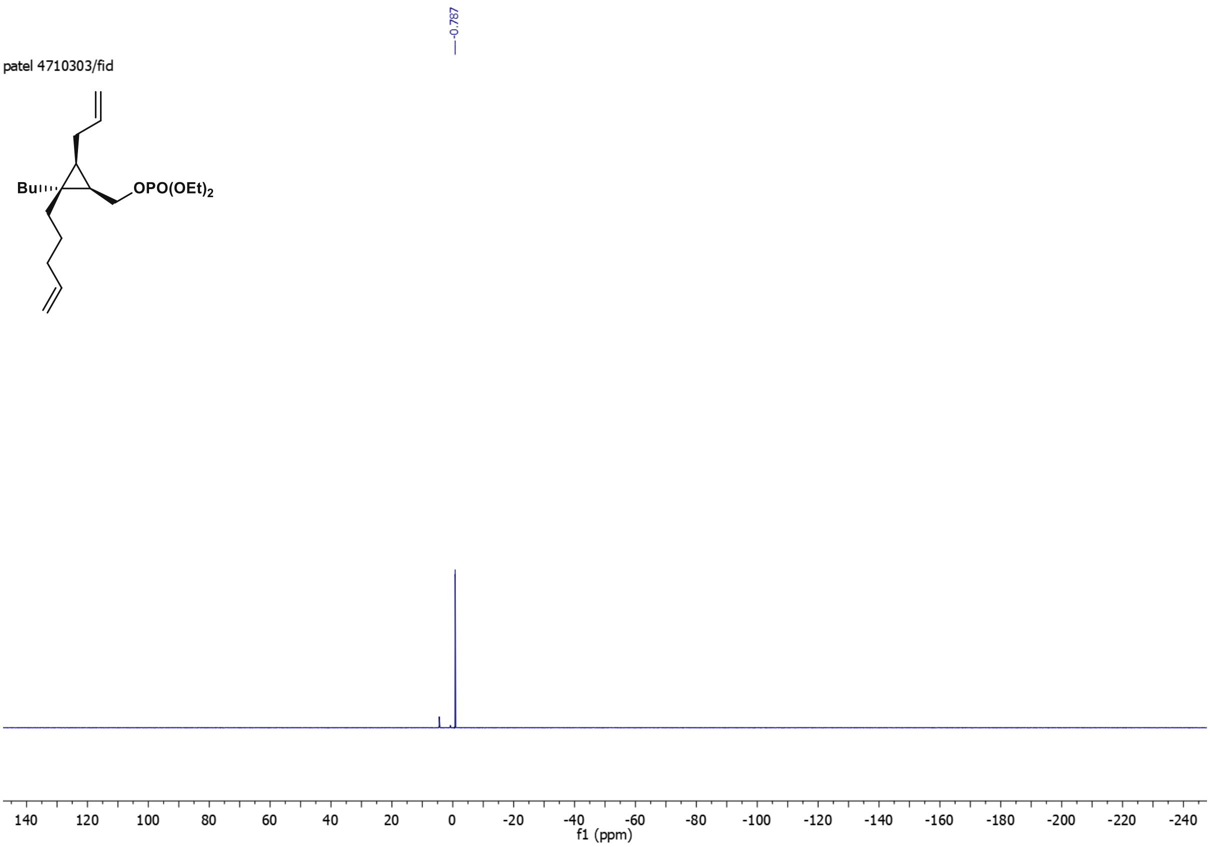
patel 4710303/fid
-0.787
Bu
OPO(OEt)$_2$
f1 (ppm)

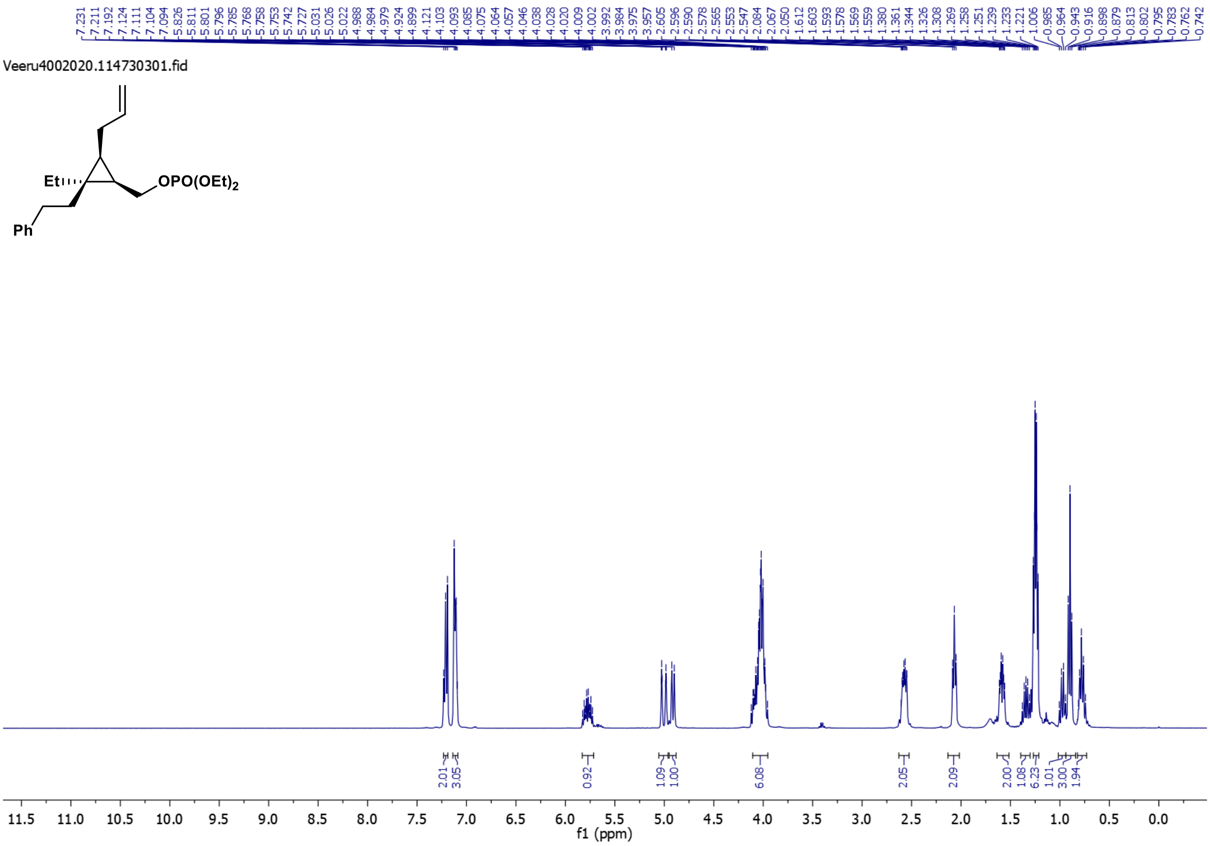
7.231 7.211 7.192 7.124 7.111 7.104 7.094 5.826 5.811 5.801 5.796 5.785 5.768 5.758 5.753 5.742 5.727 5.031 5.026 5.022 4.988 4.984 4.979 4.924 4.899 4.121 4.103 4.093 4.085 4.075 4.064 4.057 4.046 4.038 4.028 4.020 4.009 4.002 3.992 3.984 3.975 3.957 2.605 2.596 2.590 2.578 2.565 2.553 2.547 2.084 2.067 2.050 1.612 1.603 1.593 1.578 1.569 1.559 1.380 1.361 1.344 1.326 1.308 1.269 1.258 1.251 1.239 1.233 1.221 1.006 0.985 0.964 0.943 0.916 0.898 0.879 0.813 0.802 0.795 0.783 0.762 0.742
Veeru4002020.114730301.fid
Et
OPO(OEt)2
Ph
2.01 3.05 0.92 1.09 1.00 6.08 2.05 2.09 2.00 1.08 6.23 1.01 3.00 1.94
11.5 11.0 10.5 10.0 9.5 9.0 8.5 8.0 7.5 7.0 6.5 6.0 5.5 5.0 4.5 4.0 3.5 3.0 2.5 2.0 1.5 1.0 0.5 0.0
f1 (ppm)

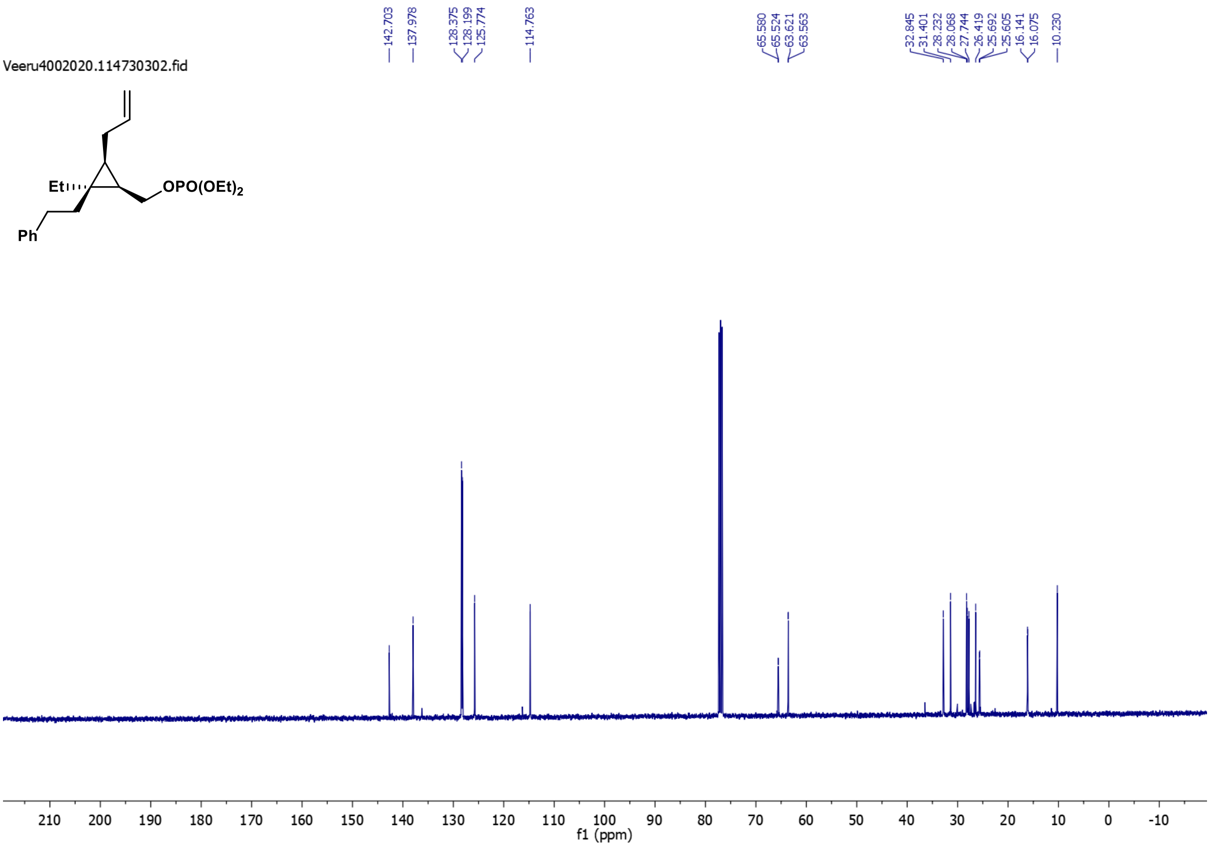
142.703 137.978 128.375 128.199 125.774 114.763 65.580 65.524 63.621 63.563 32.845 31.401 28.232 28.068 27.744 26.419 25.692 25.605 16.141 16.075 10.230
Veeru4002020.114730302.fid
Et
OPO(OEt)2
Ph
210 200 190 180 170 160 150 140 130 120 110 100 90 80 70 60 50 40 30 20 10 0 -10
f1 (ppm)

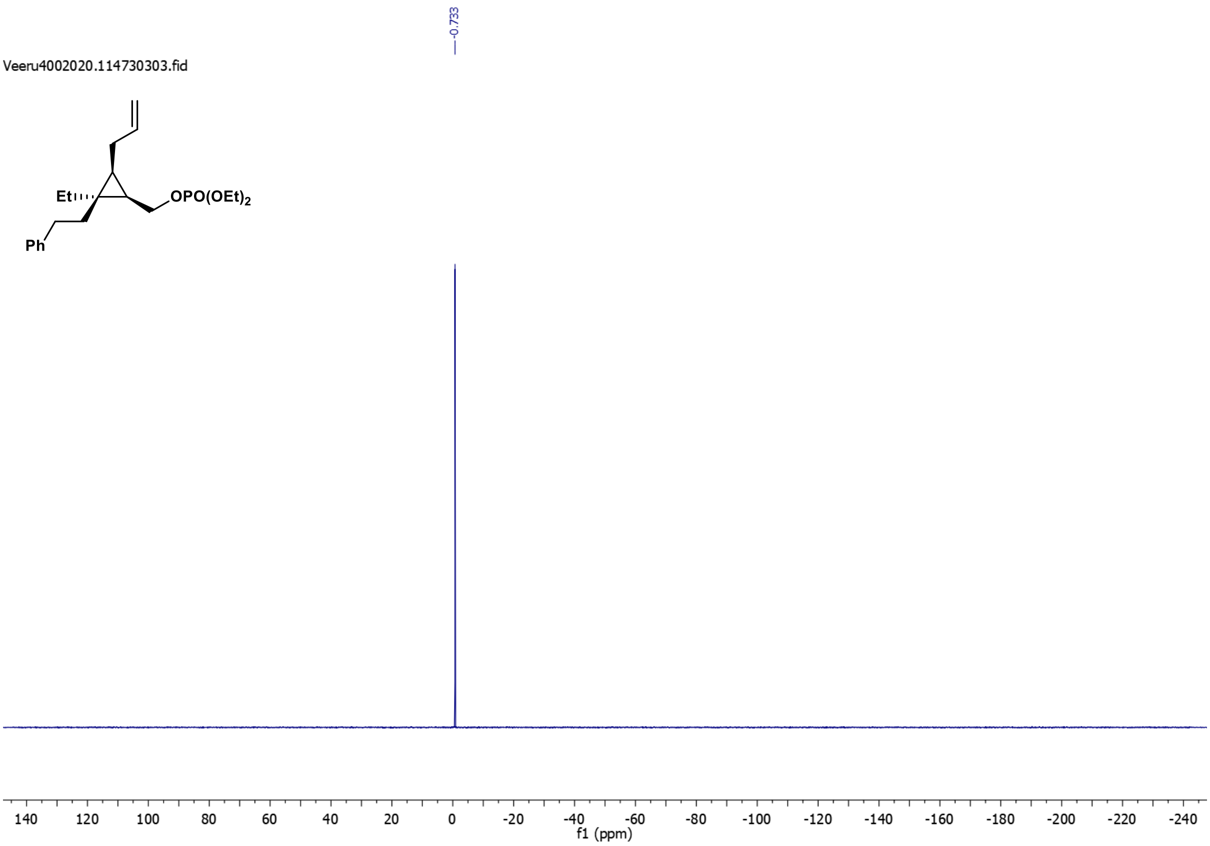
Veeru4002020.114730303.fid
—0.733
Et
OPO(OEt)2
Ph
f1 (ppm)

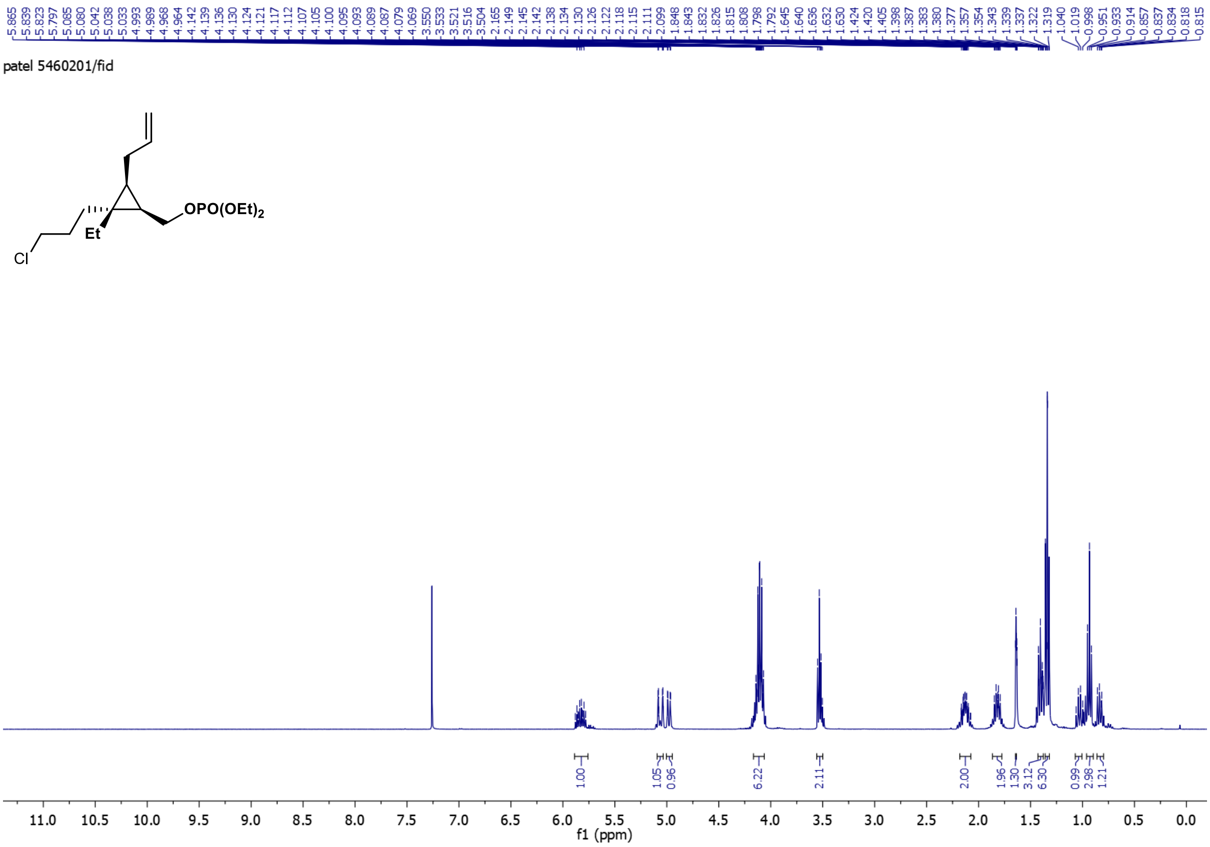
patel 5460201/fid
5.865 5.839 5.823 5.797 5.085 5.080 5.042 5.038 5.033 4.993 4.989 4.968 4.964 4.142 4.139 4.136 4.130 4.124 4.121 4.117 4.112 4.107 4.105 4.100 4.095 4.093 4.089 4.087 4.079 4.069 3.550 3.533 3.521 3.516 3.504 2.165 2.149 2.145 2.142 2.138 2.134 2.130 2.126 2.122 2.118 2.115 2.111 2.099 1.848 1.843 1.832 1.826 1.815 1.808 1.798 1.792 1.645 1.640 1.636 1.632 1.630 1.424 1.420 1.405 1.398 1.387 1.383 1.380 1.377 1.357 1.354 1.343 1.339 1.337 1.322 1.319 1.040 1.019 0.998 0.951 0.933 0.914 0.857 0.837 0.834 0.818 0.815
OPO(OEt)2
Et
Cl
1.00 1.05 0.96 6.22 2.11 2.00 1.96 1.30 3.12 6.30 0.99 2.98 1.21
f1 (ppm)

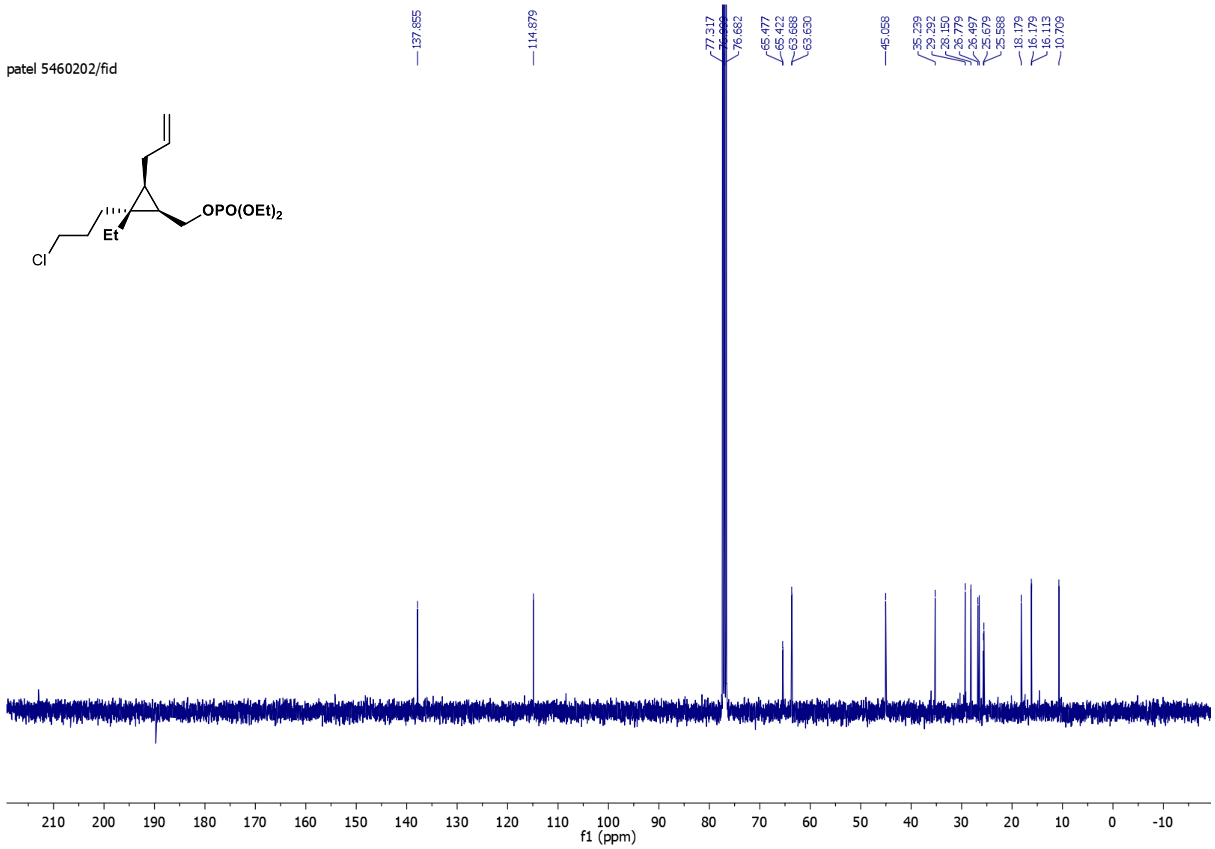
patel 5460202/fid
OPO(OEt)2
Et
Cl
137.855
114.879
77.317
76.682
65.477
65.422
63.688
63.630
45.058
35.239
29.292
28.150
26.779
26.497
25.679
25.588
18.179
16.179
16.113
10.709
f1 (ppm)

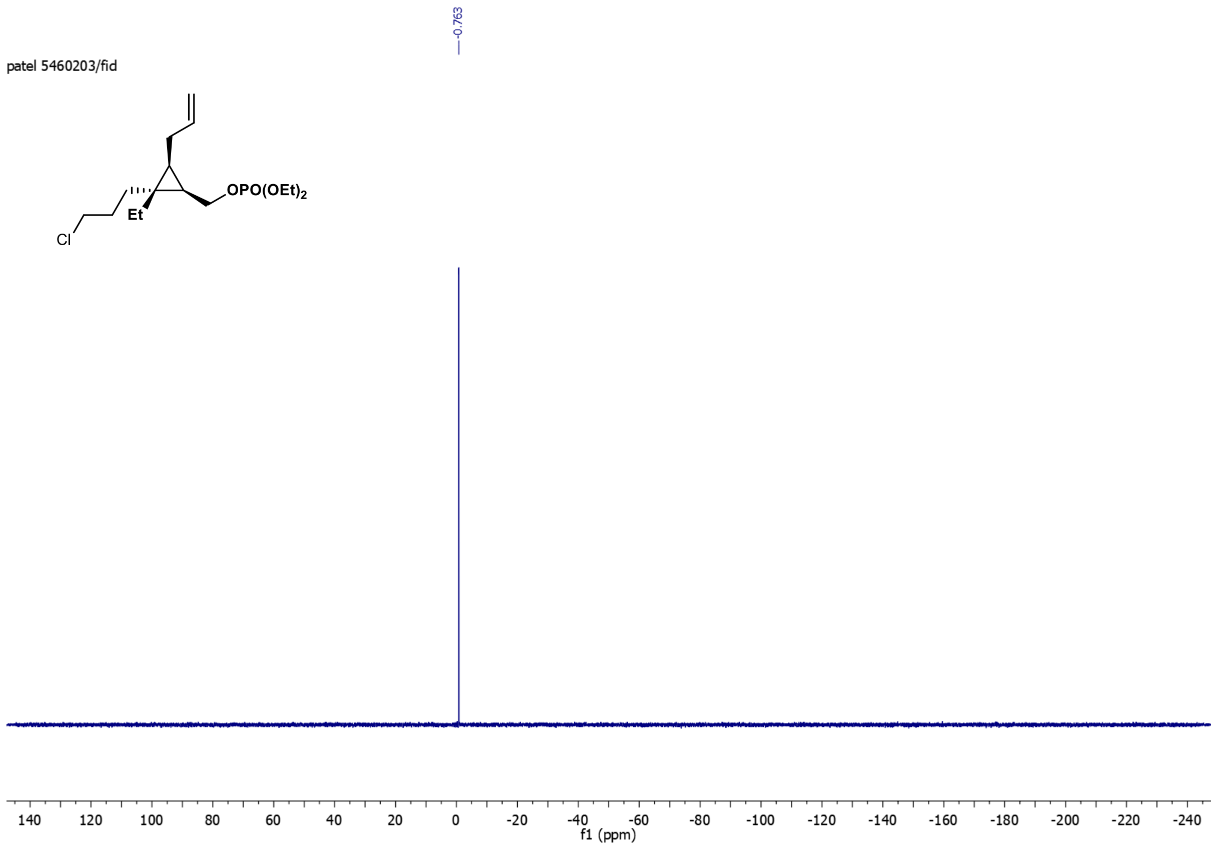
patel 5460203/fid
-0.763
OPO(OEt)2
Et
Cl
f1 (ppm)

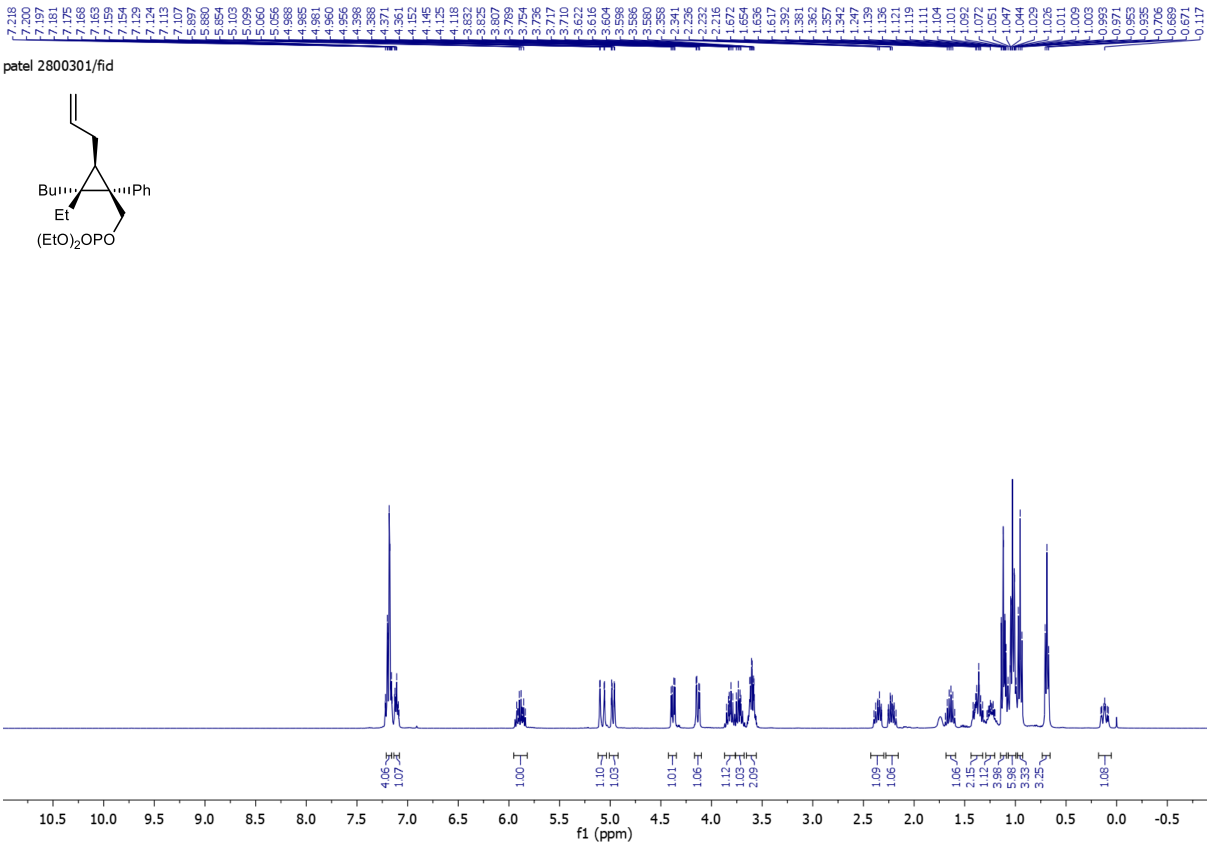
patel 2800301/fid
Bu
Ph
Et
(EtO)2OPO
f1 (ppm)

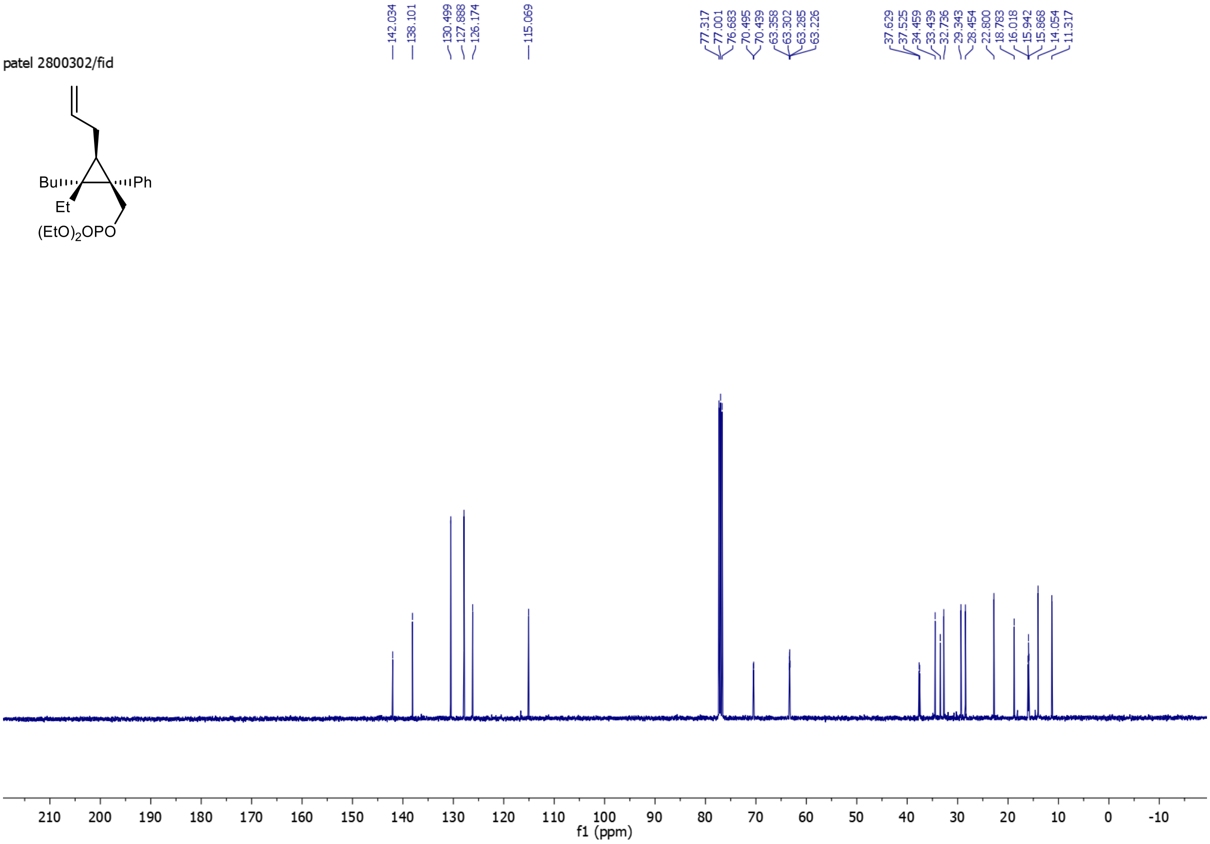
patel 2800302/fid
Bu
Ph
Et
(EtO)2OPO
f1 (ppm)

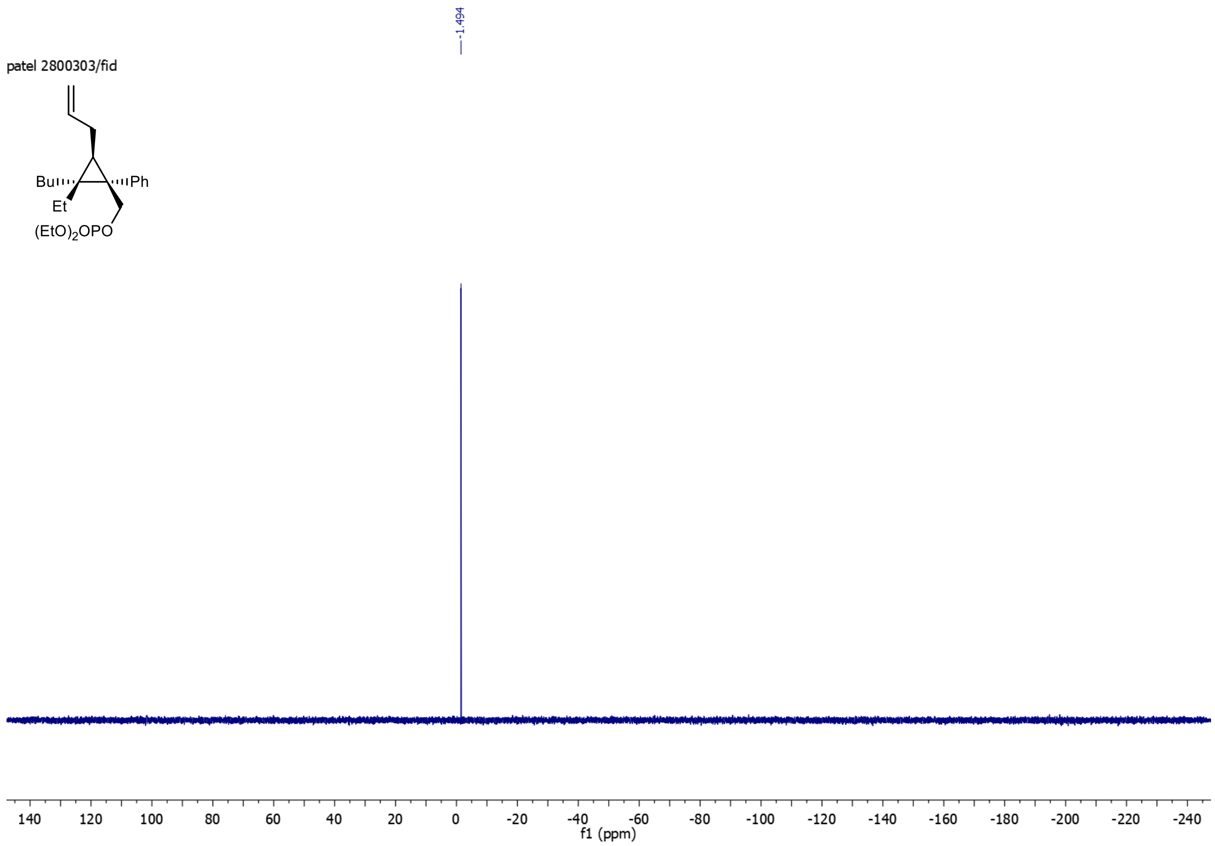

patel 2800303/fid
Bu
Ph
Et
(EtO)2OPO
-1.494
f1 (ppm)

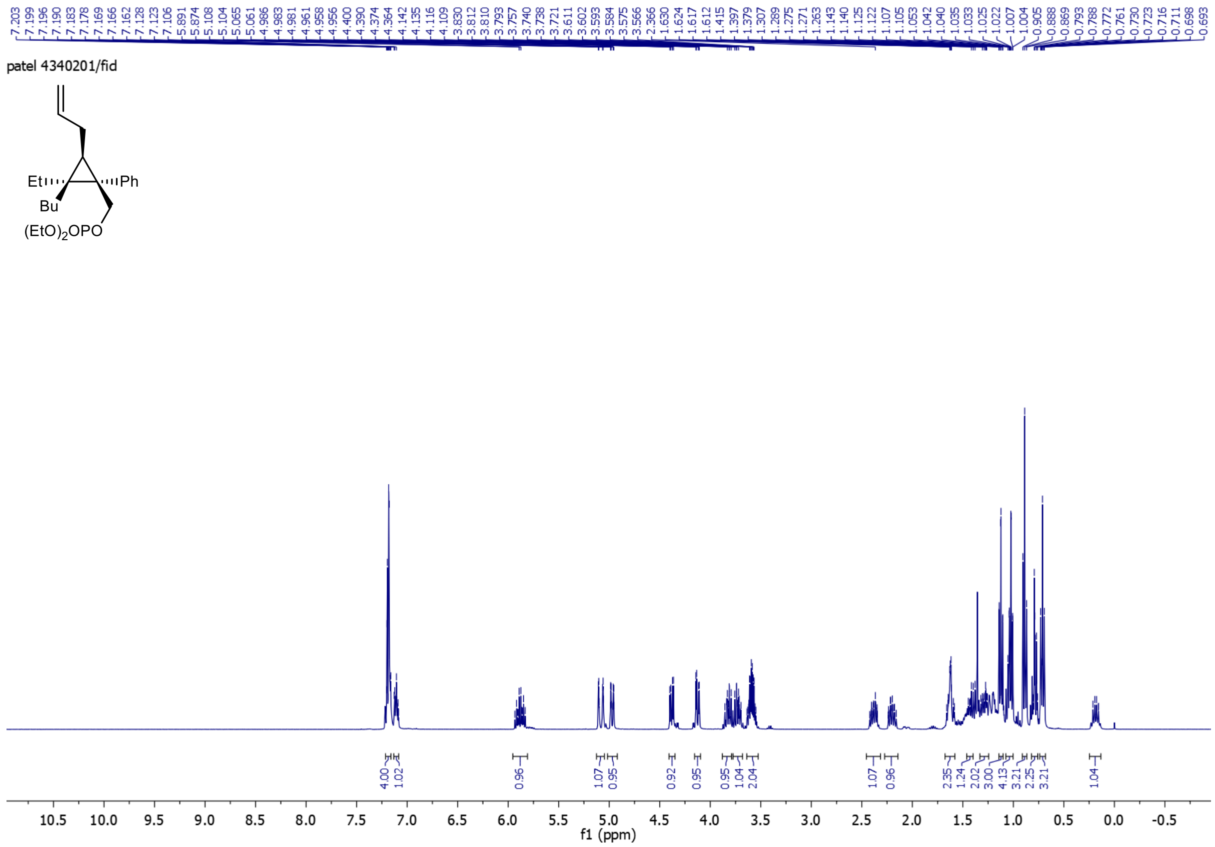

patel 4340201/fid
Et
Ph
Bu
(EtO)2OPO
f1 (ppm)

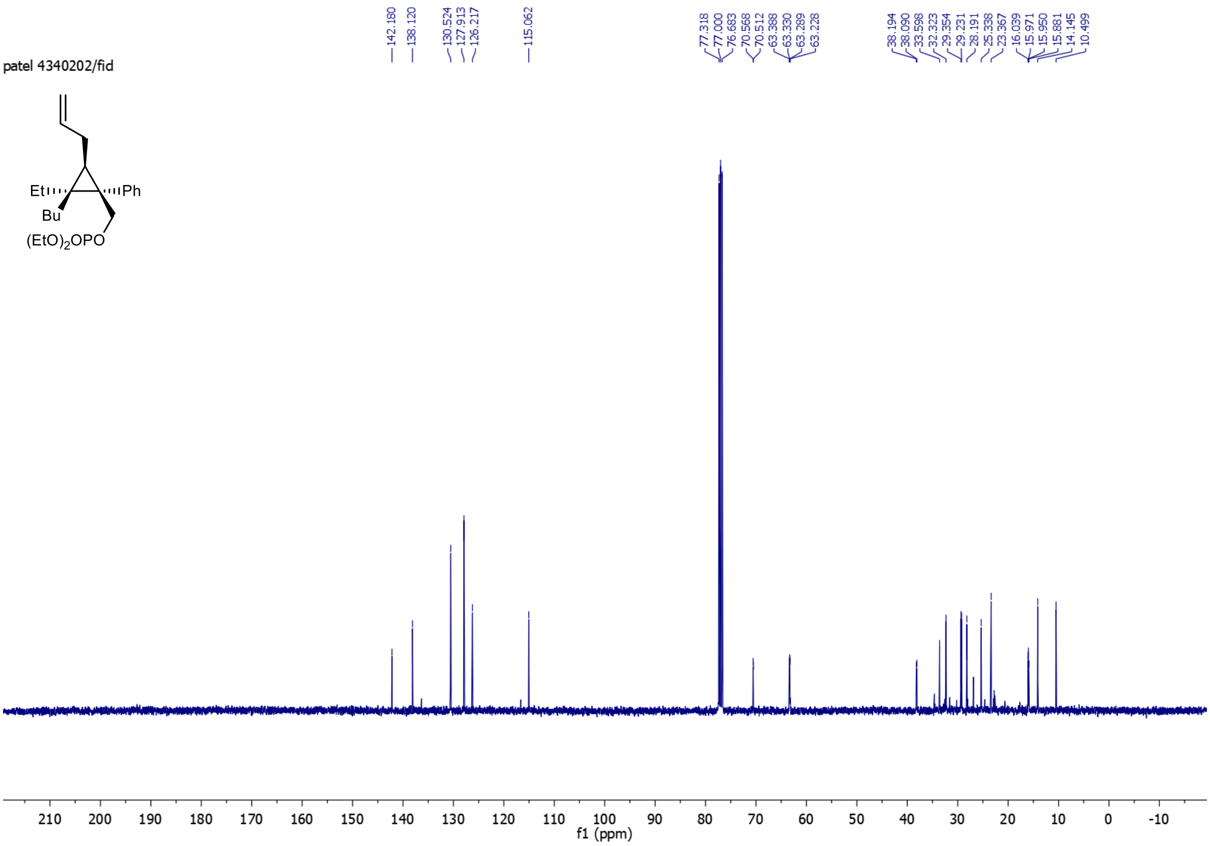
patel 4340202/fid
142.180
138.120
130.524
127.913
126.217
115.062
77.318
77.000
76.683
70.568
70.512
63.388
63.330
63.289
63.228
38.194
38.090
33.598
32.323
29.354
29.231
28.191
25.338
23.367
16.039
15.971
15.950
15.881
14.145
10.499
Et
Ph
Bu
(EtO)2OPO
f1 (ppm)

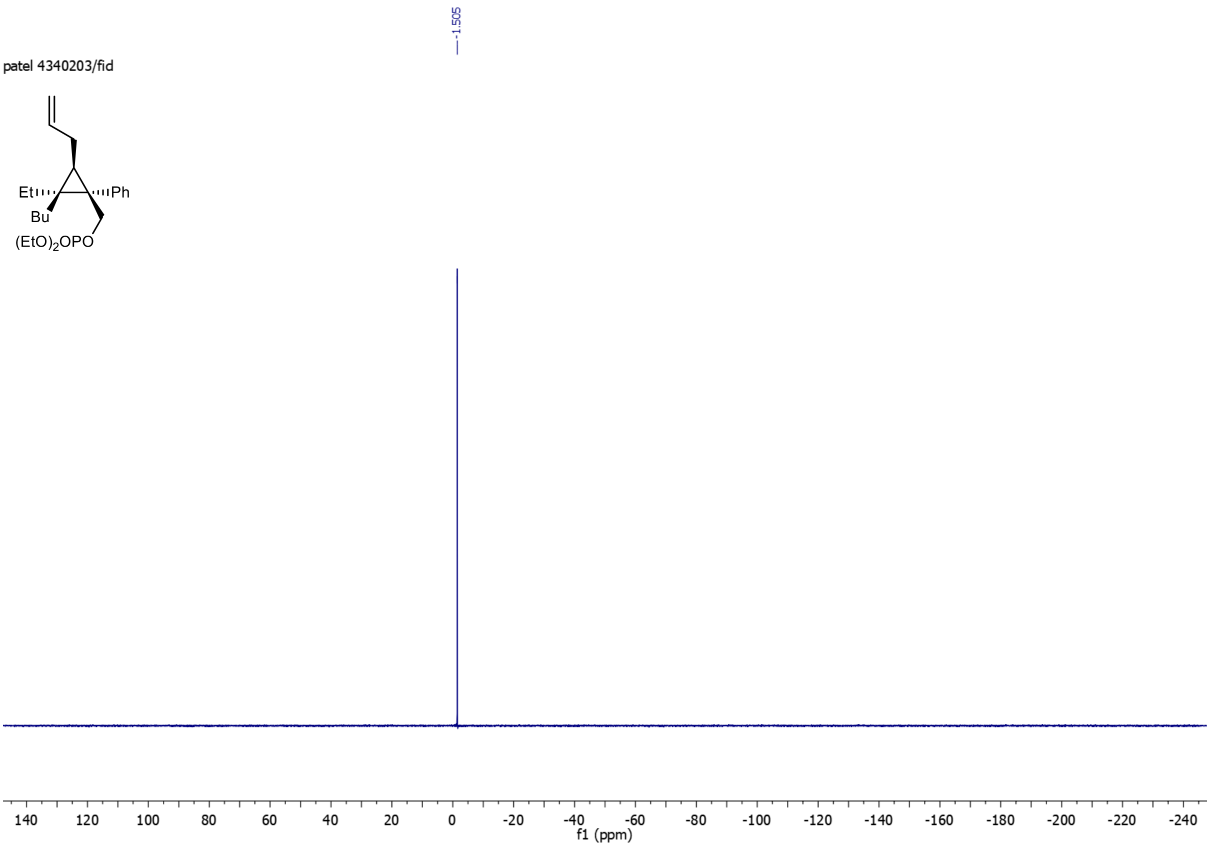
patel 4340203/fid
-1.505
Et
Ph
Bu
(EtO)2OPO
f1 (ppm)

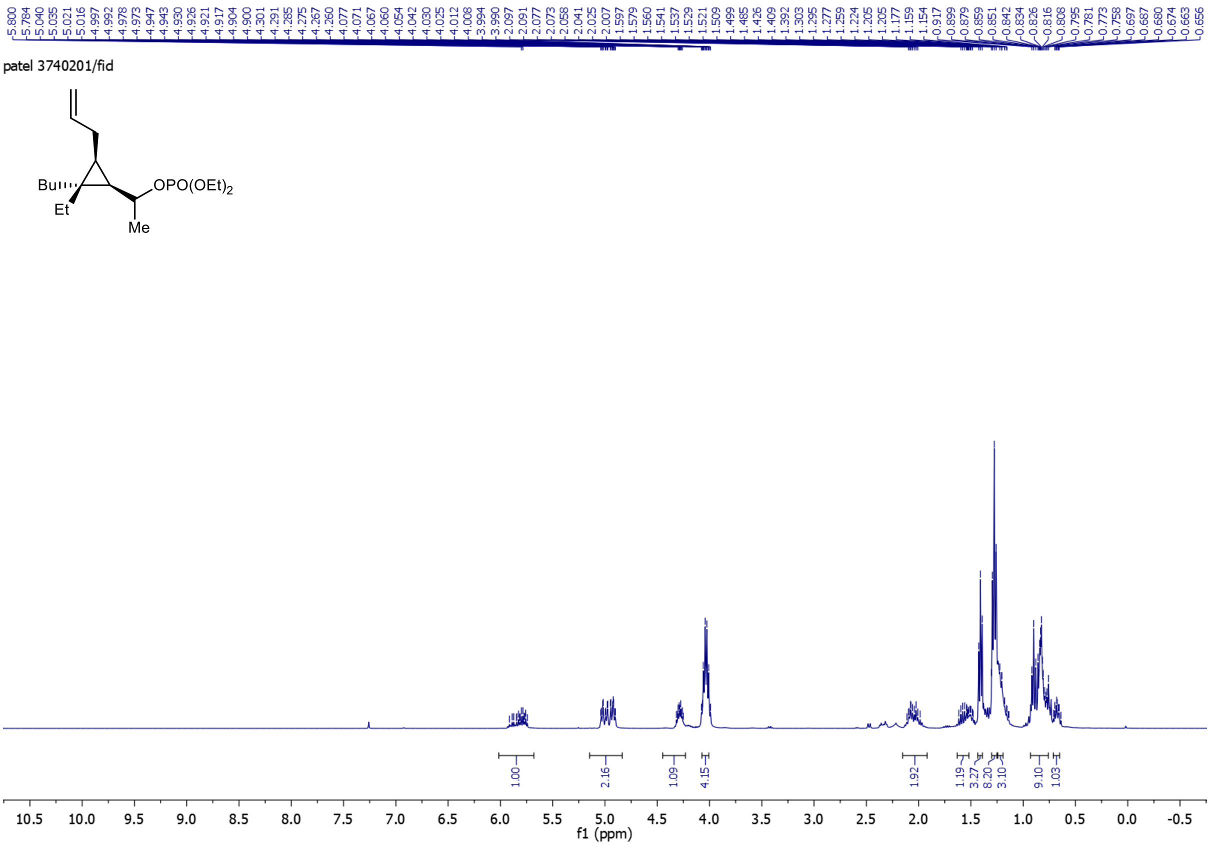
5.800 5.784 5.040 5.035 5.021 5.016 4.997 4.992 4.978 4.973 4.947 4.943 4.930 4.926 4.921 4.917 4.904 4.900 4.301 4.291 4.285 4.275 4.267 4.260 4.077 4.071 4.067 4.060 4.054 4.042 4.030 4.025 4.012 4.008 3.994 3.990 2.097 2.091 2.077 2.073 2.058 2.041 2.025 2.007 1.597 1.579 1.560 1.541 1.537 1.529 1.521 1.509 1.499 1.485 1.426 1.409 1.392 1.303 1.295 1.277 1.259 1.224 1.205 1.205 1.177 1.159 1.154 0.917 0.899 0.879 0.859 0.851 0.842 0.834 0.826 0.816 0.808 0.795 0.781 0.773 0.758 0.697 0.687 0.680 0.674 0.663 0.656
patel 3740201/fid
Bu
Et
OPO(OEt)$_2$
Me
1.00
2.16
1.09
4.15
1.92
1.19
3.27
8.20
3.10
9.10
1.03
10.5 10.0 9.5 9.0 8.5 8.0 7.5 7.0 6.5 6.0 5.5 5.0 4.5 4.0 3.5 3.0 2.5 2.0 1.5 1.0 0.5 0.0 -0.5
f1 (ppm)

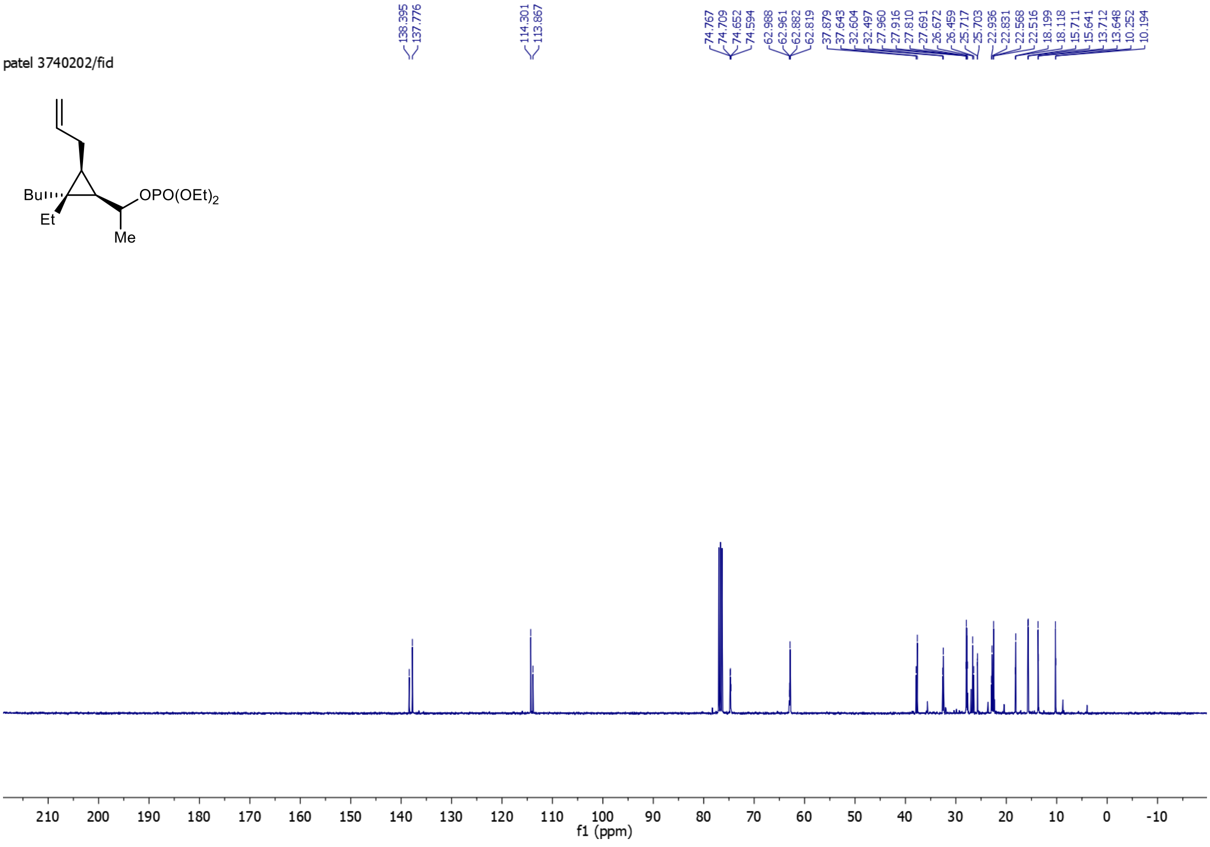
138.395 137.776
114.301 113.867
74.767 74.709 74.652 74.594 62.988 62.961 62.882 62.819 37.879 37.643 32.604 32.497 27.960 27.916 27.810 27.691 26.672 26.459 25.717 25.703 22.936 22.831 22.568 22.516 18.199 18.118 15.711 15.641 13.712 13.648 10.252 10.194
patel 3740202/fid
Bu
Et
OPO(OEt)$_2$
Me
210 200 190 180 170 160 150 140 130 120 110 100 90 80 70 60 50 40 30 20 10 0 -10
f1 (ppm)

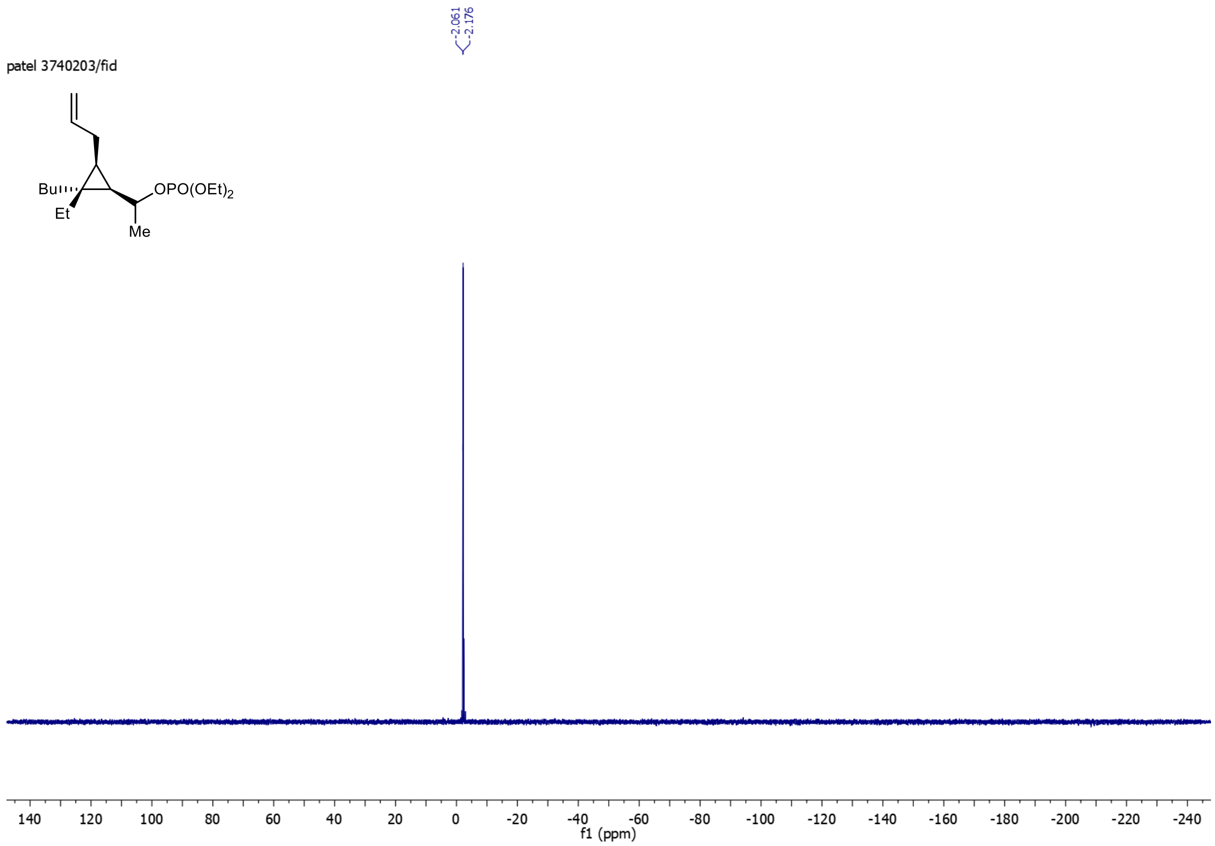
-2.061
-2.176
patel 3740203/fid
Bu
OPO(OEt)2
Et
Me
f1 (ppm)

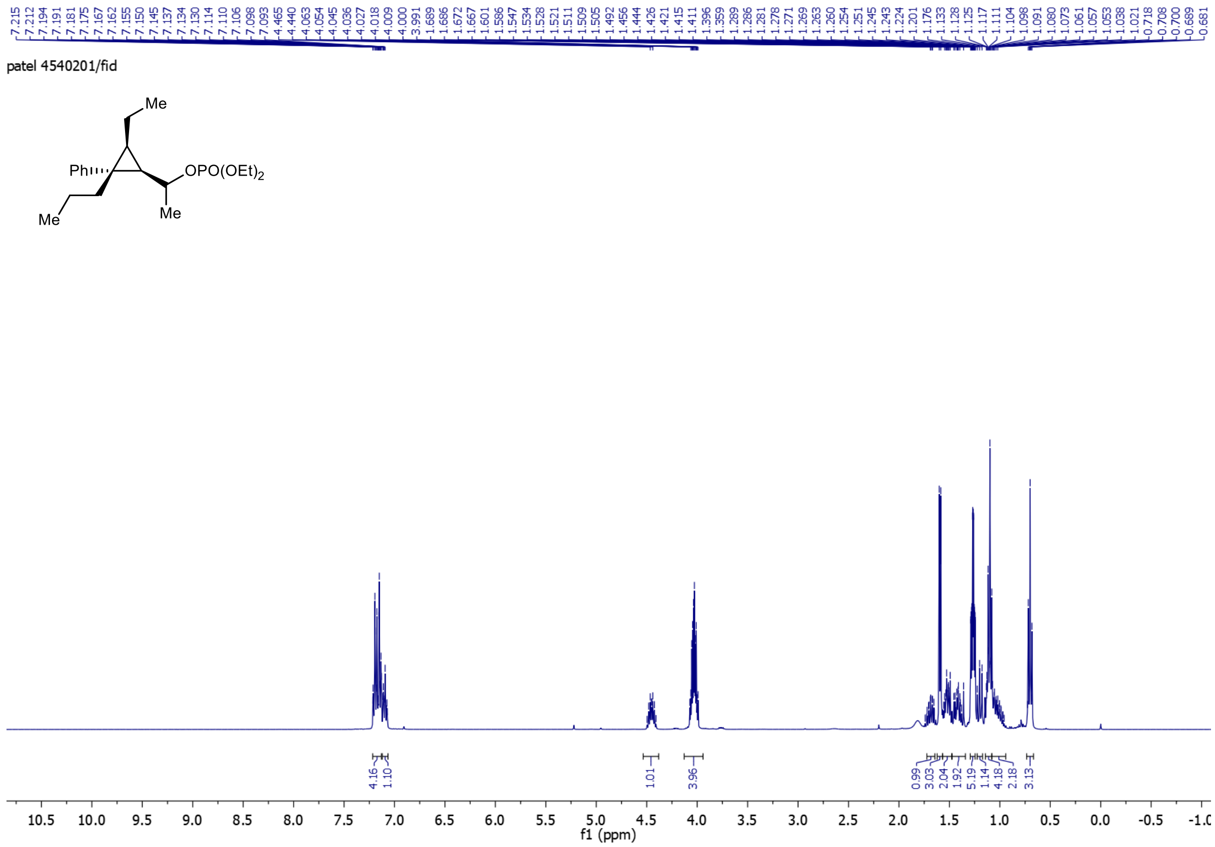
patel 4540201/fid
Me
Ph
OPO(OEt)2
Me
Me
f1 (ppm)

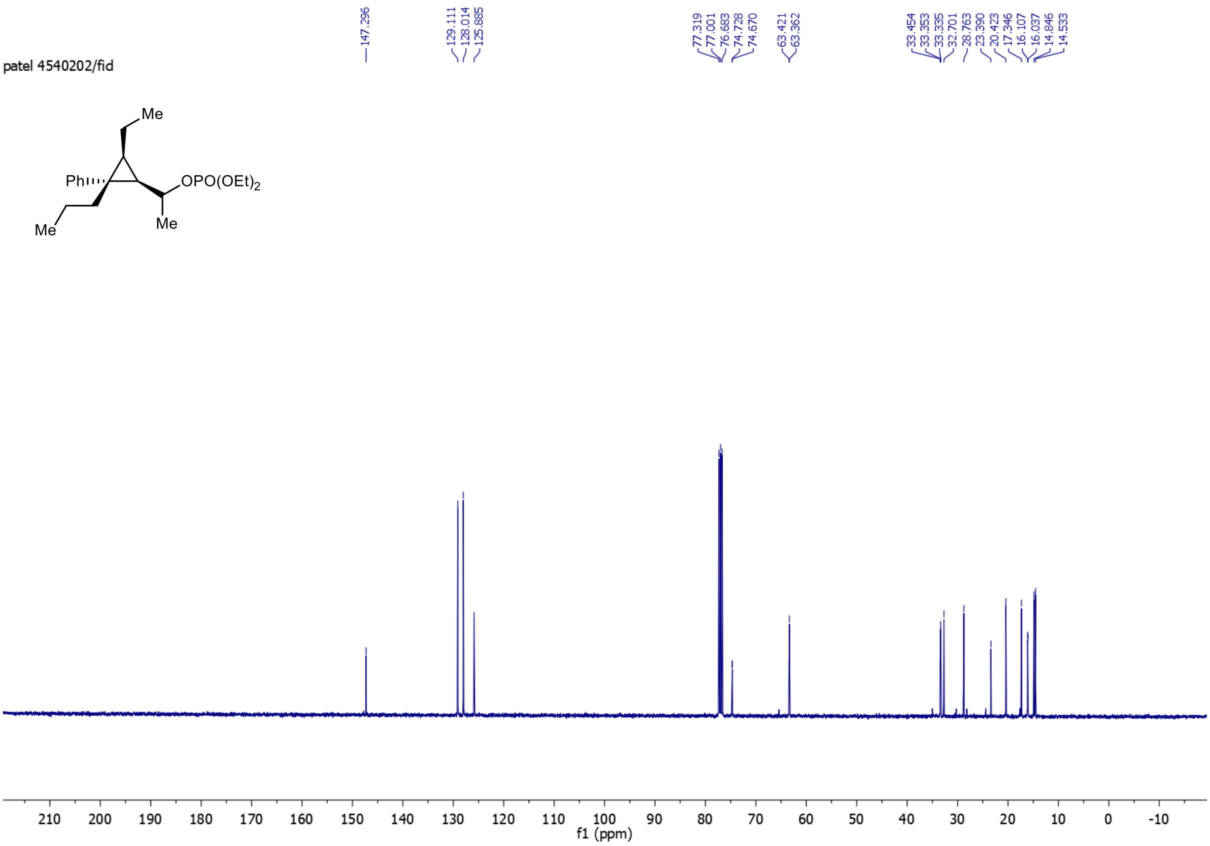
patel 4540202/fid
147.296
129.111
128.014
125.885
77.319
77.001
76.683
74.728
74.670
63.421
63.362
33.454
33.353
33.335
32.701
28.763
23.390
20.423
17.346
16.107
16.037
14.846
14.533
Me
Ph
OPO(OEt)2
Me
Me
210 200 190 180 170 160 150 140 130 120 110 100 90 80 70 60 50 40 30 20 10 0 -10
f1 (ppm)

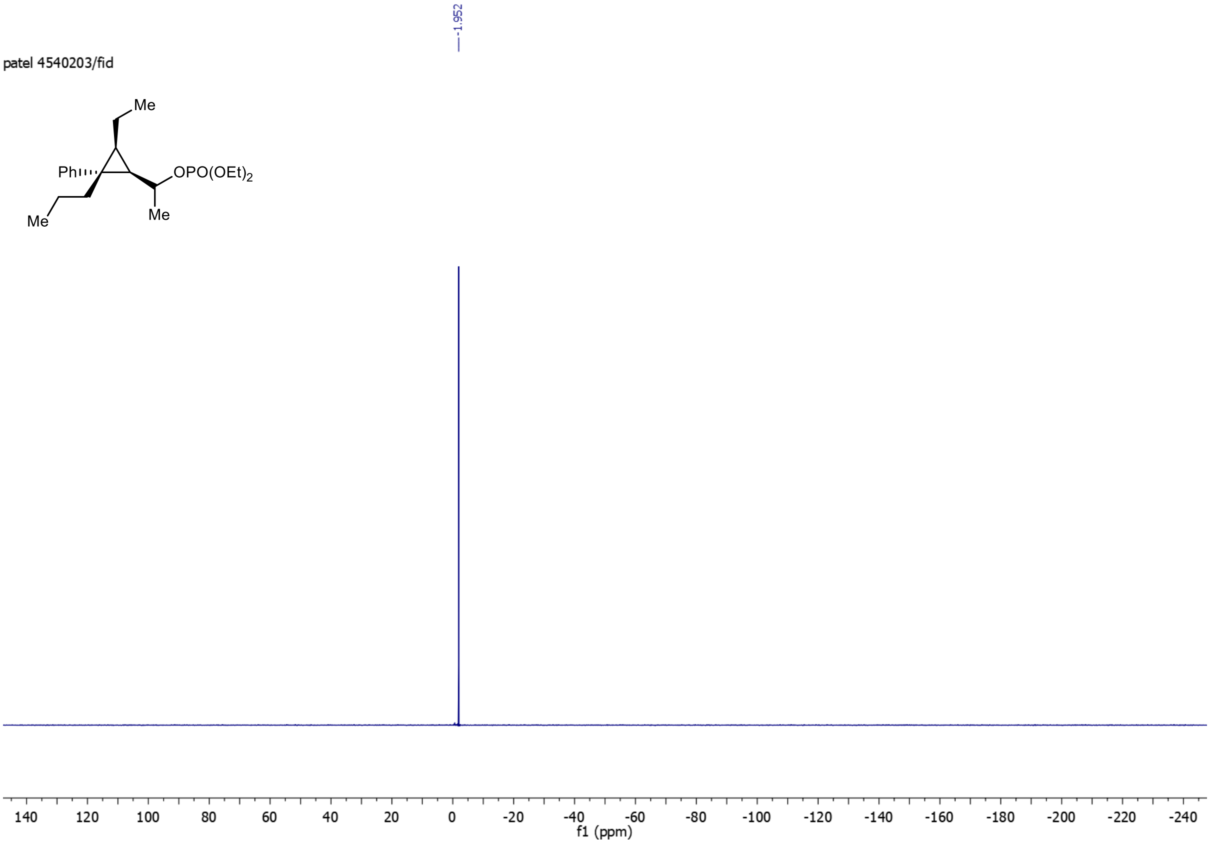
patel 4540203/fid
-1.952
Me
Ph
OPO(OEt)2
Me
Me
140 120 100 80 60 40 20 0 -20 -40 -60 -80 -100 -120 -140 -160 -180 -200 -220 -240
f1 (ppm)

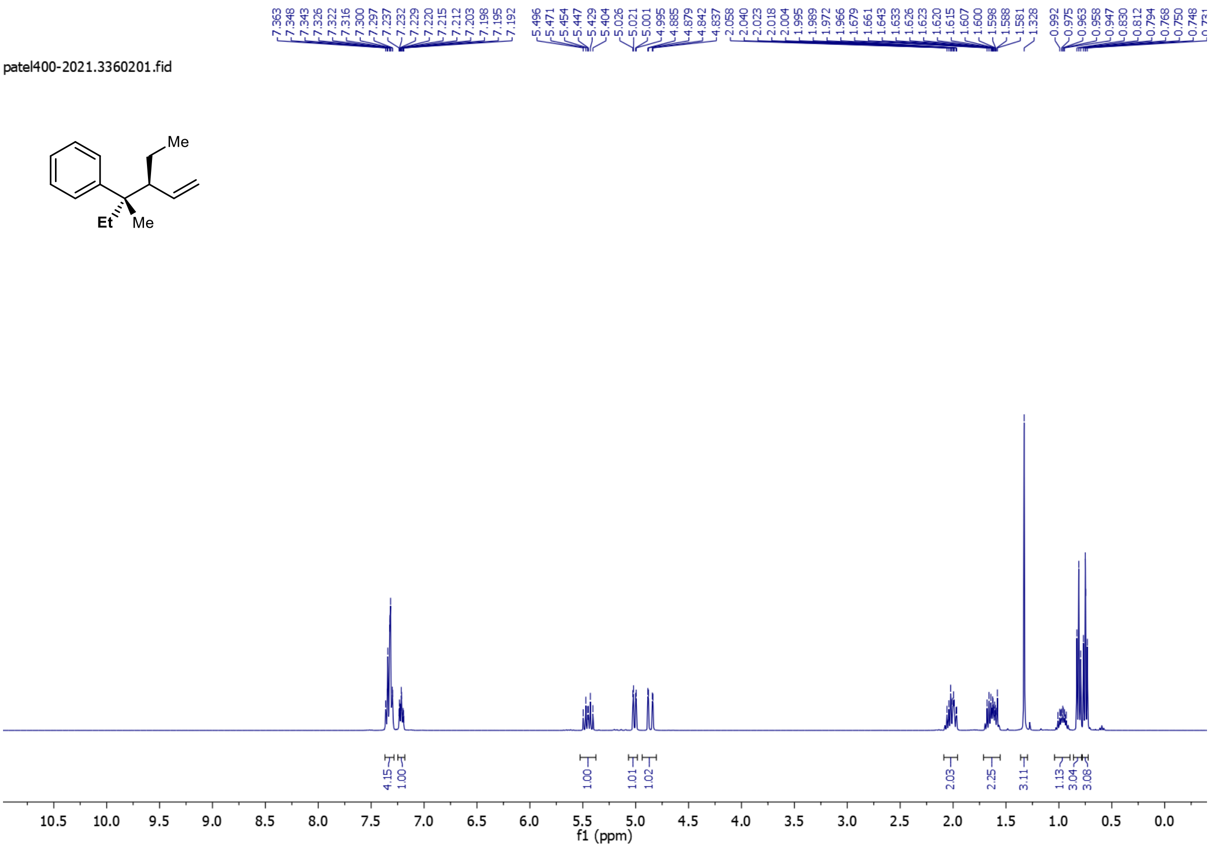
patel400-2021.3360201.fid
Me
Et
Me
f1 (ppm)

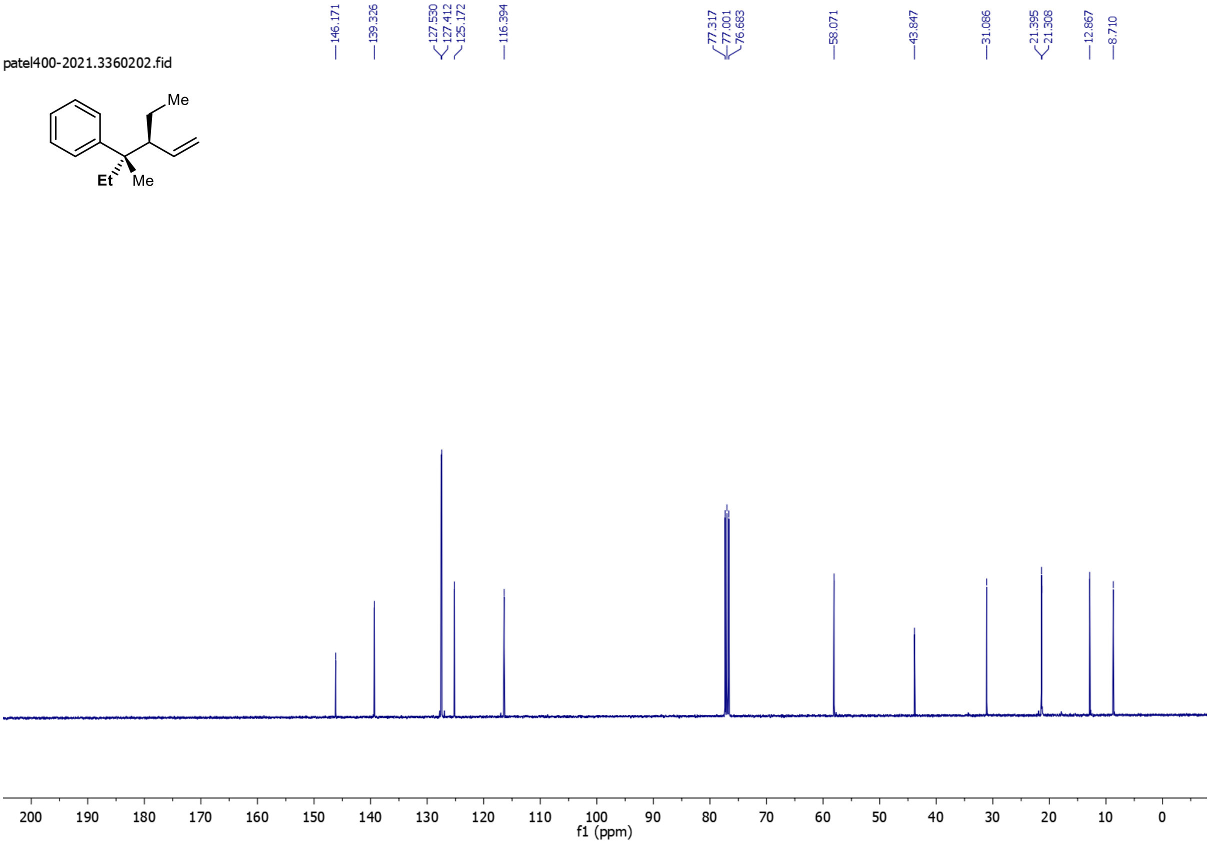
patel400-2021.3360202.fid
Me
Et
Me
f1 (ppm)

patel400-2021.3900201.fid

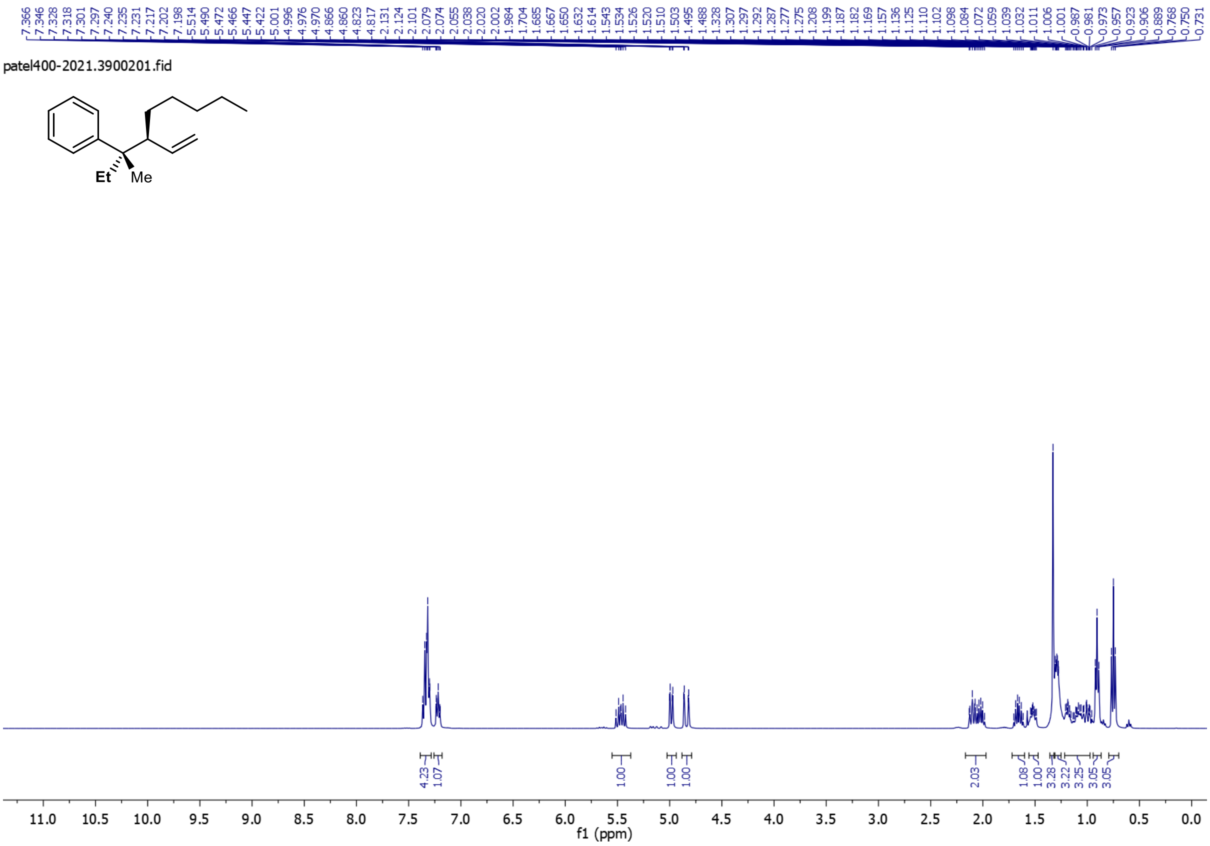

patel400-2021.3900202.fid

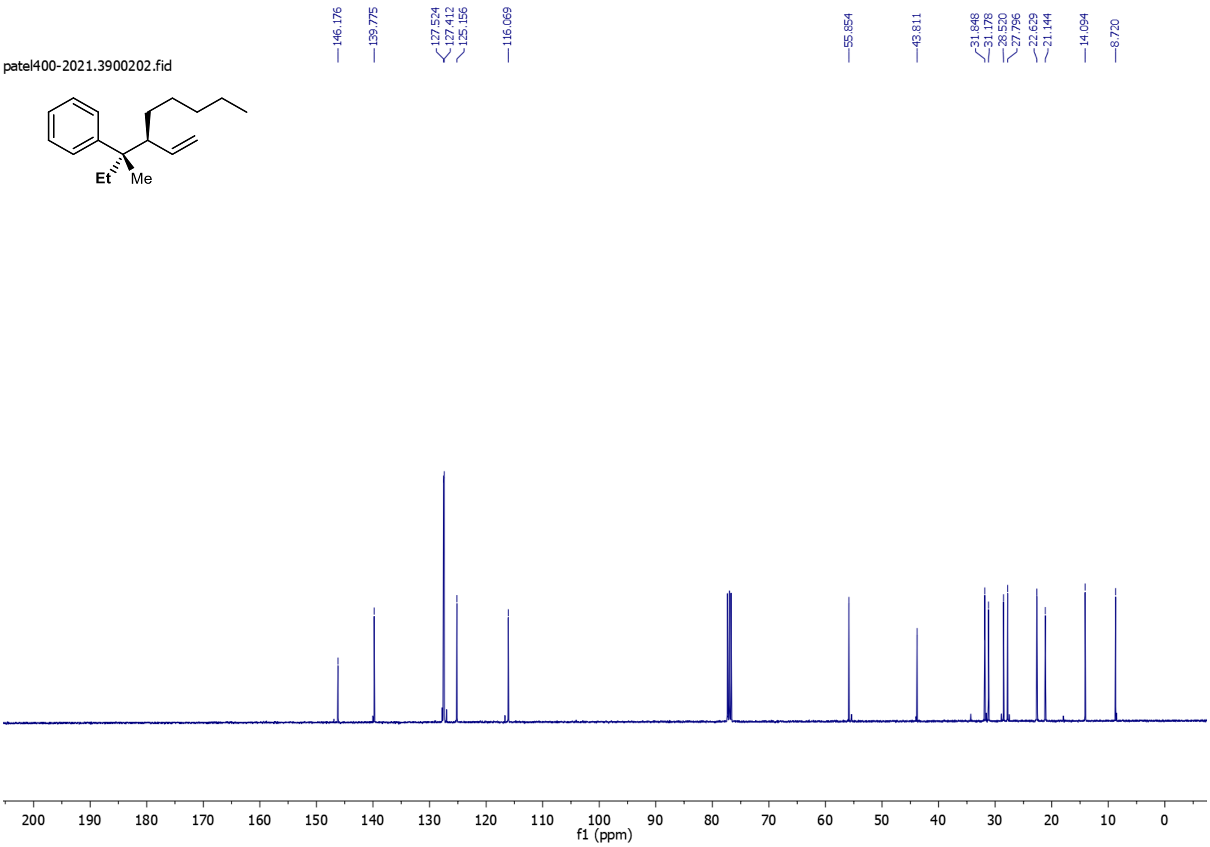

patel400-2021.3890201.fid

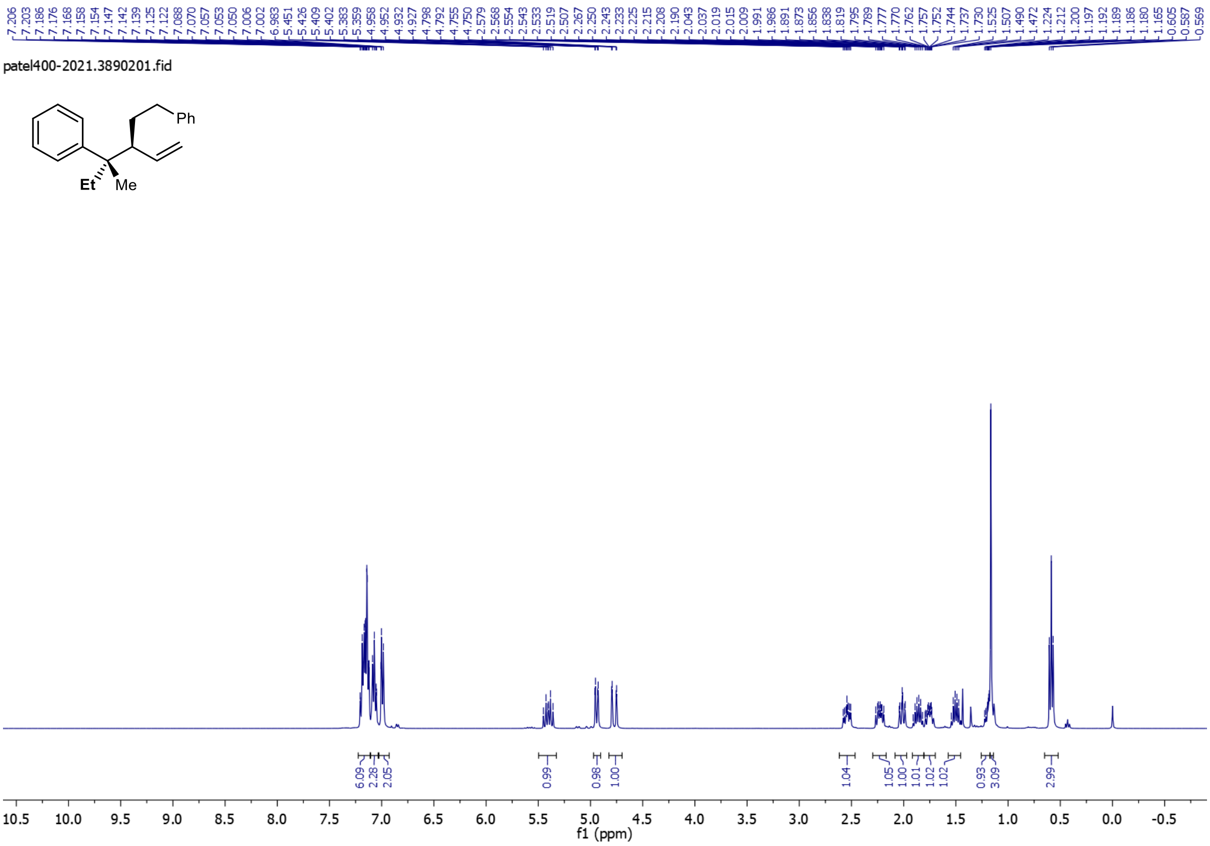

patel400-2021.3890202.fid

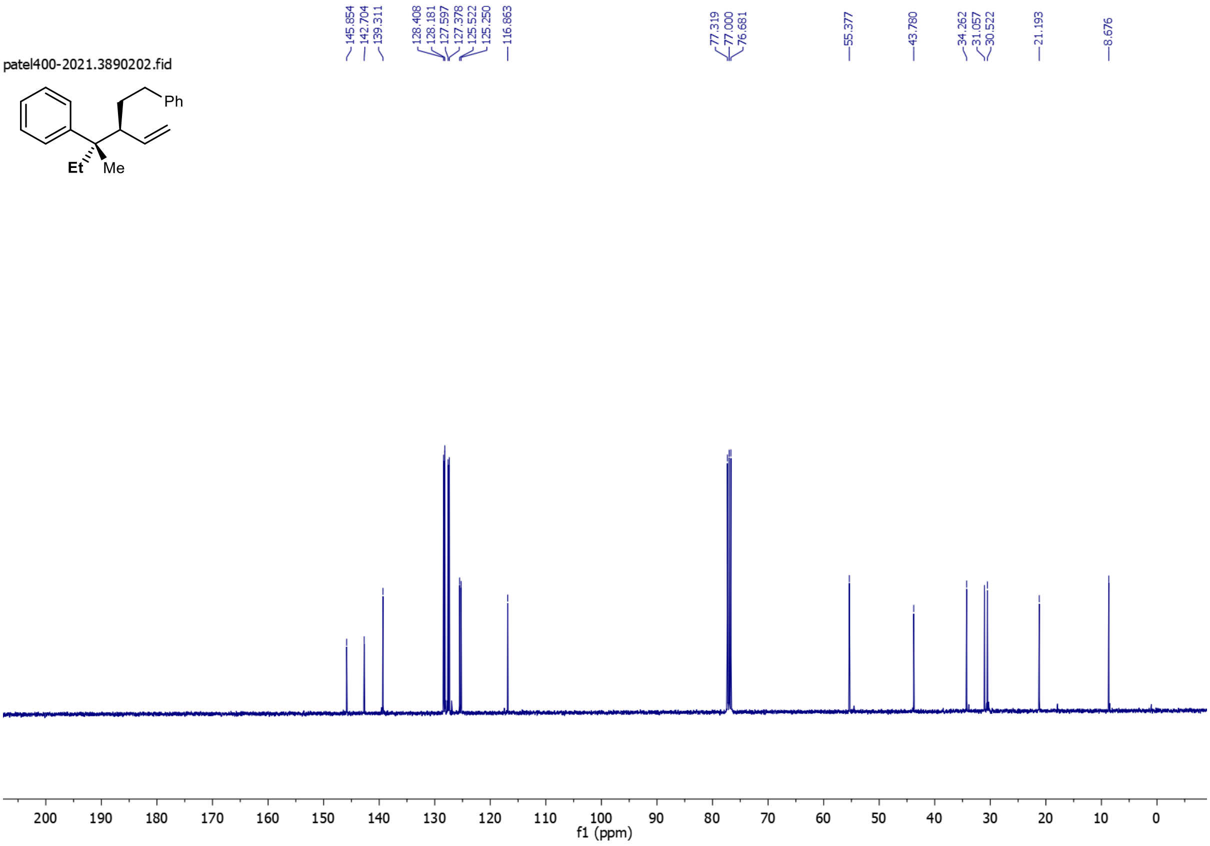

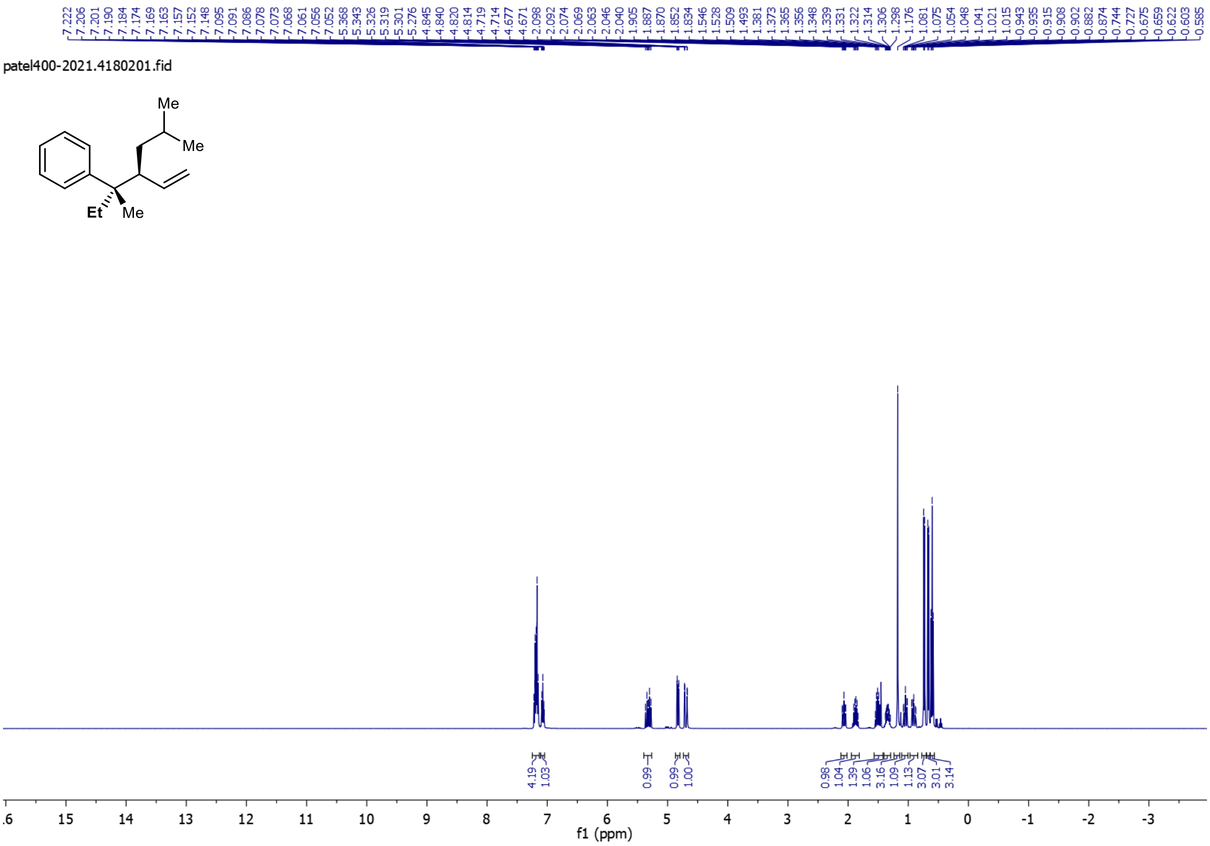
7.222 7.206 7.201 7.190 7.184 7.174 7.169 7.163 7.157 7.152 7.148 7.095 7.091 7.086 7.078 7.073 7.068 7.061 7.056 7.052 5.368 5.343 5.326 5.319 5.301 5.276 4.845 4.840 4.820 4.814 4.719 4.714 4.677 4.671 2.098 2.092 2.074 2.069 2.063 2.046 2.040 1.905 1.887 1.870 1.852 1.834 1.546 1.528 1.509 1.493 1.381 1.373 1.365 1.356 1.348 1.339 1.331 1.322 1.314 1.306 1.298 1.176 1.081 1.075 1.054 1.048 1.041 1.021 1.015 0.943 0.935 0.915 0.908 0.902 0.882 0.874 0.744 0.727 0.675 0.659 0.622 0.603 0.585
patel400-2021.4180201.fid
Me
Me
Et
Me
4.19
1.03
0.99
0.99
1.00
0.98
1.04
1.39
1.06
3.16
1.09
1.13
3.07
3.01
3.14
f1 (ppm)

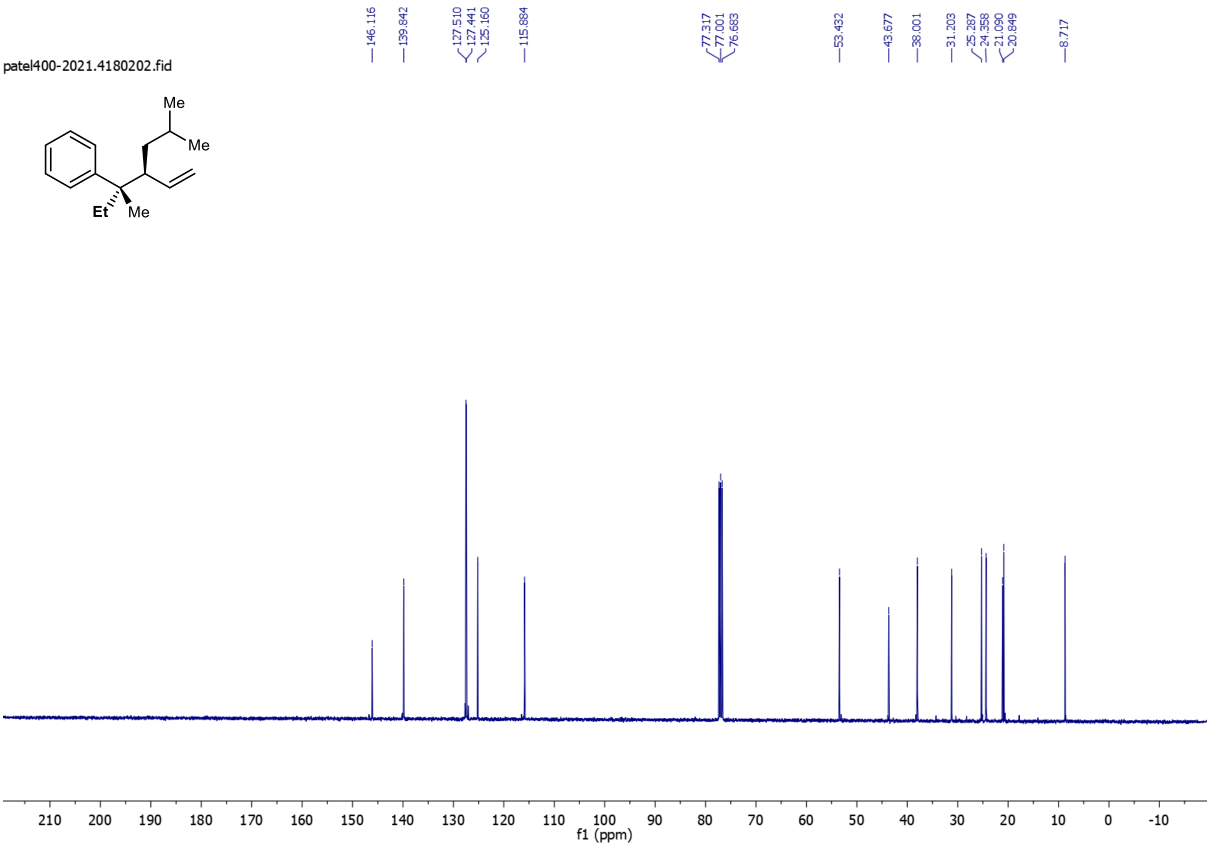
146.116
139.842
127.510
127.441
125.160
115.884
77.317
77.001
76.683
53.432
43.677
38.001
31.203
25.287
24.358
21.090
20.849
8.717
patel400-2021.4180202.fid
Me
Me
Et
Me
f1 (ppm)

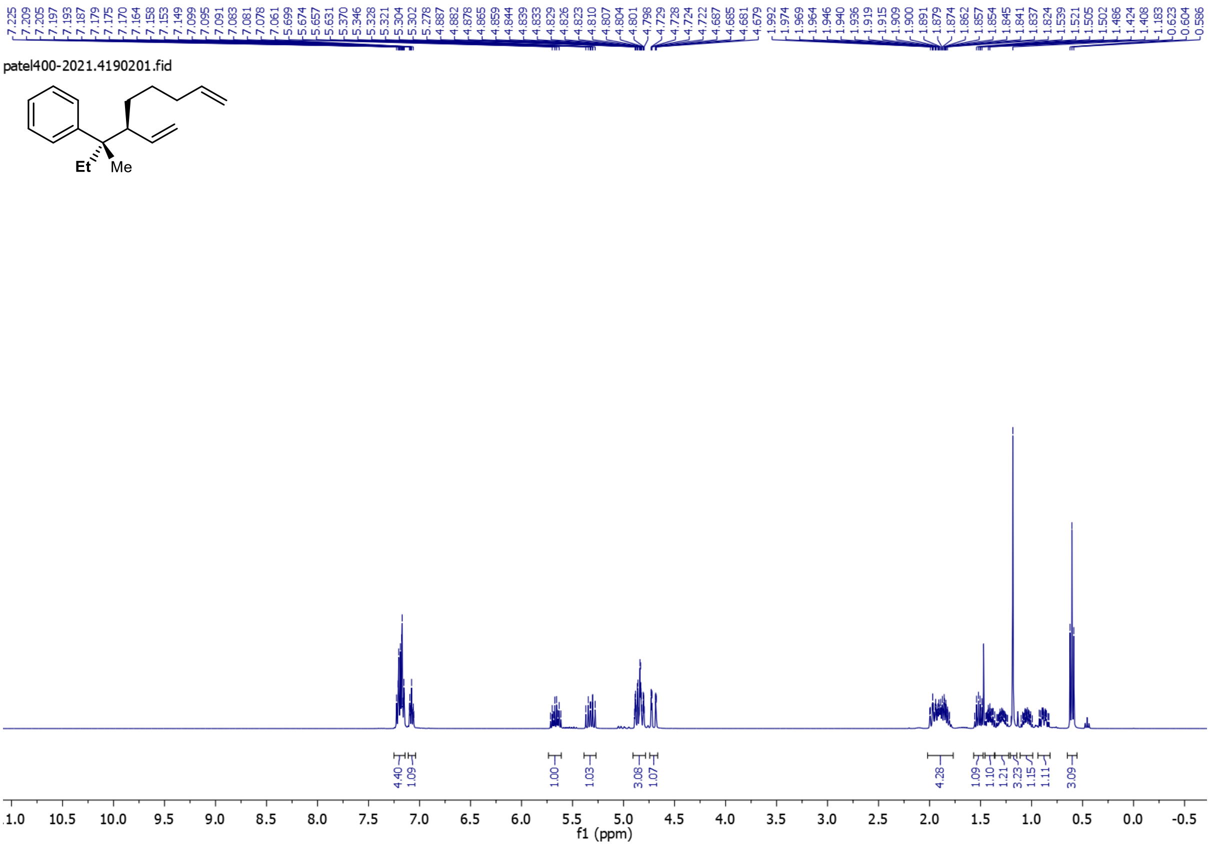
7.225 7.209 7.205 7.197 7.193 7.187 7.179 7.175 7.170 7.164 7.158 7.153 7.149 7.099 7.095 7.091 7.083 7.081 7.078 7.061 5.699 5.674 5.657 5.631 5.370 5.346 5.328 5.321 5.304 5.302 5.278 4.887 4.882 4.878 4.865 4.859 4.844 4.839 4.833 4.829 4.826 4.823 4.810 4.807 4.804 4.801 4.798 4.729 4.728 4.724 4.722 4.687 4.685 4.681 4.679 1.992 1.974 1.969 1.964 1.946 1.940 1.936 1.919 1.915 1.909 1.900 1.891 1.879 1.874 1.862 1.857 1.854 1.845 1.841 1.837 1.824 1.539 1.521 1.505 1.502 1.486 1.424 1.408 1.183 0.623 0.604 0.586
patel400-2021.4190201.fid
Et
Me
4.40
1.09
1.00
1.03
3.08
1.07
4.28
1.09
1.10
1.21
3.23
1.15
1.11
3.09
f1 (ppm)

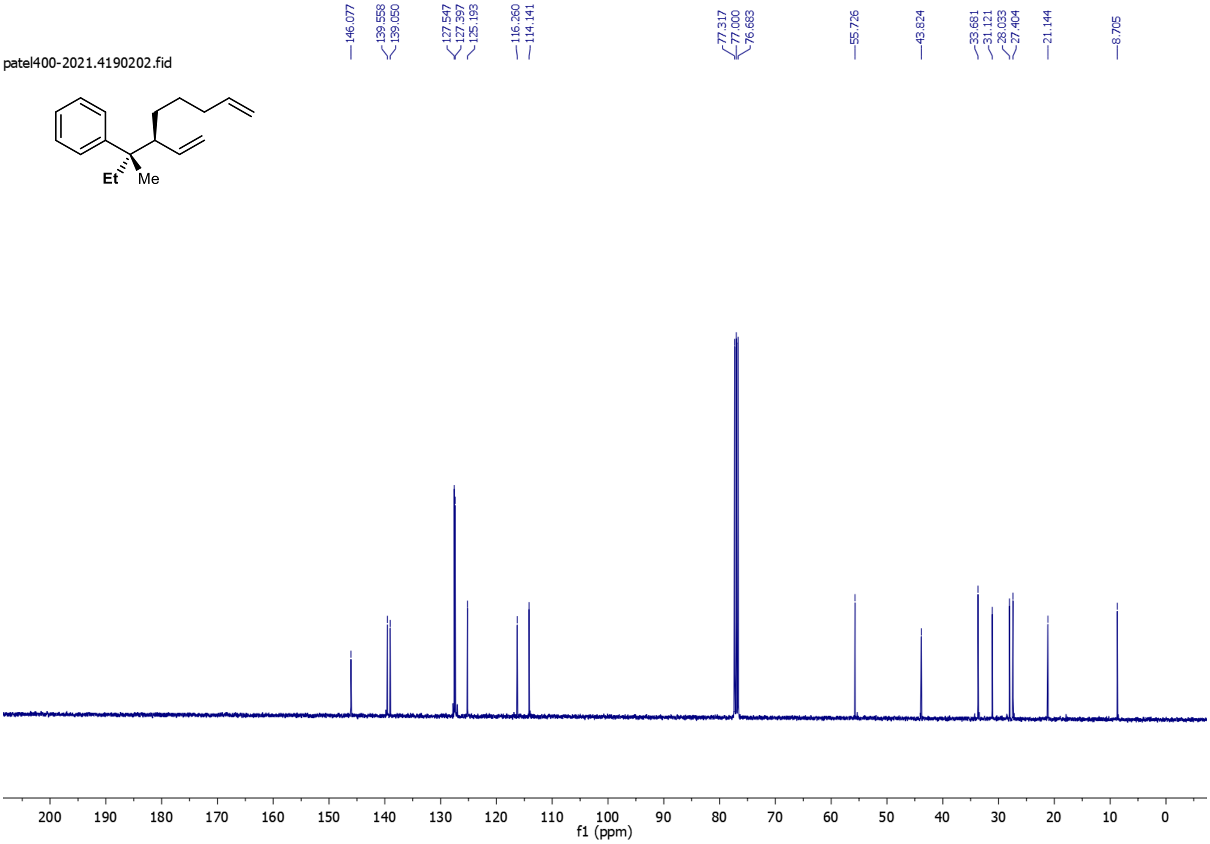
146.077 139.558 139.050 127.547 127.397 125.193 116.260 114.141 77.317 77.000 76.683 55.726 43.824 33.681 31.121 28.033 27.404 21.144 8.705
patel400-2021.4190202.fid
Et
Me
f1 (ppm)

patel400-2021.4240201.fid

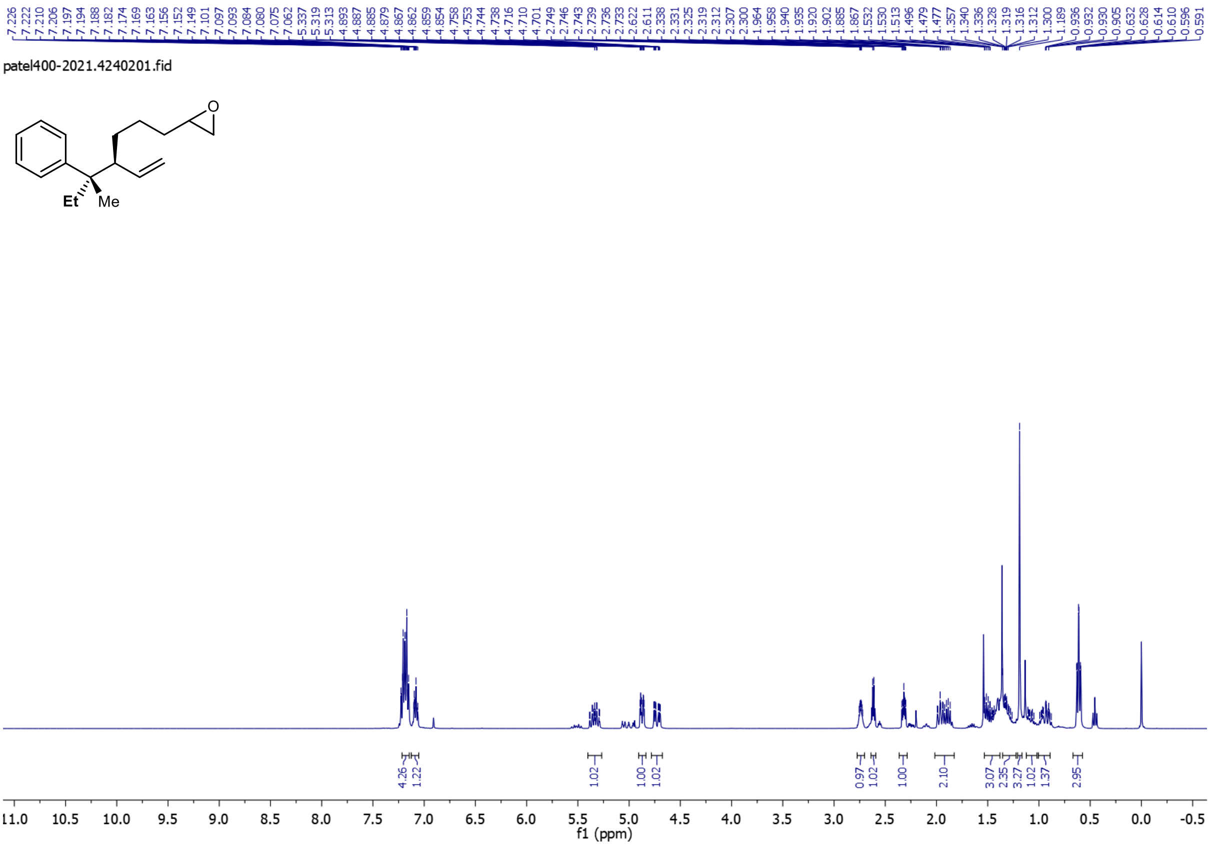

patel400-2021.4240202.fid

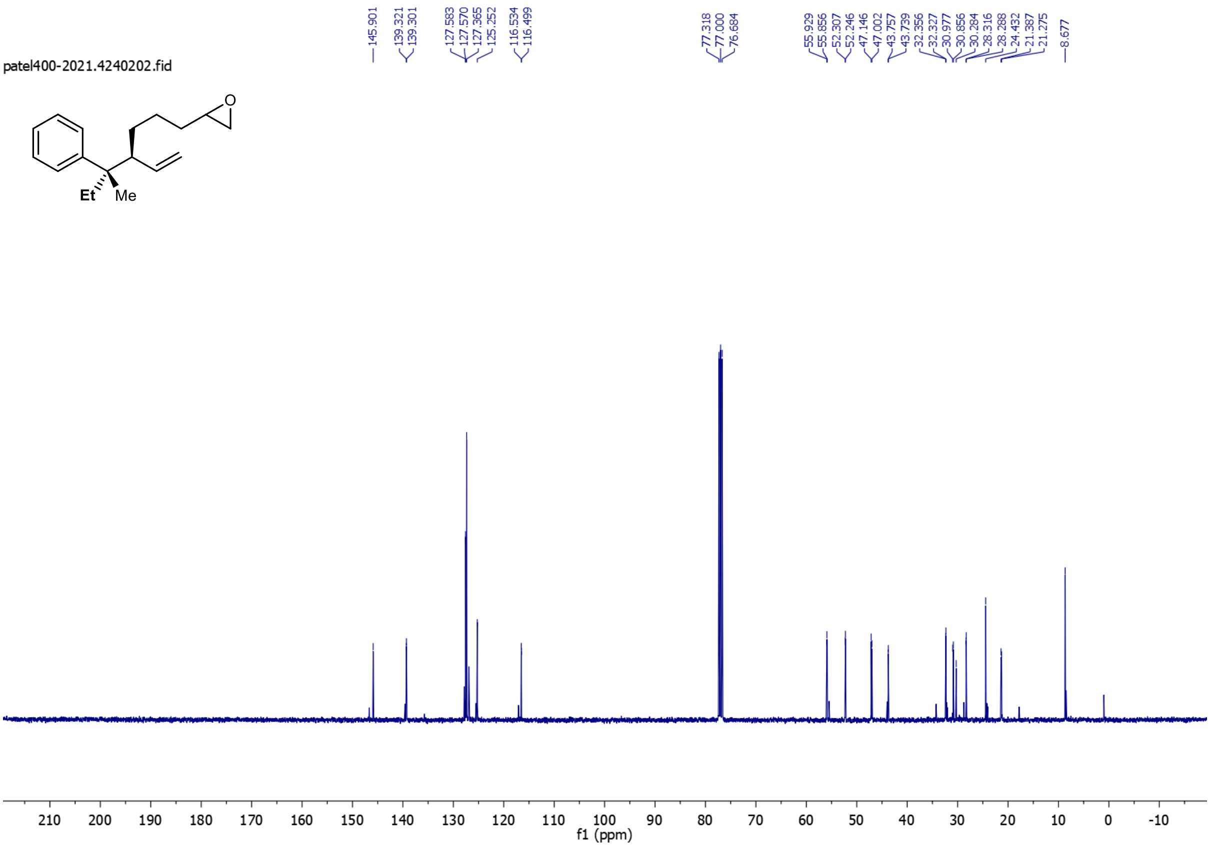

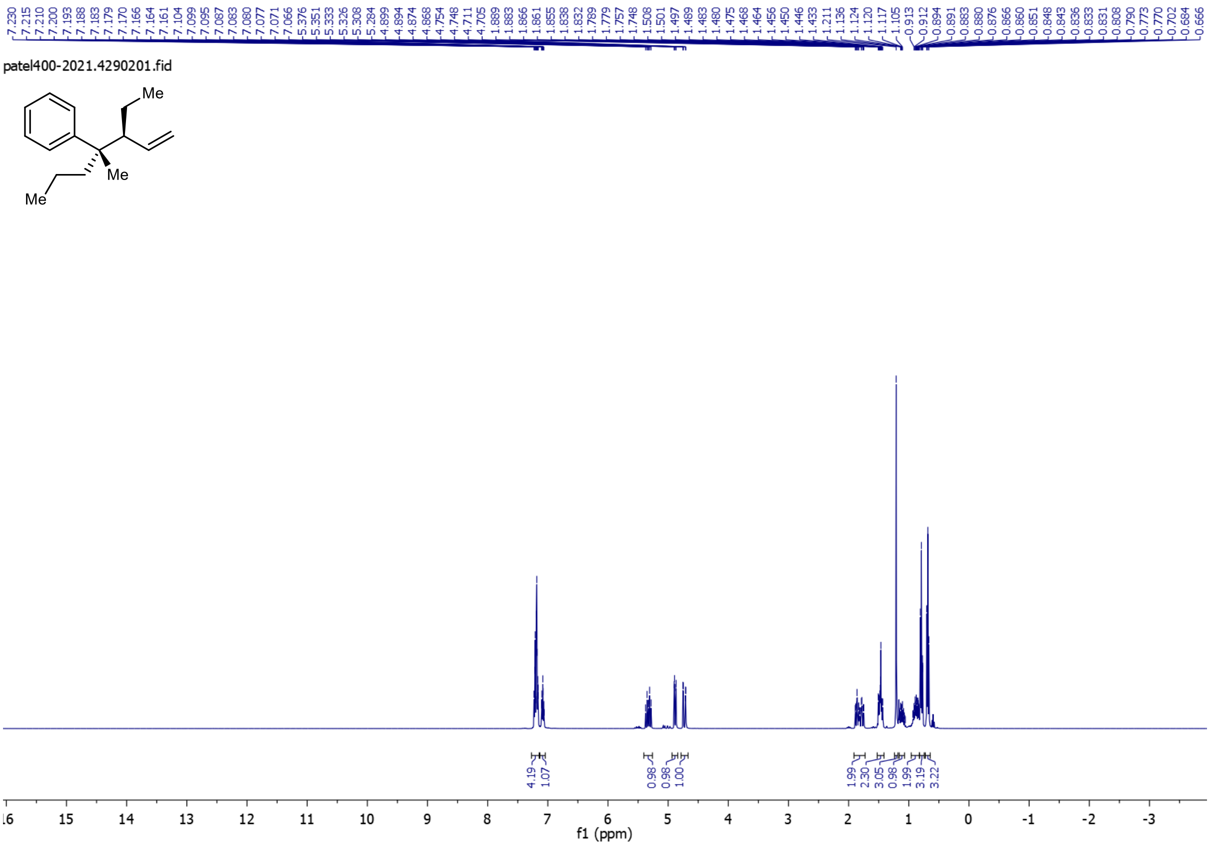
patel400-2021.4290201.fid
Me
Me
Me
f1 (ppm)

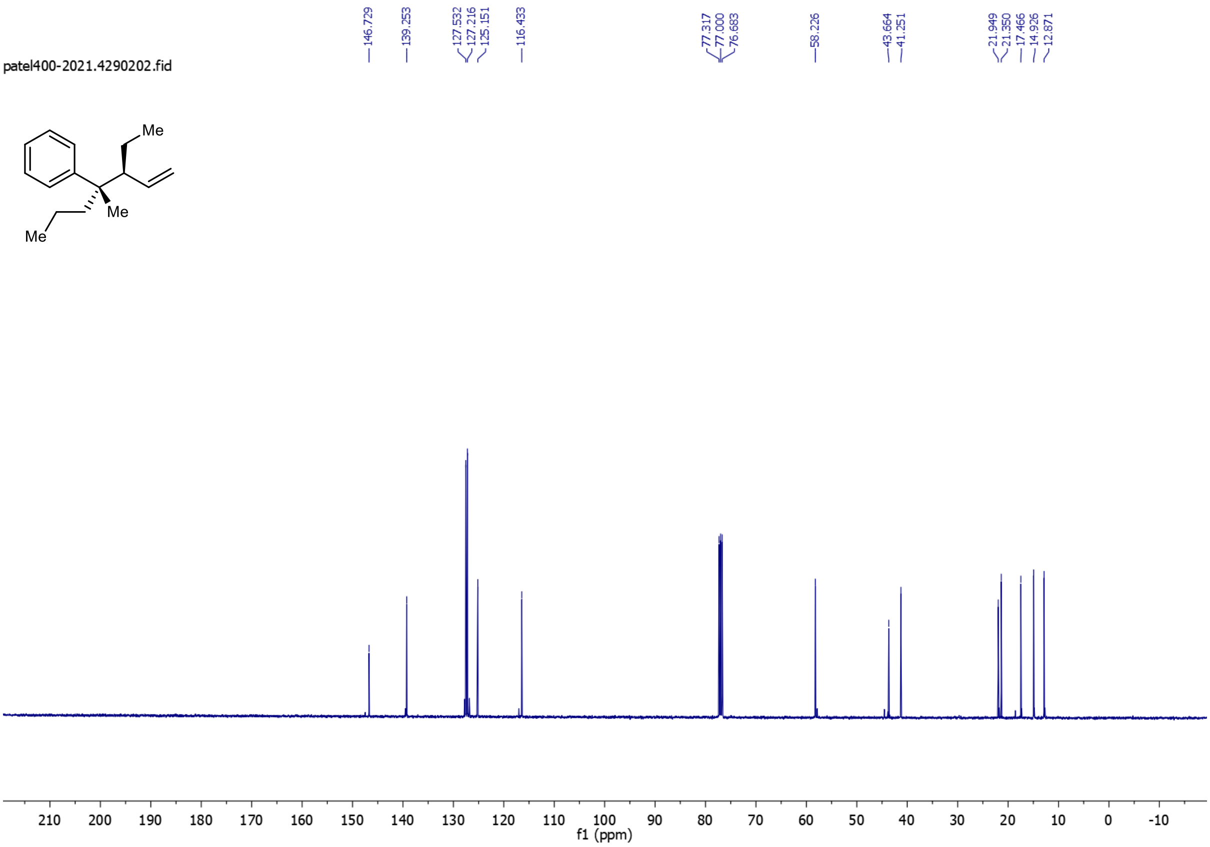
patel400-2021.4290202.fid
Me
Me
Me
f1 (ppm)

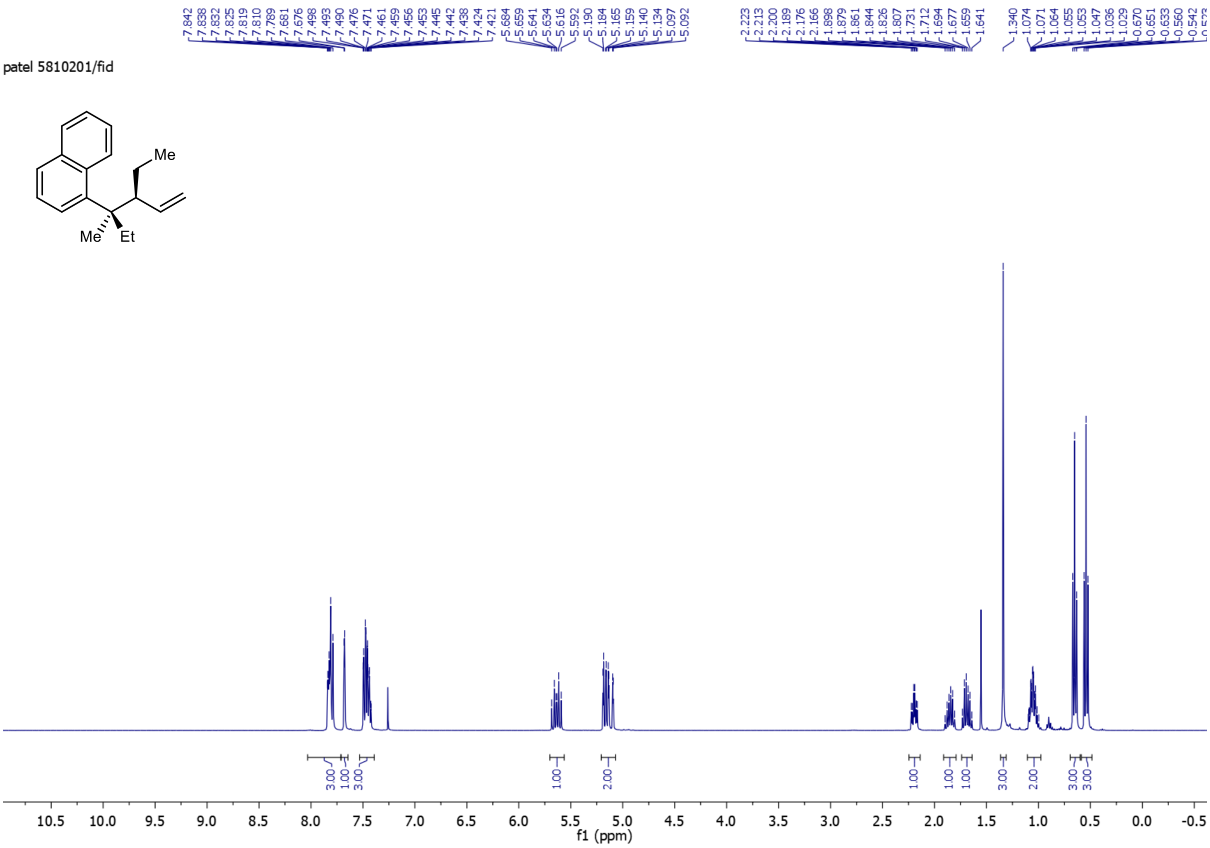
patel 5810201/fid
Me
Me
Et
f1 (ppm)

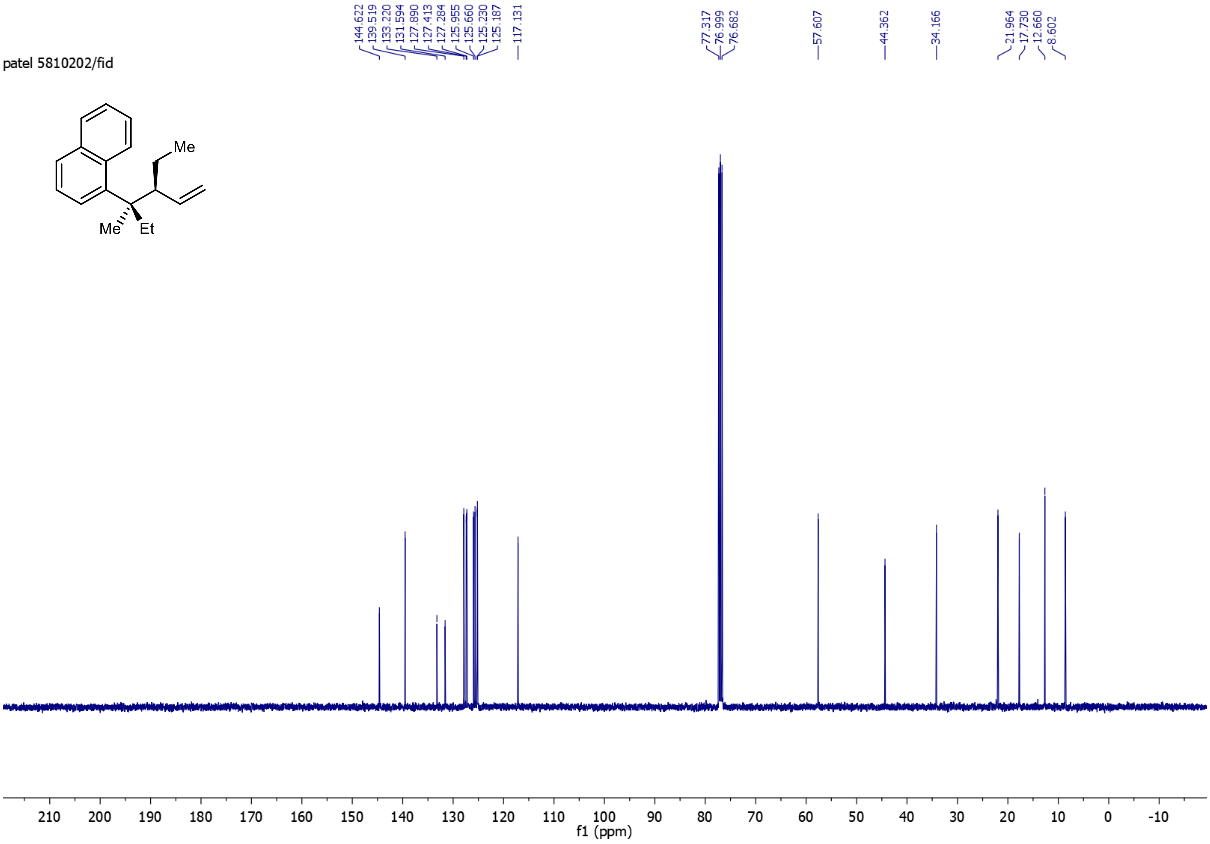
patel 5810202/fid
Me
Me
Et
f1 (ppm)

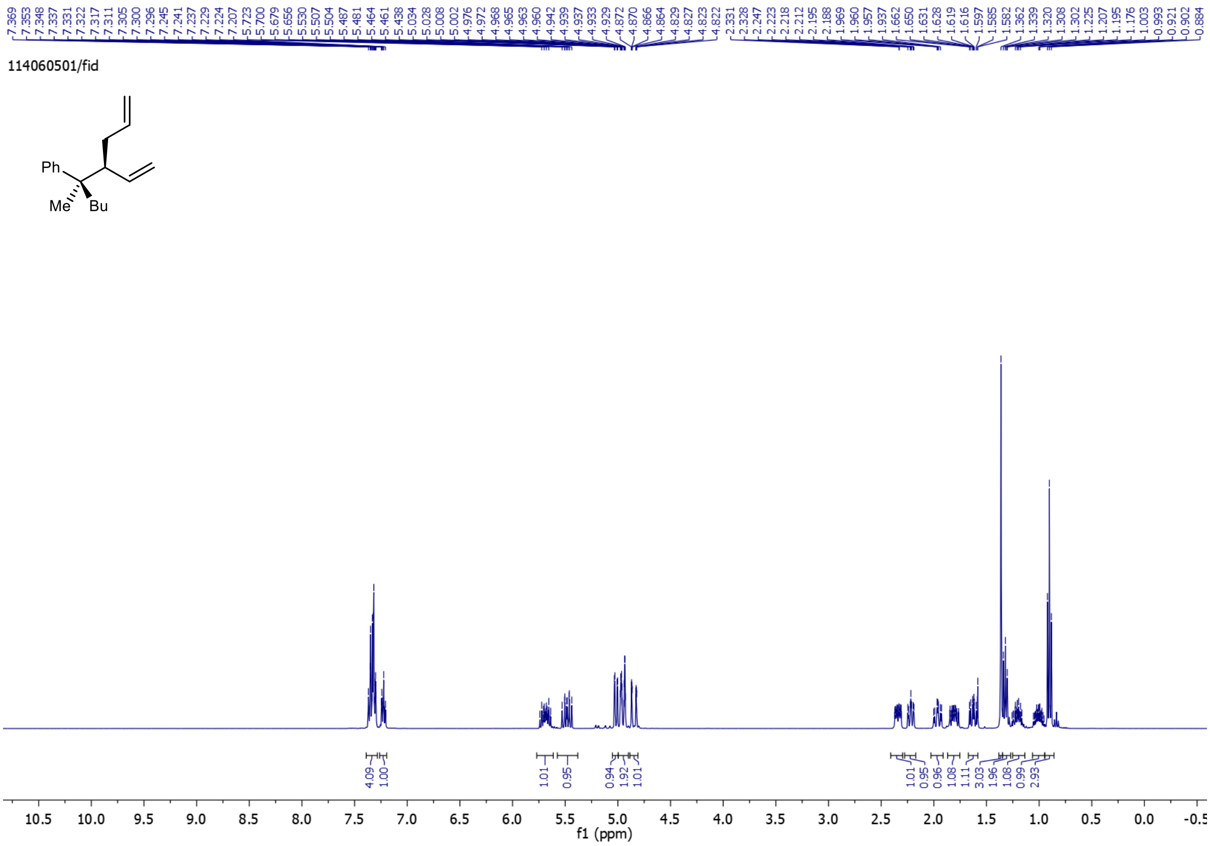
7.369 7.353 7.348 7.337 7.331 7.322 7.317 7.311 7.305 7.300 7.296 7.245 7.241 7.237 7.229 7.224 7.207 5.723 5.700 5.679 5.656 5.530 5.507 5.504 5.487 5.481 5.464 5.461 5.438 5.034 5.028 5.008 5.002 4.976 4.972 4.968 4.965 4.963 4.960 4.942 4.939 4.937 4.933 4.929 4.872 4.870 4.866 4.864 4.829 4.827 4.823 4.822 2.331 2.328 2.247 2.223 2.218 2.212 2.195 2.188 1.969 1.960 1.957 1.937 1.662 1.650 1.631 1.628 1.619 1.616 1.597 1.585 1.582 1.362 1.339 1.320 1.308 1.302 1.225 1.207 1.195 1.176 1.003 0.993 0.921 0.902 0.884
114060501/fid
Ph
Me
Bu
4.09
1.00
1.01
0.95
0.94
1.92
1.01
1.01
0.95
0.96
1.08
1.11
3.03
1.96
1.08
0.99
2.93
10.5 10.0 9.5 9.0 8.5 8.0 7.5 7.0 6.5 6.0 5.5 5.0 4.5 4.0 3.5 3.0 2.5 2.0 1.5 1.0 0.5 0.0 -0.5
f1 (ppm)

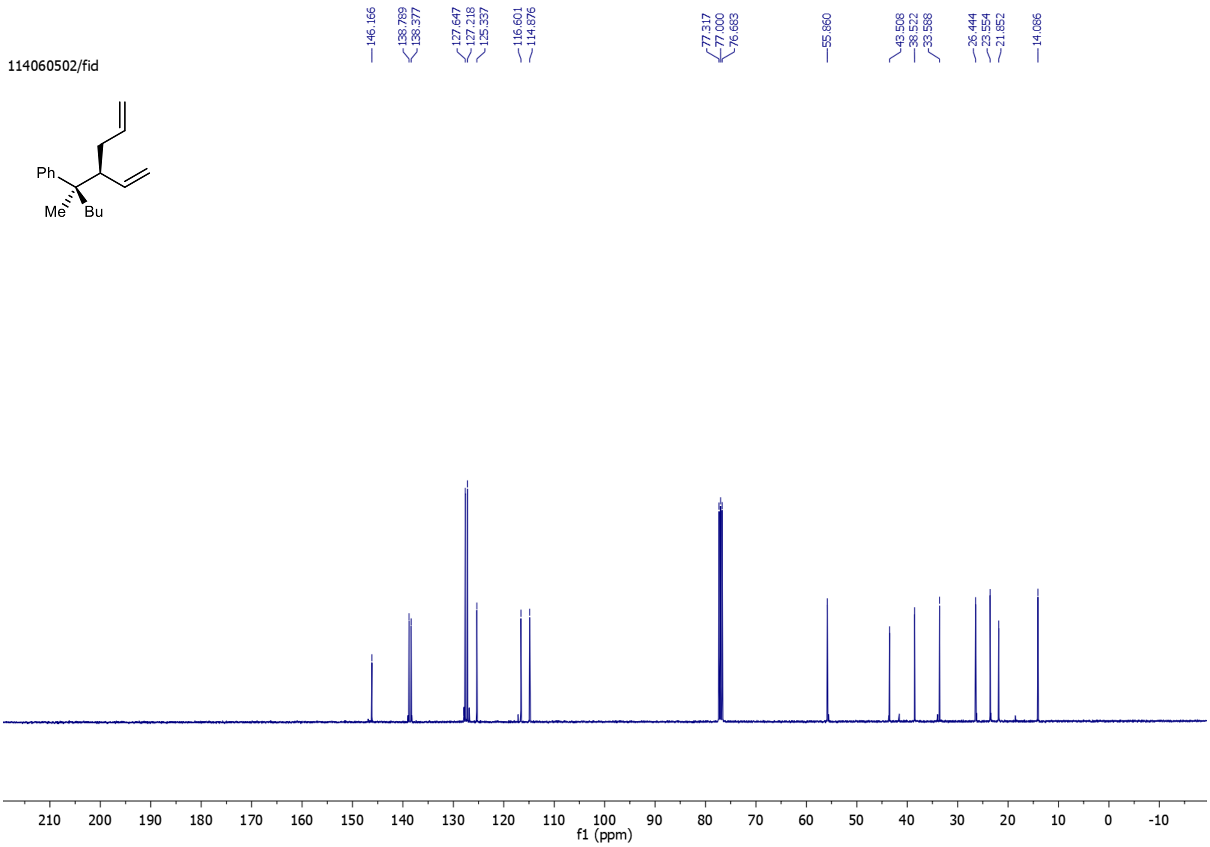
146.166 138.789 138.377 127.647 127.218 125.337 116.601 114.876 77.317 77.000 76.683 55.860 43.508 38.522 33.588 26.444 23.554 21.852 14.086
114060502/fid
Ph
Me
Bu
210 200 190 180 170 160 150 140 130 120 110 100 90 80 70 60 50 40 30 20 10 0 -10
f1 (ppm)

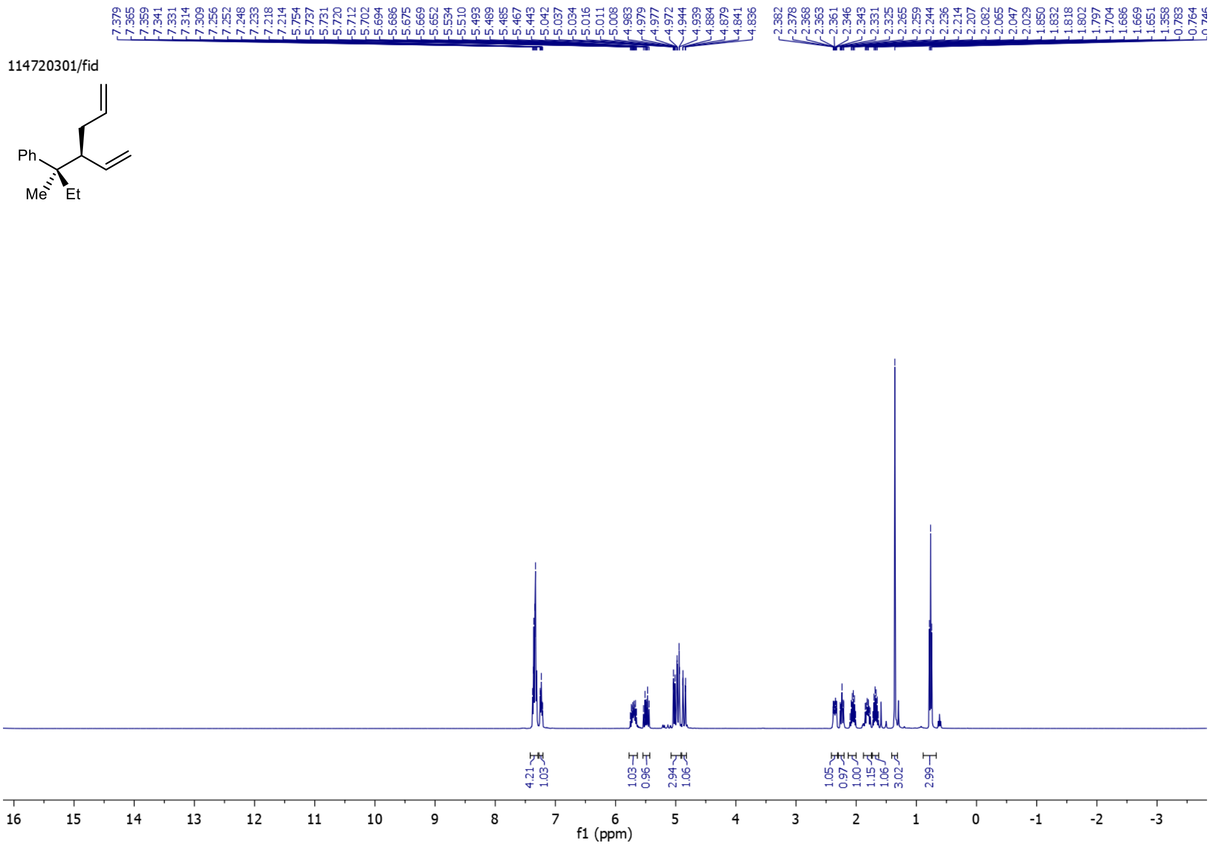
114720301/fid
Ph
Me
Et
7.379
7.365
7.359
7.341
7.331
7.314
7.309
7.256
7.252
7.248
7.233
7.218
7.214
5.754
5.737
5.731
5.720
5.712
5.702
5.694
5.686
5.675
5.669
5.652
5.534
5.510
5.493
5.489
5.485
5.467
5.443
5.042
5.037
5.034
5.016
5.011
5.008
4.983
4.979
4.977
4.972
4.944
4.939
4.884
4.879
4.841
4.836
2.382
2.378
2.368
2.363
2.361
2.346
2.343
2.331
2.325
2.265
2.259
2.244
2.236
2.214
2.207
2.082
2.065
2.047
2.029
1.850
1.832
1.818
1.802
1.797
1.704
1.686
1.669
1.651
1.358
0.783
0.764
4.21
1.03
1.03
0.96
2.94
1.06
1.05
0.97
1.00
1.15
1.06
3.02
2.99
f1 (ppm)

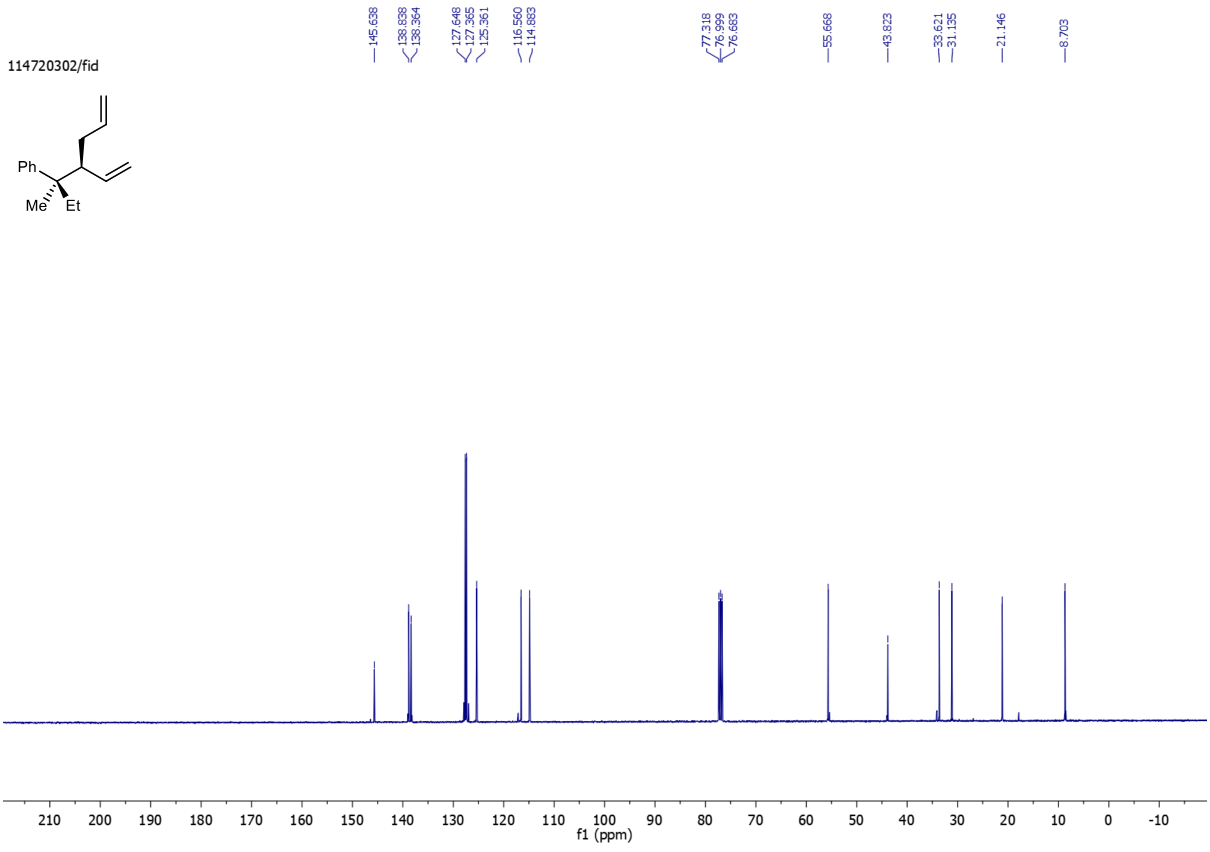
114720302/fid
Ph
Me
Et
145.638
138.838
138.364
127.648
127.365
125.361
116.560
114.883
77.318
76.999
76.683
55.668
43.823
33.621
31.135
21.146
8.703
f1 (ppm)

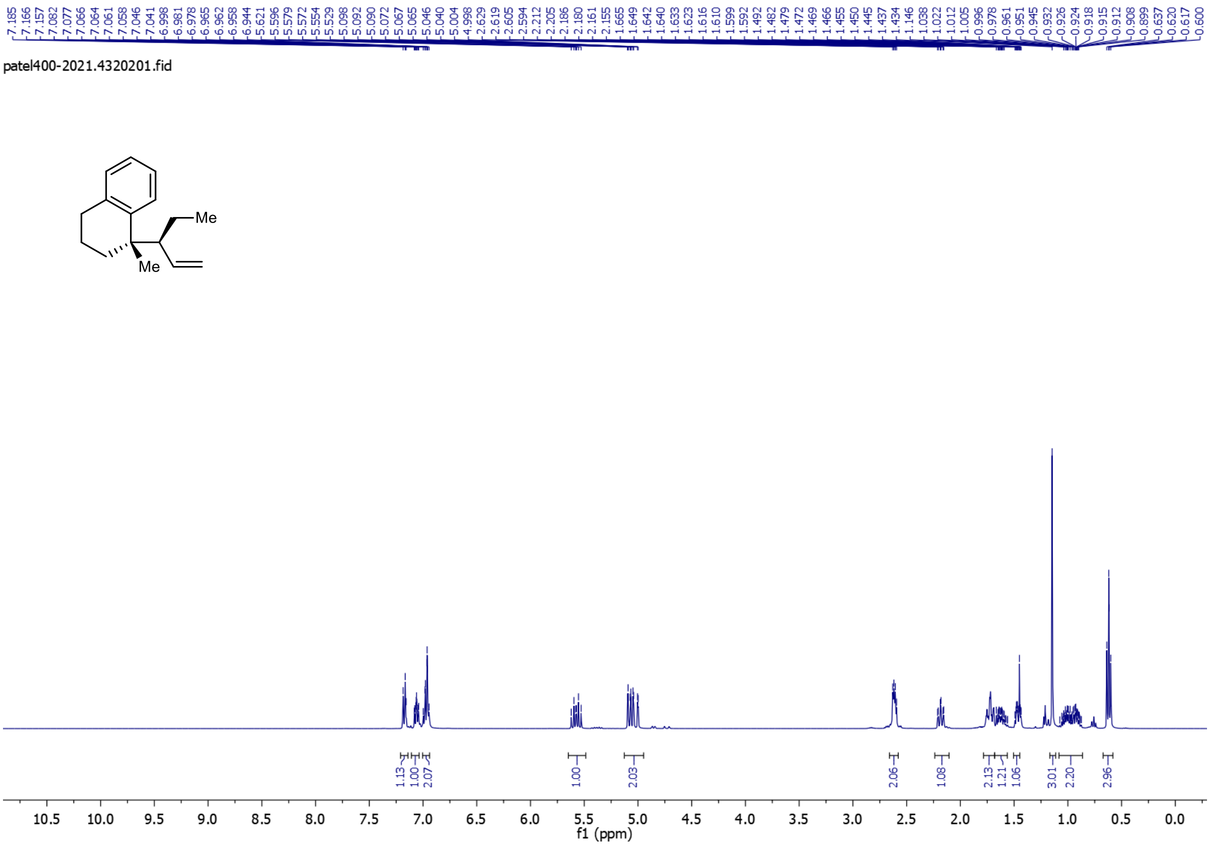
7.185 7.166 7.157 7.082 7.077 7.066 7.064 7.061 7.058 7.046 7.041 6.998 6.981 6.978 6.965 6.962 6.958 6.944 5.621 5.596 5.579 5.572 5.554 5.529 5.098 5.092 5.090 5.072 5.067 5.065 5.046 5.040 5.004 4.998 2.629 2.619 2.605 2.594 2.212 2.205 2.186 2.180 2.161 2.155 1.665 1.649 1.642 1.640 1.633 1.623 1.616 1.610 1.599 1.592 1.492 1.482 1.479 1.472 1.469 1.466 1.455 1.450 1.445 1.437 1.434 1.146 1.038 1.022 1.012 1.005 0.996 0.978 0.961 0.951 0.945 0.932 0.926 0.924 0.918 0.915 0.912 0.908 0.899 0.637 0.620 0.617 0.600
patel400-2021.4320201.fid
Me
Me
1.13
1.00
2.07
1.00
2.03
2.06
1.08
2.13
1.21
1.06
3.01
2.20
2.96
10.5 10.0 9.5 9.0 8.5 8.0 7.5 7.0 6.5 6.0 5.5 5.0 4.5 4.0 3.5 3.0 2.5 2.0 1.5 1.0 0.5 0.0
f1 (ppm)

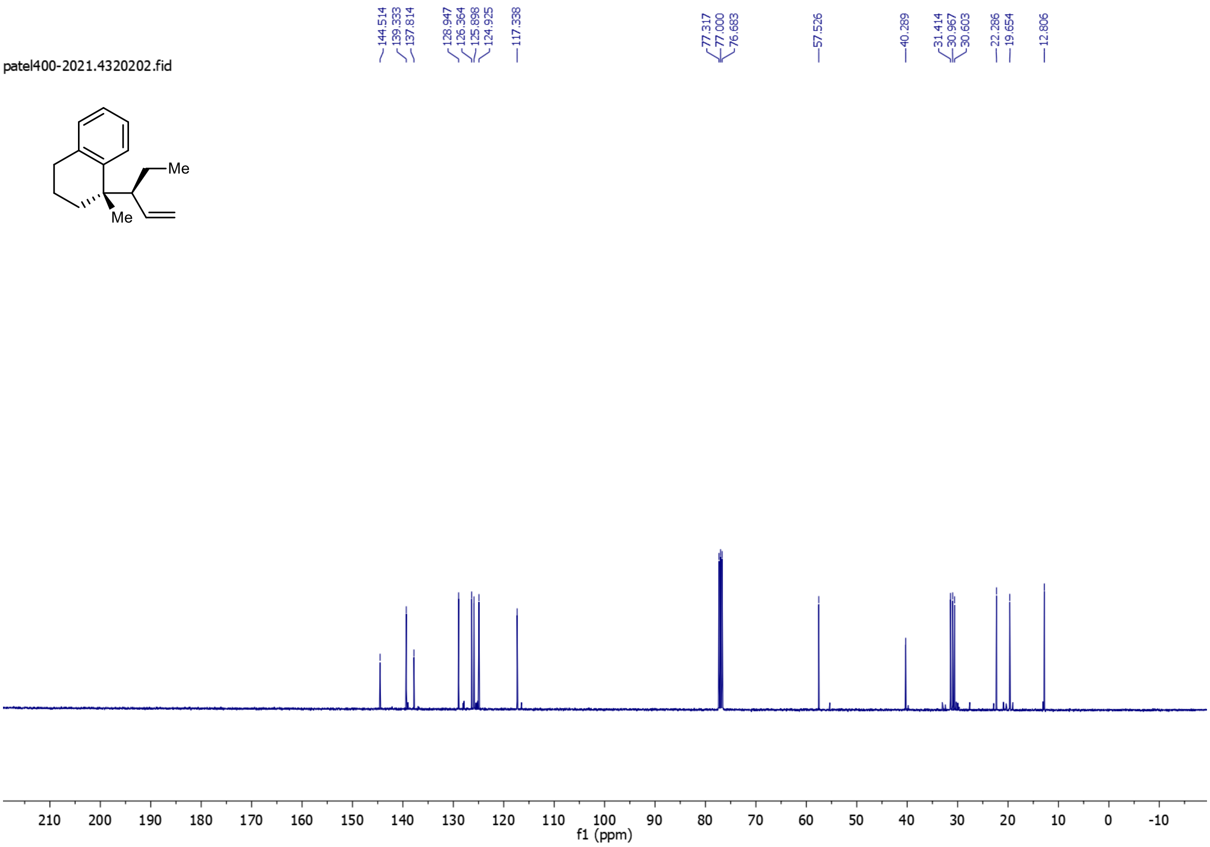
144.514 139.333 137.814 128.947 126.364 125.898 124.925 117.338 77.317 77.000 76.683 57.526 40.289 31.414 30.967 30.603 22.286 19.654 12.806
patel400-2021.4320202.fid
Me
Me
210 200 190 180 170 160 150 140 130 120 110 100 90 80 70 60 50 40 30 20 10 0 -10
f1 (ppm)

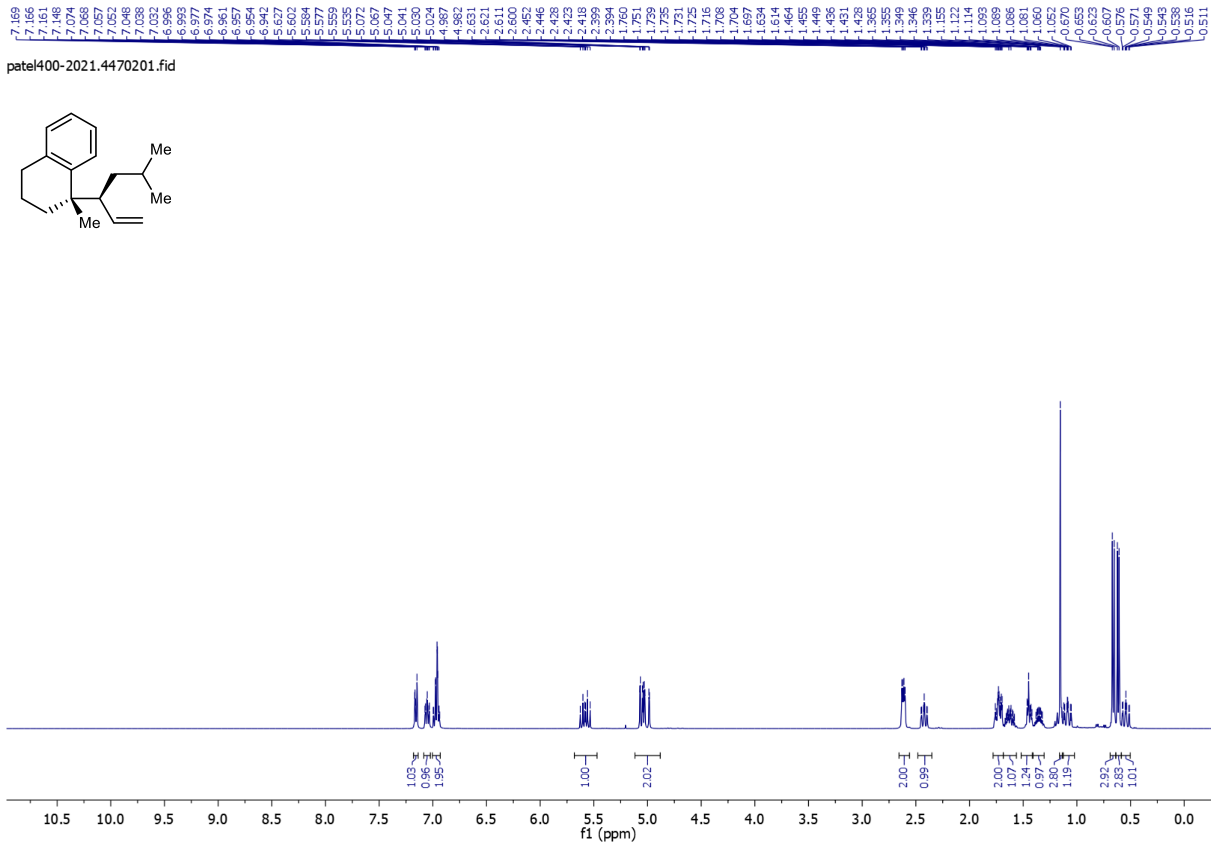
patel400-2021.4470201.fid
Me
Me
Me
f1 (ppm)

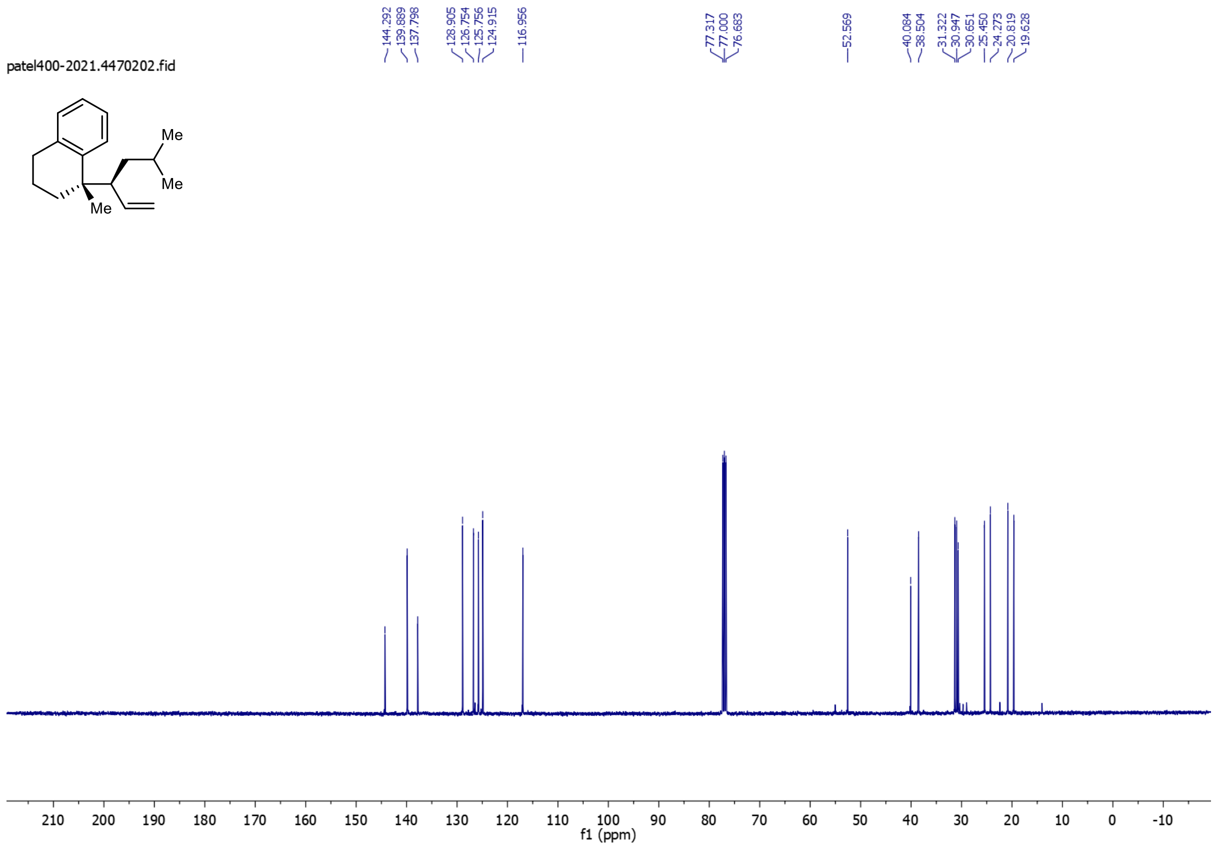
patel400-2021.4470202.fid
144.292
139.889
137.798
128.905
126.754
125.756
124.915
116.956
77.317
77.000
76.683
52.569
40.084
38.504
31.322
30.947
30.651
25.450
24.273
20.819
19.628
Me
Me
Me
f1 (ppm)

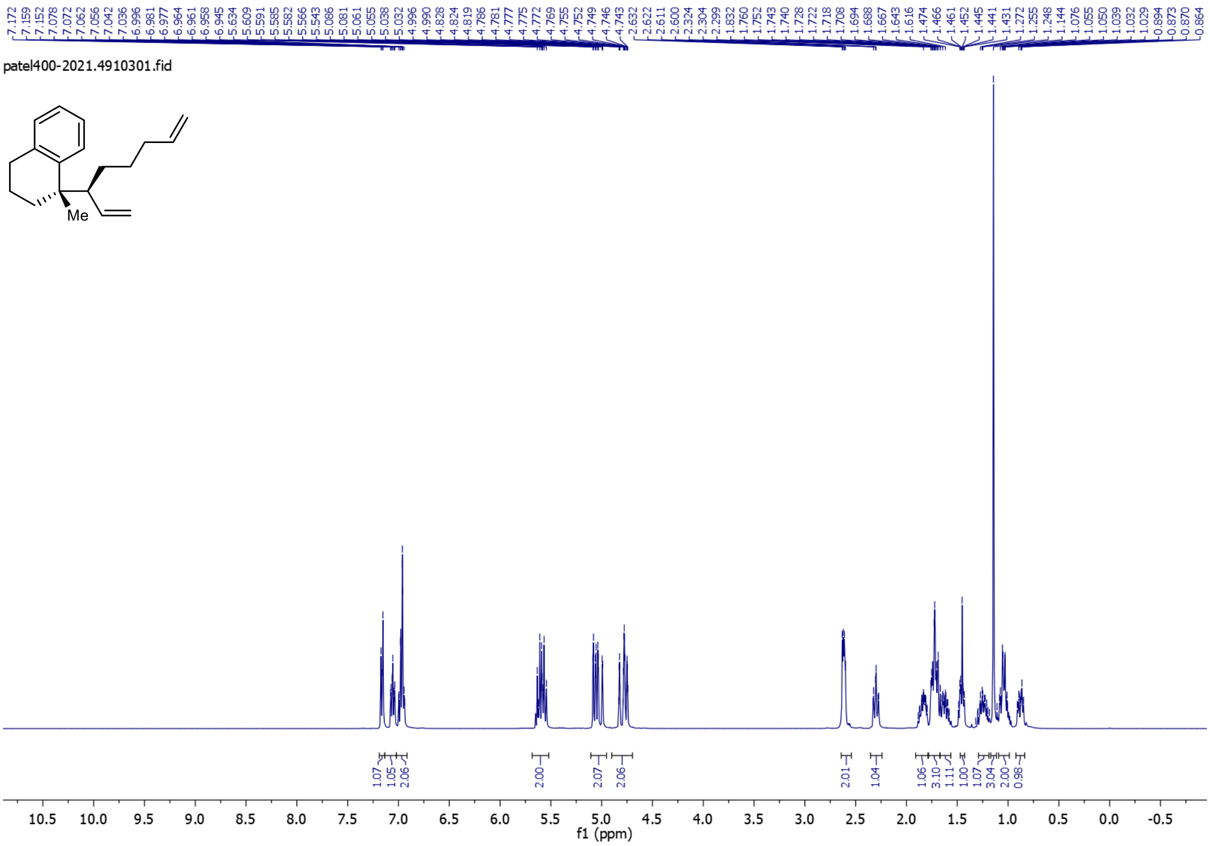
7.172 7.159 7.152 7.078 7.072 7.062 7.056 7.042 7.036 6.996 6.981 6.977 6.964 6.961 6.958 6.945 5.634 5.609 5.591 5.585 5.582 5.566 5.543 5.086 5.081 5.061 5.055 5.038 5.032 4.996 4.990 4.828 4.824 4.819 4.786 4.781 4.777 4.775 4.772 4.769 4.755 4.752 4.749 4.746 4.743 2.632 2.622 2.611 2.600 2.324 2.304 2.299 1.832 1.760 1.752 1.743 1.740 1.728 1.722 1.718 1.708 1.694 1.688 1.667 1.643 1.616 1.474 1.466 1.461 1.452 1.445 1.441 1.431 1.272 1.255 1.248 1.144 1.076 1.055 1.050 1.039 1.032 1.029 0.894 0.873 0.870 0.864
patel400-2021.4910301.fid
Me
1.07 1.05 2.06 2.00 2.07 2.06 2.01 1.04 1.06 3.10 1.11 1.00 1.07 3.04 2.00 0.98
10.5 10.0 9.5 9.0 8.5 8.0 7.5 7.0 6.5 6.0 5.5 5.0 4.5 4.0 3.5 3.0 2.5 2.0 1.5 1.0 0.5 0.0 -0.5
f1 (ppm)

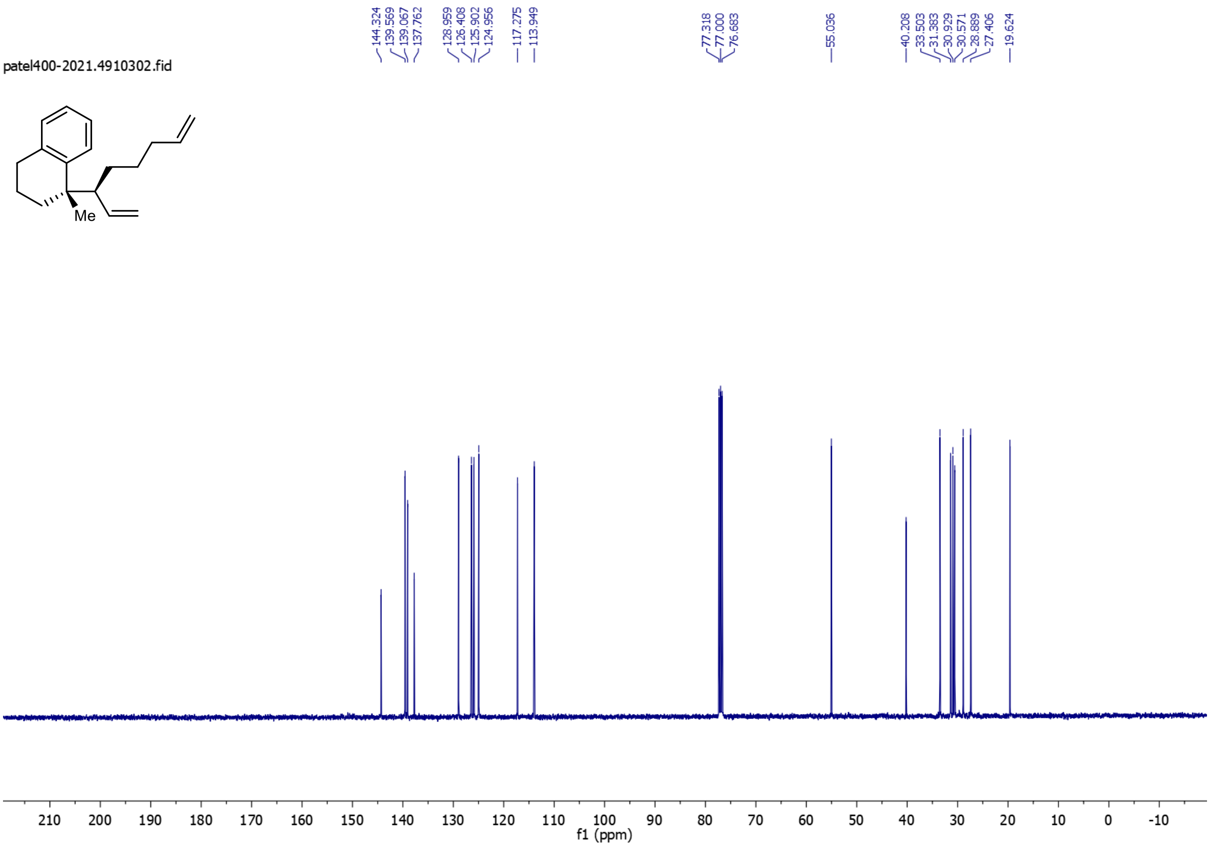
144.324 139.569 139.067 137.762 128.959 126.408 125.902 124.956 117.275 113.949 77.318 77.000 76.683 55.036 40.208 33.503 31.383 30.929 30.571 28.889 27.406 19.624
patel400-2021.4910302.fid
Me
210 200 190 180 170 160 150 140 130 120 110 100 90 80 70 60 50 40 30 20 10 0 -10
f1 (ppm)

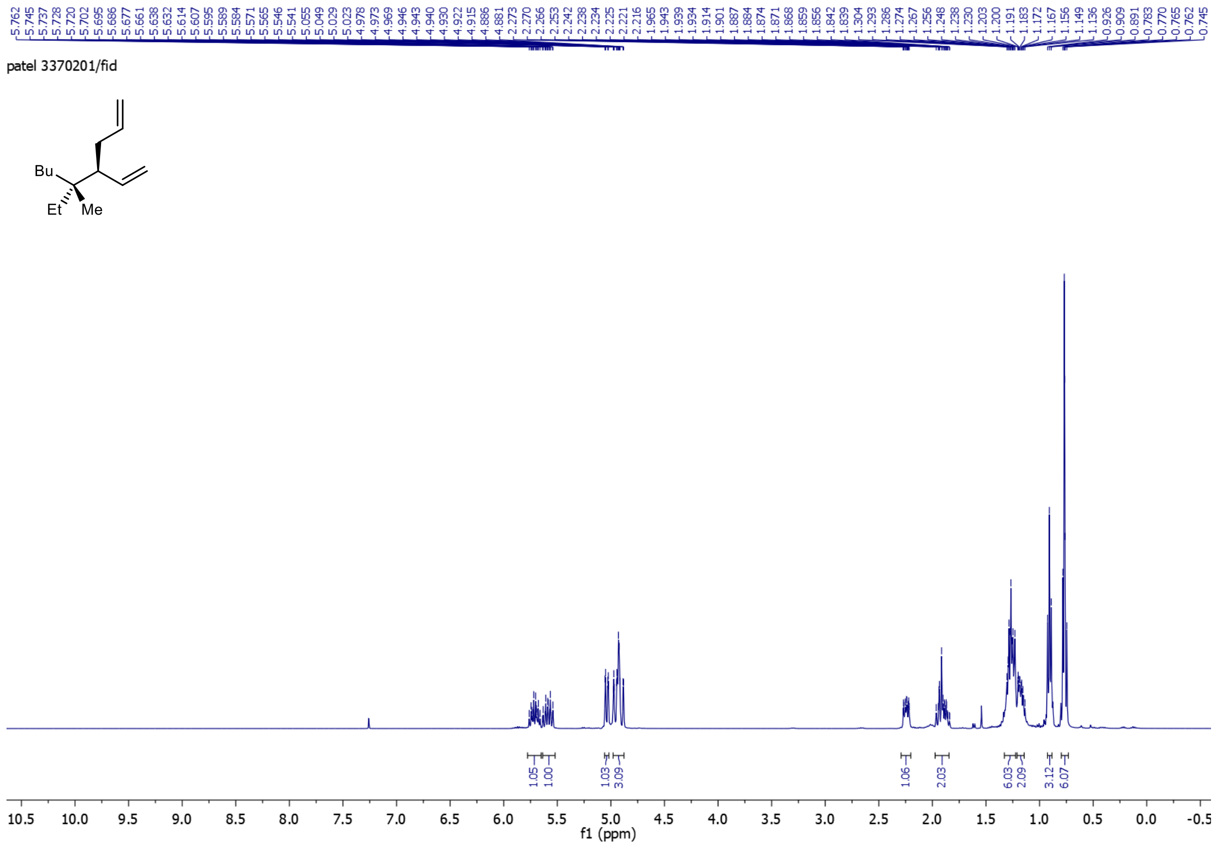

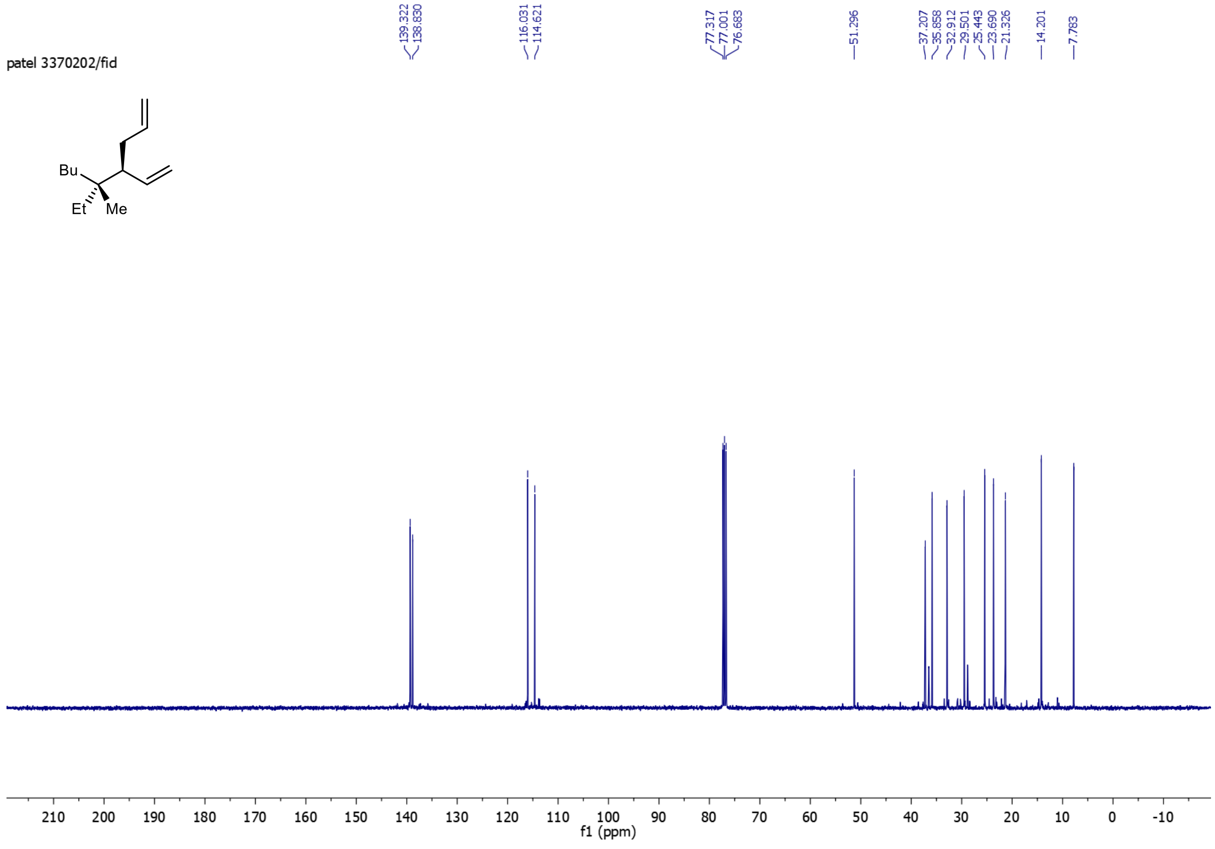

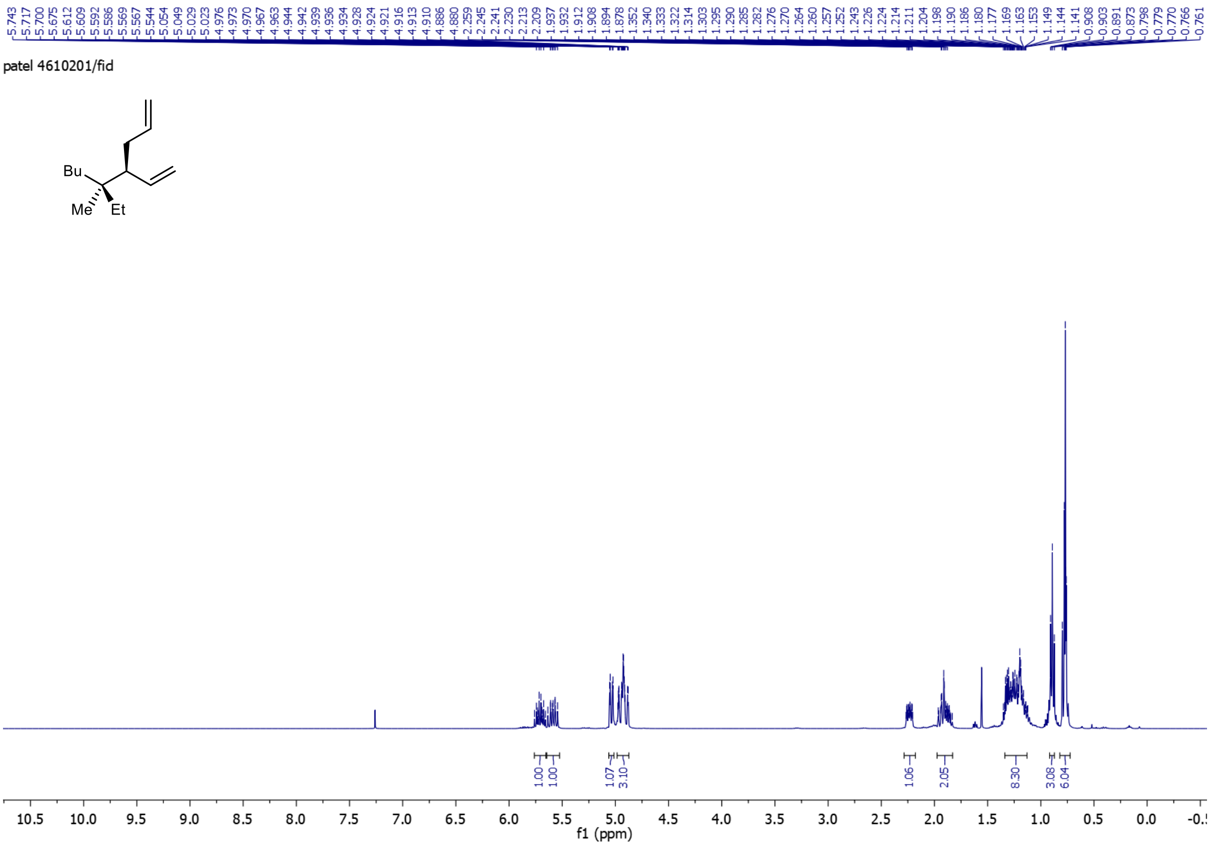
5.743
5.717
5.700
5.675
5.612
5.609
5.592
5.586
5.569
5.567
5.544
5.054
5.049
5.029
5.023
4.976
4.973
4.970
4.967
4.963
4.944
4.942
4.939
4.936
4.934
4.928
4.924
4.921
4.916
4.913
4.910
4.886
4.880
2.259
2.245
2.241
2.230
2.213
2.209
1.937
1.932
1.912
1.908
1.894
1.878
1.352
1.340
1.333
1.322
1.314
1.303
1.295
1.290
1.285
1.282
1.276
1.270
1.264
1.260
1.257
1.252
1.243
1.226
1.224
1.214
1.211
1.204
1.198
1.190
1.186
1.180
1.177
1.169
1.163
1.153
1.149
1.144
1.141
0.908
0.903
0.891
0.873
0.798
0.779
0.770
0.766
0.761
patel 4610201/fid
Bu
Me
Et
1.00
1.00
1.07
3.10
1.06
2.05
8.30
3.08
6.04
f1 (ppm)

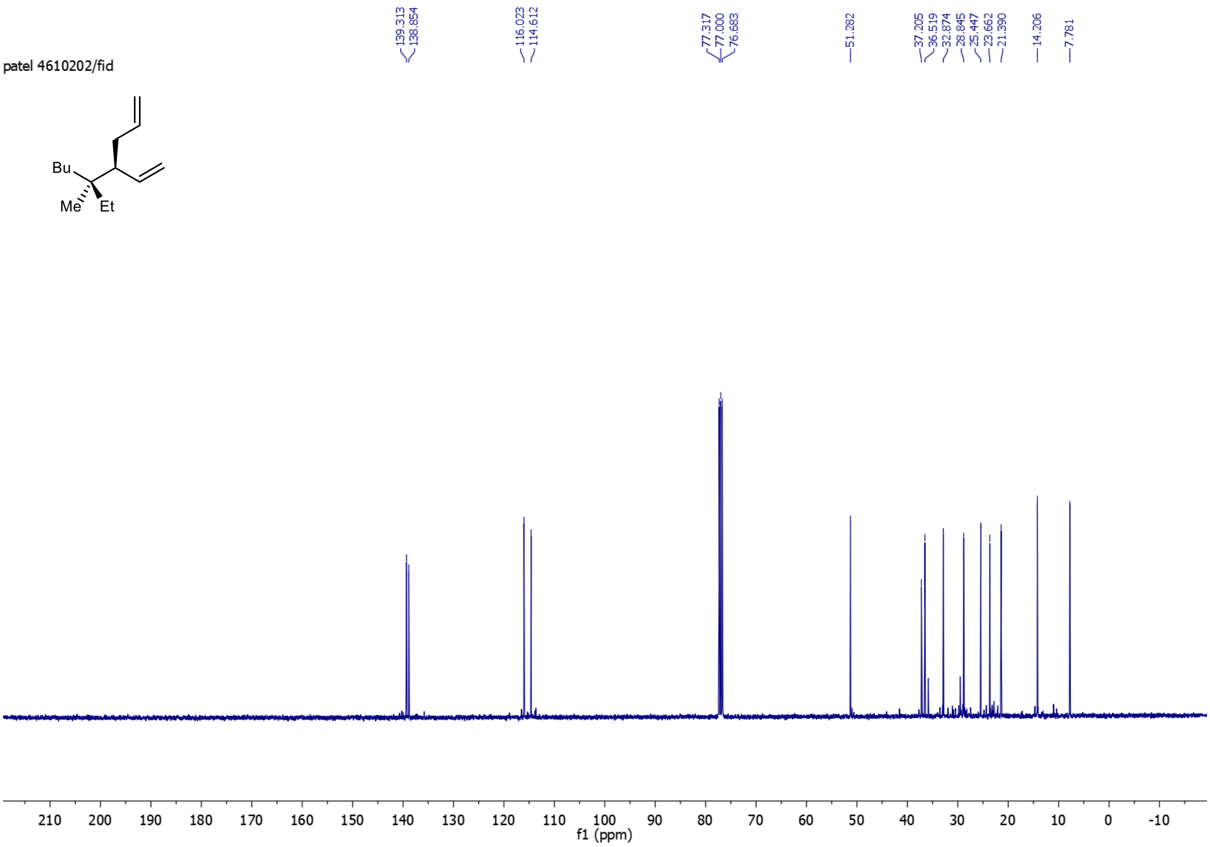
139.313
138.854
116.023
114.612
77.317
77.000
76.683
51.282
37.205
36.519
32.874
28.845
25.447
23.662
21.390
14.206
7.781
patel 4610202/fid
Bu
Me
Et
f1 (ppm)

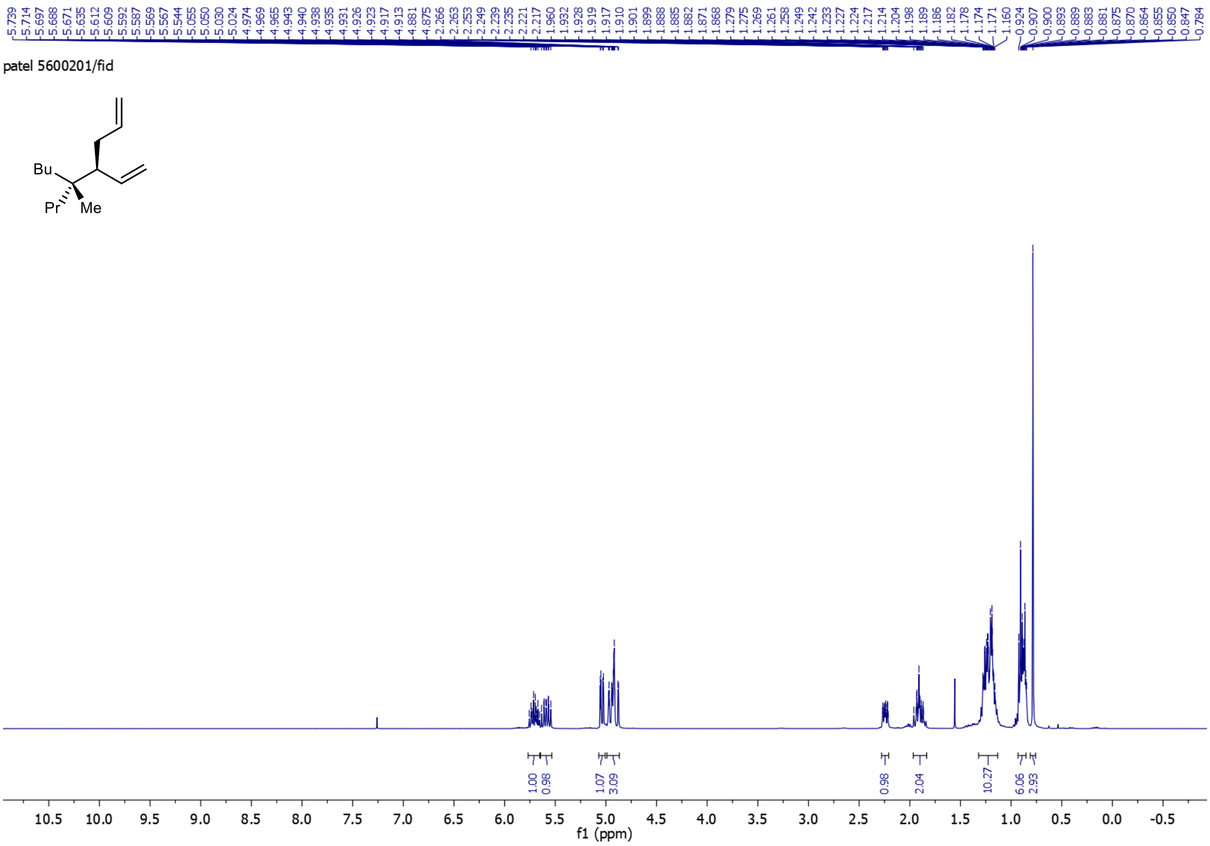
5.739 5.714 5.697 5.688 5.671 5.635 5.612 5.609 5.592 5.587 5.569 5.567 5.544 5.055 5.050 5.030 5.024 4.974 4.969 4.965 4.943 4.940 4.938 4.935 4.931 4.926 4.923 4.917 4.913 4.881 4.875 2.266 2.263 2.253 2.249 2.239 2.235 2.221 2.217 1.960 1.932 1.928 1.919 1.917 1.910 1.901 1.899 1.888 1.885 1.882 1.871 1.868 1.279 1.275 1.269 1.261 1.258 1.249 1.242 1.233 1.227 1.224 1.217 1.214 1.204 1.198 1.189 1.186 1.182 1.178 1.174 1.171 1.160 0.924 0.907 0.900 0.893 0.889 0.883 0.881 0.875 0.870 0.864 0.855 0.850 0.847 0.784
patel 5600201/fid
Bu
Pr
Me
1.00 0.98 1.07 3.09 0.98 2.04 10.27 6.06 2.93
10.5 10.0 9.5 9.0 8.5 8.0 7.5 7.0 6.5 6.0 5.5 5.0 4.5 4.0 3.5 3.0 2.5 2.0 1.5 1.0 0.5 0.0 -0.5
f1 (ppm)

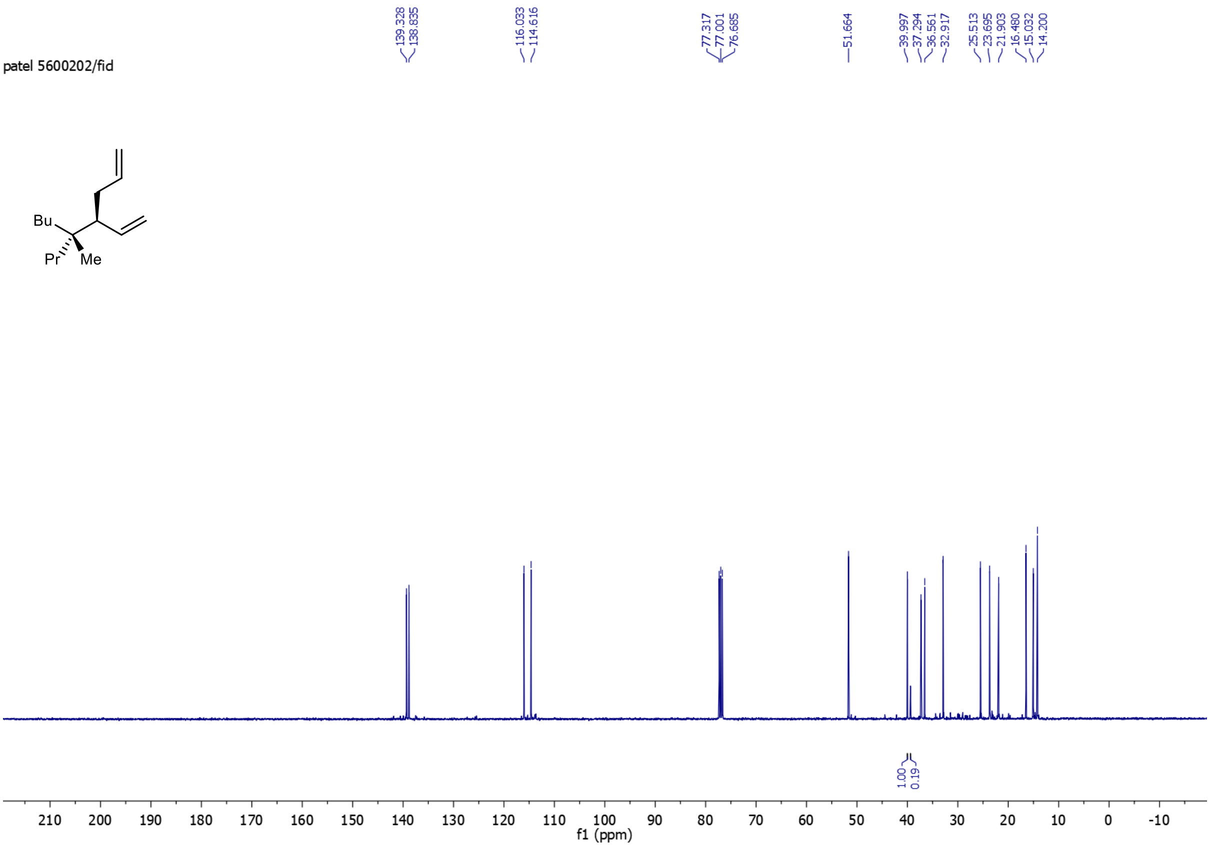
139.328 138.835 116.033 114.616 77.317 77.001 76.685 51.664 39.997 37.294 36.561 32.917 25.513 23.695 21.903 16.480 15.032 14.200
patel 5600202/fid
Bu
Pr
Me
1.00 0.19
210 200 190 180 170 160 150 140 130 120 110 100 90 80 70 60 50 40 30 20 10 0 -10
f1 (ppm)

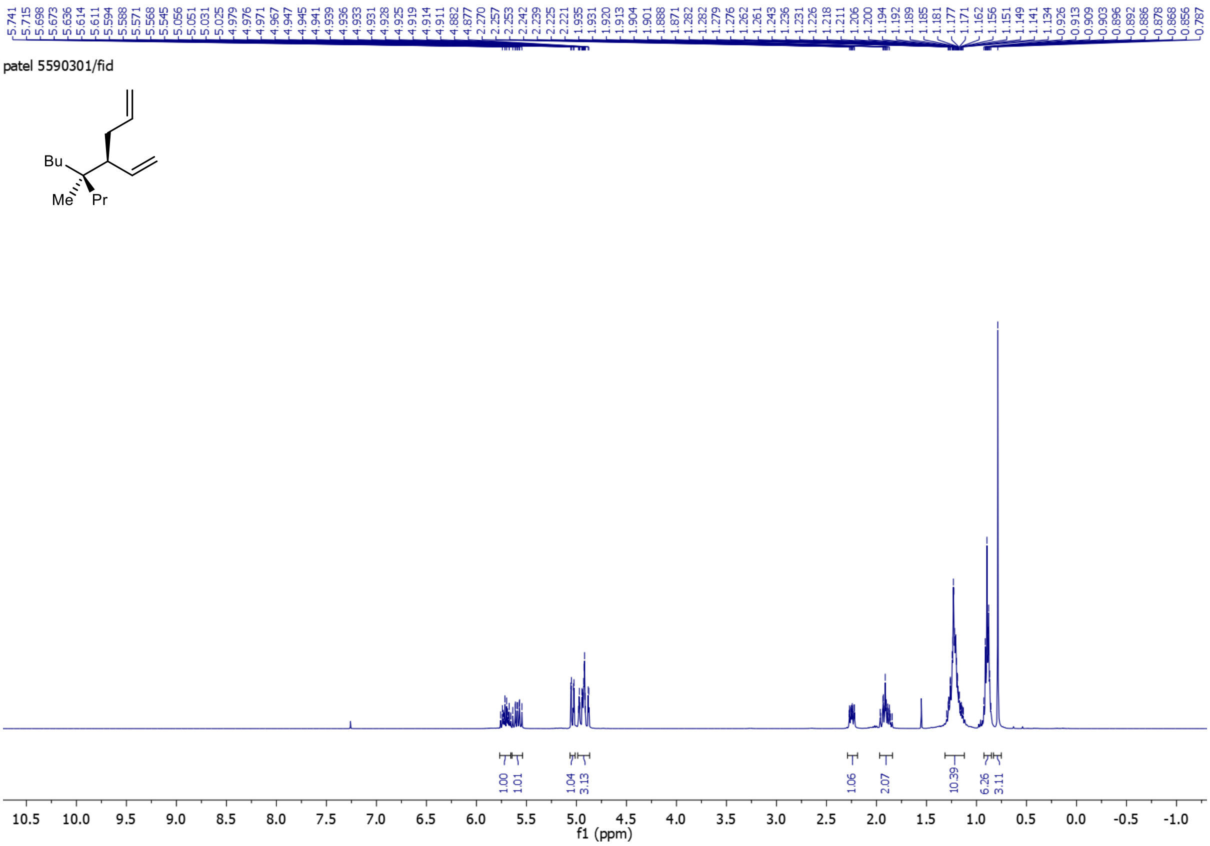
5.741 5.715 5.698 5.673 5.636 5.614 5.611 5.594 5.588 5.571 5.568 5.545 5.056 5.051 5.031 5.025 4.979 4.976 4.971 4.967 4.947 4.945 4.941 4.939 4.936 4.933 4.931 4.928 4.925 4.919 4.914 4.911 4.882 4.877 2.270 2.257 2.253 2.242 2.239 2.225 2.221 1.935 1.931 1.920 1.913 1.904 1.901 1.888 1.871 1.282 1.282 1.279 1.276 1.262 1.261 1.243 1.236 1.231 1.226 1.218 1.211 1.206 1.200 1.194 1.192 1.189 1.185 1.181 1.177 1.171 1.162 1.156 1.151 1.149 1.141 1.134 0.926 0.913 0.909 0.903 0.896 0.892 0.886 0.878 0.868 0.856 0.787
patel 5590301/fid
Bu
Me
Pr
1.00 1.01 1.04 3.13 1.06 2.07 10.39 6.26 3.11
f1 (ppm)

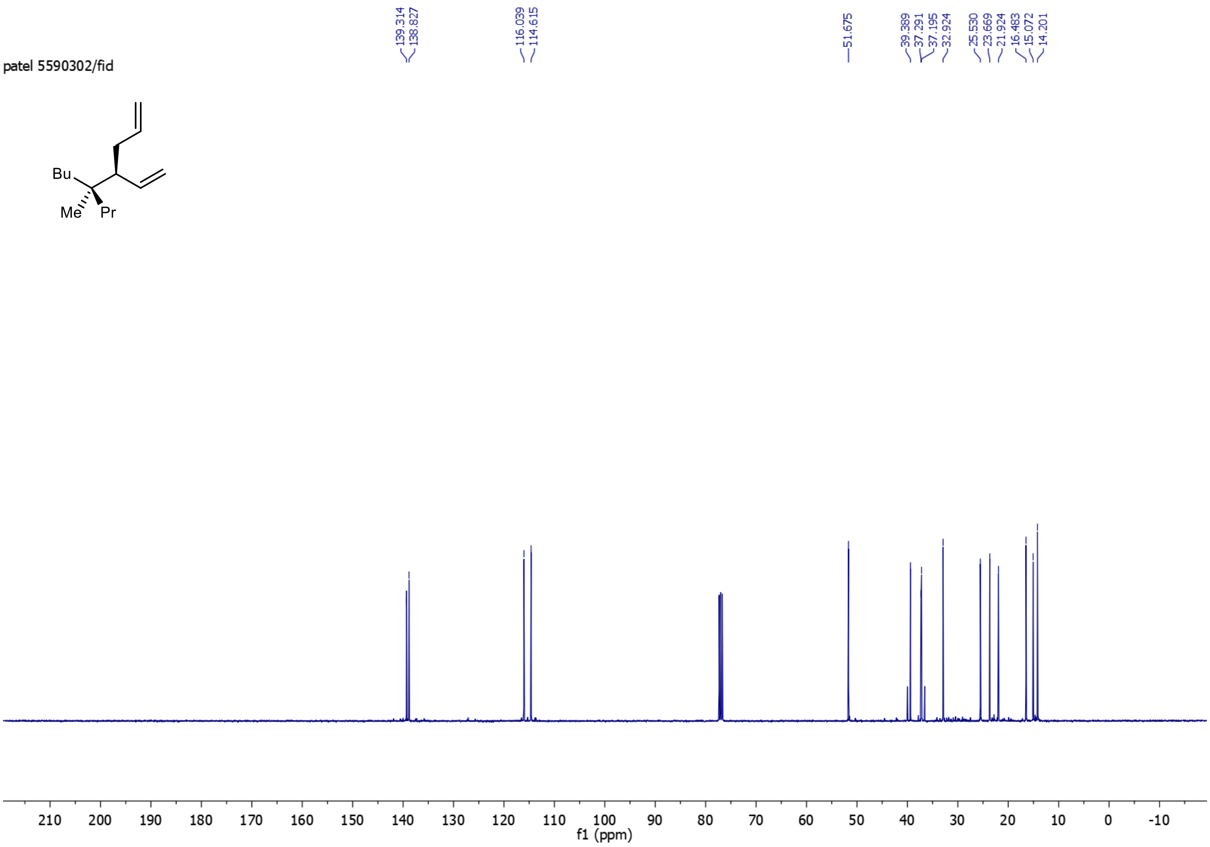
139.314 138.827 116.039 114.615 51.675 39.389 37.291 37.195 32.924 25.530 23.669 21.924 16.483 15.072 14.201
patel 5590302/fid
Bu
Me
Pr
f1 (ppm)

patel 3700201/fid

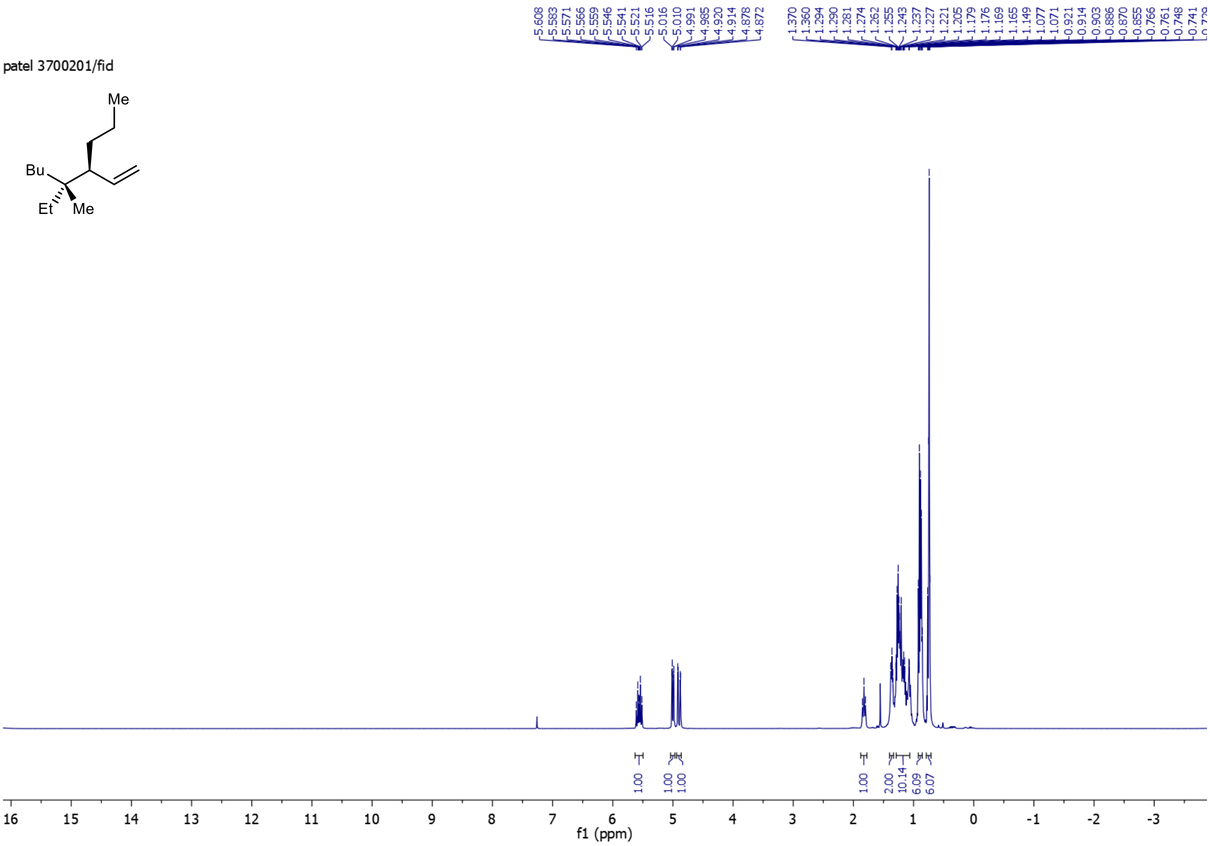

patel 3700202/fid

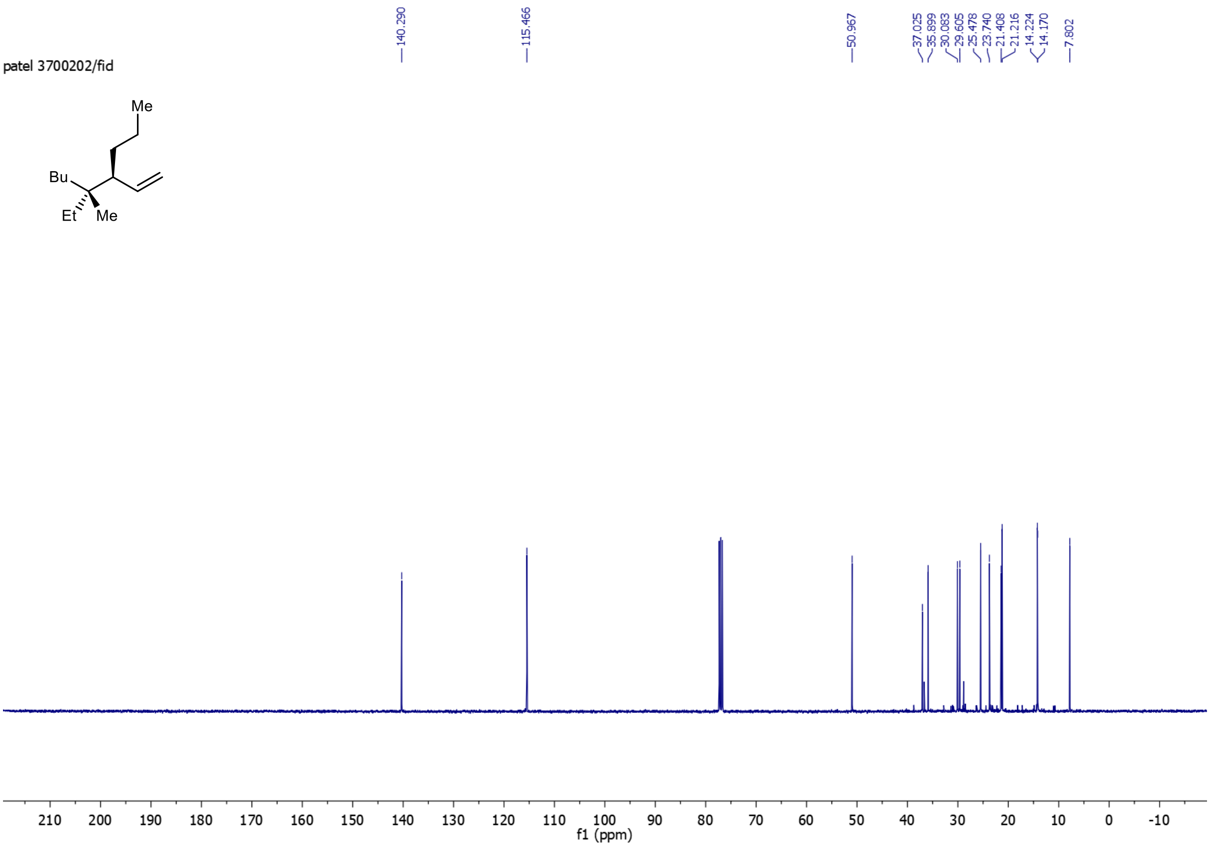

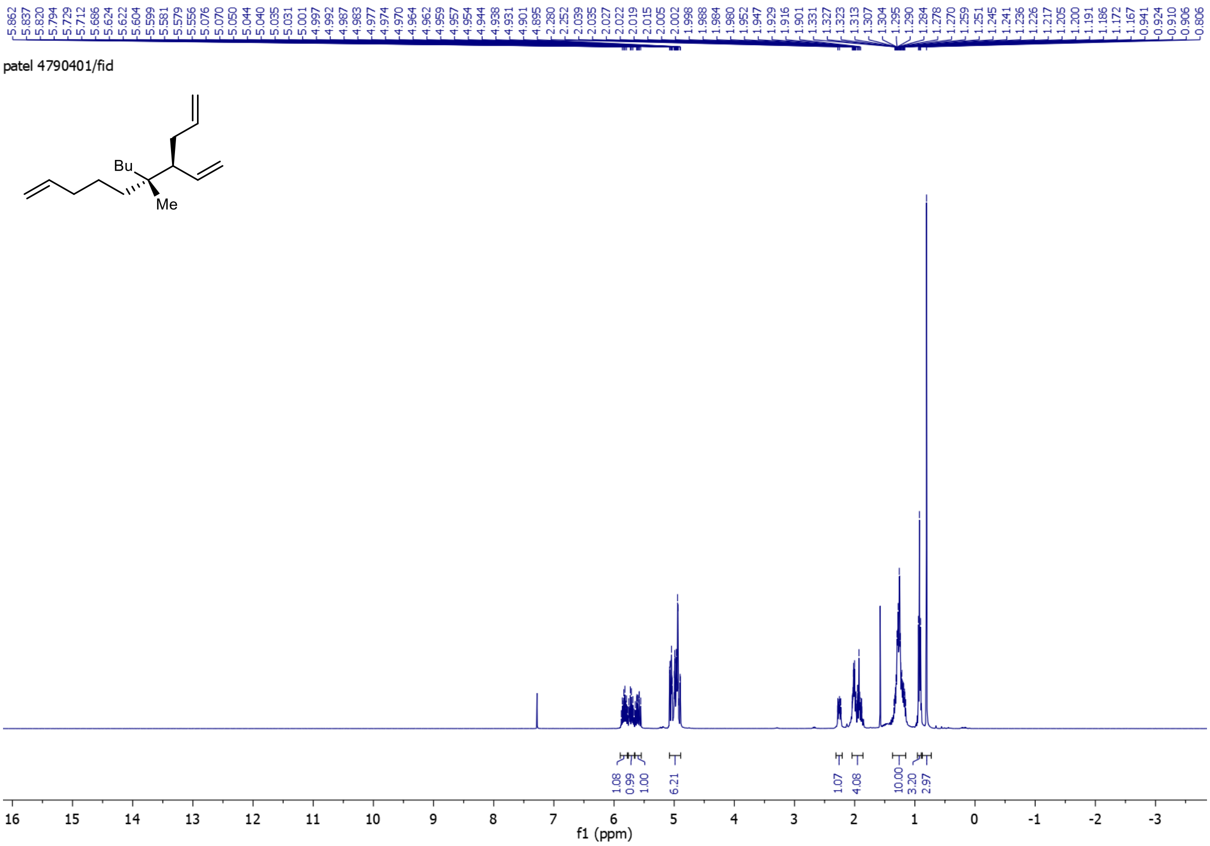
patel 4790401/fid
Bu
Me
f1 (ppm)

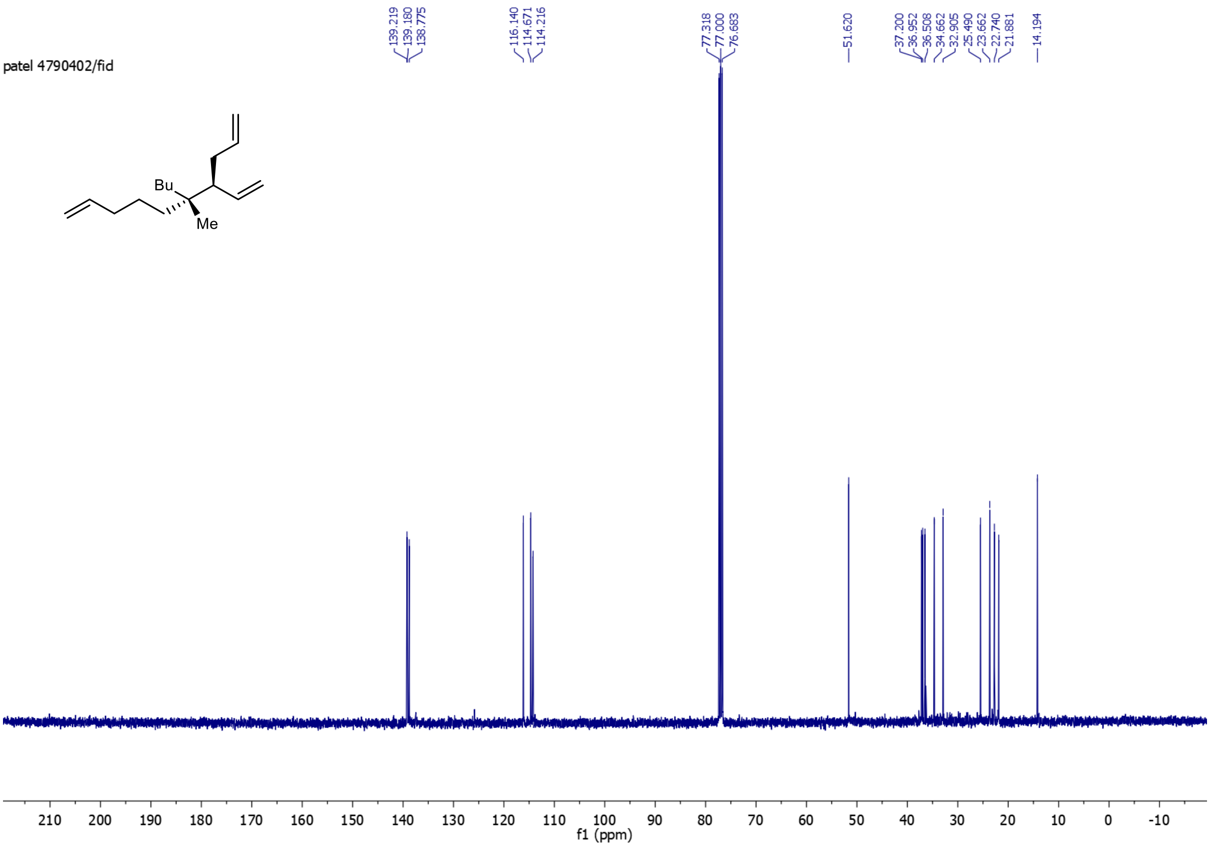
patel 4790402/fid
139.219
139.180
138.775
116.140
114.671
114.216
77.318
77.000
76.683
51.620
37.200
36.952
36.508
34.662
32.905
25.490
23.662
22.740
21.881
14.194
Bu
Me
f1 (ppm)

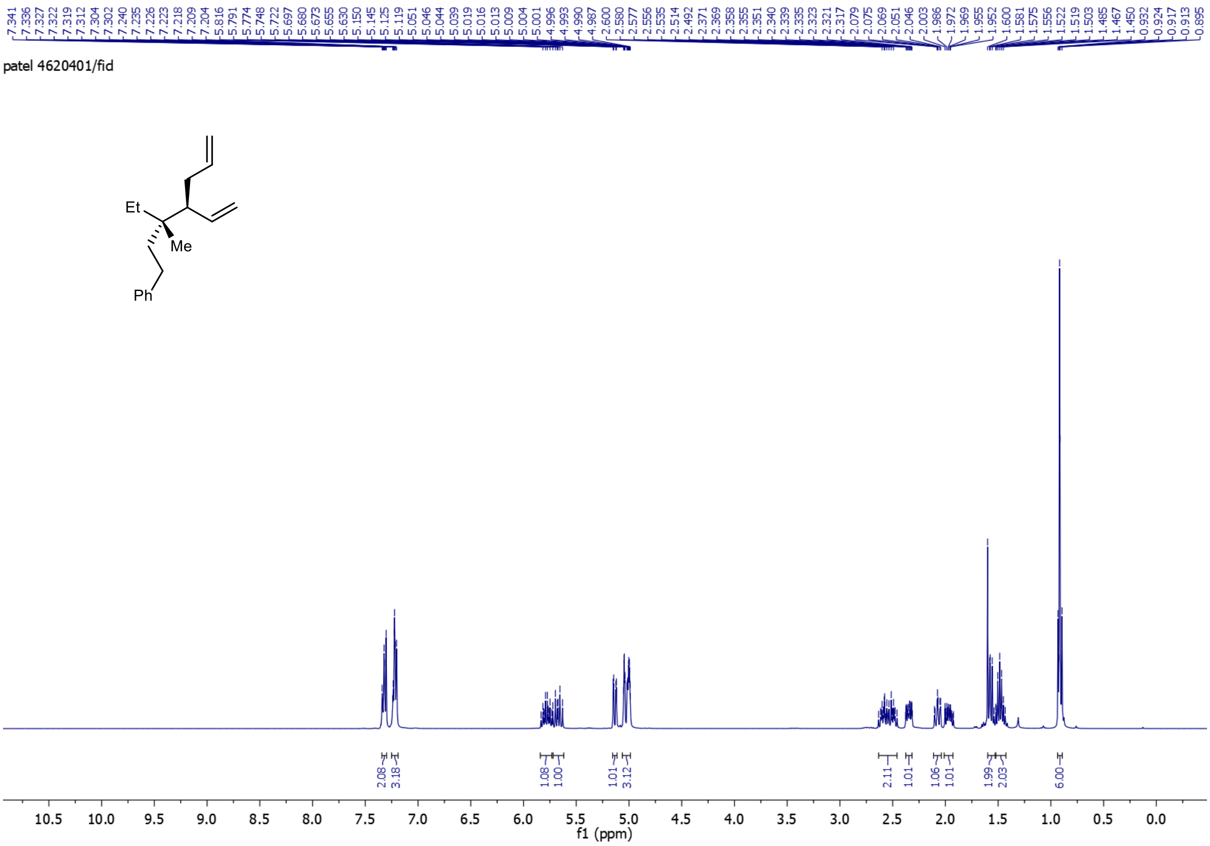
7.341 7.336 7.327 7.322 7.319 7.312 7.304 7.302 7.240 7.235 7.226 7.223 7.218 7.209 7.204 5.816 5.791 5.774 5.748 5.722 5.697 5.680 5.673 5.655 5.630 5.150 5.145 5.125 5.119 5.051 5.046 5.044 5.039 5.019 5.016 5.013 5.009 5.004 5.001 4.996 4.993 4.990 4.987 2.600 2.580 2.577 2.556 2.535 2.514 2.492 2.371 2.369 2.358 2.355 2.351 2.340 2.339 2.335 2.323 2.321 2.317 2.079 2.075 2.069 2.051 2.046 2.003 1.986 1.972 1.969 1.955 1.952 1.600 1.581 1.575 1.556 1.522 1.519 1.503 1.485 1.467 1.450 0.932 0.924 0.917 0.913 0.895
patel 4620401/fid
Et
Me
Ph
2.08
3.18
1.08
1.00
1.01
3.12
2.11
1.01
1.06
1.01
1.99
2.03
6.00
10.5 10.0 9.5 9.0 8.5 8.0 7.5 7.0 6.5 6.0 5.5 5.0 4.5 4.0 3.5 3.0 2.5 2.0 1.5 1.0 0.5 0.0
f1 (ppm)

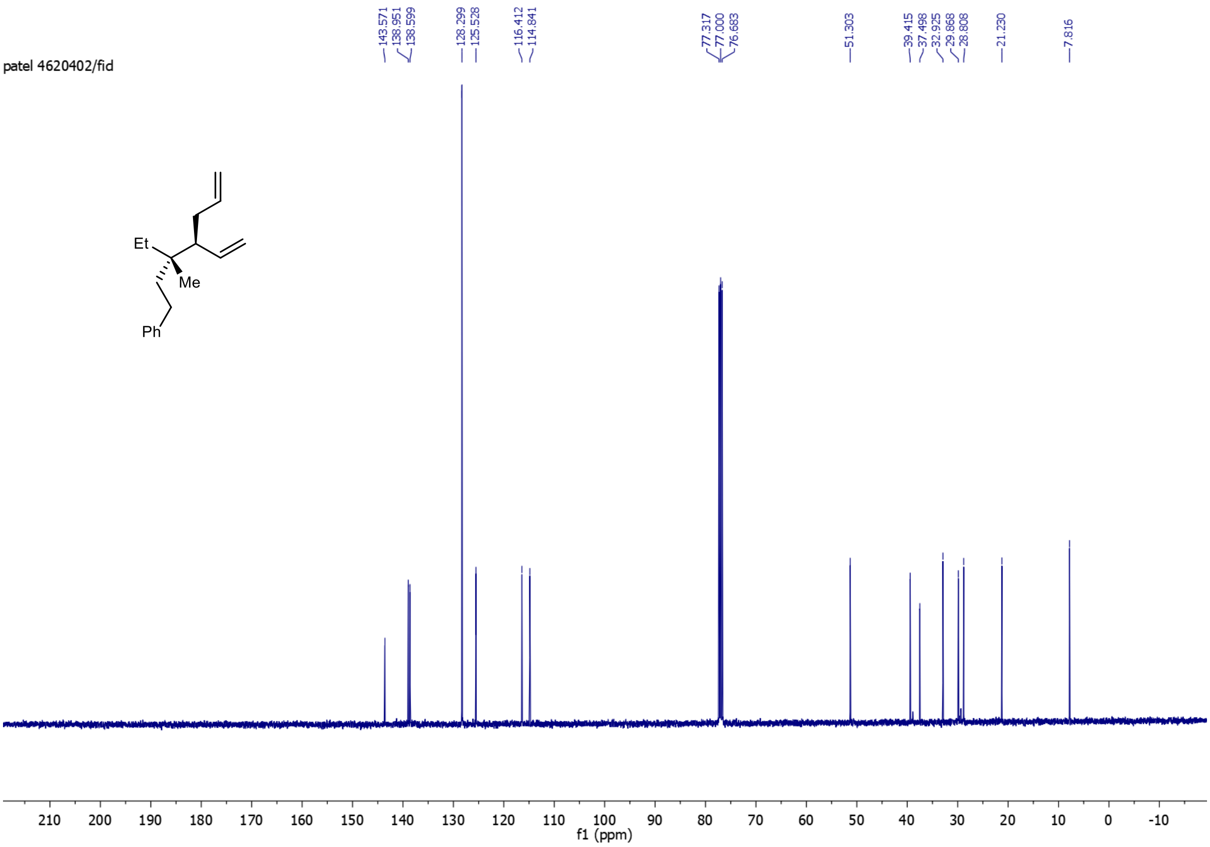
143.571 138.951 138.599 128.299 125.528 116.412 114.841 77.317 77.000 76.683 51.303 39.415 37.498 32.925 29.868 28.808 21.230 7.816
patel 4620402/fid
Et
Me
Ph
210 200 190 180 170 160 150 140 130 120 110 100 90 80 70 60 50 40 30 20 10 0 -10
f1 (ppm)

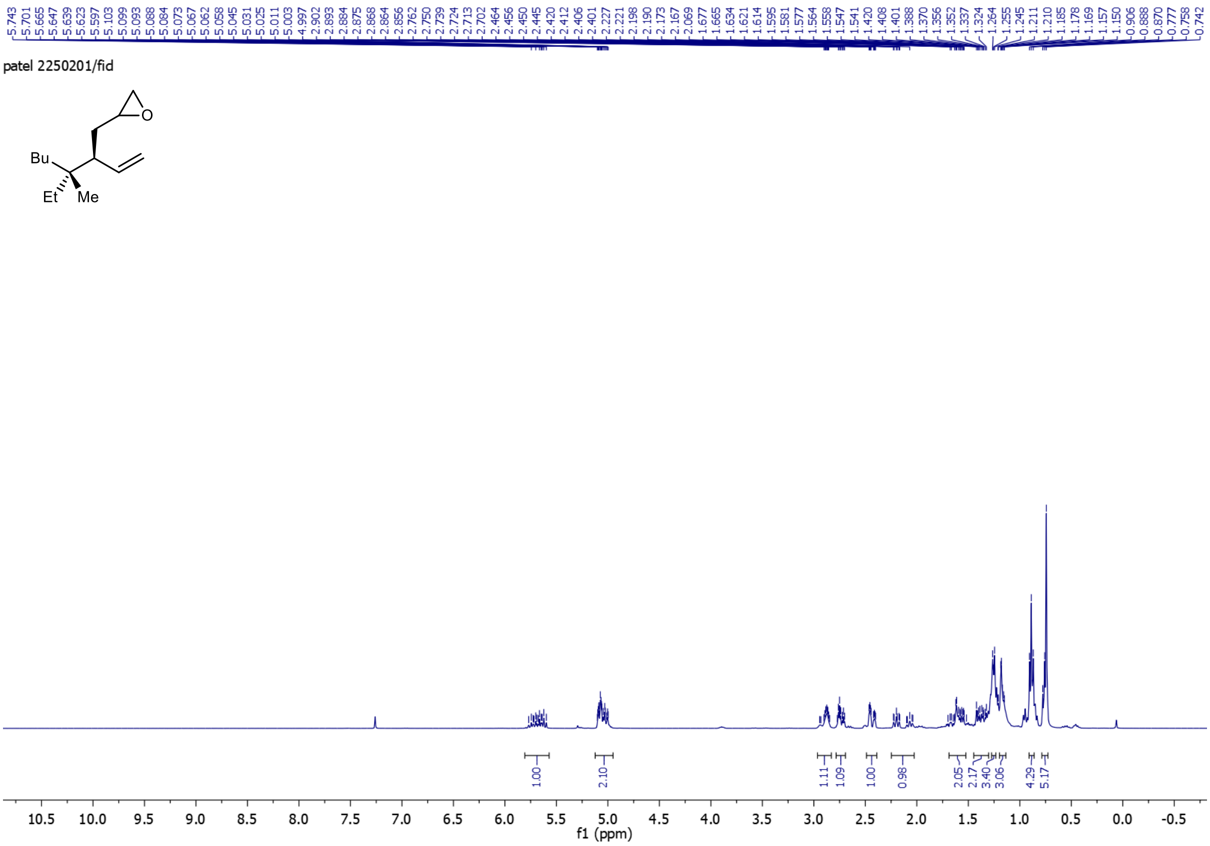
5.743 5.701 5.665 5.647 5.639 5.623 5.597 5.103 5.099 5.093 5.088 5.084 5.073 5.067 5.062 5.058 5.045 5.031 5.025 5.011 5.003 4.997 2.902 2.893 2.884 2.875 2.868 2.864 2.856 2.762 2.750 2.739 2.724 2.713 2.702 2.464 2.456 2.450 2.445 2.420 2.412 2.406 2.401 2.227 2.221 2.198 2.190 2.173 2.167 2.069 1.677 1.665 1.634 1.621 1.614 1.595 1.581 1.577 1.564 1.558 1.547 1.541 1.420 1.408 1.401 1.388 1.370 1.356 1.352 1.337 1.324 1.264 1.255 1.245 1.211 1.210 1.185 1.178 1.169 1.157 1.150 0.906 0.888 0.870 0.777 0.758 0.742
patel 2250201/fid
O
Bu
Et
Me
1.00
2.10
1.11
1.09
1.00
0.98
2.05
2.17
3.40
3.06
4.29
5.17
f1 (ppm)

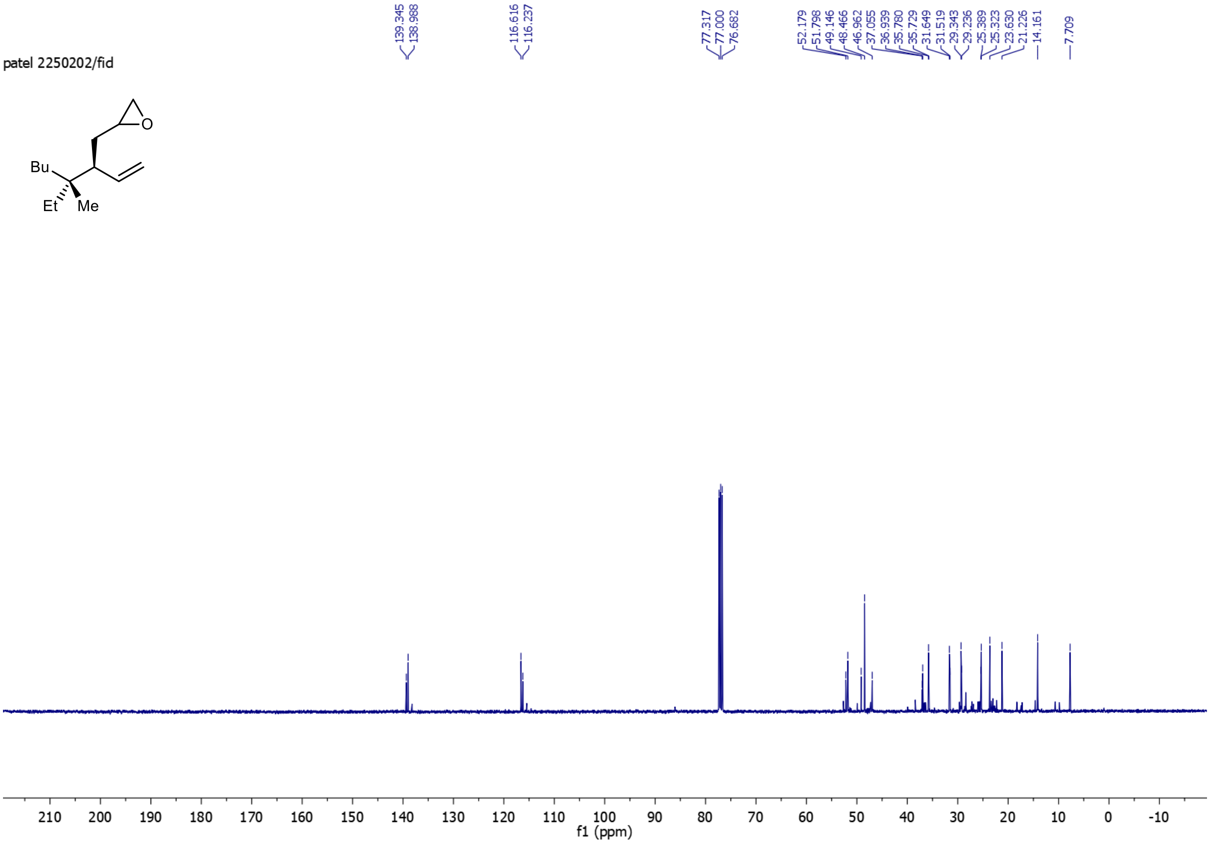
139.345 138.988
116.616 116.237
77.317 77.000 76.682
52.179 51.798 49.146 48.466 46.962 37.055 36.939 35.780 35.729 31.649 31.519 29.343 29.236 25.389 25.323 23.630 21.226 14.161
7.709
patel 2250202/fid
O
Bu
Et
Me
f1 (ppm)

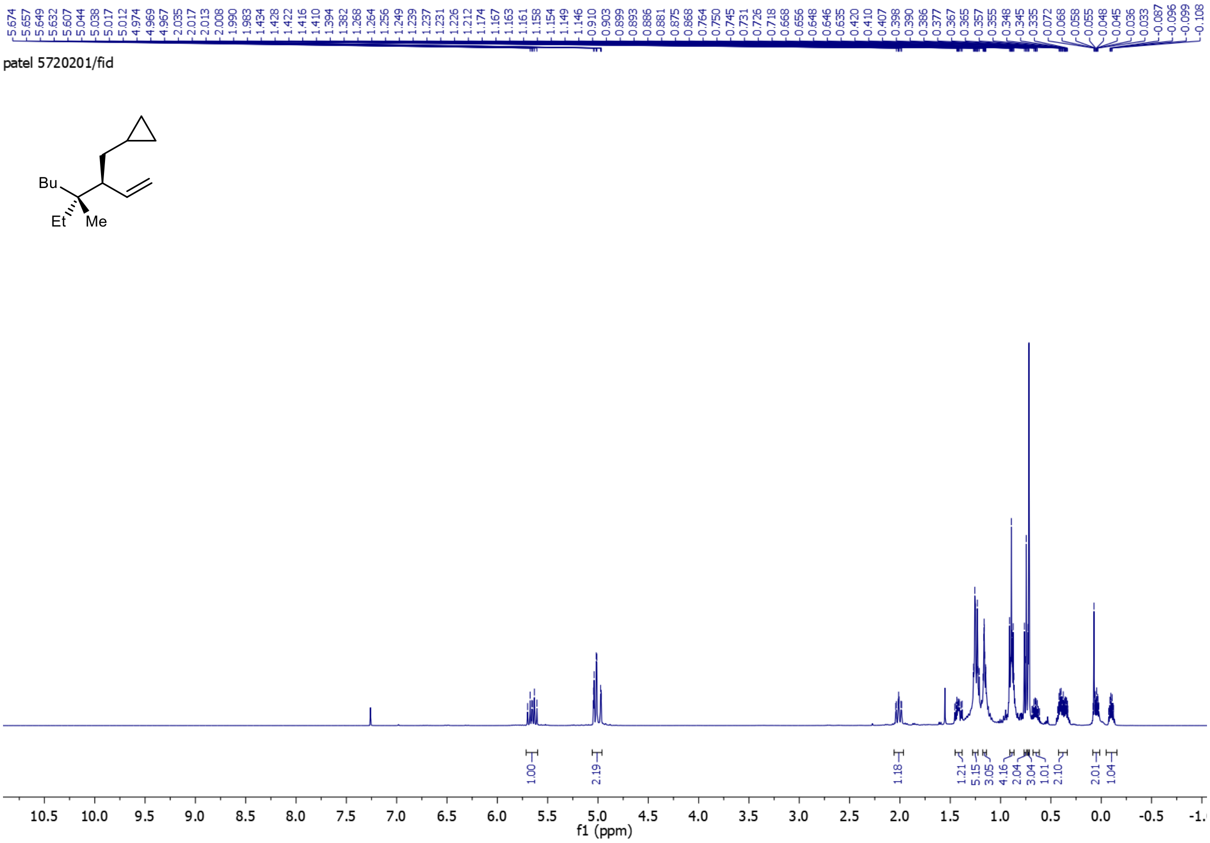
patel 5720201/fid
5.674 5.657 5.649 5.632 5.607 5.044 5.038 5.017 5.012 4.974 4.969 4.967 2.035 2.017 2.013 2.008 1.990 1.983 1.434 1.428 1.422 1.416 1.410 1.394 1.382 1.268 1.264 1.256 1.249 1.239 1.237 1.231 1.226 1.212 1.174 1.167 1.163 1.161 1.158 1.154 1.149 1.146 0.910 0.903 0.899 0.893 0.886 0.881 0.875 0.868 0.764 0.750 0.745 0.731 0.726 0.718 0.668 0.656 0.648 0.646 0.635 0.420 0.410 0.407 0.398 0.390 0.386 0.377 0.367 0.365 0.357 0.355 0.348 0.345 0.335 0.072 0.068 0.058 0.055 0.048 0.045 0.036 0.033 -0.087 -0.096 -0.099 -0.108
Bu
Et
Me
1.00
2.19
1.18
1.21
5.15
3.05
4.16
2.04
3.04
1.01
2.10
2.01
1.04
f1 (ppm)

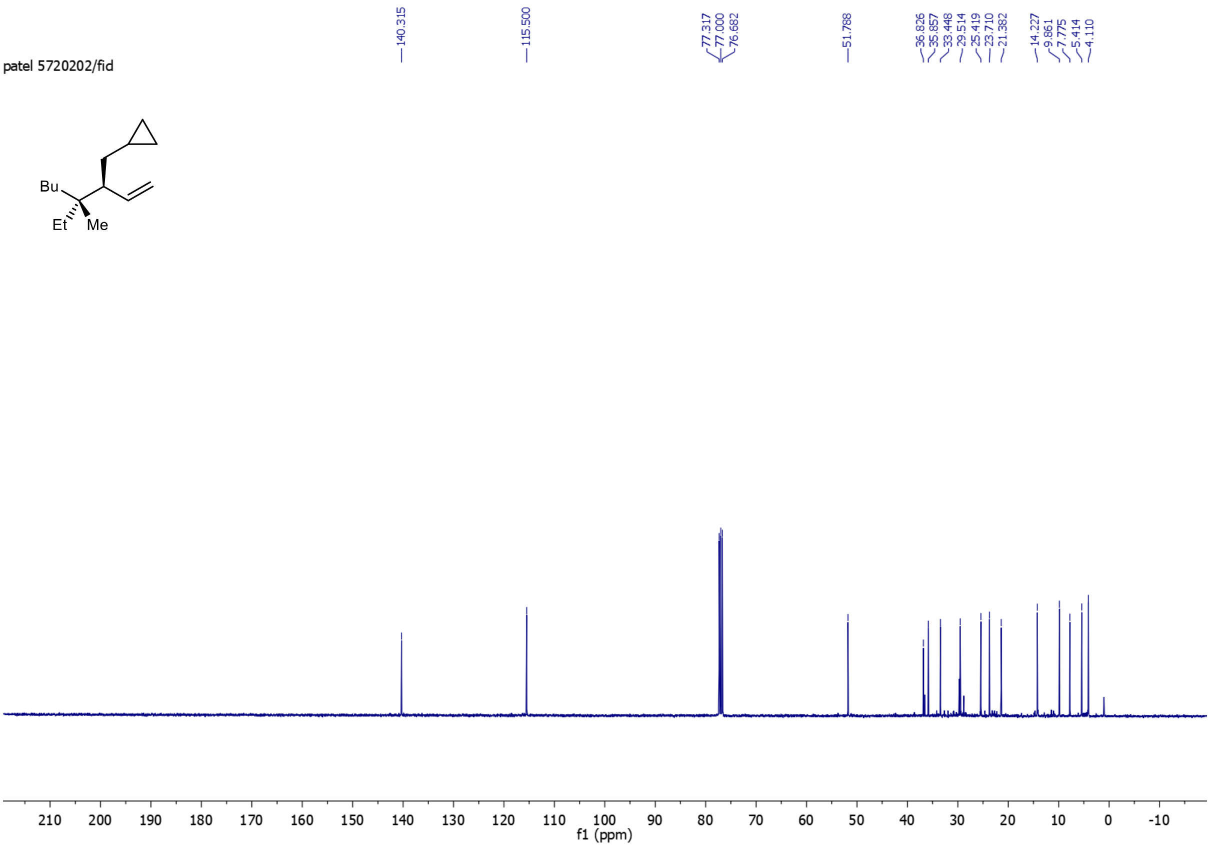
patel 5720202/fid
140.315
115.500
77.317
77.000
76.682
51.788
36.826
35.857
33.448
29.514
25.419
23.710
21.382
14.227
9.861
7.775
5.414
4.110
Bu
Et
Me
f1 (ppm)

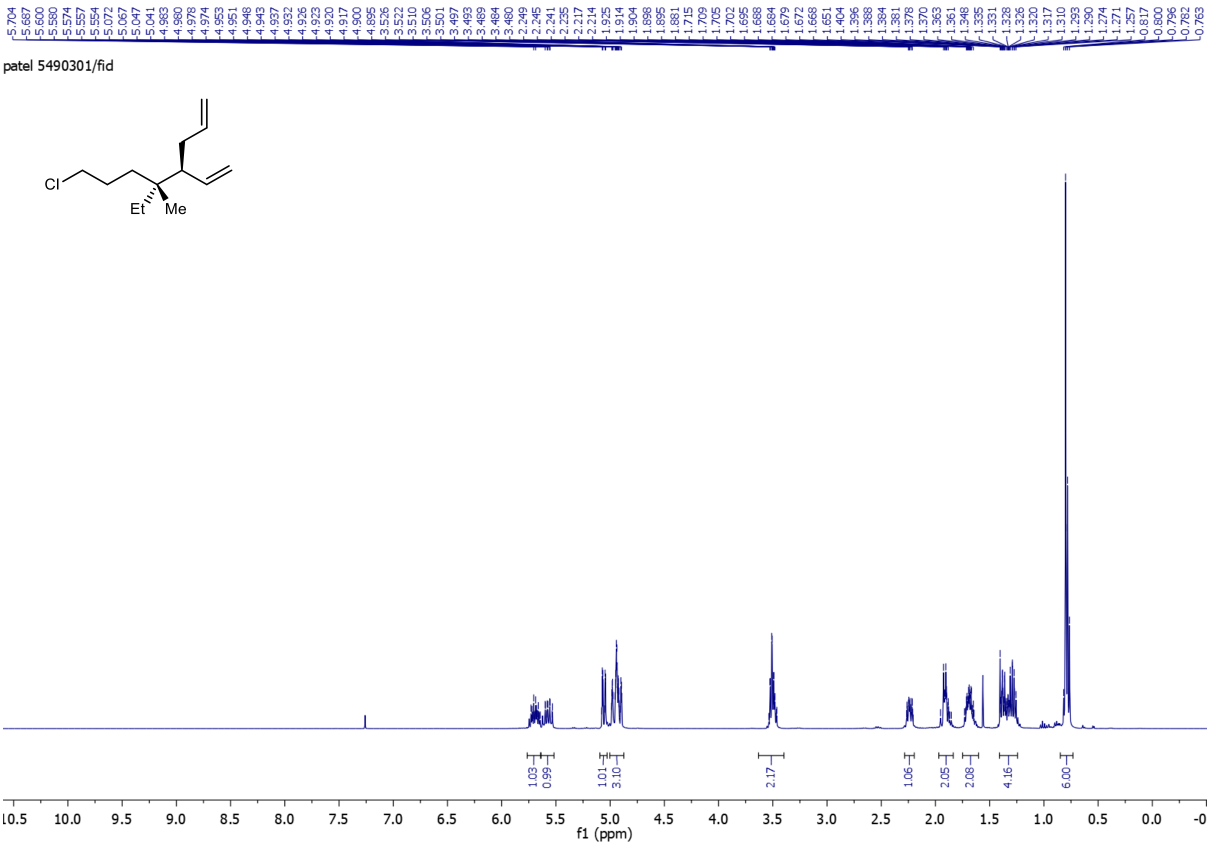
5.704 5.687 5.600 5.580 5.574 5.557 5.554 5.072 5.067 5.047 5.041 4.983 4.980 4.978 4.974 4.953 4.951 4.948 4.943 4.937 4.932 4.926 4.923 4.920 4.917 4.900 4.895 3.526 3.522 3.510 3.506 3.501 3.497 3.493 3.489 3.484 3.480 2.249 2.245 2.241 2.235 2.217 2.214 1.925 1.914 1.904 1.898 1.895 1.881 1.715 1.709 1.705 1.702 1.695 1.688 1.684 1.679 1.672 1.668 1.651 1.404 1.396 1.388 1.384 1.381 1.378 1.370 1.363 1.361 1.348 1.335 1.331 1.328 1.326 1.320 1.317 1.310 1.293 1.290 1.274 1.271 1.257 0.817 0.800 0.796 0.782 0.763
patel 5490301/fid
Cl
Et
Me
1.03
0.99
1.01
3.10
2.17
1.06
2.05
2.08
4.16
6.00
f1 (ppm)

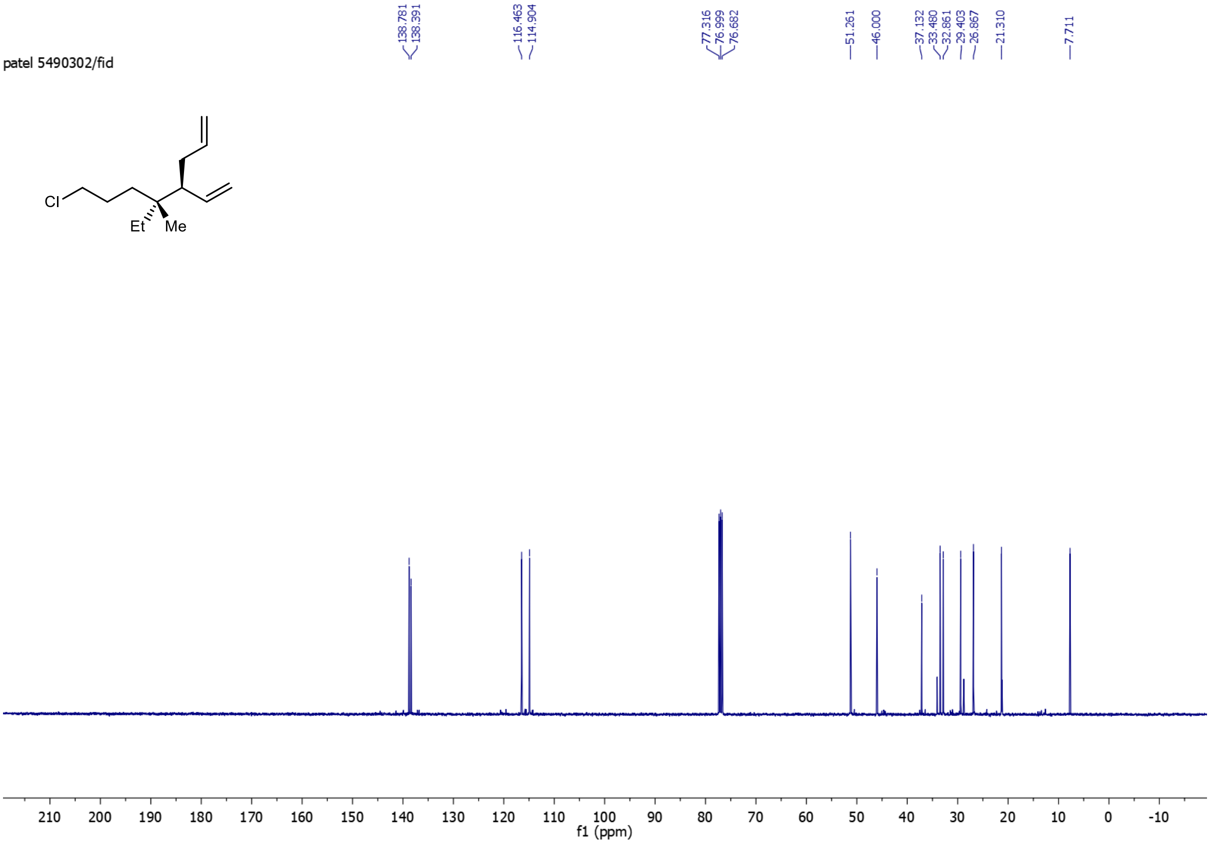
138.781 138.391 116.463 114.904 77.316 76.999 76.682 51.261 46.000 37.132 33.480 32.861 29.403 26.867 21.310 7.711
patel 5490302/fid
Cl
Et
Me
f1 (ppm)

veeru 115390201/fid

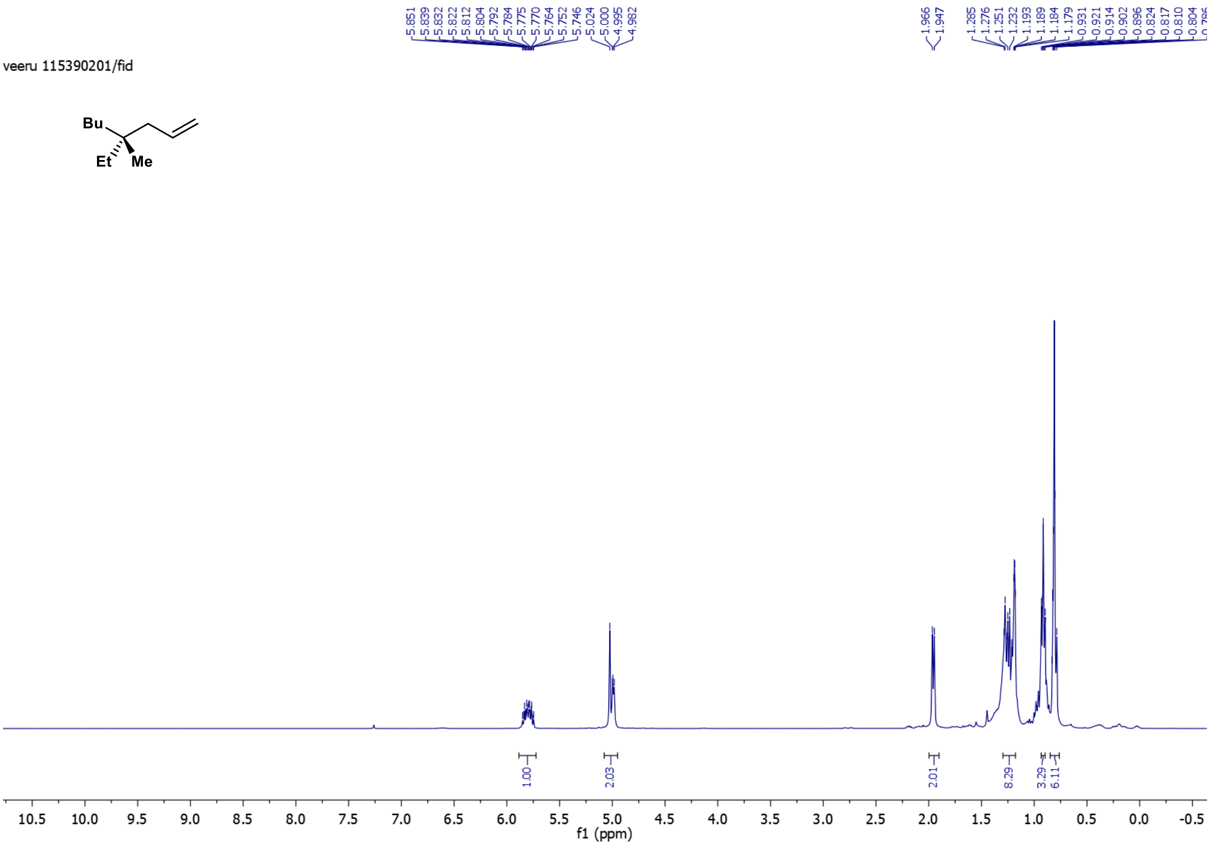

veeru 115390202/fid

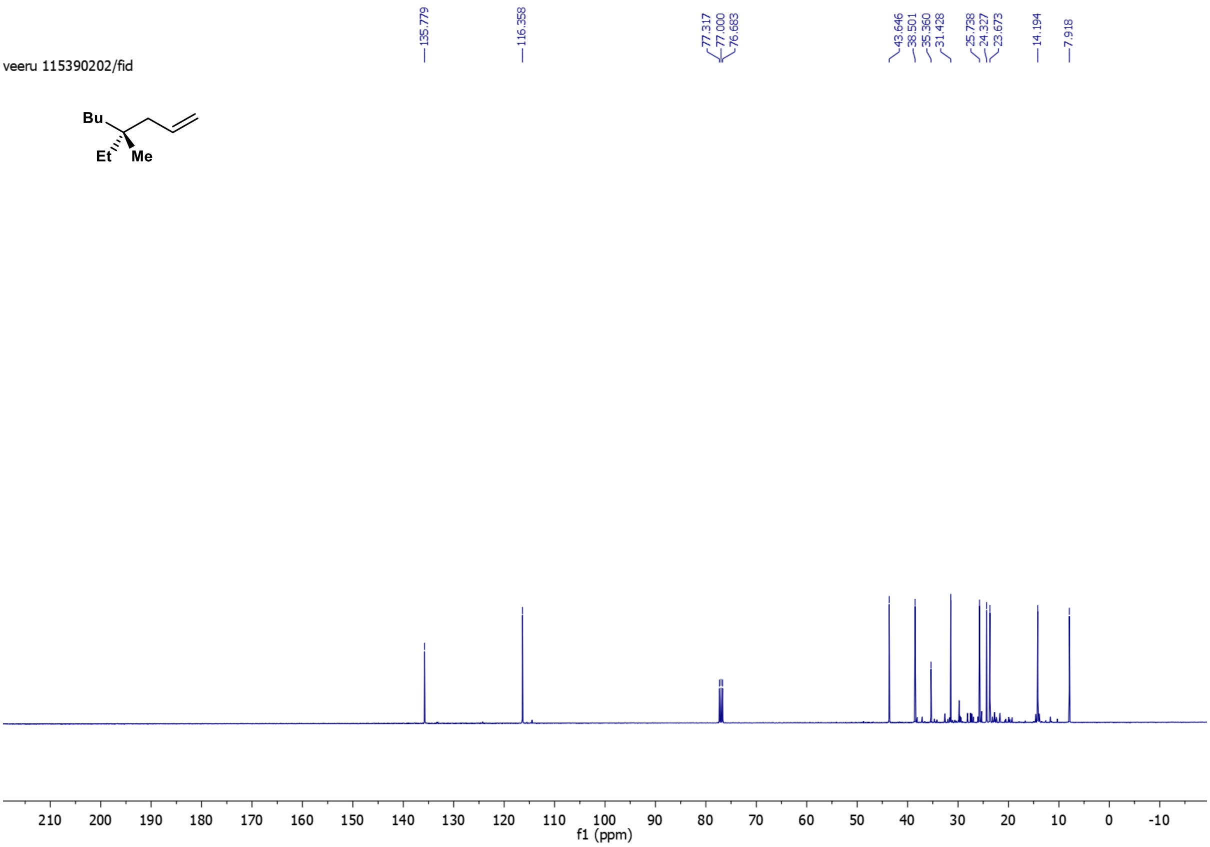

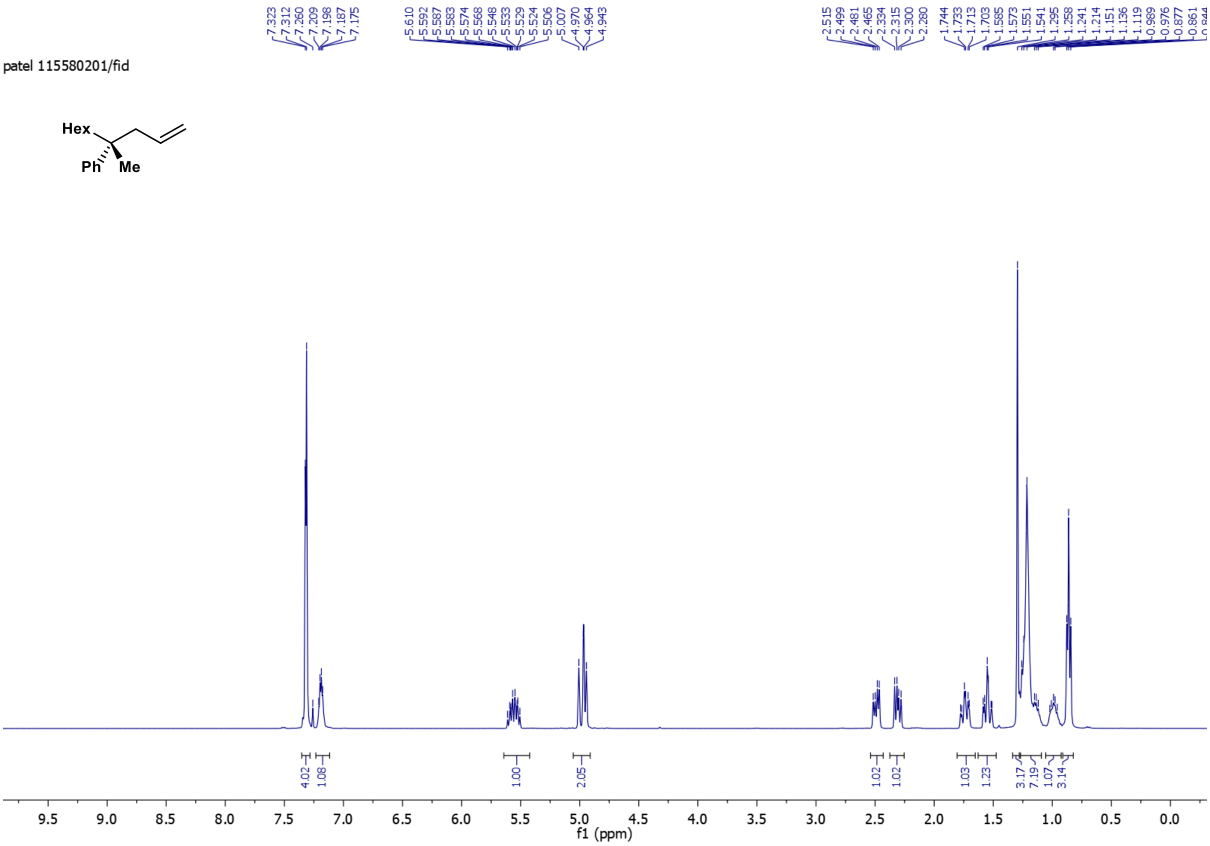
patel 115580201/fid
Hex
Ph
Me
7.323 7.312 7.260 7.209 7.198 7.187 7.175
5.610 5.592 5.587 5.583 5.574 5.568 5.548 5.533 5.529 5.524 5.506 5.007 4.970 4.964 4.943
2.515 2.499 2.481 2.465 2.334 2.315 2.300 2.280 1.744 1.733 1.713 1.703 1.585 1.573 1.551 1.541 1.295 1.258 1.241 1.214 1.151 1.136 1.119 0.989 0.976 0.877 0.861
4.02 1.08 1.00 2.05 1.02 1.02 1.03 1.23 3.17 7.19 1.07 3.14
f1 (ppm)

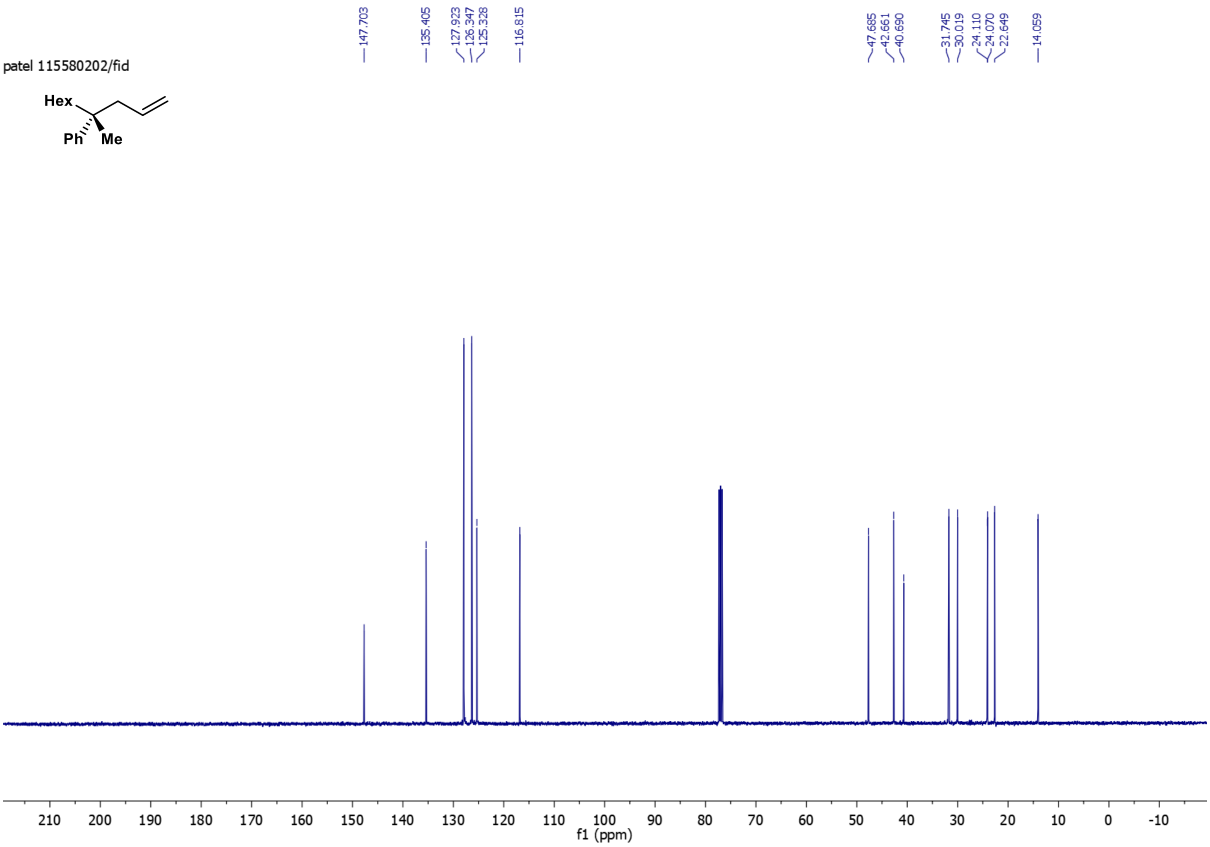
patel 115580202/fid
Hex
Ph
Me
147.703
135.405
127.923 126.347 125.328
116.815
47.685 42.661 40.690
31.745 30.019
24.110 24.070 22.649
14.059
f1 (ppm)

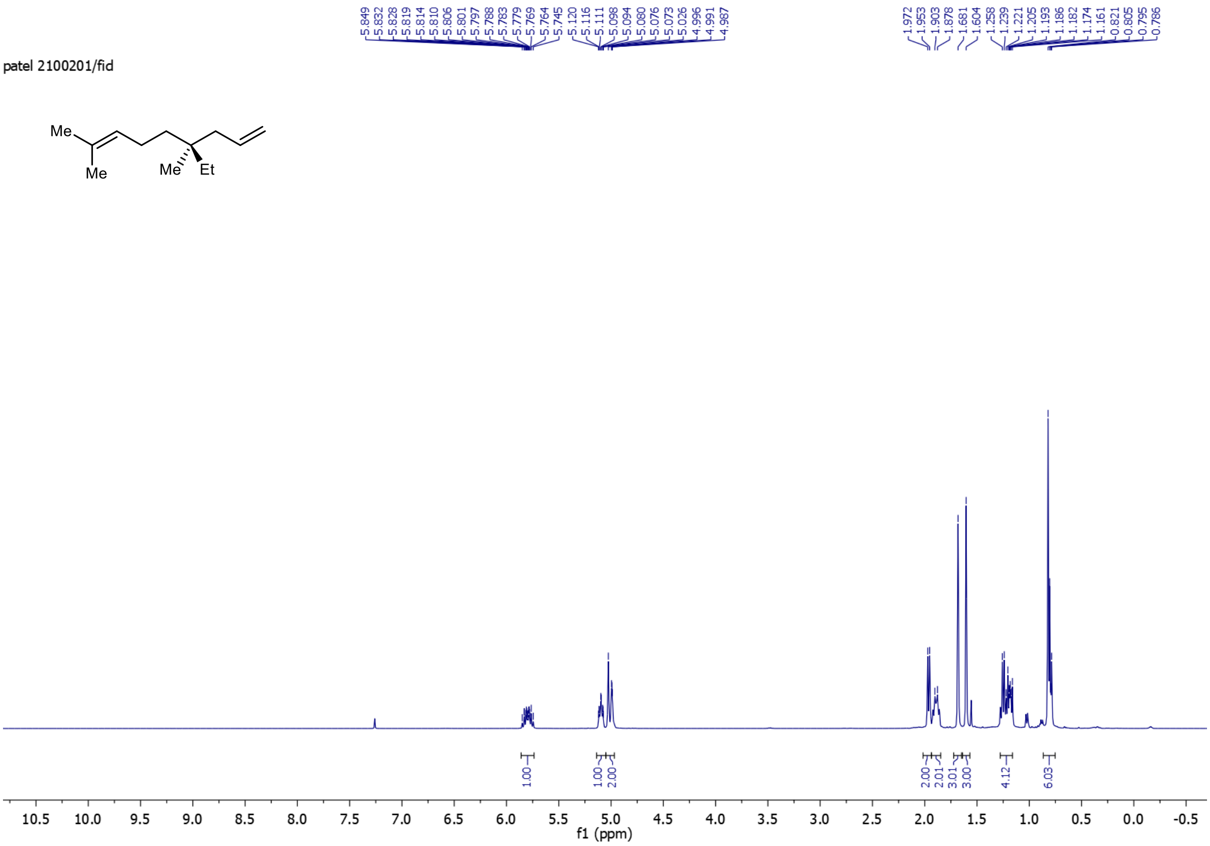

patel 2100201/fid
Me
Me
Me
Et
5.849 5.832 5.828 5.819 5.814 5.810 5.806 5.801 5.797 5.788 5.783 5.779 5.769 5.764 5.745 5.120 5.116 5.111 5.098 5.094 5.080 5.076 5.073 5.026 4.996 4.991 4.987
1.972 1.953 1.903 1.878 1.681 1.604 1.258 1.239 1.221 1.205 1.193 1.186 1.182 1.174 1.161 0.821 0.805 0.795 0.786
1.00
1.00
2.00
2.00
2.01
3.01
3.00
4.12
6.03
10.5 10.0 9.5 9.0 8.5 8.0 7.5 7.0 6.5 6.0 5.5 5.0 4.5 4.0 3.5 3.0 2.5 2.0 1.5 1.0 0.5 0.0 -0.5
f1 (ppm)

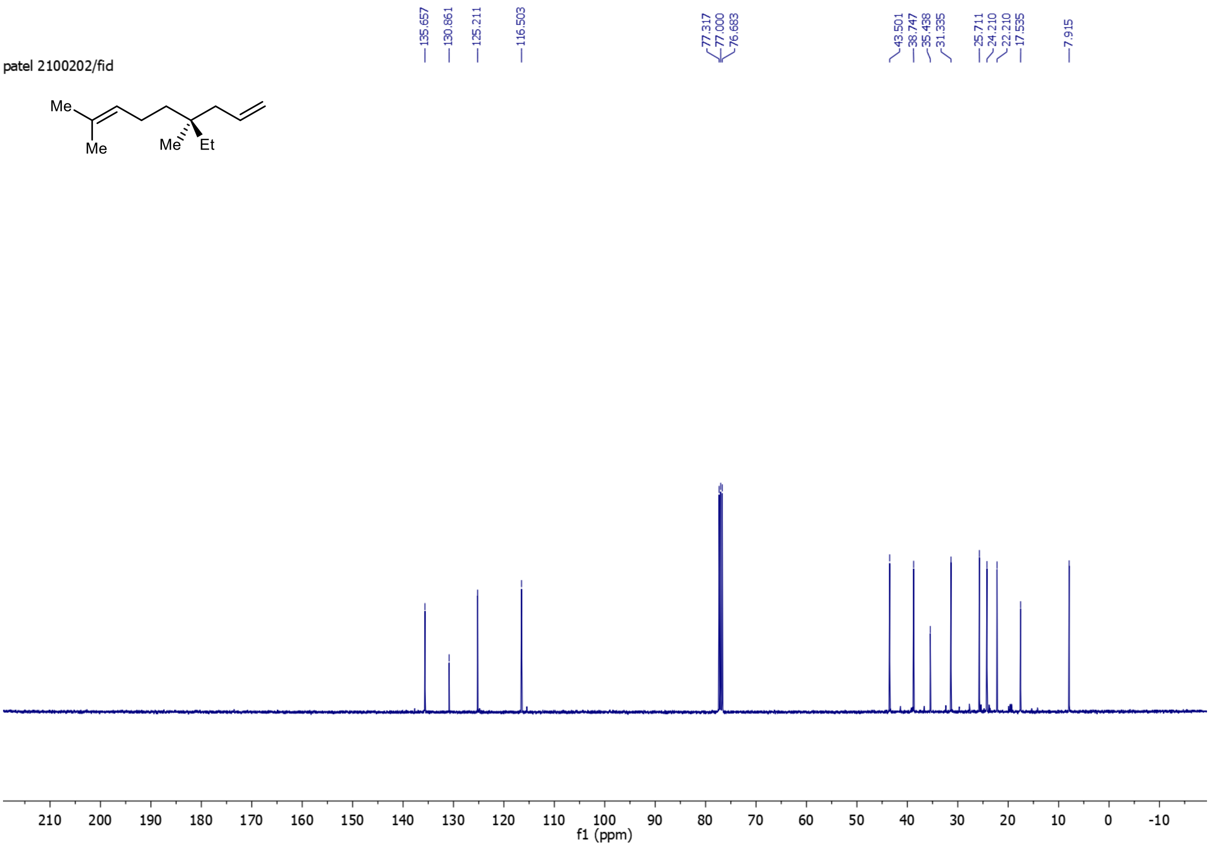

patel 2100202/fid
Me
Me
Me
Et
135.657 130.861 125.211 116.503
77.317 77.000 76.683
43.501 38.747 35.438 31.335
25.711 24.210 22.210 17.535
7.915
210 200 190 180 170 160 150 140 130 120 110 100 90 80 70 60 50 40 30 20 10 0 -10
f1 (ppm)

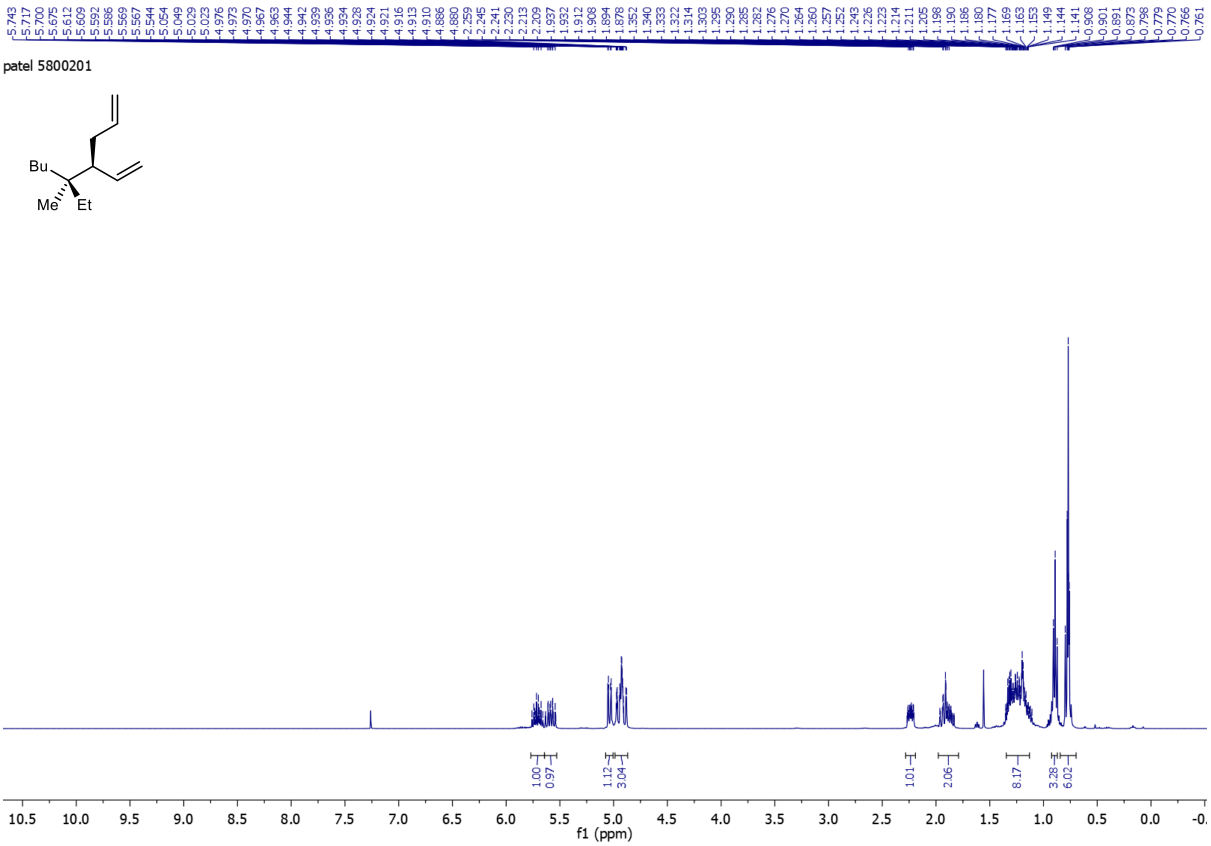
5.743 5.717 5.700 5.675 5.612 5.609 5.592 5.586 5.569 5.567 5.544 5.054 5.049 5.029 5.023 4.976 4.973 4.970 4.967 4.963 4.944 4.942 4.939 4.936 4.934 4.928 4.924 4.921 4.916 4.913 4.910 4.886 4.880 2.259 2.245 2.241 2.230 2.213 2.209 1.937 1.932 1.912 1.908 1.894 1.878 1.352 1.340 1.333 1.322 1.314 1.303 1.295 1.290 1.285 1.282 1.276 1.270 1.264 1.260 1.257 1.252 1.243 1.226 1.223 1.214 1.211 1.205 1.198 1.190 1.186 1.180 1.177 1.169 1.163 1.153 1.149 1.144 1.141 0.908 0.901 0.891 0.873 0.798 0.779 0.770 0.766 0.761
patel 5800201
Bu
Me
Et
1.00
0.97
1.12
3.04
1.01
2.06
8.17
3.28
6.02
f1 (ppm)

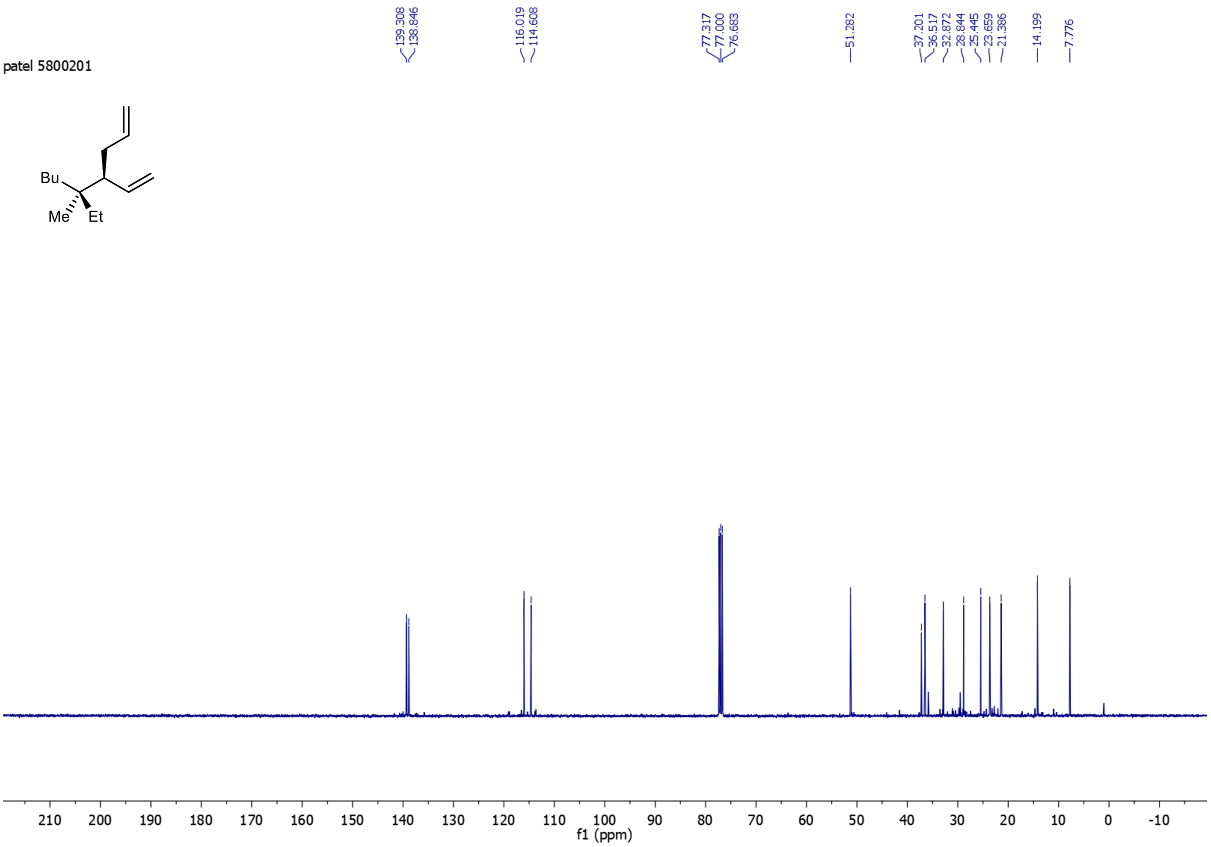
139.308 138.846
116.019 114.608
77.317 77.000 76.683
51.282
37.201 36.517 32.872 28.844 25.445 23.659 21.386
14.199
7.776
patel 5800201
Bu
Me
Et
f1 (ppm)

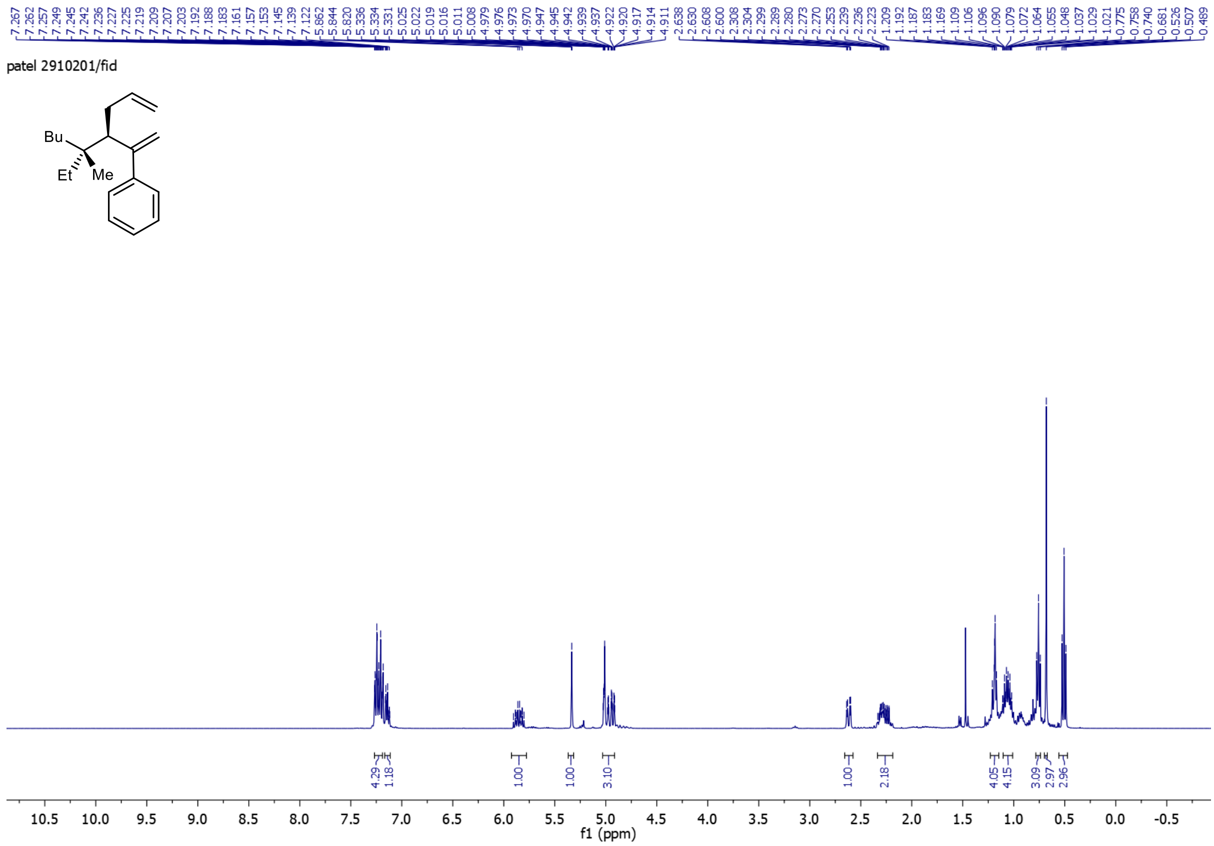
7.267 7.262 7.257 7.249 7.245 7.242 7.236 7.227 7.225 7.219 7.209 7.207 7.203 7.192 7.188 7.183 7.161 7.157 7.153 7.145 7.139 7.122 5.862 5.844 5.820 5.336 5.334 5.331 5.025 5.022 5.019 5.016 5.011 5.008 4.979 4.976 4.973 4.970 4.947 4.945 4.942 4.939 4.937 4.922 4.920 4.917 4.914 4.911 2.638 2.630 2.608 2.600 2.308 2.304 2.299 2.289 2.280 2.273 2.270 2.253 2.239 2.236 2.223 1.209 1.192 1.187 1.183 1.169 1.109 1.106 1.096 1.090 1.079 1.072 1.064 1.055 1.048 1.037 1.029 1.021 0.775 0.758 0.740 0.681 0.526 0.507 0.489
patel 2910201/fid
Bu
Et
Me
4.29 1.18 1.00 1.00 3.10 1.00 2.18 4.05 4.15 3.09 2.97 2.96
10.5 10.0 9.5 9.0 8.5 8.0 7.5 7.0 6.5 6.0 5.5 5.0 4.5 4.0 3.5 3.0 2.5 2.0 1.5 1.0 0.5 0.0 -0.5
f1 (ppm)

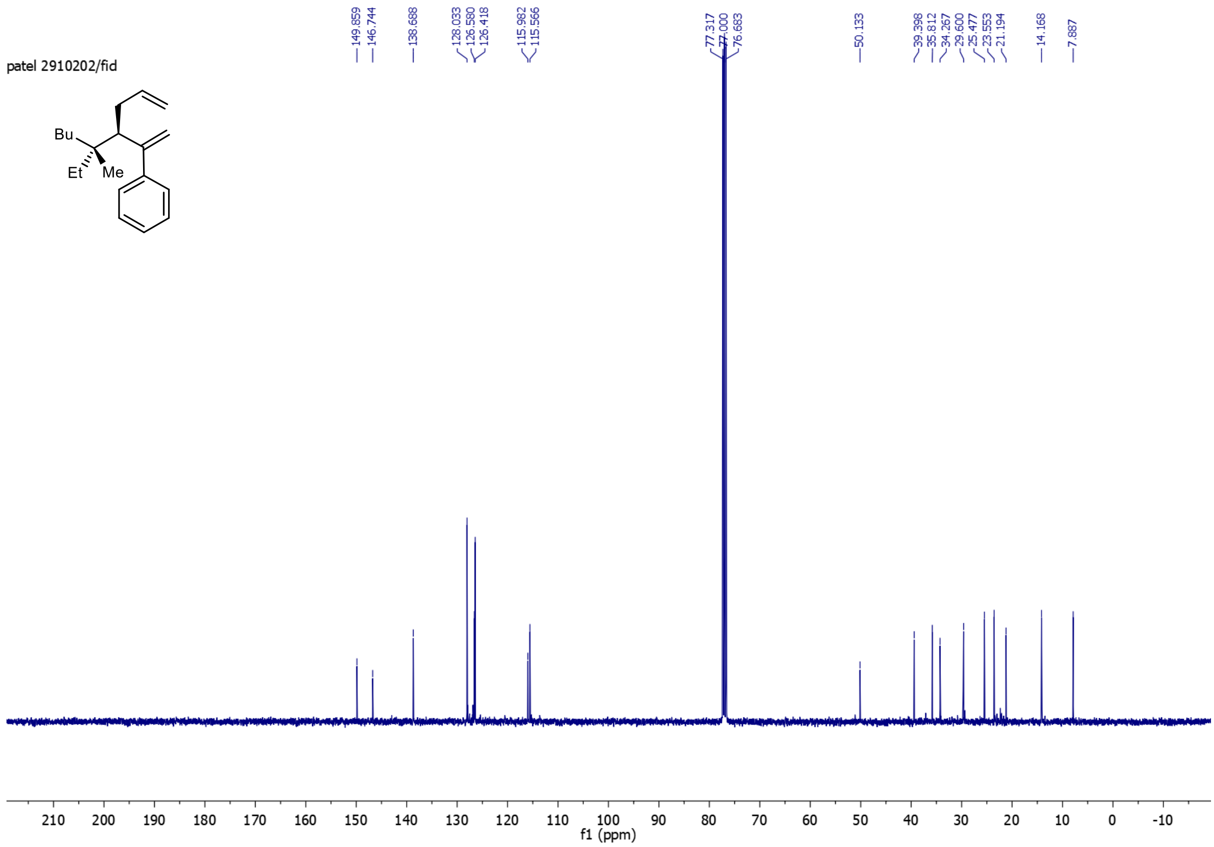
149.859 146.744 138.688 128.033 126.580 126.418 115.982 115.566 77.317 77.000 76.683 50.133 39.398 35.812 34.267 29.600 25.477 23.553 21.194 14.168 7.887
patel 2910202/fid
Bu
Et
Me
210 200 190 180 170 160 150 140 130 120 110 100 90 80 70 60 50 40 30 20 10 0 -10
f1 (ppm)

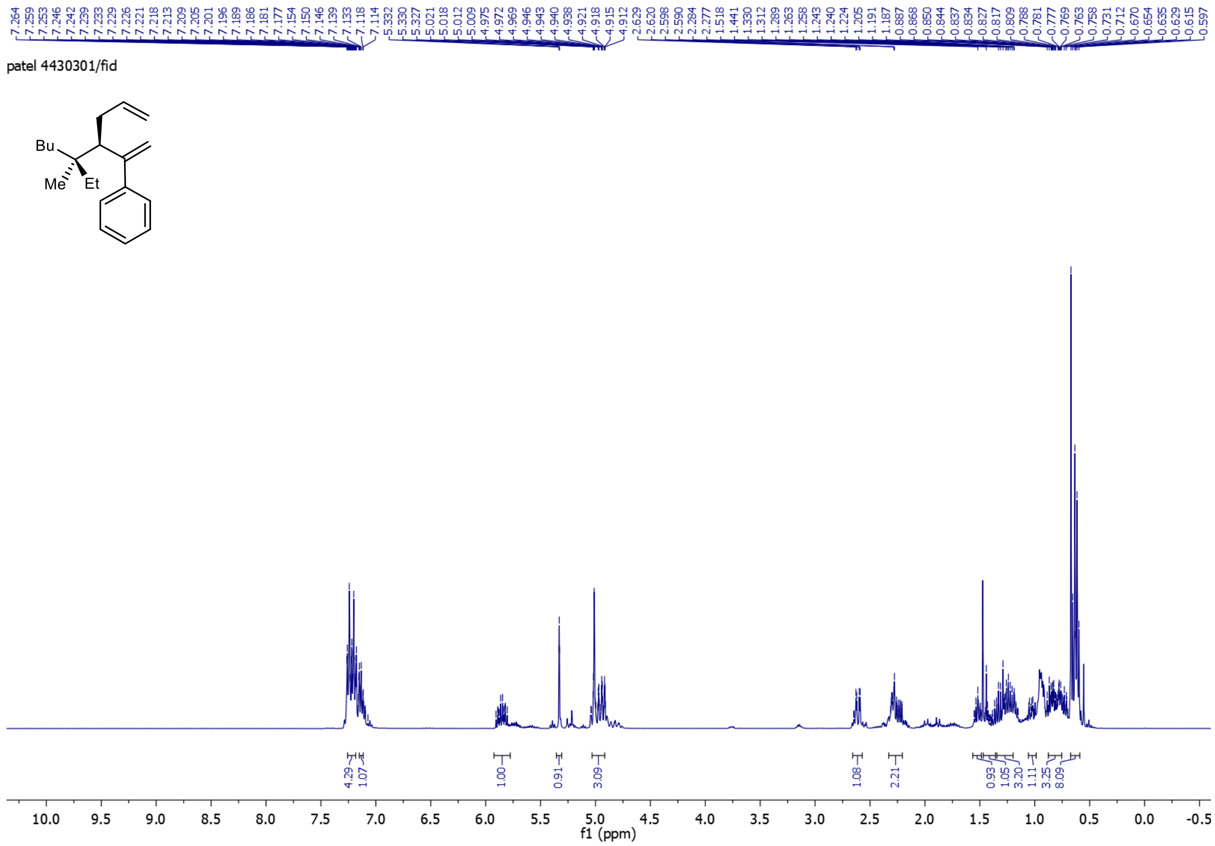
7.264 7.259 7.253 7.246 7.242 7.239 7.233 7.229 7.226 7.221 7.218 7.213 7.209 7.205 7.201 7.196 7.189 7.186 7.181 7.177 7.154 7.150 7.146 7.139 7.133 7.118 7.114 5.332 5.330 5.327 5.021 5.018 5.012 5.009 4.975 4.972 4.969 4.946 4.943 4.940 4.938 4.921 4.918 4.915 4.912 2.629 2.620 2.598 2.590 2.284 2.277 1.518 1.441 1.330 1.312 1.269 1.263 1.258 1.243 1.240 1.224 1.205 1.191 1.187 0.887 0.868 0.850 0.844 0.837 0.834 0.827 0.817 0.809 0.788 0.781 0.777 0.769 0.763 0.758 0.731 0.712 0.670 0.654 0.635 0.629 0.615 0.597
patel 4430301/fid
Bu
Me
Et
4.29 1.07 1.00 0.91 3.09 1.08 2.21 0.93 1.05 3.20 1.11 3.25 8.09
10.0 9.5 9.0 8.5 8.0 7.5 7.0 6.5 6.0 5.5 5.0 4.5 4.0 3.5 3.0 2.5 2.0 1.5 1.0 0.5 0.0 -0.5
f1 (ppm)

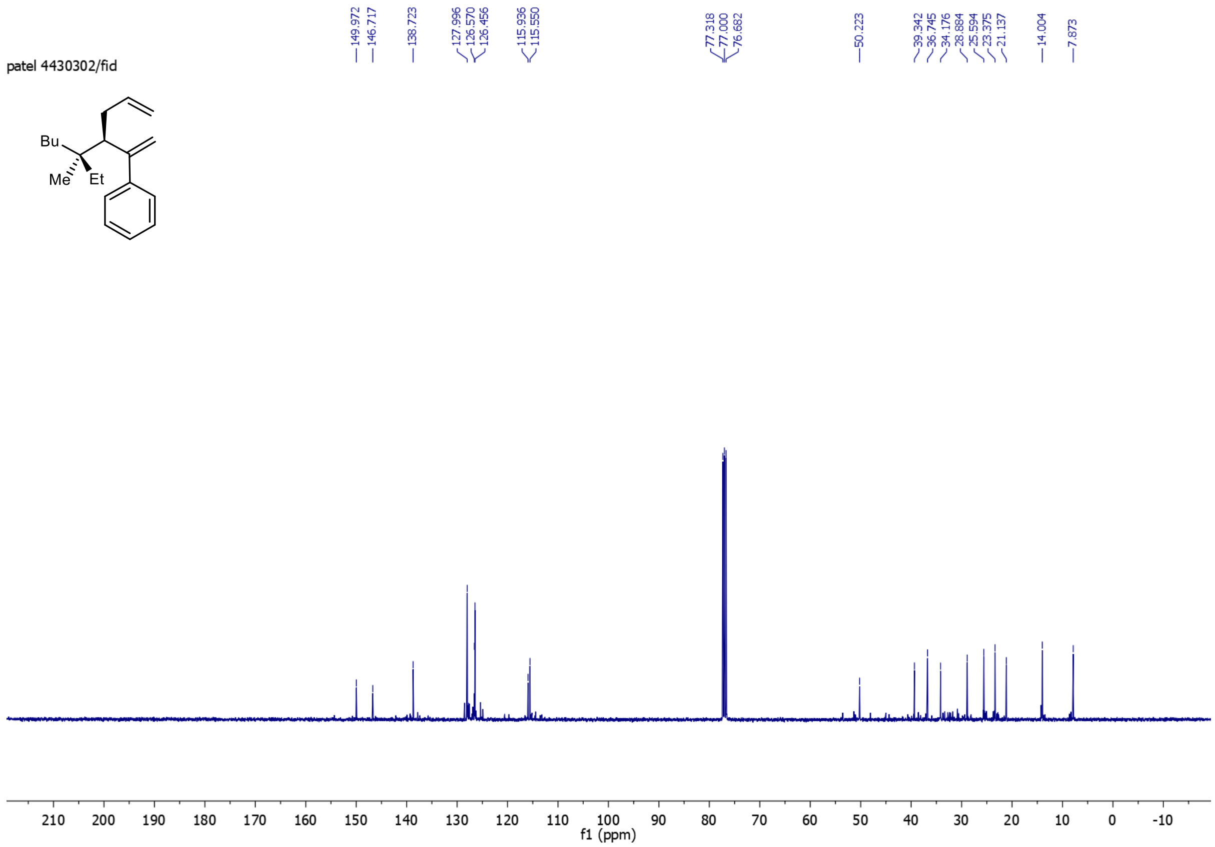
149.972 146.717 138.723 127.996 126.570 126.456 115.936 115.550 77.318 77.000 76.682 50.223 39.342 36.745 34.176 28.884 25.594 23.375 21.137 14.004 7.873
patel 4430302/fid
Bu
Me
Et
210 200 190 180 170 160 150 140 130 120 110 100 90 80 70 60 50 40 30 20 10 0 -10
f1 (ppm)

patel 3750301/fid

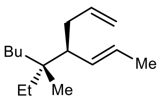

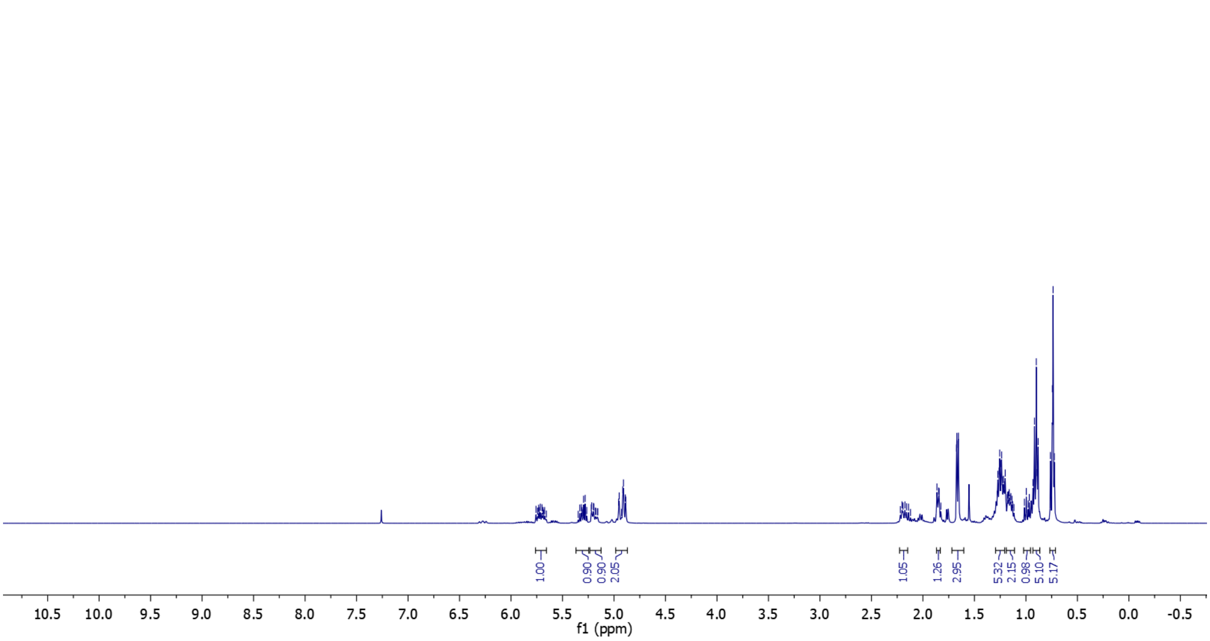

patel 3750302/fid

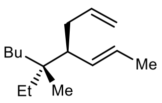

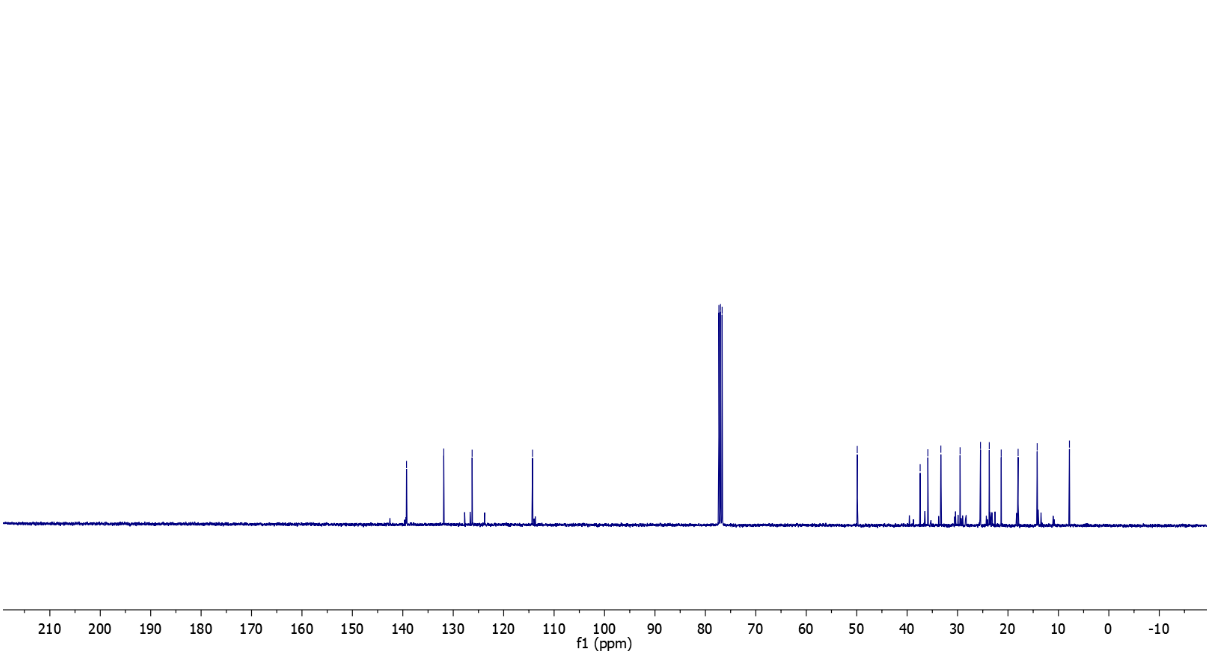

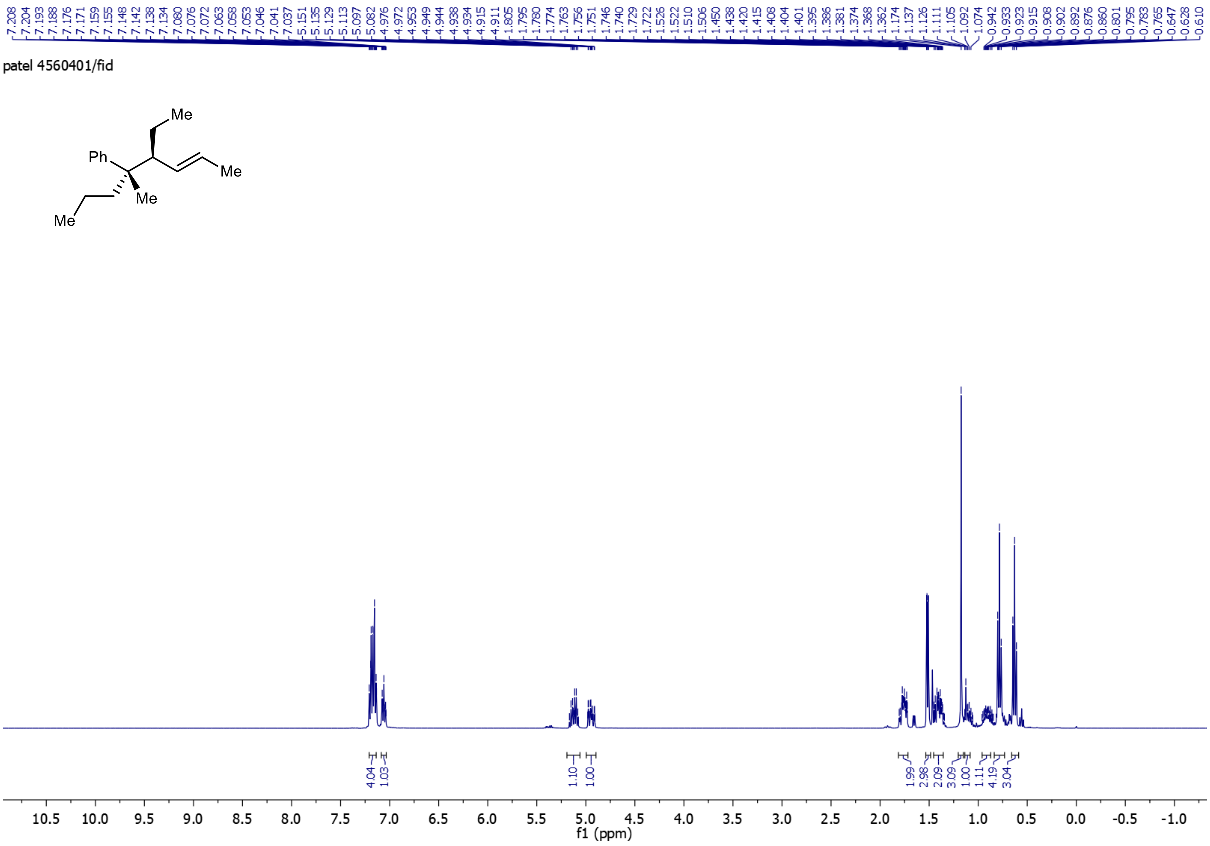
7.208 7.204 7.193 7.188 7.176 7.171 7.159 7.155 7.148 7.142 7.138 7.134 7.080 7.076 7.072 7.063 7.058 7.053 7.046 7.041 7.037 5.151 5.135 5.129 5.113 5.097 5.082 4.976 4.972 4.953 4.949 4.944 4.938 4.934 4.915 4.911 1.805 1.795 1.780 1.774 1.763 1.756 1.751 1.746 1.740 1.729 1.722 1.526 1.522 1.510 1.506 1.450 1.438 1.420 1.415 1.408 1.404 1.401 1.395 1.386 1.381 1.374 1.368 1.362 1.174 1.137 1.126 1.111 1.105 1.092 1.074 0.942 0.933 0.923 0.915 0.908 0.902 0.892 0.876 0.860 0.801 0.795 0.783 0.765 0.647 0.628 0.610
patel 4560401/fid
Me
Ph
Me
Me
Me
4.04 1.03 1.10 1.00 1.99 2.98 2.09 3.09 1.00 1.11 4.19 3.04
10.5 10.0 9.5 9.0 8.5 8.0 7.5 7.0 6.5 6.0 5.5 5.0 4.5 4.0 3.5 3.0 2.5 2.0 1.5 1.0 0.5 0.0 -0.5 -1.0
f1 (ppm)

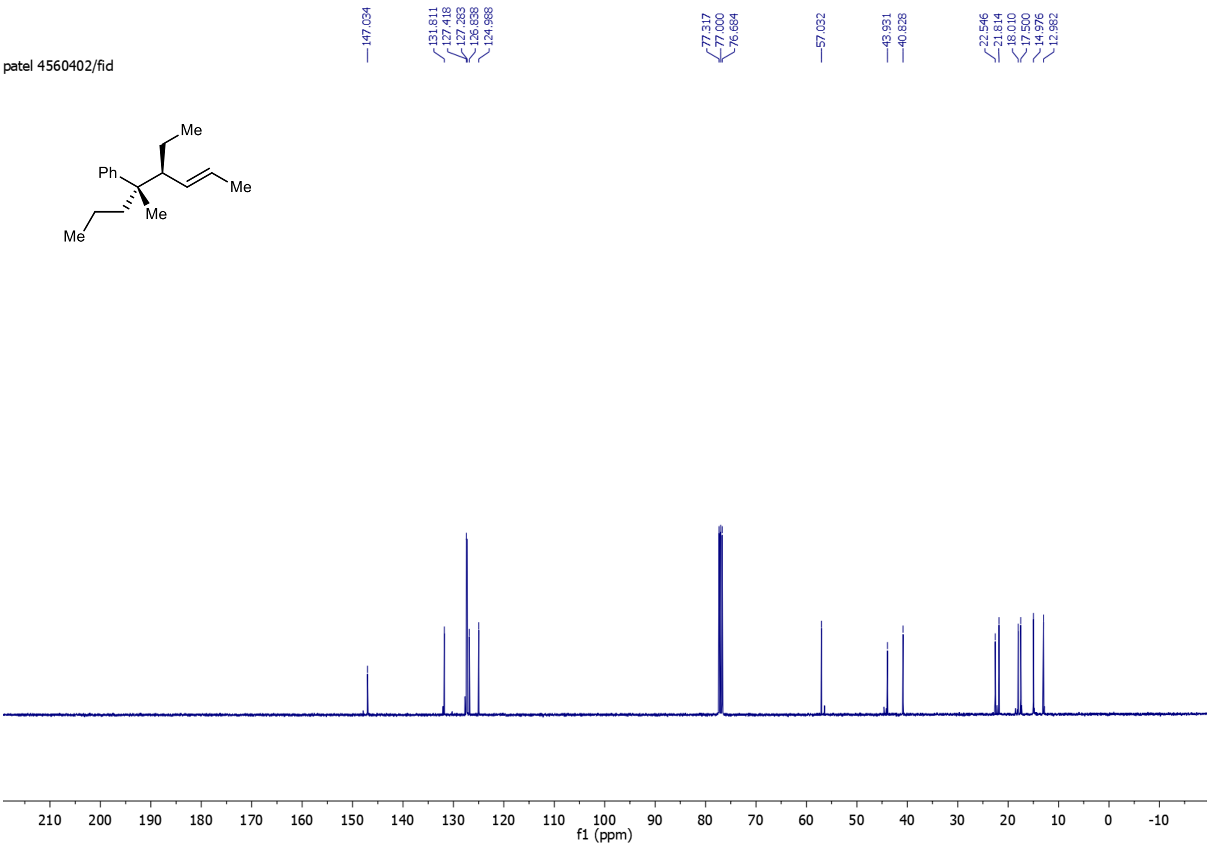
147.034
131.811 127.418 127.283 126.838 124.988
77.317 77.000 76.684
57.032
43.931 40.828
22.546 21.814 18.010 17.500 14.976 12.982
patel 4560402/fid
Me
Ph
Me
Me
Me
210 200 190 180 170 160 150 140 130 120 110 100 90 80 70 60 50 40 30 20 10 0 -10
f1 (ppm)

patel 3760201/fid

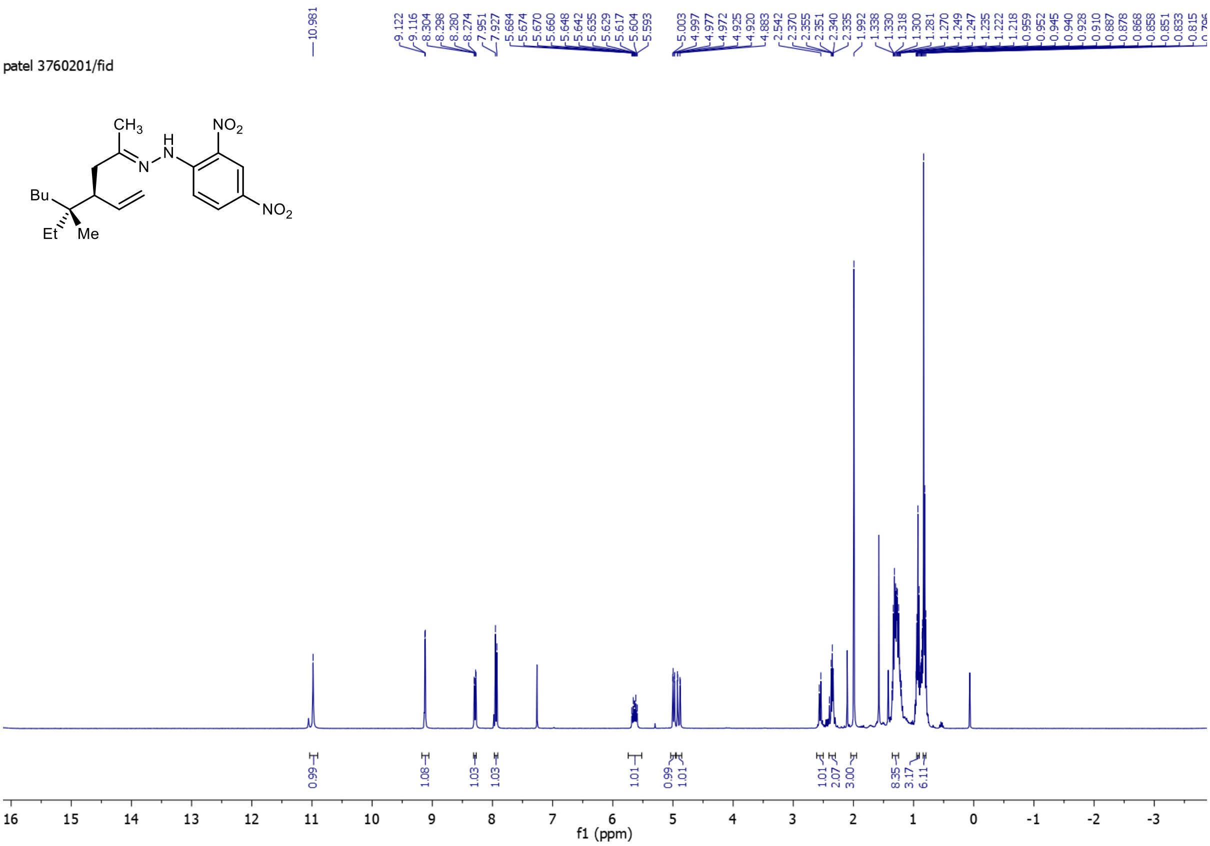

patel 3760202/fid

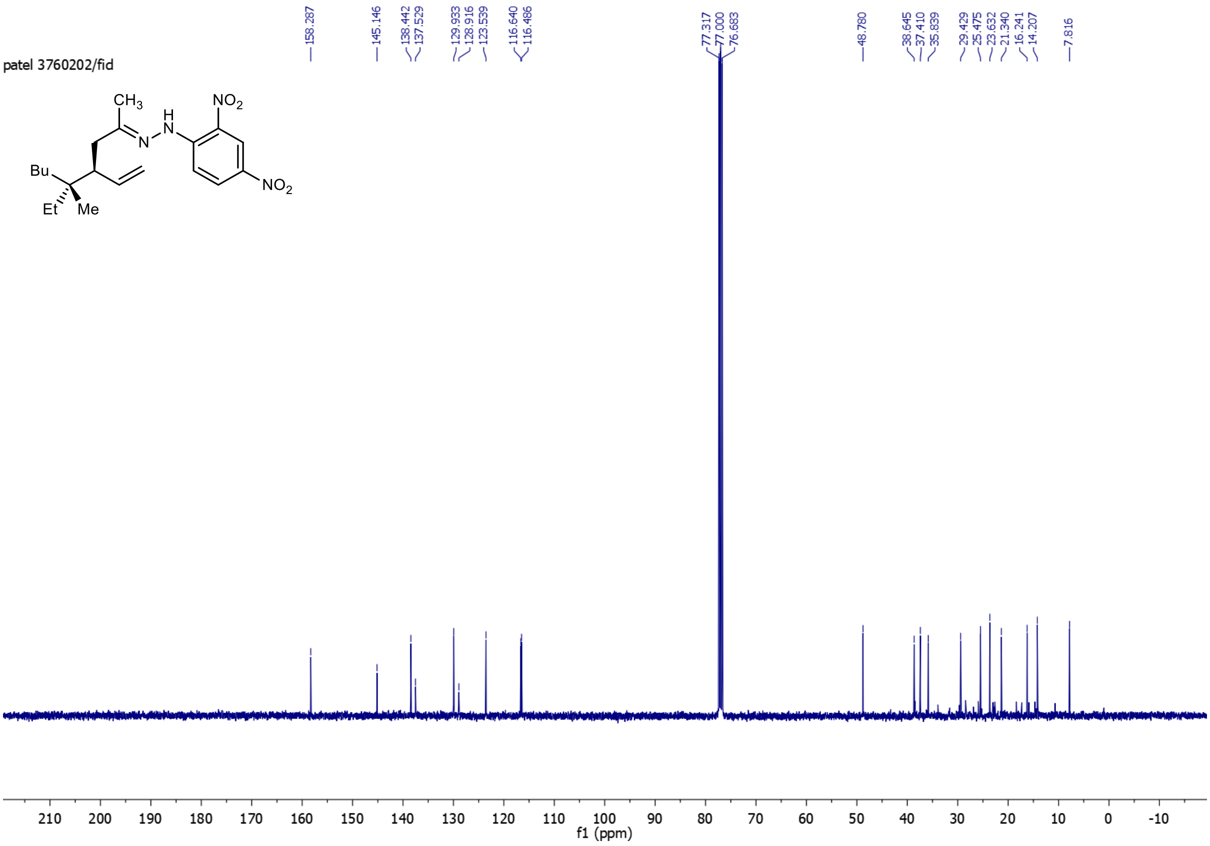

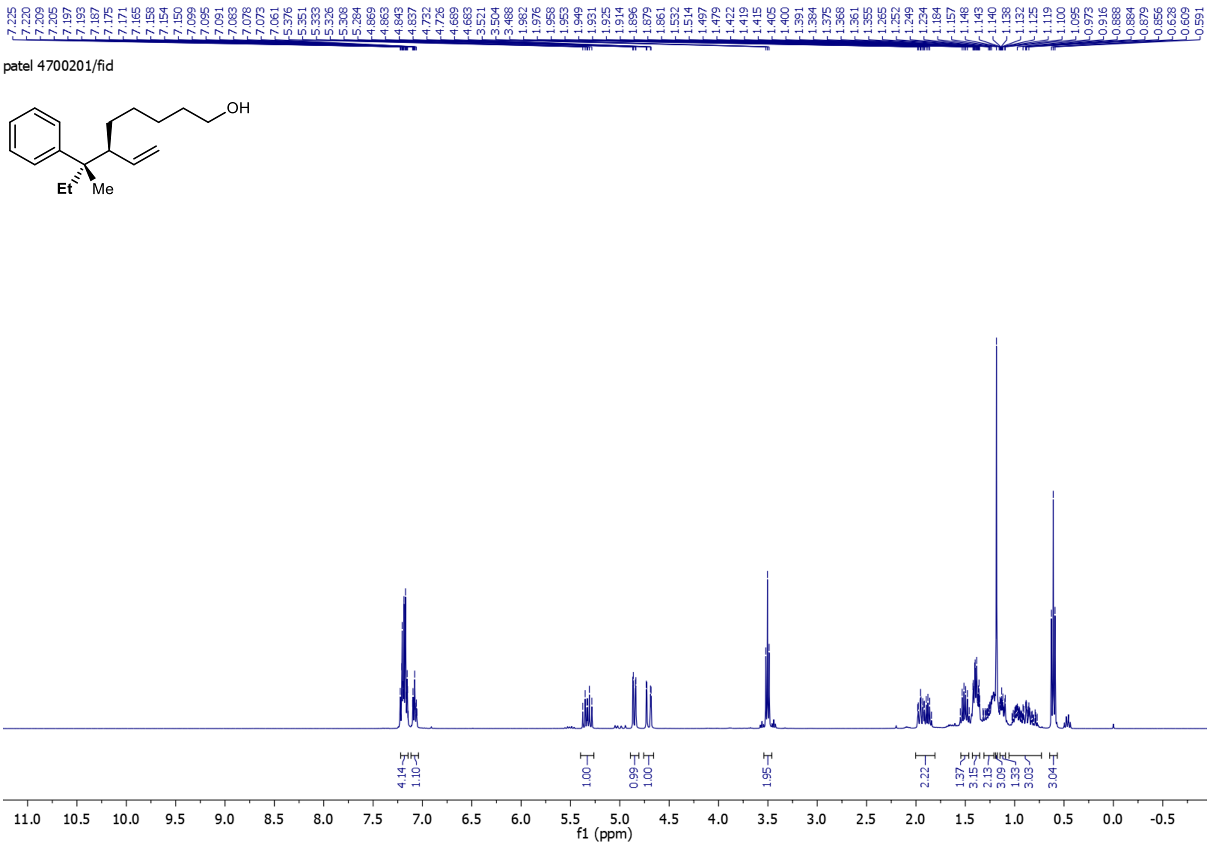
7.225 7.220 7.209 7.205 7.197 7.193 7.187 7.175 7.171 7.165 7.158 7.154 7.150 7.099 7.095 7.091 7.083 7.078 7.073 7.061 5.376 5.351 5.333 5.326 5.308 5.284 4.869 4.863 4.843 4.837 4.732 4.726 4.689 4.683 3.521 3.504 3.488 1.982 1.976 1.958 1.953 1.949 1.931 1.925 1.914 1.896 1.879 1.861 1.532 1.514 1.497 1.479 1.422 1.419 1.415 1.405 1.400 1.391 1.384 1.375 1.368 1.361 1.355 1.265 1.252 1.249 1.234 1.184 1.157 1.148 1.143 1.140 1.138 1.132 1.125 1.119 1.100 1.095 0.973 0.916 0.888 0.884 0.879 0.856 0.628 0.609 0.591
patel 4700201/fid
OH
Et
Me
4.14 1.10 1.00 0.99 1.00 1.95 2.22 1.37 3.15 2.13 3.09 1.33 3.03 3.04
11.0 10.5 10.0 9.5 9.0 8.5 8.0 7.5 7.0 6.5 6.0 5.5 5.0 4.5 4.0 3.5 3.0 2.5 2.0 1.5 1.0 0.5 0.0 -0.5
f1 (ppm)

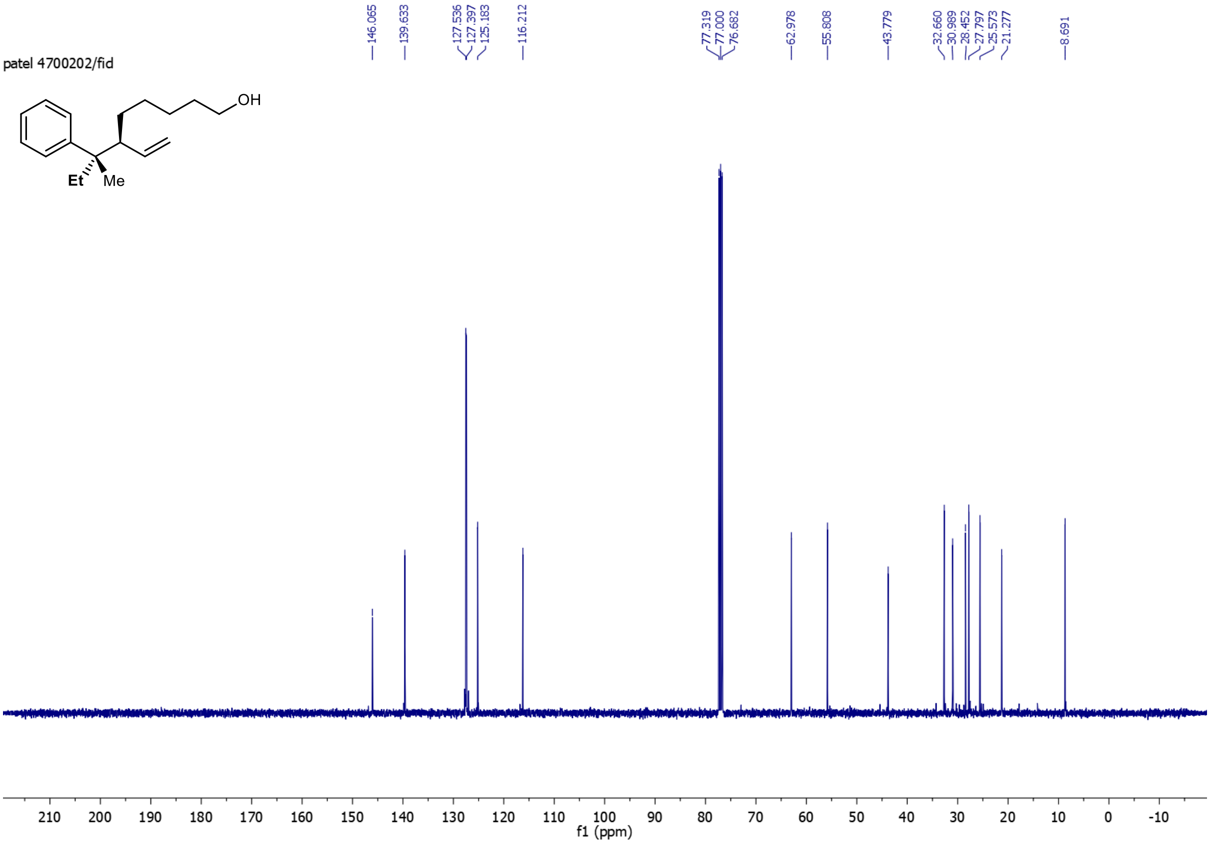
146.065 139.633 127.536 127.397 125.183 116.212 77.319 77.000 76.682 62.978 55.808 43.779 32.660 30.989 28.452 27.797 25.573 21.277 8.691
patel 4700202/fid
OH
Et
Me
210 200 190 180 170 160 150 140 130 120 110 100 90 80 70 60 50 40 30 20 10 0 -10
f1 (ppm)

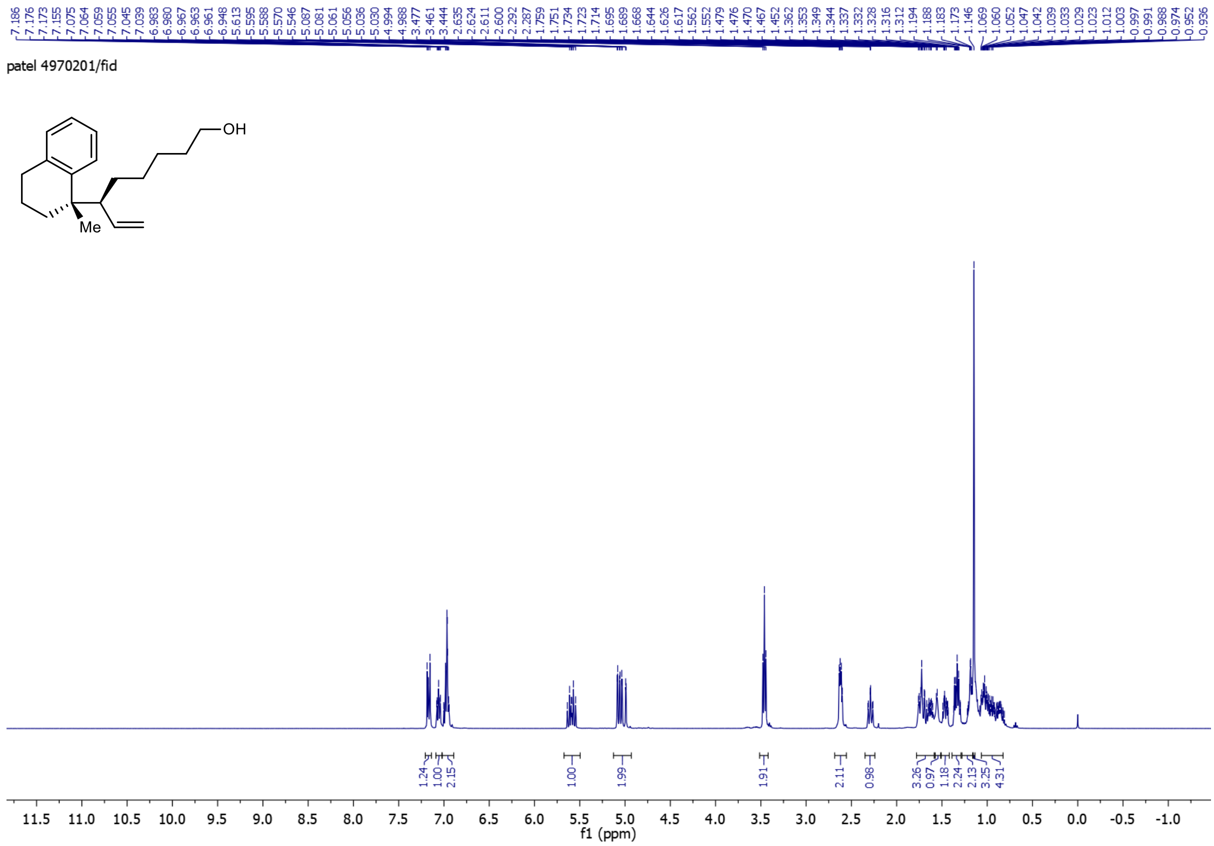
patel 4970201/fid
OH
Me
f1 (ppm)

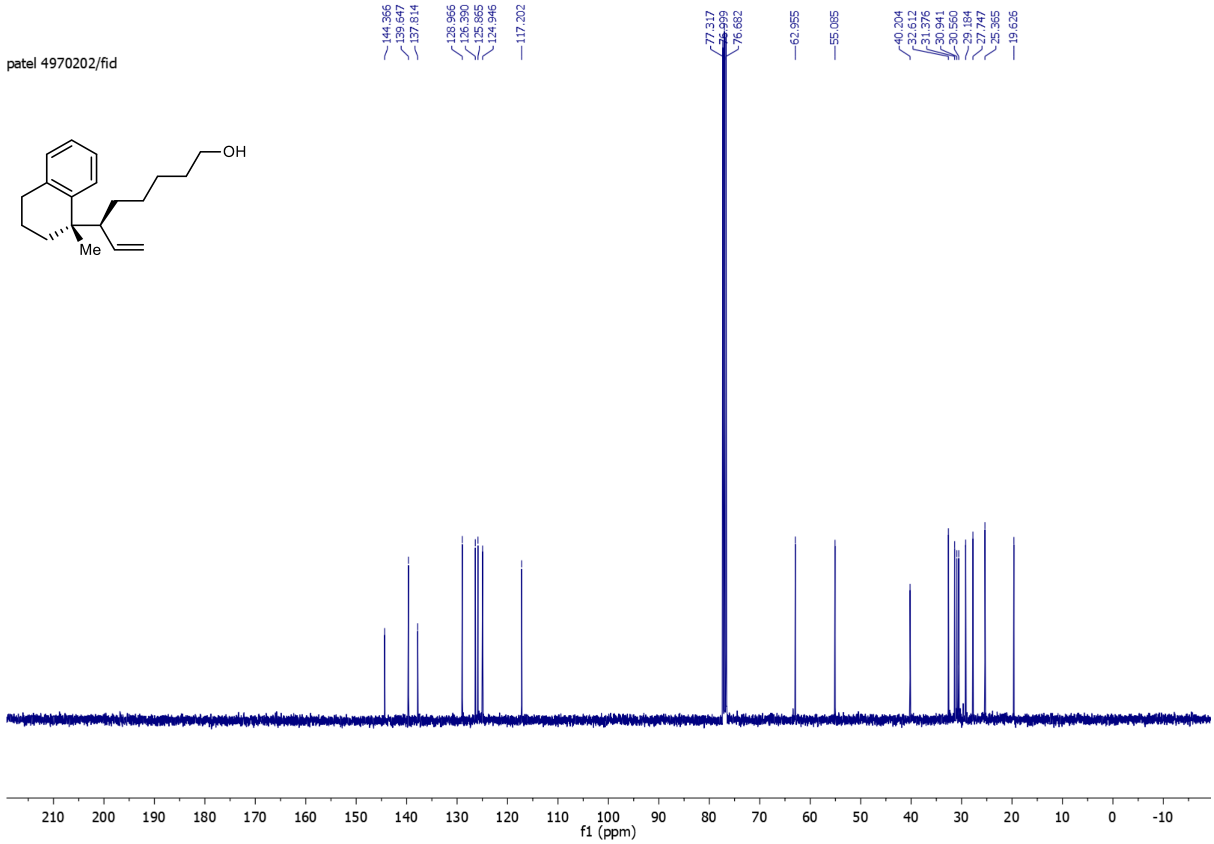
patel 4970202/fid
OH
Me
f1 (ppm)

References

(1) (a) M. Simaan, I. Marek, Asymmetric catalytic preparation of diastereo- and enantiomerically enriched polysubstituted cyclopropanol and cyclopropylamine derivatives. *Angew. Chem. Int. Ed.* **2018**, 57, 1543-1546. (b) M. Simaan, I. Marek, Diastereo- and enantioselective preparation of cyclopropanol and cyclopropylamine derivatives. *Beilstein J. Org. Chem.* **2019**, 15, 752-760. (c) L. Dian, I. Marek, Asymmetric Rh-catalyzed arylation of cyclopropenes based on directed functionalization of three-membered carbocycles (DFTMC). *Angew. Chem. Int. Ed.* **2018**, 57, 3682-3686.

(2) M. Cormier, A. de la Torre, I. Marek, Diastereoselective ring-opening of alkenylcyclopropanes: application to the total synthesis of c30 botryococcene and epi-botryococcene. *Angew. Chem. Int. Ed*. **2018**, *58*, 13237-13241.

(3) (a) Cohen, Y.; Marek, I. Directed regioselective carbometallation of 1,2-dialkyl-substituted cyclopropenes. *Angew. Chem. Int. Ed.* **2021**, *60*, 26368-26372. (b) Cohen, Y.; Augustin, A. U.; Levy, L.; Jones, P. G.; Werz, D. B.; Marek, I. Regio- and diastereoselective copper-catalyzed carbomagnesiation for the synthesis of penta- and hexa-substituted cyclopropanes. *Angew. Chem. Int. Ed*. **2021**, *60*, 11804-11808.

(4) Cohen, Y.; Cohen, A.; Marek, I. Creating stereocenters within acyclic systems by C-C bond cleavage of cyclopropanes. *Chem. Rev*. **2021**, *121*, 140-161.

(5) (a) Lebel, H.; Marcoux, J.-F.; Molinaro, C.; Charette, A. B. Stereoselective cyclopropanation reactions. *Chem. Rev*. **2003**, *103*, 977-1050. (b) Lacasse, M.-C.; Poulard, C.; Charette, A. B. Iodomethylzinc phosphates: powerful reagents for the cyclopropanation of alkenes. *J. Am. Chem. Soc.* **2005**, *127*, 12440-12441.

(6) D. A. L. Otte, D. E. Borchmann, C. Lin, M. Weck, & K. A. Woerpel, ^{13}C nmr spectroscopy for the quantitative determination of compound ratios and polymer end groups. *Organic Letters* **2015**, 17 (15), 3906-3909.

(7) A. T. Tran, V. A. Huynh, E. M. Friz, S. K. Whitney, D. B. Cordes, A general method for the rapid reduction of alkenes and alkynes using sodium borohydride, acetic acid, and palladium. *Tetrahedron Lett.* **2009**, *50*, 1817-1819.

(8) Y. Yamamoto, R. Fujikawa, T. Umemoto, N. Miyaura, Iridium-catalyzed hydroboration of alkenes with pinacolborane. *Tetrahedron* **2004**, *60*, 10695-10700.
